# Genome-wide identification and analysis of the *COI* gene family in wheat (*Triticum aestivum* L.)

**DOI:** 10.1186/s12864-018-5116-9

**Published:** 2018-10-17

**Authors:** Jian-fang Bai, Yu-kun Wang, Peng Wang, Shao-hua Yuan, Jian-gang Gao, Wen-jing Duan, Na Wang, Feng-ting Zhang, Wen-jie Zhang, Meng-ying Qin, Chang-ping Zhao, Li-ping Zhang

**Affiliations:** 1 Beijing Engineering and Technique Research Center for Hybrid Wheat, Beijing Academy of Agriculture and Forestry, Beijing, 100097 China; 2 The Municipal Key Laboratory of Molecular Genetic of Hybrid Wheat, Beijing, 10097 China; 3 Nara Institute of Science and Technology, 8916-5 Takayama, Ikoma, Nara, 630-0192 Japan

**Keywords:** *Triticum aestivum* L., COI, Gene family, JA signaling, Male sterile, Quantitative real-time PCR

## Abstract

**Background:**

COI (CORONATINE INSENSITIVE), an F-box component of the Skp1-Cullin-F-box protein (SCF^COI1^) ubiquitin E3 ligase, plays important roles in the regulation of plant growth and development. Recent studies have shown that *COIs* are involved in pollen fertility. In this study, we identified and characterized *COI* genes in the wheat genome and analyzed expression patterns under abiotic stress.

**Results:**

A total of 18 *COI* candidate sequences for 8 members of *COI* gene family were isolated in wheat (*Triticum aestivum* L.). Phylogenetic and structural analyses showed that these *COI* genes could be divided into seven distinct subfamilies. The *COI* genes showed high expression in stamens and glumes. The qRT-PCR results revealed that wheat *COIs* were involved in several abiotic stress responses and anther/glume dehiscence in the photoperiod-temperature sensitive genic male sterile (PTGMS) wheat line BS366.

**Conclusions:**

The structural characteristics and expression patterns of the *COI* gene family in wheat as well as the stress-responsive and differential tissue-specific expression profiles of each *TaCOI* gene were examined in PTGMS wheat line BS366. In addition, we examined SA- and MeJA-induced gene expression in the wheat anther and glume to investigate the role of COI in the JA signaling pathway, involved in the regulation of abnormal anther dehiscence in the PTGMS wheat line. The results of this study contribute novel and detailed information about the *TaCOI* gene family in wheat and could be used as a benchmark for future studies of the molecular mechanisms of PTGMS in other crops.

**Electronic supplementary material:**

The online version of this article (10.1186/s12864-018-5116-9) contains supplementary material, which is available to authorized users.

## Background

Jasmonates (JAs), including jasmonic acid (JA), methyl jasmonate (MeJA), and its derivatives, play important roles in the regulation of biotic and abiotic stresses as well as plant growth, development, and defense [1–4]. JA is also involved in the regulation of floral organ development, such as pollen maturation, anther dehiscence, and male fertility [5–8]. JA biosynthesis gene-deficient mutants (*fad* [9], *opr3* [8, 10], *dde1* [11], *dad1* [12], and *aos* [13]) show abnormal anther growth and development in many plants. In rice, plants with mutant JA-amino acid synthetase *osjar1–2* and *osjar1–3* are insensitive to JA signaling, and the dehiscence of anthers filled with viable pollen is impaired, resulting in pollen sterility [14, 15]. In addition, JA plays a crucial role in sex determination during male flower development in maize [16].

JA signaling is mediated by CORONATINE INSENSITIVE 1 (COI1), which is an F-box component of the Skp1-Cullin-F-box protein (SCF^COI1^) ubiquitin E3 ligase [17, 18]. The SCF^COI1^ complex is predicted to target repressors of JA signaling to the 26S proteasome for degradation in *Arabidopsis* [19, 20]. In *Arabidopsis*, a single *COI* (At2939940) and 12 members of the *JAZ* family have been characterized. An *Arabidopsis coi1* mutant exhibits a male sterile phenotype, including the inhibition of filament elongation and anther non-dehiscence [21]. *COI* is also involved in leaf senescence [22–25], apical dominance [26], inositol polyphosphates [27], and ethylene-induced root growth inhibition in the light in *Arabidopsis thaliana* [28]. In *Arabidopsis*, at least four of the 12 members of the *AtJAZ* family (JAZs*1*, *3, 9,* and *10*) interact with *COI* in a JA-Ile (jasmonoyl isoleucine)- or coronatine-dependent manner [29–33]. In *Solanum nigrum*, *COI1* controls jasmonate metabolism and the production of a systemic signal against insect attacks [34]. The rice genome has three *COI* homologs, i.e., *OsCOI1a* (Os01g0853400; AK121543), *OsCOI1b* (Os05g0449500; AK101514), and *OsCOI2* (Os03g0265500; AK100694). OsCOI1a and OsCOI1b share amino acid sequence identities of greater than 80%, and these loci share an identity of 63% with OsCOI2 [35]. *OsCOI1a* regulates *OsbHLH148* expression in response to coronatine by forming an SCF^COI1^ complex [36]. *OsCOI1b* is involved in leaf senescence [35]. Moreover, in addition to a MeJA-insensitive phenotype, *OsCOI1* RNAi lines show an altered plant height, internode length, and grain length and increased susceptibility to chewing insects [37, 38].

Wheat (*Triticum aestivum* L.) is an important food crop worldwide. Plant fertility in photoperiod-temperature sensitive genic male sterile (PTGMS) lines is controlled by temperature and/or photoperiod; thus, the PTGMS line can be used as both a maintenance line and a male-sterile line for exploiting heterosis [39–42]. The discovery and application of PTGMS wheat series provided the basis for two-line hybrid wheat. The two-line hybrid system was quickly applied to wheat production owing to the potential for simple and low-cost seed production. Recently, it has been reported that male sterility of the female parent can be attributed to lack of anther development or dehiscence in the two line hybrid wheat system [43]. Our early research indicated that the anther of the PTGMS wheat line BS366 does not fully spill out, showing impaired anther dehiscence. The defective phenotype can be recovered by the application of MeJA in vitro [7]. Wheat genome sequencing is nearing completion (http://www.wheatgenome.org/), providing basic data for whole genome research and the identification of functional genes [44–47]. In wheat, the JA family is involved in anther dehiscence and male sterility [7, 48]. However, there are few studies of *COI* in the wheat jasmonic acid regulatory pathway. In this study, we first systematically identified and characterized the *COI* genes of wheat (*TaCOI*)*,* and analyzed its architectural features, evolutionary history, and expression patterns in anther tissues in the PTGMS wheat line. In addition, the expression profiles of *TaCOI* genes in different tissues as well in response to several stresses were analyzed. Finally, the inducible expression of wheat *COI* genes was detected by qRT-PCR, and some *COIs* clearly responded to abiotic stress. The anther and glume dehiscence and the expression of *TaCOI* genes after treatment with MeJA and interactions between MeJA and SA were also analyzed to determine the role of *COI* in JA-mediated signaling pathways in anther dehiscence and male sterility.

## Methods

### Plant materials, growth conditions, and sample collection

The wheat (*Triticum aestivum* L.) PTGMS line BS366 (winter wheat) was used. The following treatments were evaluated. All plants were planted in experimental fields in Beijing (China, N 39°54′, E 116°18′) and managed conventionally.

1. Hormone cross-stimulation. After the four-leaf stage [42, 49], plants were transferred to an artificial climate incubator (CLC-BIV-M/CLC404-TV, MMM, Germany) at 20 °C (with 12-h day/12-h night) and a relative humidity of 60–80% for the entire reproductive period. After transferring samples to the artificial climate incubator, they will be incubated for about two weeks until head period [7, 48]. Then, the spikelets were sprayed with 0.5 mM, 2 mM, and 4 mM MeJA for each group at 20 °C (with 12-h day/12-h night) every day for 5 days. For the SA and MeJA interaction treatment, 0.5 mM, 2 mM, and 4 mM MeJA were applied every day for 5 days when spikelets were treated with 10 mM SA (20 °C, 12-h day/12-h night) in the artificial climate incubator. The glumes and anthers were collected for further analysis. Glume and anther dehiscence were detected using a dissecting microscope (Olympus SZX12; Tokyo, Japan).
2. Abiotic stresses. Two-week-old wheat seedlings (20 °C, 12-h day/12-h night cycle) were used for stress treatments. BS366 seedlings were sprayed with 2 mM SA, 100 mM MeJA, 100 mM GA_3_, 50 mM IAA, and 100 mM ABA. The control plants were treated with 0.1% (v/v) ethanol. For the high-salinity and drought treatments, the roots of wheat seedlings were soaked in 200 mM NaCl and PEG6000 (− 0.5 MPa). For low temperature (cold) stress, the seedlings were moved to an incubator at 10 °C (with a 12-h day/12-h night cycle) and a relative humidity of 60–80%. The leaf tissues from seedlings were collected at 0, 2, 4, 8, 12, and 24 h post-treatment.
3. Samples (root, stem, leaf, glume, stamen, and pistil) planted in beijing, were collected for tissue-specific expression analyses at the heading stage [7, 48].
4. Fertiliy analysis. The PTGMS wheat line BS366 shows thermo-photoperiod sensitivity in pollen fertiliy [49]. It shows anther indehiscent and pollen sterility (seed setting rate < 5%) in sterile condition (10 °C with 12-h day/12-h night for daily mean temperature during pollen development stages), but exhibits anther dehiscent and pollen fertility (40% < Seed setting rate < 55%) in fertile condition (20 °C with 12-h day/12-h night for daily mean temperature during pollen development stages) [42, 43, 48, 49]. In this part of the experiment, after the four-leaf stage [42, 49], plants were grouped two and transferred to artificial climate incubators at 20 °C and 10 °C (with 12-h day/12-h night), respectively, and a relative humidity of 60–80% for the entire reproductive period. Based on the leaf age index and anther length, spikelets for stage-specific expression analyses were collected from two fertility conditions for the PTGMS line BS366 at three different pollen developmental stages, which are stage 1: secondary sporogenous cells had formed; stage 2: all cell layers were present and mitosis had ceased, and stage 3: meiotic division stage [42, 49].

All samples were rapidly frozen in liquid nitrogen and stored in a − 80 °C freezer until RNA extraction.

### Sources of sequence data

The COI protein sequences of *Triticum aestivum* L. (*T. aestivum,* Ta), *Physcomitrella patens* (Pp), *Populus trichocarpa* (Pt), *Arabidopsis thaliana* (At), *Sorghum bicolor* (Sb), *Zea mays* (Zm), *Oryza sativa* (Os), and *Brachypodium distachyon* (Bd) were obtained from the JGI database (http://genome.jgipsf.org/). *Selaginella moellendorffii* (Sm), *Aegilops tauschii* (Aet), *Triticum urartu* (Tu) and *Hordeum vulgare* L. (Hv) sequences were acquired from the Ensembl Plants database (http://plants.ensembl.org/index.html). All sequence information is provided in Additional file 2: Table S1.

### Identification of *COI* gene family members in wheat

COI protein sequences were downloaded from the PLAZA database (http://hmmer.org/, ID: ORTHO001097) [50]. The hidden Markov model (HMM) profiles were constructed using the hmmbuild procedure (HMMER3.0) (http://hmmer.org/) for comparisons of the COI conserved protein structure domain in the wheat protein database (E-value ≤1.0E-10). Then, the *COI* gene family members were confirmed after removing non-COI conserved domains and repeats using the CDD (Conserved Domain Database) of NCBI and the SMART web server (http://smart.embl-heidelberg.de/). COI subgenomic copies were named A, B, and D according to their positions on chromosomes.

### Analyses of gene characteristics

*COI* structures were analyzed using the Gene Structure Display Server (GSDS) program (http://gsds.cbi.pku.edu.cn/). Protein properties, including the relative molecular weight (MW) and isoelectric point (PI), were predicted using ExPASy (http://www.expasy.org/). WoLF PSORT (http://www.genscript.com/wolf-psort.html) was used for *TaCOI* gene family member subcellular localization predictions. Multiple Expectation Maximization for Motif Elicitation (MEME) was used to identify the motifs of COI proteins, with default settings. To analyze putative *cis*-acting elements in a promoter region, 2-kb promoter regions were selected and screened against the Plant CARE database (http://bioinformatics.psb.ugent.be/webtools/plantcare/html/). The sequences of the *COI* promoters are listed in Additional file 2: Table S3.

### Multiple sequence alignment and phylogenetic tree construction

To determine the roles of COIs in the JA signaling pathway, a multiple sequence alignment of the amino acid sequences of COI in selected plant genomes was generated using DNAMAN (ver. 6.0) with default settings. Subsequently, MEGA 6.0 was used to construct an unrooted phylogenetic tree based with the neighbor-joining method (with 1000 bootstrap replicates).

### Chromosomal locations analysis

To map the corresponding *COI* loci on *T. aestivum*, *T. urartu*, *Ae. Tauschii,* and *B. distachyon* chromosomes, the genome annotation files for these plants were obtained from the JGI database (https://genome.jgi.doe.gov/portal/) and Ensembl Plants database (http://plants.ensembl.org/index.html). To detect synteny of the *COI* genes in *T aestivum* and selected plants, whole genome synteny block data were collected from the Plant Genome Duplication Database (http://chibba.agtec.uga.edu/duplication/) [51]. Chromosomal locations for the *COI* family genes were examined with default parameters to determine duplications and synteny relationships using Circos-0.69 (http://circos.ca/) [52].

### Total RNA extraction, cDNA synthesis and qRT-PCR analysis

Total RNA was isolated for each sample using TRIzol Reagent (Invitrogen, Nottingham, UK) according to the manufacturer’s instructions. The purified RNA was stored at − 80 °C until subsequent analyses.

First-strand cDNA synthesis was performed using M-MLV Reverse Transcriptase according to the manufacturer’s instructions (Takara, Shiga, Japan). Quantitative real-time PCR (qRT-PCR) was performed using a SYBR Premix Ex Taq Kit (Takara) and a real-time PCR machine (CFX96; Bio-Rad, Hercules, CA, USA), following the manufacturer’s instructions. The procedure used for qRT-PCR was 10 min at 95 °C, followed by 38 cycles of 15 s at 95 °C and 60 s at 61–62 °C. *β-Actin* was used as a reference gene for the mRNA relative expression patterns analysis. The reactions were performed with three biological replicates and at least two technical replicates per sample. The data were analyzed using the 2^-∆∆Ct^ method [53] and means ± standard errors (SE) of three biological replicates are presented. The primers for qRT-PCR are listed in Additional file 2: Table S2.

## Results

### Identification, phylogenetic analysis, and classification of wheat *COI* genes

After removing redundant reads, 18 COI candidate protein sequences were obtained by HMM searches against the *T. aestivum* genome sequence. These 18 COI protein sequences were clustered into eight groups based on high similarity (≥95%). These eight groups were referred to as *TaCOI1*–*TaCOI8* according to naming conventions. In addition, These *COI* genes were distinguished by their positions in the wheat sub-genome (A, B, and D). Gene information is summarized in Table 1. To understand the role of gene functions, subcellular localization prediction for *TaCOI* family using WoLE PSORT (http://www.genscript.com/wolf-psort.html) was performed. It was showed that these *TaCOI* genes exhibited cytoplasmic, nuclear, and chloroplast localization. *TaCOI-A*, *TaCOI2-A*, *TaCOI2-B, TaCOI3-B, TaCOI3-D, TaCOI4-D, TaCOI6-A, TaCOI6-B, TaCOI6-D,* and *TaCOI8-A* were located in cytoplasmic fractions. *TaCOI4-A* and *TaCOI4-B* were located in the cytoplasm and nuclei. The remaining genes were located in the cytoplasm and chloroplasts. Moreover, the molecular weights (MW) and isoelectric points (pI) of TaCOI proteins were calculated using the Protparam tool in ExPASy. The MW of COIs varied from 20.39 to 66.36 kDa, and the pIs ranged from 5.41 to 8.79 (Table 1). Different copies of the same TaCOI showed different physicochemical properties, such as *TaCOI3-B* owing 3 times MW than that of *TaCOI4-A*.

**Table 1:** Characteristics of *TaCOI* gene family members from wheat

Gene name	Sequence ID	Location	Protein/AA	Molecular weight of decuced protein/KD	Isoelectric point	Subcellular Localization
*TaCOI1-A*	Traes_1AS_1E22B5174.1	1A:10791781:10793235	484	54.29	6.07	Cytoplasm
*TaCOI2-A*	Traes_2AL_9EC359B65.2	2A:38684891:38689283	574	63.99	5.83	Cytoplasm
*TaCOI2-B*	Traes_2BL_370AE211F.1	2B:111631663:111636013	574	64.21	6.25	Cytoplasm
*TaCOI3-B*	TRAES3BF018800040CFD_t1	3B:443238454:443241608	594	66.36	6.58	Cytoplasm
*TaCOI3-D*	Traes_3DL_845220DFC.1	3D:93268132:93270118	184	20.39	4.94	Cytoplasm
*TaCOI4-A*	Traes_3AL_2240B0C5F.1	3A:163966748:163968217	191	20.75	6.91	Cytoplasm Nuclear
*TaCOI4-B*	TRAES3BF021600080CFD_t1	3B:655287004:655289230	381	40.87	6.83	Cytoplasm Nuclear
*TaCOI4-D*	Traes_3DL_3DB9F1EC5.1	3D:99011996:99014454	284	30.53	5.86	Cytoplasm
*TaCOI5-A*	Traes_4AS_AD3F2B991.1	4A:48344426:48347184	575	64.29	5.96	Cytoplasm Chloroplast
*TaCOI5-B*	Traes_4BL_499E7F095.1	4B:243711227:243714109	586	65.60	5.95	Cytoplasm Chloroplast
*TaCOI5-D*	Traes_4DL_8C2C2ADD4.1	4D:19191547:19194445	586	65.57	6.01	Cytoplasm Chloroplast
*TaCOI6-A*	Traes_5AL_27F194099.2	5A:77415870:77422532	569	62.09	8.79	Cytoplasm
*TaCOI6-B*	Traes_5BL_7AE04C6B2.1	5B:189453532:189459589	572	62.14	8.57	Cytoplasm
*TaCOI6-D*	Traes_5DL_4E8DD7C5F.3	5D:108986194:108992606	569	62.07	8.62	Cytoplasm
*TaCOI7-A*	Traes_6AL_0F53490CA.1	6A:185429448:185432194	343	37.63	5.75	Cytoplasm Chloroplast
*TaCOI7-B*	Traes_6BL_9E82CDBD1.1	6B:162540302:162543336	429	47.81	5.41	Cytoplasm Chloroplast
*TaCOI7-D*	Traes_6DL_AD0DAD6D1.2	6D:140761611:140765528	343	37.54	5.60	Cytoplasm Chloroplast
*TaCOI8-A*	Traes_7AS_78FEAE00C.1	7A:31411042:31413175	387	42.98	5.75	Cytoplasm

### Phylogenetic analysis

**Fig. 1:**
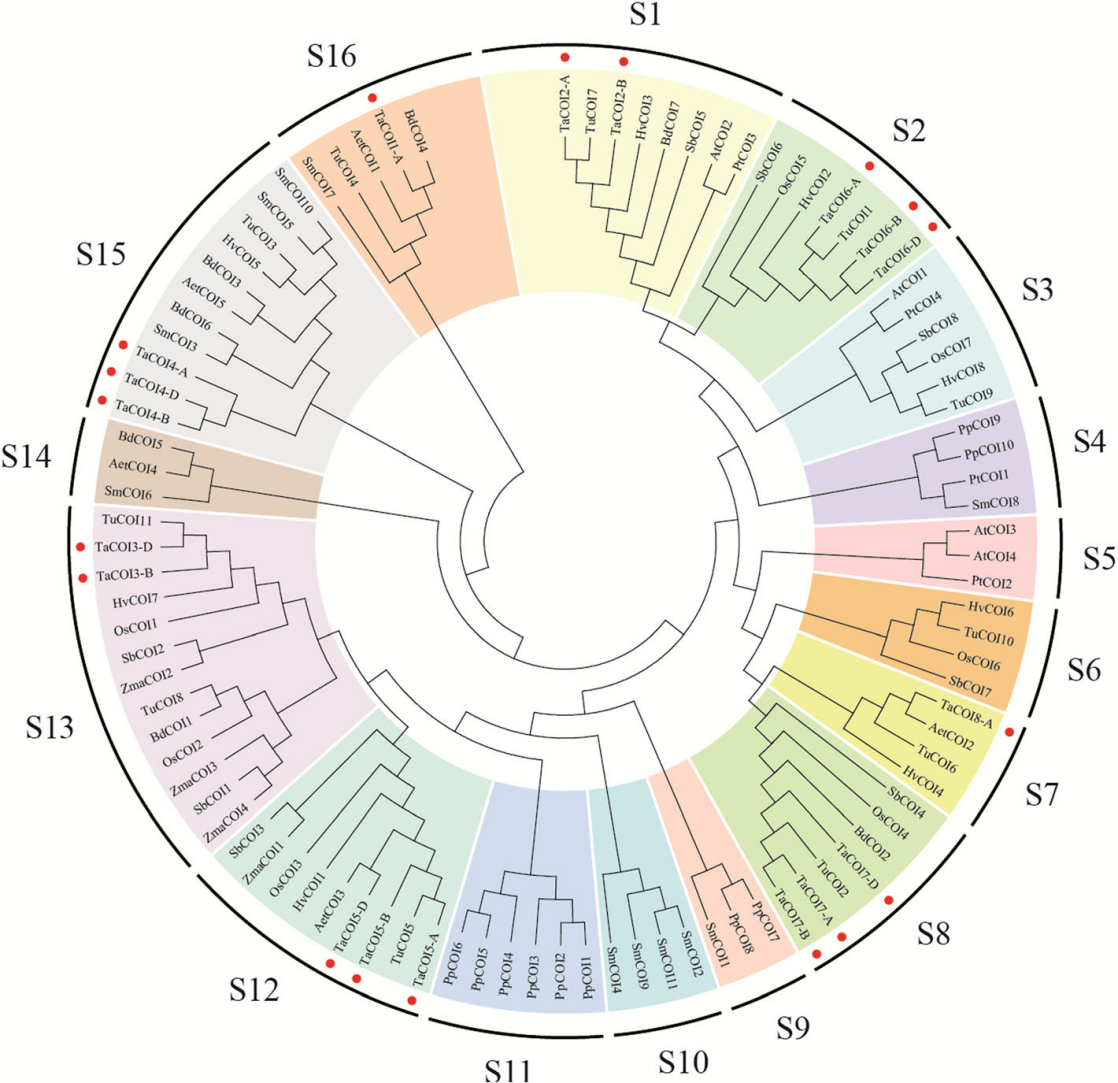
Bio Neighbor-Joining (BioNJ) phylogenetic tree (1000 bootstrap replicates), based on a protein alignment of COIs from *T. aestivum* (Ta), *P. patens* (Pp), *P. trichocarpa* (Pt), *A. thaliana* (At), *S. bicolor* (Sb), *Z. mays* (Zm), *O. sativa* (Os)*, B. distachyon* (Bd), *S. moellendorffii* (Sm), *Ae. tauschii* (Aet), *T. urartu* (Tu), and *H. vulgare* (Hv). Each TaCOI protein is indicated by a red dot

To study the phylogeny of COI proteins, a neighbor-joining phylogenetic tree was built using MEGA 6.0 with default parameters. As shown in Fig. 1, COI proteins were clustered into 16 sub-groups, S1–S16, and the 18 protein sequences from eight wheat TaCOI family members clustered into 8 sub-groups (Fig. 1). TaCOI1-A, TaCOI2 (TaCOI2-A and TaCOI2-B), TaCOI3 (TaCOI3-B and TaCOI3-D), TaCOI4 (TaCOI4-A, TaCOI4-B, and TaCOI4-D), TaCOI5 (TaCOI5-A, TaCOI5-B, and TaCOI5-D), TaCOI6 (TaCOI6-A and TaCOI6-B), TaCOI7 (TaCOI7-A, TaCOI7-B, and TaCOI7-D), and TaCOI8-A clustered into groups S16, S1, S12, S14, S11, S2, S8, and S7, respectively. The COI proteins clustered into the same clades with some OsCOI, SbCOI, BdCOI, PtCOI, AtCOI, and AetCOI proteins, indicating that the TaCOI proteins share high homology with the COI proteins of other plants (Fig. 1). Furthermore, we found that COI proteins from same species clustered into different clades. For example, PpCOI proteins were clustered into S4, S10, and S14, and SmCOI proteins were clustered into S4, S9, S13 S 14, S15, and S16, revealing that COI gene exhibited differences in evolution among species.

### Analysis of stress response-related *cis*-regulatory elements in the promoter regions of *TaCOI* genes

**Fig. 2:**
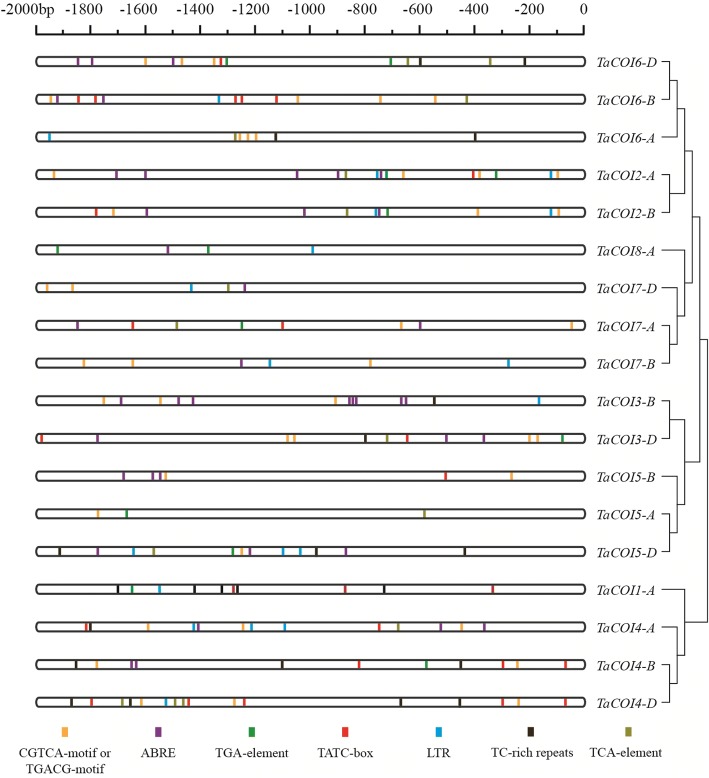
Analysis of specific cis-elements in promoters. The 2 k-bp promoter sequences of corresponding *COI* genes were used to analyze five specific hormone-related *cis*-elements, including ABRE, TGA-element, TATC-box, CGTCA/TGACG-motif, TCA-element which respond to ABA, auxin (IAA), gibberellin (GA), methyl jasmonate (MeJA), salicylic acid (SA) and two stress-responsive regulatory elements, TC-rich repeats and LTR which respond to stress/defence and low temperature, which are color-coded

In plants, gene transcription is regulated by *cis*-acting regulatory elements that bind to target transcription factors [54]. Some *cis*-regulatory elements are involved in stress responses, such as dehydration and cold responses (DRE/CRT) [55], ABA responsive element (ABRE) [56], ARF binding sites (AuxRE) [57], and SA-responsive promoter elements (SARE) [58, 59]. To analyze how the expression levels of *TaCOI* genes responded to stress stimuli, 2.0-kb upstream promoter regions of *TaCOI* genes were scanned for stress-related cis-regulatory elements using the Plant CARE online service (http://bioinformatics.psb.ugent.be/webtools/plantcare/html/) [18]. As shown in Fig. 2 and Table 3, five hormone-responsive regulatory elements, ABRE, TGA-element, TATC-box, CGTCA/TGACG-motif, and TCA-element, associated with ABA, auxin (IAA), gibberellin (GA), methyl jasmonate (MeJA), and salicylic acid (SA) responses, were identified in the promotor region of *TaCOIs*. Additionally, two stress-responsive regulatory elements, TC-rich repeats and LTR, associated with defense/stress and low-temperature responses, respectively, were identified in the *TaCOI* promoter regions. Different types and numbers of regulatory elements were present in the distinct *TaCOI* promoters (Table 2), indicating that *TaCOI* genes might be involved in the response to various stress and hormone treatments via participating different regulatory mechanisms. The CGTCA/TGACG-motif, which was associated with a methyl jasmonate (MeJA) responsive element, was enriched in the promoters of *TaCOI* genes (Fig. 2, Additional file 1: Figure S1). Other than *TaCOI1-A* and *TaCOI8-A*, *TaCOIs* contained two or more CGTCA/TGACG-motifs; for example, *TaCOI6-D* contained three CGTCA/TGACG-motifs and *TaCOI3-D* contained four CGTCA/TGACG-motifs. It was also found that ABRE-motif was also enriched in the promoters of most of all *TaCOI* genes (Fig. 2 and Table 2). The numbers and types of stress-related *cis*-elements in the 2.0-kb upstream regions of *TaCOI* family genes are listed in Table 2 and Table 3.

**Table 2:** The number and composition of *cis*- acting regulatory elements of each *TaCOI* gene

Gene	CGTCA-motif or TGACG-motif (MeJA)	ABRE (ABA)	TGA-element (IAA)	TATC-box (GA)	LTR (Cold)	TC-rich repeats (Defense/stress)	TCA-element (SA)
*TaCOI1-A*			1	3	1	5	
*TaCOI2-A*	4	5	2	1	2		1
*TaCOI2-B*	3	3	1	1	2		1
*TaCOI3-B*	3	8			1	1	
*TaCOI3-D*	4	3	1	2		1	1
*TaCOI4-A*	3	3	2		3	1	1
*TaCOI4-B*	2	2	1	3		3	
*TaCOI4-D*	3			5	1	4	3
*TaCOI5-A*	1		1				1
*TaCOI5-B*	2	3		1			
*TaCOI5-D*	1	3	1		3	3	1
*TaCOI6-A*	3		1		1	2	
*TaCOI6-B*	4	2		5	1		1
*TaCOI6-D*	3	3	2	1		2	2
*TaCOI7-A*	2	2	1	2			1
*TaCOI7-B*	3	1			2		
*TaCOI7-D*	2	1			1		1
*TaCOI8-A*		1	2		1

**Table 3:** Functions of *cis*- acting regulatory elements in promoter regions of *TaCOI* genes

Site Name	Organism	sequence	function
TC-rich repeats	*Nicotiana tabacum*	GTTTTCTTAC	cis-acting element involved in defense and stress responsiveness
TCA-element	*Nicotiana tabacum*	CCATCTTTTT	cis-acting element involved in salicylic acid responsiveness
CGTCA-motif	*Hordeum vulgare*	CGTCA	cis-acting regulatory element involved in the MeJA-responsiveness
TGACG-motif	*Hordeum vulgare*	TGACG	cis-acting regulatory element involved in the MeJA-responsiveness
LTR	*Hordeum vulgare*	CCGAAA	cis-acting element involved in low-temperature responsiveness
TATC-box	*Oryza sativa*	TATCCCA	cis-acting element involved in gibberellin-responsiveness
ABRE	*Arabidopsis thaliana*	TACGTG	cis-acting element involved in the abscisic acid responsiveness
TGA-element	*Brassica oleracea*	AACGAC	auxin-responsive element

### Structural analysis of wheat *COI* genes and proteins

**Fig. 3:**
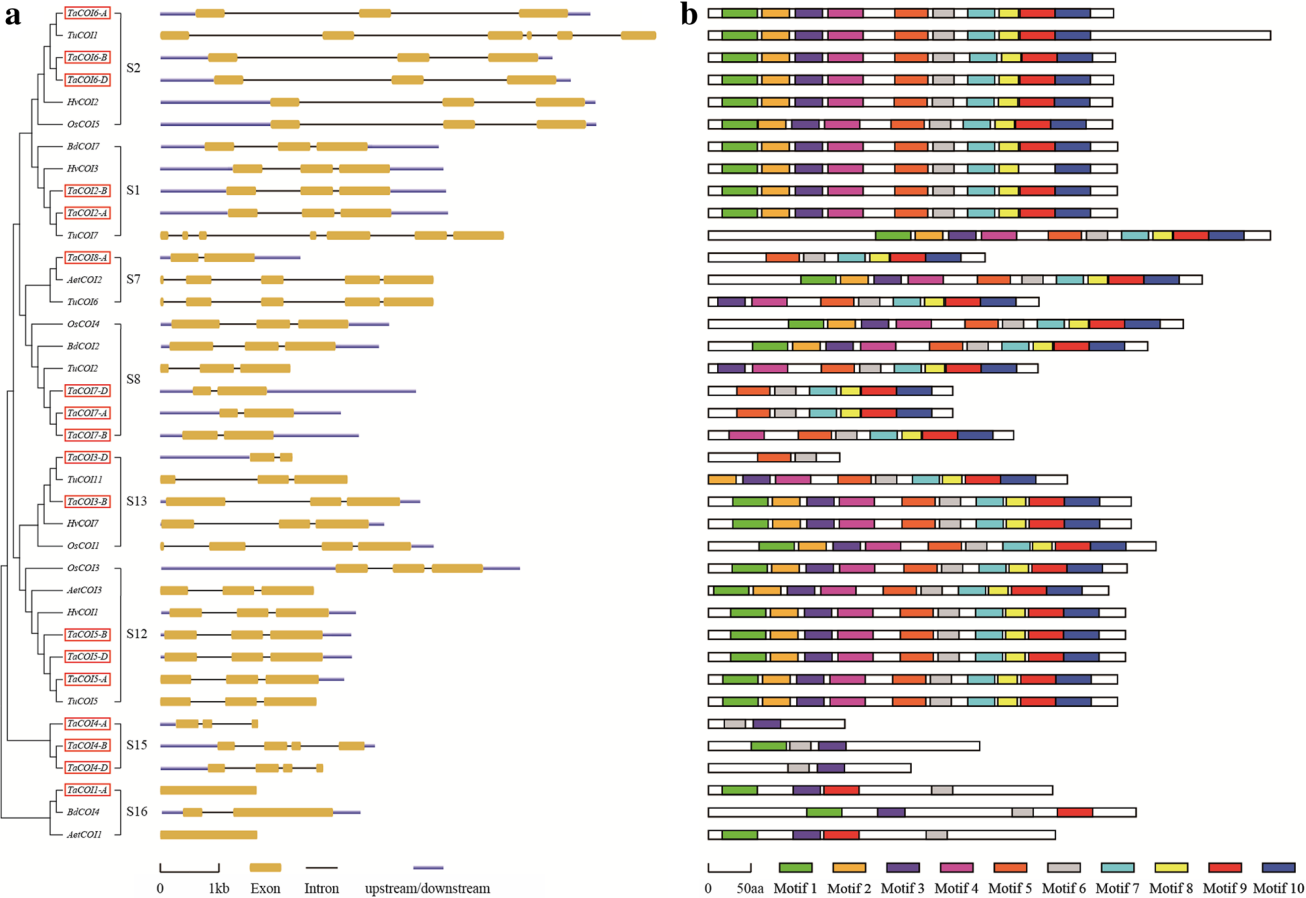
Exon-intron structures (**a**) of the *COI* genes in *T. aestivum* (Ta), *O. sativa* (Os)*, B. distachyon* (Bd), *T. urartu* (Tu), and *H. vulgare* (Hv) and a motif distribution analysis (**b**). Exons are shown as yellow boxes, introns are shown as thin lines, and UTRs are shown as blue lines. The sizes of exons and introns can be estimated using the scale below. Ten motif types are shown as colored boxes

**Fig. 4:**
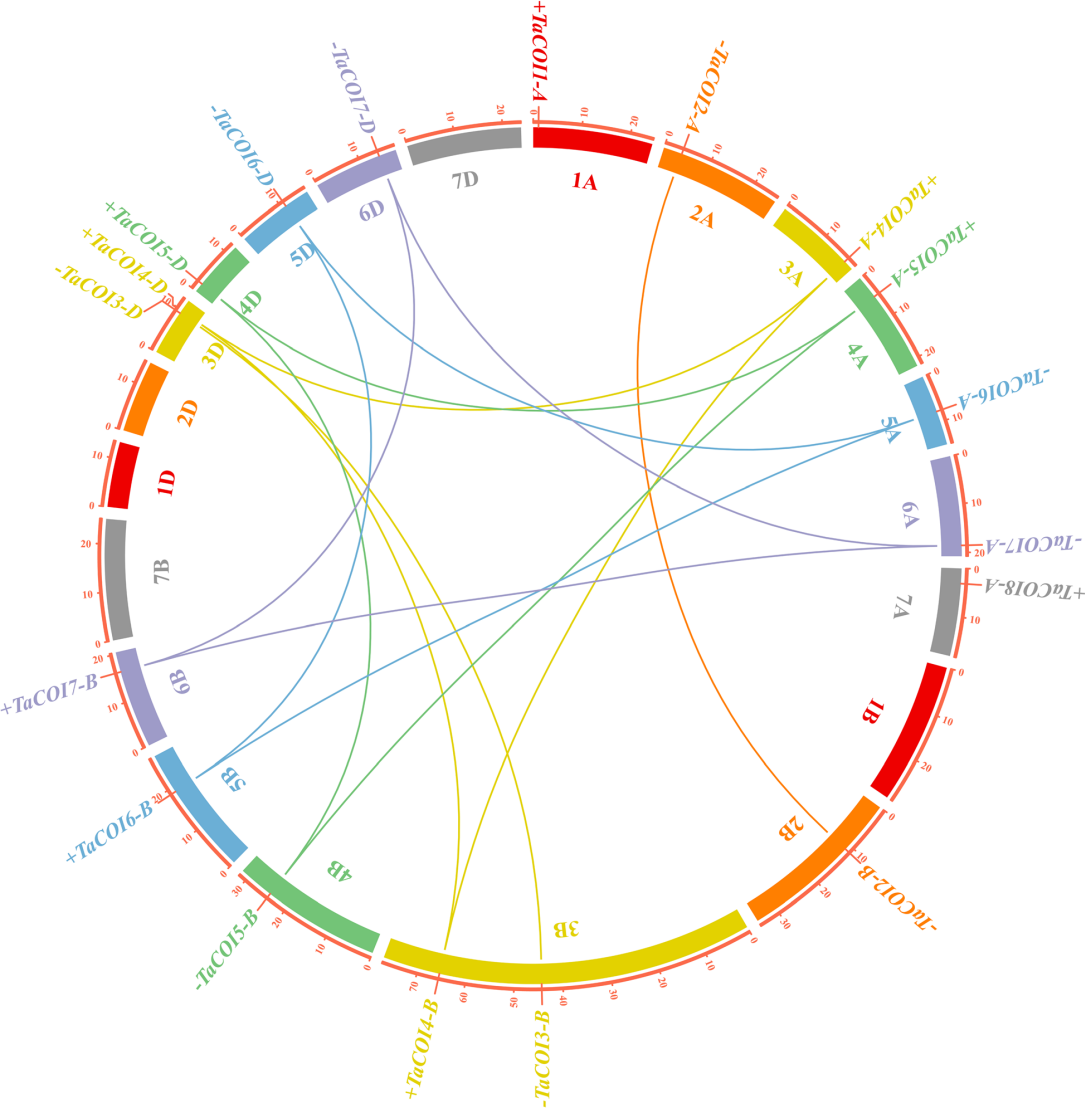
Chromosomal localizations and syntenic relationships among *TaCOI* genes in wheat. The positions of *TaCOI* genes are marked directly on chromosomes. “+” and “-” in front of gene names mean sense and anti-sense strand of genome

To further understand the function of *TaCOI* genes, the structural features of *COIs* were compared among families and species (*O. sativa*, *Ae. tauschii*, *T. urartu*, and *H. vulgare*) by aligning the predicted coding sequences (CDS) against the corresponding genomic sequences (Fig. 3a). The organization (number, length, and distribution) of exons and introns were variable (Fig. 3a). The sequence of *TaCOI6* was longest among these *TaCOIs*, while *TaCOI1-A* and *TaCOI4-A* were the shortest. The majority of *TaCOI* genes contained two or three copies, and the number of exons ranged from one to four (Fig. 3a). The copies of *TaCOI2, 5, 6,* and *7* had the same gene structures, whereas *TaCOI2* showed structural differences among copies. Furthermore, most *COIs* in the same sub-group shared the same structures; for example, *BdCOI7* and *HvCOI3* had the same structures as *TaCOI2-A* and *B* in S1, and *AetCOI3*, *HvCOI1,* and *TuCOI5* had the same structures as *TaCOI5-A*, *B,* and *D* in S12 (Fig. 3a). However, some *COIs* showed divergence in gene structure within the same sub-group. *TuCOI7* contained 7 exons, whereas other *COIs* in same sub-groups had three exons. To further investigate the function of *TaCOIs*, chromosomal locations and synteny analysis were performed. As shown in Fig. 4, 18 *TaCOI* members of 8 *TaCOI* families were located on 16 of the 21 chromosomes other than 1B, 1D, 2D, 7B, and 7D. The distribution of *TaCOI* family members in the wheat genome is depicted in Fig. 4. *TaCOI3-B* and *TaCOI4-B* as well as *TaCOI3-D* and *TaCOI4-D* were positioned on the same chromosomes, 3B and 3D, respectively. Each of the remaining chromosomes included a single *TaCOI* gene. The *TaCOI* genes showed a scattered chromosomal distribution. To analyze the relationships between the *COI* genes and gene duplications, *COI* gene synteny was analyzed (Fig. 4). The paralogous *TaCOIs* on different chromosomes were segmental duplication events and clustered together.

To characterize the architecture of COI proteins in wheat, motifs were analyzed by submitting the predicted amino acid sequences to the MEME website (Fig. 3b and Additional file 1: Figure S1). Most COIs in S1, S2, S12, and S13 possessed ten motifs, whereas COIs in S15 possessed motif3 and 6, and COIs in S16 possessed motif1, 3, 6, and 9. COIs in S7 and S8 possessed at least 6 motifs. Motif 1 was identified as an F-box domain, motif 2–6 were LRR domains (leucine-rich repeat), and motif 7–10 were also rich in leucine sequences. The motif logos and sequences are shown in Additional file 1: Figure S1.

### Tissue/organ-specific and stage-specific expression profiles of *TaCOIs*

**Fig. 5:**
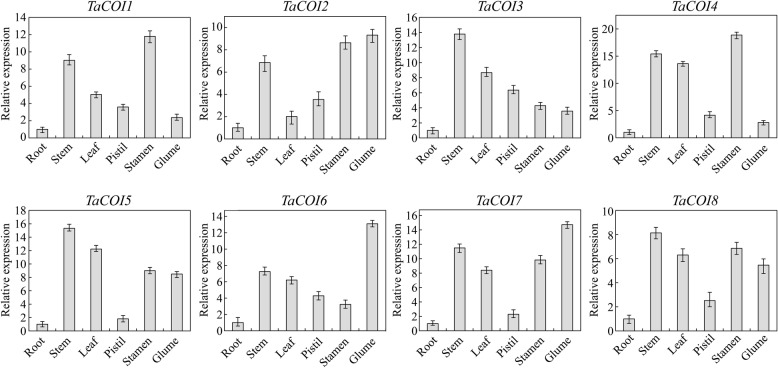
Real-time PCR analysis of *TaCOI* genes in six wheat tissues (root, stem, leaf, pistil, stamen, and glume) in the heading stage. Actin was used as a reference control gene. The 2^-∆∆Ct^ method was used to calculate the relative expression levels of the target genes. The error bars indicate the standard deviation obtained from three replicates

The *COI* gene family is primarily involved in plant growth [60], development [21, 35], and stress responses [61]. To study the biological functions of these genes in wheat, the expression profiles of the eight *TaCOI* genes were analyzed in six tissue types (i.e., root, stem, leaf, petal, pistil, stamen, and glume tissues) of PTGMS line BS366 at the heading stage by qRT-PCR. As shown in Fig. 5, eight *TaCOI* genes were expressed in different tissues, suggesting that the *TaCOI* genes were constitutively expressed in BS366. Compared with the expression of *TaCOIs* in root tissues, expression levels were higher in other aerial tissues (stem, leaf, petal, pistil, stamen, and glume). The expression levels of *TaCOI2, TaCOI6,* and *TaCOI7* in glume tissues were markedly higher than those in other tissues. *TaCOI1, TaCOI2,* and *TaCOI4* expression levels in the stamen were relatively highly than those in other tissues. Interestingly, expression levels were lower in important reproductive organs than in stem tissues.

**Fig. 6:**
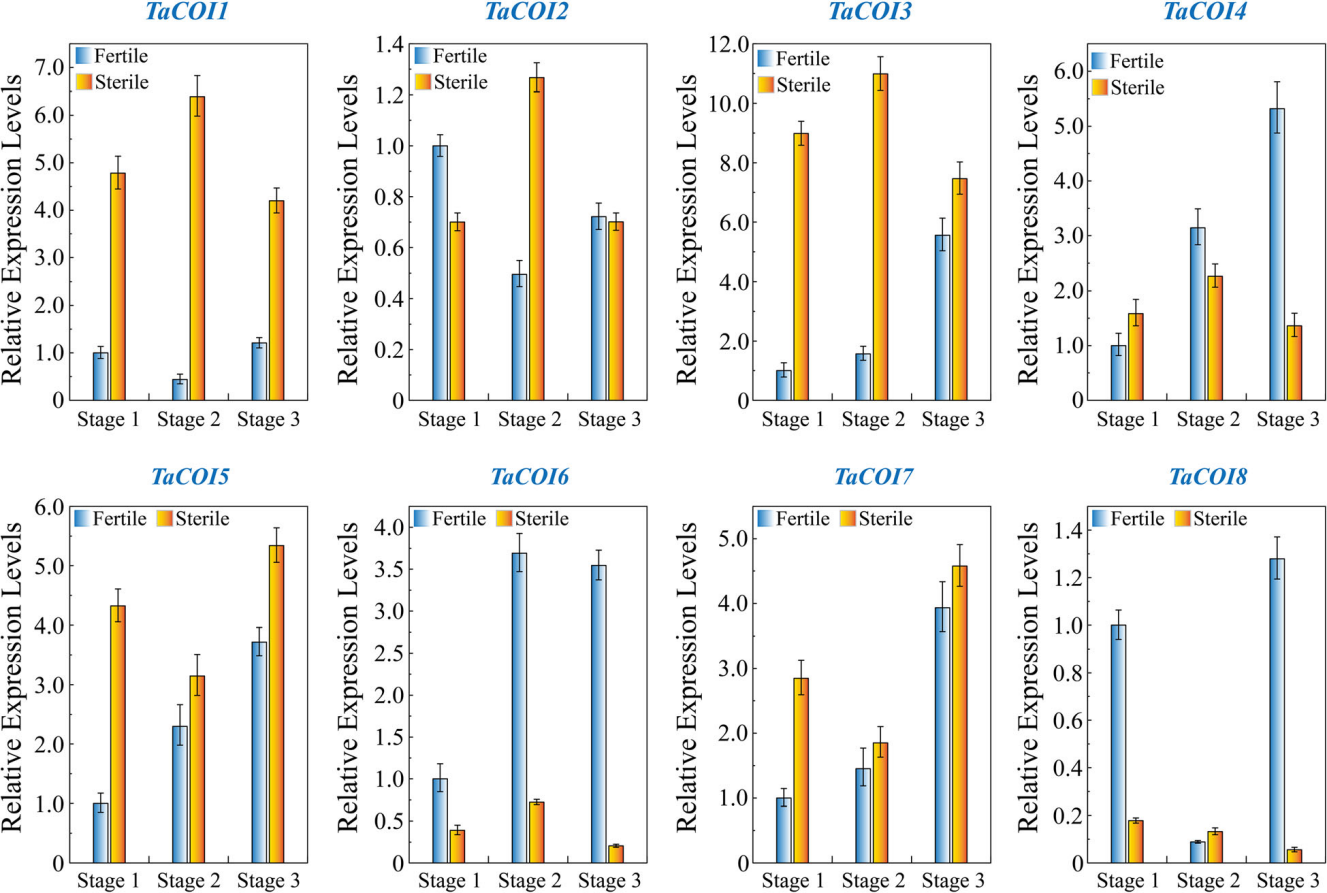
Expression profiling of *TaCOI* genes at three anther development stages in fertile (20 °C with 12-h day/12-h night for daily mean temperature during pollen development stages) and sterile conditions (10 °C with 12-h day/12-h night for daily mean temperature during pollen development stages). I, II and III are mean stage 1: secondary sporogenous cells had formed, stage 2: all cell layers were present and mitosis had ceased and stage 3: meiotic division stage, respectively. The error bars indicate the standard deviation of three replicates

To further investigate the role of *TaCOI* genes in pollen fertility, the *TaCOI* expression profiles at three stages of stamen development in different fertility environments (fertile conditions and sterile condition) were analyzed. As shown in Fig. 6, *TaCOI1, TaCOI3, TaCOI5,* and *TaCOI7* exhibited higher expression levels in all three stamen developmental stages in sterile condition than that in fertile condition, while the expression levels of *TaCOI6* and *TaCOI8* in the three stamen developmental stages were lower in sterile condition. *TaCOI4* showed higher expression in early stamen developmental stages (stage1) in sterile condition, while *TaCOI2* showed higher expression in later stamen developmental stages (stage2 and stage3) in sterile condition (stage1). These results suggested that these *TaCOI* genes may be involved in stamen development and participate in fertile transformation via some unknown pathway.

### Stress-induced expression profiles of *TaCOIs*

**Fig. 7:**
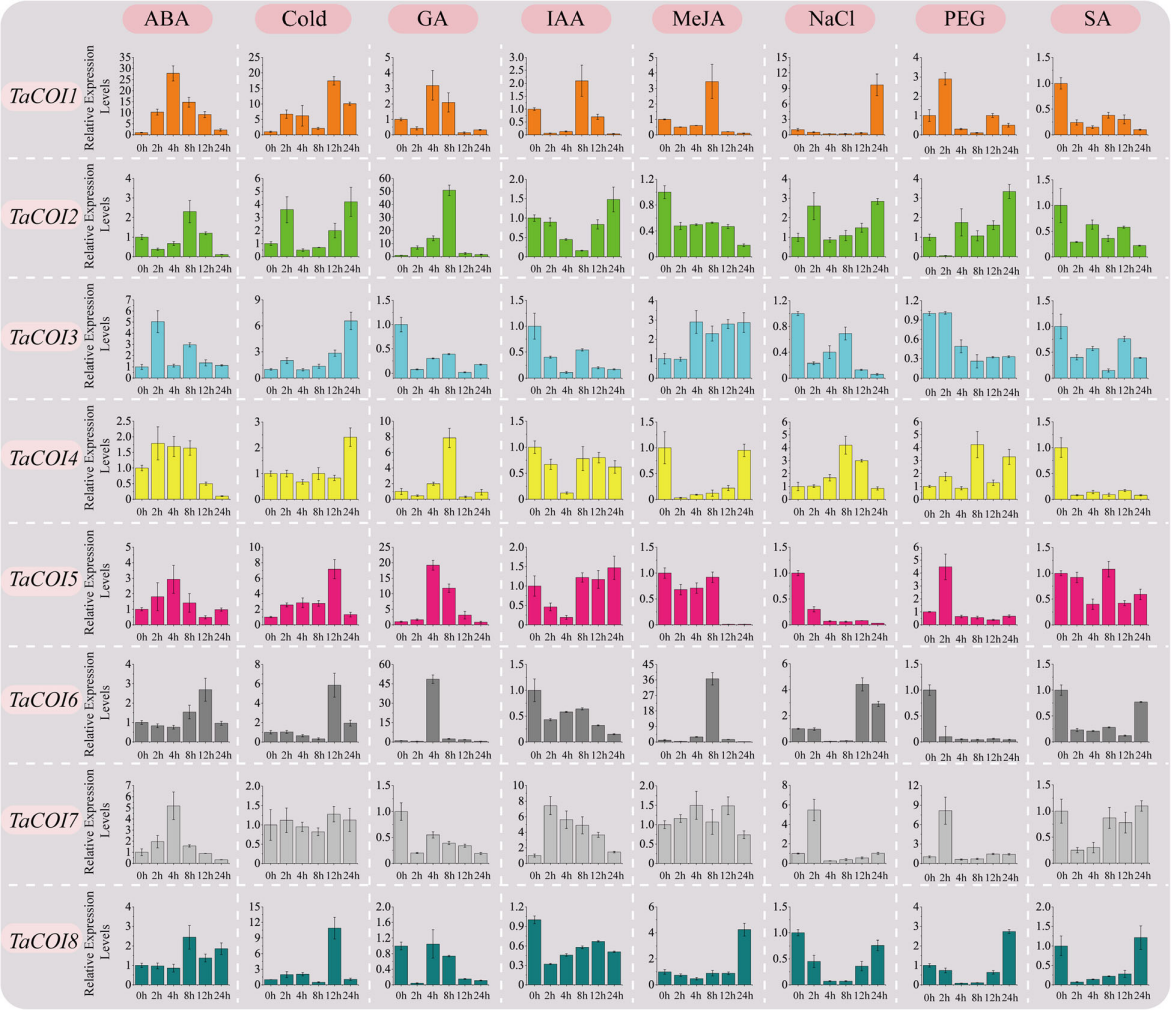
Expression profiling of *TaCOI* genes under five phytohormone (MeJA, ABA, GA, IAA, and SA), drought (PEG 6000), salt (NaCl), and cold (10 °C) treatments in BS366. Actin was used as a reference control gene. ABA (100 mM), Cold (10 °C), GA (100 mM), IAA (50 mM), MeJA (100 mM), NaCl (200 mM), PEG 6000 (− 0.5 MPa) and SA (2 mM), were preformed in this study. The 2^-∆∆Ct^ method was used to calculate the relative expression levels of the target genes. There are three replicates

In plant, Auxin, ABA, SA, MeJA, and GA are major hormones involved in plant development and metabolism [8, 62, 63]. To determine the mechanisms involved in the responses of the wheat *COI* gene family to plant hormones, cold (10 °C), salt, and drought, qRT-PCR was performed to determine the relative expression pattern of each *COI* gene using 14-day-old BS366 seedlings (Fig. 7). The results showed that all *TaCOI* genes responded to plant hormones and stress (Fig. 7). Under ABA treatment, the transcript profiles of *TaCOI* genes showed the up-regulation of *TaCOI1, 3, 4, 5, 7* and the down-regulation of *TaCOI2, 6, 8* at early treatment time points (0-4 h post-treatment). *TaCOI3* was induced 2 h post-treatment after ABA treatment and subsequently inhibited. In addition, we found that *TaCOI1, 5,* and *7* were peaked at 4 h post-treatment then inhibited (Fig. 7). Under GA treatment, *TaCOI1* and *5* were up-regulated, while *TaCOI7* and *8* were down-regulated during the treatment period; *TaCOI2* and *4* were up-regulated at 2–8 h post-treatment, but were rapidly down-regulated thereafter (Fig. 7). Under auxin (IAA) treatment, only *TaCOI7* was up-regulated during the treatment period. The remaining *TaCOIs* were down-regulated (Fig. 7). For MeJA treatment, the levels of *TaCOI2, 4, 5,* and *8* were down-regulated during the treatment period, while *TaCOI 6* was up-regulated at 8 h post-treatment. *TaCOI 3* was induced from 4 h post-treatment, following by a rapid up-regulation. Interestingly, all *TaCOI* genes were inhibited to different degrees under SA treatment. Notably, we found that the expressions of *TaCOI 4, 6, 7* were decreased after treated by both MeJA and SA, while the expressions of *TaCOI3* showed opposite pattern between MeJA and SA treaments, also between GA and ABA treaments, suggested that these hormones may play a antagonistic effect on expressions *TaCOI3.*

Under low temperature (cold) treatment, the expression levels of *TaCOI1, 2, 3,* and *5* were up-regulated during the whole treatment period (Fig. 7). For high salinity (NaCl) stress, the expression of *TaCOI3, 5,* and *8* showed down-regulation, whereas *TaCOI4* showed up-regulation during the whole treatment period. The expression of *TaCOI4* was increased at 4 h and peaked at 8 h. Under drought (PEG) stress, the expression levels of *TaCOI1*, *5,* and *7* initially increased, and subsequently decreased. Under drought (PEG) stress, the expression levels of *TaCOI3* and *6* were down-regulated at each time point after treatment. These results revealed that these *TaCOI* genes responded to various plant hormones and were involved in complex regulatory networks in the PTGMS wheat line BS366.

### The regulation of *COIs* in wheat anther/glume dehiscence

**Fig. 8:**
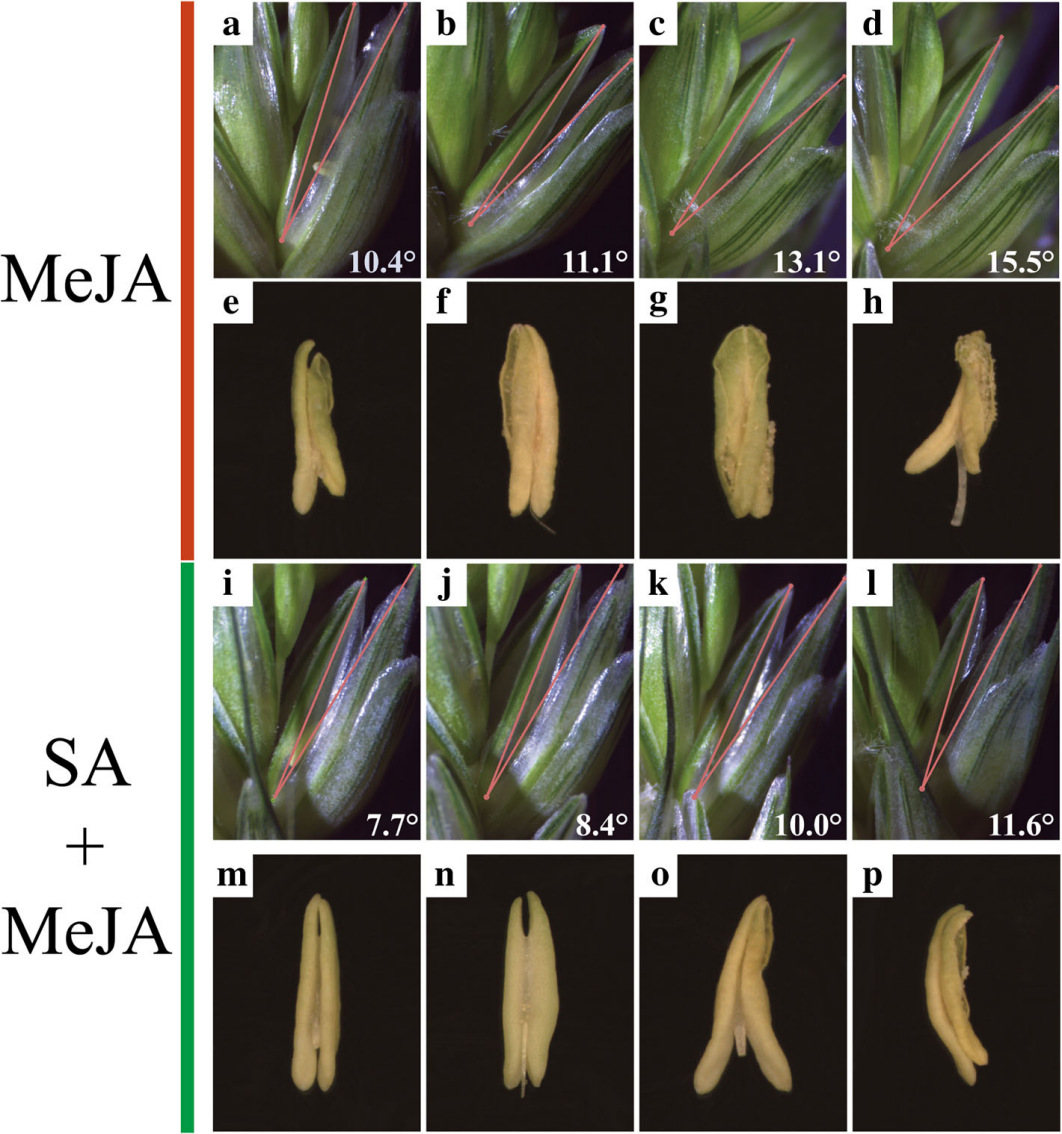
The dehiscence of glumes (**a–h**) and anthers (**i–p**) treated with MeJA and SA. **a–d** and **I–L** represent the glumes and anthers treated with MeJA (0 mM, 0.5 mM, 2 mM, and 4 mM from left to right), respectively. **e–h** and **m–p** represent the glumes and anthers treated with MeJA (0 mM, 0.5 mM, 2 mM, and 4 mM from left to right) and SA (10 mM). MeJA: spikelets were treated with MeJA (0 mM, 0.5 mM, 2 mM, and 4 mM from left to right). MeJA+SA: SA (10 mM) and MeJA interaction treatment

**Fig. 9:**
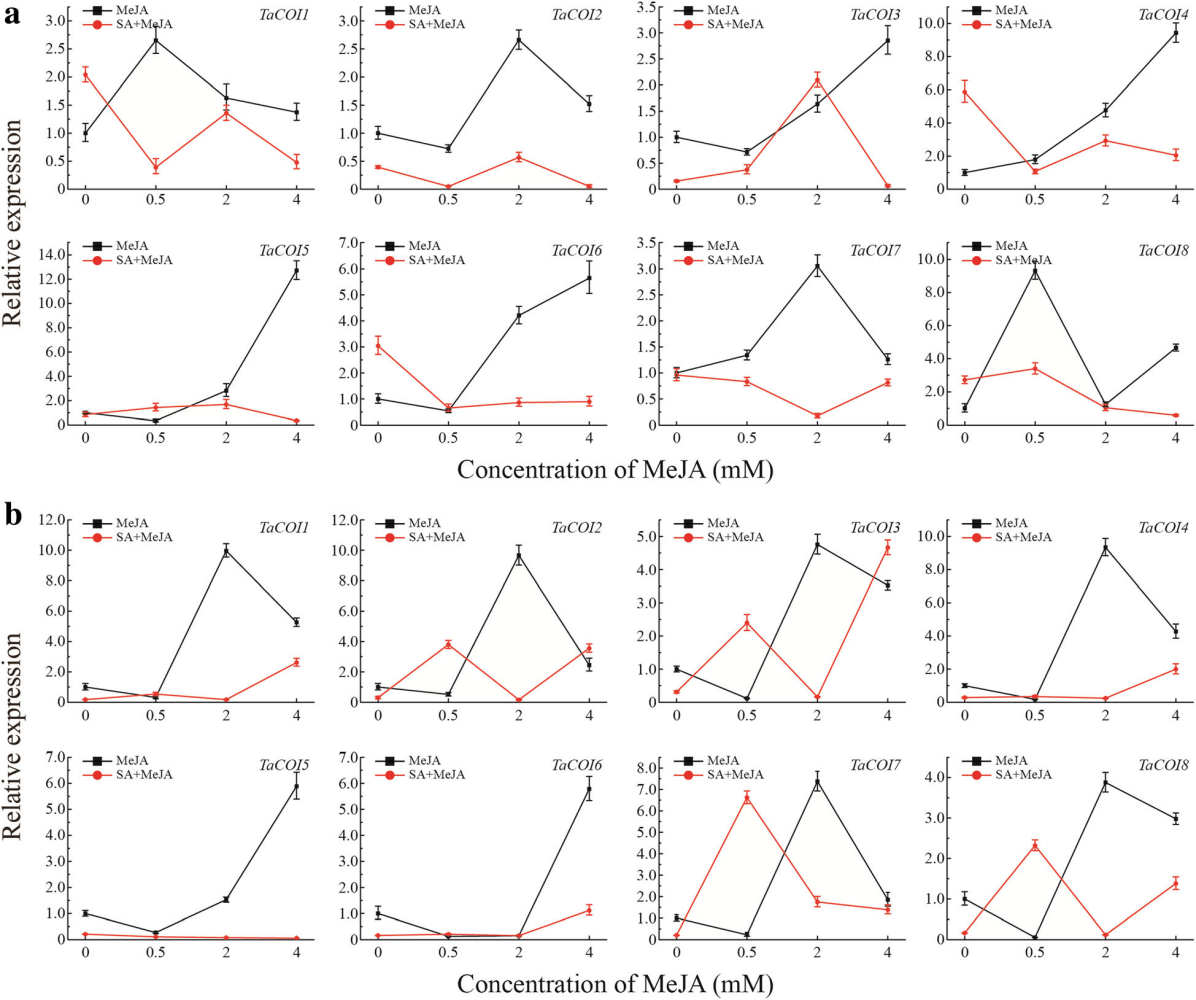
Expression profiling of *TaCOI* genes in glumes (**a**) and anthers (**b**) treated with MeJA (0 mM 0.5 mM, 2 mM, and 4 mM) and SA (10 mM). Values on the *x*-axis indicate the MeJA concentration. MeJA: spikelets were treated with MeJA (0 mM, 0.5 mM, 2 mM, and 4 mM). MeJA + SA: SA (10 mM) and MeJA interaction treatment. The error bars indicate the standard deviation of three replicates

To investigated the regulation of *COIs* in the JA signaling pathway during anther dehiscence, wheat spikelets were treated with various concentrations of MeJA and SA during anther development. Glume dehiscence was induced by increasing concentrations of MeJA (Fig. 8a–d), and the angles of glume dehiscence for 0, 0.5, 2, and 4 mM MeJA were 10.4°, 11.1°, 13.1°, and 15.5°, respectively. After treatment of MeJA-treated plants with SA, glumes dehisced at a smaller angle compared with those of MeJA-treated plants. The angles were 7.7°, 8.4°, 10.0°, and 11.6°, respectively (Fig. 8e–h). Anther dehiscence was also induced by increasing concentrations of MeJA (Fig. 8i–l). The anthers exhibited little dehiscence before MeJA treatment, but dehisced slightly in 0.5 mM MeJA, and reached full dehiscence in 2 mM MeJA. The expression levels of COI genes in glumes and anthers under MeJA and SA treatments were also investigated (Fig. 8i–p). The expression levels of *TaCOI3, 4, 5,* and *6* were up-regulated with increasing MeJA concentrations in the glume (Fig. 9a). Additionally, *TaCOI1* and *8* were induced and their expression levels peaked after treatment with 0.5 mM MeJA, while the expression levels of *TaCOI2* and *7* peaked after treatment with 2 mM MeJA and subsequently decreased. After treatment of MeJA-treated plants with SA (10 mM), the expression of all *TaCOI* genes was repressed in glumes (Fig. 9a). In the anther, the expression levels of *TaCOI5* and *6* were up-regulated as the MeJA concentration increased, and the levels of the remaining *TaCOIs* peaked after treatment with 2 mM MeJA and subsequently decreased (Fig. 9b). Furthermore, the expression levels of all *TaCOIs* in MeJA-treated plants were repressed by SA (10 mM), especially for plants treated with 2 mM MeJA (Fig. 9b).

## Discussion

JAs are oxylipin signaling molecules involved in the control of various aspects of plant developmental processes, such as plant fertility [5, 6, 8], anthocyanin accumulation [64], fruit ripening [65], root growth [66], and leaf senescence [24, 25]. In addition, they act as defense signals to mediate plant responses against abiotic and biotic stresses [18, 48, 60]. Upon exogenous or endogenous JA induction, the JA receptor CORONATINE INSENSITIVE 1 (COI1), which perceives bioactive molecules of JA [17, 21, 32, 67, 68], and triggers the degradation of the JASMONATE ZIM-domain (JAZ) proteins via the 26S-proteasome [18, 29]. The *COI* gene family has been characterized in many plants, such as *A. thaliana* [67]*, H. brasiliensis* [69], *G. hybridus* [70], and *O. sativa* [35], including analyses of gene and protein characteristics and their functions. However, in wheat, little is known about *COI* genes. In the present study, 18 candidate *COI* copies clustered into seven sub-groups (Fig. 1) and eight members of *COI* gene family were newly isolated in wheat (Additional file 2: Table S4).

The analyses of *TaCOI* genes provided insight into the important roles underlying the roles of MeJA in pollen fertility in wheat. In plants, the expression levels of *COI* family genes are regulated by various hormones, especially MeJA [70, 71]. Phytohormone crosstalk is a complex regulatory network involved in plant growth and development. Recent studies have reported that the modulation of JA responses by JAZ proteins probably plays a pivotal role in phytohormone signaling pathways [48, 81]. For example, JA-GA crosstalk could be important for defense against necrotrophic pathogens [82] and acts synergistically during stamen development, as a deficiency in either hormone results in infertility [83]. To clarify the functions of *TaCOI* genes, *cis*-acting regulatory elements and expression profiles under various stresses were analyzed. As shown in Fig. 2, five hormone-responsive (ABA, IAA, GA, MeJA, and SA) and two stress-responsive (high salinity, drought, and cold) regulatory elements were found in the *TaCOI* promoter regions, suggesting that *TaCOI* genes have different regulatory mechanisms in response to various stress and hormone treatments. However, there were some differences between *TaCOI* genes with respect to the number and type of *cis*-acting regulatory elements. For example, most *TaCOIs* contained two or more CGTCA/TGACG-motifs, but not the promoters of *TaCOI1-A* and *TaCOI8-A* (Fig. 2, Table 3)*.* These results indicated that different *TaCOI* members may respond to different stress and hormone stimulation. Therefore, we investigated the expression profiles of *TaCOI* genes in wheat seedlings under different stress conditions were evaluated. In plants, the expression levels of *COI* family genes were regulated by different hormones. In *Arabidopsis, COI1* functions in the JA signaling pathway and is required for pollen development [9, 21] and defense against pests [80]. In this study, inducible expression analyses revealed that *TaCOI* expression could be induced by at least one hormone (Fig. 7). For example, both *TaCOI2* and *TaCOI6,* with similar protein structures, were down-regulated by treatment with IAA, MeJA, SA and PEG (Fig. 7). *TaCOI1* was up-regulated in response to GA and IAA. These *TaCOI* genes were also induced by some abiotic stresses (Fig. 7). *TaCOI1* was up-regulated under salt and drought stresses, but *TaCOI3*, *TaCOI6,* and *TaCOI8* were down-regulated. Based on the composition of *cis*-acting regulatory elements in promoter regions, *TaCOI7-A*, *B,* and *D* had two or more CGTCA/TGACG-motifs in their promoter regions (Fig. 2, Table 3), but the response to MeJA was not obvious for *TaCOI7* (Fig. 7). Similar results were obtained for *TaCOI 1* (Fig. 2 and Fig. 7). There was no obvious response to SA for *TaCOI1*, but there was a single TGA-element in its promoter region (Fig. 2 and Fig. 7). Therefore, these *TaCOI*s might be directly or indirectly involved in phytohormone crosstalk [48].

Recently, many studies have shown that the LRR domain (LXXLXXLXX LXLXXNXLVGXIP) may function in determining resistance specificity, suggesting that LRR domain-contained proteins are involved in stress resistance in plants [76, 77]. Function studies showed that LRR domain-containing proteins, such as CLAVATA1 and ERECTA, which are membrane receptor kinases, are involved in signal reception and transmission in plant development [78, 79]. FLOR1 is a flower-specific LRR protein involved in the development of floral organs in *A. thaliana* [6], suggesting that *TaCOIs* play a role in plant defense and floral organ development. In *Arabidopsis*, the sequence of the COIl protein contains a degenerate F-box motif [17, 21, 67, 72] and 16 imperfect leucine-rich repeats (LRRs) [73]. Some studies have shown that LRRs and F-box motifs are involved in protein–protein interactions [74, 75]. In our study, the structural features of TaCOI proteins in different families and species were also investigated. It was found that the most of COI proteins were enriched for the LRR domain and F-box domain, especially LRR domains (with 2–9 LRR domains per TaCOI) (Fig. 3 and Additional file 1: Figure S2). For example, TaCOI6 and TaCOI2 shared similar structures and contained the same ten motifs, and 9 out of 10 motifs were LRR domains or leucine-rich sequences, which was consistent with the conclusion obtained by the previous scholar [76, 77], suggesting that these *TaCOI* genes may play a role in plant defense.via these enriched domains.

The tissue expression profiles of *TaCOI* genes were also investigated to further examine *TaCOI* gene functions (Fig. 5). In this study, the expression levels of *TaCOI2*, *TaCOI6,* and *TaCOI7* in the glume and *TaCOI1* and *TaCOI4* in the stamen were higher than those in other tissues (Fig. 5). Additionally, all *TaCOI* genes showed lower expression levels in root tissues than in other tissues (Fig. 5). These results suggested that *TaCOI* genes are involved in stamen and glume development. Plant hormones often play a key role in plant growth and development synergistic ally or antagonistically, through a series of complex networks [87]. Studies reported that GA and ABA showed antagonistic roles in plant growth and development [87]. Our results also showed that some *TaCOI3* genes exhibited opposite expression pattern under GA and ABA treaments (Fig. 7). Recently, studies have indicated that JA and SA signaling act in a mutually antagonistic manner [84]. MeJA plays a positive regulatory role in glume opening, while SA inhibits glume opening in wheat [85]. It has been reported that JAs are involved in the regulation of anther dehiscence, filament elongation, and pollen fertility in plants [48, 83],. However, the mechanisms underlying JA–SA crosstalk and particularly the mechanisms by which JA signaling antagonizes SA signaling are still largely unknown. MeJA and SA may have antagonistic effect on expressions *TaCOI* genes in PTGMS wheat line (Fig. 7) Thus, the regulation of *TaCOIs* in JA–SA signaling crosstalk in anther dehiscence and glume opening was examined by evaluating the effects of various concentrations of MeJA and SA on wheat spikelets during anther development (Fig. 8). Glume dehiscence increased as the concentration of MeJA increased; the angles of glume dehiscence for 0, 0.5, 2, and 4 mM MeJA were 10.4°, 11.1°, 13.1°, and 15.5°, respectively (Fig. 8a–h). Changes were also detected in anthers (Fig. 8i-p). In addition, SA showed an inhibitory effect on anther and glume dehiscence. The glumes dehisced at a smaller angle compared with those of MeJA-treated plants (7.7°, 8.4°, 10.0°, and 11.6° respectively), consistent with the results of previous reports (Fig. 8a–h) [85, 86]. The expression of *TaCOI* genes in the glume and anther under various concentrations of MeJA and SA were also investigated (Fig. 9). *COI* is associated with jasmonate-regulated defense and fertility in *Arabidopsis* [67]. In the present study, some *TaCOI* genes were inhibited by SA and induced by MeJA (Fig. 9). For example, the expression levels of *TaCOI 3, 4, 5,* and *6* were up-regulated with increasing concentrations of MeJA in the glume and the expression levels of *TaCOI 5* and *6* were up-regulated with increasing concentrations of MeJA in the anther (Fig. 9). In addition, all *TaCOI* genes were inhibited by SA in the glume and anther (Fig. 9). It was suggested that *TaCOIs* are involved in JA–SA signaling crosstalk, associating with anther and glume dehiscence. In addition, the expression results for *TaCOI* genes during anther development in different fertility environments showed that *TaCOI1, TaCOI3, TaCOI5,* and *TaCOI7* exhibit higher expression, while the expression levels of *TaCOI6* and *TaCOI8* were lower at all three anther development stages in the sterile environment (Fig. 6), suggesting that *TaCOI* genes have different roles in fertility.

Taken together, these results demonstrated that *TaCOIs* may have multiple functions, in plants, including roles in abiotic stress resistance and pollen fertility. Structural analyses and the expression patterns of the *TaCOI* genes would provide a more comprehensive understanding of gene functions. Further studies are needed to confirm the conclusions presented in this study.

## Conclusions

In this study, a comprehensive overview of the *COI* family in wheat, including gene structures, phylogenetic relationships, and expression profiles, was provided. The roles of *TaCOI* gene expression differences in anther development and in the responses to various abiotic stresses provide insight into the roles of MeJA signaling in the anther and glume dehiscence of wheat.

## Additional files

Additional file 1: **Figure S1.** Consensus sequence and logos of motifs from wheat COI proteins. (TIF 2339 kb)

Additional file 2: **Table S1.** Summary of *COI* or *COI*-like genes in different plants. **Table S2.** Specific primers for *TaCOI* genes for qRT-PCR. **Table S3.** Promoter sequences of *TaCOI* genes. **Table S4.** Protein sequences, CDS sequences, and genomic sequences of *TaCOI* and *COI* or 12 *COI*-like loci in various plants. (DOCX 241 kb)

## Abbreviations

ABA: Abscisic acid
COI: Coronatine insensitive
GA: ibberellin
IAA: Indole-3-acetic acid
JA-Ile: Jasmonoyl isoleucine
JAZ: Jasmonate-ZIM domain
MeJA: Methyl jasmonate
ORF: Open reading frame
PTGMS: Photoperiod-Temperature sensitive Genic Male Sterile
qRT-PCR: Quantitative real-time PCR
SA: Salicylic acid
SCF^COI1^: Skp1-Cullin-F-box protein
