# Supplementary figures and images for: Genome-wide identification and analysis of the COI gene family in wheat (Triticum aestivum L.)
Source: BMC Genomics. 2018 Oct 17;19:754. doi: 10.1186/s12864-018-5116-9 (PMC6192174; doi:10.1186/s12864-018-5116-9)

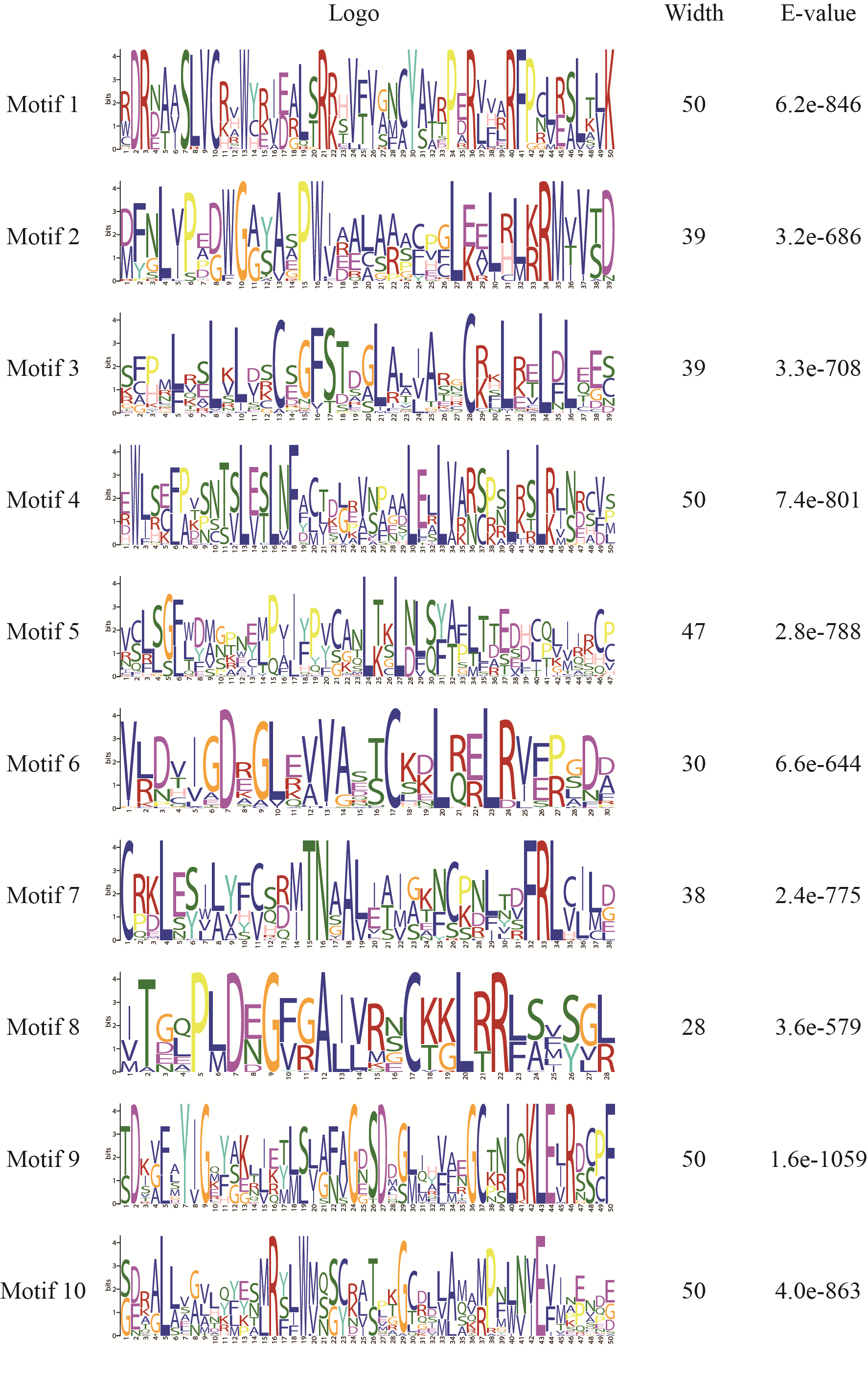

Supplement: Supplementary file 1 — Figure S1. Consensus sequence and logos of motifs from wheat COI proteins. (TIF 2339 kb) [file 12864_2018_5116_MOESM1_ESM.tif]
