# Supplementary material for: Genome-wide identification and analysis of the COI gene family in wheat (Triticum aestivum L.)
Source: BMC Genomics. 2018 Oct 17;19:754. doi: 10.1186/s12864-018-5116-9 (PMC6192174; doi:10.1186/s12864-018-5116-9)
Supplement: Supplementary file 2 — Table S1. Summary of COI or COI-like genes in different plants. Table S2. Specific primers for TaCOI genes for qRT-PCR. Table S3. Promoter sequences of TaCOI genes. Table S4. Protein sequences, CDS sequences, and genomic sequences of TaCOI and COI or 12 COI-like loci in various plants. (DOCX 241 kb) [file 12864_2018_5116_MOESM2_ESM.docx]

Table S1 The information of *COI* or *COI*-like genes in different plants

| Species | Locus name/Accession number | Gene name | A.A length |
| --- | --- | --- | --- |
| *Aegliops tauschii* | EMT18237 | *AetCOI1* | 562 |
|  | EMT11181 | *AetCOI2* | 694 |
|  | EMT22616 | *AetCOI3* | 480 |
|  | EMT31633 | *AetCOI4* | 506 |
|  | EMT03750 | *AetCOI5* | 487 |
| *Arabidopsis thaliana* | AT3G62980.1 | *AtCOI1* | 594 |
|  | AT1G12820.1 | *AtCOI2* | 577 |
|  | AT5G49980.1 | *AtCOI3* | 619 |
|  | AT4G24390.1 | *AtCOI4* | 623 |
| *Brachypodium distachyon* | BRADI2G23730.2 | *BdCOI1* | 596 |
|  | BRADI3G58320.1 | *BdCOI2* | 617 |
|  | BRADI4G05157.1 | *BdCOI3* | 666 |
|  | BRADI4G06170.1 | *BdCOI4* | 601 |
|  | BRADI4G08400.1 | *BdCOI5* | 533 |
|  | BRADI4G11900.1 | *BdCOI6* | 623 |
|  | BRADI5G08680.1 | *BdCOI7* | 575 |
| *Hevea brasiliensis* | MLOC_18524.1 | *HvCOI1* | 586 |
|  | MLOC_52024.5 | *HvCOI2* | 568 |
|  | MLOC_56088.1 | *HvCOI3* | 574 |
|  | MLOC_67830.1 | *HvCOI4* | 417 |
|  | MLOC_72489.1 | *HvCOI5* | 502 |
|  | MLOC_73542.1 | *HvCOI6* | 560 |
|  | MLOC_80547.1 | *HvCOI7* | 594 |
|  | MLOC_9864.2 | *HvCOI8* | 464 |
| *Oryza sativa* | LOC_Os01g63420.1 | *OsCOI1* | 630 |
|  | LOC_Os05g37690.1 | *OsCOI2* | 597 |
|  | LOC_Os03g15880.1 | *OsCOI3* | 589 |
|  | LOC_Os02g52230.1 | *OsCOI4* | 667 |
|  | LOC_Os11g31620.1 | *OsCOI5* | 568 |
|  | LOC_Os03g08850.1 | *OsCOI6* | 603 |
|  | LOC_Os05g05800.1 | *OsCOI7* | 594 |
| *Physcomitrella patens* | PP1S277_20V6.1 | *PpCOI1* | 592 |
|  | PP1S211_131V6.1 | *PpCOI2* | 593 |
|  | PP1S24_60V6.1 | *PpCOI3* | 584 |
|  | PP1S140_66V6.1 | *PpCOI4* | 615 |
|  | PP1S187_72V6.1 | *PpCOI5* | 667 |
|  | PP1S49_196V6.1 | *PpCOI6* | 693 |
|  | PP1S12_347V6.1 | *PpCOI7* | 693 |
|  | PP1S3_119V6.1 | *PpCOI8* | 626 |
|  | PP1S44_198V6.1 | *PpCOI9* | 570 |
|  | PP1S196_87V6.1 | *PpCOI10* | 567 |

Table S1 The information of *COI* or *COI*-like genes in different plants (*Continued*)

| Species | Locus name/Accession number | Gene name | A.A length |
| --- | --- | --- | --- |
| *Populus trichocarpa* | POPTR_0004s03400.1 | *PtCOI1* | 579 |
|  | POPTR_0002s10310.1 | *PtCOI2* | 635 |
|  | POPTR_0001s33030.1 | *PtCOI3* | 571 |
|  | POPTR_0020s00280.1 | *PtCOI4* | 584 |
| *Sorghum bicolor* | Sb09g022040.1 | *SbCOI1* | 599 |
|  | Sb03g040150.1 | *SbCOI2* | 596 |
|  | Sb01g040110.1 | *SbCOI3* | 591 |
|  | Sb04g033850.1 | *SbCOI4* | 662 |
|  | Sb06g014420.1 | *SbCOI5* | 574 |
|  | Sb05g018860.1 | *SbCOI6* | 578 |
|  | Sb01g044720.1 | *SbCOI7* | 602 |
|  | Sb09g003870.1 | *SbCOI8* | 591 |
| *Selaginella moellendorffii* | EFJ04886 | *SmCOI1* | 727 |
|  | EFJ07502 | *SmCOI2* | 574 |
|  | EFJ09817 | *SmCOI3* | 600 |
|  | EFJ14966 | *SmCOI4* | 616 |
|  | EFJ15060 | *SmCOI5* | 657 |
|  | EFJ20341 | *SmCOI6* | 407 |
|  | EFJ24298 | *SmCOI7* | 630 |
|  | EFJ29076 | *SmCOI8* | 600 |
|  | EFJ30612 | *SmCOI9* | 572 |
|  | EFJ31715 | *SmCOI10* | 637 |
|  | EFJ32115 | *SmCOI11* | 568 |
| *Triticum urartu* | TRIUR3_17029-P1 | *TuCOI1* | 789 |
|  | TRIUR3_18749-P1 | *TuCOI2* | 462 |
|  | TRIUR3_21512-P1 | *TuCOI3* | 536 |
|  | TRIUR3_22304-P1 | *TuCOI4* | 409 |
|  | TRIUR3_23066-P1 | *TuCOI5* | 575 |
|  | TRIUR3_23956-P1 | *TuCOI6* | 464 |
|  | TRIUR3_24621-P1 | *TuCOI7* | 790 |
|  | TRIUR3_31653-P1 | *TuCOI8* | 477 |
|  | TRIUR3_32567-P1 | *TuCOI9* | 541 |
|  | TRIUR3_34918-P1 | *TuCOI10* | 451 |
|  | TRIUR3_35106-P1 | *TuCOI11* | 504 |
| *Zea mays* | ZM01G10730 | *ZmaCOI1* | 591 |
|  | ZM03G26700 | *ZmaCOI2* | 598 |
|  | ZM06G24870 | *ZmaCOI3* | 599 |
|  | ZM08G14420 | *ZmaCOI4* | 503 |

Table S2 Specific primers of *TaCOI* genes for qRT-PCR

| Primer name | Forward sequence | Reverse sequence |
| --- | --- | --- |
| *TaCOI1*-RT | CAGCTATTGTGAGACGAGGACG | AGAAGTTGGCGGTGTTTAGCA |
| *TaCOI2*-RT | ATGAAGCGGATGGTGGTGTC | AGGAATCAGGGAAGCAGGAGA |
| *TaCOI3*-RT | TTACTGACGAAGGTGGTGAATGG | CAATCAGGTCTGAAAGGTCACACTC |
| *TaCOI4*-RT | TAGAGGGGAATCAAAGGGGTG | GCGAGGAACAGTGAGATGAAAAC |
| *TaCOI5*-RT | GCCATCTCGCTCGTCTGC | CTCGGGGATGAGGTTGAACA |
| *TaCOI6*-RT | GCTTGGATGGAGCAGTGAGTG | GAATAGCACTGTAGAGACTGGGGAG |
| *TaCOI7*-RT | GCAGCATACCTCCCAGCAATC | CATGTTTCAGCCACAGCCCTAA |
| *TaCOI8*-RT | CCTTCCAGCAATCTACCCAGTG | GTGGCATCCAGCGGAAATACT |
| *TaActin*-RT | TACTCCCTCACAACAACCG | AGAACCTCCACTGAGAACAA |

Table S3 The promoter sequences of *TaCOI* genes

>*TaCOI1-A*

AAGCCATTCATTGATGAGATCAACGGCTAAGATCTATTTTATACTCTTACCTCTCATGTGAAAAGAGGGGGTAAGAGGGATCATGTTAAAACTGCTCATGGAAAAATGACACAGGACTTATTCCTAGAAAGCAATACCCACGGCGTACGCCTGCTCATATATTTCCAATTCTCCTCCATCAACAACTCATGCACTGAACGACTTGAGTTAACAATGCAGTAGAGTTTTTCTTTGATGGTATCCTTCCTTGTTTATTTGATCATGTAAAACTGCTTCATTATATATGTCTTAAGCTATTCTCTAAAAGGTTTTCCTTTTCGCTTCTCAGCTGGTACATGGACCAAGTATAGATAAGGACTCATGAAGGGTGGCGCGATGCAATGCAACTACAAATTGCGGGAAAATTCCATGCTTGAATTCTATCCAGCGGCAGTGGTAGAAATAAAGGTTTCGGTGTTGAATTCTGTTTAACACAAGTCTTGTTGGAAGTCGTGTGTCCGTGGGAATATGAGGCCATGGCTGCAACTTTCTGGAACTCAAAGCCTGTGCAGCCAAGCAGCATCTTGACCACACAAATTGAACAAAATACAAACTCAAGTGGGTTATTGGCGCTTTTTGTACGTCTACCAGCCCATGTATGTGTTGGTTGCCGTGTAACATCTGACACGCCAAAATTTTCTTCTTCAGTGTCGCCATCTATTCCTCTGTTTTCTCCTGTGGAAAAAATGACAGATTAATTTTCTCCTGTGAATTTTTTCCTAGACAGTGATGCAAACTCTGTAGCTCACGGGCTAGCAAGTTCAGTTTTTGTAAGAAGTGTGACGATAGTTGGACTAATGAACCTCCAGGGTTTTTACTTAGCCAACTAGTGAACAATGTAATTTGTGTGTTTGGCTGCATCTTGTGAAATTCAAGGCATCTAGAAGCAAGCAGCATCTTGGCCACACCCCACACAAACTGAAGAAGAACTGCAACCTCCTGTACGTTTGCCGGATTGCCATGGCGGCGCTTCCTGTACATCTGCCGGATTGCTAATGTAAACAAATAAACTATTCTCCCTTTTTTTAGCAAGAGAAAGGTGCGTGTTTCATTTTAGCTCAGGTATAAAAACAGAAATTAAATAGCCAGCAAAGCAAAGTTGGCATCTTGATGAGCTACAAATACAAGGCAAATGCTGTTCCTACTAGCTGAACTGATGCAACGAGCTATCAACATATATACTCTTGATAGGTAAAAAGAGATGTGGCCCCCTAAAAAAGTTAAGAAAATGTTTTTGAGTGTTGGATGTCCAGAGCGAAGACATGATATGATGGATATGCTATATGCTTTTAGTAGTCACTCAAGTTTCAGTTGTACCTCAACTTCATGTCATTTTGGAATCTATACATCCACAATTATGCTACTACCTCTGTACACTAATGCAAGACGTACAGAGGGAGTAGTAACTAGCACTATTCTTTTGAGAAGCTATCAAAGTCATCCTCCAATGGAGTCAATATCAACAAAACAAGCATCAAAACTTGACTTATTACTACATTCATTCATACCAAAATTGGCACGCGAACATAGCAGGCAGATATATGTCTTCAAAATCACTTACCCAATGAAAAAAGGGTTCACGACATTTGCTTAAATAATACAAATAACACCAAAAGGTCATATAGTGCCTCCACAGCAGAAACAGGGAATCTTGTGGCCTACTTATGCCGGTTTCGCTATAGAGGGTAGAAATTAAGGAACCAGAATTACGGTTGACAATAATACACAAGTTGTGCCTACTACTAAAGTTTCATTAATTAGTTGCCATCTGGAATTCATCATTATAGACTGTCTAAAGGAAAATCATTGCCGTAAAGGTAGAGGGCAACAAAAAGTTCAGAGAGGTGAATCTGGGTCGGATGATCCACACTGAACAACTTAATGGCTTCAGCAGCAAATTCATACTGAAAATTTGGAGACCACTTCAT

>*TaCOI2-A*

GTACTCTATCTCTTCTTCTTGTAACCTGTGCTGTCAAATGGGTACAAGCCTGGTGAAACGTCACATTTTGGGATGAGGACGAAACACCAATCCTAACGAACTCCCTCTTAAGTTATTCGGTTAAAACTACTTCGAACGGTTATGTAAGTTACATCACTGGGAACACCGACGTTGCATAGTACAAACGTCCAAATGACATTAGCTTACCTTCTATGCTTCATCCCAATGCCGTGATAAGAGTTGAGAACCGACACTCACCTACCTGCGGTGTTCTGATCTTGTAACAGAGGCGACGTACTAACATAATATTCGATCGGGTACTCGGTTCGTAAACACATAACATTTTCGTGACCTAGATGCTACATTATGATACTGTACACTACATAAGAACCATAAAGTAGGAGCTGACACGTACGGTTGATAACCACGTCCCTGACTGTGCCATTCGCATCTCTAAGTCCGAGGATATTCCCTGGCCCAGCACTGTAGTTGCTAAGAACAAGGTTTAAAGTAAGCCAAGTTCACTAGGAATGGTCGGTTATGTAAAATGCCGTTGCTCTTATATAACAAAACTCCATATAGTTTTGGTTATGAGAAAATATTAAGAGTAAAAACTGCTAAGGGAATACGGTGCGTATTATTCTTGAGGTTTTTCTTTAATTGGTTACTCATTTAAACCTTGTAAACGAGCGTTTATTTGTTTGAACGAGATGGAGGTACAAACAAATAGTTCACTGAATATAGTAGCGGTAGTTATTATGGGCAAATTTTTAAAGTAAACTTAGTTCACTAGGAACCACCACCGGTTACGTAAGGAACCGTTGGTCTTATATGAGAAAACTCCACCGGGCAAGCATAAAGCAGCTGGTCAGAAAGCATGGAGTGGAAAACTAGTAAAGAAAAAATTAAAGAGTGTAGTGCATTTCTGGTTGGGCCACTCCCGACCAAACCTACGCACGTAGAGGTCGGAGGAGGGTTCAGGATGAGGATGATGACTTCTCTCTCTCTCTCTCTCTCTCTCCCTCTCGTAAGGGGAGGAGGTTGGAGGGGGGGTCGACGGAGGGGAGGGTTTCGAGGGGTGAAGAGGGCGAGGCCGCAGCGTAAGCGGGGCGGGCGGGGCGGCTAAGGGAGCCCAGAATAGGAGCGAACGCCGGACGGCTGTCGCCCCGAGGGGAGGCGAGCCTCAGAGGGGCGCGGGGCGGCTTAAAGAGCCCAGTAAGAGAGGCCGCCGAGGCCCACGCCCGAAAGCCAGGCCCCGCGGCGCCGGGCGCTAGTAACGACCTCCTCTCGAAAAAAGCGCCTCCCCAGACCACGCCTCAGACGTCATGGCCGCCGCAGGTATCCAATCTAGACCTCCAAAGCCGCGAGACCTAGACTCGACAACCATCCGACGTAAAAAAAAAAGCAAAGGAAGAGGGGCAGCGATAAACAAAGGAAAAAGAATCGTTCAAGGGCCTCCGCCGTTCTTGAGTCGCCAGCGACTAGAAAGAAACGTCCAAAACCCACCTCCTTAGCCCTGACCGCCAGCGTTAGGAGGATCGGTAAAACCACGACAGGCAAGACCCAATGATCCCCCAAACGCACAAGATTTATCCCATACCTGCCGTCTACGTCATTGATGATCATAGAGAACTTCTTTGAAGTCGACGTCTAGACTTTGGGTAAAAACACGGGGAACGACATCAACATCGGGACGGTCTACTCCACTTGGTCCGGCGAAAGATCACGGGGGAACGGTAACGAACAACGGTCACAACCAACGGTCAAAATCGAGTTGTTTAGACCGACCTCGGACTAGACGGGTTCGGTCGTAATCAACACACGAGAGACACAGACCAACTCGTTCCAACGTCGAAGGTAAACAACTAAAGCAGCCGAAACAAATCATCGAAACTATTGGACCTCTGACGTTCGTTTGTCTAGGGCAGGACGTCGAGATCATTATTCCACCACGACCCCCTTCCGTGG

>*TaCOI2-B*

CTCCGAGGCAGCTCGTGCTCGATCTCTTCTTCTTGACACCTGTGCTATTAAATGGGTCTAAGCCCGGTGGAACGCCACATTTTGGGGCGAGGACGAAACATCAACCCTAACGAACCCCCCTCTTAGGTTATTCGGTTAAAACTACTTCGAACGGTTATGTAAGTTACATCGCTGGGAACACCGACATCGCATAGTACAAACGTTCAAATTCTTTATCCCAATGCTGTGATATAAGTTGAGAACAGACACTCACCTGCCTGCGGTGCTCTGATCTTGTAATAGAGGTGACGTACTAACATAATATTCGATCGGGTACTCGGTTCGTAAACACATAACATTTCGTGACCTAGATACTACATTATGATACCGTACACTACATAAGAACCATAAAGTAGGAGCTGGCATGTACGGTCAATAACTACGTACCTGACTGTGTAATTTGCGTCTCTAAGTCCGAGGATATTCCCTGGCCGAGCACTGTAGTTGCTAAGAACAAGGTTCAAAGTAAGCCAAGTTCACTAGGAATAGCCGGTTATGTAGAACACCGTTGCTCTTATATAACAAAGCTCCATATAGTTTTAATTCAAAAGCTAAGTATAAACAACCTGGTTATTAGAAACAATTAAGAATAAAAACTGCTAAGAGAATACGGTGTGTATATTCTTGATTTTTATATATTTTTATTGGTTACTCATTTAAACCTTGTAAACGAGCGTTTATTTGTTTGAACGAGATGGAGGTCCAAATAAATAGTTCATAAATATTAGTAGTGGTAGTTAATATGGGCAAATTTTAAAGTAGACTTAGTTTACTAGGAACCACCGGTTACGTAAGGAACCGTTCGTCTTATATGAGAAAACTCCACCGGGCAAGCATAAAGCAGCTGGTCAGAAAGCATGAAGTGGAAAACTAGTAAAAAAAATTAAAGAGTGCATTTCTGGTTGGGCCACTCCAAAACCAAACCTACGCACGTAGAGGTCGGAGGAGGGTTCAGGATGAGGATGATGACTTGTCTCTCTCCCTGTCTCGTAAGGGGAGGAGGTTGGAGGGGGGGTCGACGGAGGGGAGGGTTTCGAGGGGTGAAGAGAGCGAGGCCGCAGCGTAAGCGGGGCCGGCGGGGCGGCTAAGGGAGCCCAGAATAGGAGCGAGCGCCGGACGGCAGTCGCCCCGAGGGGAGGCGAGCCTCAGAGGGGGCGCGGGGCGGCTTAAAGAACCCAGTAAGAGAGGCCGCCGAGGCCCACGCCCGAAAGCCAGGCCCCGCGGCGCCGGGTGCTAGTAACGACCTCCTCTCGAAAAAGGCGCCTACCCAAACCAAGCCTCAGACCTCATGGACGCCGCAGGTATCCAATCTAGACCTCCAAAGCCGCGAGACCTAGACTCGACAACCATCCGACGTAAAAATAAAAGCAAAGGAAGAGGGGCAGCGATAAACAAAGGAAAAAGAATCGTTCAAGGGCCTCTGCCGTTCTTGAGTCGCCAGCGACTAGAAAGAAACGTCCAAAGCCCACCTCCTCAGCCCTGACCGCCAGCGTTAGGAGGGTTGGTAAAACCACGACAGGCAAGACCCAATGATCCCCCAAACGCACAAGATTTATCCCGTACCTACCGTCTACGTCATTGATGATCATAGAGAACTTCTTTGAAGTCGACGTCTAGACTTTGGGTAAACACGGGGAACGAGATCAACGTCGGGACGGTCTACTCCACTTGGTCCGGCGAAAGGTCACAGGGGAACGGTAACGAACAACGGTCACAACCAACGGTCAAAACCGAGTTGTTTAGACCCACCTCGGACTAGACAGGTTCGGTCGTAATCAACACACGAGAGACGCAGACCAACTCGTTCCAACGTCGATTAAAACAACTAAAGCAACCGAAACAAATCATCGAAACTATTGGACCTCTGACGTTCGTTTGTCTAGGGCAGGACGTCGAGATCATTATTCCACCACGACCCCCTTCCGTGG

>*TaCOI3-B*

ATACCGGATAGAACAAACTTTTTTAACGGGATCCAATAATTTTTTTTAGTACAATAAATCGAGTTTTGATCGGGCTCTAGGAATGCTCGACTCGCGTTCGGTGAAAAGTGTGGGACTAGAACTCGAAGCGAGTGAAATAACCGACGTTCTTTTAAAAGTCCTTTGTTGGGTACAAAAAGTTAGTCTTTTTGTAATGTTTCTGTTCCCGTTCCCCTGGTACGCTTGTTCTAAACGTTACTTCGGGACAACGTCACATAACCGTGTACAGTACGACTGTAGCCAGTTGAAAATACTTACACATACGAAATATACACGTACGGGTCGATTGGAATCGAAAAGAAAATCTCCTCAATTAGGATCGAGACGCATCTGATCGAAGTAGGTTAAAAGTACATCTATAAAACTCGTTAAAACACCGGGTCACTTTTTCTAAGGGGATCTCCGTTTCCCGTCAAACAGCCAGGTTGCCTGGCGGAAGGCGGTTGACTGCAGGAGCTGAGACACGAGGTGGGGGACCGTAAGAGCTCGTAGCGCGACCACAATCCGGGCTGGTGCCGTAGGAGGTACCACGGTACGTGCTGCAGGGAGGCGTCCCTGCGCGCCTACTCATGCGGGTGTTCTTTTTTATATACAATAAAAACAACATGAGGCATAATCTTTTAACACTAACAGTTAGTTATCATCTCTATTACTATGGGCGTTTTTTTAATCATCTCTATTATTATTATCATTTACCCGCATCCTAATCGCTAAACAACATCTCTTTATAAATTGAAACAAAACCTTCCAATAACTCTTATTAGAATGCAGACATACAGAAAATCCTTCCAGTAGAATGTATATATACGGTAAAGATTTAAGAATTAATGGATAATAAATGCACTAACTTAACTTAGTCGCGAACGGTTTCGTTCGTGCCTTGTTTTAAAGGGTGAAATTACTCTAAACTAAAAATTACCTTCTCTCTTTTTAAGTCACGGTCACCAACCACCCTCAATTCCGCCTTTTAACTCTGCTATTATTTGGCCACTTCTTTTTGATGGTTGAGGATTCAGGAGAAATCCTCATCTCTACTGTAAGACCACATAACCGTCAAACCGTGTGTGGTACAATTGTAGACGGTAGAAAATACTAACAGCGACGAAATAGACACGTGCACGTACCGACGTACGGGTCGATTGGAGTCGAAAAGAAAACCCCCTCGATTAGAGACGTATGTGACCGGTGTAGGTTAAAGGCACATCTATGAAGCTCGTTAAAACACCGGGTCACTTATTCTATGGGGGTATGTCTTTTTCCGGCTACCCGTCCGTCGGTTTTGCTCGTTCGTGCGTTATTACGTGGGGTGTTGGTGTTCGGATACGTTGCGCTAGAACGTGTTCTAGCTCCCACAAAATAGGTGATCAACATATTTAGGTGAACGGCGATTTGTACTACTGATTCATCGGCGACCCGGTCGTGACAGAAAAGTCGAGGGGTTCAGGCCGCGTTCACTTTAGCTTAGTTATTAGTTATTAGCACACTTTTACCGCATGCACTAGCAGCCGCCGCCGTGCGACTACTCGCAACTCGGCGCCGTGCATGTCACGATCCCTCGTCTCGCCTCGACGGGCATGAGCATCTGACCATGGCACGACCGACATGGGCCACCCGCTCCGGTAAATAGCGAGAGCAGGTGGCCGCCCCGCATGCACCGCGCCGTGGTGGTCTGCCCTGCCCTGCTCGGCGCGTCCATTTTCGACGACGGAGGGGTACAGTGCAGGCGGGGACGAGGATAGGGAGGGAAGAGGGAGGTTATTAAAGGAAGGGAAGTGAAGAGGGCAGGAGGGCGTTTCGGGTGGGGGCATCGTCTTTCCCTCCCTCCCTCCTCCTTAGAGGCAGAGGTGGAGGTGGAGGTACGGGGGCGGGGGGCGGCCCGGGCCGGGTCTAGAGGGCGCGCCGCCGGCGATCGGCTAGGCTAGGCCGGGC

>*TaCOI3-D*

TCCTTTCTACATACTGAAATTTCAGTCTGTTTAAACTTGTTGAGATAGGGGAAATAAGCACACCAATGTGTAATTTGGTTTCTTTCACTCCTTACTCAATTCAATCACCATGAGTTCAGAAACATCGATGTACCGAGAGAACGTTCGAGAGCGAGAATCAAATTGTATCTAACGTTGAGTTTGATACAAAGACCATCTCAACGATTTCATTATACTTGAGCCATGGCAACGTGTATTTCTTCAGTAGAAGTGGAAACCGGTCATTTCTATTATAAATCATCGTACATATTATACTTGAGTTTATCATCGGGACCGAGGTCACCACCACGATCTGGATACTGATGTACTACAGGTATAGAAATTTGAGAAGTATTAATGTACTTAAACCTACTTATACTAAACAAATTTATTCGATCTCATCAACTTAAACTTCCTTTATACGGGACCTCCGTTATTATTTCAACAATAAAATATAAAGGAATATAGTACTATTTACAAATAATAAGTACGATCTTANNNNNNNNNNNNNNNNNNNNNNNNNNNNNNNNNNNNNNNNNNNNNNNNNNNNNNNNNNNNNNNNNNNNNNNNNNNNNNNNNNNNNNNNNNNNNNNNNNNNNAGAAAATAAAAGAGAAAAAAAACGGGAGAACGGACAACTAAACACCACCGCTCAAGGTCATTAACACTGCCAACTAGACGAGTAACTACTTACTCCTAACTAGACCAGACACACAACCAACCAAAGCGGTTTACCTCATATGACCTATTCCTACTCAATTCACGGTCAAAGATATTAATCTATAAACAAACGAGATAGTACTCGACATTGTTTATGCCAGTACACTCCCTTCCACAGTTCAATCGAAGGTGTGTGAAACAAACCCGCGGTACTATGTAGTACCGTGATCAAAAGATGCTGTTTAACTATACTCGTCATATACGTTTACTGACGGCTTCCCTCTATATACTCTAAACCACATGAAGATACCAAATGGACAGCAAGGACAGGTCGTGTACAGGCTAGGCCAGAGATCAAACATTATTCATAACTGAGTGTTGTCGAGTGTTCAGGGTTGGTTCAGTCCGAATTCGTTTGATATTAAACTCCAACGTAGTCAGTACACTAGATAGAAGGGGTCCTCACGATAGCATGATGAAACTAACCTGAACTTGTTCTGATGTAGGCGGGGATACCCTGTATAAAACTTGGTTTTATTACAACGTTCAACCACCAAAGACAATGGTCAGTTAAGATAGTACAAGTCTAATCACACCGTGATACAGTATGGCAGAACAGGAAGGAAAAATGAGAAAACCGTCTTCGGAAAGGTACACAGGCACAATTGAAACGAAACAGACTTAAGACGTTGATGCTTCAACACACTCCCTTTAATAAAAGGGAATCTTACGTTCATACTAAACACACTCGAACAGTTCAAGTGTATACCTCGACCCTCTGATTTCCAATCGTACGATGTTTCCTAGACATCACGAAATCCATTTTACGTACATGTCACACGAGGAAACACGGACCTTTACGGAGTATACGTTGGCACTTAACTTTTTTTTTGACTGAACTGAAGTTACCGACAAGGCGACAGGGTGATAATTACTTTCCCCTGTCTTGTACCCCTGTAGCCCCGTACAAGTACTTCAAACTCTGCACAGAAATATCCAAAGGACCAAGTGGTCATTAGGTGAAAAGTAGCGTGAAAGGTAAGAAGGAATCGAATGTTGATAAAAACACGAGGAAATCGTTCCGTAAGCCTGACACCATGTAATTAAGTTTCACTAAAATGACAGTGACGTACTAACGCACGATAGTGACGTCATTGTACGATAAGATTTAATAGAACCGTCTAGGAACTCTTGTAATAAAGAACTTCTTACGCACTAATGACCACTTCCACCACTTACCGAAGTACTTGAACGACAGTTGTTAAGACAAGAGCACTGTGACTTGAAGATG

>*TaCOI4-A*

GTTGTTGATTTCATCAAGTATCCTCTCATCTTGTAAAGTTCATGCTCAATTCCTTCCATTTACAGTCAGAGTGTTGTCCAAATTATATCATTCTTATTCCTTCTCTATCTTGTTTTTAACCGGAGTGGTTTCAATATCTATCTTGTCACTTAACCTCTAGGTTCTCGTCGTATCCCATGTTCCTCTTTTCCATCGAAATGTTTTCAACTTTGTTCGTCTTCATTGCATTCTTTCTTTCAATTTGTTCAACCTCTCAAGGTTCGTGGTTTCACTCATTTGTCCAAGAAGCAACTTAGTTTTACCTCTTCTCTTCCTCTTCAGTTTCTCTCTGGTGCCATCCTTAGATCTCGGGACAAGATCCTCTTGTAGTGGTGGAGTGTTGTAACGCCCCAAGACCGATGCGTCAGGTGTCTTCCAGTTATTCGCTGTTGTTGCCTTGTCATTCACTTGCGTGTTGCATCTTGCCATGTCATCACCGCATTGTATCTGCATGTTTTCAAAACTTGCATCCATCCCGGTCTCCTCGTTTTTTCCGTTGTCCGTTCTGAGCCCGGACACACTTGCACACGCCGACGCCACGTCCGAAATAGCATTTTATAAGTGGACGGAAATGTTCTCGGAGTGGGATGAAAGTTGGCGTGATGTCTTATTATAGTGTAGATAGAACGCCTGTCAAGTTTCATCGCATTCGGAGTTCGTTTGATAGCCCAATAGTTAAACATATAACGGCACTATAGCCGGTCACGTCAGACGTTTTCGGTCTCCGAGGACTGCTGCCGGGTCTCTCTCTCTCTCTCTTCTCTCCCGCAACCCTTGGCCGTCTATACAGTCACAATCAGCCCACCTGCAGCCCACTAAATCCACCCTCCGACCGGAACCGTCGGATCGTGATTGGAGGCTCCGAAACGGTCGAGAAACCCCCAACCCTAGCCACTTTTGCTATAAAAGGAAACCCCTCCTTCCAAATCTGCACATCCCTTCCTCTCCTCCTCGTTTTCAGCGCCTCCTAATCATTAGGGTCGCCGCCTGATAACCCACAAGTATAGGGGATCGCAACAGTTTTCGATGGTAAAGTATTCAACCCAAATTTATTAATTCGACACAACGGGAGCCAAAGAATATTCTCAAGTATTAGCAGCTGAGTTGACAATTCAACCACACCTGGAAACTTAGTATCTGCAGCAAAGTATTTAGTAGCAAAGTAATATGATAGTAGTGATAACTATAGCAAAAGGTAACAGTAGTAAAAGTAGTGTTTTTGGTATTTTGTAGTGATGATAGCAATAGCAACGGGAAAGTAAATAAGCGGAGAACAATATATGAAAAGCTCGTAGACAAAGGATCGGTGATGGAGAATTATGCCGGATGCAGTTCATCATGTAGCAGTCATAACCTAGGGTGACACAGAACTAGCTCCAGTTCATTGATGTAATGTAGGCATGTATTCCGAACATAGTCATACGTGCTTATGGAAAAGAACTTGCATGACATCTTTTGTCCTACCCTCCCGTGGCAGCGGGGTCCTGACGGAAACTAAAGGATATTAAGGCCTCCTTTTAATAGAGAACGGGAACAAAGCATTAACACATAGTGAATACATGAACTCCTCAAACTACGGTCATCACCGNNNNNNNNNNNNNNNNNNNNNNNNNNNNNNNNNNNNNNNNNNNNNNNNNNNNNNNNNNNNNNNNNNNNNNNNNNNNNNNNNNNNNNNNNNNNNNNNNNNNNAGCTGTCCACCTTGATTCTAGAAAGAACAAAAAAAAAATGTTGTAGTTATACAGCAATTAAAACTGCTCAATCAGATGATATATTTGTCCCAAGAAACTTTAATTTCTTAGTTCGAAATAAATAAATGGTTTGTTCAGGGCCTTATGTGACAACTACAAAATCTTGTCTTATTAGCAATGTTTGAAGTGCAGTCCAGATATTGATTGACCAAAGTGTCATTTTCAGGTGCCAGGACCAC

>*TaCOI4-B*

TTTTTCTTCCAAACATATCAATCCAGCACAGGATCTGACAGTTCTGCCCCCAGCACAAAACCTACCCCACCCCACCCACAGAAAAGCCACTGTGCCCCACACCAACGGCCGTTCCTGCGTACCCCCCGGGCCGGGGCGTTTCCAGAAGTTTCGTGGTGGCGCCTAGGCGTGCTGACAGACAGCAACGGCCCACAGACACCCCTCCCGTTTTCCCGTCACCGCGTCTGGGAAAACGCAAAACAAAACGGGAGGCTGAGAAAAAAAGAGTCCGCCGCCGTATACGGGAGAAAAAAAAGGAAACGCAGTCCCCAGGCCGTCGCGGGTCCCCACGCCCTGCTCACGTGCGCAGCCCCCCCGCCCGCCACGCGCCGCCCTCGTGAGCCCCCCGTCCTCATCCCCTCCTGCCCCCCTCGCCGCCTATTAGAACCCGGCCTCGCTGCCTCCTCAGGCAGCCGCCAGTGCTCAACTGCGGCAGCTAAAACTCGATTTAATCCCCGGAAAATAGATAGATCGCCTCTGGGCAGCGGCGACCGCGCCGCCGCTTCGCTTCTCGGCTTGCTCGGCTGGGTGAGTAGAGCTCGCGTCCCTTTCTCCCCGTGTAAAATCTATCCTTGCTGTTTGTTTTTAGGATTTGTTCTAGTCCTGTTTGGTGCTCGGGAGCGGGATCGCCCCCGGAAACTGGATCTTTCCGTGGTTCTGCTTCGAATCGAAAGCCGTTTCCGTAGTTTTTTTTTTGTGGCTGCCTGTGCGCGAAAGCTACTTCGATCCTGGGCAAAGCTCCTGGTTGTTTTCTTGGATATTCCTAGGACTCGGATCCTGATATTTGCACCGGGTCCCTGTCTTCCGCGGACGCTTATCCGCCATGGAGGCTGGCCCGGTTAGCATTTAGTCCCTGAAGATTCGAGCTAATCTCTCCATTGAATTCTCTGGATTAGCTGATTAATGGAGATTTTTCTGGATTAGCCGATTCGCTTCTGGCAGCGGCGGAGCATCTTATGCTTTAGATTGGTTGTTCTCGCTTATTAAAGGTTTGCGTTGCTGTGCACGCCTCTGGCGGTTTATAGAAGTAGAAGATCCAAGAGGAAGGGGCCGGAACCTCTGGCGCTTGCTTGCCGTTTCCATTTTATTTTGGCTTTGTGCTTTCCACGGCTGCAGACCCCAAAGCTCCTTTTGAGAAGGTCTGCGTTGTGCAGAGTTCACTCTCCCGGGATTTTTGCGCAAGTTCCTCGATTGGAAGTTGCTAGTTGCCACCCGCAAATATATTTGATTCTAGGTGATCTCATAGATTGGATTGTTGATACAGAGGTGCTGATCCTAAGGCTGTTCATTCTTACGCCACTTCTAGCTGACTTTTGGTTCCTTCTGAACGCTTTGATTGACTTGAGAGATTAGTGCAGTAGTCGTTGCTTCTTGAAGAATTCTTTTCTCGAACACGCATCTAAATGCGTGACATTTTGTATTACAAGGAACTCTGCCGTGGAAGAGATCTCCATGGTTAACAACGGCTTATTGGCATAAGCCAACCAAAAAAATTAAAGCACAGGGCAAGAAAACGAAACAAAGCAAAAGCAGAAAAGAGAAATCAAGTACAGGGCAAGAAACGAAACAAAGCAAAGAGACAAAGGGTGGCACTACTTATCGAGGCAATAGCGTCTAGGTTCTCGATTGTATTATTGCTCCATTCCTCTGTTTGTGTTCCCTACCTAATTTTGCGGGTATTTATCTTCCGTTCACTAGATGTGACGTGTTTAAGTAAAAGTAGTAAGAACCCTACCTTGATCAAATAATTGCTAGATCTCGGTGAGGGCTTCTGACAGCGACATTCCAGTATGGTCTAGCGCATTCCTGGGAGGTTACTTGGGTGCATTTACGTTTTTGTTGCGGATACTTATTACTAATCTAACCTATTGACTCTGTTTATATAAGTTTGCTAATAGCTGCACTACTGAATGCAGGTTTATCTGTGA

>*TaCOI4-D*

NNNNNNNNNNNNNNNNNNNNNNNNNNNNNNNNNNNNNNNNNNNNNNNNNNNNNNNNNNNNNNNNNNNNNNNNNNNNNNNNNNNNNNNNNNNNNNATATCTTCAAAGAAAGTCGCATCATTCGACTCCATGATCGTACCGACATGCATGTCAGGTACCTCAAATTTTACAACCAAGAATCTATAACCAATGCTATGAAAAGCATATCCCAAGAAAACACAATCCACAGTCTTTGGTCCAAGCTTGTGCTTCTTTGGAATTGGCACGTTGACTTTCGCCAAACAACCCCAAGTTCGTAGATAAGAGAGCTTTAACCTTTTCTTCTCCCATTCCCCGAATGGAGCTATCACTTTGTTCTTTGTGGGAACTCGGTTTAGGACATGACATGCCGTCAATATCGCTTCCCCCCCATGCCTTGGAGAGACCCGATGTGTCTAACATAGCGTTAACCAAATCAGTTAGAGTATGGTTCTTTATTTCGGCCACCCCCATTTAACTGAGATGAATAGGGAGGCATCCTCTCATGGATTATACCATGTTCCGCACAAAAGGAATCAAATTCATTTGAGAAATACTCTCCACCACGATCGGACCTAAGCCTCTTGATCTTTCGATCAAGTTGGTTTTCCACATCAGCTTTGTAGATCTTGAAATAGTTCAGAGCCTCATCCTTAGATTTCAGAAGATACACATGACAGAGTCTAGTGGAGTCATCAATTAACGTCATAAAATACTTCTTTCCACCTTTTGTCAAAACACCATTCATTTGACATAGATCTGAATGTATGAGCTCTAGTGGTGCAAGATTTCTCGTTTCCGCCGCCACATGAGACTTACGAGGTTGCTTAGCTTGCACACACACTTGACACTTAGATCCCTTGACAGTGGTGAAACTAGGGATTAAGTTCAACTTCACTAGTTGCGACATGCAACCAAAGTTAACATGACAAAGACGTGAATGCTACACATTTGGTTCACTATTGTTGCAAACATGATTAACAACTTTATTGCAAACGTATGATAAGGATATACGAAACATGCCTCCTGACTCATAGCCTTTACCAACAAATGTTCCATACTTGGATATTACAAATTTATTCGACTCAAAGACAAGCTTGTAGCCATCTCTACACAGAAGAGANNNNNNNNNNNNNNNNNNNNNNNNNNNNNNNNNNNNNNNNNNNNNNNNNNNNNNNNNNNNNNNNNNNNNNNNNNNNNNNNNNNNNNNNNNNNNNNNNNNNNAGATATATTTCTTTTAGGTGCTCTCGTAAGGAGAGATTGGATTGTTAATACGGAGGTACTGATCCTAAGGCCATCCGTAGCAGTTTTTCTTACGCCACTTCTATCTGACTTTTGGTTCCTTCTGAACGCTCTGATTGACTTGACAGATCAGTGCAGTAGTCTTTGCTTCTTGAAGAATTCTTTTCTCGAACACGCATCTAAATGCGTGACATTCTGCATCAGAAGACACTCTACCTCGGAAGAGAACTCCATGGTTAACAACGGCTCATTAGCATAAGCCAACCAAAAATATTAAAGCGCAGGGCAAGAAAACCAAAACAAAGCAAAAGCAGAAAAGAGAATATAAGTACAGGGCAAGAAACGAAACAAAGCAAAGAGACAAGGACAGCACTACTTATCTAGGCAATAGCGTCTAGGTTCTCGATTGTATTATTGCTCCATTCCTCTGTTTGTGTTCCCTACCTAATCTTGCGGGTATTTATCTTCAGTTCACTAGATGTGACGTGCTTAAGTAAAAGTAGTAAGAACCCTACCATGATCAAGTAATTGCTAGATCTCGGTGAGGGCCTCTGACAGCGACATTCCAGTATGGTTTAGCGCATTCCTGGGAGGTTACTTGGATGCATTTACGTTTTCGTTGCGGATACTTATTACTAATCTAACCTATTGACTCTGTTTATATAAGTTTGCTAATAGCTGCACTACTGAATGCAGGTTTCGCTGTGAAA

>*TaCOI5-A*

GCAGCGGCTAGGCTCAATTTCCCCTTCGGCACATGTTCGGTCGACCTCGTCCCGCTGAAGTGAGGGGTGGTGAGCTCGGCTATGGCGCGGGAGGACCGCGAGACTGAGGCCGCCACCGAGGCCTACATGCAAGAGCTCCGCCGACAGCACCCGGAGCTCGTGGAGGCGGAGCGAATGATCTTTTCCGGTGCATAGGGCGGCGAGGTTATCATGCTCTCCTCCGATGACGAGGTCAAATGCAGCGAGGACGTCGGTACGGAGGGCATTGAGGTGGGAGGCGAGGATGAGGAGATCGATGTCGACGAGTGGAGGAGTGTCTTCCCGAACGAGCCCAACGACGGCACCGGCCCGAACCCGGCTGCGGGACACTGTACCTAACGAGGAAGGATTGGCTCGACCTCCACTTTGACCGAACGTAGTTTATTTAGTTTTAAGTTTATGTTTTATGTTCGGTTATACAAAACTATCTACGTTTATGTTTCAACAATAACACTAAGAATTACGATATTTGGTTGTTGTTAAGGATGTTGCAGCTAACACGCCTTTCCGTTTTTTAAACAATAACTATTTTTACTTTAGACCTGCACAATACCGGTTTGAATGACTATATTTGCTTGTTTTCAAATGTTATGCTTGTTGTAGTTAACATACCTTTCCACTTCTTATTTTGCTTAACTAGCATGCTTAAACCTGCACACTAAAGGTATCATTTGTAGATTATGTTGTCTACCGTGGCTATATTTGGTTGATGTTTAGCCCGTTGTGCTTAACATGCGTTTATATGTACTCATACATGATAATATTGAGAACAATTGTTGTTTTCCATCGAGTTAGTTTCCTCGCGTTGCTTATATATACTCAATGTTCAGGATATTGGTAGCTGGTACCATATAGCCTGGGGGCTGATAAGGGACTAATCATCGAATTGGACATTTCACTGAATATATATGTAAGTCATCTATGGGTAGGTTAACTAGGGTCATATTTAATATATTTGAGGCTACATAGCAACACGCATTATACTGAATGACTAAATATGCAATCCCAGGCTACATAGCAACAAGGAAGAGTGTTACACTAATGTTCTAATGATGTTTAGATATACATGTAGTAACATATTATATAATGGGATGAGGGGAGTGTTGTACTACATCATTTTGCAATTGTTGTTTAGCTTCTAATATTAACTTGGTGCTTGTAGTTACATATGTCATTGCCAAAGATCCACACTTCGGCCCTTTCTGCGTATGGGGCAATGAGATATTCATGAACCAATAACAAGTGAGGAAACTGATAAAGATTGTTATTAAAATGGGTCAAAAGATGTCAATCGAACTATTTGTTTACACTTTATGCAAGACAACAGTGAACTACGGGATGGTAAGTAAGAACTCCGTAGCCTTCTTTTTACTGTCCGTAATTAGTTGTGTTAGATGACAATGTCTGAATTTTTTACCTTTATAGTGGATCCTGAAGCAGTTTACTCAAGATTATCTCTCAAACTACATGATTGGCGCTCGGGCATCACAGAGGGTTGGACTAGACTCGTGTGTGCCTTCTGCATCGAGGAGATCCACTACTAGGAAAAGGACTACTAGTGGCGCACCTGTTTTCGCTACTAATGGCGCACTACAGGTGCGCCACTAGTACCACGCCATTAGTATCTGGTATACTAATGGCGCACCACGCAGTGCACCATTAGTATAGCCCATGGTGCGCCATTAGTATGCCTCCGAGGGGGCCATATTTACCCATGTGCTTTGGATACTAATGGCGCACTGGTGGATGATGCGCCATTAGTGTGCTCTGGCTTACTAATGGCGCACTATGTGGTGGTGCGCCACTAGTATGAATATTAGGNNNNNNNNNNNNNNNNNNNNNNNNNNNNNNNNNNNNNNNNNNNNNNNNNNNNNNNNNNNNNNNNNNNNNNNNNNNNNNNNNNNNNNNNNNNNNNNNNNNNNAGGACC

>*TaCOI5-B*

AAGACTTCTAAATCCGCAACCAAAGTAGCCAAAACCTGTTTGCTCGATTTTCAATGCAATCAAAATTCTAATCCCGATTAGACGCTATTTTGATAAGCACCTATCCAGGGACCGGCTTTTATTTATCTTTTTCTTTTTAGATAGCATGCTTGCAGGCGATTATCTCTGATCACTTGCTTAAGCAAACAATTGTCTAGGTTTACCCACTTGTAAGCGATTGATTGCTTGATTATTTTTATTTTGGCTAGATTAGATTATTTGGCTTTGATTTTTTTGACTAGCCAGCCCTGTCAAAAATGGCCAGAAACCGGCCAAGCCAGCTGCCGCCGCCGAGCTGGTTGCTGCCGCCGCCACTGAACGCCCGCCCCGCCGAGTCACCACTGCCGCCCAAGGCCGCCGCCCCACGCACACGCCGCCGCGTGTCGTCGCTGCCACTGTCGTGCGCTACCGCCGCCACGTCACCACCGTCTCCGCCCGCCGCGCCGAACTCCTCTTTTTACCCCACCGCCGCNNNNNNNNNNNNNNNNNNNNNNNNNNNNNNNNNNNNNNNNNNNNNNNNNNNNNNNNNNNNNNNNNNNNNNNNNNNNNNNNNNNNNNNNNNNNNNNNNNNNNCCAAAACTGTGGCTGTAACCACGAAAGTAACTCTCAAGGAGACACAGCAGCGATATTCCGAGCTACCGAGGGGGTTGGAAGTTGTTGCGCCAGGTCCCACTCTGAAAAGAGGGGCCTATCTAGAAGCACAAACCGCCGAAGCGTGAAGCACGGTTGAGCGAACCGGTAGACCTCGTCTAGCTGTCGATGCGGGGACCGGCAGTCCAGTTAAAGCCTTTGAATTTCATGTGCTGGCTATAGGTACCTCTGAACTAGAAACTGCCTAAGCCCGGATACAGTCCTCGCGGCTTGTCAGTGCCACTCGTACTGAATTTAGACGACAACCTGTCATAAGCCCTATAGCGGGGACGACGCCGAGGACTGGATTTAGGCTCCATCTAGCGCAGCAGGTTCCTGCCCACCTACCTGGGGTGCTGCTTCCGGCGTATGAGAAGTCGCCATCTCGGCTTGTGTCTGAGGCGGTAATTCCTTTAGATGCAGTGGCCTGGGAGCCTGAACACAGGCCTACACCCAAGACTTGGCGCGCGCGGGTCTGGGTAGCTCAGGCTGACCCGAGGTCATTACCTCAAGTGGTGGCACCTATAGAAAGTCATGAGGGGGAACCCTCTTCGCGAATTAAGTAATTTCAGAGAGAGAAACAGTCTTCTGAGAACCGGCTTGATACAGACCGGGCTCACCCTGTGCTTGCTACGTCTTTTAGCGGAGGGTGGGTGGTGGGTGAAGTATCGGTAACAGTTACTGGATTGTCTGTACGAGTTAAAGCTGAGGCTACTGAAACTGCCATACCTGCAGCTGCGTCCCCTGCTAGGCCTTGGTGGCGGGTACCCCGCGACCTGGCGGTGAAGTAGTATACTGTAGATGTACTACCTTTACGGATTCCTTCTGTTGCCACTGTTTTTCCTGTGTTACCTCCTATTTGGGGGGCCTGTTTTTGTTTTTGTTGCCGCGTCCGCGGAGAGATTCAGGTCAGTGTCATTTTTATCGTTATTGTCGCGTTCCTTTTTACTGTGAGGCCAACTGAGGCTTCCGTTGTTATACTTGGGTTGTTACCTCGGTCTTCTCGATCCGGTACGACTTGTTTACGGGCTAGTGTCACTAGGGCTCCCGGCTTGAGTAGTTTGACGGATGCCTCTCCTCCTATCAGACCAGCTACTACGTAAGTAGTAGGGCCTTTTTATAAACCTTGTTCTATTNNNNNNNNNNNNNNNNNNNNNNNNNNNNNNNNNNNNNNNNNNNNNNNNNNNNNNNNNNNNNNNNNNNNNNNNNNNNNNNNNNNNNNNNNNNNNNNNNNNNNTGGGGGCAACTTTAGGCGCCGCCCTGTCTAGCCCCGCGTCTAGCCCCCGGCCGAGGGCCGC

>*TaCOI5-D*

ACCTCGTTATACTACAACGCCGTTATCGCATAGTTCCAGGTAAAGGTCGGCCTCTTCTTGACTACTTACAATATACACGCTAAGTATGTAAGCAAACAAACTTCACCTAAAGTTAATAAAAATCAGATAATTATCGATATAGAAAAAGATATGTATATTTTAAACCGTACGTAATACAAATGTCTACGTTGGTAATTAAAAAAATTATCGGGCACCGTTACGTGCCCGTAAAATGATCATATATATTAAAAACCGGAGCACTTCCTCTTTCATAGCGAGATCGAACCCCCTCCGAATTCAGTTACAATATAAGTACTGGGTTAGTACTCGAGAGTTCTCTTTAATAATAGGTCGTAAAAATACGAACCGAAAGAGCATTACTAGTTTGGTACGAACTATGAAGAACACAACCAAGTAAATACTTCTTCTGATAACTTAAGACCACCCTAGAAAACCTTTATTAATTTACGTTGAGACTTCTAACCCTTGAGCTGCTTCCATTTCTCAATCCATAATTTGAATTCATACTAACACAATTTAGAAAATACCTATNNNNNNNNNNNNNNNNNNNNNNNNNNNNNNNNNNNNNNNNNNNNNNNNNNNNNNNNNNNNNNNNNNNNNNNNNNNNNNNNNNNNNNNNNNNNNNNNNNNNNCTACCCAACGCAAAATGATGATGTGTACTACTAAAACTATCTATATTATACGTACACGAACGACGAGGATGCACAAAGGTTGTGCTATTTTAACGTTCTTTGACGAATACGATACATAACCGGAAAGGAAACACACGTACTTAACAAGAAAATACTGTACGGCTACGTAGCCTTCTCTCAATCTGAAGCAGTAATGTACTATATACAATTAAACACGAGTGATGATTTAATGTTTAGTAACAATTAATTTTAACCGAAACTATATGGAACCCTAGGCCCACCTATGTAATGAACTCGTGATATACGGATCGAATTACCGAAATTTCTTTCGCGACGGTCCCTCTGTTGGGCCTTCAAAATCTCTCAATAAATAAAGACAACGTACGAAAATATTTCATGTTTAGGTAACATAGTATTTTTATTACACGGTTCTAAACGGAAATCCTACAAATGTAACGAACAACCAAACACGCCACGTTTAGTCTTTGAAACCGACATCACGCGCTAAAATGTAAAAATGACCTTACAGTTTACCAAGACTTTGAAAAACGTGACAGAAAGATAGGTTTAACAAATAAAAAGGATTAAAAAAGGTTTAAACACCTCATGGTCTTCATACCACTTACAAGTCTAATGATGTCTGACAGGACGGTGAGCTTGGGTCTCGATCGACCAAGAGGGGGTTCCAACAGTCTTAGCACTACGACTTAGCTTAGCCTCGAGGCTACCAACCTAGCATTTAGTATCTTAGAAGTGATAGTACTAGTATCTTTGCATCTAAGATCTCTGATTTTAGCATCTTAGTCTCCCCAATCAAACCTAGCATTTCAGCATCTTAGTAAGTTATCTTAGCGCTAAGACTGTTGGAACTAGCATAGATGTTAAAAAAATAACAAGGTACGGTTATAAGATGTTGAAAATGTATGAAAACCGTTAAAAAATATGATAAAAACCCTGATTGTATAACTAGGTCACGGGTCACGGTCAAGTACAAACAACGTACAAAAAACAAAGCGTCTTTTAGGTATAGTTTGCCTCATGTTTGTCCTATTTTTGACTGCCTCTAAAAAAACCCTTATATACACTAAAAACCCTTCTTCTTAGTTGCGCTCTGCTACGGGCTCCCCCGGTGCTCCGTCCCCCNNNNNNNNNNNNNNNNNNNNNNNNNNNNNNNNNNNNNNNNNNNNNNNNNNNNNNNNNNNNNNNNNNNNNNNNNNNNNNNNNNNNNNNNNNNNNNNNNNNNNGGTGGGGTGTGGGGGCAACTTTAGGCGCCGCCCTGTCTAGCCCCGCGTCTAGCCCCCAGCCGAGGGCCGC

>*TaCOI6-A*

GTTCACCGCTTTGTACTACTTCTCTAGAAGGGGTCTATATCTCCGAAACCATTACAATAAACCCAATGATTAAGAAGATACGAAACATGAACATCCGAGGAGCATAATCGTATGCGAATGAACGATTACAATCTGAGGGAGGTAGATGTTTATATTCTGCTACAAAACCTATAAAGTTGTACTTGATGTATGTTTTTTTTAATGTAATGACAATCTTGTAGGATATAACGAAAGAAGCAAGGTTTTATGAACAACACCAAAAATCAAGTCAAGTTTAAATTTGACTTTGGTATCGCTCACAAAACCTTGCCTCCCTTATAAACATTAGCTTCCCTCATGAACAGAGGATACTTCGTGGCTATGCCGCTGTGACTATGCCACTATGCCTATGCCGCTATGCCCTATGCTGTTAAAGATTTTTATTATTATGCCGCTATGCCGTTCGTATATATTTAAAGATCAAAACATCGTTCGTTAAAATACTAAAACAGGTTGGTTGTAATGATGTAATCGTTCGTGGTTGTCTAGCTTCATGTCCATGTAGTGTGAATACTGATACTCTCTCATGATGATGATGGATGTAGTTGCCTAGTTACTATGAGATGTAGTTGTCTTCGGTCGCCGACGGGTCGTCGGTTAGTATGTAGTTGCCTAGTTGATGTGTAGTCATACGTAGTCCCGAGCCCGTTTATCAAGTGGAACGCCCCCGCCGCCTCTTATCGTCTTCTGAGCTCCGTCCCTCCGCTGAGGTCCGTCTAGTCGTCAACCCCGCCGCCNCCATCGCTCGATGACGACTGCCGCCGCCGTCAGGACTCAGTCGGGAGCTAGGGACCTAACGGCAGGGTCTGCACAGCGCGCACAGGGCCGGCATAGCCGTAATAAATAAATAAAAAATTTAGTTTTTGACCCTATGAAGACCCTATGGGTATAGATTCGGCATAAACCACATAGCCGCATAGCCGCTGCAGGACTTGCCGCTATGTCTCTTAAGACTGCATAGCCACGAAGCATCGAACAGAGCTGCAACCACGATCTTTTAAGAGTTAACGCTCGTGAACTTTTTGTCTCGTTCCGGTTTACATGTGTTGATCCTGATGAAACAACCATTTGAAAAGACACTGTAACCACGAGCTAACTTGTTTGCTATCTCCTAGTGCGGTACCTGCCGGGTTCCTATGTCGTGTGTGGAGGAGACGAGACCAGGTTTCAGGTTTGGTTTTACCTTCTATCGGTGTAGCTAGAAACGGGAACCTTCCGATAGATAGGTATACTTGTCAGAGGACAGCTAACAATGAAGCCTGGGTGACCGCTCTNNNNNNNNNNNNNNNNNNNNNNNNNNNNNNNNNNNNNNNNNNNNNNNNNNNNNNNNNNNNNNNNNNNNNNNNNNNNNNNNNNNNNNNNNNNNNNNNNNNNNACCTGGCGGCTAGGCAGGCCTGCCCCCGACCACTGCTTGGGATTGAACGAACATGACAGGCCTACCAAAACTAGAGCATAAAAGAAAGGAAAGACACTCGACACTCCTCAAATTAGGTAGATCGAAGTACGCCTAAGCAAGCGACGAACAACTCGACTAGTATCGACACCCAAGGAAAAGGCCTTCTTTTTCTTCTCCTTTAAAACCCCAAGGTCAGGTCGGGTTGGGGTACCGTGAGAAACCCACACGCCAGCCTCCTGTAGAGACCCAAAAGAACGCTTGTGAAGATGAATACCTTTCTACGTTTTTTAAGCACGCTGACAACGAATTAATTGACACAATTAAGAACCTTTAAATACTAGTTCATGAATAAACTCATACTTATATATAGTATATAGTCGGACATGTCATGGTTAAAGTTCGTTTACCATCAATTAACCATGGGTCACGATTGTGTTACAAAACAAAGAACACGACCACACTGACACTTGTCCAGTGCCGGCCTTTGACATTTTCAATTAACACCAAACAATCCTAAGACCCTCCGCCGTTG

>*TaCOI6-B*

TCATGTGGTACACCTTGCCGGTATGGTTCACCTTGGTCATCATGATGTTGATCAGCCTGCCGCTCTGCGTCACGTACGAGATCCACTGCATCTGGGGATGAAGCCCCTTCCTCATCCTTCCAACCTGGAACCATACGCAGGATCATCAGACAAAAGGGATGAAATAGTAATACATATCTATTATCGGGTTTCCAGATGTTAACCATGGAAGAAACATCCATTTCCTCAACAGACACGCGTAAGTTACAAAATCATGATCAGCATTCACCTCTCAAAAATATTACTTACAAAAACATGATCAACATTCACCATGATTTCCCTAAGAAAGCATACACCATGGTTAACGTAGGACCTATCATAACAATTATTCCAAGTTTGCAATATAGCAGGAGTAACACATCCAAAGAAAGGGTTCGGTCATATGATTGCTGAGAGACTATAAATGGTACTGGTACTTTAAAATTCTCGACTCGCTGTGGTCATGTTTGGCCACTAGAACACTTGGTTGCAAAAGAAGTTAGCATGGTCTGCAGACGGACATGGAAATATGACTTGAGCAGCTCCATGGGCGATTCTACTGGACAGACGCTGTGCTCTAGTTTCCAGCTGCTGCTGATCTGATTCTGTTAAGGTGGTTAAGAACTGAGCAGTGCATATTTTGATTTAGGCCATTTGGCTACTTTCGGTTACAGATAGTCTGCACCTGCATACTTATTGTCCTAGCCGAGCCCAAACAGATCAGCGAATCTACTGTCCAAACAGATCGAACAGCTAGGGTTACAGTACCTGGGGAGGAATCACCGACGAAGAAGAGAGGGGTGCTGCTGGTCGCCGGCCTGCCGCTCGTCCCTGTCCTCCTTCGCGCTGCAGCTCGGAGAGGAAAAAACAGATCAGGGTGGAGAGAGGGGGAGGGGAACCAGAGCAGGGAGAGAGGGCATCCAACGCCGCCGCCGTCAGCCACCCATGGGGACGCCCGAGAGAGAGAGGGGGCAGAGCAGCCCTAAGGGGAAGGGGGCGAGGGAGAGGAGAGGGGCAGCACCTCGACGCGCGCCGCCGGACGCCGCCGCTGCTCTTCCGCTGGTGACTCGTGTGTGAGAGAGAGAGGGGCGCTGGAGAGGGTTCTGTCAGCGAGGGCCCACCTGTTAGTGAGAGGGGAGGCGGGGTGGGTGAGATAATAATAAAGCGAATAAAAAAACGATTCGATAATAACATAGCTCCATCCCCCTCGTTCATCTTCATTCCCCTCTGACGGTCATCGCGCTCCACTCCGCCGGCCGAGCACCCGCCCCCGGGTCCAGGCCTCCTTCCACCGAGGAACGCCACCTCCAAGGGATTCTCCTCTCAATCCACGGTCGCCGCCGTCCTCCTCCTCATCCGCAGCTTCTCCGCTCCACTGGACTGCCGATCCGTCCGGACGGGGACTGGTGACGAACCCTAACTTGCTTGTGCTGCCCGGATAGTTTCGATCTCGTCTTTTCTTTTCTTTCCTTGCCGTGATGTGTGAGAAGTTCAATCCGTCGAGTTTCCTGCGGATTCTGTCGCTGCTTGGTGAGCTGATGATAGCTGTGAGTTCCTTTTCCGGAAGAAAAAAAAAGAAGAGGAAATTTTGGGGTTCCAGTCCAGCCGAACCCCATGGCACTCTTCGGGTGTGCGGTCGGAGGACGGCGCCGGTCGTGCTCGGTGCTTTTCTTGCGAACGCTTTTACTTTTGGAAATATGCAAAAATTCGTGTGACTGTTGCTTAGTTAACTGTGTTAATTCTTGGAAATTTATGATCAAGTACTTCTTTGAGTATGAATATATATCGCATATCAGCCAGTACAAGTACCAATTTCAAGCAAATGGTCGTAAATTGGTGCCCTGTGCTAATACAATCTTTTGTTTCTTTGCTTGTGTGACTGTGAACAGGTCATAGCCGTAAACTGTAAAAGTTAATTGTGGCTTGTTAGGATTCTCGGAGGCGGCAAC

>*TaCOI6-D*

GTCTCCTCTGCCCCGTCGACCCGCGCCACCACCGCCGGACGAAGGCCCGCCTCCCTTCCCTCCCGGCCGGCCCAGCCCCGCCAAGCGCAACCTCGAGCCCAGCCCGCCCCTTCCTGAACCCCTCCCCGAAGGCACCACTCGTCGCCGCCGCCCCTCGTCGGACCGCCGCGGTTGGGATCGTCTTCCTTCCAACCCCTGCTTTCCTCAAACGCCCGTGTCCTGTCCTCCTTCCTCAACGCGAGCCCAGACAACCCAGCCCCTCACCGTAAAACCGGCATTAAAGTCACGGGTTATCGCCATCTCATGAACAAATTTTGCTTGTCAAAATAAAACGGTCTTTAACCAGTAAGATCTAATGTGAACCTCAACACTCTACTAGCACCCGTTAAGGACCCCGTCCCGTCATTAAGAAGTGTTAACTCGGTTTTCTTGACCGGGAATGACAGGTAGATGTTGTCTTTTAACTTTGGTCTCTTTTTCATTGAACTTCTATACTGTACGTGTTTTGACTTGGTACAGCAGGATGTTTGACGTCGGTTGGTTAATCTTTCTTCGTACGGTATGTTTTTTCCATATTTGTGCTCGGTAGCGTATGTGTGTTTAGTGTTTGCTTGTCGTGAAGTCTAGTCGTGCGTGTGTTAGTCGGTTAGATGACGGGTTCGACTAGCTTGTCCATCTATCCCAATAAATATGGACCCCCCGTTTGGTGGTTGCTTCTTCTCTCTCCACGACGACCAGCGGCTGGACGGCGAGCAGGGACAGGAGAAAACGCGACGTCGGGTCTCTCCTTTTTGTCTAGTCCCCTCTCTCCCCCTTGGTCTCGTCCCTCTCTCCCGTAGGCGGCGGGGCGGCGGCAGCCGGTGGGTACTCCTGCAGGCTCTCTCTGCCCCGTCTCGTCGGTGTTCCCCTTCTCTACCTCTCTCTCTCCTCTCCCCGTCGTGGAGCGGCGGCGGCGAGAAGACAACCACTGAGCGCACTCTCTCTCCCTCGCTCTCCGCGACCTCTCCCGAGACAGTCACTCCCGGGTGGACAATCACTCTCCCTCCGCCCCACCCACTCTATTATTATTTCGCTTATTTTTTTTGCTAAGCTATTATTGTATCGAGGTAGGGGGAGCAAGTAAGGGGAGACTGCCAGGCCGGCTCGTGGGGGTCCAGTTCCAGAGGAGGGAGGCTCCTTGCGGCGGAGGTTCCTTGAGAGGAGAGTGAGGTGCCGGCAGCGGCAGGAGGAGGCGGCGGTAGAAGAGGCGAGGCGAGCTGACCGCTAGGCAGGCCGGCCTGCCCCTGGCCAAGGAACGACCACTGGTTGGGATTGAACGAAAATAATGACAGGCCTACTAAAGCTAGAGCAGAAAAGAAAGGAAATACACTAGTTAGATTGGGTCTGTGAGCTTAGGTGGGCGCCTAAGAAAACGAAGGACCACTCGACTACTATCTATCGTCGCTCAAGGAAAGGGGTTTTCTTTTTGTTTTTTATGTTCCTTTAAACCCCAAGGTCAGGTCGGCTTGGGGTGTCTCCTGTAGAAAGACGCCAAGCCAGGCAGCTAAAGCTAGAACCTCGACTGTTGAAGATAGGGAAAGGGCCCAAAACCTAAGCAGAAGTCACAAGACTAAAGAGTTGCTGGAACCCATAAAAGAGAGTCAAAGTACCGCACGAAAAAGAAGGCTGATGAAAAGATAAATATCTTTCTGCGTTTTAAAGTACACTGGCCAACGAATCAAATGACATGAGACAGGCCTTGGGCGAGATTGAACACAATTAAGAACCCTTAAATACTAGTTTATGAAGAAACTCACTCATATATCGTGTATCGTTTCTCAGCCCATTGTTAAGTTCGTTTACCAGGATTTCACTATGGGTCAGGATTGTGTTACAAGGCAAAGAACACACACACACTGACACTTGTCCAGTGTTGGCCTTCGACGTCTTTTATTCCCGCCGAATAATCCTAAGACCCTCCGCCGTTG

>*TaCOI7-A*

TAATAAACACTTCGATTTGAAGTAACGATTCATACAATGGTATGCTACAAAGAAGTTTTCCGTGAGGGCTACTTACAACACGGAATACCCTAGCTCTGATTTCCATGGAACTCACAATAATCGAATGCTGGTTCTATAGGATGTACAATGAAATCACGTAAGTTCTAGTCACTACTTACGAATTATCACGTTCTGACCTGGTCTATACACTACCCCCTCGCGTCTNNNNNNNNNNNNNNNNNNNNNNNNNNNNNNNNNNNNNNNNNNNNNNNNNNNNNNNNNNNNNNNNNNNNNNNNNNNNNNNNNNNNNNNNNNNNNNNNNNNNNCGGTACATCCTCATGTACCACCCAACAGAGTGACCTTGGCAGGGATTCTTGACTCAAGACACATACAACAGGCTACTGTTCTATGATGGTGTGTAACCCAAGACCATTTAGCTGGGGAGAGCTGAATAATCGGTTGAACTAGAGACAGAACCTCAACGTTTATCAAAAACCACAAACATCCATCACGATAAAAAGACGGCTCACCGTGGGCCGTCCCACCTGAACCCTGTCTGATCCGTGGCACCGGGCCACATGGCTCACCGTGGGCCTACCACCCGAGTCCTTGGGACATGTGTAGCAAACCTAGGCACTGGCTTTGGGGCCGGCCTAGAGGAACGCCTACCTTGGACTTATCCGCTATTTGGACCTGATCTCAGAACACACCAATCAGTCCAGCACCGGCTGTGGGATCGGTCCGAAGGTGAACTTCCAACGACTCTATGTACTGCACATATACCACTATTCACCACTCTCTCACACACTTCTTCATGTGGGGACGTCCCAATAGTACTAGATAAGCTTATCGGCACAGGAGCCAATAGCTGAAGAACCTACGAATGCATCATGTATCTGTTGAATTTCACCTATGAGATTTTACGAGTTCTGTTCACACTCACGATACCTACCGGAAGANNNNNNNNNNNNNNNNNNNNNNNNNNNNNNNNNNNNNNNNNNNNNNNNNNNNNNNNNNNNNNNNNNNNNNNNNNNNNNNNNNNNNNNNNNNNNNNNNNNNNCCGCGGATGCAGAGGGGGACCCAGCGGCGCGAGCCGGGGCGGATGGGCGCGGAGCTCGCGTAGACGGAGTTCGCCTACTGGCAGAGGCTGCTGCTCGAGCGCGAGTAGGGGTTCAGGAAGGGCGAGAAGTTCCTCGAGAGCGAGCAGACGACGCTGCCGAAGTGGTGGGCGCCGGAGCGGCAGTAGCGGCTCCCGACGGCCATGCGAAGGGAAGGGAAAGAGCAGAAACCCATAGTTGCTTGCATAGTTGAAATGCCCGACCCTGTCATAGCATGACGGTAACCGCGTAAACAAGTAAAGAGTCGACGTAGTACGGTAAGGGGCGAAATAAATTACACGTGCGGATGAGGAAATACGAAAAAAGCCAAAATAACCAGGCTGAAACGGGACCTAGTCTTAGACTAGATCCGATGTGATCGAAAATAACGGCGCGGCCCAAGAGTAGGGATGTGCAAGAGAGTTTCGATCTAGAAATAACAAGAACTCGAGATGAGTCAAGCACTGTGAACCATCACGGTGTGAGCTTAGAAACGGCAATCATCGTGAGTTGAAAACTAGCAAAAGAACAGTACCAATACCGTTTATCAAGATCGTTTGTTTACAGAGTTTGATACTATATGATCATGAGTTCTCACCATAGTAGAATACCTAGCAACGTAAATAAAAAATCCGTAGAAGCCCATGACCTAGACTGACTTCTAATAAAGGTACTCCTCCTCTCGCTCCACCACCTAACCTAGAGGTTCAAAGGTCTCTCGTTGTGCAGCGACCTTAGCGAACATAAACTAACACAATCACAGGGTAAGTTGAAACTCCGGGACCTCCGTGAACAACGTGCGAGTGGTCGAGACNAGACGCAGCAGACGCGCAGTTACTAGTACACAGCTATCTCGTCAACGCAGCAGAG

>*TaCOI7-B*

TTAACCATCAACACTTGATGCTCCTTCTTGATATCTACCTCCGCAGCTTTTAGTATTGCGAAGAGCTCAGGAATAGTATTTTCCATCCCTTGCATATTGTAGTTCATCACGAAGTCTTTGTAGCTTGGTGGCAGTGATTGAAGAACTCTGTCAATGACACAATCATCTAGAAGATCAACTCCCAACTGCGTCAAGTGATTATGGTACCAAGACATTCTGAGTATGTTTTCACTAACAGAACTGTTCTCCTCCATCTTGCAGCTATAGAACTTGTTGGAGACTTCATATCTCCCAACTCGGGCATTTGCTTGAAATATTAACTTCAACTTTTGGAACATCTCATATGGTCGATGACGTTCAAAACATCTTTGAAGTCCTGATTCTAAGCCGTAAAGCATGGCACACTGAACTAACAAGTAGTCATCAACATGCGACTGCCAAGCATTCACAATGTCTGCAGTTGCTGGGGCGGGTGGTACACCTAGTGGTGCATCAAGGACATAATTATTCTATGCAGCAATGAGGATAATCCTCAAGTTACGGACCCAGTCCGTGTAGTTGCTACCATCATCTTTCAACTTAGCTTAGGAATGCATTAAAATTCAAGGGAACGAAAGCACGGGCCATTGATCTACAACATAGATATGCAAAAACTATTAGGACTAAGTTCATGATAAATTAAGTTCAATCAATCATATTACTTAAGAACTCCCACTTAGATAGACATCCCTCGAGTCATCTAAATGATACGTGGTCCAAATCGACTAAACCATGTCCGATCATCATGTGAGATGGAGTAGTCATCAATGGTGAACATCTCTATGTTGATAATATCTACTATATGATTCAGGTTCGACCTTTCGGTCTCCAGTGTTCTGAGGCCATGTCTATACATGCTAGGCTCATCAAGTTTAACCCGAGTATTCCGCATGTGCAAAACTGGCTTGCACCCGTTGTATTTGAACGTAGAGCCTATCACACCCGATGGTGTCTCACCATGAAGAACTTTCACAATGGTGCATACTCGGGGAGAACACTTATACCTTGAAATTTAGTTAAGGGATCATCTCATAATGCTACCGCCGTACTAAGCAAAATAAGATGCATAAAGGATAAACATAACATGCAATCAAAATATGTGACATGATATGGCCATCATCATCTTGTGCTTTTGATCTCCATCACCAAAGCAACGTCATGATATCCATCGTCCCGGCTTGACACCTTGATCTCCATCATAGCGTCGTGGTCTTCTTGCCAACTATTGCTTCTACGACTATTGCTACCGCTTAGTGATAAAGTAAAGCAATTACATGGCGTTTGTATTTCATACAATAAAGCGACAACAGTAAGGCTCCTGCCAGTTGTCGATAACTTTTACAAAACATGATCATTTCATACAACAACGTATATCACATCATGTCTTGACCATATCACATCACAANNNNNNNNNNNNNNNNNNNNNNNNNNNNNNNNNNNNNNNNNNNNNNNNNNNNNNNNNNNNNNNNNNNNNNNNNNNNNNNNNNNNNNNNNNNNNNNNNNNNNGCTGCCGGTACGGTTCCCTTTCTCGTCTTTGCGTATCAACGTATCAGCTTTACGGGCTGGGACAGTATCATACTGTCATTGGCGCATTTGTTCACTTCTCAGCTGCATCATGCCATTCCCACTTTATTTAATGTGCACGCCTACTCCTTTATGCTTTTTTCGGTTTTATTGGTCCGGCTTTGCCATGGATCAGAATCTTATCTAGGCAATAGCTTTTATTGCCTTGCTGGGTTCTCATCCCTACACGTTCTGTCAAAGCTAGATCTTTATTGTTCTTGAGCTCTACTCAGTTCGTGACACTTGGTAGTGTCACACTCGAATCTTTGCCGTTACTCGCACTCAGCTTTTGATAGTTTTTCTGTTATGGTTATGGCAAATAGTTCTAGCAAACTGTGATATACTAGTACTCAAGTGTGTATCTT

>*TaCOI7-D*

AGTGCGGCTGCCGCCTGAAGTTCGACCTCCACTATCCCGTCATCGACCGCGATGGATGTGCTCGAATCACGCCGCCGCCGACCTTGATGGTTGAGCTCCTCCTATCTCCCGCCGCAGACCGAGGATTGACCTCCTATGACGCCGCCACCGCCCATCGACCAAGGATTGACCTCTTGTCCGGTAGCCGCTGCCTAGGCTGCCGCTGCCATATCTATATGCAGAGCGGCTATTTTTTTACGGATATCCGCAGAGCGGCTGTTTACACGGTATCACTGTAACTCAATTATATGTAAAGGGTAGTACATTACATACGGTTATGTAACGGCAACCGTGTCTCACCTACGTTATCTTCTCACACGGTTTGGTGTGGAAATCGTGTGTGACATTGTAATACCTGACACAAACAACATGTATAAACAATGTGCCGTATACAACATATAGTGCTATAAGTAGTTCCCGACCGTTAGCTTATCCTCACAGTTTCCCCATTTCCCCCAAATCCCTACATAGCCTCCAAATTCCCTCGCGTGGCGCTGACAGTGGGGGAATGCGGACGAGTGGTGCTGATGGTTTCGGTGCGGCTGCTCCTGCTCGTATCGCCGCCACGACGGCAGTCGTCGCCGAGCACTTGCCTAAGGTCATCCGAAGGCAGGTGTACTACGGATCCGAATCATCCTGTCAGGTTCCAATCTATCCCGATCTTTTTTAGCATGTCCAAAGTAGCTCCTTGCTGAATCCGTCCTGAATGAACCCCCCTAGGAAAGCAATCTGCCACCGGATCCGGAGCATGACTCCAGACTCCCGCAACACGAGTTCGACTCCGAGTTTTGCGCCACCGATAAGTACCAAAAGCTGAGTTGTGTGCACGGGCACACTCCCGCTCGATGTGTATGCACGGAGGGATTCGACATTGGCAGGCGGTTTCTCAGTTGCACCTTAGAGGTTAGTGCTAAGTTCATAATTTTACTTGAGTTAATGCCACCGCTCATCCTGAATACTATTTAACATTGGCAGGGATATGAGGCTTGTGCCTTTGTTAATTGGCTGGATGAAGAATGGCATGGCAGGGCTCAGAGTGTTATCACCAAGCTCGCAGAAGATAACATAAAGCTGAAGAAGAAACTGGCTGATCTGGAATGTACAATTAGTACGATGAAGCAAGAAAGAATCAATCACAGGCAATAGATGAAAGCAAGAGACAAGAAGGAACTAGCATGTGTAGTTGTGGTTGTCGCATTAGCCATGTTCTATGCGCTGTTTGCAATGATGATTAGGGGTTTTGTTTGACAGTATTGCTAAAGCACATCTAGATGTGCTCTAAGTAATGCACATCTAGGTCCTATNNNNNNNNNNNNNNNNNNNNNNNNNNNNNNNNNNNNNNNNNNNNNNNNNNNNNNNNNNNNNNNNNNNNNNNNNNNNNNNNNNNNNNNNNNNNNNNNNNNNNGTCCGGCCTTGCCCTGGATCAGAATCTGATCTAGGCCATAGCTTTTTATTGCCGTGCTGGGTTCTCATCCCTACACGTTCTGTCAAAGCTAGATCTTTATTGTTCTTGAGCTCTACTCAGTTCGTGACACTTGGTAGTGCCACACTCGAATCTTTGCCGTTAGTGGTACTCAACTTTTGATCGTTTTCTTGTTATGTTATGGCAAATAGGTCTAGCAAACAAATGTCTCAAACTATGATATAGTAGTACTCAGGTTGTGTTATCTTATGGATCGTGACATTTATGTTTTAGGCATCTTCGAGTCCTGGATCTGACTGAAGATTATTTCCATGAGGAGGAGAGCGAGGTGGTGGATTGGATCTCCAAGTTTCCAGAGTGCAGCACGTCGCTGGAATCTCTTGTCTTTGATTGTGTTAGTGTCCCATTCAACTTTGAGGCCCTGGAGGCACTCGTTGCACGCTCACCAGCTCTCCGTCGTCTGCGCGTGAATGACCATGTCTCGATAGAGCAGCTGCGTCGTCTC

>*TaCOI8-A*

TTGTTCTTTGACAAAACAATATGCGCTAGTAAAATAAACTTAAATGGACATCACCTGATTTATTTGGACCATAGACGGTCCATACACAGATCCAAAATCATGCACAAAGGCACCATCAATACAGATGTGTTATTTACTTGATTCTCCGTGAAGTATTATCAGCTCCAATTGATTGACTCCTCCACTGCGTGATCTCCCTTTTATTCATATGTAAATTAATAGTAGCTTCTAGGTACACATAATGTGTATTATAATGCCAAAGCATGCCAGAACTTTTTGTCATTTTTTGCCTGACTGTGGTCATTACATGGGCAGTAACCACTTTTTCCACCACGGCCTCAGAACCGGAAAAATATGCAAGCGTTTGAAACTATTCNNNNNNNNNNNNNNNNNNNNNNNNNNNNNNNNNNNNNNNNNNNNNNNNNNNNNNNNNNNNNNNNNNNNNNNNNNNNNNNNNNNNNNNNNNNNNNNNNNNNNTGGGCCACGTTGGCCTTTACGAGCACAGGCTGCTAGCGCTCATGGGCTTTTGTTCGCCGATAATTCTGATGATGGCGATGAGATAATTGATGGTGCTAATCCAGGACCATGCCTCGGCCTCCCTCGGTGATAGGACAGCCTCGTCGTTTCAATGCATTTGCGGGGCCCATATTTAGTGTCACTACTATAGAGTACTCCCTCCGTAAACTAATATAAGAGTGTTTAGATCACTACTTTAGTTATCTAAACGCTCTTATATTAGTTTACAGAGGGAGTACTAACTATTCTCCCACCAGACCACTAATCATAGACCGATGATAGATCTCGGGGAACATGGAGGCTGGAGAAGTAACGGGGTGTTTGTTTTCATGGATTTTTTTGTGTAGGGACTAGCAAAAGTCTTTTTTAGAGACTTTTTTACTAAATAGGAGGGATTTTTTAGGACTAAATTAGGTATTTGGGACTAAATGAAGAAGACTCTTAAAGAGAGTTTTTTTGGAACTTTTTGAGACTTTTCGGTGGTGTTTGGTTCTCTGGTCCTAGGACTTTTTCTAGTCCAACTAAAAAGTCCCTAGTCCCTAAAAAGTTCCTCCAGTTTGTTTCCAGAGACTAAAAAGTCCCTAGTCCCTTCNNNNNNNNNNNNNNNNNNNNNNNNNNNNNNNNNNNNNNNNNNNNNNNNNNNNNNNNNNNNNNNNNNNNNNNNNNNNNNNNNNNNNNNNNNNNNNNNNNNNNAGGCACAAAAAAGCCATTTGAAAGATTCAAATGTCTCATCACGGAGAAGTGAAGCACCAAATACTAATGTTTCTCTGAAATGGTTGAATCCAATAGACACACCAAATGGCATGTACTCTTTATTTGTCCCAAAAGTAGTGTCAAAGGTCATAACATAACCAAAATGAGCATAATCTATGATCATTTTAGCATCAGCCTAGTCATTTGTTCCTCGCTGTCCGAATGTATAACAAATTGAAAGGAAGGATTCTCAACGGCTTTGTCTTCAAAATACTTCAACATACTTCCACCCTCACCATACTTCAATTCTCTTTGGCGCTTTGTTCGCAAGTGGTTCTTGTGATCTACGCGTGTGTAAGTTAGACCAGCTGTTACACCAAAATGTCCACTAGCAAATTCATTAGCATTTTCTGGTGCAATACCTGAGTCATCAACAATTTCAAAAGCTTGCACTTTAGAGATCCTTCTTTGTGATGGCNNNNNNNNNNNNNNNNNNNNNNNNNNNNNNNNNNNNNNNNNNNNNNNNNNNNNNNNNNNNNNNNNNNNNNNNNNNNNNNNNNNNNNNNNNNNNNNNNNNNNCTTGGCTTCAGTTTATAAAGGAGAGGAGTCGAGTGTGGCATCTAATCGACTGATGTACTTAATACTTGTGTAGGCACCTGAGGGTTCTGGATCTGATCGAGGACTATGTGGATCAGGAGGACGAAGCAGTGGACTGGATCTCCAAGTTCCCAGTGTCAAACACATCCCTAGAATCCCTC

Table S6 The protein sequences, CDS sequences and genomic sequences of *TaCOI* genes and *COI* or *COI*-like genes in different plants

Protein sequences:

>TaCOI1-A Traes_1AS_1E22B5174.1

MEDLQEALMTEILKRITTISDLNSLSLVSKQLCKIEGNQRGAIRVGSRLCTATEALTSLCARFPNLRRVEIDYFGWIPGHGRQLDNKGFSVFSSHYSSLIDLTLSFCSCIDDSGLACLAYCKTLVSLRLNSAPKITSVGLFSVAVGCRSLSALHLIDCEKIDTVEWLEYLGRDGSLEELVVKNCQGINHHDFLKFGSGWMKLQKFEFESKRERYDRLPGDVVYDSSYHAHSTDIYDFCCESLKDLRLAHIKTWPEVGLRLVLGKCKALEKLCLEYVRALNDNDIIALSRSCSNLKSISLWLNLQRYSSDVSYCETRTSFTDNSLYALARNCRMLQMVDLSFIGCSRDWPSEIGFTQEGFLVLIQSCPIRVLVLNTANFFDDEGMKALSSSPHLETLELILCHAVTDAGMHFIAHTPCLSNLTLRLCHNVTDVGVAELGHAHKLESLVVEYCGEVSLQAVQGVAKLVHYSDYSYFFMKKIGLGAY

>TaCOI2-A Traes_2AL_9EC359B65.2

MTYFPEEVVEHIFSFLPAQCDRNTVSLVCKVWYEIERLSRRTVFVGNCYAVRPERVVLRFNVRALTVKGKPHFADFNLVPPDWGGYAGPWIEAAARGCVGLEELRMKRMVVSDESLELLAKSFPRFRALVLISCEGFSTDGLAAIASHCKLLRELDLQENEVDDRGPRWLSCFPDSCTSLVSLNFACIKGEVNAGSLERLVARSPSLRSLRLNRSVSVDTLSKILMSAPNLEDLGTGNLTDDFQAESYLRLTLALEKCKLLRSLSGFWDASPLCLPFIYPVCGQLTGLNLSYAPTLDSSDLTKMISHCVKLQRLWVLDCIADKGLQVVASSCKDLQELRVFPSDFYIAGYSPVTEEGLVAISLGCQKLSSLLYFCHQMTNAALLTIAKNCPNFTRFRLCILEPGKPDAMTNQPLDEGFGAIVRECKGLRRLSISGLLTDKVFMYIGKFAKQLEMLSIAFAGDSDAGMMHVMEGCNNLRKLEIRDSPFGDAALLENVTKYETMRSLWMSSCNVTEKGCQILASKMPMLNVEVINEVDESNEMDENHGIPKVDKLYVYRTTAGARDDAPNFVKIL

>TaCOI2-B Traes_2BL_370AE211F.1

MTYFPEEVVEHIFSFLPAQCDRNTVSLVCKVWYEIERLSRRTVFVGNCYAVRPERVVLRFPNVRALTVKGKPHFADFNLVPPDWGGYAGPWIEAAARRCVGLEELRMKRMVVSDESLELLAKSFPRFRALVLISCEGFSTDGLAAIASHCKLLRELDLQENEVDDRGPRWLSCFPDSCTSLVSLNFACIKGEVNAGSLERLVARSPSLRSLRLNRSVSVDTLSKILMRAPNLEDLGTGNLTDDFQAESYLRLTLALEKCKLLRSLSGFWDASPLCLPFIYPVCGQLTGLNLSYAPTLDSSDLTKMISHCVKLQRLWVLDCIADKGLQVVASSCKDLQELRVFPSDFYIAGYSPVTEEGLVAISLGCQKLSSLLYFCHQMTNAALLTIAKNRPNFTRFRLCILEPGKPDAMTNQPLDEGFGAIVRECKGLRRLSISGLLTDKVFMYIGKFAKQLEMLSIAFAGDSDAGMMHVMEGCNNLRKLEIRDSPFGDAALLENVTKYETMRSLWMSSCNVTEKGCQILASKMPMLNVEVINEVDESNEMDENHGIPKVDKLYVYRTTAGARDDAPNFVKIL

>TaCOI3-B TRAES3BF018800040CFD_t1

MGGEVPEPRRLSRALSFGVPDEALHLVMGYVDAPRDREAASLVCRRWHRIDALTRKHVTVAFCYAADPSRLLARFPRLESLALKGRPRAAMYGLISDDWGAYAAPWVARLAAPLECLKALHLRRMTVTDDDVATLIRSRGHMLQELKLDKCSGFSTDALRLVARSCRSLRTLFLEECVITDEGGEWLHELAVNNSVLVTLNFYMTELKVVPADLELLAKNCKSLLSLKISECDLSDLIGFFEAANALQDFAGGSFNEVGELTKYEKVKFPPRVCFLGLTFMGKNEMPVIFPFSASLKKLDLQYTFLSTEDHCQLISKCPNLFVLEVRNVIGDRGLEVVGDTCKKLRRLRIERGDDDPGLQEEQGGVSQLGLTAVAVGCRDLEYIAAYVSDITNGALESIGTFCKNLYDFRLVLLDRQKQVTDLPLDNGVRALLRSCTKLRRFALYLRPGGLSDIGLDYIGQYSGNIQYMLLGNVGESDHGLIRFAIGCINLRKLELRSCCFSEQALSLAVLHMPSLRYIWVQGYKASPAGLELLLMARRFWNIEFTPPSPEGLFRMTLEGEPCVDKQAQVLAYYSLAGQRQDCPDWVTPLHPAA

>TaCOI3-D Traes_3DL_845220DFC.1

MTELKVVPADLELLAKNCKSLLSLKISECDLSDLIGFFEAANALQDFAGGSFNEVGELTKYEKVKFPPRICFLGLTFMGKNEMPVIFPFSASLKKLDLQYTFLTTEDHCQLISKCPNLFVLEVRNVIGDRGLEVVGDTCKKLRRLRIERGDDDPGLQEEQGGVSQLGLTAVAVGCRDLEYIAAY

>TaCOI4-A Traes_3AL_2240B0C5F.1

MNELVISLAHKFTKLQVLSLRQIKPQLEDSAVEAVANSCHDLRELDLSRSFRLSDRSLYALAHGCPHLTRLNISGCSNFSDAALIYLTSQCKNLKCLNLCGCVRAASDRALQAIARNCSQLQSLNLGWCDTVTDGGVTSLASGCPELRAVDLCGCVLITDESVVALANGCPHLRSLGLYYCQNITDRAMYS

>TaCOI4-B TRAES3BF021600080CFD_t1

MVNAQMVSGYLDNSFNALVVSGGGESGGQTQNGVTDTTLSGWKDLPMELLLRIISVAGDDRMAIVASGVCTGWRDALGWGATSLSFSWCQDHMNELVISLAHKFTKLQVLSLRQIKPQLEDSAVEAVANSCHDLRELDLSRSFRLSDRSLYALAHGCPRLTRLNISGCSNFSDAALIYLTSQCKNLKCLNLCGCVRAASDRALQAIACNCSQLQSLNLGWCDTVTDGGVTSLASGCPELRAVDLCGCVLITDESVVALANGCPHLRSLGLYYCQNITDRAMYSLAENSRIRSKGMSWDTAKNSRGRDDKDGLASLNISQCTALTPPAVQAVCDSFPALHTCPERHSLIISGCLSLTTVHCACAHHRHRAGAGRAILSNHAY

>TaCOI4-D Traes_3DL_3DB9F1EC5.1

MVNAQMLSGYLDNSFNALMVSGGGESGQPQNGGTDTTLSGWKDLPMELLLRIISVAGDDRXIVASGVCTGWRDALGWGATSLSFSWCQDHMNELVISLAHKFTKLQVLSLRQIKPQLEDTAVEAVANSCHDLRELDLSRSFRLSDRSLYALAHGCPHLTRLNISGCSNFSDAALIYLTSQCKNLKCLNLCGCVRAASDRALQAIACNCSQLQSLNLGWCDTVTDGGVTSLASGCPELRAVDLCGCVLITGATFILLQDQVCSPKFISVGNDLHHIRHCYLSSAS

>TaCOI5-A Traes_4AS_AD3F2B991.1

MSFGIPDVALGLVMGCVEDPWDRDAISLVCRHWCKVDALSRKHVTVAMAYSTTPDRLFRRFPCLESLKLKAKPRASMFNLIPEDWGGSASPWIRELSASFHFLKVLHLRRMIVSDDDVAVLVRAKAHMLVSLKLDRCSGFSTSSLALLARCCKKLETLFLEESSVAEKENDEWLRELATSNTVLETLNFFLTDLRASPAHLLLLVRNCRRLKTLKISDCFMSDLVDLFRTAETLQDFAGGSFDDQDQGGNYANYYFPPSVQHLSLLYMGTNEMQILFPYGATLKKLDLQFTFLTTEDHCQLVQRCPNLEVLEVRDVIGDRGLEVIARTCKKLQRLRVERGDDDQGGLEDEQGRVTQVGLMAVAEGCPDLEYWAVHVSDITNAALEAIGAFSKNLNDFRLVLLDREVHITELPLDNGVRALLRGCTKLRRFAFYVRPGALSDIGLSYVGEFSKTVRYMLLGNAGGSDDGLLAFARGCPSLQKLELRSCCFSERALAVAALQLKSLRYLWVQGYKASPTGTDLMAMVRPFWNIEFIAPNQDEPCPEGQAQILAYYSLAGARTDCPQSVIPLHPSVGS

>TaCOI5-B Traes_4BL_499E7F095.1

MGGDERHLGRTMSFGIPDVALGLVMGCVEDPWDRDAISLVCRHWCKVDALSRKHVTVAMAYSTTPDRLFRRFPCLESLKLKAKPRASMFNLIPEDWGGSASPWIRELSASFHFLKVLHLRRMIVSDDDVAVLVRAKAHMLVSLKLDRCSGFSTSSLALLARCCKKLETLFLEESSVAEKENDEWLRELATSNTVLETLNFFLTDLRASPAYLLLLVRNCRRLRTLKISDCFMSDLVDLFRTAETLQDFAGGSFDDQDQGGNYANYYFPPSVQRLSLLYMGTNEMQILFPYGATLKKLDLQFTFLTTEDHCQLVQRCPNLEVLEVRDVIGDRGLEVVAQTCKKLQRLRVERGDDDQGGLEDEQGRVTQVGLMAVTQGCPDLEYWAVHVSDITNAALEAIGTFSKNLNDFRLVLLDREVHITELPLDNGVRALMRGCTKLRRFAFYVRPGALSDIGLSYVGEFSKTVRYMLLGNAGGSDDGLLAFARGCPSLQKLELRSCCFSERALAVAALQLKSLRYLWVQGYKASPTGTDLMAMVRPFWNIEFIAPNQDEPCPEGQAQILAYYSLAGARTDCPQSVIPLYPSVGG

>TaCOI5-D Traes_4DL_8C2C2ADD4.1

MGGDERHLGRTMSFGIPDVALGLVMGCVEDPWDRDAISLVCRHWCKVDALSRKHVTVAMAYSTTPDRLFRRFPCLESLKLKAKPRASMFNLIPEDWGGSASPWIRELSASFHFLKVLHLRRMIVSDDDVAVLVRAKAHMLVSLKLDRCSGFSTSSLALLARCCKKLETLFLEESSVAEKENDEWLRELATSNTVLETLNFFLTDLRASPAYLFLLVRNCRRLKTLKISDCFMSDLVDLFRTAETLQDFAGGSFDDQDQGGNYANYYFPPSVQRLSLLYMGTNEMQILFPYGATLKKLDLQFTFLTTEDHCQLVQRCPNLEVLEVRDVIGDRGLEVVARTCKKLQRLRVERGDDDQGGLEDEQGRVTQVGLMAVAEGCPDLEYWAVHVSDITNAALEAIGAFSKNLNDFRLVLLDREVHITELPLDNGVRALLRGCTKLRRFAFYVRPGALSDIGLSYVGEFGKTVRYMLLGNAGGSDDGLLAFARGCPSLQKLELRSCCFSERALAVAALQLKSLRYLWVQGYKASPTGTDLMAMVRPFWNIEFIAPNQDEPCPEGQAQILAYYSLAGARTDCPHSVIPLYPSVGS

>TaCOI6-A Traes_5AL_27F194099.2

MPYFPDEVVEHILGFISSHRDRDAASLVCHAWYRIEGLTRRSVFISNCYAVRPERVHARFPCLRSLTVKGKPRFADFNLVPAGWGASAEPWVDACARACPGLEELRLKRMVVTDGCLNHLAHSFPNLRSLVLVSCEGFSTDGLATIATNCRFLKELDLQGSQVEFRGRHWFSCFPKPSTSLESLNFACLDGAVSANALESLVARSPNLKSLRLNRAVPPAVLAKILTSAPKLVDLGTGLVAQSNNAGALPSLYSAIQQCSSLNSLSGFWDSPRWITPIIQYICKNLTCLNLSYAPMFRTVDLIGIIRQCQNLRHLWVLDHIGDAGLKVVASSCLELQELRVFPANANVLISTGVTEEGLVAVSSGCRKLNSVLYSCRRMTNSALITVAKNCSRITSFRLHICLHGSVDAVTGQPLDEGFGAIVRSCKGLRRLSMSGLLTDSVFLYIGMYAERLETLSVSFAGDSDDGMIYVLNGCKNLRKLEIRNCPFGNTALLAGMHRYEAMRSLWMSSCDITLGGCRSLAAAMPGLNVEVISQADGGANDAKKVEKLYVYRTLAGPRVDAPGFVSAL

>TaCOI6-B Traes_5BL_7AE04C6B2.1

MPYFPDEVVEHILGFVSSHRDRDAASLVCHAWYRIEGLTRRSVFISNCYAVRPERVHARFPCLRSLTVKGKPRFADFNLVPAGWGASAEPWVDACARACPGLEELRLKRMVVTDGCLNHLAHSFPNLRSLVLVSCEGFSTDGLATIATNCRFLKELDLQGSQVEFRGRHWLSCFPKPSTSLESLNFACLDGAVSANALESLVARSPNLKSLRLNRAVPPAVLAKILTSAPKLVDLGTGLVAQSNNAGALPSLYSAIQQCTSLNSLSGFWDSSRWITPIIHCICKNLTCLNLSYAPMFRTVDLIGTIHQCQNLRHLWVLDHIGDAGLKVVASSCLELQELRIFPANPANANVLASTGVTEEGLVAVSSGCRKLNSVLYSCSRMTNSALITVAKNCSRITSFRLHICLHGSVDAVTGQPLDEGFGAIVRSCKGLRRLSMSGLLTDSVFLYIGMYAERLETLSVSFAGDSDDGMIYVLNGCKNLRKLEIRNCPFGNTALLAGTHRYEAMRSLWMSSCDITLGGCRSLAAAMPGLNVEVICQADGGANDAKKVDKLYVYRTLAGPRDDAPGFVSAL

>TaCOI6-D Traes_5DL_4E8DD7C5F.3

MPYFPDEVVEHILGFVSSHRDRDAASLVCHAWYRIEGLTRRSVFISNCYAVRPERVHTRFPCLRSLTVKGKPRFADFNLVPAGWGASAEPWVDACARACPGLEELRLKRMVITDGCLNHLAHSFPNLRSLVLVSCEGFSTDGLATIATNCRFLKELDLQGSQVEFRGRHWLSCFPKPLTSLESLNFACLDGAVSANALESLVARSPNLKSLRLNRAVPPAVLAKILTSAPKLVDLGTGLVAQSNNAGALPSLYSAIQQCTSLNSLSGFWDSQRWITPIIHYICKNLTYLNLSYAPMFRTVDLIGTIHQCQNLRHLWVLDHIGDAGLKVVASSCLELQELRIFPANANVLASTGVTEEGLVAVSSGCRKLNSVLYSCSRMTNSALITVAKNCSRITSFRLHICLHGSVDAVTGQPLDEGFGAIVRSCKGLRRLSMSGLLTDSVFLYIGMYAERLETLSVAFAGDSDDGMIYVLNGCKNLRKLEIRNCPFGNTALLAGTHRYEAMRSLWMSSCDITLGGCRSLAAAMPGLNVEVISQADGGANDAKKVEKLYVYRTLAGPRDDAPGFVSAL

>TaCOI7-A Traes_6AL_0F53490CA.1

MARAPHLTHLGTGSFRSEPGPGGALSVSELATSFAASRSLVCLSGFLDANAAYLPAIYQVCANLTSLNFSFAGLTDEEFIPVIRHCINLRTLWVLDTVGDEGLRAVAETCSNLRELRVFPLDATEDSEGSVSDIGLQAISEGCRKLESILYFCQRMTNAAVIAMSENCPDLLVFRLCIMGRHRPDRITGAPMDEGFGAIVMNCKKLTRLSVSGLLTDKAFAYIGRHGKLIKTLSVAFAGNSDMSLQHVFEGCTRLQKLEVRDSPFGDKGLLSGLNYFYNMRFFWMNSCRLTVKGCGDVAQQMPNLVVEVMKENEGEMDTVDKLYLYRSLAGPREDAPSFVNIL

>TaCOI7-B Traes_6BL_9E82CDBD1.1

MDRCIYFFRHLRVLDLTEDYFHEEESEVVDWISKFPECNTSLESLVFDCVSVPFNFEALEALVARSPALRRLRVNDHVSIEQLRRLMARAPHLTHLGTGSFRSEPGPGGALSVSELATSFAASRSLVCLSGFLDANAAYLPAIYQVCANLTSLNFSFAGLTAEEFIPVIRHCVNLRTLWVLDTVGDEGLRAVAETCSNLRELRVFPLDATEDSEGSVSDIGLQAISEGCRMLESILYFCQRMTNAAVIAMSENCPDLLVFRLCIMGRHRPDRITGAPMDEGFGAIVMNCKKLTRLSVSGLLTDKAFAYIGRHGKLIKTLSVAFAGNSDMSLQHVFEGCTRLQKLEVRDSPFGDKGLLSGLNYFYNMRFFWMNSCRLTVKGCGDVAQQMPNLVVEVMKENEGEMDTVDKLYLYRSLAGPREDAPSFVNIL

>TaCOI7-D Traes_6DL_AD0DAD6D1.2

MARAPHLTHLGTGSFRSEPGPGGALSVSELATSFAASRSLVCLSGFLDANAAYLPAIYQVCANLTSLNFSFAGLTDEEFIPVIRHCVNLRTLWVLDTVGDEGLRAVAETCSNLRELRVFPLDATEDSEGSVSDIGLQAISEGCRKLESILYFCQRMTNAAVIAMSENCPDLLVFRLCIMGRHRPDRITGAPMDEGFGAIVMNCKRLTRLSVSGLLTDKAFAYIGGHGKLIKTLSVAFAGNSDMSLQHVFEGCTRLQKLEVRDSPFGDKGLLSGLNYFYNMRFFWMNSCRLTVKGCGDVAQQMPNLVVEVMKENEGEMDTVDKLYLYRSLAGPREDAPSFVNIL

>TaCOI8-A Traes_7AS_78FEAE00C.1

MFDCVAVPFNFEALEALVARSPSLRRLRVNHYVSVEQLRRLMARAPQLTHLGTGAFRPEAPQGGGMSVSELAPSFAASTSIVCLSGFQEVNPEYLPAIYPVCGNLTSLNVSFASLTAEDLTPVIRQCHKLQTFWVLDTAGDECLRAVAETCSDLRELRVFPLDATEDSDGSVSDVGLQAISEGCRKLESILYFCQRMTNAAVVAMSNNCPDLVVFRLCIMGRHRPDRITGEPMDDGFGAIVKNCKKLTRLSVSGLLTDKAFAYIGQYGKLIKTLSLAFSGNSDLSLQFLFEGCTRLQKLEVRDSPFSDRGLLCGLDYFYNMRFLWMNSCRLTMRGCREVAQRMPNLVVEVMEEQNEDKVETETVDKLYLYRSLAGPRGDAPPLVKIL

>AetCOI1 EMT18237

MGCVEDPWDRDAISLVCRHWCKVDALSRKHVTVAMAYSTTPDRLFRRFPCLESLKLKAKPRASMFNLIPEDWGGSASPWIRELSASFHFLKVLHLRRMIVSDDDVAVLVRAKAHMLVSLKLDRCSGFSTSSLALLARCCKKLETLFLEESSVAEKENDEWLRELATSNTVLETLNFFLTDLRASPAYLFLLVRNCRRLKTLKISDCFMSDLVDLFRTAETLQDFAGGSFDDQDQGGNYANYYFPPSVQRLSLLYMGTNEMQILFPYGATLKKLDLQFTFLTTEDHCQLVQRCPNLEVLEVRDVIGDRGLEVVARTCKKLQRLRVERGDDDQGGLEDEQGRVTQVGLMAVAEGCPDLEYWAVHVSDITNAALEAIGAFSKNLNDFRLVLLDREVHITELPLDNGVRALLRGCTKLRRFAFYVRPGALSDIGLSYVGEFGKTVRYMLLGNAGGSDDGLLAFARGCPSLQKLELRSCCFSERALAVAALQLKSLRYLWVQGYKASPTGTDLMAMVRPFWNIEFIAPNQDEPCPEGQAQILAYYSLAGARTDCPHSVIPLYPSVGS

>AetCOI2 EMT11181

MAMTAVSGRWSLAVIDKNISEFDINARHPSPAPQQQAPKKRMTDHLLTRPRRGSIADMQLCGPPRRLTKSEALAVERIRPGQHPGHVSELQIWHPTTTKTPKRETIPVIHATRTQRTFRLPDIVDADYNLHPLLDCNQFYYSLFLLRAQTRRELFIGNCYAVDPRRAVARFRAVAAVVLKGKPRFADFSLLPDGWGANVKPWLEALGPAYPCLERICLKRMTVTDDDLGLVARSFPGFQELSLVCCDGFSTLGLAVIAEGCRHLRVLDLIEDYVDQEDEAVDWISKFPVSNTSLESLMFDCVGAPFNFEALEALVARSPSLRRLRVNHHVSVEQLRRLMARAPQLTHLGTGAFRPEAPQGEGMSVSELAPSFAASTSIVCLSGFQEVNPEYLPAIYPVCGNLTSLNVSFASLTAEDLTPVIRQCHKLQTFWVGPYSYLFAMVLDTVGDEGLRAVAETCSDLRELRVFPLDATEDSDGSVSDVGLQAISEGCRKLESILYFCQRMTNAAVVAMSNNCPDLVVFRLCIMGRHRPDRITGEPMDDGFGAIVKNCKKLTRLSVSGLLTDKAFAYIGQYGKLIKTLSLAFSGNSDLSLQFLFEGCTRLQKLEVRDSPFSDRGLLCGLDYFYNMRFLWMNSCRLTMRGCREVAQRMPNLVVEVMEEQNEDKVETETVDKLYLYRSLAGPRGDAPPLVKIL

>AetCOI3 EMT22616

MCYKNFVSAAAAEDAAAEADYTSDLPEELLAVVFGLLGSGDRKRCSLVCRRWLATEASSRLRLALDARAPLLAAAPAILARFSAVSKLALKCDRRAESVGDPALALVAHRLGPGLRRLKLRSVRAVTDHGVAALAAAAINLRKLSVGSCTFGAKGIEAVLRSCPQLEELSVKRLRGLADSEPITVSSPRLQSLALKELYNGQCFSCLITRSPNLKTLKIIRCSGDWDPVLQAIPQGALLAELHLEKLQVSDLGVAALCGLEVLYLAKAPEVTDVGLAALATKSPRLRKLHVDGWKANRIGDRGLATVAQKCAALQELVLIGVNLTSASLELIAANCPTLERLALCGSDTFGDAEISCVATKCASLRKLCIKACPVSDAGMDKLAAGCPRLVKVKVKKCRRVTFECAERLRASRHGALAVNFDTPGGAGELQDASVDESGVLENAGSDVWQTIRVESMDEYFDSKELVGFHVSQTSAWELP

>AetCOI4 EMT31633

MGGRAAVPGTFWSSSLVSGATSTSAEGRRYSDREAAALAAASGLRELNLEKCLGVTDMGLAKVAVGCPRLENLSFKWCREISDIGVDLLVKKCRELRSLDISYLKIRLDSFYGVHSLYLGAIYNCILVNNNKAFCPKQFGVSNESLRSISTLEKLEELAMVACSCIDDEGLELLSRGSNSLQGYSCILSCLPSPEIIWLTLSSNMSVDVSRCDHVTSQGLASLIDGHSFLQKLYAADSLHEIGQDFLSKLVTLKATLAVLRLDGFEVSSSLLSAIGEGCTNLVEIGLSKCNGVTDEGISSLVACCSYLRTIDLTCCNLVTNNSLDSIADNCKLLECLRLESCSSINEKGLERIASCCPNLKEIDLTDCGVNDEGIGISSVVTGCKSLVELDLKRCYSVDDSGLWALARYALNLRQLTISYCQVTGLGLCHLLSSLRCLQDVKMVHLSWVSIEGFEMALRAACGRLKKLKILSGLKTVLSPDLLQLLQACGCRIRWVNKPLVYKDAI

>AetCOI5 EMT03750

MEDLPEPLLAEIVKRITKTRDLNSLSLVSKQLCTVEAEHRDAIRVGCGLNPSTEALVSLFSRFPNLAKVEINYSGWWPSNGNQLNNQGLCVLSSHCPSLSDLSLSFCSYIGDSGLGYLADCKKLRSLRLNCAPAVTSTGIFRVAVGCRHLSVLHLVDCMAVDSREWLEYLGRYGSLVELVVKDCDGISQYDLLKFGPGWENLQKFVFEINGNYWMSGASDPSYVSGYPYRYDICCDSLKDLRLAHIITEPETGLRFLLGKCKALETLYLEYVIGLKENETIALFQRCSNLKTISLRFMPLRCEDYEFRTPLTDVSLKALALSCPMLQVVELTFTFCEPMYPTEIGFTQEGIVSLVQSCPVRTLLLNGASILYDEGLKCLSSSQFLEKLELVDCRSITDAGMSFIILAPCFSNLTLRKCKKVTDNGMAELARSQKLESLTVIGCCQISQEGVQGAAKSVHYSAETESHGSLKGMNTDRDMNRKRRRSP

>AtCOI1 AT3G62980.1

MQKRIALSFPEEVLEHVFSFIQLDKDRNSVSLVCKSWYEIERWCRRKVFIGNCYAVSPATVIRRFPKVRSVELKGKPHFADFNLVPDGWGGYVYPWIEAMSSSYTWLEEIRLKRMVVTDDCLELIAKSFKNFKVLVLSSCEGFSTDGLAAIAATCRNLKELDLRESDVDDVSGHWLSHFPDTYTSLVSLNISCLASEVSFSALERLVTRCPNLKSLKLNRAVPLEKLATLLQRAPQLEELGTGGYTAEVRPDVYSGLSVALSGCKELRCLSGFWDAVPAYLPAVYSVCSRLTTLNLSYATVQSYDLVKLLCQCPKLQRLWVLDYIEDAGLEVLASTCKDLRELRVFPSEPFVMEPNVALTEQGLVSVSMGCPKLESVLYFCRQMTNAALITIARNRPNMTRFRLCIIEPKAPDYLTLEPLDIGFGAIVEHCKDLRRLSLSGLLTDKVFEYIGTYAKKMEMLSVAFAGDSDLGMHHVLSGCDSLRKLEIRDCPFGDKALLANASKLETMRSLWMSSCSVSFGACKLLGQKMPKLNVEVIDERGAPDSRPESCPVERVFIYRTVAGPRFDMPGFVWNMDQDSTMRFSRQIITTNGL

>AtCOI2 AT1G12820.1

MNYFPDEVIEHVFDFVASHKDRNSISLVCKSWHKIERFSRKEVFIGNCYAINPERLIRRFPCLKSLTLKGKPHFADFNLVPHEWGGFVHPWIEALARSRVGLEELRLKRMVVTDESLDLLSRSFANFKSLVLVSCEGFTTDGLASIAANCRHLRELDLQENEIDDHRGQWLNCFPDSCTTLMSLNFACLKGETNVAALERLVARSPNLKSLKLNRAVPLDALARLMSCAPQLVDLGVGSYENEPDPESFAKLMTAIKKYTSLRSLSGFLEVAPLCLPAFYPICQNLISLNLSYAAEIQGNHLIKLIQLCKRLQRLWILDSIGDKGLAVVAATCKELQELRVFPSDVHGEEDNNASVTEVGLVAISAGCPKLHSILYFCKQMTNAALIAVAKNCPNFIRFRLCILEPHKPDHITFQSLDEGFGAIVQACKGLRRLSVSGLLTDQVFLYIGMYAEQLEMLSIAFAGDTDKGMLYVLNGCKKMRKLEIRDSPFGNAALLADVGRYETMRSLWMSSCEVTLGGCKRLAQNSPRLNVEIINENENNGMEQNEEDEREKVDKLYLYRTVVGTRKDAPPYVRIL

>AtCOI3 AT5G49980.1

MTQDRSEMSEDDDDQQSPPLDLPSTAIADPCSSSSSPNKSRNCISNSQTFPDHVLENVLENVLQFLDSRCDRNAASLVCKSWWRVEALTRSEVFIGNCYALSPARLTQRFKRVRSLVLKGKPRFADFNLMPPDWGANFAPWVSTMAQAYPCLEKVDLKRMFVTDDDLALLADSFPGFKELILVCCEGFGTSGISIVANKCRKLKVLDLIESEVTDDEVDWISCFPEDVTCLESLAFDCVEAPINFKALEGLVARSPFLKKLRLNRFVSLVELHRLLLGAPQLTSLGTGSFSHDEEPQSEQEPDYAAAFRACKSVVCLSGFRELMPEYLPAIFPVCANLTSLNFSYANISPDMFKPIILNCHKLQVFWALDSICDEGLQAVAATCKELRELRIFPFDPREDSEGPVSELGLQAISEGCRKLESILYFCQRMTNAAVIAMSENCPELTVFRLCIMGRHRPDHVTGKPMDEGFGAIVKNCKKLTRLAVSGLLTDQAFRYMGEYGKLVRTLSVAFAGDSDMALRHVLEGCPRLQKLEIRDSPFGDVALRSGMHRYYNMRFVWMSACSLSKGCCKDIARAMPNLVVEVIGSDDDDDNRDYVETLYMYRSLDGPRNDAPKFVTIL

>AtCOI4 AT4G24390.1

MTEEDSSAKMSEDVEKYLNLNPPCSSSSSSSSAATFTNKSRNFKSSPPPCPDHVLENVLENVLQFLTSRCDRNAVSLVCRSWYRVEAQTRLEVFIGNCYSLSPARLIHRFKRVRSLVLKGKPRFADFNLMPPNWGAQFSPWVAATAKAYPWLEKVHLKRMFVTDDDLALLAESFPGFKELTLVCCEGFGTSGIAIVANKCRQLKVLDLMESEVTDDELDWISCFPEGETHLESLSFDCVESPINFKALEELVVRSPFLKKLRTNRFVSLEELHRLMVRAPQLTSLGTGSFSPDNVPQGEQQPDYAAAFRACKSIVCLSGFREFRPEYLLAISSVCANLTSLNFSYANISPHMLKPIISNCHNIRVFWALDSIRDEGLQAVAATCKELRELRIFPFDPREDSEGPVSGVGLQAISEGCRKLESILYFCQNMTNGAVTAMSENCPQLTVFRLCIMGRHRPDHVTGKPMDDGFGAIVKNCKKLTRLAVSGLLTDEAFSYIGEYGKLIRTLSVAFAGNSDKALRYVLEGCPKLQKLEIRDSPFGDVGLRSGMHRYSNMRFVWLSSCLISRGGCRGVSHALPNVVVEVFGADGDDDEDTVTGDYVETLYLYRSLDGPRKDAPKFVTIL

>BdCOI1 BRADI2G23730.2

MGGEAPEPRRLSRALSLDGSVVPEEALHLVMGYVDHPRDREAASLVCRRWHRIDALTRKHVTVPFCYAASPARLLARFPRLESLAVKGKPRAAMYGLIPDDWGAYARPWVAELAAPLECLKTLHLRRMVVTDDDLATLVRARGHMLQELKLDKCSGFSTDALRLVARSCRSLRTLFLEECSITDNGTEWLHDLAVNNPVLVTLNFYMTYLRVVPADLELLAKNCKSLISLKISDCDLSDLIGFFQMATSLEEFAGAEFNEQGELTKYGNVKFPSRLCSLGLTCLGTNEMHIIFPFSTVLKKLDLQYTFLTTEDHCQLIAKCPNLLVLAVRNVIGDRGLGVVADTCKKLQRLRVERGDDDPGLQEEQGGVSQVGLTAVAVGCRELDYIAAYVSDITNGALESIGTFCKNLCDFRLVLLDRQERITELPLDNGVRALLRGCAKLRRFALYLRPGGLSDVGLGYIGQHSGTIQYMLLGNVGDTDNGLLLFASGCVNLRKLELRSCCFSERALALAMLQMPSLRYVWVQGYKASQTGRDLMLMARPFWNIEFTPPSPENANRLTVDGEPCVDRYAQILAYYSLAGKRSDCPQSVVPLYPA

>BdCOI2 BRADI3G58320.1

MRDPEEDSDSTPSRMSEDDDCSGGGGGGGWTPDLRGGGGRWAPPDQVLEIVLESVLEFLTAARDRNAASLVCRSWYRAEAQTRRELFIGNCYAVSPRRAVERFGGVRAVVLKGKPRFADFSLVPHGWGAYVSPWFAALGPAYPRLERICLKRMTVSDDELALIPRSFPLFKELSLVCCDGFSTRGLAIIAEGCRHLRVLDLTEDYFHEEENEVVDWISKFPESNTSLESLVFDCVSVPFNFEALEALVARSPALRRLRVNDHVSIEQLRCLMARAPRLTHLGTGSFRSEPGSGGTSSVSELATSFAASRSLVCLSGFLDVNAEYLPAIYPVCANLTSLNFSFASLTAEEIIPVINHCVSLRTFWVLDTVGDEGLRAVAETCSDLRELRVFPLDATEDSEGSVSDIGLQAISEGCRKLESILYFCQRMTNAAVIAMSENCPNLVVFRLCIMGRHRPDRITGEPMDEGFGAIVMNCKKLTRLSVSGLLTDKAFAYIGKYGKLIKTLSIAFAGNSDMSLQHVFEGCTRLQKLEVRDSPFGDKGLLSGMNYFYNMRFFWMNSCRLTAKGCRDVAQQMPNLVVEVMKEHPEDEGETDTVDKLYLYRSLAGPRNDAPSFVNIL

>BdCOI3 BRADI4G05157.1

MSEDAQRYGGGGGSGGGGSSGGIGALSLDLLCQVLDRVQERRDRKACRLVSRAFARAEAAHRRALRVLRREPLPRLLRAFPALELLDLSACASLDDASLAAAVAGADLGAVRQVCLARASGVGWRGLDALVAACPRLEAVDLSHCVGAGDREAAALAAAAGLRELNLEKCLGVTDMGLAKVAVGCPKLEKLSFKWCREISDIGVDLLVKKCRELRNLDISYLEVSNESLRSISTLEKLEELSMVGCLCIDDKGLELLSRGSNSLQSVDVSRCDHVTSEGLASLIDGHSFLQKLNAADSLHEIGQNFLSKLATLKETLTMLRLDGFEVSSSLLLAIAEGCNNLVEVGLSKCNGVTDEGISSLVARCGYLRKIDLTCCNLLTDNALVSIADNCKMLECLLLESCSSLSEKGLERIATCCPNLSEIDLTDCGVNDAALQHLAKCSELLILKLGLCSSISDKGLGFISSKCVKLTEVDLYRCNSITDDGLATLAKGCKKIKMLNLCYCNKITDGGLSHLGSLEELTNLELRCLVRITGIGISSVAIGCKSLVEIDLKRCYSVDDSGLWALARYALNLRQLTISYCQVTGLGLCHLLSSLRCLQDVKMVHLSWVSIEGFEMALRAACGRLKKLKMLSSLKSVLSPELLQLLQACGCRIRWVNKPLVYKDGI

>BdCOI4 BRADI4G06170.1

MEPSPVTDSEETEGVRCGSGSSVRRPSRRPQLAEGARVLTENATRVAAANPRRARLTVLMPVRSPILRMMPLRARSASATFRTSPSAKALRAPVAISTIQRFVYSPNLQITQPAQHHFSLEDLPDAMLTEIIKRITLTSDLNSFSLVSKRLYTIEADQRGAIRVGCNLCPATEALASLCTRFPNLWKVDIDYSGWTPGHGNQLDNQGFLEFSSRCPSLTDLTLSFCSRIHDSGLGCLDYCKKLMSLRLKSAPKITSRGLLSVVVGCKSLSTLHIVDCHKIGSADWLEYLGQNGSLEELVVKNCQRISQYDLLKFGPGWMELQKFEFMTDAGLWDHLERCYDSSYNAHNPSRYDFHCDSLKDLTFARFTTGPEIGLRNLLGKCKALEKLRLEYVHGLNDNDLIVLSKSCSKLKSISLWLTPIFHDFYKCTTSFTDDSLKALALNCPMLQTVELTFGDCEPTYPSEIGFTRKGLVMLMKSCPIRVLVLNGANFFDDKGIKALSFAPLLETLELIDCKAITDIGMRFIVHIPRLSNLALRWCEHVTDDGVAELVQAHKLESLSIECCLQVSLEAVQGAARSVHYYTKCVSNSLLGKRMFLKYCS

>BdCOI5 BRADI4G08400.1

MGQCPSASRHHRHHRKLPPPPSPAAQATSPRFSSDGAAAAAEDAAAAATADYTSDLPEELLAVVFGFLGSGDRKRCSLVCRRWLAAEAASRLRLALDARAPLLAAAPGILARFSAVSKLALKCDRRAESVGDPALALVAQRLGPGLRRLKLRSVRAVTDHGVATLAAAAGNLRKLSVGSCAFGAKGIEAVLRSCPQLEELSVKRLRGLANSEPVAISGPRLQSLSLKELYNGQCFSCLITQSPNLKTLKVIRCSGDWDPVLQAVPQDALLAELHLEKLQVSDHGVSALCGLEVLYLAKAPEVTDVGLAALATKSPLLRKLHVDGWKANRIGDRGLATVARKCAALQELVLIGVNLTSVSLELIAANCPTLERLALCGSDTFGDAEISCVATKCASLRKLCIKACPVSDAGMDKLAEGCPRLVKVKVKKCRGVTFECAERLRASRHGALSVNFDTPGVAGELQDARSVDESGVLENAGSDTVPDDLDDQIGIPDLSCGSSGRPSGWKARLGAFMSRSLSVSVFRRRLQASSCNS

>BdCOI6 BRADI4G11900.1

MRGRRVDLINVALPEELLEEVLLRVGGAKRDLDACALVCRRWRRLERGTRRSAKLPASGAGANELARLVAETFSALVDVRVDERLSAGTGPGLVAVPPPGSRRRRVSGSTSARRRRMSRSRWLFPSDQTANGDGIEGNFFTDVGLTNLAEGCKGLEKLSLKWCTNITSTGLVRISENCKNLTSLDIEACYIGDPGLVAIGEGCKRLNNLNLNYVEGATDEGLIGLIKNCGPSLISLGVTICAWMTDASLRAVGSHCPKLKILSLEAEHVKNEGVISVAKGCPLLKSLKLQCVGAGDEALEAIGSYCSFLESFCLNNFERFTDRSLSSIAKGCKNLTDLVLSDCQLLTDKSLEFVARSCKKIARIKINGCQNMETAALEHIGRWCPGLLELSLIYCPRIRDSAFLELGRGCSLLRSLHLVDCSRISDDAICHIAQGCKNLTELSIRRGYEIGDKALISVAKNCKSLKVLTLQFCERVSDTGLSAIAEGCSLQKLNLCGCQLITDDGLTAIARGCPDLIFLDIGVLQIIGDMALAEIGEGCPQLKEIALSHCPEVTDVGLGHLVRGCLQLQVCHMVYCKRITSTGVATVVSSCPRLKKLFVEEAKVSERTRRRAGPVLAFLCTGI

>BdCOI7 BRADI5G08680.1

MTYFPEEVVEHIFSFLPGQHDRNTVSLVCKVWYEIERLSRRTVFVGNCYAVRPERVVLRFPNMRALTVKGKPHFADFNLVPPDWGGYAAPWIEAAARGCVGLEELRMKRMVVTDESLELLAKTFPRFRALILISCEGFSTDGLAAIASHCKLLRELDLQENEVEDRGPRWLSCFPDSCTSLVSLNFACIKGEVNAGSLERLVARSPNLRSLRLNRSVPVDTLSKILMRTPNLEDLGTGNLADDFQTESYIRLALAFDKCKMLRSLSGFWDASPFCLPFIYPVCAQLTGLNLSYAPTLDSSDLTKMISHCVKLQRLWVLDCIADKGLQVVASSCKDLQELRVFPSDFYIAGYSPVTEEGLVAISLGCQKLSSLLYFCHQMTNAALITIAKNCPNFTRFRLCILEPGKPDAMTNQPLDEGFGAIVRECKGLRRLSISGLLTDKVFMYIGTYAKELEMLSIAFAGDSDAGMMHVMKGCKNLRKLEIRDSPFGDAALLENVAKYETMRSLWMSSCNVTEKGCQVLASKMPMLNVEVINELDENNEMDENHGGLPKVDKLYVYRTTAGGRDDAPNFVKIL

>HvCOI1 MLOC_18524.1

MSGDERHLGRTMSFGIPDVALGLVMGCVEDPWDRDAISLVCRHWCKVDALSRKHVTIAMAYSTTPDRLFRRFPCLESLKLKAKPRAAMFNLIPEDWGGYASPWIRELSASFQFLKVLHLRRMIVSNDDLAVLVRAKAHMLVSLKLDRCSGFSTPSLALVARRCKKLETLFLEESSVAEKENDEWLRELATSNTVLETLNFFLTDLRASPAHLLLLVRNCRRLKTLKISDCFMSDLVDLFRTAETLQDFAGGSFDDQDQGGNYANYYFPPSVQRLSLLYMGTNEMQILFPYGATLKKLDLQFTFLTTEDHCQLVQRCPNLEVLEVRDVIGDRGLEVVAQTCKKLHRLRVERGDDDQGGLEDEQGRVTQVGLMAVAQGCPDLEYWAVHVSDITNVALEAIGTFSKNLNDFRLVLLDREVHIADLPLDNGVRALLRGCTKLRRFAFYVRPGALSDIGLNYVGEFSKTVRYMLLGNVGGSDDGLLAFARGCPSLQKLELRSCCFSERTLAVAALQLKSLRYLWVQGYKASSTGTDLMAMVRPFWNIEFIAPNQSEPCPEGQAQVLAYYSLAGARTDCPMSVIPLYPSVGS

>HvCOI2 MLOC_52024.5

MPYFPDEVVEHILGFVSSHRDRNAASLVCHAWYRVEGLTRRSVFISNCYAVRPERVHARFPCLRSLTVKGRPCFADFNLVPAGWGATAEPWVDACARTCPGLEELRLKRMVITDDCLNHLARSFPNLRSLVLVSCEGFSTDGLATIATNCRFLKELDLHGSQVEFRGPHWFSCFPKPSTSLESLNFACLDGTVSANALESLVARSPNLKSLRLNRAVPAAVLANILTSAPKLVDLGTGLVAQNNNADALSLYNAIQQCSSLNSLSGFWDSPRWITPVIHYICKNLTCLNLSYAPTFQTADLIGAIRHCQNLRHLWVLDHIGDAGLKVVASCCLELQELRVFPANADVLASTDVTEEGLVAVSSGCRKLSSVLYSCSRMTNSALITVAKNCSRITSFRLRICLHGSVDAVTGQPLDEGFGAIVRSCKGLRRLSMSGLLTDSVFLYIGMYAERLETLSVAFAGDSDDGMIYVLNGCKNLRKLEMRNCPFGDTALLAGMHRYEAMRSLWMSSCDITLGGCRSLAATMPNLNVEVVSQVDGVSCDAKKVEKLYVYRTLAGPRGDAPGFVSAL

>HvCOI3 MLOC_56088.1

MTYFPEEVVEHIFSFLPAQCDRNTVSLVCKVWYEIERLSRRTVFVGNCYAVRPERVVLRFPNVRALTVKGKPHFADFNLVPPDWGGYAGPWIEAAARGCVGLEELRMKRMVVSDESLELLAKSFPRFRALVLISCEGFSTDGLAAIASHCKLLRELDLQENEVDDRGPRWLSCFPDSCTSLVSLNFACIKGEVNAGSLERLVARSPSLRSLRLNRSVSVDTLSKILMRAPNLEDLGTGNLTDDFQAESYLRLTLALEKCKLLRSLSGFWDASPFCLPFIYHVCGQLTGLNLSYAPTLDSSDLTKMISHCAKLQRLWVLDCIGDKGLQVVASSCKDLQELRVFPSDFYIAGYSPVTEEGLVAISLGCPKLSSLLYFCHQMTNAALHTIAKNCPNFTRFRLCILEPGKPDAMTNQPLDEGFGAIVRECKGLRRLSISGLLTDKVFMYIGKFAKQLEMLSIAFAGDSDAGMMHVMEGCNNLRKLEIRDSPFGDAALLENVTKYETMRSLWMSSCNVTEKGCQILASKMPMLNVEVINEVDESNEMDENHGIPKVDKLYVYRTTAGARDDAPNFVKIL

>HvCOI4 MLOC_67830.1

MSASPSEGDEAVINGVLTDDELRAVLTRLGPESERDAFGLVCRRWLRIQSSERRRLRARAGPSMLRRLAARFPGILELDLSQSPSRSFYPGVIDDDLNVIAGGFCNLRVLALQNCKGITDVGMVKLGEGLPCLQTLDVSHCKKLSDKGLKVVASGCRKLRQLHIAGCRLITDNLLRAMSKSCLNLEELGAAGLNSITDAGISALADGCHKMKSLDISKCNKVGDPGICKIAEASSSSLVSLKLLDCSKVGNKSIHSLAKFCCNLETLIIGGCQHISDESIEALALACCSRLRILRMDWCLKITDASLRSLLCNCKLLAAIDVGCCDQITDAAFQGMEANLFRSELRVLKINNCVGLTVLGVSRVIESCKALEYLDVRSCPQVTRQSCEEAGLQLPGSCKVNFEGSLSESDSSVDRFF

>HvCOI5 MLOC_72489.1

MGLGCIAVGCPDLRDLTLNWCLGITDLGIQLLALKCNKLRNLHLSYTMISKDCLPAIMKLPNLEVLALVGCVGIDDDALSGLENESSKSLRVLDMSTCRNVTHTGVSSVVKAVPNLLELNLSYCCNVTASMGKCFQMLPKLQTLKLEGCKFMADGLKHIGISCVSLRELSLSKCSGVTDTDLSFVVSRLKNLLKLDITCNRNITDVSLAAITSSCHSLISLRIESCSHFSSEGLRLIGKRCCHLEELDITDSDLDDEGLKALSGCSKLSSLKIGICMRISDQGLIHIGKSCPELRDIDLYRSGGISDEGVTQIAQGCPMLESINLSYCTEITDVSLMSLSKCAKLNTLEIRGCPSISSAGLSEIAIGCRLLAKLDVKKCFAINDVGMFFLSQFSHSLRQINLSYCSVTDIGLLSLSSICGLQNMTIVHLAGITPNGLLAALMVSGGLTRVKLHAAFRSMMPPHMLKVVEARGCAFQWIDKPFKVEQERCDIWQQQSRDVLVR

>HvCOI6 MLOC_73542.1

RGAASLVCRSWHRAESATRASVAVRNILAASPARAARRFPNAHHILLKGRPRFADFNLLPPGWAASAFRPWAAALAAAAFPALRSLSLKRITVTDDDLDLLARSLPPSFRELSLLLCDGFSSRGLASLASHCRGLRVLDVVDCELNEEEDDEVSDWVAAFPRGHTDLESLSFECFTPQVPFAALEALVARSPRLRRLRVNQHVSLGQLRRLMTLTPRLTHLGTGSFRPGDGADDEGLDFGQMLTAFASAGRANSLVSLSGFRDLAPEYLPTIATVAANLTTMDLSFAPVNPDQVLLFIGQCRSLETLWVLDSVRDEGLQAVAMCCKKLQVLRVLPLDAHEDADELVSEVGLTAISEGCRDLRSILYFCQRMTNVAVVTMSQNCPELKVFRLCIMGRHRPDHVTGEPMDEGFGAIVRNCSRLTRLSTSGHLTDRAFEYIGKYGSSLRTLSVAFAGDSDLALQHILQGCSKLEKLEIRDCPFGDAGLLSGMHHFYNMRFVWMSGCSLTLEGCKAVARQLPRMVVELINSQPENEKTDGVDILYMYRSLEGPREDVPPFVRIL

>HvCOI7 MLOC_80547.1

MGGEVPEPRRLSRALSFGVPDEALHLVMGYVEAPRDREAASLVCRRWHRIDALTRKHVTVAFCYAADPARLLARFPRLESLALKGRPRAAMYGLISDDWGAYAAPWVARLAAPLECLKALHLRRMTVADDDVAALIRSRGHMLQELKLDKCSGFSTDALRLVARSCRSLRTLFLEECVITDEGGEWLHELAVNNSVLVTLNFYMTELKVAPADLELLAKNCKSLLSLKISECDLSDLIGFFEAASALQDFAGGAFNEVGELTKYEKVKFPPRVCFLGLTFMGKNEMPVIFPFSASLKKLDLQYTFLTTEDHCQLISKCPNLFVLEVRNVIGDRGLEVVGDTCKKLRRLRIERGDDDPGLQEEQGGVSQLGLTAVAVGCRDLEYIAAYVSDITNGALESIGTFCKNLYDFRLVLLDRQKQVTDLPLDNGVRALLRSCTKLRRFALYLRPGGLSDIGLDYIGQYSGNIQYMLLGNVGESDQGLIRFAIGCTNLRKLELRSCCFSEQALSLAVLHMPSLRYIWVQGYKASPAGLELLLMARPFWNIEFTPPSPGGLHRMTLDREPCGERQAQVLAYYSLAGQRQDCPDWVTPLHPAA

>HvCOI8 MLOC_9864.2

MVVTDECLEMIASSFRNFQVLRLVSCEGFSTAGLAAITEGCRNLRELDLQENYIEDCSNHWLSYFPESFTCLETLNFSSLEGEVNFAVLERLVSRCRNLKTLKLNNAIPLDKVASLLRKAPQLVELGTGKFSAEYHSDLFAKLEAVFAGCKSLRRLSGAWDAVPDYLPAFYGVCEGLTSLNLSYATVRGPELIKFIGRCKNLQQLWVMDLIGDHGLAVVACSCSKLQELRVFPSEPFGAGQVLLTERGLVDVSASCPMLESVLYFCGQMTNEALITIAKNRPNFTCFRLCILEPRTPDYVTRQSLDAGFSAIVESCKGLRRLSVSGLLTDLVFKSIGANGNRLEMLSIAFAGNSDLGLHYILSGCKSLKKLEIRDCPFGNKPLLANAAKLETMRSLWMSSCSLTLGACRQLAEKMPRLTVEIMNDPGRTCPVESLPDDSPVETLYVYRTIAGPRSDTPDYVQIV

>OsCOI1 LOC_Os01g63420.1

MPPYETARAEPASNNKQRPISGETAAGSSRSGPDPMGGEVPEPRRLNRALSFDDWVPDEALHLVMGHVEDPRDREAASRVCRRWHRIDALTRKHVTVAFCYAARPARLRERFPRLESLSLKGKPRAAMYGLIPDDWGAYAAPWIDELAAPLECLKALHLRRMTVTDADIAALVRARGHMLQELKLDKCIGFSTDALRLVARSCRSLRTLFLEECHITDKGGEWLHELAVNNSVLVTLNFYMTELKVAPADLELLAKNCKSLISLKMSECDLSDLISFFQTANALQDFAGGAFYEVGELTKYEKVKFPPRLCFLGLTYMGTNEMPVIFPFSMKLKKLDLQYTFLTTEDHCQIIAKCPNLLILEVRNVIGDRGLEVVGDTCKKLRRLRIERGDDDPGLQEEQGGVSQLGLTAVAVGCRELEYIAAYVSDITNGALESIGTFCKNLYDFRLVLLDRERQVTDLPLDNGVCALLRNCTKLRRFALYLRPGGLSDDGLSYIGQYSGNIQYMLLGNVGESDHGLIRFAVGCTNLQKLELRSCCFSERALSLAVLQMPSLRYIWVQGYRASQTGLDLLLMARPFWNIEFTPPSPESFNHMTEDGEPCVDSHAQVLAYYSLAGRRSDCPQWVIPLHPA

>OsCOI2 LOC_Os05g37690.1

MGGEAPEARRLDRAMSFGGAGSIPEEALHLVLGYVDDPRDREAVSLVCRRWHRIDALTRKHVTVPFCYAASPAHLLARFPRLESLAVKGKPRAAMYGLIPEDWGAYARPWVAELAAPLECLKALHLRRMVVTDDDLAALVRARGHMLQELKLDKCSGFSTDALRLVARSCRSLRTLFLEECSIADNGTEWLHDLAVNNPVLETLNFHMTELTVVPADLELLAKKCKSLISLKISDCDFSDLIGFFRMAASLQEFAGGAFIEQGELTKYGNVKFPSRLCSLGLTYMGTNEMPIIFPFSALLKKLDLQYTFLTTEDHCQLIAKCPNLLVLAVRNVIGDRGLGVVADTCKKLQRLRVERGDDDPGLQEEQGGVSQVGLTTVAVGCRELEYIAAYVSDITNGALESIGTFCKNLCDFRLVLLDREERITDLPLDNGVRALLRGCTKLRRFALYLRPGGLSDTGLGYIGQYSGIIQYMLLGNVGETDDGLIRFALGCENLRKLELRSCCFSEQALARAIRSMPSLRYVWVQGYKASKTGHDLMLMARPFWNIEFTPPSSENANRMREDGEPCVDSQAQILAYYSLAGKRSDCPRSVVPLYPA

>OsCOI3 LOC_Os03g15880.1

MGGEAGERRLGRAMSFGIPDVALGLVMGFVEDPWDRDAISLVCRHWCRVDALSRKHVTVAMAYSTTPDRLFRRFPCLESLKLKAKPRAAMFNLIPEDWGGSASPWIRQLSASFHFLKALHLRRMIVSDDDLDVLVRAKAHMLSSFKLDRCSGFSTSSLALVARTCKKLETLFLEDSIIAEKENDEWIRELATNNSVLETLNFFLTDLRASPAYLTLLVRNCRRLKVLKISECFMLDLVDLFRTAEILQDFAGGSFDDQGQVEESRNYENYYFPPSLLRLSLLYMGTKEMQVLFPYGAALKKLDLQFTFLSTEDHCQLVQRCPNLEILEVRDVIGDRGLEVVAQTCKKLQRLRVERGDDDQGGLEDEHGMVTQVGLMAVAQGCPHLEYWAVHVTDITNAALEAIGTYSSSLNDFRLVLLDREANITESPLDNGVRALLRGCTKLRRFAFYVRPGALSDVGLGYIGEFSKTIRYMLLGNVGESDQGLLQLSTGCPSLQKLELRGCFFSERALAVAVLQLKSLRYLWVQGYKASPNGTDLMAMVRPFWNIEIIAPNQDEVCPDGQAQILAYYSLAGMRSDYPHSVIPLYPSV

>OsCOI4 LOC_Os02g52230.1

MSTSPSCSSSSPIPQSLTLASTSSSSSSSGMRDAGEGSDSPPSEMSEDGSGGSGDGDGDGDGGGGGGDRWMPDLRGGNGGGGGGGGGGGRWAPPDQVLENVLESVLEFLTAARDRNAASLVCRSWYRAEAQTRRELFIGNCYAVSPRRAVERFGGVRAVVLKGKPRFADFSLVPYGWGAYVSPWVAALGPAYPHLERICLKRMTVSNDDLALIAKSFPLFKELSLVCCDGFSTLGLAAIAERCRHLRVLDLIEDYIDEEEDELVDWISKFPESNTSLESLVFDCVSVPFNFEALEALVARSPAMRRLRMNHHVTVEQLRRLMARAPQLTHLGTGAFRSEPGPGGALSVTELATSFAASRSLICLSGFRDVNPEYLPAIHPVCANLTSLNFSFANLTAEELTPIIRNCVRLRTFWVLDTVGDEGLRAVAETCSDLRELRVFPFDATEDSEGSVSDVGLQAISEGCRKLESILYFCQRMTNAAVIAMSKNCSDLVTFRLCIMGRHRPDRITGEPMDDGFGAIVMNCKKLTRLSVSGLLTDKAFAYIGKYGKLIKTLSVAFAGNSDMSLQSVFEGCTRLQKLEVRDSPFSDKGLLSGLSYFYNMRFLWMNSCRLTMRGCRDVAQQMPDLVVEVMKDHLDDEGEMETVDKLYLYRSLAGARNDAPSFVNIL

>OsCOI5 LOC_Os11g31620.1

MVFFPEEVVEHILGFLASHRDRNAVSLVCREWYRVERLSRRSVLVRNCYAARPERVHARFPGLRSLSVKGRPRFVPAGWGAAARPWVAACVAACPGLEELRLKRMVVTDGCLKLLACSFPNLKSLVLVGCQGFSTDGLATVATNCRFMKELDLQESLVEDRDSRWLGCFPKPSTLLESLNFSCLTGEVNSPALEILVARSPNLRSLRLNRSVPLDVLARILCRRPRLVDLCTGSFVRGNIVGAYAGLFNSFQHCSLLKSLSGFWDATSLFIPVIAPVCKNLTCLNLSSAPMVRSAYLIEFICQCKKLQQLWVLDHIGDEGLKIVASSCIQLQELRVFPANANARASTVTEEGLVAISAGCNKLQSVLYFCQRMTNSALITVAKNCPRFTSFRLCVLDPGSADAVTGQPLDEGYGAIVQSCKGLRRLCLSGLLTDTVFLYIGMYAERLEMLSVAFAGDTDDGMTYVLNGCKNLKKLEIRDSPFGDSALLAGMHQYEAMRSLWLSSCNVTLGGCKSLAASMANLNIEVMNRAASINEADNANDAKKVKKLYIYRTVAGPRGDAPEFISTF

>OsCOI6 LOC_Os03g08850.1

MSEEDDDQPPPLPAQKRPRASPPPDQVLDNVLETVLQFLDSARDRCAASLVCRSWSRAESATRASVAVRNLLAASPARVARRFPAARRVLLKGRPRFADFNLLPPGWAGADFRPWAAAVAAAAFPALASLFLKRITVTDDDLDLVSRSLPASFRDLSLLLCDGFSSAGLASIASHCRGLRVLDVVDCEMNDDDDEVVDWVAAFPPGTTDLESLSFECYVRPVSFAALEALVARSPRLTRLGVNEHVSLGQLRRLMANTPRLTHLGTGAFRPGDGPEDVGLDIEQMASAFASAGRTNTLVSLSGFREFEPEYLPTIAAVSGNLTNLDFSYCPVTPDQFLPFIGQCHNLERLYVLDSVRDEGLQATARTCKKLQVLHVLPLNALEDADELVSEVGLTAIAEGCRGLRSTLYFCQSMTNAAVIAISQNCVDLKVFRLCIMGRHQPDHVTGEPMDEGFGAIVRNCSKLTRLSTSGHLTDRAFEYIGKYAKSLRTLSVAFAGDSNLALQHILQGCSKLEKLEIRDCPFGDAGLLSGMHHFYNMRFLWMSGCNLTLQGCKEVARRLPRLVVELINSQPENERTDSVDILYMYRSLEGPREDVPPFVKIL

>OsCOI7 LOC_Os05g05800.1

MGRGGSRAACAAAAPPWHSLPDEVWEHAFSFLPAAADRGAAAGACSSWLRAERRSRRRLAVANCYAAAPRDAVERFPSVRAAEVKGKPHFADFGLVPPAWGAAAAPWIAAAADGWPLLEELSFKRMVVTDECLEMIAASFRNFQVLRLVSCDGFSTAGLAAIAAGCRHLRELDLQENEIEDCSIHWLSLFPESFTSLVTLNFSCLEGEVNITVLERLVTRCHNLKTLKLNNAIPLDKLASLLHKAPQLVELGTGKFSADYHSDLFAKLEAAFGGCKSLRRLSGAWDAVPDYLPAFYCVCEGLTSLNLSYATVRGPELIKFISRCRNLQQLWVMDLIEDHGLAVVASSCNKLQELRVFPSDPFGAGFLTERGLVDVSASCPMLESVLYFCRRMTNEALITIAKNRPNFTCFRLCILEPHTPDYITREPLDAGFSAIVESCRGLRRLSISGLLTDLVFKSIGAHADRLEMLSIAFAGNSDLGLHYILSGCKSLKKLEIRDCPFGDKPLLANAAKLETMRSLWMSSCLLTLGACRQLARKMPRLSVEIMNDPGRSCPLDSLPDETPVEKLYVYRTIAGPRCRMKIHSLFQDMEMHGL

>PpCOI1 PP1S277_20V6.1

MEFERRRAPGAGSGIPLSDETLACVLKYVENWQDRAAVSLVCQQWRRVDGATRKFVTISYMYSTNPELLTRRFKRLEGVKIKGKPRAEEYGLLVPNWGGYAEPWIRELGRVYRGLQTLLLRRCQVSDSDLELIASSPFHSVLQVLHLHKCAGFSTSGLLPVAKACRSLRTLSIEDSNVNDEGGEWLHVLARHNTVLEVLNFAVLGLEDVDVADLALLLEKCKSLVSLKVGEIELVDMVGALGKSSSLLELGAGSCNYLNDEDSRVYASISLPLQLTSLSGLWSMGDFGLCMILPIAPNLKKLDLKFTFLSRKAYCQLFSQCHSLEELQIRNGVGDEGLEVLGKSCKSLRRLRIEHDEAGAITQRGVVAVAQGCNNLQQLVLYVSDISNAALAMVGQGCPHLTDFRLVLTGTQHVVDLPLDDGFKLLLKGCPNISKLAVYLRHGGLTDKGMSYMGDFGKNLKWVLLGCTGESDIGLANFAYKAQKLERLEIRDCPFGEAGLVAAVVAMSSLKFLWVQGYRAPEAGYQLLGLARPWLNIEISLPSGTMPGQLIAHYAIVAARNDYPPDVKVLVEETEELEGMLPPCTNRRTVDP

>PpCOI2 PP1S211_131V6.1

MEMGREKRPSGSGTGLSDETLACVLKYVESAEDRASVSLVCKQWRLVDGATRKFVTIAYMYSTSPEMLTRRFKRLEGLKLKGKPRAAEYDLLVPDWGGYAEPWIRDLGRAYTSLQTLQLRRCQVSNADLTLIASSPCQASLQVLYLHKCAGFSTAGLLPVAKSCRSLKSLSVEDSDVTDEGGEWLFELARNNSVLEVLNFAVLGLEDVDAADLVLLVERCKSLVSLKVGEVEMVDMISAISRASSLTEFGTGSCNFFGDEDSRTHVSISLPSSLTGLSGLWAMSDPGLAMVLPIAPNLRKLDLKFTLLSRKAYCQLFSQCHALEELQVRNAVGDEGMEVIGKTCKSLRRLRVEHDNAGAITQRGVVAVAQGCARMQQLIVYVSDITNAALAMLGQCCAQLTDFRLVLETAARRVVDLPLDDGIKLLLKGCRKISKLAVYLRHGGLTDRGMGYIGEFGTNLKWLLLGCTGESDIGLASLAYKAQRIERLECRDCPFGEAGLAAAVVAMSSLKFIWIQGYRAPWAGEHLLALSRPYLNIEVISSTDTQPGQLIAHYTTVGPRTDNPLEVKQLTLNPDDHLQEMRPSLHSPGSTRH

>PpCOI3 PP1S24_60V6.1

MVMGVVDAEKLESRKSLPCGMSDETLACVLNHIESPQDRAAVSMVCQQWRRVDGMTRKFVTIANMYATSPASLTRRFKGLEGIKLKGKPRAAEYNLVRSDWGGYGEPWLKVLGRQYADLHILQLRRLTVLDSDLELIASSTFSSALHVLHLHKCVGFTTKGLLPVVRACRSLRRLSLEDSEVEDKGGEWLHALALNDSTLEELHFGVLGIEAIDIEDLTILVEKSKSLVCLKVAEIELLDMIDVLQRVPSLEDLGAGSCNYLGAKDVDDFVSIPWPKKLNALSGMWSLMDSGLPQILPIAPNLIKLDLKYTLLSCEGHCLLLSHCFSLQELQTRNTLGDDGMETLSRSCKGLKKLRVEDDETGAITQRGIVAVAQGCEQLVQLILYVANISNAALAMVGQGCPHLVDVRIVLEPSARYAPDFPLDDGLKLMLKGCVNLRRLAVYLRYGGLTDKGMEYIGVYGKNLQWLLVGCAGNSDVGLANFAHWAQRIQRLEIRDCPFGETGMAEAVSAMSSLKYLWVQGSRALEAGEKLSALSLPCLNVEVCPPPAGQPGGQLFAYYSLAGPRKDGPTGLKTFISNTVKDQ

>PpCOI4 PP1S140_66V6.1

MTMNPAIVTKFLEKHFSIQLTRQSSKKKCLSVLDETLDLIFSYLDPEDRASASLVCKHWHRVDGETREQVSVSNCYSVSPSALSKRFPNIEKFKIKGKPRAVEFNLLVDDWGGYASAWVEEIVRAYPRLHTLHFRRMDVSDDDLKILAQGCGSALQVLKLDKCSGFSTLGLQHIARSCRSLKTLYLEESDIEDEGHEWLLDLGRNVPGLERLNLASTGIEEGDVNDVLVVLMQNCKSLNSLKVGEMTLENFKEIMKYSTTPLLELGNGCYSMRNGVREELTFDAAFIPWVSRLKVLDLKFMNLNAAGHCQLLACCPLLEELEARIEILDEGLEVVGKTCKYLKRIRIDDQDSPGFITHRGLTAIAKGCRELEFLVMYMRDVTNSSLEAVGRYSENLNDFRIVLLKTLAHPEDLPLDKGVCSLLQGCPKLTRFSVYLRPGGLSDIGLSYIGKYGGRLKWILLGCSGESDQGLLDLAYGCQNLRRLELRGCPFSDAALAQGMMNMAKMKYLWVQGIGATEMLGRYLVGSHPCLHVEWMPSEQQLLAYYSLASHRTDTPPTVEILSQSRFDYDSEIFGDYDEECVGNLESGVFDGEYGEGPDVGDFDGEYGHGTNWDF

>PpCOI5 PP1S187_72V6.1

MSEVASWGDSPQLSNKKGKNRLANESTPSTECKFNLLPEPIIESIFNRVEARGDRNAMSQVCKLWQKMDGMTRKNIYISNCYSIAPSDVSRRFKSLQKIKIKGKPRAYEFGLLVERWGGHAGPWIGEMSRAYPELLGLSMRRMDVTDNDLRILASRCPKLQKLKLHKCCGFSTGGLEHITRSCRTLRVLDIEESDDIEDTGGPWLELLENSDGRLESLNIASAGLEEENIKEVLPVVGRSLKCISSLKVSDMELGSFFKILDNSNVPVVELGLGCYCSSPEDPKELASSFALRLSKVKVLDLKFATLNAEIQIELLRHCSSLEELELRSAVGDRGMQVIGETCKQLKRIRVDQDTSEYMTDYITQKGMIAICEGCRELDFLVMYLSDVNNEALAAVGRCLPKLTDFRIVLLEVRNDVKDLPLDEGVRLLLQGCPILTRFSVYLRQGGLSDKGVGYIGQFGTKLKWVLLGCSGETDKGLRLMAEGCRQLERLELRCCPFTELQLASSILNSWRHLKYLWVQGVGATSGLGVDLVTHKSGFLVEFMGETQQILGYYSATRPRTDNPRSVCLINYVPPEDRPEADAKGFQGNTHASQGDAHSFYSDYADGYVRNSCSEAEAFYPGFNENNLYPDADENAFYTGVVDAGLYLGVGDSCGYPEFGEDEAFIY

>PpCOI6 PP1S49_196V6.1

MAAEVVWREDGNIRSRFFRGNEHGPTKKCIFNQLPESVIELIFDRLGSKGDRRAISQVCKQWHRVDGLTRKNIYIFNCYSIAPSNLSKRFPNLEKIKIKGKPRAYEFGLLVESWGAHAGPWIEEIASVYPNLEGLALRRMDITDKDLMLLASRCPNLRRSSHSRDYFCTWQIVSAGLSSLSIDNCGSRLWQKLKLHKCSGFSTRGLEFITRSCRTLRVLDIDESHDMEDTGGPWLQLLEKGDGKLESLNIASAGLEEESIKEVLLKLAPSLKCISSLRVSDMELGSFFKILDNSEVPVVELGLGCYSLSQEDPKELVPSFSSRLSKLKILDLKFTTLNAEIQIELLRHCCSVEELELRSVVGDWGMQVISENCKQLKKIRVDQDTSPYMTNHVTQKGMISICEGCRELDFLVMYLTDVNNAALAAVGQYLPKLSDFRIVLLEDQDDVEDLPLDDGIRLLLQGCPMLSRFSVYLRPGGLSNKGLGYIGEFGSKLKWVLLGSSGESDEGFRLMAEGCRQLERLELRNCPFSDKQLAISILNNLPHLKYLWVQGFGATSGLGVALVTQMPGFVVEVMATDQQILGYYTVTHPRTDSPDSVCVINYDPLLDNSAEVCEEGSQGDACSYYAERLYSEHIYSYLRVDEEAPYPGVNKNSHLPKIDENTLYSGVVGLEMYLAGDDNSASSEFCEDSACIC

>PpCOI7 PP1S12_347V6.1

MWCNCLVSVKIKFSGTMGWLQLRDGVQRGNSGWSSGLEIGYSGVGNSVCDENVEFLHPGVMLEASFPFADGSVAAWGRRICKMGSWRGAKATRVDEDHFPLLQFPDEIIEKVIGFLTNPVDRNSTSLVCTRLKAIEGESRETVLISNCYAIQPGTLKSRFPNAKSITIKGKPRIVDFSLIPHAEVWGAYATPWVDLLKEHYRPIRHLKMKRMTISDSDIKRFVSACGYSLERLEFEKCSGFSTTGLQYIAGACRNLVVLNLSEADILQGDAPYWMTSLVNTASSLRVLDLYLTEVEDVEQSVLERLAKQCHTLRLCDALKINHVLPVVTAACETVRHLGIGLSFQNGDSPNQIAEALGRCKELEGISAVWDPDEVSAMMLMPVAARLKTLDLTYALLEQPELTDLLGACVNLEDLQCTDVIRDRGLLEVGTCCKKLRSLVVQQDAAGFVTQNGLTAVAKGCFLLEKIIIYAADMTNEALETLATNCPNLSDIRICLVQKYDGSHPVVELEGNSTLNLGVKALLMKCPKARRLALCFSRFGLTNVVITDEGMKHIGEYGGNLHIITLTNCGGSNAGLEYIAKGCNELRKLELRHCPFGDASMEALARGCKSLKQLWVQACQVELRGVRLLAQRPGLTVEIVEESNNDGDITPWQLIAYASVAPPRKDLPDNIDYVHEGYCKPLYCKNYLCPSTV

>PpCOI8 PP1S3_119V6.1

MANLGRRRRSTVESEHCSLLQCPDEILEKIVDLISDTADRSAISLVCKSLKVLEGHTRGVVLVSNCYAIQPLTLKDRFPNAWSITIKGKPRIVDFALIPHAEVWGAYATPWMEILVNFDRPIRHLRMKRMTVSDSDIQLLVSRCGEGLQRLELEKCSGFSTFGLEIIARACRNLIELNISESEIQNGGHRSWLTTLVNTAKSLQVLDLSLTDVEHVEQSVLEKLAGQCHTLKLSAALEIERVLPVVEAANHSMRHLGTRFYSQNIENPHQIAEAFGRCRVLEGISAPLDLDEGSMMMVMPIAGRLTTLDLTYANLGQPELSDLLRTCINLEDFQCTDVIGDRGLRVIGTHCQKLRRLVVQQDAQGFVTQHGLTAVANGCFLLEKIIIYAADMTNAALETLANNCPGLSDIRICLVQKYHPSHPVIELEGNSTLNLGVRALLMRCRRARRLALCFSRFGLSNVVITDEGIRYIGEYGGNLHIITLTNCGSSDAGLESIAKGCTNLRRFELRHCPFGDRSMEFLATSCHSLKQLWVQACQVELNGVRVLARRKDLVVEVVKESTNENGDPIPWQFIAYASVASPRNDRPENIDYVHSQYDTPLRSEYFMCPSTGAELIDDLEDGLQNV

>PpCOI9 PP1S44_198V6.1

MPPSVFPDEVLEHVLVFLDSHKDRNSVSLVCKSWYKAEGWSRRKVFIGNCYAVSSATLIRRFPKLVSLEMKGRPRFTDFGLVPQNWGGFIQPWIQVMAEYYPGLEGLKLKRMTVSDESLRMIAVAFPNFRSLRLTSCDGFSTDGITEITKNCRNLAVLDLQENYMDIRNGDWLKAFPESLTSLESLNFATVKCAVDEEAFQCLEALVARCRCLKTLKVNKDISLGQLRSLLLRAPQLEELGTGIYNQNLSWGKLHELQGSLKRCKNLRSLSGLWEVIPMCLPTMYPVCLNLTSLDLSNVTLMTTDFTKFISYCTKVRRLLVQDFVGDKGLAAAAFNCKELQELRVYPVGVDGYVTEQGFIAISKGCPELRKILYFCKQMTNAAMVSFAQNCPKMTHFRLCIMKCYMEDCETGQPLDEGFGAVCRLCVDLRRLSLSGKMTDKTFEYIGQYAKKLEMLSVAFAGDSDDGMQYVLDGCPSLRKLEVRDCPFGDEALLTGIEKYESMRSLWMSSCHLTRDGCQFLASHNSSLNVEIIKDVDKAPLEQGQYVEKLYVYRTIAGPRADAPHFVETL

>PpCOI10 PP1S196_87V6.1

MLSVFPDEVLEHVLVFLTDHRDRNSVSLVCKAWCRTEGWSRRSVFIGNCYAASPNLLLRRFPKLTSLEMKGRPRFTDFGLVPSNWGAFIQPWIEALAEHYAGLECLRLKRMTVSDESLRIIALAFPNFRSLRLASCDGFTTDGLQWITRHCRHLKELDLQENEIQVRSVGWLTAFPESQTTLESLSFANIQTPLDEYDFHSLYALVARCPRLKRLKLNREVTLEQMQKLLLLAPQLEDLGTGAYNQKLTWGKLHDLQASFRKVKNIRSLSGFWDVSPRCLPTCFPICNELITLDLSTVALTTADFTKSTTNCVKLRRLLVQDSVGDEGLLHVARCCKQLTELRVYPFNNQSNVTEKGFIAISEGCRDMRKILYFCKQMSNAAMIQFARNCPNMTHFRMAMVTVYDRDCVTNDPLDEGFGAVCKLCKNLRRLSLSGLLTDKTFEYIGMYAKKLETLSVAFAGDTDLGMVNVLDGCPALRKLEVRDCPFGDEALLSGIEKYESMRALWMSSCQLTRDGVQFLADKNPNLNVEIIVDVEKSHDPEYVEKLYVYRSIAGPREDAPYFVDTL

>PtCOI1 POPTR_0004s03400.1

MKREFLDSTRSSPFPDEVLERVLSLLKSHKDRSAVSLVCKDWYNAESWSRTHVFIGNCYSVSPEIVARRFPIIKSVTLKGKPRFSDFNLVPENWGADVHPWLVVFATKYPFLEELRLKRMAVSDESLEFLAVNFPNFKVLSLLSCDGFSTDGLAAIATHCKSLTQLDIQENGIDDKSGGWLSCFPENFTSLEVLNFANLNTDVNFDALERLVSRCKSLKVLKVNKSISLEHLQRLLVCAPQLTELGTGSFTPELTTRQYAELESAFNQCKNLHTLSGLWEATALYLPVLYPVCSNLTFLNLSYTFLQSLELASLLRQCPRLRRLWVLDTVGDKGLEAVGSNCPLLEELRVFPADPFDEEIIHGVTEAGFVAVSYGCRRLHYVLYFCRQMTNAAVATIVQNCPDFTHFRLCIMNPGQPDYLTNEPMDEAFGAVVRTCTKLQRLSVSGLLTDLTFEYIGQYAKNLETLSVAFAGSSDRGMQCVLEGCPKLRKLEIRDCPFGNAALLSGLEKYESMRSLWMSACNVTMNGCRLLAREMPRLNVEVMKEDGSDDSQADKVYVYRSVAGPRRDAPPCVLTLSGL

>PtCOI2 POPTR_0002s10310.1

MITNKKPRSSDTDSNYMRDDRTDMSEDDDRSPPSDSIANDSCPTRTCTPGSGSGSSSIPEYSAPYPDQVLENVLENVLWFLTSRKDRNAASLVCRLWYRVEAMTRSDLFIGNCYAVSPERATSRFTRIRSVTLKGKPRFADFNLMPPNWGAHFAPWVSAMAKAYPWLEKIHLKRMSVTDDDLALLAESFSGFKELALVCCDGFGTSGLAVVASKCRQLKVLDLIESEVSDDEVDWILCFPDTETCLESLILDCVECPIDFDALERLVTRSPSLKKLRLNRFVSIGQLYRLMVRAPQLTHLGTGSFSQSEDVAQGELELDYGSAFAACKSLVCLSGFREIIPDYLPAIYPVCANLTSLNFSYANISAEQLKPIISNCHKLQTFWVLDSICDEGLQAVATTCKELRELRVFPFEAREDIEGPVSEVGLQAISEGCRKLQSILYFCPRMTNAAVIAMSKNCPDLVAFRLCIMGLHQPDHVTGEPMDEGFGAIVMNCKKLTRLAVSGLLTDRAFAYIGKYGKIVRTLSVAFAGDSDMGLKYVLEGCPKLQKLEIRDSPFGDAALLSGLHHYYNMRFLWMSACKLSHQGCQQIAQALPHLVVEVIKHEDNVDMDEYVDTLYMYRSLAGRRHDVPRFVSIL

>PtCOI3 POPTR_0001s33030.1

MNYFPDEVLEHIFDFVTSQRDRNSVSQVCKPWYKIESTSRQKVFVGNCYAISPERVIERFPGLKSITLKGKPHFADFNLVPHDWGGFVYPWIEAFARNNMGLEELKLKRMIISDECLELISRSFANFKSLVLVSCEGFSTDGLAAIASNCRFLRELDLQENDVEDHRGHWLSFFPDTCTSLVSLNFACLKGDVNLAALERLVARSPNLRSLRLNHAVPLDILQKILMRAPHLVDLGVGSYVHDPDSETYNKLVTALQKCKSVKSLSGFLEAAPQCLSAFHLICPNLTSLNLSYAPGIHGTELIKLIRHCRKLQRLWILDCIGDEGLEVVASTCKHLQEIRVFPSDPFVGNAAVTEVGLVALSSGCRNLHSILYFCQQMTNAALITVAKNCPNFTRFRLCILDPTKPDADTNQPLDEGFGAIVHSCKGLRRLSMSGLLTDQVFLYIGMYAEQLEMLSIAFAGDTDKGMQYLLNGCKKLRKLEIRDCPFGNAALLMDVGKYETMRSLWMSSCDITLGGCKSLAKKMPRLNVEIINESDQMDITADDGQKVEKMFLYRTLAGRRKDAPEFVWTL

>PtCOI4 POPTR_0020s00280.1

MPNKASTFPEEVLEHVLSFITNDKDRNAVSVVCKSWYEIERWCRKRIFVGNCYAVRPDMVIRRFPELRSVELKGKPHFADFNLVPDGWGGYFYPWIAALATAYPWLEEIRLKRMVISDESLEFIAKSFKNFKVLVLSSCEGFSTDGLSAIAADCRNLRELDLRESEVDDPSGQWLNSFPDSFTSLVSLNISCLGAELSFSALERLVGQCPDLKNLQLNHAVPVERLAKLIRQAPQLVELGTGEFSAKLQPEIFSNLAGAFSVCKELRSLSGFWDVNPAYLPAVYPVCSGLTSLNLRYANIQSADLIKLVSQCSNLQRLWVLDYIEDVGLEALAACCKDLTELRVFPSDPFAAEPNVSLTERGLVSVSEGCPKLQSVLYFCRQMTNAALVTVAKNRPSMTCFRLCIIEPQAPDYQTLQPLDLGFGAIVENYKDLRRLSLSGLLTDRVFEYIGTYGKKIEMLSVAFAGDSDLGLHHVLSGCERLCKLEIRDCSFGDKALLANAAKLETMRSLWMSSCSVSFGACKLLGQKMPRLNVEVIDERGPPESRPESCPVEKLYIYRTIAGPRLDMPGFVRTMDADSVSRFC

>SbCOI1 Sb09g022040.1

MGGEAPEPRRLTRALSIGGGDGGWVPEEMLHLVMGFVEDPRDREAASLVCRRWHRVDALSRKHVTVPFCYAVSPARLLARFPRLESLAIKGKPRAAMYGLIPDDWGAYARPWVAELAAPLECLKALHLRRMVVTDDDLAELVRARGHMLQELKLDKCTGFSTDGLRLVARSCRSLRTLFLEECQINDKGSEWIHDLADGCPVLTTLNFHMTELQVMPADLEFLARSCKSLISLKISDCDVSDLIGFFQFATALEEFAGGTFNEQGELTMYGNVRFPSRLCSLGLTFMGTNEMPIIFPFSAILKKLDLQYTVLTTEDHCQLIAKCPNLLVLAVRNVIGDRGLGVVADTCKKLQRLRIERGDDEGGVQEEQGGVSQVGLTAIAVGCRELEYIAAYVSDITNGALESIGTFCKKLYDFRLVLLDREERITELPLDNGVRALLRGCTKLRRFALYLRPGGLSDAGLGYIGQCSGNIQYMLLGNVGETDDGLFSFALGCVNLRKLELRSCCFSERALALAILRMPSLRYVWVQGYKASQTGRDLMLMARPFWNIEFTPPSSENAGRLMEDGEPCVDSHAQILAYHSLAGKRLDCPQSVVPLYPA

>SbCOI2 Sb03g040150.1

MGGELPEPSRLRRALSFGCGAVPEEALHLVFGYVDDPRDREAASLVCRRWHRIDALSRKHVTVGFCYAVEPARLLARFPRLESLALKGRPRAAMYGLIPEDFGAYAAPWVAQLAAPLDCLKALHLRRMTVTDEDIAVLVRARGYMLQVLKLDKCSGFSTDALRLVARSCRSLRTLFLEECTIADEGSEWLHELAVNNSVLVTLNFYMTDLRVEPADLELLAKNCKSLISLKMSECDLSDLIGFLQTSKGLQEFAGGAFSEVGEYTKYEKVKFPPRLCFLGGLTFMSKNEMQVIFPYSAMLKKLDLQYTCLTTEDHCQLIAKCPNLLVLEVRNVIGDRGLEVVGDTCKKLRRLRIERGDDDPGQEEQGGVSQIGLTAVAVGCRELEYIAAYVSDITNGALESIGTFCKNLYDFRLVLLDKQNKIADLPLDNGVRALLRNCTKLRRFAFYLRPGGLSDVGLGYIGLYSGNIQYMLLGNVGESDNGLIQFAMGCTNLRKLELRSCCFSERALAVAVLQMPLLRYIWVQGYRASQTGQDLMLMARPYWNIEFVPPGPESAYRVMADGQPCVDTHAQVLAYYSLAGRRPDCPQWLVTLHPA

>SbCOI3 Sb01g040110.1

MGGEAEGGERRLGRVLSFGIPDTALGLVMGYVEDPWDRDAISLVCRHWCRVDALSRKHVTVAMAYSTTPERLFRRFPCLESLKLKAKPRAAMFNLISEDWGGSASPWIQQLSATFHFLKKLHLRRMIVCDDDINILVRAKAHMLVALKLDRCSGFSTASIALIARSCKKLETLFLEESTIDERDNDEWIRELATSNSVLETLNFFLTDLRASPEYLTLLVRNCQRLKTLKISECFMPDLVSLFRTAQTLQEFAGGSFEEQGQPVASRNYENYYFPPSLHRLSLLYMGTNEMQILFPYAAALKKLDLQFTFLSTEEHCQIVQRCPNLETLEVRDVIGDRGLQVVAQTCKKLQRLRVERGDDDQGGLEDEQGRISQVGLMAIAQGCPELTYWAIHVSDITNAALEAVGTCSKNLNDFRLVLLDREAHITELPLDNGVRALLRGCTKLRRFAFYVRPGALSDVGLGYVGEFSKSIRYMLLGNVGESDNGIIQLSKGCPSLQKLELRGCFFSEHALAMAALELKSLRYLWVQGFRTSPTGTDLMAMVRPFWNIEYIVPDQDEPCPEHQKQILAYYSLAGRRTDCPPSVTLLYPAF

>SbCOI4 Sb04g033850.1

MSTPHSSSTSSVPHRASPPQIPPSLTLAPTASSASPSSSSASGMRDAAEDDSDSPPSQMSEDDPGGGGDRWEPDLRGGNNGGGGRWAPPDQVLENVLETVLEFLTAARDRNAASLVCRSWYRAEAQTRRELFIGNCYAVSPRRAVERFGGLRAVVLKGKPRFADFSLVPYGWGAYVSPWVAALGPAYPRLERICLKRMTVSDDDLALIAKSFPLFRELSLVCCDGFSTVGLAVIVERCRHLRVLDLIEDYLEDEEDELVDWISKFSESNTSLESLVFDCVSVPFNFEALEALVARSPALRRLRVNHHVSVEQLRRLMARAPQLTHFGTGAFRSEGAPDGGLAVTELATSFAAARSLVCLSGFREVDPQYLPAIYPVCAKLTSLNFSFASLTAAELKPVIRNCTNLRTFWVLDTVGDEGLRAVADTCSDLRELRVFPLDASEDSEGSVSDVGLQAISEGCRKLESILYFCQRMTNEAVIAMSKNCPELVAFRLCIMGRHRPDRVTGDPMDEGFGAIVMNCKKLTRLSVSGLLTDKAFAYIGKYGKLIKTLSVAFAGNSDMSLQYVFEGCTKLQKLEVRDSPFTDRGLLSGLNYFYNMRFLWMNSCRLTMRGCKDVAQQMQNLVVEVIKDHSEDEGEAEIVDKLYLYRSLAGPRNDAPPFVTLL

>SbCOI5 Sb06g014420.1

MTYFPEEVVEHIFSFLPSHSDRNTVSLVCKVWYEVERLSRRAVFVGNCYAVRPERVVLRFPNVKALTVKGKPHFADFNLVPPDWGGYAGPWIEAAARSCVGLEELRMKRMVVSDENLELLARSFPRFKVLVLISCEGFSTDGLAAVASHCKLLRELDLQENDVEDRGPRWLSFFPDSCTSLVSLNFACIKGEVNSGALERLVARSPNLRSLRLNRSVSVDTLSKILARTPNLEDLGTGNLTDEFQAESYARLTSALEKCKMLRSLSGFWDASPICVPYIYPLCHQLTGLNLSYTPTLDYSDLTKMVSRCVKLQRLWVLDCISDKGLQVVASSCKDLQELRVFPSDFYVAGASAVTEEGLVAISSGCPKLSSLLYFCHQMTNEALITVAKNCPNFIRFRLCILEPKKPDAMTGQPLDEGFGAIVRECKGLRRLSMSGLLTDRVFMYIGKYAKYLEMLSIAFAGDSDKGMMDVMNGCKNLRKLEIRDSPFGDVALLGNVAKYETMRSLWMSSCDVTLKGCQVLASKMPMLNVEIMNELDGSSEMENHTDLSKVDKLYVYRTTAGARDDAPNFVKIL

>SbCOI6 Sb05g018860.1

MAYFPEEVVEYILGYVTSHRDRNAASLVCRVWYDIERRGRRSVLVSNCYAVHPERVHMRFPNMRALSVKGKPHFADFNLVPAGWGASAEPWVDACARACPGLEELRLKRMVVTDECLKLLSCSFTNFESLVLVCCEGFSTAGLANIATNCRFLKELDLQESCVKHQGHQWINCFPKPSTSLECLNFSCLTGEVNAVALEELVARSPNLKSLRLNPSVPIDVLPRILSHTPMLEDLGTGSFVLGNNAGAYISLYRALGKCTLLKSLSGFWDAPGLYVRGMLLPICRTRALTCLNLSYAPLIQSDQLISIVRQCTRLHVLWVLDHIGDEGLKVLSYSCPDLQELRVYPSDPNAAARTSVTEEGLAAISFCRKLECVLFFCDRMTNTALITIAKYCPLLTSFRLCILEPRSADAVTGQPLDEGFGAIVQSCKGLRRFAMSGLLTDSVFLYIGMYAEKLEMLSVAFAGDTDDGMVYVLNGCKNLKKLEIRDSPFGDAALLAGAHRYESMRSLWMSSCEITLGACKTLAAAMPNINVEVISEAGASVGATDDGISNARKVDKLYLYRTIAGPRSDTPGFVSIL

>SbCOI7 Sb01g044720.1

MSEEDEDQPPPKRPTSASPSPTADQVLDNVLETVLQFLDAPRDRSAASLVCRSWHRAESATRDSVAVRNLLAASATRTARRFPNARSLLLKGRPRFADFNLLPHGWDASAFRPWAAAVAAGSFPALASLYLKRIPVTDADLDLLSRSLPASFRDLTLHLCDGFTSRGLASIASHCSGLRVLDVVECDMAEEQEGVVDWVAAFPPEPTNLESLSFECYEPPVDFDALEALVARSPLLNRLGVNMHVSLGQLRRLMALAPRLSHLGTGSFRPADGGEEGAGFGEVFSAFVSAGRARTLVSLSGFRDLAQEYLPTIAVVCAHLKSLDLSYTAVTPNQILMFIGQCYNLETLWVLDSVRDEGLESVGMSCKKLQSLRVLPLNAREDADELVSEVGLTAISRGCPALRSILYFCQTMTNAAVIAMSRNCPELKVFRLCIMGRHQPDHATGEPMDEGFGAIVQNCSKLTRLSTSGQLTDRAFEYIGRYGKSLRTLSVAFAGNSDVALQYILQGCSKLEKLEIRDCPFGDAGLLSGMHHFYNMRFVWMSGCNLTLQGCKEVAQGLPRMVVELINGQPDEKERNESVDILYMYRSLDGPREDVPPFVKIL

>SbCOI8 Sb09g003870.1

MGRGGGSRAAPAATAPPWHVLPDEVWEHAFSFLPADSDRGAAAAACRSWLRAERRSRRRLAVANCYAASPQDAVDRFPAVRAVEVKGKPHFADFGLVPPAWGAAAAPWVAAAADGWPLLEEISFKRMVVTDDCLEMIAASFRNFQVLRLVSCEGFSTAGLAAIAAGCRNLRELDLQENEIEDCSIHWLSLFPESFTSLVTLNFSCLEGDVNITVLERLVTRCCNLKTLKLNNAIPLDKLANLLRKAPQIVELGTGRFSADYHPDLFSKLEAAFAGCKSLRRLSGAWDAVPEYLPAFYCVCEGLTSLNLSYATVRGPELIKFISRCKNLQLLWVMDLIEDHGLAVVASSCNKLQELRVFPSAPFDAAEQVSLTERGLVDVSASCPMLESVLYFCRRMTNEALITIAKNRPNFTCFRLCIIEPHTPDYTTHQPLDAGFSAIVESCKGLRRLSVSGLLTDSLFKSIGAHADRLEMLSIAFAGNSDLGLHYILSGCKSLKKLEIRDCPFGDKPLLANAAKLETMRSLWMSTCSLTLGACRQLARKMPRLSVEVMNDPRRGCPLDSLTDESPVETLYVYRTIAGPRSDTPACVQIV

>SmCOI1 EFJ04886

MVESTRRRVPDDSRGLLCCIDDVLEKIFGYIKKPVERNAISAVCKRFHELEARTRHHVLVYNMYAVNPMKLFERFPSVRSITIKGNPRLVDFDILPRDWAGHAGPWIAAIKAHPQLNRFRIKRMTITDSQIEELCAACGPNLKIMQFDKCSGFSTQGLQALAKFCKNLTHLGLAQSMIDSTSDTKWLKDLVNSCPALEYLDLSLIEMGDVDEAVLVKLAERCKLLKLWESETQNSERFLPVLQKCSSNLSDLGIERINSNSETSLLAKCTALEGLSGIFDLVDDGMHAFMSVSSRLTRLDLSYSNLTEVEIAEVLRACPNLQYLRVLDLAGDHGLQALGNSCKDLHRLVVESPSAIDGGVVTHAGLMAVAQGCRNLQKLIFYPSFITNEAFYALAYNCPNLMDVRICLIQSSSTGENMPWECLDEGVTALVRECRSLYRLTLCFDVQADVEFLTDAGVAAIGEYGKKIRVLTLVHCGSSDMGLVPVLRGCNKLQRLEIRKCRFGDESMQEIALNSELHLKHLFVQGCEVTIDGLSSLAYRAKHTNSRFYVEVIGCKDGRCLEEHRYSCTDESCENHHAFSLSCSHWQILAYHSLTEPRDDTPWFIHRFDSILSEASSLPFTRAEPGGALQLMPESSSGGIDLDLSTGGIDLNEDSSEPLQLMPESSGSGGGGGSGGIDLNEPAEESCSSFPGFERKEVFHFPDILERVAAEPTLHLGAPLVLQASLL

>SmCOI2 EFJ07502

MEERSNNKKKKKSTVLWLCDELLERILALIADPCDRAAVSEVNRQWYRVEARTRSRLVVKCSYAVHPWRLAQRFTGLASVTIKGRPRIYDWGLLGDDWGGAADTWIRVLVACCPSLAAIHLRRFDVPDSAIAAIATAAFASSLQVLKLDRCSGFSTRGLLEIARHCKNLRVLSLDESIVDGGGEQWLRALADTATKLEVLSFSLTGIEVRGLDDVAAIVSRNKRLASLRLDEVRTTNDAISRARGILRDAASLQEMLLLYRSVDESSIIEKLELPKTVTSLAGDISIPLDCGLASRLLKLDLMLTTLDSSQLSLLHQTFQACPNLEELKVRNSIGDEGVEAIAKHCRKLKRIRIENLEDNHHSVSQRGLITLASSCPHLRTVAIYASDVSNAAFAAFGHCCRDLYDFRIAVLDSPTPLTDTPLDAGVKSLLQGCRGLRKLALYLKRGGLSDHGLAEMGVLAGNLKWLLLGCAGYSDAGFVGLAAGCARLTKLELRHCPFSEAGMAAGVARMERLRYVWSQGYREVDARELLALGPAWNIEYMPSRDAAVTQFVAYRSLLGPRMDCPPRVMQLVG

>SmCOI3 EFJ09817

MKGHDLLNDVLPDEALIHILSYLDVPSDRGSCSLVCKRWWQLESETRHSIRIGASGNPDACVTAVVRRFTGLRDVSFDERFGFSLIQNGDATSRRGRKRRRGTDELSPLLTESLWSSLSDSGLMLLGQGCPRLEKLTLVWCSAISSTGFKSLAENCCGLKNLELQGCYVGDDGLKAIGQFCKLEDLNLRFCDGVTDLGLMAIATGCAKSLKALIISVCPRVTDATLAAVGKNCSLLERLTLDSEGFKSDGVQAVARGCPRLKYLRMLCVNVEDEALDSVGRYCRSLETLALHSFQKFDKGFLAIGHGCKQLTSLTLSDCYFLTDTTLAAIASGCTELSSLEINGCHNISTSGVRAVGRSCRKLTEVVLKYCQKIGDDGLSEIGRGCKLLQALILVDCSAIGDSSIRSIAGGCPGLKRLHIRRCYKIGDKAIVAVGQHCERLTDLSMRFCDRVGDDGLAAIGAGCPELKHLNVSGCHRVGDAGISAIAKGCPELIHLDVSVCQSVGDEGLAALAGGCRSLREIILSHCRSITDAGLGFLVASCTKLEACHMVYCPYVTAAGVATVVTGCLSIKKVLVEKWKVTPRTRRRAASILTELCMDL

>SmCOI4 EFJ14966

MGERSCCSRTRQRNSRDGDASIIDQVCEHPLLLESVLSIIFGMVDSPAERRAMSEVCRQWHAMDRETRKHVYVAFVYSVSPATLTRRFPNLRSLKLKAKPRAYEFDLLPHNWGGHVHPWLENIGPAYPQLSALHLRRMEVRDQDLSAVATAYAASLETLKLDFCSGFSTTGLRAITGSCKCLKVLYVENSYVSDEGGQWLNELALHNRVLEVLDFQLAIGISKVNVEDVRTIIEKCPNLTSLKLVEGEDGLGDGLRKALASSTSLRELGIFLTAQEEDDQEEIDQGTSSTGQQTMRALLPRNLTSISGDIPLPLYTSVAAQLLKLDLMTTTSIEAEQHHALLRCCTRLQNLQVRTVIGDEGLAIVGECCKDLRKARIEDHNDEGTSVSHTGLMALARGCSKLEKLAIYVADMSNQALAAVGSGCPDLRDFRLILTEANDLSSMTELPLDAGFDELMRGCHRLSRLCIYVRPGALSDHGLVRIGHRGANLKALLLGCCGDSDAGFLAIARGCKRLQRLEIRDCPFTDDGLLRGVGCMEDLKLMWIQGFRMDDYGKLDVLGGEKYRNVECTRRDPIQCLIYRSLAGPRLDCPEEVVSPQGNGMLRHNWLTPSGGLVVG

>SmCOI5 EFJ15060

MADRKMSKNSGDSVIDLLDDNILLQILERLEDRFDRQAWCLSCKHFLRLEASTRNRIQLMRHEVLEGILHRYTRLEHLDLSHCIQLVDENLALVGQIAGNRLASINLSRVGGFTSAGLGLLARSCCASLTDVDLSYCSNLKDSDVLALAQISNLQALRLTGCHSITDIGLGCLAAGCKMLKLLTLKGCLGITDIGIALVAVNCKQLRTLDLSYTEVTDEGLASIATLHSLEVLNLVSCNNVDDGGLRSLKRSCRSLLKLDVSRCSNVSDAGLAALATSHLSLEQLTLSYCSIITDDLLATFQKFDHLQSIVLDGCEIARNGLPFIARGCKQLKELSLSKCRGVTDRGIAAVAQGCTALHKLNLTCCRELTDASLCRISKDCKGLESLKMESCSLITEDGLCGLGEGCPRLEELDFTECNMSDTGLKYISKCTALRSLKLGFCSTITDKGVAHIGARCCNLRELDFYRSKGIGDAGVAAIASGCPKLKLLDLSYCSKITDCSLQSLSQLRELQRVELRGCVLVSSTGLAVMASGCKRLTEIDIKRCSQIGNAGVSALSFFCPGLRMMNISYCPISNAGLLSLPRLSCLQSVRLVHLKNVTVDCFVTVLQNCKSLKNVKLPSYLRTLLPPGIAEEMESRGCRIRWMDKALEEDDVLEPH

>SmCOI6 EFJ20341

LPEECLGLIFDRLDTRGRNVASLVCRRWLVAEANSRKILSLSAPLSLPVSCLESSLMRFTVLSKLGLKCERGVPSITDEGLVLIATHCRRLSKLKLKNCTGLQDDGLVAFAAAVCRASFRSFSCCSCGFGSRGLNAIIKNCVALEDLSVKRLRMGGEPGQLVEGPSKLKRLSIKNILDGGHAFTPLIASSKHLHTLIIFKATGQWDKLLELSVEGLSELTELRIEKLHLGDQGLVALAKCRKLQVLFLARTPECSNTGLSAIANGCRSLRKLHVDGCFTGRIGDKGLLTVGERCPELKELVLIGVSVTSNSLGTVFTNCMGLERLAVWNSETFGDGELACIGSKCQALRKLCIKCCPISDQGLEALASGCPSLTKVKIKRCRSVSASGAASLMMAHDGLVVTLEADQ

>SmCOI7 EFJ24298

MSMELEDVRFNHLFQKSFLHENSYAFLSKEELCSQLIRGLQSLQTGRRRKLSEIGNTEIYNAPKRFLPTPPPTAVPAAKKYQPKDLINALPDELLVEVFRYVAAPADRYACASVCTRWLMLQSHLHSSEIKDDEQELSLGSGDELKRSLEGKRATDVRLAVVALGTQSRGGLGKLIIKGGPRQKLSKAVSNVGMSSVGICCGNLKVLSVWDCPNIDDVGFSWIGKGCPQLKVLNIMNCPGFGDAALRAIAAGCPLLSSLTLDGCDKVGDEGLQAVGKRCSQLSCLSVSRCNKVGDVGVTAVVSSCKVLKAMKLEKLSINDEGLVAVGEHGGSLQKLKLLQLEKISSEGFFLFGKSSGMGQLKHLQISACPGLTDSLLDSVGKTSKEIKFLSLANCTSLDESKLLTFVKDCTFLEGLHLEKCAFTASAATMTTTLLSSGSRSLKVLGIVNCTGVGAGLLASLSGSGSSCLLELNVSGLSALSDESLVPFLSASGSGLTSLNLSGCTRLTNRALAAVASFCPSLGLLTLDGCASVTDQGIRYVAQGPQAVQELSLAGCDVTDDGMVALVLAKGSSLKTLSLAGCGRVTDRSLLAMKTACNTLEALNVKDCKGLSRAKLEWFEAGLWRCHLCY

>SmCOI8 EFJ29076

MELEQEKEEDEREEEEEQGRLTTFPDEVLENVLKFVTGHKDRNAVSVVCKAWYKAEGWNREAVFIGNCYAVSPDILTRRFPRLKSMTLKGKPRFADFSLVPPNWGAFFHPWMPVIVESYPWLEALRLKRMTVSDESLFMISQLLPNFRALNLVNCDGFSTEGIAAITSHCRYLQELDLQECLVDDRGGEWLSYFPESCNTLVTLNFSCLESDVNFECLEKLVSRCRSLKKLNLNKGVTLEQLLRLLVKAPQLTDLGTGTYSQMQNWSQYVELRTALSNCKDLRHLSGFWMVEPIFIPLIYPLAQNLLSLNLSYATIRATEFAKLIQRCPKLETLWVLDSVEDRGLQTVGETCKNLVELRVFPTDHGGQGSVTEAGLVAVSQGCPNLSSVLYFCKQCTNQAIETVATNCPMLTRFRLCIITPRQRDYITGETMDEGFGAIVKNCKNLSRLAVSGWLSDRAFEYIGHYAKKLETLSVAFAGESDAAMQHVLSGCPRLRKLEIRDSPFGDSALLAGLHQYESMRFLWMSACRVSLAGCGWLAGAMPRLNVEVIREQGDAEGGEGGEKGGGELVMDCSEPVEKLYAYRTLAGCRSDAPSWVITL

>SmCOI9 EFJ30612

MKISRRWIHADDDPDSINSRLPDDLLKIIFSRLGDDQDHASVARVCRQWRDAESATREKITVNFSYAVSPGYVIDRFGQLRALKIKGKPRASDFGLIPVDWGGYGGPWIAALALARARSLFGALASLHFKRMEISDEDLALLAETFRDALQVLKLEKCSGFTSLGLESIARSCRDLRVLSLDESDIEDKGSQWLRELIHSCASLEALNLSMTGLELRDIRLVEEIVSSSKLKSLKLNDLEDPSRNRRLDLRQSSLQELGFCGLIQVSLPSSLSSFSGDLQLAMEPNLASALTSLDLLYTTANHEQHLEIIKGCRNLQVFKANIIGDIGLELLASHCKGLQRIRIENMRQQEQHGFSISNSGMLALAKSCVHLQSFSMYVHDAANSSLEALAESCPGLLDFRLGILETAPDMAEPLDAGVQSLLQRCPSITKLALYLKEGGLTDRGLESIGRLGQQLKWILLGCLSDSDTSDRGLVSLARGCSNLRKLEVRNCPFSDAAIVCGIRGLPLLRYLWFQCYHRVSDRHFALLEPEWRIELMPEFYSVLCYRALVSGSRGDHPPSVRPMLGGGAGGG

>SmCOI10 EFJ31715

MRLEADIWSLNNHLLVKILEKLDEVVDRKSWRLTCKRFYAAGAESQKTMRLFNSELLPRALARHTGIESLDLSSCIKITDEDLALVGELAGTRLRSLGLARMGGFTVAGIVALARNCSALVELDLRCCNSLGDLELAAVCQLGSLRKLDLTGCYMISDAGLGCLAAGCKKLQVVVLKGCVGISDAGLCFLASNCKELTTIDVSYTEITDDGVRCLSNLPSLRVLNLAACSNVGDAGLTRTSTSLLELDLSCCRSVTNVGISFLSKRSLQFLKLGFCSPVKKRSQITGQLLEAVGKLTQIQTLKLAGCEIAGDGLRFVGSCCLQLSDLSLSKCRGVTDSGMASIFHGCKNLRKLDLTCCLDLTEITAYNIARSSAGLVSLKIEACRILTENNIPLLMERCSCLEELDVTDCNIDDAGLECIAKCKFLKTLKLGFCKVSDNGIEHVGRNCSDLIELDLYRSGNVGDAGVASIAAGCRKLRILNLSYCPNITDASIVSISQLSHLQQLEIRGCKRVGLEKKLPEFKNLVELDLKHCGIGDRGMTSIVYCFPNLQQLNLSYCRISNAGLVMLGNLRCLQNVKLVQIGDVSIEVLAAALLSCVCLKKAKLFCNALLNDSINARYQQLEDRGCRIRWMIKPER

>SmCOI11 EFJ32115

MEWMSDELLDSIFSFIDHPMDRRALSEVCKRWYLADARTRKSITVGFSYAIEPSNLSRRFRNIQALKIKGKPRVSEFGMVVKDWGAYCEPWIQELVSQRHPSSATAFASLTSLHFRRMEVSDTALRLLARGFGSSLQVLRLDKCSGFSTAGLEAVARECKSLRVLYLEESVIEDDGSQWLHELAVSNSALEVLNFFLTGLDLSNLSDLAHIIANCKSLTSLKLGEISRGVVDLPADIFIAAKSLKELAVIFARNNISVNLPKTLTSFAGDLLFPLDPLVCSNFRELDLMSTTLTAEEHMQVIQCCPNLEVLKVRNIIGDAGVATLASLCPKLRRIRIENLEDAYGFCSYKGLITLASRCVNLQHVAIYVSDIANSALRAFGTHCPHMLDFRIVLLESTLPVTELPLDSGVRALLQGCRKITRLAIYLRNGGLTDAGLAAIGSLGEHLTWLLLGCVGTSDRGLIDLASGCRSLQKLELRDCPFTEGGIAVSVRLLASLRFLWIQKYRESNPYDLLQMGDWVVEYIVPSSDTTPSQVVAYRSTVGHRSDFPEEVIPLSQIAWAFGGGFHI

>TuCOI1 TRIUR3_17029-P1

MPYFPDEVVEHILGFVSSHRDRDAASLVCHAWYRIEGLTRRSVFISNCYAVRPERVHARFPCLRSLTVKGKPRFADFNLVPAGWGASAEPWVDACARACPGLEELRLKRMVVTDGCLNHLAHSFPNLRSLVLVSCEGFSTDGLATIATNCRFLKELDLQGSQVEFRGRHWFSCFPKPSTSLESLNFACLDGAVSANALESLVARSPNLKSLRLNRAVPPAVLAKILTSAPKLVDLGTGLVAQSNNAGALPSLYSAIQQCSSLNSLSGFWDSPRWITPIIQYICKNLTCLNLSYAPMFRTVDLIGIIRQCQNLRHLWVLDHIGDAGLKVVASSCLELQELRVFPANANVLISTGVTEEGLVAVSSGCRKLNSVLYSCRRMTNSALITVAKNCSRITSFRLHICLHGSVDAVTGQPLDEGFGAIVRSCKGLRRLSMSGLLTDSVFLYIGMYAERLETLSVSFAGDSDDGMIYVLNGCKNLRKLEIRNCPFGNTALLAGMHRSLAAAMPGLNVEVISQADGGTNDAKKKKWNAMLWFHLGVWLEPMKRMKRFHFNVLDDGFAISHGSEFSGGHRQEWRGTGAGTSAWVGLGGGCTGRKKRSGRSCRHAQPVATPAPASTRHHTSCSDPKPFDPLSHAMALADLNFEIGQDFVVEVWRKWGVPINYEEGKDMEEFLLVAEFTRSQIRLTEESLITILLSCFGGRASLFKVQFLRIGRLNSLSPPKKWGFRSSREATSLFLYRISTSYFGAMVVLIPVGNWINTSKKKKMLGRTSFIVTLGSPTSRLCNLQGSI

>TuCOI2 TRIUR3_18749-P1

MTVSDDELALIPKSFPLFKELSLVCCDGFTTRGLAVIAEGCRHLRVLDLTEDYFHEEESEVVDWISKFPESNTSLESLVFDCVSVPFNFEALEALVARSPALRRLRVNDHVSIEQLRRLMARAPHLTHLGTGSFRSEPGPGGALSVSELATSFAASRSLVCLSGFLDANAAYLPAIYQVCANLTSLNFSFAGLTDEEFIPVIRHCVNLRTLWVLDTVGDEGLRAVAETCSNLRELRVFPLDATEDSEGSVSDIGLQAISEGCRKLESILYFCQRMTNAAVIAMSENCPDLLVFRLCIMGRHRPDRITGAPMDEGFGAIVMNCKKLTRLSVSGLLTDKAFAYIGRHGKLIKTLSVAFAGNSDMSLQHVFEGCTRLQKLEVRDSPFGDKGLLSGLNYFYNMRFFWMNSCRLTVKGCGDVAQQMPNLVVEVMKENEGEMDTVDKLYLYRSLAGPREDAPSFVNIL

>TuCOI3 TRIUR3_21512-P1

MTDMGLGCIAVGCPDLRDLTLNWCLGITDLGVQLLALKCKKLRTLNLSYTMISKDCLPAIMKLPNLEVLALVGCVGIDDDALSGLENECSKSLRVLDLSTCRNVTHTGVSSVVKAVPNLLELNLSYCCNVTPSMGKCFQMLPKLQTLKLEGCKFMADGLKYIGISCVSLRELSLSKCSGVTDTDLSFVVSRLKNLLKLDITCNRNITDVSLATITSSCPSLVSLRMESCSHFSSEGLRLIGKRCCHLEELDITDSDLDDEGLKALSGCRKLSSLKIGICMRISDEGLIHIGKSCPELRDIDLYRSGGISDEGVTQIAQGCPMLESINLSYCTEITDVSLVSLSKCAKLNTLEIRGCPSVSSAGLSEIAIGCRLLAKLDVKKCFAINDVGMLFLSQFSHSLRQINLSYCSVTDIGLLSLSSICGLQNMTIVHLAGITPNGLMAALMVSGGLTRVKLHAAFRSMMPPHMLKVVEARGCAFQWIDKPFKVFYLPPYKPRSSKSDATYGNNSLEMCLYDEIMRNACYRRLDCQPYMNMMV

>TuCOI4 TRIUR3_22304-P1

MSLPDVVYSCGSCGYALNLSSSNRSTSDVGSSYQKSLKKGLISFTSIDLSRFTQVDEISCFPFLTWRSYRPKTKLLCRKCGSSIGYGYAYKEHAQCLPEMLARSPYLQLISLAGLNELSDSALYEVGVSGTSLQSFSLYSCSGITDDGLAQVSIGCPNLVIVELYRCLNITDLGLESLSQGCHSLKSLNLGYCTAISDRGISSIFRNCRNICALIISYCRGVSGVGFRGCPSTLSYLEAESCMLSSEGMLDISSGGGLQYINLYNLRSSAGLDCLGGVGSMKKLRVLNLRMCRYLTDDSVVAIASGCPLIEEWSLAVCHGVRLPGWSAIGLNCNKLRILHVNRCRNICDQGLHALKDGCVRLEVLHIHGCGKITNNGLALFSIARPSVKQMVDEAMSIGPSIEDLFRLQ

>TuCOI5 TRIUR3_23066-P1

MSFGIPDVALGLVMGCVEDPWDRDAISLVCRHWCKVDALSRKHVTVAMAYSTTPDRLFRRFPCLESLKLKAKPRASMFNLIPEDWGGSASPWIRELSASFHFLKVLHLRRMIVSDDDVAVLVRAKAHMLVSLKLDRCSGFSTSSLALLARCCKKLETLFLEESSVAEKENDEWLRELATSNTVLETLNFFLTDLRASPAHLLLLVRNCRRLKTLKISDCFMSDLVDLFRTAETLQDFAGGSFDDQDQGGNYANYYFPPSVQHLSLLYMGTNEMQILFPYGATLKKLDLQFTFLTTEDHCQLVQRCPNLEVLEVRDVIGDRGLEVIAQTCKKLQRLRVERGDDDQGGLEDEQGRVTQVGLMAVAEGCPDLEYWAVHVSDITNAALEAIGAFSKNLNDFRLVLLDREVHITELPLDNGVRALLRGCTKLRRFAFYVRPGALSDIGLSYVGEFSKTVRYMLLGNAGGSDDGLLAFARGCPSLQKLELRSCCFSERALAVAALQLKSLRYLWVQGYKASPTGTDLMAMVRPFWNIEFIAPNQDEPCPEGQAQILAYYSLAGARTDCPQSVIPLHPSVGS

>TuCOI6 TRIUR3_23956-P1

MTVTDDDLGLVARSFPGFKELSLVCCDGFSTLGLAVIAERCRHLRVLDLIEDYVDQEDEAVDWISKFPVSNTSLESLMFDCVAVPFNFEALEALVARSPSLRRLRVNHYVSVEQLRRLMARAPQLTHLGTGAFRPEAPQGGGMSVSELAPSFAASTSIVCLSGFQEVNPEYLPAIYPVCGNLTSLNVSFASLTAEDLTPVIRQCHKLQTFWVLDTVGDEGLRAVAETCSDLRELRVFPLDATEDSDGSVSDVGLQAISEGCRKLESILYFCQRMTNAAVVAMSNNCPDLVVFRLCIMGRHRPDRITGEPMDDGFGAIVKNCKKLTRLSVSGLLTDTAFAYIGQYGKLIKTLSLAFSGNSDLSLQFLFEGCTRLQKLEVRDSPFSDRGLLCGLDYFYNMRFLWMNSCRLTMRGCREVAQRMPNLVVEVMEEQNEDKVETETVDKLYLYRSLAGPRGDAPPLVKIL

>TuCOI7 TRIUR3_24621-P1

MAAMRADRQILEEHLDFEATVDTATWLCTLKYSSCCLNIPTGRASPTGHHCCNGSIIQARICQNRGCGESRGLLGGEFNKPILMKLANTFNVVTLVAATYHVCRFTVIEWKVLGGGIGTGGRNPPSHFGAVRSGLLGGLRVLNRCWLPVLAQQIWLEPDLPKPALVVCSLCLVEQGCSFHLLISSALFSSFDNLETASKQIPSCSSSNKVVLGEGTMTYFPEEVVEHIFSFLPAQCDRNTVSLVCKVWYEIERLSRRTVFVGNCYAVRPERVVLRFPNVRALTVKGKPHFADFNLVPPDWGGYAGPWIEAAARGCVGLEELRMKRMVVSDESLELLAKSFPRFRALVLISCEGFSTDGLAAIASHCKLLRELDLQENEVDDRGPRWLSCFPDSCTSLVSLNFACIKGEVNAGSLERLVARSPSLRSLRLNRSVSVDTLSKILMRAPNLEDLGTGNLTDDFQAESYLRLTLALEKCKLLRSLSGFWDASPLCLPFIYPVCGQLTGLNLSYAPTLDSSDLTKMISHCVKLQRLWVLDCIADKGLQVVASSCKDLQELRVFPSDFYIAGYSPVTEEGLVAISLGCQKLSSLLYFCHQMTNAALLTIAKNCPNFTRFRLCILEPGKPDAMTNQPLDEGFGAIVRECKGLRRLSISGLLTDKVFMYIGKFAKQLEMLSIAFAGDSDAGMMHVMEGCNNLRKLEIRDSPFGDAALLENVTKYETMRSLWMSSCNVTEKGCQILASKMPMLNVEVINEVDESNEMDENHGIPKVDKLYVYRTTAGARDDAPNFVKIL

>TuCOI8 TRIUR3_31653-P1

MVVTDDDLAALVRARGHMLQELKLDKCSGFSTDALRLVARSCRSLRTLFLEECTITDNGTEWLHDLAANNPVLVTLNFYLTYLRVEPADLELLAKNCKSLISLKISDCDLSDLIGFFQIATSLQEFAGAEISEQKYGNVKLPSKLCSFGLTFMGTNEMHIIFPFSAVLKKLDLLYSFLTTEDHCQLIAKCPNLLVLAVMAVLVHYYIMKVRNVIGDRGLGVVGDTCKKLQRLRVERGEDDPGMQEEEGGVSQVGLTAIAVGCRELENIAAYVSDITNGALESIGTFCKNLHDFRLVLLDRQETITELPLDNGARALLRGCTKLRRFALYLRPGGLSDVGLGYIGQHSGTIQYMLLGNVGQTDGGLISFAAGCRNLRKLELRSCCFSERALALAIRQMPSLRYVWVQGYRASQTGRDLMLMARPFWNIEFTPPSTETAGRVMEDGEPCVDRQAQVLAYYSLSGKRSDYPQSVVPLYPV

>TuCOI9 TRIUR3_32567-P1

MAECRSAGAASDLGQSAGVGEGGAGWARTGGAERENGGKPHFADFGLVPPAWGAEAAPWVAAAAEGWPLLEELSFKRMVVTDECLEMIASSFRNFQVLRLVSCEGFSTAGLAAITEGCRNLRELDLQENYIEDCSSHWLSYFPESFTSLETLNFSCLEGEVNFAVLERLVSRCRNLKTLKLNNAIPLDKVASLLRKAPQLVELGTGKFSAEYHSDLFAKLEAAFAGCKSLRRLSGAWDAVPDYLPAFYCVCEGLTSLNLSYATVRGPELIKFISRCKNLQQLWVMDLIEDHGLAVVAGSCSKLQELRVFPSDPFGAGQVLLTERGLVDVSASCPMLESVLYFCGQMTNEALITIAKNRPNFTCFRLCILEPRTPDYVTRESLDAGFSAIVESCKGLRRLSVSGLLTDLVFKSIGANGNCLEMLSIAFAGNSDLGLHYILSGCKSLKKLEIRDCPFGNKPLLANAAKLETMRSLWMSSCSLTLGACRQLAEKMPRLTVEIMNDPGRICPVESLPDDSPVETLYVYRTIAGPRSDTPDYVQIV

>TuCOI10 TRIUR3_34918-P1

MAGEDVDPKSSASAAAVPGLRVLDVVDCELNEEEDDEVSDWVAAFPRGHTDLESLSFECFTPQVPFAALEALVARSPRLRRLRVNQHVSLGQLRRLMALTPRLTHLGTGSFRPGDGAEDEGLDFGQMLTAFASAGRANSLVSLSGFRDLAPEYLPTIATVAANLTSMDLSYAPVNPDQVLLFIGQCRSLETLWVLDSVRDEGLQAVAMYCKKLQVLRVLPLDAHEDADELVSEVGLTAISEGCRDLRSILYFCQRMTNAAVITMSQNCPEMTNCPEMKVFRLCIMGRHRPDHVTGEPMDEGFGAIVRNCSKLTRLSTSGHLTDRAFEYIGNYGSSLRTLSVAFAGDSDLALQHILQGCSKLEKLEIRDCPFGDAGLLSGMHHFYNMRFVWMSGCSLTLQGCEEVARQLPRMVVELINSQPEIEKTDGVDILYMYRSLEGPREDVPPFVKIL

>TuCOI11 TRIUR3_35106-P1

MYGLISDDWGAYAAPWVARLAAPLECLKALHLRRMTVTDDDVAALIRSRGHMLQELKLDKCSGFSPAALRLVARSCRSLRTLFLEECVITDEGGEWLHELAVNNSVLVTLNFYMTELKVVPADLELLAKNCKSLLSLKISECDLSDLIGFFEAANALQDFAGGSFNEVGELTKYEKVKFPPRVCFLGLTFMGKNEMPVIFPFSASLKKLDLQYTFLTTEDHCQLISKCPNLFVLEVRNVIGDRGLEVVGDTCKKLRRLRIERGDDDPGLQEEQGGVSQLGLTAVAVGCRDLEYIAAYVSDITNGALESIGTFCKNLYDFRLVLLDRQKQVTDLPLDNGVRALLRSCTKLRRFALYLRPGGLSDTGLDYIGQYSGNIQYMLLGNVGESDHGLIRFAIGCTNLRKLELRSCCFSEQALSLAVLHMPSLRYIWVQGYKASPAGLELLLMARRFWNIEFTPPSPEGLFRMTLEGEPCVDKQAQVLAYYSLAGQRQDCPDWVTPLHPAA

>ZmaCOI1 ZM01G10730

MGGEAEGGERRLGRVLSFGIPDTALGLVMGYVEDPWDRDAISLVCRHWCRVDALSRKHVTVAMAYSTTPERLFGRFPCLESLKLKAKPRAAMFNLISDDWGGSASPWIRQLSATFHSLKKLHLRRMIVSNDDINTLVRAKAHMLVSLKLDRCSGFSTPSIALIARSCRKLETLFLEESMIDEKENDEWIRELATSNSVLETLNFFQTDLRASPEYLTLLVRNCQRLKTLKISECFMPDLVSLFRTAQTLQEFAGGSFEDQGQPVAGRNYENYYFPPLLHRLSLLYMGTNEMQILFPYAAALKKLDLQFTFLSTEDHCQIVQRCPNLETLEVRDVIGDRGLQVVAETCKKLQRLRVERGDDDQGGLEDEQGRISQVGVMAIAQGCPELTYWAIYVSDITNAALEAVGTCSRNLNDFRLVLLDREAHITELPLDNGVRALLRGCTKLRRFAFYVRPGVLSDVGLGYVGEFSKSIRYMLLGNVGESDNGIIQLSKGCPSLQKLELRGCLFSEHALAMAALELKSLRYLWVQGFRSSPTGTDLMAMVRPFWNIEYILPDQDEPCPEYKKQILAYYSLAGRRTDCPPSVTPLYPAV

>ZmaCOI2 ZM03G26700

MGGEVPEPRRLSRALSFGCGGVPEEALHLVFGYMDDPRDREAASLVCRLWHRIDALSRKHVTVGFCYAVEPARLLARFPRLESLALKGRPRAAMYGLIPEDFGAYAAPWVAELAAPLDCLKALHLRRMTVTDEDIAVLVRARGHMLQVLKLDKCSGFSTDALCLVARSCRSLRTLFLEECIIEDEGSEWLHELAVNNSVLVTLNFYMTELKVEPADLELLAKNCKSLISLKMGDCDLSDLIGFFQTSKALQEFAGGAFFEVGEYTKYEKVIFPPRLCFLGGLTFMGKNEMPVIFPYSTMLKKLDLQFTFLTTEDHCQLIAKCPNLSVLEVRNVIGDRGLEVVAATCKKLRRLRIERGDDDPGQEEQGGVSQIGLTAVAVGCRELEYIAAYVSDITNGALESIGTFCKNLYDFRLVLLDKQKKITDLPLDNGVRALLRNCVKLRRFAFYLRPGGLSDVGLGYIGLYSGNIQYMLLGNVGESDNGLIQFATGCTNLRKLELRGCCFSERALAVAVLQMPSLRYIWVQGYRASRTGQDLMLMARPYWNIEFAPPIPESAYRVMADGQPCVDTHAQVLAYYSLAGRRPDCPQWLVTLHPASL

>ZmaCOI3 ZM06G24870

MGGEAPEPRRLTRALSIGGGGGGWVPDEMLQLVLGFVEDPRDREALSLVCRRWHRIDALSRKHVTVPFCYAVSPARLLARFPRLESLAVKGKPRAAMYGLIPDDWGAYARPWVTELAAPFECLKALHLRRMVVTDDDLAELIRARGHMLQELKLDKCTGFSTDGLRLVARSCSSLRTLFLEECQINDKGSEWIRDLAVNCPVLATLNFHMTELEVMPVDLELLAKSCKSLISLKIGDCDLSDLIGFFQSATSLEEFAGGTFNGQGELTKYGDVKFPSRICSLGLTFMGANEMPIIFPFSAILKKLDLQYTFLTTEDHCQLIAKCPNLLVLAVRNVIGDRGLGVVADTCKKLQRLRIERGDDEGGVQEEQGGVSQVGLTAIAVGCRELEYIAAYVSDITNGALESIGTFCKKLYDFRLVLLDREERITDLPLDNGVRALLRGCTMLRRFALYLRPGGLSDAGLGYIGQCSGNIQYMLLGNVGETDDGLISFALGCVNLRKLELRSCCFSERALALAILSMPSLRYVWVQGYKASQTGRDLMLMARPFWNIEFTPPSSQNAGRLIEDGEPCVDSHAQILAYGSLAGKRLDCPQSVVTLYPA

>ZmaCOI4 ZM08G14420

MYGLIPDDWGAYARPWITELAAPLECLKALHLRRMVVTDDDLAELVRARGHMLQELKLDKCTGFSTHGLRLVARSCRSLRTLFLEECQIDDKGSEWIHDLAVCCPVLTTLNFHMTELEVMPADLKLLAKSCKSLISLKISDCDLSDLIEFFQFATALEEFAGGTFNEQGELSKYVNVKFPSRLCSLGLTYMGTNEMPIMFPFSAILKKLDLQYTFLTTEDHCQLIAKCPNLLVLAVRNVIGDRGLGVVADTCKKLQRLRIERGDDEGGVQEEQGGVSQVGLTAIAVGCRELEYIAAYVSDITNGALESIGTFCKKLYDFRLVLLDREERITDLPLDNGVRALLRGCTKLRRFALYLRPGGLSDAGLGYIGQCSGNIQYMLLGNVGETDDGLISFALGCVNLRKLELRSCCFSERALALAILHMPSLRYVWVQGYKASQTGRDLMLMARPFWNIEFTPPNPKNGGWLMEDGEPCVDSHAQILAYHSLAGKRLDCPQSVVPLYPA

CDS sequences:

>TaCOI1-A Traes_1AS_1E22B5174.1

ATGGAGGACCTACAGGAGGCTCTGATGACAGAGATTCTCAAGAGGATCACCACGATAAGTGATCTGAATTCTCTTTCCCTTGTGTCAAAGCAGCTCTGCAAGATAGAGGGGAATCAGAGGGGTGCTATCCGTGTTGGTTCCCGTCTTTGCACTGCTACAGAAGCACTGACATCATTGTGCGCCCGCTTCCCAAATCTGCGAAGAGTGGAAATCGATTACTTTGGTTGGATACCTGGACATGGAAGGCAGTTGGACAACAAAGGCTTTTCTGTGTTTTCATCTCACTATTCCTCACTGATTGACCTAACCTTAAGCTTCTGCTCATGCATCGATGACTCTGGGCTTGCTTGTTTAGCTTATTGCAAGACATTGGTGTCTCTCAGGCTCAACTCCGCACCAAAAATAACGTCAGTTGGGCTTTTCTCGGTTGCAGTTGGTTGCAGAAGTCTATCTGCTCTCCACCTTATTGATTGCGAGAAAATCGACACTGTAGAGTGGCTGGAATACCTTGGTAGGGATGGATCGTTGGAAGAGCTTGTAGTGAAGAATTGCCAAGGAATCAATCATCATGACTTTCTAAAGTTTGGTTCAGGATGGATGAAGCTCCAGAAGTTTGAGTTTGAGAGTAAAAGAGAAAGATATGATCGTCTTCCAGGTGATGTGGTCTATGACTCCTCGTACCATGCTCACAGCACGGATATATATGATTTCTGCTGTGAGAGTTTGAAGGATTTAAGGTTGGCTCATATTAAAACTTGGCCAGAAGTAGGTCTTCGTCTTGTCCTAGGGAAGTGTAAAGCATTGGAGAAGCTTTGCCTTGAGTATGTTCGTGCCCTAAATGACAATGACATTATTGCATTATCTCGGAGCTGCAGCAACCTTAAAAGCATCTCACTTTGGCTCAACCTGCAGCGCTACTCTAGTGATGTCAGCTATTGTGAGACGAGGACGTCATTTACTGATAACAGCCTTTACGCTCTAGCACGAAACTGTCGTATGCTTCAGATGGTAGACCTCAGCTTTATAGGATGTTCCCGTGACTGGCCATCAGAAATAGGATTCACACAAGAGGGTTTTCTGGTGCTCATTCAGTCCTGCCCGATTCGTGTTCTCGTGCTAAACACCGCCAACTTCTTTGATGACGAGGGGATGAAGGCCCTCTCATCCTCACCACATCTGGAGACACTCGAGCTTATATTGTGCCATGCGGTAACTGATGCTGGGATGCACTTCATTGCGCACACCCCATGCTTGAGTAATCTCACACTTCGGTTGTGTCATAACGTTACTGATGTGGGAGTGGCTGAACTGGGACATGCACATAAGTTAGAATCTTTGGTTGTCGAGTATTGTGGTGAGGTCTCTCTGCAAGCTGTGCAGGGTGTTGCCAAGTTAGTTCACTACAGTGACTATTCATATTTCTTTATGAAGAAAATTGGTCTTGGCGCCTATTGA

>TaCOI2-A Traes_2AL_9EC359B65.2

ATGACCTACTTTCCGGAGGAGGTGGTGGAGCACATATTCAGCTTCTTGCCCGCGCAATGTGACCGGAACACCGTTTCACTTGTTTGCAAGGTATGGTATGAGATTGAAAGGCTGAGCCGGCGAACTGTCTTTGTGGGTAACTGCTATGCTGTGCGCCCTGAGCGCGTGGTGCTTCGGTTCCCCAATGTGCGGGCACTGACAGTGAAGGGGAAACCACACTTCGCTGATTTCAACCTTGTGCCACCTGATTGGGGTGGGTACGCCGGACCATGGATCGAGGCAGCAGCCAGGGGCTGCGTGGGTCTTGAGGAGCTGCGGATGAAGCGGATGGTGGTGTCAGATGAGAGCCTGGAGCTGCTTGCCAAATCATTCCCACGATTCAGGGCCCTAGTTCTTATCAGCTGTGAGGGGTTCAGCACCGATGGACTAGCAGCTATTGCAAGTCACTGCAAGCTCCTGAGGGAGTTAGATTTGCAGGAAAATGAGGTGGATGATCGAGGGCCAAGGTGGCTCTCCTGCTTCCCTGATTCCTGCACGTCCCTTGTCTCCTTGAATTTCGCCTGCATCAAAGGGGAGGTTAATGCTGGTTCATTAGAGAGACTTGTTGCTAGGTCCCCAAGTCTTCGGAGTTTGAGGTTGAATCGATCTGTGTCAGTAGATACACTCTCGAAGATATTAATGAGCGCCCCTAATTTGGAGGATCTAGGGACTGGGAACTTGACAGATGACTTCCAAGCTGAATCGTATCTCAGGCTGACCCTTGCATTGGAGAAATGCAAACTGCTGAGGAGTTTATCGGGCTTTTGGGATGCTTCGCCTTTGTGCCTTCCATTCATCTATCCTGTATGTGGGCAACTAACAGGTTTAAACTTGAGCTATGCTCCGACACTTGATTCTTCTGATCTCACCAAAATGATCAGCCACTGTGTGAAACTCCAACGTCTTTGGGTACTGGATTGCATCGCGGATAAGGGCTTGCAAGTGGTGGCCTCCAGTTGCAAGGATCTACAAGAACTCAGGGTATTCCCATCAGACTTCTATATCGCCGGGTATTCCCCAGTAACAGAGGAGGGACTTGTTGCAATATCCTTGGGCTGTCAAAAACTGAGCTCATTGCTATATTTTTGTCATCAAATGACGAATGCTGCACTGCTTACTATAGCTAAGAACTGCCCAAATTTCACGCGGTTCAGACTCTGTATTCTTGAGCCTGGGAAGCCTGATGCCATGACAAACCAACCATTAGATGAAGGTTTTGGTGCTATTGTTCGTGAATGCAAAGGGCTAAGGCGATTGTCAATATCGGGTCTTCTCACCGACAAGGTTTTCATGTATATTGGTAAATTCGCGAAACAACTTGAGATGCTTTCAATAGCATTTGCTGGAGATAGTGATGCGGGAATGATGCATGTTATGGAAGGATGCAATAATCTGAGGAAGCTGGAGATTAGAGATAGCCCATTTGGTGATGCTGCACTCCTGGAGAATGTTACCAAGTATGAGACAATGCGATCCCTTTGGATGTCATCATGCAATGTCACAGAAAAGGGGTGCCAAATCCTTGCATCAAAGATGCCAATGCTTAATGTGGAGGTTATAAATGAGGTAGATGAGAGCAATGAAATGGATGAGAACCATGGAATCCCCAAAGTTGACAAGTTATATGTTTACCGCACAACTGCTGGGGCAAGGGATGATGCGCCAAATTTTGTTAAAATCCTATAG

>TaCOI2-B Traes_2BL_370AE211F.1

ATGACCTACTTTCCCGAGGAGGTGGTGGAGCACATATTCAGCTTCTTGCCTGCGCAGTGTGACCGGAACACGGTTTCACTTGTTTGCAAGGTATGGTATGAGATTGAAAGGCTGAGCCGGCGAACTGTCTTTGTGGGTAACTGCTATGCTGTGCGCCCTGAGCGTGTGGTGCTTCGGTTCCCCAATGTGCGGGCACTGACAGTGAAGGGGAAACCACACTTCGCTGATTTCAACCTTGTGCCACCTGATTGGGGTGGGTATGCCGGACCATGGATCGAGGCAGCAGCCAGGCGCTGCGTGGGTCTTGAGGAGCTGCGGATGAAGCGGATGGTGGTGTCAGATGAGAGCCTGGAGCTGCTTGCCAAATCATTCCCACGATTCAGGGCCCTAGTTCTTATCAGCTGTGAGGGGTTCAGCACCGATGGACTAGCAGCTATTGCAAGTCACTGCAAACTCTTGAGGGAGTTAGATTTGCAGGAAAATGAGGTGGATGATCGAGGGCCAAGGTGGCTCTCCTGCTTCCCTGATTCCTGCACGTCCCTTGTCTCCTTGAATTTCGCCTGCATCAAAGGGGAGGTTAATGCTGGTTCATTAGAGAGACTTGTTGCTAGGTCCCCAAGTCTTCGGAGTTTGAGGTTGAATCGATCTGTGTCAGTAGATACACTCTCGAAGATATTAATGCGCGCCCCTAATTTGGAGGATCTAGGGACTGGGAACTTGACAGATGACTTCCAAGCTGAATCGTATCTCAGGCTGACCCTTGCATTGGAGAAATGCAAACTGCTGAGGAGTTTGTCGGGCTTTTGGGATGCCTCCCCTTTGTGCCTTCCATTCATCTATCCTGTATGTGGGCAACTAACAGGTTTAAATTTGAGCTATGCTCCGACACTTGATTCTTCCGATCTCACCAAAATGATCAGCCACTGTGTGAAACTCCAACGTCTTTGGGTACTGGATTGCATCGCGGATAAGGGCTTGCAAGTGGTGGCCTCCAGTTGCAAGGATCTACAAGAACTCAGGGTATTCCCGTCAGACTTCTATATCGCCGGGTATTCCCCAGTAACAGAGGAGGGACTTGTTGCAATATCCTTGGGCTGTCAAAAACTGAGCTCATTGCTATATTTTTGTCATCAAATGACGAATGCCGCACTGCTTACTATAGCTAAGAACCGCCCAAATTTCACGCGATTCAGACTCTGTATCCTTGAGCCTGGGAAGCCTGATGCCATGACAAACCAACCATTAGATGAAGGTTTTGGTGCTATTGTTCGTGAATGCAAAGGGCTAAGGCGATTGTCAATATCGGGTCTTCTCACCGACAAGGTTTTCATGTATATTGGTAAATTCGCGAAACAACTTGAGATGCTTTCAATAGCATTTGCTGGAGATAGTGATGCGGGAATGATGCATGTTATGGAAGGATGCAATAATTTGAGGAAGCTGGAGATTAGAGATAGCCCATTTGGTGATGCTGCACTCTTGGAGAATGTTACCAAGTATGAGACAATGCGATCCCTTTGGATGTCATCGTGCAATGTCACAGAAAAAGGGTGCCAAATCCTTGCATCAAAGATGCCAATGCTTAATGTGGAGGTTATAAATGAGGTGGATGAGAGCAATGAAATGGACGAGAACCATGGAATCCCCAAAGTTGACAAGTTATATGTTTACCGCACAACTGCTGGGGCAAGGGATGATGCGCCAAATTTTGTTAAAATCCTATAG

>TaCOI3-B TRAES3BF018800040CFD_t1

ATGGGCGGGGAGGTGCCGGAGCCGCGGCGGCTCAGCCGCGCGCTCAGCTTCGGCGTGCCCGACGAGGCGCTGCACCTCGTCATGGGCTACGTCGACGCCCCGCGCGACCGGGAGGCCGCCTCGCTCGTCTGCCGCCGCTGGCACCGCATCGACGCGCTCACCCGCAAGCACGTCACCGTCGCCTTCTGCTACGCCGCCGACCCCTCGCGCCTCCTCGCCCGCTTCCCGCGCCTCGAGTCGCTGGCCCTCAAGGGCAGGCCGCGCGCCGCCATGTACGGCCTCATCTCCGACGACTGGGGCGCCTACGCCGCGCCCTGGGTCGCACGGCTCGCCGCGCCGCTCGAGTGCCTCAAGGCGCTCCACCTGCGACGCATGACCGTCACCGACGACGACGTCGCCACGCTCATCCGCTCCCGCGGCCACATGCTGCAGGAGCTCAAGCTCGACAAGTGCTCCGGCTTCTCCACCGACGCGCTCCGCCTCGTCGCCCGCTCCTGCAGATCCTTAAGAACATTATTTCTTGAAGAATGCGTGATTACTGACGAAGGTGGTGAATGGCTTCATGAACTTGCTGTCAACAATTCTGTTCTTGTGACACTGAACTTCTACATGACTGAGCTCAAAGTGGTGCCGGCTGATCTGGAGCTTCTAGCAAAGAACTGCAAATCATTACTTTCTTTAAAGATCAGTGAGTGTGACCTTTCAGACCTGATTGGTTTTTTCGAAGCAGCCAATGCATTGCAAGATTTTGCTGGAGGATCGTTCAATGAGGTAGGAGAGCTAACAAAGTATGAAAAAGTCAAGTTTCCACCAAGAGTATGCTTCTTGGGGCTTACGTTCATGGGGAAAAATGAGATGCCTGTTATCTTCCCCTTTTCTGCTTCATTAAAGAAGCTGGACTTGCAGTACACTTTCCTCAGCACTGAGGATCATTGCCAGCTTATCTCAAAATGCCCGAACCTATTTGTTCTTGAGGTGAGGAATGTGATAGGAGACAGAGGGCTGGAGGTTGTCGGCGATACATGCAAGAAGCTACGAAGACTTCGAATTGAGCGAGGGGATGATGATCCAGGTCTACAAGAAGAGCAAGGAGGAGTTTCTCAGTTAGGCCTGACAGCGGTAGCTGTTGGTTGCCGAGACCTGGAGTACATAGCTGCCTATGTATCTGATATCACCAACGGTGCTCTTGAATCCATTGGGACCTTCTGCAAAAATCTCTACGACTTCCGGCTTGTCCTGCTCGACAGACAAAAGCAGGTAACTGATCTGCCACTCGACAACGGTGTTCGTGCTCTGTTAAGGAGTTGCACCAAGCTCCGGAGATTTGCTCTCTACCTGAGACCTGGAGGGCTCTCAGACATAGGCCTCGACTACATCGGGCAGTACAGCGGCAACATTCAGTACATGCTGCTGGGCAACGTCGGTGAATCTGACCACGGGTTGATCCGCTTTGCGATAGGATGCATCAACCTGCGGAAGCTTGAGCTTCGGAGCTGCTGCTTCAGCGAGCAAGCCCTGTCCCTCGCGGTGCTCCACATGCCCTCGCTCAGGTACATATGGGTGCAAGGCTACAAAGCCTCTCCAGCAGGCCTCGAGCTCCTGCTCATGGCGAGGCGATTCTGGAACATCGAGTTCACGCCCCCCAGCCCCGAGGGCTTGTTCCGCATGACGCTTGAAGGAGAACCCTGCGTGGATAAGCAGGCCCAGGTTCTTGCCTACTACTCCCTTGCTGGGCAGAGGCAGGACTGCCCTGACTGGGTGACCCCGTTGCATCCAGCTGCATGA

>TaCOI3-D Traes_3DL_845220DFC.1

ATGACTGAGCTCAAAGTGGTGCCGGCTGATCTGGAGCTTCTAGCAAAGAACTGCAAATCATTACTTTCTTTAAAGATCAGTGAGTGTGACCTTTCAGACCTGATTGGTTTTTTCGAAGCAGCCAATGCATTGCAAGATTTTGCTGGAGGATCGTTCAATGAGGTAGGAGAGCTAACAAAGTATGAAAAAGTCAAGTTTCCACCAAGAATATGCTTCTTGGGGCTTACGTTCATGGGGAAAAATGAGATGCCTGTTATCTTCCCCTTCTCTGCTTCATTAAAGAAACTGGACTTGCAGTACACTTTCCTCACCACTGAGGATCATTGCCAGCTTATCTCAAAATGTCCAAACCTATTTGTTCTCGAGGTGAGGAATGTGATAGGAGATAGAGGGCTAGAGGTTGTCGGCGATACATGCAAGAAGCTACGAAGACTTCGAATTGAGCGAGGGGATGATGATCCAGGTCTACAAGAAGAGCAAGGAGGAGTTTCTCAGTTAGGCCTGACAGCGGTAGCTGTTGGTTGCCGTGACCTGGAGTACATAGCTGCCTAT

>TaCOI4-A Traes_3AL_2240B0C5F.1

ATGAATGAGTTGGTGATATCACTGGCCCACAAATTTACAAAGCTTCAAGTCCTTTCTCTAAGGCAAATCAAGCCTCAGCTTGAAGACAGTGCAGTGGAGGCTGTAGCAAATTCTTGTCATGATTTGCGCGAGTTGGATCTGAGCAGAAGCTTCAGGCTTAGTGATCGGTCCTTGTATGCTCTGGCACATGGGTGCCCTCACCTTACAAGGCTGAACATCAGTGGATGTTCCAATTTCAGTGATGCTGCTTTGATCTACCTCACTAGTCAGTGCAAGAACTTGAAATGCTTGAATCTGTGCGGGTGTGTGCGGGCAGCATCCGACAGAGCATTGCAGGCCATAGCCCGCAACTGTAGTCAGCTGCAATCTTTGAACCTTGGTTGGTGCGATACTGTCACTGACGGGGGAGTCACTAGCTTGGCATCAGGATGTCCTGAACTTAGGGCCGTCGACTTGTGTGGCTGTGTTCTTATAACAGATGAGAGCGTGGTTGCTCTTGCAAACGGCTGCCCACACCTGCGTTCCCTGGGGCTGTACTACTGCCAGAACATCACCGACCGGGCCATGTACTCG

>TaCOI4-B TRAES3BF021600080CFD_t1

ATGGTCAATGCACAGATGGTGAGTGGGTACTTGGACAATTCGTTCAATGCACTCGTGGTTTCTGGTGGCGGTGAGAGTGGTGGACAGACACAGAATGGTGTCACAGACACCACCTTGTCAGGTTGGAAGGACCTCCCCATGGAGCTTCTGCTGAGGATCATATCAGTCGCTGGAGATGACAGGATGGCCATTGTAGCCTCCGGTGTTTGCACCGGGTGGCGTGATGCACTAGGATGGGGAGCCACTAGTCTCTCCTTCTCGTGGTGCCAGGACCACATGAATGAGTTGGTGATATCACTCGCCCACAAATTTACAAAGCTTCAAGTCCTTTCTCTAAGGCAAATCAAGCCTCAGCTTGAAGACAGTGCAGTGGAGGCTGTAGCAAATTCTTGTCATGATTTGCGCGAGTTGGATCTGAGCAGAAGCTTCAGGCTTAGCGATCGGTCCTTGTATGCTCTGGCACATGGGTGCCCTCGCCTTACAAGGCTGAACATCAGTGGATGTTCCAATTTCAGTGATGCTGCTTTGATCTACCTCACTAGTCAGTGCAAGAACTTGAAATGCTTGAATCTATGCGGGTGTGTGCGGGCAGCATCCGACAGAGCGTTGCAGGCTATAGCCTGCAACTGTAGTCAGCTGCAATCTTTGAACCTTGGTTGGTGCGATACTGTCACTGACGGGGGAGTCACTAGCTTGGCATCAGGATGTCCTGAACTTAGGGCCGTCGACTTGTGTGGCTGCGTTCTTATAACAGATGAGAGTGTGGTTGCTCTTGCAAACGGCTGCCCTCACCTGCGTTCCCTGGGGCTGTACTACTGCCAGAACATCACCGACCGTGCCATGTACTCGCTGGCAGAGAACAGCCGCATCAGGAGCAAGGGCATGAGCTGGGACACGGCTAAGAACAGCCGCGGCCGCGACGACAAGGACGGCCTCGCCAGCCTGAACATCAGCCAGTGCACCGCGCTGACGCCCCCGGCAGTGCAGGCGGTGTGCGACTCCTTCCCGGCGCTCCACACCTGCCCGGAGAGGCACTCCCTCATCATCAGCGGCTGCCTGAGCCTCACCACCGTGCACTGCGCGTGCGCCCACCACCGGCACCGCGCCGGGGCCGGGAGAGCCATCCTGTCTAACCATGCCTACTGA

>TaCOI4-D Traes_3DL_3DB9F1EC5.1

ATGGTCAATGCACAGATGTTGAGTGGGTACTTGGACAATTCGTTCAATGCACTCATGGTTTCTGGTGGCGGTGAGAGTGGACAGCCACAGAATGGTGGCACAGACACCACCTTGTCAGGTTGGAAGGACCTTCCCATGGAGCTTCTGCTGAGGATCATATCAGTCGCTGGAGATGACAGGNNCATTGTAGCCTCCGGTGTTTGCACCGGGTGGCGTGATGCACTAGGATGGGGAGCCACAAGTCTCTCCTTCTCGTGGTGCCAGGACCACATGAATGAGTTGGTGATATCACTGGCCCACAAATTTACAAAGCTTCAAGTCCTTTCTCTAAGGCAAATCAAGCCTCAGCTTGAAGACACTGCAGTGGAGGCTGTAGCAAATTCTTGTCATGATTTGCGCGAGTTGGATCTGAGCAGAAGCTTCAGGCTTAGCGATCGGTCCTTGTATGCTCTGGCACATGGGTGCCCTCACCTTACAAGGCTGAACATCAGTGGATGTTCCAATTTCAGTGATGCTGCTTTGATCTACCTCACTAGTCAGTGCAAGAACCTGAAATGCTTGAATCTGTGCGGGTGTGTGCGGGCAGCATCCGACAGAGCGTTGCAGGCCATAGCCTGCAACTGTAGTCAGCTGCAATCTTTGAACCTTGGTTGGTGCGATACTGTCACTGACGGGGGAGTCACTAGCCTGGCATCAGGATGTCCTGAACTTAGGGCCGTCGACTTGTGTGGCTGTGTTCTTATAACAGGTGCAACCTTCATACTTCTCCAAGATCAAGTGTGCTCACCCAAGTTTATTTCGGTTGGTAATGATCTTCACCATATACGGCATTGTTATCTAAGTTCAGCATCG

>TaCOI5-A Traes_4AS_AD3F2B991.1

ATGAGCTTCGGGATCCCGGACGTGGCGCTGGGGCTGGTCATGGGGTGCGTGGAGGACCCCTGGGACCGCGACGCCATCTCGCTCGTCTGCCGCCACTGGTGCAAGGTCGACGCGCTCAGCCGCAAGCACGTCACCGTCGCCATGGCCTACTCCACCACCCCCGACCGCCTCTTCCGCCGCTTCCCCTGCCTCGAGTCGCTCAAGCTCAAGGCCAAGCCCCGCGCCTCCATGTTCAACCTCATCCCCGAGGACTGGGGAGGCTCCGCCTCGCCCTGGATCCGCGAGCTCTCCGCCTCCTTCCACTTCCTCAAGGTGCTGCACCTCCGCCGGATGATTGTCTCCGACGACGACGTTGCCGTGCTCGTGCGCGCCAAGGCCCACATGCTCGTCTCCCTCAAGCTTGACCGCTGCTCCGGCTTCTCCACCTCCTCCCTCGCTCTCCTCGCCCGCTGCTGCAAGAAACTGGAAACGTTGTTTCTTGAAGAAAGTTCTGTTGCTGAGAAAGAAAATGATGAATGGCTCCGTGAGCTTGCTACCAGCAATACTGTCCTTGAGACGCTGAATTTCTTTCTGACGGATCTCAGGGCATCCCCTGCACATCTTCTCCTCCTTGTGCGAAATTGCCGAAGGCTGAAAACTCTCAAGATTAGCGACTGTTTCATGTCTGACCTGGTCGACCTGTTCCGTACAGCAGAAACACTACAAGACTTTGCTGGTGGTTCCTTTGATGATCAAGATCAAGGTGGGAATTATGCTAACTACTATTTCCCTCCTTCAGTACAGCACTTGAGTTTGCTCTACATGGGAACAAATGAGATGCAGATATTATTTCCATATGGTGCCACACTCAAGAAGTTGGACCTTCAGTTCACATTCCTTACCACAGAGGATCACTGTCAATTAGTCCAGCGCTGCCCAAATCTAGAAGTTTTGGAGGTGAGGGATGTGATAGGAGATCGAGGGTTAGAAGTTATTGCGCGGACCTGCAAGAAATTACAGCGACTCAGAGTCGAGAGAGGAGATGATGACCAAGGAGGTCTTGAGGACGAACAGGGTAGAGTGACACAAGTAGGATTGATGGCTGTAGCTGAAGGCTGTCCTGATTTGGAGTACTGGGCAGTACATGTGTCTGACATTACAAATGCAGCTCTTGAGGCTATTGGCGCATTCAGCAAAAACCTGAACGATTTCCGACTTGTCCTGCTTGATAGAGAGGTGCATATAACTGAACTGCCCCTTGACAACGGGGTTCGGGCTTTGCTGAGAGGTTGCACCAAACTCCGGAGGTTTGCATTTTATGTGAGACCTGGAGCTCTATCAGATATTGGCCTTTCTTATGTTGGCGAATTTAGCAAGACCGTCCGCTACATGTTGCTTGGGAATGCCGGGGGGTCTGATGATGGACTGCTGGCATTTGCACGAGGATGCCCAAGCTTGCAGAAATTGGAGCTAAGGAGTTGCTGCTTTAGTGAACGTGCATTGGCAGTTGCAGCCTTACAGCTGAAGTCACTCAGATATCTTTGGGTGCAGGGATACAAGGCATCTCCTACTGGCACCGATCTCATGGCAATGGTACGCCCCTTCTGGAACATTGAGTTTATTGCACCAAATCAAGATGAGCCTTGCCCAGAGGGTCAGGCACAGATTCTGGCATACTACTCTCTGGCTGGGGCAAGGACAGATTGTCCTCAGTCAGTAATTCCCCTCCATCCGTCAGTGGGAAGCTAA

>TaCOI5-B Traes_4BL_499E7F095.1

ATGGGCGGCGACGAGCGGCACCTGGGGAGGACCATGAGCTTCGGGATCCCGGACGTGGCGCTGGGGCTCGTCATGGGGTGCGTGGAGGACCCCTGGGACCGCGACGCCATCTCGCTCGTCTGCCGCCACTGGTGCAAGGTCGACGCGCTCAGCCGCAAGCACGTCACCGTCGCCATGGCCTACTCCACCACCCCCGACCGCCTCTTCCGCCGCTTCCCCTGCCTCGAGTCGCTCAAGCTCAAGGCCAAGCCCCGCGCCTCCATGTTCAACCTCATCCCCGAGGACTGGGGCGGCTCCGCTTCGCCCTGGATCCGCGAGCTCTCTGCCTCCTTCCACTTCCTCAAGGTGCTGCACCTCCGCCGAATGATTGTCTCCGACGACGACGTCGCCGTGCTCGTGCGCGCCAAGGCCCACATGCTCGTCTCCCTCAAGCTTGACCGATGCTCCGGCTTCTCCACGTCCTCCCTCGCTCTCCTCGCCCGCTGCTGCAAGAAACTGGAAACCTTGTTTCTTGAAGAAAGTTCTGTTGCTGAGAAAGAAAATGATGAATGGCTCCGCGAGCTTGCTACCAGCAATACTGTCCTTGAGACGCTGAATTTCTTTCTGACAGATCTCAGGGCATCCCCTGCATATCTTCTCCTCCTTGTGCGAAATTGCCGAAGGCTGAGAACTCTCAAGATTAGCGACTGTTTCATGTCTGACCTGGTTGACCTGTTCCGTACAGCAGAAACGCTACAAGACTTTGCTGGTGGTTCCTTTGATGATCAAGATCAGGGTGGGAATTATGCTAACTACTATTTCCCTCCTTCAGTACAGCGCTTGAGTTTGCTCTACATGGGAACAAATGAGATGCAGATATTATTTCCATATGGCGCCACACTCAAGAAGCTGGACCTTCAGTTTACATTCCTTACCACAGAGGATCACTGTCAATTAGTCCAGCGCTGCCCAAATCTAGAAGTTTTGGAGGTAAGGGATGTGATAGGGGATCGAGGGTTGGAAGTCGTTGCACAGACCTGCAAGAAATTACAGCGACTCAGAGTCGAGAGAGGAGACGATGACCAAGGAGGTCTTGAGGACGAACAGGGTAGAGTGACACAAGTAGGACTGATGGCTGTTACTCAAGGCTGTCCTGATTTGGAGTACTGGGCAGTACATGTGTCTGACATTACAAATGCAGCTCTTGAGGCCATTGGTACGTTCAGCAAAAACCTGAATGATTTCCGACTTGTCCTGCTTGATAGAGAGGTGCATATAACCGAACTGCCCCTTGACAACGGGGTTCGGGCTTTGATGAGAGGTTGCACCAAACTCCGGAGGTTTGCATTTTATGTGAGACCTGGAGCTCTATCAGATATCGGCCTTTCCTATGTTGGTGAATTTAGCAAGACCGTCCGCTACATGTTGCTTGGGAATGCCGGGGGATCTGATGATGGACTGCTGGCATTTGCACGAGGATGCCCAAGCTTGCAGAAATTGGAGCTAAGGAGTTGCTGCTTTAGTGAACGTGCATTGGCAGTTGCAGCCTTACAGCTGAAGTCACTCAGATATCTTTGGGTGCAGGGATACAAGGCATCTCCTACTGGCACCGATCTCATGGCAATGGTACGCCCCTTCTGGAACATTGAGTTTATTGCACCAAATCAAGATGAGCCTTGCCCAGAGGGTCAGGCACAGATTCTGGCATACTACTCTCTGGCTGGGGCAAGGACAGATTGTCCTCAGTCTGTAATTCCCCTCTATCCATCAGTCGGAGGCTAA

>TaCOI5-D Traes_4DL_8C2C2ADD4.1

ATGGGCGGCGACGAGCGGCACCTGGGGAGGACCATGAGCTTCGGGATCCCGGACGTGGCGCTGGGGCTGGTCATGGGGTGCGTGGAGGACCCCTGGGACCGCGACGCCATCTCGCTCGTCTGCCGCCACTGGTGCAAGGTCGACGCGCTCAGCCGCAAGCACGTCACCGTCGCCATGGCCTACTCCACCACCCCCGACCGCCTCTTCCGCCGCTTCCCCTGCCTCGAGTCGCTCAAGCTCAAGGCCAAGCCCCGCGCCTCCATGTTCAACCTCATCCCCGAGGACTGGGGCGGCTCCGCCTCGCCCTGGATCCGCGAGCTCTCCGCCTCCTTCCACTTCCTCAAGGTGCTGCACCTCCGCCGGATGATTGTCTCCGACGACGACGTCGCCGTGCTCGTGCGCGCCAAGGCCCACATGCTCGTCTCCCTCAAGCTTGACCGCTGCTCCGGCTTCTCCACCTCCTCCCTCGCTCTCCTCGCCCGCTGCTGCAAGAAACTGGAAACGTTGTTTCTTGAAGAAAGTTCTGTTGCTGAGAAAGAAAATGATGAATGGCTCCGCGAGCTTGCTACCAGCAATACTGTCCTTGAGACGCTGAATTTCTTTCTGACGGATCTCAGGGCATCCCCAGCATATCTTTTCCTTCTTGTGCGAAATTGCCGAAGGCTGAAAACTCTCAAGATTAGCGACTGTTTCATGTCTGACCTGGTCGACCTGTTCCGTACAGCAGAAACACTACAAGACTTTGCTGGTGGTTCCTTTGATGATCAAGATCAAGGTGGGAATTATGCTAACTACTATTTCCCTCCTTCGGTACAGCGCTTGAGTTTGCTCTACATGGGAACAAATGAGATGCAGATATTATTTCCATATGGTGCCACACTCAAGAAGTTAGACCTTCAGTTTACGTTCCTTACCACAGAGGATCACTGTCAATTAGTCCAGCGATGCCCAAATCTAGAAGTTTTGGAGGTGAGGGATGTGATAGGAGACCGAGGGTTAGAAGTTGTTGCGCGGACCTGCAAGAAATTACAGCGACTCAGAGTCGAGAGAGGAGACGATGACCAAGGAGGTCTTGAGGACGAACAGGGTAGAGTGACACAAGTAGGATTGATGGCTGTAGCTGAAGGCTGTCCTGATTTGGAGTACTGGGCAGTACATGTGTCTGACATTACAAATGCAGCTCTTGAGGCCATTGGTGCGTTCAGCAAAAACCTGAACGATTTCCGACTTGTCCTGCTTGATAGAGAGGTGCATATAACTGAACTGCCCCTTGACAACGGGGTTCGGGCTTTGCTGAGAGGTTGCACCAAACTCCGGAGGTTTGCATTTTATGTGAGACCTGGAGCTCTATCAGATATTGGCCTTTCTTATGTTGGCGAATTTGGCAAGACTGTCCGCTACATGTTGCTTGGGAATGCTGGGGGATCTGATGATGGACTGCTGGCATTTGCACGAGGATGCCCAAGCTTGCAGAAATTGGAGCTAAGGAGTTGCTGCTTTAGTGAACGTGCATTGGCAGTTGCAGCCTTACAGCTGAAGTCACTCAGATATCTTTGGGTGCAGGGATACAAGGCATCTCCTACCGGCACCGATCTCATGGCAATGGTACGCCCCTTCTGGAACATTGAGTTTATTGCACCAAATCAAGATGAGCCTTGCCCAGAGGGTCAGGCACAGATTCTGGCATACTACTCTCTGGCTGGGGCAAGGACAGATTGTCCTCACTCAGTAATTCCCCTCTATCCGTCAGTCGGAAGCTAA

>TaCOI6-A Traes_5AL_27F194099.2

ATGCCCTACTTCCCAGATGAAGTGGTGGAGCACATCCTTGGCTTCATATCGTCGCACCGTGACCGCGATGCTGCATCTCTCGTGTGTCACGCGTGGTACCGCATCGAGGGCCTCACTCGCCGCTCCGTGTTCATTTCCAACTGCTATGCCGTGCGCCCAGAGCGTGTGCACGCGCGTTTCCCCTGCCTGCGCTCGCTGACCGTGAAGGGCAAGCCACGCTTTGCTGACTTCAACCTTGTCCCTGCGGGGTGGGGTGCCTCAGCGGAGCCATGGGTGGATGCGTGTGCCCGTGCGTGCCCTGGCCTTGAAGAGCTCCGCCTGAAGCGGATGGTTGTCACTGATGGTTGCCTCAATCACCTTGCTCACTCATTTCCCAATTTGAGATCACTAGTCCTTGTTAGCTGTGAGGGGTTCAGCACTGATGGCCTTGCTACTATTGCCACCAATTGCAGGTTTCTGAAGGAACTTGACTTACAAGGGAGTCAGGTGGAGTTTCGAGGCCGTCATTGGTTTAGTTGTTTCCCCAAGCCTTCGACATCATTAGAATCCTTGAATTTTGCTTGCTTGGATGGAGCAGTGAGTGCTAATGCATTGGAAAGTCTTGTTGCAAGGAGTCCAAATCTTAAAAGCTTAAGGTTAAATCGTGCAGTTCCACCAGCTGTTTTAGCCAAAATTCTTACTTCCGCTCCTAAGCTGGTGGATTTAGGTACAGGATTGGTTGCTCAAAGCAATAATGCTGGTGCACTCCCCAGTCTCTACAGTGCTATTCAACAATGCAGTTCTCTGAATAGTTTATCTGGCTTTTGGGATTCTCCACGTTGGATTACTCCAATAATACAATATATTTGCAAGAACCTAACATGCTTGAACCTTAGCTATGCTCCAATGTTTCGGACAGTTGATCTTATTGGAATTATTCGCCAATGTCAGAATCTCCGACACTTGTGGGTACTAGATCACATTGGTGATGCAGGATTAAAGGTTGTAGCCTCTTCTTGCCTGGAGCTGCAAGAGTTGAGGGTATTTCCTGCGAATGCAAATGTGTTAATAAGCACTGGTGTGACAGAGGAAGGGCTGGTTGCAGTATCTTCAGGCTGTCGGAAGCTAAACTCTGTTCTCTATTCCTGCCGTCGAATGACTAATTCTGCTCTGATCACGGTGGCAAAGAACTGCTCGCGAATCACGTCCTTCAGACTGCATATCTGCCTGCATGGGTCAGTAGATGCCGTGACAGGCCAGCCACTGGACGAGGGTTTTGGGGCAATCGTCCGGTCATGCAAGGGCCTCAGGCGCTTATCTATGTCTGGCCTTCTCACGGACAGTGTGTTCCTGTACATCGGCATGTACGCCGAGAGGCTGGAGACGCTCTCTGTCTCGTTTGCAGGAGATAGTGATGATGGCATGATCTATGTGCTCAATGGCTGCAAGAATCTCAGGAAGCTGGAGATCAGGAACTGCCCATTTGGCAACACCGCGCTTCTCGCAGGCATGCACCGGTACGAGGCAATGCGCTCGCTTTGGATGTCGTCGTGCGACATCACCCTGGGTGGCTGCAGGTCCCTCGCGGCAGCCATGCCAGGCCTCAACGTCGAGGTCATCAGCCAGGCGGATGGAGGCGCCAACGATGCAAAGAAGGTGGAGAAGCTGTATGTCTACAGGACACTCGCCGGCCCAAGGGTTGATGCCCCTGGATTCGTCTCGGCACTGTAA

>TaCOI6-B Traes_5BL_7AE04C6B2.1

ATGCCCTACTTCCCAGATGAAGTGGTGGAGCACATCCTTGGCTTCGTATCGTCGCACCGTGACCGCGACGCTGCATCTCTCGTGTGCCACGCATGGTACCGCATCGAGGGCCTCACTCGCCGCTCCGTGTTCATTTCCAACTGCTATGCGGTGCGCCCGGAGCGTGTGCACGCCCGTTTCCCCTGCCTGCGCTCGCTGACCGTGAAGGGCAAGCCACGCTTTGCTGACTTCAACCTTGTCCCTGCGGGGTGGGGTGCCTCAGCGGAGCCATGGGTGGATGCGTGTGCCCGTGCATGCCCTGGCCTTGAAGAGCTCCGCCTGAAGCGGATGGTTGTCACTGATGGTTGCCTCAATCACCTTGCTCACTCATTCCCTAATTTGAGGTCACTAGTCCTTGTTAGCTGTGAGGGGTTCAGCACTGATGGCCTTGCTACTATTGCCACCAATTGCAGGTTTCTCAAGGAGCTTGACTTACAAGGGAGTCAGGTGGAGTTTCGAGGCCGTCATTGGTTGAGTTGTTTCCCCAAGCCTTCGACATCATTAGAATCCTTGAATTTTGCTTGCTTGGATGGAGCAGTGAGTGCTAATGCATTGGAAAGTCTTGTTGCAAGGAGTCCAAATCTTAAAAGCTTAAGGTTAAATCGTGCAGTTCCACCAGCTGTTTTAGCCAAAATTCTTACTTCCGCTCCTAAGCTGGTGGATTTAGGTACAGGATTGGTTGCTCAAAGCAATAATGCTGGTGCACTCCCCAGTCTCTACAGTGCTATTCAGCAATGCACTTCTTTGAATAGTTTATCAGGCTTTTGGGATTCTTCACGTTGGATTACTCCAATAATACATTGTATTTGCAAGAACCTAACATGCTTGAACCTTAGCTATGCTCCAATGTTTCGGACAGTTGATCTTATTGGAACTATTCACCAATGTCAGAATCTCCGACACTTGTGGGTACTAGATCATATTGGCGATGCAGGATTGAAGGTTGTAGCCTCTTCTTGCCTGGAGCTGCAAGAGTTGAGGATATTTCCTGCGAATCCTGCGAATGCAAATGTGTTAGCAAGCACTGGTGTGACAGAGGAAGGGCTGGTTGCAGTATCTTCAGGCTGTCGGAAGCTAAACTCTGTGCTCTATTCCTGCAGTCGAATGACTAATTCTGCTCTGATTACGGTGGCAAAGAACTGCTCGCGAATCACGTCCTTCAGACTGCATATCTGCCTGCATGGGTCAGTAGATGCCGTGACAGGCCAGCCACTCGACGAGGGCTTTGGGGCAATTGTCCGGTCATGCAAGGGCCTCAGGCGTCTATCTATGTCTGGCCTTCTCACGGACAGCGTGTTCCTGTACATCGGCATGTACGCCGAGAGGCTGGAGACGCTCTCTGTCTCGTTTGCAGGAGATAGTGATGATGGCATGATCTATGTGCTCAACGGCTGCAAGAATCTCAGGAAGCTGGAGATCAGGAACTGCCCATTTGGCAACACGGCGCTTCTTGCAGGCACTCACAGGTACGAGGCGATGCGCTCGCTTTGGATGTCGTCGTGCGACATCACCCTGGGTGGCTGCAGGTCCCTCGCGGCAGCCATGCCAGGCCTCAACGTCGAGGTCATCTGCCAGGCGGATGGAGGCGCCAACGATGCAAAGAAGGTGGACAAGCTGTATGTCTACAGGACACTCGCCGGCCCGAGGGATGACGCCCCTGGATTCGTCTCGGCGCTGTAA

>TaCOI6-D Traes_5DL_4E8DD7C5F.3

ATGCCCTACTTCCCAGATGAAGTGGTGGAGCACATCCTTGGCTTCGTATCGTCGCACCGTGACCGCGATGCTGCATCTCTCGTGTGCCACGCGTGGTACCGCATCGAGGGCCTCACTCGCCGCTCCGTGTTCATTTCCAATTGCTATGCGGTGCGCCCAGAGCGTGTGCACACACGTTTCCCCTGCCTGCGCTCACTGACCGTGAAGGGCAAACCACGCTTTGCTGACTTCAACCTTGTCCCTGCGGGGTGGGGTGCCTCAGCGGAGCCATGGGTGGATGCGTGTGCCCGTGCATGCCCTGGCCTTGAAGAGCTCCGCCTGAAGCGGATGGTTATCACTGATGGTTGCCTCAATCACCTTGCTCACTCATTTCCCAATTTGAGGTCACTAGTCCTTGTTAGCTGTGAGGGGTTCAGCACTGATGGCCTTGCTACTATTGCCACCAATTGCAGGTTTCTCAAGGAACTTGACTTACAAGGGAGTCAGGTGGAGTTTCGAGGCCGTCATTGGTTGAGTTGTTTCCCCAAGCCTTTGACATCATTAGAATCCTTGAATTTTGCTTGCTTGGATGGAGCAGTGAGTGCTAATGCATTGGAAAGTCTTGTTGCAAGGAGTCCAAATCTTAAAAGCTTAAGGTTAAATCGTGCAGTTCCACCAGCTGTTTTAGCCAAAATTCTTACTTCCGCTCCTAAGCTGGTGGATTTAGGTACAGGATTGGTTGCTCAAAGCAATAATGCTGGTGCACTCCCCAGTCTCTACAGTGCTATTCAGCAATGCACTTCTCTGAATAGTTTATCAGGCTTTTGGGATTCTCAACGTTGGATTACTCCAATAATACATTATATTTGCAAGAACCTAACATACTTGAACCTTAGCTATGCTCCAATGTTTCGGACAGTTGATCTTATTGGAACTATTCACCAATGTCAGAATCTCCGACACTTGTGGGTACTAGATCACATTGGCGATGCAGGATTGAAGGTTGTAGCCTCTTCTTGCCTGGAGCTGCAAGAGTTGAGGATATTTCCTGCGAATGCAAATGTGTTAGCAAGCACTGGTGTGACAGAGGAAGGGCTGGTTGCAGTATCTTCAGGATGTCGGAAGCTAAACTCTGTGCTCTATTCCTGCAGTCGAATGACTAATTCTGCTCTGATTACGGTGGCAAAGAACTGCTCGCGAATCACGTCCTTCAGACTGCATATCTGCCTGCATGGGTCAGTAGATGCCGTGACAGGCCAGCCACTGGACGAGGGTTTTGGGGCAATCGTCCGGTCATGCAAGGGCCTCAGGCGTCTATCTATGTCTGGCCTTCTCACGGACAGCGTGTTCCTGTACATCGGCATGTACGCCGAGAGGCTGGAGACTCTCTCTGTCGCGTTTGCAGGAGATAGTGATGATGGCATGATCTATGTGCTCAACGGCTGCAAGAATCTCAGGAAGCTGGAGATCAGGAACTGCCCATTTGGCAACACCGCGCTTCTTGCAGGCACGCACAGGTACGAGGCGATGCGCTCGCTTTGGATGTCGTCGTGCGACATCACCCTGGGTGGCTGCAGGTCCCTCGCGGCAGCCATGCCAGGCCTCAACGTCGAGGTCATCAGCCAGGCAGATGGAGGCGCCAACGATGCAAAGAAGGTGGAGAAGCTGTATGTCTACAGGACACTCGCTGGTCCGAGAGATGATGCCCCTGGATTCGTCTCGGCACTGTAA

>TaCOI7-A Traes_6AL_0F53490CA.1

ATGGCAAGGGCACCCCATCTAACTCACCTTGGCACTGGATCATTCCGATCTGAGCCAGGCCCTGGGGGTGCTTTGTCTGTGTCTGAGCTCGCTACCTCTTTCGCGGCGTCCAGATCACTTGTTTGTTTGTCAGGTTTCTTGGATGCCAATGCAGCATACCTCCCAGCAATCTACCAAGTTTGTGCCAATCTCACTTCCCTCAATTTTAGCTTTGCGGGTCTAACAGATGAAGAGTTCATACCAGTTATTCGCCATTGCATCAATCTTCGCACTTTATGGGTTCTTGATACTGTGGGTGATGAAGGCCTTAGGGCTGTGGCTGAAACATGCTCAAATCTCCGTGAGCTACGTGTTTTTCCTCTGGATGCCACCGAGGATTCTGAGGGCTCAGTCTCAGATATTGGTCTCCAGGCAATCTCAGAAGGCTGCCGAAAGCTCGAATCAATTCTCTACTTTTGCCAGCGCATGACAAATGCAGCAGTAATTGCTATGTCCGAGAACTGCCCTGACCTTTTGGTGTTCCGCCTCTGTATTATGGGCCGCCACCGCCCTGATCGGATTACCGGGGCGCCCATGGATGAGGGTTTTGGGGCGATTGTGATGAACTGCAAGAAGCTCACCAGACTCTCAGTCTCTGGCCTGCTCACTGATAAGGCGTTTGCATACATTGGGAGACACGGAAAACTCATAAAGACTCTGTCTGTTGCCTTTGCTGGGAATAGTGACATGTCTCTTCAGCATGTGTTTGAGGGGTGCACTAGGTTGCAGAAGCTCGAGGTCAGAGATAGCCCTTTTGGCGACAAAGGATTGCTCTCTGGCCTGAACTATTTTTACAACATGAGGTTCTTTTGGATGAACTCATGCAGGCTAACTGTGAAGGGTTGTGGGGATGTAGCTCAGCAAATGCCTAATCTGGTGGTTGAAGTAATGAAGGAAAATGAAGGGGAAATGGATACCGTTGATAAGCTGTACCTGTATCGATCGTTGGCAGGACCAAGGGAAGATGCTCCATCATTTGTCAACATCTTGTAG

>TaCOI7-B Traes_6BL_9E82CDBD1.1

ATGGATCGTTGCATCTATTTTTTTAGGCATCTTCGAGTACTGGATCTGACTGAAGATTATTTCCATGAGGAGGAGAGTGAAGTAGTGGATTGGATCTCCAAGTTTCCAGAGTGCAACACGTCGCTGGAGTCGCTTGTATTTGATTGTGTTAGTGTCCCATTCAACTTTGAGGCCCTGGAGGCACTTGTTGCACGCTCACCAGCTCTGCGTCGTCTTCGCGTTAATGACCATGTGTCGATAGAGCAGTTGCGTCGTCTCATGGCAAGGGCACCCCATCTAACTCACCTTGGCACTGGATCATTCCGATCTGAGCCAGGCCCTGGGGGTGCTTTGTCTGTGTCTGAGCTCGCTACCTCTTTCGCAGCGTCCAGATCACTTGTTTGTTTGTCAGGTTTCTTGGATGCCAATGCAGCATACCTCCCAGCAATCTACCAAGTTTGTGCCAATCTCACTTCCCTCAATTTTAGCTTTGCGGGTCTAACTGCTGAAGAGTTCATACCAGTTATTCGCCACTGCGTCAATCTTCGCACTTTATGGGTTCTTGATACTGTGGGTGATGAAGGCCTTAGGGCTGTGGCTGAAACATGCTCAAATCTCCGTGAGCTACGTGTTTTTCCTTTGGATGCCACCGAGGATTCTGAGGGCTCAGTCTCAGATATTGGTCTCCAGGCAATCTCAGAAGGCTGCCGAATGCTTGAATCAATTCTCTACTTTTGCCAGCGCATGACAAATGCAGCAGTAATTGCTATGTCCGAGAACTGCCCTGACCTTTTGGTGTTCCGCCTCTGTATTATGGGCCGCCACCGCCCTGATCGGATTACCGGGGCGCCCATGGATGAGGGTTTTGGGGCGATTGTGATGAACTGCAAGAAGCTCACCAGACTTTCAGTCTCTGGCCTGCTCACTGATAAGGCGTTTGCATACATTGGAAGACACGGAAAACTCATAAAGACTCTGTCTGTTGCCTTTGCCGGGAATAGTGACATGTCTCTTCAACACGTGTTTGAGGGGTGCACTAGGTTGCAGAAGCTTGAGGTCAGGGATAGCCCTTTTGGCGACAAAGGATTGCTCTCTGGCCTGAACTATTTTTACAACATGAGGTTCTTTTGGATGAACTCATGCAGGCTAACTGTGAAGGGTTGTGGGGATGTAGCTCAGCAAATGCCTAATCTGGTGGTTGAAGTAATGAAGGAAAATGAAGGGGAAATGGATACCGTTGATAAGCTGTACCTGTATCGATCGTTGGCAGGACCAAGGGAAGATGCTCCATCATTTGTCAACATCTTGTAG

>TaCOI7-D Traes_6DL_AD0DAD6D1.2

ATGGCAAGGGCACCCCATCTGACTCACCTTGGCACTGGATCATTCCGATCTGAGCCAGGCCCTGGTGGTGCGTTGTCTGTGTCTGAGCTCGCTACCTCTTTTGCGGCGTCCAGATCACTTGTTTGTTTGTCAGGTTTCTTGGATGCCAATGCAGCATACCTCCCAGCAATCTACCAAGTTTGTGCCAACCTCACTTCCCTCAATTTTAGCTTTGCGGGTCTAACTGATGAAGAGTTCATACCAGTTATTCGCCACTGCGTCAATCTTCGCACTTTATGGGTTCTTGATACTGTGGGTGATGAAGGCCTTAGGGCTGTGGCTGAAACATGCTCAAATCTCCGTGAGCTACGTGTTTTTCCTCTGGATGCCACCGAGGATTCTGAGGGCTCAGTCTCAGATATTGGTCTCCAGGCAATCTCAGAAGGCTGCCGGAAGCTTGAATCAATTCTCTACTTTTGCCAGCGCATGACAAATGCAGCAGTAATTGCTATGTCCGAGAACTGCCCTGACCTTTTGGTGTTCCGCCTCTGTATTATGGGCCGCCACCGCCCTGATCGGATTACCGGGGCGCCCATGGATGAGGGTTTTGGGGCGATTGTGATGAACTGCAAGAGGCTCACCAGACTTTCAGTCTCTGGCCTGCTCACTGATAAGGCGTTTGCATACATTGGAGGACACGGAAAACTCATAAAGACTCTGTCTGTTGCCTTTGCCGGGAATAGTGACATGTCTCTTCAGCACGTGTTTGAGGGGTGCACTAGGTTGCAGAAGCTTGAGGTCAGAGATAGCCCTTTTGGCGACAAAGGATTGCTCTCTGGCCTGAACTATTTTTACAACATGAGGTTCTTTTGGATGAACTCATGCAGGCTAACTGTGAAGGGTTGTGGGGATGTAGCTCAGCAAATGCCTAATCTGGTGGTTGAAGTGATGAAGGAAAATGAAGGGGAAATGGATACCGTTGATAAGCTGTACCTGTATCGATCATTGGCAGGACCAAGGGAAGATGCTCCATCATTTGTCAACATCTTGTAG

>TaCOI8-A Traes_7AS_78FEAE00C.1

ATGTTTGATTGCGTTGCTGTCCCATTCAACTTTGAGGCCCTAGAGGCCCTTGTGGCACGCTCACCCTCTCTTCGCCGGCTGCGTGTGAACCACTACGTTTCGGTGGAGCAGCTACGCCGTCTCATGGCACGGGCTCCGCAGCTCACACACCTTGGCACTGGGGCCTTTCGTCCTGAGGCTCCACAAGGTGGAGGCATGTCGGTGTCTGAACTCGCACCCTCTTTTGCAGCCTCAACGTCTATAGTCTGCCTCTCTGGGTTTCAGGAGGTCAACCCTGAATACCTTCCAGCAATCTACCCAGTGTGTGGTAATCTCACCTCCCTTAACGTTAGCTTCGCGAGCCTAACTGCTGAGGATCTGACACCAGTCATTCGCCAATGCCACAAACTTCAGACATTCTGGGTTCTTGATACTGCGGGTGATGAATGCCTCAGGGCTGTGGCTGAGACATGCTCTGATCTTCGTGAGCTGCGAGTATTTCCGCTGGATGCCACCGAAGACTCTGATGGGTCTGTGTCAGATGTTGGGCTTCAGGCTATATCCGAAGGTTGCCGAAAGCTTGAATCCATACTTTACTTTTGCCAGCGGATGACAAATGCTGCGGTCGTAGCTATGTCCAATAACTGCCCTGACCTTGTGGTATTCCGTCTTTGCATCATGGGTCGCCACCGCCCTGATCGTATTACTGGGGAGCCCATGGATGACGGCTTCGGCGCGATAGTGAAGAACTGCAAGAAACTTACTAGGCTCTCAGTCTCTGGCCTGCTCACTGATAAGGCATTTGCATACATTGGGCAATATGGCAAACTAATAAAGACTTTGTCTCTTGCCTTCTCGGGGAACAGTGACCTGTCGCTTCAGTTTTTGTTCGAGGGATGCACTCGGTTACAGAAGCTTGAGGTCAGAGATAGCCCATTCAGTGATAGGGGACTGCTTTGTGGTTTGGATTATTTCTACAACATGAGGTTCCTGTGGATGAATTCATGCAGGCTAACCATGAGGGGTTGTAGAGAGGTAGCTCAGCGGATGCCTAACTTGGTTGTTGAAGTAATGGAGGAGCAAAATGAGGATAAGGTGGAAACAGAGACTGTTGATAAATTGTACCTGTACCGCTCACTAGCAGGGCCAAGGGGTGATGCACCGCCATTGGTGAAAATTTTGTAG

>AetCOI1 EMT18237

ATGGGGTGCGTGGAGGACCCCTGGGACCGCGACGCCATCTCGCTCGTCTGCCGCCACTGGTGCAAGGTCGACGCGCTCAGCCGCAAGCACGTCACCGTCGCCATGGCCTACTCCACCACCCCCGACCGCCTCTTCCGCCGCTTCCCCTGCCTCGAGTCGCTCAAGCTCAAGGCCAAGCCCCGCGCCTCCATGTTCAACCTCATCCCCGAGGACTGGGGCGGCTCCGCCTCGCCCTGGATCCGCGAGCTCTCCGCCTCCTTCCACTTCCTCAAGGTGCTGCACCTCCGCCGGATGATTGTCTCCGACGACGACGTCGCCGTGCTCGTGCGCGCCAAGGCCCACATGCTCGTCTCCCTCAAGCTTGACCGCTGCTCCGGCTTCTCCACCTCCTCCCTCGCTCTCCTCGCCCGCTGCTGCAAGAAACTGGAAACGTTGTTTCTTGAAGAAAGTTCTGTTGCTGAGAAAGAAAATGATGAATGGCTCCGCGAGCTTGCTACCAGCAATACTGTCCTTGAGACGCTGAATTTCTTTCTGACGGATCTCAGGGCATCCCCAGCATATCTTTTCCTTCTTGTGCGAAATTGCCGAAGGCTGAAAACTCTCAAGATTAGCGACTGTTTCATGTCTGACCTGGTCGACCTGTTCCGTACAGCAGAAACACTACAAGACTTTGCTGGTGGTTCCTTTGATGATCAAGATCAAGGTGGGAATTATGCTAACTACTATTTCCCTCCTTCGGTACAGCGCTTGAGTTTGCTCTACATGGGAACAAATGAGATGCAGATATTATTTCCATATGGTGCCACACTCAAGAAGTTAGACCTTCAGTTTACATTCCTTACCACAGAGGATCACTGTCAATTAGTCCAGCGCTGCCCAAATCTAGAAGTTTTGGAGGTGAGGGATGTGATAGGAGACCGAGGGTTAGAAGTTGTTGCGCGGACCTGCAAGAAATTACAGCGACTCAGAGTCGAGAGAGGAGACGATGACCAAGGAGGTCTTGAGGACGAACAGGGTAGAGTGACACAAGTAGGATTGATGGCTGTAGCTGAAGGCTGTCCTGATTTGGAGTACTGGGCAGTACATGTGTCTGACATTACAAATGCAGCTCTTGAGGCCATTGGTGCGTTCAGCAAAAACCTGAACGATTTCCGACTTGTCCTGCTTGATAGAGAGGTGCATATAACTGAACTGCCCCTTGACAACGGGGTTCGGGCTTTGCTGAGAGGTTGCACCAAACTCCGGAGGTTTGCATTTTATGTGAGACCTGGAGCTCTATCAGATATTGGCCTTTCTTATGTTGGCGAATTTGGCAAGACTGTCCGCTACATGTTGCTTGGGAATGCTGGGGGATCTGATGATGGACTGCTGGCATTTGCACGAGGATGCCCAAGCTTGCAGAAATTGGAGCTAAGGAGTTGCTGCTTTAGTGAACGTGCATTGGCAGTTGCAGCCTTACAGCTGAAGTCACTCAGATATCTTTGGGTGCAGGGATACAAGGCATCTCCTACCGGCACCGATCTCATGGCAATGGTACGCCCCTTCTGGAACATTGAGTTTATTGCACCAAATCAAGATGAGCCTTGCCCAGAGGGTCAGGCACAGATTCTGGCATACTACTCTCTGGCTGGGGCAAGGACAGATTGTCCTCACTCAGTAATTCCCCTCTATCCGTCAGTCGGAAGCTAA

>AetCOI2 EMT11181

ATGGCGATGACTGCAGTTTCAGGCCGCTGGTCCCTTGCAGTCATCGACAAGAACATCTCTGAGTTTGACATCAACGCCCGTCACCCGTCTCCAGCACCACAACAGCAGGCGCCGAAGAAAAGAATGACGGATCACCTCCTCACCCGACCTCGACGCGGCTCCATCGCTGATATGCAGCTTTGCGGACCTCCAAGGCGGCTCACAAAAAGTGAAGCCCTTGCCGTTGAACGAATCAGACCGGGGCAACACCCCGGGCACGTCAGCGAACTCCAGATCTGGCACCCGACCACGACTAAGACGCCGAAGAGGGAAACCATACCTGTCATCCACGCAACACGAACCCAGCGCACGTTCCGTCTTCCAGATATCGTCGATGCAGACTACAATCTGCATCCGCTCCTGGACTGTAACCAATTCTACTACTCTTTATTTCTTTTGCGGGCGCAGACGCGCCGCGAGCTCTTCATCGGCAACTGCTACGCCGTCGACCCGCGCCGCGCCGTCGCCCGCTTCCGCGCCGTCGCCGCCGTCGTGCTCAAGGGCAAGCCGCGCTTCGCCGACTTCAGCCTCCTCCCCGACGGCTGGGGCGCCAACGTCAAGCCCTGGCTGGAGGCGCTCGGCCCCGCCTACCCGTGCCTCGAGCGCATCTGCCTCAAGCGCATGACCGTCACCGACGACGACCTCGGCCTCGTCGCGCGCTCCTTCCCGGGGTTCCAGGAGCTCTCCCTCGTCTGCTGCGACGGCTTCAGCACCCTCGGCCTCGCCGTCATCGCCGAGGGTTGCCGGCACCTGAGGGTTCTGGATCTGATCGAGGACTATGTGGATCAGGAGGACGAAGCAGTGGACTGGATCTCCAAGTTCCCAGTGTCAAACACATCCCTAGAGTCCCTCATGTTTGACTGCGTTGGTGCCCCATTCAACTTTGAGGCCCTGGAGGCCCTTGTGGCACGCTCGCCCTCTCTTCGCCGGCTGCGTGTGAACCACCACGTTTCAGTGGAGCAGCTCCGCCGTCTCATGGCGCGGGCTCCGCAGCTCACACACCTTGGCACTGGGGCCTTTCGTCCTGAGGCTCCACAAGGTGAGGGCATGTCGGTGTCTGAACTCGCGCCCTCTTTTGCAGCCTCGACGTCTATAGTCTGCCTCTCTGGATTCCAGGAGGTCAACCCTGAATACCTTCCAGCAATCTACCCAGTGTGTGGTAATCTCACCTCCCTTAACGTTAGCTTTGCAAGCCTGACTGCTGAGGATCTGACACCAGTCATTCGCCAATGCCACAAACTTCAGACATTCTGGGTAGGTCCATACTCTTATTTGTTTGCCATGGTTCTTGATACTGTGGGTGATGAAGGCCTCAGGGCTGTGGCCGAGACATGCTCTGATCTTCGTGAGCTGCGAGTATTTCCGCTGGATGCCACCGAGGACTCTGATGGGTCTGTGTCAGATGTTGGGCTTCAGGCTATATCCGAAGGTTGCCGAAAGCTTGAATCCATACTTTACTTTTGCCAGCGGATGACAAATGCTGCGGTCGTAGCTATGTCCAATAACTGCCCTGACCTTGTGGTGTTCCGTCTTTGCATCATGGGTCGCCACCGCCCTGATCGCATTACTGGGGAGCCCATGGATGACGGCTTTGGTGCGATAGTGAAGAACTGCAAGAAACTTACTAGGCTCTCTGTCTCTGGCCTGCTCACTGATAAGGCATTTGCATACATTGGGCAATATGGCAAACTAATAAAGACTTTGTCTCTTGCCTTCTCGGGGAACAGTGACCTGTCGCTTCAGTTTTTGTTCGAGGGATGCACTCGGTTACAGAAGCTAGAGGTCAGAGATAGCCCTTTCAGTGACAGGGGATTGCTTTGTGGTCTGGATTATTTCTACAATATGAGGTTCCTGTGGATGAATTCATGCAGGCTAACCATGAGGGGCTGTAGGGAGGTAGCTCAGCGGATGCCTAACTTGGTCGTTGAAGTAATGGAGGAGCAAAATGAGGACAAGGTGGAAACAGAGACCGTTGATAAGTTGTACCTGTACCGATCACTAGCAGGGCCAAGGGGTGATGCGCCGCCATTGGTGAAAATTTTGTAG

>AetCOI3 EMT22616

ATGTGCTACAAAAATTTCGTCAGCGCCGCAGCCGCCGAGGACGCGGCCGCGGAGGCTGACTACACTTCCGACCTCCCCGAGGAGCTCCTCGCCGTCGTATTCGGGCTCCTCGGCTCCGGCGACCGCAAGCGCTGCTCCCTGGTGTGCCGCCGCTGGCTCGCCACCGAGGCCTCCTCGCGCCTGCGCCTCGCCCTCGACGCGCGGGCGCCCCTCCTCGCCGCGGCCCCGGCCATTCTCGCGCGCTTCTCCGCCGTATCCAAGCTCGCGCTCAAGTGCGACCGCCGCGCGGAGAGCGTCGGCGATCCCGCGCTCGCGCTCGTCGCGCATCGCCTCGGCCCCGGCCTTCGCCGCCTCAAGCTCCGCTCGGTCCGTGCTGTCACCGACCACGGCGTCGCCGCCCTCGCAGCCGCGGCTATAAACCTCCGCAAGCTCTCAGTTGGGTCATGTACCTTCGGCGCCAAGGGGATCGAGGCAGTTCTCCGGTCCTGCCCCCAGCTCGAGGAGCTCTCTGTCAAGCGGCTGCGCGGCCTAGCTGACTCAGAGCCCATCACCGTCTCCAGCCCTCGTCTCCAGTCCCTGGCCCTTAAAGAGCTCTACAACGGGCAGTGCTTCTCCTGTTTAATCACGCGCTCCCCCAACCTCAAAACCCTCAAGATCATCCGCTGCTCCGGTGATTGGGACCCGGTTCTCCAGGCGATCCCACAGGGTGCCTTGCTAGCCGAGCTTCATCTCGAGAAACTGCAGGTCAGTGACCTTGGTGTGGCGGCGCTATGTGGGCTAGAGGTCCTGTACCTTGCCAAGGCGCCGGAGGTCACAGATGTTGGGTTGGCAGCACTTGCCACCAAGTCGCCACGTCTACGCAAGCTGCATGTAGATGGATGGAAGGCGAATAGGATTGGCGACCGTGGGCTTGCAACCGTGGCGCAGAAATGTGCTGCTTTGCAGGAATTAGTCCTCATTGGTGTGAATTTGACATCGGCGAGTCTTGAGTTGATTGCTGCCAACTGCCCCACTCTTGAGCGGCTTGCGCTTTGCGGGTCTGACACATTTGGGGATGCAGAGATATCTTGCGTGGCGACTAAGTGTGCTTCTCTGCGGAAGCTGTGCATCAAGGCATGCCCTGTGTCTGATGCCGGAATGGACAAGCTTGCAGCAGGCTGCCCACGCCTTGTCAAGGTGAAGGTGAAAAAGTGCCGCAGGGTGACGTTTGAGTGTGCTGAGCGGCTTCGTGCTAGTCGGCATGGCGCTCTTGCTGTGAATTTTGACACGCCAGGTGGTGCAGGCGAATTGCAAGATGCTAGTGTGGATGAGAGTGGTGTACTGGAGAATGCAGGGAGTGATGTGTGGCAGACCATCAGGGTGGAAAGCATGGATGAGTACTTTGATTCCAAGGAGCTTGTCGGTTTCCATGTTTCGCAGACGTCCGCGTGGGAGCTCCCATAA

>AetCOI4 EMT31633

ATGGGCGGGCGGGCGGCCGTCCCGGGGACATTTTGGTCATCTTCCTTGGTCTCAGGTGCTACCTCCACCTCGGCCGAGGGCAGGCGCTACTCCGACAGGGAGGCTGCCGCGCTGGCCGCGGCCTCCGGGCTGAGGGAGCTGAATCTGGAAAAGTGCCTGGGCGTCACTGACATGGGACTCGCCAAGGTAGCCGTGGGGTGCCCCAGACTGGAGAATCTGAGCTTCAAGTGGTGCCGTGAAATCTCTGACATCGGCGTCGATCTGCTTGTGAAGAAGTGCCGCGAGCTCCGCAGCCTTGACATCTCCTACCTAAAGATTAGACTTGACTCATTTTATGGAGTGCACTCATTATATCTTGGTGCAATTTATAATTGTATTTTGGTCAACAACAACAAAGCCTTTTGTCCCAAGCAATTTGGGGTGAGCAATGAGTCCCTTAGATCAATATCAACACTTGAGAAGCTTGAGGAGTTGGCCATGGTTGCTTGCTCATGTATAGACGATGAAGGCCTGGAATTGCTTAGCAGAGGAAGTAATTCATTGCAGGGATACTCTTGCATATTATCATGTCTTCCGTCACCTGAAATCATATGGCTTACTCTCTCGTCAAACATGAGTGTTGATGTGTCAAGATGTGATCATGTGACTTCCCAGGGGTTAGCTTCACTGATAGATGGTCACAGTTTTCTCCAGAAGCTATATGCCGCAGATAGTTTGCATGAGATTGGACAGGATTTTCTATCCAAGTTAGTAACACTGAAGGCAACCTTGGCCGTGTTGAGACTTGACGGCTTTGAAGTGTCATCCTCTCTTCTTTCAGCGATTGGTGAAGGTTGTACCAACTTGGTTGAGATTGGACTAAGCAAATGCAACGGTGTTACAGATGAAGGCATCTCTTCGCTTGTAGCTTGCTGTAGCTACCTAAGGACAATTGATCTCACATGCTGCAATCTAGTCACAAACAATTCCCTTGATTCAATAGCTGACAACTGTAAGCTGCTTGAATGCCTCCGGTTGGAGTCCTGCTCTTCAATAAACGAGAAAGGACTAGAGAGAATTGCGAGCTGTTGCCCCAATCTAAAGGAGATAGATCTCACTGATTGTGGAGTGAATGACGAAGGCATCGGAATTTCTTCGGTTGTCACTGGCTGTAAGAGCCTGGTAGAACTTGACTTGAAGCGCTGCTATTCTGTCGATGATTCTGGCCTATGGGCTCTTGCTCGATATGCTCTAAACCTTAGACAGCTTACCATATCATACTGCCAAGTTACTGGCCTAGGCTTGTGCCACCTGCTTAGCTCCCTGAGGTGCCTCCAGGACGTGAAGATGGTGCACCTCTCGTGGGTTTCCATAGAAGGGTTCGAGATGGCTCTGAGAGCCGCTTGTGGGAGGCTGAAGAAGCTGAAGATACTCAGCGGTTTGAAGACCGTGCTATCCCCTGATCTGCTCCAGCTGCTGCAAGCCTGCGGCTGCCGCATCAGATGGGTCAACAAGCCTCTTGTCTACAAGGATGCCATCTGA

>AetCOI5 EMT03750

ATGGAGGATCTCCCAGAGCCACTGCTTGCAGAGATCGTCAAGAGGATTACCAAGACAAGGGATCTTAATTCTCTTTCCCTCGTGTCGAAGCAGCTCTGCACCGTTGAGGCGGAGCACAGGGATGCTATCCGTGTTGGCTGTGGACTCAACCCTTCTACAGAAGCCTTGGTATCGTTGTTCTCCCGGTTCCCCAATTTGGCGAAAGTAGAGATAAACTATTCCGGATGGTGGCCCAGTAACGGGAACCAGTTGAACAACCAAGGCCTCTGTGTTCTATCATCTCACTGCCCCTCACTGTCCGATCTTTCTTTAAGCTTCTGCTCTTACATTGGTGACTCTGGTCTTGGTTATCTAGCTGATTGCAAAAAATTGAGGTCGCTCAGGCTGAACTGTGCACCAGCAGTAACTTCTACTGGGATTTTTCGTGTGGCGGTTGGTTGCAGGCATCTTTCGGTTCTTCACCTTGTTGACTGTATGGCAGTAGACAGCAGGGAGTGGCTGGAGTACCTTGGGAGATATGGATCATTGGTAGAACTTGTTGTAAAGGATTGCGATGGAATCAGCCAGTATGACCTCTTAAAGTTTGGCCCAGGCTGGGAGAATCTCCAAAAATTTGTGTTTGAGATTAATGGAAACTACTGGATGTCAGGGGCGTCTGACCCCTCCTACGTGTCTGGCTACCCATATAGGTATGACATCTGCTGTGATAGCTTGAAGGATCTTAGGTTAGCTCATATTATAACCGAGCCAGAAACTGGACTTCGTTTTCTCCTGGGGAAGTGCAAAGCATTGGAGACGCTTTACCTAGAGTATGTGATTGGTCTAAAAGAGAATGAGACGATTGCGCTATTCCAGAGGTGCAGCAACCTTAAAACCATCTCACTTCGGTTCATGCCTCTGCGGTGTGAAGATTATGAGTTTAGGACGCCATTGACTGATGTTAGCCTTAAGGCTCTAGCTCTCAGCTGCCCTATGCTTCAGGTTGTAGAGCTCACATTCACATTTTGCGAACCCATGTATCCAACAGAAATAGGTTTCACACAAGAGGGTATTGTGTCGCTCGTCCAATCTTGTCCAGTCCGCACTCTTTTGCTAAATGGTGCCAGCATTTTGTATGACGAGGGGTTGAAGTGCCTCTCATCCTCACAGTTCCTGGAGAAGCTTGAGCTCGTGGATTGCAGGTCTATAACCGATGCTGGTATGAGTTTCATTATACTGGCTCCTTGCTTCAGCAATCTCACCCTCCGCAAATGCAAGAAAGTGACAGACAATGGGATGGCTGAGCTGGCACGTTCACAGAAGTTGGAGTCACTGACCGTTATAGGCTGCTGCCAGATCTCTCAGGAGGGTGTGCAAGGGGCTGCCAAATCAGTTCACTACTCTGCAGAGACTGAAAGCCACGGCAGTCTAAAAGGAATGAACACGGACAGGGACATGAACAGGAAAAGACGCCGGTCGCCCTGA

>AtCOI1 AT3G62980.1

ATGCAGAAGCGAATAGCCTTGTCGTTTCCAGAAGAGGTACTAGAGCATGTGTTCTCGTTTATTCAGCTGGATAAGGATAGGAACTCAGTCTCTCTGGTGTGCAAGTCATGGTACGAGATCGAGCGGTGGTGCAGGAGGAAAGTCTTCATCGGGAACTGCTACGCCGTGAGTCCAGCGACGGTGATTAGGAGGTTCCCGAAAGTGAGATCCGTGGAGCTTAAAGGAAAACCTCACTTTGCTGACTTTAATTTGGTACCTGACGGATGGGGAGGTTACGTGTATCCATGGATTGAGGCCATGTCTTCGTCTTACACGTGGCTTGAAGAGATAAGGCTGAAGAGGATGGTGGTCACCGACGATTGCTTGGAGCTCATAGCCAAGTCTTTTAAGAATTTTAAGGTTCTTGTGCTTTCTTCCTGCGAAGGCTTCTCCACCGATGGTCTCGCTGCTATCGCTGCCACTTGCAGGAATCTGAAAGAGCTTGACTTACGAGAGAGTGATGTTGACGACGTTAGTGGCCACTGGCTTAGCCATTTCCCAGATACATACACTTCTTTGGTATCACTCAATATATCTTGCTTAGCATCTGAGGTCAGTTTCTCTGCTCTGGAAAGGCTGGTGACTAGGTGTCCCAATCTCAAGTCTCTCAAGCTTAACCGAGCTGTTCCACTTGAAAAATTGGCTACTTTACTTCAAAGAGCACCTCAATTGGAGGAATTGGGCACTGGTGGGTACACTGCAGAAGTGCGACCAGATGTTTACTCTGGTTTATCTGTAGCGCTCTCTGGGTGCAAGGAATTGAGGTGCTTATCTGGATTTTGGGATGCTGTTCCTGCCTATCTTCCAGCAGTTTATTCGGTTTGCAGTCGGCTTACAACTTTGAATCTGAGTTATGCAACAGTCCAGAGCTATGATCTTGTCAAGCTTCTTTGTCAATGCCCTAAACTGCAGCGCCTCTGGGTGCTTGACTACATCGAGGATGCTGGTCTTGAGGTGCTTGCTTCAACCTGCAAGGACCTACGCGAGCTGAGAGTGTTTCCGTCCGAGCCTTTTGTCATGGAACCAAATGTGGCATTGACGGAACAGGGGCTTGTCTCCGTCTCCATGGGCTGTCCAAAACTCGAGTCGGTTCTCTACTTCTGCCGTCAAATGACCAATGCTGCATTGATAACCATTGCTAGGAACCGTCCCAACATGACTCGCTTCCGTTTGTGCATCATTGAGCCAAAAGCCCCAGACTATCTGACTCTAGAGCCACTGGATATTGGATTTGGAGCCATAGTAGAGCACTGCAAGGATCTCCGTCGCCTCTCTCTATCTGGCCTCTTGACCGACAAGGTTTTTGAATACATTGGGACATATGCCAAGAAGATGGAAATGCTCTCAGTGGCATTTGCAGGAGACAGTGACTTAGGCATGCATCATGTTTTGTCCGGGTGCGATAGCTTGAGGAAACTAGAGATAAGGGACTGCCCGTTTGGAGACAAGGCGCTTTTGGCCAATGCTTCAAAGCTGGAGACAATGCGATCCCTTTGGATGTCTTCTTGTTCCGTGAGTTTTGGAGCCTGCAAGTTACTAGGACAGAAGATGCCAAAGCTGAATGTGGAAGTCATCGATGAACGGGGTGCACCGGACTCGAGACCAGAGAGCTGCCCTGTTGAGAGAGTCTTCATATACCGAACAGTGGCTGGTCCTCGATTTGACATGCCTGGCTTCGTCTGGAACATGGACCAAGACTCAACAATGAGGTTTTCCAGGCAAATCATTACTACTAACGGATTATAA

>AtCOI2 AT1G12820.1

ATGAATTATTTCCCAGACGAGGTTATAGAGCACGTGTTTGACTTCGTAGCTTCTCACAAAGACAGGAACTCGATATCTCTGGTCTGCAAATCATGGCACAAGATCGAGAGGTTTAGTAGGAAGGAAGTGTTCATCGGAAACTGCTACGCGATTAACCCGGAGAGGTTGATCAGGAGGTTTCCATGTCTCAAATCCTTAACTTTAAAAGGGAAGCCTCATTTTGCAGACTTCAACTTGGTTCCTCATGAATGGGGAGGTTTCGTGCATCCTTGGATTGAAGCTTTGGCTAGAAGCCGTGTGGGACTTGAGGAGCTGAGGTTGAAGCGGATGGTTGTAACAGATGAAAGCTTGGACCTTCTTTCACGTTCTTTTGCAAATTTCAAGTCTTTGGTTCTTGTTAGCTGTGAAGGGTTTACCACTGATGGCTTAGCTTCCATTGCCGCTAATTGCAGGCATCTTCGTGAGCTGGACTTGCAAGAGAATGAGATTGATGATCATAGAGGTCAATGGCTGAACTGTTTTCCAGATAGCTGCACTACTCTTATGTCGTTGAATTTCGCTTGCCTTAAAGGAGAGACCAATGTTGCTGCTTTAGAAAGGCTTGTTGCTAGGTCACCAAACCTGAAGAGCTTGAAGTTAAACCGTGCAGTACCGCTTGACGCACTCGCAAGGTTAATGAGTTGTGCGCCGCAGCTAGTGGACTTAGGAGTAGGGTCTTATGAGAATGAGCCAGATCCTGAATCTTTTGCAAAACTCATGACTGCCATTAAGAAATACACATCGTTAAGGAGCTTGTCTGGCTTTTTAGAGGTTGCTCCACTCTGCCTCCCAGCGTTCTACCCAATTTGCCAAAACCTTATCTCTTTGAACCTCAGCTATGCAGCTGAAATCCAAGGCAACCACCTCATTAAGCTTATTCAGCTTTGCAAGAGACTTCAACGATTATGGATATTGGATAGTATTGGTGACAAAGGACTTGCGGTTGTCGCTGCCACATGTAAAGAGTTACAAGAGCTTAGAGTTTTTCCCTCTGATGTACATGGTGAAGAAGATAACAACGCATCTGTGACTGAGGTTGGACTAGTCGCCATTTCCGCAGGTTGCCCTAAACTTCATTCGATTCTGTACTTCTGCAAACAGATGACAAACGCAGCGCTCATAGCCGTGGCCAAAAACTGTCCAAACTTCATCCGGTTCAGGCTATGCATTCTCGAGCCACACAAACCTGACCACATTACATTTCAATCACTGGACGAGGGCTTTGGTGCAATCGTACAAGCTTGCAAGGGTCTAAGACGGCTCTCTGTCTCCGGTCTCTTAACCGATCAAGTCTTTCTCTACATCGGTATGTACGCGGAACAGCTCGAGATGCTTTCGATAGCTTTTGCGGGGGACACTGACAAAGGAATGCTCTATGTGTTGAATGGATGCAAAAAAATGAGGAAGCTGGAGATAAGGGACAGTCCTTTTGGGAACGCTGCGCTTCTTGCTGACGTGGGTAGGTACGAAACAATGCGATCCCTTTGGATGTCGTCTTGTGAAGTAACACTCGGTGGCTGCAAGAGGCTCGCGCAGAATTCGCCACGGCTTAACGTAGAGATCATCAACGAGAATGAGAATAATGGGATGGAACAGAATGAAGAAGATGAAAGAGAGAAGGTTGATAAACTTTACCTCTACCGAACAGTGGTTGGGACTAGAAAAGATGCACCACCATATGTTAGGATTCTTTAG

>AtCOI3 AT5G49980.1

ATGACACAAGATCGCTCAGAAATGTCTGAAGATGACGATGACCAACAATCTCCACCGTTGGATCTACCCTCTACCGCCATAGCTGATCCTTGCTCATCTTCCTCTTCACCAAACAAATCTCGTAACTGTATCTCAAATTCTCAAACTTTCCCTGACCATGTTCTCGAAAACGTACTTGAGAACGTTCTTCAGTTCCTAGATTCAAGATGTGACCGTAACGCTGCTTCTCTAGTTTGCAAATCTTGGTGGCGTGTTGAAGCTTTGACTCGATCTGAGGTTTTTATTGGTAACTGTTACGCTCTTTCTCCGGCGAGGTTGACTCAGAGATTCAAGCGTGTTAGGTCTCTTGTGCTGAAAGGGAAACCTAGGTTTGCTGATTTCAATCTCATGCCTCCTGATTGGGGTGCTAATTTTGCTCCTTGGGTTTCTACTATGGCTCAAGCTTATCCTTGTCTTGAGAAAGTTGATTTGAAGAGGATGTTTGTTACTGATGATGATTTAGCTCTTCTTGCTGACTCTTTTCCTGGGTTTAAAGAGCTTATCTTGGTTTGTTGTGAAGGTTTTGGTACTAGTGGTATCTCTATTGTTGCCAACAAGTGCAGAAAGCTGAAAGTGCTTGATTTGATTGAGTCTGAGGTCACGGATGATGAAGTGGATTGGATCTCTTGTTTCCCTGAGGATGTAACTTGTTTGGAGTCTTTAGCTTTTGACTGTGTGGAAGCTCCTATCAATTTTAAGGCGCTTGAGGGTCTTGTTGCTAGGTCACCGTTCTTGAAGAAACTTAGGCTAAACAGGTTTGTGTCTCTTGTGGAGCTACATCGTCTGCTACTTGGAGCTCCACAGCTTACTAGTCTTGGGACTGGTTCATTTAGCCATGATGAGGAACCTCAGAGTGAGCAAGAACCAGATTATGCTGCTGCATTTCGTGCTTGTAAATCTGTAGTTTGCTTGTCAGGGTTTAGAGAGTTGATGCCGGAGTATCTTCCAGCTATCTTTCCGGTGTGCGCTAATCTCACCTCCCTGAACTTCAGTTATGCTAACATTTCTCCTGACATGTTCAAGCCCATCATACTCAATTGCCACAAACTCCAGGTGTTCTGGGCCCTTGATTCAATATGTGATGAAGGACTACAGGCAGTTGCAGCCACTTGCAAGGAACTCCGTGAACTCAGGATCTTCCCTTTTGATCCTCGGGAAGACAGTGAAGGTCCTGTCTCTGAATTAGGCCTCCAAGCAATCTCCGAGGGTTGTAGGAAACTAGAATCTATTCTCTACTTTTGCCAGCGCATGACTAATGCCGCTGTGATAGCCATGTCAGAGAACTGTCCAGAGCTTACTGTGTTTAGGCTGTGCATAATGGGTCGACATAGGCCTGACCATGTAACAGGAAAGCCTATGGACGAGGGATTTGGTGCCATTGTTAAAAACTGCAAGAAGCTAACTCGCCTTGCAGTGTCGGGATTGCTGACAGATCAAGCTTTTAGGTATATGGGTGAGTATGGGAAATTGGTCCGTACGCTTTCAGTAGCTTTTGCAGGGGACAGTGACATGGCTCTGAGACATGTCCTAGAAGGTTGCCCTAGACTGCAGAAACTTGAGATAAGGGACAGTCCCTTTGGAGATGTTGCATTACGGTCTGGTATGCATCGCTATTACAACATGAGGTTTGTTTGGATGTCAGCATGTAGCTTGTCTAAGGGATGCTGCAAGGATATTGCACGAGCAATGCCGAATCTAGTTGTGGAAGTAATTGGATCGGATGATGATGATGACAATAGGGATTATGTCGAGACTTTATACATGTATCGGTCTCTTGATGGTCCAAGGAATGATGCACCAAAGTTCGTCACGATTTTATAG

>AtCOI4 AT4G24390.1

ATGACAGAAGAAGATAGCTCAGCTAAAATGTCAGAGGATGTTGAGAAATATCTCAACTTAAATCCACCTTGCTCCTCCTCCTCCTCTTCTTCCTCCGCCGCTACATTCACGAACAAGTCTCGAAATTTCAAATCTTCTCCCCCGCCGTGTCCAGATCATGTCCTTGAGAACGTTTTAGAGAACGTGCTTCAGTTCCTCACTTCCAGATGCGATCGCAACGCAGTCTCATTGGTCTGCAGATCGTGGTATCGCGTCGAGGCTCAGACTCGATTAGAGGTTTTTATTGGAAACTGTTACTCGCTCTCTCCTGCTCGGCTTATTCACCGGTTCAAGCGTGTTAGGTCTCTTGTGCTTAAAGGGAAACCTAGGTTTGCTGATTTTAATCTCATGCCTCCTAATTGGGGAGCTCAATTCTCTCCTTGGGTTGCTGCTACAGCTAAGGCTTATCCTTGGCTCGAGAAGGTTCATTTGAAGCGTATGTTTGTTACGGATGATGATTTGGCTCTTCTTGCTGAGTCGTTTCCTGGGTTCAAAGAGCTTACTTTGGTCTGCTGTGAAGGTTTTGGGACTAGTGGTATTGCTATTGTTGCTAACAAATGCAGGCAGCTAAAGGTCCTTGATTTGATGGAGTCAGAAGTCACAGATGATGAGTTGGATTGGATTTCTTGTTTTCCTGAGGGTGAAACTCATCTGGAGTCTTTGTCTTTTGACTGTGTTGAATCCCCTATCAATTTCAAGGCATTGGAGGAGCTCGTGGTTAGGTCACCATTCTTGAAGAAACTTAGAACGAACAGGTTTGTTTCCCTTGAAGAGCTGCATCGACTAATGGTTCGAGCGCCGCAGTTAACGAGTCTTGGGACGGGGTCATTTAGTCCAGACAATGTGCCTCAGGGAGAACAACAACCGGATTATGCAGCTGCTTTTCGTGCTTGTAAATCCATAGTTTGTCTCTCAGGATTCAGGGAATTTAGACCGGAATACCTCCTAGCCATCTCTTCAGTTTGTGCTAATCTCACCTCTCTTAACTTCAGTTATGCTAACATTTCTCCTCACATGCTCAAGCCCATCATAAGCAACTGTCACAATATCCGAGTCTTCTGGGCTCTTGACTCGATACGTGATGAAGGACTACAGGCAGTGGCTGCCACATGCAAGGAGCTCCGTGAGCTTCGGATTTTCCCTTTTGATCCTCGTGAAGACAGTGAAGGTCCTGTCTCGGGAGTAGGCCTCCAAGCAATTTCAGAGGGCTGTAGGAAACTGGAATCTATCCTGTACTTTTGCCAGAATATGACCAATGGAGCTGTGACAGCCATGTCGGAGAACTGCCCGCAGCTTACTGTGTTTAGACTTTGCATAATGGGTCGCCATAGGCCTGACCACGTGACAGGAAAGCCAATGGACGATGGATTTGGTGCCATTGTTAAAAACTGCAAGAAGCTAACCCGACTTGCAGTATCAGGGTTACTAACAGATGAAGCTTTTAGCTATATAGGAGAATATGGGAAATTGATCCGTACGCTATCTGTAGCGTTTGCTGGGAACAGTGACAAGGCTCTGAGATACGTTCTTGAGGGTTGTCCTAAACTACAAAAGCTTGAGATCAGGGACAGTCCCTTTGGAGATGTTGGATTGCGCTCTGGTATGCATCGGTATTCCAATATGAGGTTTGTTTGGTTGTCGTCATGTCTCATATCCCGTGGAGGCTGCAGGGGTGTTTCTCATGCTCTGCCTAATGTAGTCGTGGAAGTATTTGGAGCCGATGGTGATGATGACGAAGACACTGTCACTGGGGATTATGTTGAGACATTGTACTTGTATCGATCCCTTGATGGCCCAAGGAAGGATGCTCCAAAGTTTGTAACAATTTTATGA

>BdCOI1 BRADI2G23730.2

ATGGGCGGGGAGGCCCCGGAGCCGCGGCGCCTGAGCCGCGCGCTCAGCTTAGATGGCAGCGTCGTCCCGGAAGAGGCGCTGCACCTGGTGATGGGATACGTCGACCACCCGCGCGACCGGGAGGCGGCGTCGCTCGTGTGCCGCCGGTGGCACCGCATCGACGCGCTCACCCGGAAGCATGTCACCGTGCCCTTCTGCTACGCGGCGTCCCCGGCGCGCCTGCTCGCGCGGTTCCCGCGCCTCGAGTCGCTCGCGGTCAAGGGGAAGCCTCGCGCCGCCATGTACGGCCTCATCCCCGACGACTGGGGCGCGTACGCTCGTCCCTGGGTCGCCGAGCTCGCTGCGCCGCTCGAGTGCCTCAAGACGCTCCACCTGCGGCGCATGGTCGTCACCGACGACGACCTCGCTACTCTTGTCCGCGCCCGCGGCCACATGCTGCAGGAGCTCAAGCTCGACAAGTGCTCTGGCTTCTCCACCGACGCCCTCCGCCTCGTCGCTCGCTCCTGCAGCAGATCACTGAGAACTTTGTTTCTGGAAGAATGTTCAATTACTGATAATGGCACTGAATGGCTCCATGACCTTGCTGTTAACAATCCTGTACTGGTGACCTTGAACTTCTATATGACTTACCTCAGAGTGGTGCCAGCTGACCTCGAGCTTCTTGCCAAGAACTGCAAGTCATTAATTTCATTGAAGATTAGCGACTGTGACCTTTCTGATTTAATTGGATTTTTCCAAATGGCCACATCATTGGAAGAATTTGCTGGAGCGGAATTTAATGAGCAAGGGGAACTCACAAAGTATGGAAATGTTAAATTTCCTTCAAGGCTTTGCTCCTTAGGACTTACTTGCTTGGGGACTAATGAGATGCATATCATCTTTCCCTTTTCCACTGTACTCAAGAAGCTGGATTTGCAGTACACTTTTCTCACCACTGAAGATCATTGCCAGCTCATTGCAAAATGTCCCAACTTGTTAGTTCTCGCGGTGAGGAATGTGATTGGAGACAGAGGATTAGGGGTTGTCGCAGACACATGCAAGAAGCTACAAAGGCTCAGAGTTGAGCGAGGGGATGATGATCCTGGTCTGCAAGAAGAGCAAGGAGGAGTTTCTCAGGTAGGCCTGACAGCTGTAGCCGTTGGTTGCCGTGAACTGGACTACATAGCTGCCTATGTGTCTGATATCACAAATGGGGCCCTGGAATCTATTGGGACTTTCTGCAAAAATCTGTGTGACTTTCGGCTTGTCCTGCTTGATAGACAAGAGAGGATAACAGAATTGCCACTAGACAATGGTGTTCGTGCGCTGCTGAGGGGCTGTGCTAAGCTTCGGAGATTCGCGCTATACTTGAGACCAGGGGGGCTTTCAGATGTAGGCCTTGGCTATATTGGACAGCACAGTGGAACTATCCAGTACATGCTTCTGGGTAATGTTGGAGACACAGACAATGGACTGCTCCTTTTTGCGTCTGGGTGTGTGAACCTACGGAAGCTTGAATTAAGGAGCTGTTGCTTCAGTGAGCGGGCTTTGGCTCTTGCTATGCTACAAATGCCTTCTCTGAGGTACGTATGGGTACAAGGTTACAAAGCCTCCCAAACTGGTCGCGACCTCATGCTCATGGCCAGACCTTTCTGGAACATAGAATTTACACCTCCCAGTCCTGAGAATGCAAATAGGCTGACGGTAGATGGGGAGCCTTGTGTAGATCGTTATGCTCAGATTCTTGCATACTACTCCCTTGCCGGAAAGAGGTCGGACTGCCCGCAATCTGTGGTTCCTTTGTATCCTGCGTGA

>BdCOI2 BRADI3G58320.1

ATGAGCACTCCCCGCTCCTCCGCCTCCTCCCCCGATCCGATCCCCCACAAATCCGTAACCCTAGCACCCTCCCTCGGCATGCGCGATCCGGAGGAGGACTCGGACTCGACGCCGTCGCGGATGTCGGAGGACGACGACTGCTCCGGCGGGGGAGGCGGCGGCGGGTGGACGCCGGATCTGAGGGGCGGCGGCGGCAGGTGGGCGCCGCCGGACCAGGTGCTGGAGATCGTGCTCGAGAGCGTGCTCGAGTTCCTCACCGCTGCGCGCGATCGGAACGCCGCCTCGCTCGTCTGCCGCTCCTGGTACCGCGCCGAGGCCCAGACGCGCAGGGAGCTCTTCATCGGCAACTGCTACGCCGTCTCGCCGCGCCGCGCCGTCGAGCGGTTCGGGGGCGTGCGGGCCGTCGTTCTCAAGGGGAAACCGCGCTTCGCGGACTTCAGCCTCGTGCCCCACGGCTGGGGCGCCTACGTTTCGCCCTGGTTCGCCGCGCTTGGCCCCGCGTATCCCCGCCTCGAGCGCATCTGCCTCAAGCGGATGACCGTCTCCGACGACGAGCTCGCGCTCATTCCCAGGTCGTTCCCGCTCTTCAAGGAGCTGTCGCTGGTGTGCTGCGACGGATTTAGCACCCGCGGCCTCGCCATCATCGCCGAGGGATGCAGGCATCTCCGAGTGCTGGATCTGACTGAAGATTATTTTCATGAGGAGGAGAACGAGGTAGTGGATTGGATCTCCAAGTTTCCAGAGAGCAACACGTCGCTGGAGTCGCTTGTATTTGATTGTGTTAGTGTACCATTCAACTTCGAGGCCCTCGAGGCACTTGTTGCACGGTCACCAGCTCTGCGTCGGCTGCGTGTGAATGACCATGTATCGATAGAGCAGCTGCGTTGTCTCATGGCAAGGGCACCCCGTCTCACGCACCTTGGTACTGGTTCATTCCGATCTGAACCAGGCTCTGGCGGCACATCATCTGTGTCTGAGCTCGCCACCTCTTTTGCAGCATCGAGGTCACTAGTTTGTTTGTCAGGTTTCTTGGATGTCAATGCAGAATACCTCCCAGCAATTTACCCAGTTTGTGCCAATCTCACATCCCTCAACTTTAGCTTTGCGAGCCTAACTGCTGAGGAGATCATACCAGTTATTAACCACTGCGTCAGTCTTCGCACTTTCTGGGTTCTTGATACAGTGGGTGATGAAGGCCTTCGGGCTGTGGCTGAAACATGCTCAGATCTCCGTGAGCTGCGTGTTTTTCCTCTCGATGCCACAGAGGATTCTGAGGGCTCAGTCTCGGATATTGGTCTTCAAGCAATCTCAGAAGGCTGCCGGAAGCTCGAATCGATTCTCTACTTCTGCCAGCGTATGACAAATGCAGCAGTAATCGCTATGTCTGAGAACTGCCCTAACCTTGTGGTGTTCCGCCTCTGTATTATGGGCCGTCACCGCCCTGATCGGATTACAGGGGAGCCCATGGATGAGGGTTTTGGGGCAATTGTGATGAACTGCAAGAAGCTCACTAGACTTTCAGTCTCTGGCCTGCTCACTGATAAAGCGTTTGCATACATCGGGAAGTATGGAAAACTAATAAAGACTCTGTCTATTGCCTTTGCCGGGAATAGTGACATGTCTCTTCAACATGTCTTTGAGGGATGCACTAGGTTGCAGAAGCTTGAGGTCAGAGATAGCCCTTTCGGCGACAAGGGGTTGCTCTCTGGCATGAACTATTTTTACAACATGAGGTTCTTTTGGATGAACTCCTGCAGGCTAACCGCGAAGGGCTGTCGAGATGTAGCTCAGCAAATGCCAAATTTGGTGGTGGAAGTAATGAAGGAGCATCCTGAAGATGAAGGGGAGACAGATACCGTTGATAAGCTGTACCTGTATCGATCACTTGCAGGACCAAGGAATGATGCTCCATCATTTGTGAACATCTTGTAG

>BdCOI3 BRADI4G05157.1

ATGAGCGAGGATGCGCAGAGGTACGGCGGCGGCGGCGGCAGCGGCGGGGGCGGGAGCAGCGGCGGCATCGGGGCGCTGTCCCTGGATCTGCTCTGCCAGGTGCTCGACCGCGTGCAGGAGCGGCGGGACCGCAAGGCGTGCCGCCTCGTCAGCCGCGCCTTCGCGCGCGCCGAGGCCGCGCACCGCCGCGCGCTGCGGGTGCTCCGGCGGGAGCCGCTCCCTCGCCTGCTCCGCGCGTTCCCGGCGCTCGAGCTGCTCGACCTCTCCGCCTGCGCCTCGCTCGACGACGCGTCTCTCGCGGCCGCCGTGGCCGGCGCCGACCTCGGCGCCGTCCGCCAGGTCTGCCTCGCGCGGGCCAGCGGGGTCGGGTGGCGCGGCCTGGACGCCCTCGTCGCCGCCTGCCCCAGGCTGGAGGCAGTCGACCTGTCGCACTGCGTCGGCGCTGGGGACAGGGAGGCCGCGGCTCTGGCGGCGGCCGCTGGGCTGAGGGAGCTGAATCTGGAGAAGTGCCTCGGCGTCACTGACATGGGCCTCGCCAAGGTAGCTGTGGGCTGCCCCAAGCTTGAGAAGCTGAGCTTCAAGTGGTGCCGTGAGATTTCCGACATCGGTGTTGATCTGCTCGTCAAGAAGTGCCGCGAGCTCCGCAACCTTGATATCTCCTACCTAGAGGTGAGCAATGAGTCTCTTAGATCAATATCGACTCTTGAAAAGCTAGAGGAGTTGTCGATGGTTGGTTGCTTATGTATAGATGACAAAGGCCTGGAATTGCTAAGCAGAGGAAGTAATTCACTGCAGAGTGTTGATGTGTCAAGATGTGATCACGTGACTTCCGAAGGCTTAGCTTCACTCATAGATGGTCACAGTTTTCTCCAGAAGCTAAATGCTGCTGATAGTTTGCATGAGATTGGGCAGAATTTTCTATCCAAGCTAGCAACACTGAAGGAAACATTGACAATGTTGAGACTTGACGGCTTTGAAGTCTCATCCTCTCTTCTTTTAGCGATTGCTGAAGGCTGTAACAACTTGGTTGAGGTTGGACTTAGCAAATGCAATGGTGTTACAGATGAAGGCATCTCTTCGCTTGTAGCTCGGTGTGGCTACCTAAGGAAAATTGATCTCACATGCTGTAATCTACTCACTGACAATGCCCTTGTTTCAATAGCTGACAACTGTAAGATGCTTGAATGCCTCCTGTTGGAGTCCTGCTCTTCATTAAGTGAGAAAGGACTAGAGAGAATCGCTACCTGCTGCCCCAATCTTAGTGAGATAGATCTTACTGACTGTGGAGTGAATGATGCAGCGCTGCAGCATTTGGCTAAGTGCTCTGAACTGCTGATATTGAAGTTAGGCTTGTGCTCAAGTATTTCTGACAAAGGCCTTGGTTTTATTAGTTCGAAGTGTGTAAAACTCACAGAAGTTGACCTCTATCGCTGCAACTCTATTACCGACGATGGGTTAGCAACTTTAGCAAAGGGCTGCAAAAAGATTAAGATGCTGAACCTGTGCTACTGCAACAAGATTACTGATGGTGGTTTGAGCCACCTGGGCTCTCTGGAGGAGCTTACAAACCTTGAGCTGAGGTGCCTGGTCCGCATTACAGGTATTGGAATCTCGTCAGTTGCCATTGGTTGCAAGAGCCTGGTAGAAATTGACTTGAAGCGTTGCTATTCTGTTGATGATTCTGGCTTGTGGGCTCTCGCACGATATGCCCTAAACCTTAGACAGCTTACCATATCATACTGCCAAGTTACTGGCTTGGGATTGTGCCACCTGCTGAGCTCCCTGAGGTGCCTCCAGGACGTGAAGATGGTGCACCTCTCATGGGTCTCCATAGAAGGGTTTGAGATGGCACTGAGAGCTGCTTGCGGCAGGCTGAAGAAGCTGAAGATGCTTAGCAGTCTGAAGTCTGTGCTGTCCCCTGAGCTGCTCCAGCTGCTGCAGGCCTGTGGCTGCCGCATCAGATGGGTCAACAAGCCTCTCGTCTACAAGGATGGCATCTGA

>BdCOI4 BRADI4G06170.1

ATGGAGCCTTCTCCCGTGACCGACAGCGAAGAAACCGAAGGCGTTCGTTGTGGATCGGGCTCGTCCGTTCGCCGGCCGTCGCGTCGTCCCCAGCTGGCCGAGGGGGCGAGGGTTCTGACGGAGAACGCGACACGGGTTGCTGCGGCGAATCCAAGGAGAGCCAGGCTCACGGTGTTGATGCCCGTGCGATCTCCGATTCTCAGGATGATGCCTCTCAGGGCGAGGTCTGCGTCAGCGACATTTCGGACATCCCCGTCAGCAAAAGCCCTGCGAGCTCCCGTAGCCATCTCAACAATCCAGCGCTTTGTATATTCGCCGAACCTTCAGATTACCCAACCAGCACAGCACCATTTCTCATTGGAGGATCTCCCAGATGCAATGTTAACAGAGATCATCAAGAGGATTACCCTGACAAGTGATCTGAATTCTTTTTCCCTTGTGTCGAAGCGACTCTACACCATTGAGGCAGATCAGAGGGGTGCTATTCGTGTTGGTTGCAACCTTTGCCCTGCTACAGAAGCCTTGGCATCACTCTGCACACGGTTCCCCAATCTCTGGAAAGTGGAAATTGATTACTCTGGTTGGACGCCAGGACATGGGAATCAGTTGGACAACCAAGGGTTCCTTGAGTTTTCATCTCGCTGTCCCTCGCTTACTGATCTGACCTTGAGCTTCTGCTCACGCATCCATGACTCTGGTCTTGGTTGTCTAGATTATTGCAAGAAATTGATGTCCCTTAGGCTGAAATCCGCACCAAAAATAACTTCACGTGGGCTTCTCTCGGTTGTAGTTAGTTGCAAGAGTCTATCTACTCTCCATATTGTTGATTGTCACAAAATAGGCAGCGCAGATTGGCTGGAGTACCTTGGTCAGAATGGATCACTGGAAGAACTTGTAGTAAAGAATTGCCAGAGAATTAGCCAGTATGACCTTCTAAAGTTTGGTCCAGGATGGATGGAGCTCCAAAAGTTTGAGTTTATGACCGATGCAGGTTTATGGGACCATCTTGAAAGATGTTATGATTCCTCATACAATGCTCACGACCCGAGTAGATATGATTTTCACTGTGATAGTTTGAAGGACTTGACATTTGCACGTTTTACAACTGGGCCAGAAATAGGGCTACGTAATCTTCTGGGCAAATGCAAAGCACTGGAGAAACTGCGCCTTGAATATGTTCATGGCCTAAATGACAATGACTTGATTGCGCTATCCAAGAGCTGCAGCAAGCTTAAAAGCATCTCACTTTGGCTCACACCAATTTTCCATGATTTCTATAAGTGCACAACATCATTTACTGATGACAGCCTTAAGGCCCTAGCCCTCAACTGTCCTATGCTTCAGACTGTTGAACTCACGTTTGGAGACTGCGAGCCCACGTACCCTTCAGAAATAGGCTTCACAAGGAAGGGCCTTGTGATGCTCATGAAGTCTTGCCCAATTCGTGTTCTTGTGCTAAATGGGGCCAACTTCTTTGATGACAAGGGAATTAAGGCCCTTTCATTTGCACCATTGCTAGAGACACTTGAGCTTATTGACTGCAAGGCGATAACGGATATCGGGATGCGTTTCATTGTGCATATCCCACGCTTGAGTAATCTGGCACTCCGGTGGTGTGAACATGTAACTGATGATGGAGTGGCTGAACTGGTACAAGCACACAAGTTAGAGTCTTTGAGCATTGAATGTTGTCTTCAGGTTTCTCTGGAGGCTGTGCAGGGGGCTGCCAGATCAGTACACTACTACACCAAGTGTGTATCCAACAGCCTTTTGGGAAAAAGAATGTTCTTAAAATACTGCTCGTGA

>BdCOI5 BRADI4G08400.1

ATGGGCCAGTGCCCCTCCGCATCCCGCCATCACCGTCACCATCGCAAGCTGCCGCCGCCGCCGTCCCCCGCCGCGCAGGCGACGTCGCCTCGTTTCTCCTCCGATGGTGCCGCGGCCGCTGCAGAGGACGCCGCCGCCGCGGCGACGGCGGACTACACCTCTGATCTCCCCGAGGAGCTCCTGGCCGTGGTGTTTGGGTTTCTCGGCTCGGGCGACCGCAAGCGCTGCTCCCTCGTCTGCCGCCGCTGGCTCGCCGCCGAGGCTGCCTCGCGCCTCCGGCTCGCCCTCGACGCGCGAGCGCCGCTCCTCGCGGCGGCCCCTGGGATCCTCGCGCGCTTCTCCGCCGTCTCCAAGCTCGCGCTCAAGTGCGACCGCCGCGCCGAGAGCGTCGGCGACCCCGCGCTCGCCCTCGTCGCACAACGCCTCGGCCCCGGCCTCCGCCGCCTCAAGCTCCGCTCCGTCCGTGCCGTCACCGATCACGGCGTCGCCACGCTCGCCGCTGCCGCCGGCAACCTCCGCAAGCTGTCTGTCGGCTCATGTGCCTTCGGTGCCAAGGGGATCGAGGCAGTCCTCCGCTCCTGCCCCCAGCTCGAGGAGCTCTCTGTCAAGCGCCTGCGAGGCCTTGCTAACTCGGAGCCCGTCGCAATCTCTGGCCCTCGTCTCCAGTCTCTGTCCCTCAAGGAGCTCTACAATGGCCAGTGCTTTTCATGTTTAATCACGCAGTCCCCTAACCTCAAAACTCTCAAGGTAATCCGATGCTCTGGCGATTGGGACCCCGTGCTGCAGGCAGTCCCGCAGGATGCTTTGCTGGCCGAGCTTCATCTCGAGAAGCTGCAGGTCAGCGACCATGGCGTCAGTGCTCTATGTGGACTCGAAGTCTTGTACCTTGCCAAGGCCCCAGAGGTCACAGATGTTGGGTTGGCAGCGCTAGCCACCAAGTCGCCACTCTTACGCAAGCTGCATGTTGATGGGTGGAAGGCGAATCGGATTGGTGACCGGGGGCTTGCAACCGTGGCACGGAAATGTGCTGCTTTGCAAGAATTGGTTCTTATTGGTGTAAATTTGACATCAGTGAGCCTTGAATTGATAGCTGCTAACTGTCCCACCCTCGAGCGGCTTGCCCTATGTGGGTCTGACACATTTGGGGATGCGGAGATCTCTTGCGTGGCGACCAAGTGTGCTTCTTTGCGGAAACTCTGCATCAAGGCATGCCCTGTGTCTGATGCTGGAATGGATAAGCTCGCTGAAGGCTGCCCACGCCTTGTCAAGGATGCTAGGAGTGTGGATGAGAGTGGTGTGCTGGAAAATGCAGGGAGCGACACTGTACCAGATGATCTTGATGATCAGATAGGGATCCCTGACCTTTCTTGTGGCAGCAGTGGCAGGCCATCAGGGTGGAAAGCACGACTGGGTGCTTTCATGTCAAGGAGTTTGTCAGTTTCCGTGTTCCGGAGGCGTCTGCAGGCGAGCTCGTGTAACTCATAA

>BdCOI6 BRADI4G11900.1

ATGAGGGGCAGGCGCGTCGACCTCATAAACGTGGCCCTCCCGGAGGAGCTCCTGGAGGAAGTGCTCCTCCGCGTGGGCGGCGCCAAGCGCGACCTGGACGCCTGCGCCCTCGTCTGCCGCCGCTGGCGCCGCCTCGAGCGCGGCACGCGCCGGTCCGCCAAGCTCCCGGCCTCCGGGGCTGGAGCCAACGAGCTGGCCCGCCTGGTCGCCGAGACGTTCTCCGCGCTCGTCGATGTCAGAGTGGACGAGCGCCTCTCCGCCGGCACCGGTCCCGGTCTCGTCGCCGTCCCGCCGCCTGGATCGCGCCGCCGCAGAGTTAGCGGGAGCACATCTGCGCGCCGCAGAAGGATGTCCCGCTCGCGATGGTTATTTCCATCGGACCAGACAGCCAATGGTGATGGGATAGAGGGCAATTTTTTCACCGATGTTGGCTTGACGAATCTCGCAGAAGGTTGCAAAGGGCTTGAAAAACTAAGTCTCAAATGGTGTACTAACATCACTTCGACTGGCCTAGTGAGAATATCAGAGAATTGTAAGAATTTGACTTCTTTGGATATCGAGGCTTGCTATATTGGAGATCCAGGACTTGTTGCTATTGGGGAAGGCTGCAAGCGACTTAATAATTTGAACTTGAATTATGTTGAAGGCGCAACAGATGAAGGCTTGATTGGACTAATAAAGAACTGTGGACCATCACTGATTTCTCTTGGTGTTACTATTTGTGCTTGGATGACTGATGCATCTTTACGTGCTGTTGGATCCCACTGTCCTAAACTTAAAATCCTGTCACTGGAAGCAGAACATGTTAAAAATGAGGGAGTGATATCTGTCGCTAAAGGATGTCCCTTGTTGAAATCTTTAAAGCTACAGTGTGTTGGTGCTGGCGATGAGGCTCTAGAAGCTATTGGTTCATATTGTTCGTTCTTAGAAAGCTTTTGCCTCAATAACTTTGAAAGATTTACGGACAGGAGCCTTTCTTCCATTGCAAAAGGTTGCAAGAATCTCACAGATCTTGTTCTCAGTGATTGCCAATTGCTAACTGATAAAAGCCTTGAATTTGTGGCACGCAGCTGCAAAAAAATAGCACGTATAAAGATCAACGGTTGTCAGAATATGGAAACTGCTGCACTGGAGCACATTGGACGATGGTGCCCGGGTCTTTTGGAGCTCTCTCTAATTTACTGCCCAAGGATCCGCGATAGTGCATTTTTGGAGCTTGGTAGAGGCTGCTCCCTTCTCCGTTCTCTTCATTTAGTTGACTGTTCAAGAATAAGTGATGATGCTATCTGCCACATAGCTCAAGGTTGCAAGAACTTAACTGAACTTTCCATTCGCCGTGGTTATGAGATAGGAGATAAAGCATTGATATCAGTTGCCAAGAATTGTAAATCACTTAAAGTGTTAACACTCCAATTTTGTGAGAGGGTATCTGATACAGGGCTGTCTGCAATTGCTGAAGGTTGCTCTCTTCAAAAGTTAAACTTGTGTGGCTGCCAATTAATCACTGATGATGGACTGACTGCCATTGCAAGAGGATGTCCTGATCTTATCTTTCTGGATATAGGTGTTCTCCAGATCATAGGAGACATGGCGCTGGCAGAGATTGGCGAAGGTTGCCCCCAGCTTAAGGAGATTGCACTCTCGCACTGCCCAGAAGTCACCGATGTCGGCCTGGGACACCTCGTCAGGGGGTGCTTGCAGCTGCAGGTGTGCCACATGGTATACTGTAAGAGAATAACCAGCACCGGAGTAGCGACTGTGGTCTCGAGCTGCCCGAGGCTGAAGAAGCTCTTCGTCGAGGAGGCTAAGGTCAGCGAGAGGACACGGCGTAGGGCAGGACCCGTCTTAGCCTTCCTCTGCACCGGGATTTAG

>BdCOI7 BRADI5G08680.1

ATGACCTACTTTCCTGAGGAGGTGGTGGAGCACATATTCAGCTTCTTGCCTGGTCAACATGACCGGAACACAGTTTCGCTTGTATGCAAGGTGTGGTATGAGATTGAAAGGCTGAGCCGTCGTACTGTCTTTGTGGGGAACTGCTACGCTGTTCGTCCTGAGCGAGTAGTTCTGCGTTTCCCTAATATGCGGGCACTGACGGTCAAAGGAAAGCCACACTTTGCCGATTTCAACCTTGTGCCACCTGATTGGGGTGGGTATGCTGCTCCGTGGATTGAGGCAGCAGCCAGGGGCTGTGTGGGTCTTGAGGAGCTGCGGATGAAGCGGATGGTGGTGACGGATGAGAGCCTTGAGCTGCTAGCTAAAACATTTCCACGCTTCAGGGCTCTCATCCTAATCAGCTGCGAGGGGTTCAGCACCGATGGGCTAGCAGCTATTGCAAGTCACTGCAAGCTCCTGAGGGAGTTGGATTTACAGGAAAATGAAGTGGAAGACCGAGGGCCAAGGTGGCTCTCCTGTTTCCCCGATTCCTGTACATCACTTGTCTCCTTGAATTTTGCCTGCATCAAGGGGGAGGTTAATGCTGGTTCATTGGAGAGGCTTGTTGCTAGGTCCCCAAATCTCCGGAGTCTGAGATTGAATCGATCTGTACCCGTAGATACACTCTCGAAGATATTAATGCGCACCCCTAATTTGGAGGACCTTGGGACTGGGAATTTGGCAGATGACTTCCAAACTGAGTCCTACATCAGGCTGGCCCTTGCATTTGATAAATGCAAAATGCTAAGGAGTTTGTCAGGATTTTGGGATGCTTCCCCTTTCTGCCTCCCTTTCATTTATCCTGTTTGTGCGCAACTAACAGGTTTAAACTTAAGCTATGCTCCCACACTTGATTCTTCCGATCTCACTAAAATGATCAGCCACTGTGTGAAACTCCAACGCCTTTGGGTACTGGATTGCATTGCGGATAAGGGCTTGCAAGTGGTGGCCTCCAGTTGCAAGGATCTACAAGAACTCAGGGTATTCCCATCAGACTTCTATATTGCTGGGTATTCCCCGGTGACTGAAGAGGGACTTGTTGCAATATCCTTGGGCTGTCAAAAATTGAGCTCATTGCTCTATTTCTGTCACCAAATGACCAACGCTGCACTAATTACTATAGCTAAGAACTGCCCAAATTTCACGCGATTCAGACTCTGTATTCTTGAGCCGGGGAAGCCTGATGCCATGACAAACCAACCATTAGATGAAGGCTTTGGTGCTATTGTTCGTGAATGCAAAGGGCTAAGGCGACTGTCAATATCAGGCCTTCTCACGGATAAGGTTTTCATGTATATCGGAACATATGCAAAAGAACTTGAGATGCTTTCAATAGCATTTGCCGGAGATAGTGATGCAGGAATGATGCATGTTATGAAAGGATGCAAAAATCTGAGGAAGTTGGAGATTAGAGATAGCCCATTTGGTGATGCTGCACTCTTGGAGAATGTTGCCAAGTATGAGACAATGCGATCCCTTTGGATGTCATCGTGCAATGTCACAGAAAAGGGGTGCCAAGTCCTTGCATCAAAGATGCCAATGCTTAATGTGGAGGTCATAAATGAGCTAGATGAAAACAATGAAATGGATGAGAACCATGGAGGGCTCCCCAAAGTGGACAAACTATATGTTTACCGCACAACTGCCGGGGGAAGGGATGATGCACCAAATTTTGTTAAAATTCTATAG

>HvCOI1 MLOC_18524.1

ATGAGCGGCGACGAGCGGCACCTGGGGAGGACCATGAGCTTCGGGATCCCGGACGTGGCGCTGGGGCTCGTCATGGGGTGCGTGGAGGACCCCTGGGACCGCGACGCCATCTCGCTCGTCTGCCGCCACTGGTGCAAGGTCGACGCGCTCAGCCGCAAGCACGTCACCATCGCCATGGCCTACTCCACCACCCCCGACCGCCTCTTCCGCCGCTTCCCCTGCCTCGAGTCGCTCAAGCTCAAGGCCAAGCCCCGCGCCGCCATGTTCAACCTCATCCCCGAGGACTGGGGCGGCTACGCCTCGCCCTGGATCCGCGAGCTCTCCGCCTCCTTCCAGTTCCTCAAGGTGCTGCATCTCCGCCGGATGATTGTCTCCAACGACGACCTCGCAGTGCTCGTGCGCGCCAAGGCCCACATGCTCGTATCCCTCAAGCTTGACCGCTGCTCCGGCTTCTCTACGCCTTCCCTTGCTCTCGTCGCCCGCCGCTGCAAGAAACTGGAAACGTTGTTTCTTGAAGAAAGCTCCGTTGCTGAGAAAGAAAATGATGAATGGCTCCGCGAGCTTGCTACCAGCAATACTGTCCTTGAGACGCTGAATTTCTTTCTGACAGATCTCAGGGCATCCCCTGCACATCTTCTCCTCCTTGTGCGAAATTGCCGAAGGCTGAAAACACTCAAGATTAGCGACTGTTTCATGTCTGACCTGGTCGACTTGTTCCGCACAGCAGAAACACTACAAGACTTTGCTGGTGGTTCCTTCGATGATCAAGATCAAGGTGGCAATTATGCTAACTACTATTTCCCTCCTTCGGTACAGCGCTTGAGTTTGCTCTACATGGGAACAAATGAGATGCAGATATTATTTCCATATGGCGCCACACTCAAGAAGTTAGACCTTCAGTTTACATTCCTTACTACAGAGGATCACTGTCAATTAGTCCAGCGCTGCCCAAATCTAGAAGTTCTGGAGGTGAGGGATGTGATAGGAGATCGAGGGTTAGAAGTTGTTGCACAGACCTGCAAGAAATTACATCGACTCAGAGTCGAGAGAGGAGACGATGATCAAGGAGGTCTCGAGGACGAACAAGGTAGAGTGACACAAGTAGGATTGATGGCTGTAGCTCAAGGCTGTCCTGATTTGGAGTACTGGGCAGTACATGTGTCTGACATTACAAATGTAGCTCTTGAGGCCATTGGTACGTTCAGCAAAAACCTGAACGATTTCCGACTTGTCCTGCTTGATAGAGAGGTGCATATAGCTGACCTGCCACTTGACAACGGGGTTCGGGCTTTGCTGAGAGGTTGCACCAAACTCCGGAGGTTTGCATTTTATGTGAGACCTGGAGCTCTATCAGATATTGGCCTTAACTATGTTGGTGAATTTAGCAAGACCGTCCGCTACATGTTGCTTGGTAATGTCGGGGGATCTGATGATGGGCTGCTGGCATTCGCACGAGGATGCCCAAGCTTGCAGAAATTGGAGCTAAGGAGTTGCTGCTTTAGTGAACGTACATTGGCAGTTGCGGCCTTACAGCTGAAGTCACTCAGATATCTATGGGTGCAGGGATACAAGGCATCTTCTACTGGCACCGATCTCATGGCAATGGTACGCCCCTTCTGGAACATTGAGTTTATTGCGCCAAATCAAAGTGAGCCTTGCCCAGAGGGTCAGGCACAGGTTCTGGCGTACTACTCTCTAGCTGGGGCAAGGACAGATTGTCCCATGTCAGTAATTCCCCTCTATCCGTCAGTCGGAAGCTAA

>HvCOI2 MLOC_52024.5

ATGCCCTACTTTCCAGACGAAGTGGTGGAGCACATCCTTGGCTTCGTGTCGTCGCATCGTGACCGCAACGCTGCATCTCTCGTGTGCCACGCGTGGTACCGCGTCGAGGGCCTCACTCGCCGCTCCGTCTTCATTTCCAACTGCTATGCGGTGCGCCCTGAGCGAGTGCACGCACGTTTCCCCTGCCTGCGCTCGCTGACCGTGAAGGGCAGGCCATGTTTTGCTGACTTCAACCTTGTCCCCGCGGGGTGGGGTGCCACAGCGGAGCCATGGGTGGATGCATGTGCCCGTACATGCCCTGGCCTTGAAGAGCTCCGCCTGAAGCGGATGGTTATCACTGATGATTGCCTCAATCACCTTGCCCGTTCATTCCCCAATTTGAGGTCACTAGTCCTTGTTAGCTGTGAGGGGTTCAGCACGGATGGTCTTGCTACTATTGCCACCAATTGCAGGTTTCTCAAGGAACTTGACTTACATGGGAGTCAGGTGGAGTTTCGAGGCCCTCATTGGTTTAGTTGTTTCCCCAAGCCTTCGACATCATTAGAATCCTTGAATTTTGCTTGCTTGGATGGAACAGTGAGTGCTAATGCATTGGAAAGTCTTGTTGCAAGGAGTCCAAATCTTAAAAGCTTAAGGTTAAATCGTGCAGTTCCAGCAGCTGTTTTAGCCAACATTCTTACTTCCGCTCCTAAGCTGGTGGATTTAGGTACTGGATTGGTTGCTCAAAACAATAATGCTGATGCACTCAGTCTCTACAATGCTATTCAACAATGCAGTTCTCTGAATAGTTTGTCTGGCTTTTGGGATTCTCCACGTTGGATTACTCCAGTAATTCATTATATTTGCAAGAACCTAACATGCTTGAACCTTAGCTATGCTCCAACGTTTCAGACAGCTGATCTTATTGGAGCTATTCGCCATTGTCAGAATCTCCGACACTTGTGGGTACTAGATCACATTGGCGATGCAGGATTGAAGGTTGTAGCCTCTTGTTGCCTGGAGCTGCAAGAGTTGAGGGTATTTCCTGCAAATGCAGATGTGTTAGCAAGCACTGATGTGACAGAGGAAGGGCTGGTTGCAGTATCTTCAGGCTGTCGGAAGCTAAGCTCTGTTCTCTATTCCTGCAGCCGAATGACTAATTCTGCTCTGATTACGGTGGCAAAGAATTGCTCGCGAATCACATCCTTCAGACTGCGTATCTGCTTGCATGGGTCAGTGGATGCCGTGACAGGCCAGCCACTGGATGAGGGTTTTGGGGCAATCGTCCGGTCATGCAAGGGCCTCAGGCGTCTATCTATGTCTGGCCTTCTCACGGACAGCGTGTTCCTGTACATCGGCATGTACGCTGAGAGGCTGGAGACGCTCTCTGTTGCGTTTGCAGGAGATAGTGATGATGGCATGATCTACGTGCTCAATGGCTGCAAGAATCTGAGGAAGCTGGAGATGAGAAACTGCCCGTTTGGCGACACGGCGCTTCTTGCAGGCATGCACAGGTACGAGGCGATGCGCTCTCTTTGGATGTCGTCGTGCGACATCACCCTGGGTGGCTGCAGGTCCCTCGCGGCAACCATGCCAAACCTAAACGTCGAGGTCGTCAGCCAGGTGGATGGAGTCTCGTGCGATGCAAAGAAGGTGGAGAAGCTGTATGTCTACAGGACACTCGCCGGGCCGAGGGGTGATGCCCCTGGATTCGTCTCGGCACTGTAA

>HvCOI3 MLOC_56088.1

ATGACCTATTTTCCCGAGGAGGTGGTGGAGCACATATTCAGCTTCTTGCCCGCACAATGTGACCGGAACACGGTTTCACTTGTTTGCAAGGTATGGTATGAGATTGAAAGGCTGAGCCGGCGAACTGTCTTTGTGGGTAACTGCTACGCCGTGCGCCCCGAGCGTGTGGTGCTTCGGTTCCCTAATGTGCGGGCACTGACAGTGAAGGGGAAACCACATTTCGCTGATTTCAACCTTGTGCCGCCTGATTGGGGTGGGTATGCCGGACCATGGATCGAGGCAGCAGCCAGGGGCTGCGTGGGTCTTGAGGAGCTGCGGATGAAGCGGATGGTGGTGTCAGATGAGAGCCTGGAGCTGCTTGCCAAGTCATTCCCACGATTCAGGGCCCTAGTTCTTATCAGCTGCGAGGGGTTCAGCACTGATGGACTAGCAGCTATTGCAAGTCACTGCAAGCTCCTGAGGGAGTTAGATTTGCAGGAAAATGAGGTGGATGATCGAGGGCCAAGGTGGCTCTCCTGCTTCCCTGATTCCTGCACGTCCCTTGTCTCCTTGAATTTCGCCTGCATCAAAGGGGAGGTCAATGCTGGTTCATTAGAGAGACTTGTTGCTAGATCCCCAAGTCTTCGGAGTTTGAGGTTGAATCGCTCTGTGTCAGTAGATACACTCTCGAAGATATTAATGCGCGCCCCTAATTTAGAGGATCTAGGGACTGGGAACTTGACAGATGACTTCCAAGCTGAGTCGTATCTCAGGCTGACCCTTGCATTGGAGAAATGCAAACTGCTGAGGAGTTTGTCGGGCTTTTGGGATGCTTCCCCTTTCTGCCTTCCATTCATCTATCATGTATGTGGGCAACTAACAGGTTTAAACTTGAGCTATGCTCCGACACTCGATTCTTCTGATCTCACCAAAATGATCAGCCACTGTGCGAAACTCCAACGTCTTTGGGTACTGGATTGCATCGGGGATAAGGGCTTGCAAGTGGTGGCCTCCAGTTGCAAGGATCTACAAGAACTCAGGGTATTCCCGTCAGACTTCTATATCGCCGGGTATTCCCCAGTAACAGAGGAGGGACTTGTTGCAATATCCTTGGGCTGTCCAAAACTGAGCTCATTGCTATATTTTTGTCACCAAATGACCAACGCCGCACTGCATACTATAGCTAAGAACTGCCCTAATTTCACGCGATTCAGACTCTGTATTCTTGAGCCTGGGAAGCCTGATGCCATGACAAACCAACCATTAGATGAAGGTTTTGGTGCCATTGTTCGTGAATGCAAAGGGCTAAGACGATTGTCAATATCAGGTCTTCTCACCGACAAGGTTTTCATGTATATTGGTAAATTTGCAAAACAACTTGAGATGCTTTCAATTGCATTTGCTGGAGATAGTGATGCGGGAATGATGCATGTTATGGAAGGATGCAATAATCTGAGGAAGCTGGAGATTAGAGATAGCCCATTTGGTGATGCTGCACTCTTGGAGAATGTTACCAAGTATGAGACAATGCGATCCCTTTGGATGTCATCGTGCAACGTCACAGAAAAGGGGTGCCAAATTCTTGCATCAAAGATGCCAATGCTTAATGTGGAGGTTATAAATGAGGTAGATGAGAGCAATGAAATGGATGAGAACCATGGAATCCCCAAAGTTGACAAGTTATATGTTTACCGCACCACTGCTGGGGCAAGGGATGATGCACCAAATTTTGTTAAAATCCTATAG

>HvCOI4 MLOC_67830.1

ATGTCTGCATCACCGTCGGAAGGCGACGAGGCCGTCATCAACGGGGTGCTGACCGACGATGAGCTCCGTGCGGTCCTCACCCGCCTGGGGCCCGAGTCGGAGCGCGACGCGTTCGGGCTCGTGTGCAGGCGCTGGCTCCGGATCCAGAGCTCCGAGCGCCGCCGCTTGCGCGCGCGCGCCGGTCCATCCATGCTGCGCCGCCTGGCAGCACGCTTCCCGGGCATTCTCGAGCTCGATCTTTCCCAATCGCCGTCCCGCTCGTTCTACCCCGGTGTTATCGACGACGATCTGAACGTCATTGCAGGGGGGTTCTGCAATCTGCGAGTCCTCGCCCTGCAGAACTGCAAAGGTATCACTGATGTTGGAATGGTCAAATTAGGAGAAGGGCTGCCATGTCTGCAAACTCTAGATGTCTCTCACTGCAAAAAACTTAGTGATAAAGGTTTAAAGGTGGTTGCGTCAGGGTGCCGGAAGTTGAGACAGTTGCACATCGCAGGTTGTAGATTAATAACTGATAATTTGTTGCGTGCTATGTCAAAAAGCTGTTTAAACTTGGAAGAGCTTGGGGCTGCAGGATTGAACAGCATAACAGATGCTGGAATCTCAGCTCTAGCCGATGGTTGTCATAAAATGAAGTCGCTAGACATAAGTAAATGCAATAAAGTTGGTGATCCTGGAATTTGCAAAATTGCCGAGGCCTCGTCGTCATCTCTGGTGTCATTGAAACTGTTGGATTGCAGCAAAGTGGGCAATAAGTCCATCCATTCACTAGCCAAGTTCTGCTGCAACCTAGAGACTCTCATCATCGGTGGATGTCAGCATATTAGCGATGAGTCCATAGAAGCACTAGCTCTTGCCTGTTGTAGCAGACTCAGGATCTTAAGAATGGACTGGTGCTTGAAAATAACGGATGCCTCATTGAGAAGTCTGCTGTGTAACTGTAAACTTCTTGCTGCCATCGACGTCGGATGCTGCGACCAAATAACTGATGCGGCATTTCAGGGCATGGAAGCGAACTTGTTTCGGTCGGAACTGAGAGTTCTGAAGATCAACAATTGTGTTGGCCTCACAGTTCTGGGGGTGAGCAGGGTGATAGAATCTTGCAAGGCACTCGAGTACCTCGACGTCCGGTCATGTCCTCAGGTTACACGGCAGAGCTGTGAGGAAGCTGGGTTGCAGCTGCCTGGTAGCTGCAAGGTGAACTTTGAAGGTAGCTTGTCGGAGTCTGATTCATCTGTTGATAGGTTCTTCTAG

>HvCOI5 MLOC_72489.1

ATGGGCCTCGGCTGCATCGCCGTCGGCTGCCCAGACCTGCGGGACCTCACGCTCAACTGGTGCCTCGGGATCACGGATTTGGGGATCCAGCTCCTCGCCCTCAAGTGCAACAAACTCAGGAACCTGCATCTTTCCTACACCATGATCTCCAAAGACTGCCTTCCAGCCATCATGAAGCTACCCAATCTTGAGGTGTTGGCACTGGTGGGATGTGTTGGAATAGATGATGATGCCCTTAGTGGTCTTGAGAATGAAAGCAGCAAATCACTACGGGTTCTCGATATGTCTACCTGTCGAAATGTCACTCATACGGGAGTTTCATCAGTTGTGAAGGCAGTGCCAAATCTCTTGGAGTTGAATCTGTCCTACTGCTGTAATGTTACTGCATCTATGGGAAAATGCTTCCAAATGCTTCCTAAATTGCAGACCTTGAAATTGGAAGGCTGCAAGTTCATGGCTGATGGACTAAAACACATTGGAATTTCTTGTGTCTCTTTAAGAGAGTTGAGCCTGAGCAAGTGCTCAGGAGTGACAGATACTGATCTGTCTTTCGTTGTGTCAAGACTAAAGAATTTGCTGAAGCTGGACATTACTTGCAATCGCAATATCACTGATGTTTCGTTAGCTGCCATCACTAGCTCATGCCATTCCCTCATCTCTCTAAGAATAGAGTCCTGTAGCCATTTTTCTAGTGAAGGGCTCCGACTTATTGGGAAGCGATGTTGCCATTTGGAAGAGTTGGATATCACCGACAGTGATTTGGACGATGAAGGTTTGAAAGCTTTGTCTGGATGCAGCAAACTGTCAAGCTTAAAAATTGGAATATGCATGAGGATAAGTGACCAAGGCCTTATCCACATTGGGAAGTCTTGTCCAGAACTCCGAGATATTGATTTGTATAGGTCTGGGGGTATTAGTGATGAGGGGGTTACTCAAATTGCCCAAGGTTGTCCAATGCTAGAGTCTATCAACCTGTCGTACTGTACAGAAATAACAGATGTTTCGTTGATGTCGCTCTCAAAATGTGCAAAGCTAAACACACTGGAGATCCGTGGTTGCCCCAGTATTTCATCTGCTGGGCTCTCAGAAATAGCAATCGGATGCAGGCTACTTGCCAAGCTTGATGTCAAGAAGTGCTTTGCGATCAATGATGTGGGGATGTTTTTTCTTTCCCAGTTCTCTCATAGCCTCCGTCAGATAAACTTGTCATACTGTTCGGTCACCGATATTGGGCTTCTGTCCCTCTCTAGCATATGCGGGCTTCAGAACATGACGATTGTACACTTGGCGGGTATTACGCCTAATGGCTTGCTGGCTGCTCTGATGGTCTCTGGTGGTTTGACAAGGGTGAAGCTTCATGCAGCGTTCAGATCTATGATGCCCCCGCATATGCTCAAAGTCGTTGAGGCTCGCGGCTGTGCTTTCCAGTGGATTGATAAACCATTCAAGGTCGAGCAAGAACGATGCGACATATGGCAACAACAGTCTCGAGACGTGCTTGTACGATGA

>HvCOI6 MLOC_73542.1

CGGGGCGCGGCCTCCCTCGTCTGCCGCTCCTGGCACCGCGCCGAGTCCGCCACCCGCGCCTCCGTCGCCGTCCGCAACATCCTCGCCGCCTCCCCGGCGCGCGCCGCCCGGCGCTTCCCCAACGCGCACCACATCCTGCTCAAGGGCCGCCCACGGTTCGCCGACTTCAACCTGCTGCCCCCCGGCTGGGCCGCCTCCGCCTTCCGCCCCTGGGCCGCCGCCCTCGCCGCCGCCGCCTTCCCCGCGCTCCGCTCCCTCTCGCTCAAGCGCATCACCGTCACCGACGACGACCTCGACCTCCTCGCCCGCTCCCTCCCGCCCTCCTTCCGCGAGCTCTCGCTCCTCCTCTGCGACGGCTTCTCCTCCCGCGGCCTCGCCTCCCTCGCCTCCCATTGCAGAGGGCTGCGTGTGCTCGACGTGGTGGACTGCGAGCTCAACGAGGAAGAAGACGACGAGGTGTCGGACTGGGTGGCGGCGTTCCCGCGCGGGCACACCGACCTGGAGTCCCTCTCCTTCGAGTGCTTCACCCCGCAGGTACCCTTCGCCGCGCTCGAGGCTCTGGTAGCGCGCTCGCCGCGCCTCCGCCGCCTGCGCGTCAACCAGCACGTCTCGCTCGGGCAGCTGCGCCGGCTCATGACGCTCACGCCCCGCCTCACGCACCTCGGCACGGGCTCGTTCCGGCCGGGGGACGGCGCCGACGACGAGGGGCTCGACTTCGGGCAGATGCTGACCGCCTTCGCGTCCGCCGGCCGGGCCAACTCGCTGGTCTCGCTGTCCGGCTTCCGTGATCTCGCGCCGGAGTACCTGCCGACCATTGCCACGGTGGCCGCGAACCTAACCACCATGGATCTCAGCTTCGCCCCTGTCAACCCCGACCAAGTCCTGCTCTTCATCGGCCAATGCCGCAGCCTCGAGACGCTATGGGTGCTCGACTCGGTGCGCGACGAGGGGCTCCAAGCCGTGGCGATGTGCTGCAAGAAGCTCCAGGTTCTCCGCGTGCTCCCATTGGACGCGCACGAGGACGCAGACGAGCTGGTGTCGGAGGTCGGGCTCACCGCCATCTCGGAGGGCTGCCGGGACCTTCGCTCCATCCTCTACTTCTGCCAGAGGATGACCAACGTCGCCGTGGTCACCATGTCCCAGAACTGCCCCGAGCTCAAGGTGTTCCGGCTGTGCATAATGGGGAGGCACCGGCCGGACCACGTGACGGGGGAGCCGATGGACGAGGGGTTCGGCGCCATCGTACGCAACTGCAGCAGGCTCACCAGGCTCTCGACGTCAGGGCACCTGACCGATCGAGCGTTCGAGTACATCGGCAAGTACGGCAGCTCCCTGCGGACGCTCTCGGTGGCGTTCGCCGGAGACAGCGATCTGGCGCTGCAGCACATCCTCCAGGGCTGCTCCAAGCTGGAGAAGCTCGAGATAAGGGATTGCCCGTTCGGCGACGCCGGCCTGCTCTCCGGCATGCACCATTTCTACAACATGCGGTTCGTCTGGATGTCCGGCTGCAGCCTGACGCTGGAAGGCTGCAAGGCGGTGGCGCGGCAGCTCCCGCGGATGGTGGTGGAGCTGATAAACAGCCAGCCGGAGAACGAGAAGACGGACGGCGTCGACATCCTATACATGTACCGGTCGCTGGAGGGGCCAAGGGAGGACGTTCCACCATTCGTGAGGATCCTGTAA

>HvCOI7 MLOC_80547.1

ATGGGCGGGGAGGTGCCGGAGCCGCGGCGGCTGAGCCGCGCGCTCAGCTTCGGCGTGCCCGACGAGGCGCTGCACCTCGTCATGGGCTACGTCGAGGCCCCGCGCGACCGGGAGGCCGCCTCGCTCGTCTGCCGCCGCTGGCACCGCATCGACGCGCTCACACGCAAGCACGTCACCGTCGCCTTCTGCTACGCCGCCGACCCGGCCCGCCTCCTCGCACGCTTCCCGCGCCTCGAGTCGCTCGCGCTCAAGGGCAGGCCGCGCGCGGCCATGTACGGCCTCATCTCCGACGACTGGGGCGCCTACGCCGCGCCATGGGTCGCCCGCCTCGCCGCGCCTCTCGAGTGCCTCAAGGCGCTCCACCTCCGCCGCATGACCGTCGCCGACGACGACGTCGCCGCGCTCATCCGCTCCCGCGGCCACATGCTGCAGGAGCTCAAGCTCGACAAGTGCTCCGGATTCTCCACTGACGCGCTCCGCCTCGTCGCACGCTCCTGCAGATCCCTGAGAACATTATTTCTTGAGGAATGTGTGATTACTGATGAAGGTGGTGAATGGCTTCATGAACTTGCTGTCAACAATTCTGTTCTTGTGACACTGAACTTCTACATGACTGAGCTCAAAGTGGCGCCGGCTGATCTGGAGCTTCTAGCAAAGAACTGCAAATCATTACTGTCTTTAAAGATCAGTGAGTGTGATCTTTCAGACCTGATTGGTTTTTTCGAAGCAGCCAGTGCATTGCAAGATTTTGCTGGAGGAGCATTCAACGAGGTAGGAGAGCTAACAAAGTATGAAAAAGTCAAGTTTCCACCAAGAGTATGCTTCTTGGGGCTTACATTCATGGGGAAAAATGAGATGCCTGTCATCTTCCCATTTTCTGCTTCACTAAAGAAGCTGGACTTGCAGTACACTTTCCTCACCACCGAGGATCATTGCCAGCTTATCTCAAAATGCCCGAACCTATTTGTTCTTGAGGTGAGGAATGTGATAGGAGACAGAGGGTTAGAGGTTGTTGGCGATACATGCAAGAAGCTACGAAGACTCCGAATTGAGCGAGGGGATGATGATCCAGGTCTACAAGAAGAGCAAGGAGGAGTTTCTCAGTTAGGCCTGACAGCGGTAGCTGTTGGTTGCCGTGACCTGGAGTACATAGCTGCCTATGTATCTGATATCACCAACGGTGCTCTTGAATCTATCGGGACTTTCTGCAAGAATCTTTATGACTTCCGGCTTGTCCTGCTCGACAGACAAAAGCAGGTAACTGATCTGCCACTCGACAACGGCGTTCGAGCTCTGTTAAGGAGCTGCACCAAGCTCCGGAGATTTGCTCTCTACCTGAGACCTGGAGGGCTCTCGGACATAGGCCTCGACTACATCGGGCAGTACAGCGGCAACATCCAATACATGCTACTGGGCAACGTCGGTGAATCTGACCAGGGATTGATCCGCTTCGCAATCGGATGCACCAACCTGCGGAAGCTTGAGCTTCGGAGCTGCTGCTTCAGCGAGCAAGCCCTTTCCCTCGCGGTGCTCCATATGCCGTCGCTCAGGTACATATGGGTGCAAGGCTATAAGGCCTCCCCAGCAGGCCTCGAGCTCCTTCTCATGGCGAGGCCATTCTGGAACATCGAGTTCACGCCTCCGAGCCCTGGCGGCCTGCATCGCATGACGCTCGACCGTGAACCCTGTGGGGAGAGGCAAGCCCAGGTTCTCGCGTACTACTCCCTTGCTGGGCAGAGGCAGGACTGCCCTGACTGGGTGACTCCGTTGCATCCAGCTGCATGA

>HvCOI8 MLOC_9864.2

ATGGTCGTCACCGACGAGTGCCTCGAGATGATCGCCTCCTCCTTCAGGAACTTCCAGGTCCTGCGACTCGTCTCCTGCGAGGGGTTCAGCACCGCAGGGCTCGCCGCCATTACCGAAGGTTGCAGAAATTTAAGAGAACTTGACCTGCAAGAGAACTACATTGAGGATTGTTCCAATCATTGGCTCAGTTACTTTCCGGAATCCTTTACTTGTCTGGAAACTCTGAACTTTTCAAGCTTAGAAGGGGAGGTCAATTTCGCTGTACTTGAGCGGCTAGTGAGCAGATGTCGCAACCTCAAGACTCTGAAGCTCAACAATGCTATCCCTCTTGACAAGGTTGCTAGCCTTCTTCGTAAGGCTCCACAACTAGTAGAACTTGGAACTGGCAAATTCTCTGCTGAGTATCATTCAGATCTCTTTGCAAAGCTCGAAGCGGTATTTGCAGGTTGTAAAAGCCTGAGAAGGCTCTCTGGGGCTTGGGATGCTGTTCCAGATTACCTGCCAGCTTTCTACGGTGTATGTGAAGGCCTCACATCACTTAATCTGAGTTATGCTACTGTACGAGGCCCCGAGCTAATAAAATTTATTGGCAGATGCAAGAATCTGCAGCAATTATGGGTGATGGACCTAATTGGCGACCATGGTTTAGCTGTCGTGGCATGCTCTTGCAGTAAACTGCAAGAGCTGCGGGTCTTCCCGTCTGAACCTTTTGGTGCCGGGCAAGTTTTATTGACTGAAAGAGGCCTTGTTGATGTTTCTGCCAGTTGCCCCATGTTGGAGTCAGTTCTCTACTTCTGCGGCCAGATGACTAATGAGGCCCTCATTACAATTGCGAAGAATCGACCCAACTTCACTTGTTTCCGGTTATGCATCCTTGAGCCCCGTACTCCAGATTACGTCACACGGCAGTCTCTTGATGCTGGCTTCAGTGCCATTGTGGAATCATGCAAGGGCCTTAGGCGCCTCTCTGTGTCTGGCCTTCTGACAGATCTCGTGTTCAAATCAATCGGTGCAAACGGTAACCGTCTAGAGATGCTGTCAATCGCCTTTGCTGGGAACAGTGATCTTGGCCTTCATTATATCCTCTCTGGCTGCAAGAGCTTGAAGAAGCTGGAGATAAGGGACTGCCCATTTGGAAATAAGCCGTTGCTGGCAAATGCTGCCAAGCTGGAGACAATGCGATCCCTTTGGATGTCGTCGTGCTCATTGACCCTGGGCGCATGCCGACAGCTTGCAGAGAAGATGCCTCGCCTTACCGTGGAGATAATGAACGATCCTGGACGAACATGCCCTGTGGAGTCACTTCCGGATGATAGCCCGGTCGAGACATTGTATGTGTACCGGACAATTGCCGGTCCAAGGTCCGACACACCAGATTACGTCCAGATTGTTTAA

>OsCOI1 LOC_Os01g63420.1

ATGCCTCCGTATGAAACAGCTAGGGCAGAGCCAGCCAGCAACAACAAGCAGCGGCCGATCTCCGGCGAGACGGCCGCTGGGAGCAGCCGATCCGGCCCCGATCCGATGGGTGGCGAGGTGCCGGAGCCGCGGCGGCTCAACCGGGCGCTCAGCTTCGACGACTGGGTCCCCGACGAGGCGCTGCACCTCGTGATGGGCCACGTCGAGGACCCGCGGGACAGGGAGGCGGCGTCGCGGGTGTGCCGCCGCTGGCACCGCATCGACGCGCTCACGCGCAAGCACGTCACCGTCGCCTTCTGCTACGCCGCGCGCCCCGCGCGCCTCCGGGAGCGGTTCCCGCGGCTCGAGTCGCTCTCGCTCAAGGGCAAGCCCCGCGCCGCCATGTACGGGCTCATCCCCGACGACTGGGGCGCCTACGCCGCGCCATGGATCGACGAGCTCGCCGCGCCGCTCGAGTGCCTCAAGGCGCTCCACCTCCGCCGCATGACCGTCACCGACGCCGACATCGCCGCCCTTGTCCGCGCCCGCGGACACATGCTGCAGGAGCTCAAGCTCGACAAGTGCATCGGCTTCTCCACTGACGCCCTCCGCCTCGTCGCCCGCTCGTGCAGATCCCTGAGAACTTTATTTCTGGAAGAGTGCCATATTACTGATAAGGGTGGTGAATGGCTTCATGAACTTGCTGTCAACAATTCTGTTCTGGTGACACTGAACTTCTACATGACTGAACTCAAAGTGGCGCCAGCTGATCTAGAGCTTCTTGCAAAGAATTGCAAGTCATTGATTTCATTGAAGATGAGTGAGTGTGATCTTTCAGATCTGATTAGTTTTTTTCAAACAGCCAATGCGCTGCAAGACTTTGCTGGAGGAGCATTCTACGAGGTAGGAGAGCTCACCAAGTATGAAAAAGTTAAGTTCCCACCCAGATTATGCTTCTTGGGGCTTACCTACATGGGAACAAATGAGATGCCTGTTATCTTCCCTTTTTCGATGAAACTCAAGAAACTGGACTTGCAATACACTTTTCTCACAACAGAAGATCATTGTCAGATTATTGCAAAATGTCCCAATCTACTAATTCTTGAGGTGAGGAACGTGATAGGAGATAGAGGGCTAGAAGTTGTTGGTGATACATGCAAGAAGCTACGAAGACTCCGAATTGAGCGGGGTGATGATGATCCAGGTCTGCAGGAAGAGCAAGGAGGAGTTTCTCAGCTAGGCTTGACAGCCGTTGCTGTTGGTTGCCGTGAATTGGAGTACATAGCTGCCTATGTATCGGATATCACCAATGGGGCCCTGGAGTCTATTGGGACTTTCTGCAAAAATCTATACGACTTTCGGCTTGTGCTACTTGACAGAGAAAGACAGGTAACAGATCTGCCACTTGACAATGGTGTCTGTGCTCTGTTAAGAAATTGCACAAAGCTTCGGAGGTTTGCTCTCTACCTTAGACCAGGAGGGCTTTCAGATGATGGCCTTAGCTACATCGGACAGTACAGTGGAAATATCCAATACATGCTACTGGGCAATGTTGGTGAATCTGACCATGGATTGATCCGTTTCGCAGTGGGCTGCACCAACCTTCAGAAGCTTGAATTGAGAAGCTGCTGCTTCAGCGAGCGAGCTTTGTCCCTCGCTGTACTGCAGATGCCCTCCCTGAGATACATATGGGTGCAAGGATACAGAGCATCTCAAACAGGCCTTGACCTCCTGCTCATGGCCAGGCCTTTCTGGAACATCGAGTTTACACCTCCGAGCCCTGAGAGTTTTAATCATATGACAGAAGATGGAGAACCCTGTGTGGATAGCCATGCTCAGGTTCTTGCCTACTATTCCCTTGCTGGAAGGAGGTCTGACTGCCCTCAGTGGGTGATCCCCTTGCATCCTGCGTGA

>OsCOI2 LOC_Os05g37690.1

ATGGGAGGGGAGGCACCGGAGGCGCGGCGGTTGGACCGCGCGATGAGCTTCGGCGGCGCGGGCAGCATCCCGGAGGAGGCGCTGCACCTGGTGCTGGGGTACGTGGACGACCCGCGGGACAGGGAGGCGGTGTCGCTCGTGTGCCGCCGCTGGCACCGCATCGACGCGCTCACGCGGAAGCACGTCACCGTGCCCTTCTGCTACGCCGCGTCGCCCGCGCACCTGCTCGCGCGGTTCCCGCGGCTGGAGTCGCTCGCGGTCAAGGGGAAGCCGCGCGCCGCCATGTACGGGCTCATCCCGGAGGACTGGGGCGCCTACGCGCGCCCCTGGGTCGCCGAGCTCGCCGCGCCGCTCGAGTGCCTCAAGGCGCTCCACCTGCGCCGCATGGTCGTCACCGACGACGACCTCGCCGCGCTCGTCCGCGCCCGCGGCCACATGCTGCAGGAGCTCAAGCTCGACAAGTGCTCCGGCTTCTCCACCGACGCTCTCCGCCTCGTCGCCCGCTCCTGCAGATCACTGAGAACATTATTTCTGGAGGAATGCTCAATTGCTGATAATGGTACTGAATGGCTCCACGACCTTGCTGTCAACAATCCTGTTCTGGAGACATTGAACTTCCACATGACCGAACTCACAGTGGTGCCAGCTGACCTGGAGCTTCTCGCAAAGAAGTGCAAGTCACTAATTTCATTGAAGATCAGTGACTGTGACTTTTCAGATTTAATTGGATTTTTCCGGATGGCTGCATCATTGCAAGAGTTTGCGGGAGGGGCATTCATTGAGCAAGGGGAGCTCACTAAGTATGGAAATGTTAAATTCCCTTCAAGACTGTGCTCCTTAGGACTTACGTACATGGGGACAAACGAGATGCCCATTATCTTCCCTTTCTCTGCATTACTCAAGAAGCTGGACTTGCAGTACACTTTTCTCACCACTGAAGATCACTGCCAACTCATTGCAAAATGTCCCAACTTACTAGTTCTTGCGGTGAGGAATGTGATTGGAGATAGAGGATTAGGGGTTGTTGCAGACACATGCAAGAAGCTACAAAGACTCAGAGTTGAGCGAGGAGATGATGATCCAGGTTTGCAAGAAGAACAAGGAGGAGTCTCTCAAGTCGGGTTGACAACTGTAGCCGTAGGATGCCGTGAACTGGAATACATAGCTGCCTATGTGTCTGATATCACAAATGGGGCCCTGGAGTCTATTGGGACTTTCTGCAAAAATCTTTGCGACTTCCGTCTTGTCCTACTCGATAGAGAAGAGAGGATAACAGATTTGCCCTTAGACAATGGTGTCCGTGCACTGCTGAGGGGCTGCACGAAACTTCGGAGGTTTGCTCTATACTTGAGACCAGGGGGACTTTCAGATACAGGCCTTGGCTATATTGGACAGTACAGTGGAATTATCCAATACATGCTTCTGGGTAATGTTGGGGAAACAGATGATGGTCTGATCCGGTTTGCATTGGGGTGTGAGAACCTGCGGAAGCTTGAGCTAAGGAGTTGTTGCTTCAGTGAGCAAGCTTTAGCCCGCGCTATACGGAGTATGCCTTCCCTGAGATACGTGTGGGTACAGGGCTACAAGGCTTCTAAGACTGGTCACGATCTCATGCTCATGGCCAGGCCCTTCTGGAACATAGAGTTTACACCTCCCAGTTCTGAGAATGCAAATCGAATGAGAGAAGATGGTGAACCTTGTGTAGATAGTCAAGCTCAGATACTTGCATACTACTCCCTTGCCGGGAAGAGGTCGGACTGCCCACGATCTGTGGTTCCTTTGTATCCTGCGTGA

>OsCOI3 LOC_Os03g15880.1

ATGGGAGGCGAGGCGGGGGAGCGGCGGCTGGGGAGGGCGATGAGCTTCGGGATCCCGGACGTGGCGCTGGGGCTGGTGATGGGGTTCGTGGAGGACCCCTGGGACCGGGACGCCATCTCGCTCGTGTGCCGCCACTGGTGCAGGGTCGACGCGCTCAGCCGCAAGCACGTCACCGTCGCCATGGCGTACTCCACCACGCCCGACCGGCTGTTCAGGCGGTTCCCCTGCCTCGAGTCGCTCAAGCTCAAGGCCAAGCCCCGGGCGGCCATGTTCAACCTCATCCCCGAGGACTGGGGCGGCTCCGCCTCGCCGTGGATCCGCCAGCTCTCGGCCTCGTTCCACTTCCTCAAGGCGCTCCACCTCCGCAGGATGATCGTGTCCGACGACGACCTCGACGTCCTCGTCCGCGCCAAGGCGCACATGCTCTCCTCGTTCAAGCTTGACCGCTGCTCTGGCTTCTCAACATCCTCCCTCGCCCTCGTCGCCCGCACCTGCAAGAAACTTGAAACGTTATTCCTGGAGGATAGCATAATTGCTGAGAAAGAAAATGACGAATGGATTCGGGAGCTTGCTACCAACAATAGTGTCCTTGAGACACTGAATTTCTTTTTGACAGATCTCAGGGCATCCCCAGCATATCTTACCCTCCTTGTGCGCAATTGTCGAAGGCTGAAAGTTTTGAAGATTAGTGAGTGCTTCATGCTTGACCTGGTTGATTTGTTCCGTACAGCAGAAATATTACAAGACTTTGCCGGTGGTTCCTTTGATGATCAGGGTCAAGTTGAAGAGAGTAGAAATTATGAAAACTACTATTTCCCTCCTTCATTGCTCCGCTTGAGTTTGCTCTACATGGGAACAAAAGAGATGCAAGTGCTATTTCCATATGGTGCTGCACTCAAGAAGTTGGATCTTCAATTCACATTCCTTTCCACAGAGGATCACTGTCAGCTGGTCCAGCGCTGCCCAAATCTGGAAATCTTAGAGGTGAGGGATGTCATAGGGGATCGTGGGTTAGAAGTTGTTGCACAAACCTGCAAGAAATTACAGAGACTTCGAGTAGAGAGAGGAGATGATGATCAAGGAGGTCTTGAGGATGAACATGGTATGGTTACACAGGTGGGGTTGATGGCTGTAGCTCAAGGCTGCCCTCATTTAGAATACTGGGCAGTGCATGTCACTGACATTACAAATGCTGCTCTAGAAGCCATCGGTACATACAGCAGCAGTCTTAACGATTTCCGGCTTGTCCTGCTTGATAGAGAAGCAAATATAACAGAATCACCCCTTGACAATGGGGTGCGTGCTTTACTGAGAGGCTGCACCAAGCTCCGGAGGTTCGCATTTTATGTAAGACCTGGGGCTCTATCAGATGTTGGCCTTGGGTACATTGGTGAATTTAGTAAGACAATCCGTTACATGTTACTTGGGAATGTTGGGGAGTCTGACCAAGGACTGCTTCAACTCTCAACAGGATGCCCAAGCTTGCAGAAATTGGAGCTGAGGGGTTGTTTCTTCAGTGAACGTGCATTGGCTGTTGCTGTTTTACAACTTAAATCACTGAGATACCTATGGGTGCAAGGATACAAGGCATCTCCCAATGGCACTGATCTAATGGCAATGGTGCGACCCTTCTGGAACATTGAGATTATTGCCCCGAATCAAGATGAAGTTTGCCCGGACGGTCAGGCACAGATTCTTGCATATTACTCACTTGCAGGAATGAGATCAGATTACCCTCACTCAGTAATTCCCCTCTATCCTTCGGTTTGA

>OsCOI4 LOC_Os02g52230.1

ATGAGCACTTCCCCCTCCTGCTCCTCCTCCTCGCCGATCCCCCAATCCCTAACCCTAGCCTCCACCTCCTCCTCCTCCTCATCCTCCGGGATGCGCGATGCGGGGGAGGGGTCGGACTCGCCGCCGTCGGAGATGTCGGAGGATGGGTCAGGAGGGAGCGGGGACGGGGACGGGGACGGGGACGGGGGAGGGGGAGGCGGGGACAGGTGGATGCCGGATCTGAGGGGAGGGAACGGCGGCGGCGGCGGAGGAGGCGGAGGGGGAGGGAGGTGGGCGCCGCCGGACCAGGTGCTGGAGAACGTGCTGGAGAGCGTGCTGGAGTTCCTGACGGCGGCGCGGGACCGGAACGCGGCGTCGCTGGTGTGCCGGTCGTGGTACCGCGCCGAGGCGCAGACGCGGCGGGAGCTGTTCATCGGCAACTGCTACGCGGTGTCGCCGCGCCGCGCCGTGGAGCGGTTCGGAGGGGTGCGCGCCGTGGTGCTCAAGGGGAAGCCGCGGTTCGCGGACTTCAGCCTCGTGCCCTACGGCTGGGGCGCCTACGTCTCCCCCTGGGTCGCCGCGCTCGGCCCCGCCTACCCGCACCTCGAGCGCATCTGCCTCAAGCGCATGACCGTCTCCAACGACGACCTCGCGCTCATCGCCAAGTCATTCCCGCTGTTCAAGGAGCTGTCGCTGGTGTGCTGCGATGGGTTCAGCACGCTAGGCCTCGCCGCCATCGCCGAGCGGTGCCGGCATCTCCGTGTGCTGGATCTGATTGAAGACTATATTGACGAGGAGGAGGATGAGCTAGTGGATTGGATCTCCAAGTTCCCGGAGTCCAACACGTCGCTGGAGTCACTTGTGTTTGATTGTGTTAGTGTCCCATTCAACTTTGAGGCCCTGGAGGCGCTTGTTGCACGCTCACCAGCTATGCGCCGGTTGCGAATGAATCACCATGTGACAGTAGAGCAATTGCGCCGTCTAATGGCAAGGGCTCCCCAGCTCACACACCTTGGTACTGGTGCATTCCGTTCTGAGCCAGGCCCTGGTGGTGCTCTGTCTGTTACTGAGCTTGCTACATCTTTTGCGGCATCTAGGTCTCTGATTTGTTTGTCAGGTTTCCGGGATGTCAATCCAGAATACCTCCCAGCAATCCACCCAGTCTGCGCTAATCTCACTTCCCTTAATTTTAGCTTTGCAAACCTAACTGCTGAGGAGCTCACACCGATTATTCGCAACTGCGTCCGTCTTCGCACTTTCTGGGTTCTAGATACAGTGGGTGATGAAGGCCTTCGGGCTGTGGCTGAGACATGCTCAGATCTTCGTGAGCTGCGAGTTTTTCCTTTCGATGCCACTGAGGATTCTGAGGGATCGGTTTCAGATGTTGGTCTTCAGGCAATCTCGGAAGGGTGCCGGAAGCTTGAATCAATTCTCTACTTTTGCCAGCGCATGACAAATGCAGCAGTAATTGCTATGTCCAAGAACTGTTCTGACCTGGTAACATTCCGTCTTTGTATTATGGGGCGACACCGCCCTGATCGGATCACTGGGGAGCCCATGGATGATGGTTTTGGGGCAATTGTGATGAACTGCAAGAAGCTCACTAGACTTTCAGTCTCTGGTCTGCTCACTGATAAGGCGTTTGCATACATTGGAAAATATGGGAAACTAATAAAGACACTGTCTGTTGCCTTCGCTGGAAATAGTGACATGTCTCTCCAATCTGTGTTTGAAGGATGCACTAGGTTGCAAAAGCTTGAGGTCAGAGATAGTCCTTTTAGTGATAAGGGATTGCTCTCTGGCCTGAGCTATTTTTACAACATGAGGTTCTTATGGATGAATTCATGCAGGCTAACCATGAGGGGTTGTAGAGATGTAGCTCAGCAAATGCCTGACTTGGTGGTTGAAGTGATGAAGGATCATCTTGATGATGAAGGGGAGATGGAGACTGTTGATAAACTGTACTTGTATCGATCACTGGCAGGAGCAAGGAATGATGCACCTTCATTTGTCAACATCTTGTAG

>OsCOI5 LOC_Os11g31620.1

ATGGTGTTCTTCCCGGAGGAGGTGGTGGAGCACATCCTTGGGTTTCTAGCGTCGCACCGCGACCGCAACGCGGTGTCGCTGGTGTGCCGGGAGTGGTACCGCGTCGAGCGCCTCAGCCGCCGCTCGGTGCTCGTGCGCAACTGCTACGCGGCGCGCCCGGAGCGCGTGCACGCGCGCTTCCCCGGCCTGCGCTCGCTGAGCGTGAAGGGGAGGCCGCGCTTTGTCCCCGCGGGGTGGGGTGCCGCGGCGCGGCCATGGGTGGCCGCGTGCGTCGCCGCGTGCCCTGGCCTCGAGGAGCTCCGGCTGAAGCGGATGGTTGTCACTGATGGGTGCCTCAAGCTGCTCGCTTGCTCATTCCCCAATTTGAAGTCGCTCGTCCTTGTTGGCTGTCAGGGGTTCAGCACTGATGGGCTTGCTACTGTCGCTACCAATTGCAGATTTATGAAGGAACTGGACTTACAAGAGAGTCTGGTGGAGGATCGAGACTCTCGTTGGCTTGGATGTTTTCCCAAGCCTTCTACATTACTAGAATCCTTGAATTTTTCTTGCTTGACCGGGGAGGTGAATAGTCCTGCATTGGAAATACTGGTTGCAAGGAGTCCAAATCTTAGAAGCTTGAGATTGAACCGTTCAGTTCCACTTGATGTTTTGGCCAGAATTCTTTGTCGCAGACCTAGGCTGGTGGATTTATGTACAGGATCTTTTGTACGAGGCAATATTGTTGGTGCATATGCTGGGCTATTCAATAGTTTTCAACATTGCAGTTTGCTGAAGAGTTTATCTGGGTTTTGGGATGCTACAAGCTTGTTTATTCCAGTGATTGCTCCTGTTTGCAAGAATCTAACGTGCTTGAACCTTAGCTCTGCTCCAATGGTCAGGAGTGCTTATCTTATTGAATTTATTTGTCAATGCAAGAAACTCCAACAATTGTGGGTGTTAGATCACATTGGTGATGAAGGATTGAAAATTGTAGCCTCATCCTGTATACAGCTCCAAGAGTTGAGAGTATTTCCTGCGAATGCAAATGCCAGAGCAAGCACTGTGACAGAGGAAGGGCTAGTTGCCATATCTGCAGGCTGTAACAAGTTACAGTCTGTGCTCTATTTTTGCCAACGGATGACAAACTCTGCACTGATTACTGTCGCAAAGAACTGCCCACGATTCACGTCCTTCAGACTGTGTGTTCTTGATCCAGGATCAGCAGACGCTGTGACAGGGCAGCCATTGGATGAGGGTTATGGGGCAATCGTACAGTCATGCAAAGGCCTTAGACGGCTATGTTTGTCTGGACTTCTCACAGACACAGTGTTCCTCTACATTGGCATGTATGCTGAGAGGCTGGAGATGCTTTCTGTAGCATTTGCAGGAGATACCGACGATGGCATGACCTACGTGCTCAATGGCTGCAAAAATCTCAAGAAGCTGGAAATCAGGGACAGTCCTTTTGGTGACAGCGCGCTTCTTGCAGGCATGCATCAGTACGAGGCAATGCGCTCACTCTGGTTGTCGTCGTGTAATGTCACCCTGGGGGGTTGCAAGTCTCTTGCAGCAAGCATGGCAAACCTCAACATCGAGGTCATGAATAGAGCAGCAAGTATCAATGAGGCAGACAATGCTAATGATGCAAAGAAGGTGAAGAAGTTGTATATTTACAGGACAGTTGCTGGACCGAGGGGTGATGCGCCTGAATTCATCTCAACGTTCTAA

>OsCOI6 LOC_Os03g08850.1

ATGTCCGAGGAGGACGACGACCAGCCGCCGCCGCTGCCGGCGCAGAAGCGGCCGCGCGCGTCGCCGCCGCCGGACCAGGTGCTCGACAACGTCCTCGAGACGGTGCTCCAGTTCCTCGACTCGGCGCGGGACCGGTGCGCGGCGTCGCTGGTGTGCCGCTCGTGGAGCCGGGCCGAGTCCGCCACCCGCGCCTCCGTCGCCGTCCGCAACCTCCTCGCCGCGTCCCCGGCGCGCGTCGCGCGACGCTTCCCGGCCGCGCGGCGCGTCCTCCTCAAGGGCCGCCCGCGCTTCGCCGACTTCAACCTCCTCCCGCCAGGCTGGGCCGGCGCCGACTTCCGCCCCTGGGCAGCCGCCGTCGCCGCCGCCGCGTTCCCCGCGCTCGCCTCCCTCTTCCTCAAGCGCATCACCGTCACCGACGACGACCTGGACCTCGTCTCCCGCTCCCTCCCCGCCTCCTTCCGCGACCTCTCGCTCCTCCTCTGCGACGGCTTCTCCTCCGCTGGCCTCGCATCCATCGCTTCCCATTGCAGGGGGCTGCGAGTGCTCGATGTGGTTGACTGCGAGATGAACGACGACGACGACGAGGTGGTGGACTGGGTGGCGGCGTTCCCGCCGGGGACGACCGACCTCGAATCGCTCTCCTTCGAGTGCTACGTCCGGCCGGTGTCCTTCGCCGCGCTCGAGGCGCTCGTGGCGCGCTCGCCGCGCCTCACCCGCCTGGGCGTCAACGAGCACGTGTCGCTGGGGCAGCTGCGCCGGCTCATGGCGAACACGCCTCGCCTGACGCACCTCGGCACCGGAGCGTTCCGGCCGGGGGACGGCCCCGAGGATGTGGGGCTCGACATCGAGCAGATGGCGTCCGCGTTCGCGTCCGCTGGCCGGACGAACACGCTGGTTTCGCTGTCTGGCTTCCGCGAGTTCGAGCCGGAGTACCTGCCCACCATTGCCGCCGTGTCCGGCAACCTAACGAACCTCGACTTCAGCTATTGCCCGGTCACTCCCGATCAATTCCTGCCCTTCATCGGGCAATGCCACAACCTTGAGAGACTATATGTGCTTGATTCGGTGCGTGACGAGGGGCTCCAGGCCACGGCGAGGACTTGCAAGAAGCTCCAGGTTCTCCATGTGCTTCCATTGAACGCACTTGAGGATGCCGATGAGCTGGTGTCGGAGGTCGGGCTTACTGCCATTGCTGAGGGCTGCCGAGGGCTCCGTTCGACGCTTTACTTCTGCCAGAGTATGACCAACGCTGCGGTGATCGCCATTTCTCAAAATTGCGTGGACCTTAAGGTATTCCGGTTATGCATAATGGGACGTCACCAGCCTGACCATGTGACTGGGGAGCCCATGGATGAAGGGTTTGGTGCCATTGTTAGGAACTGCAGCAAGCTTACTAGGCTCTCCACATCTGGACACCTGACTGATCGAGCTTTCGAGTACATTGGCAAGTATGCCAAGTCGCTCCGGACGCTCTCTGTTGCGTTCGCTGGAGACAGCAATCTGGCGTTGCAACACATCCTCCAGGGGTGCTCGAAGCTGGAGAAGCTGGAGATAAGGGATTGCCCATTTGGGGATGCTGGCCTCCTCTCCGGAATGCACCATTTCTATAACATGCGGTTCCTCTGGATGTCAGGTTGCAACCTTACGCTGCAAGGTTGCAAGGAGGTCGCACGGAGGCTACCAAGATTGGTGGTGGAGCTGATAAATAGCCAGCCTGAGAACGAAAGGACCGACAGCGTGGACATCTTATACATGTATCGGTCGCTTGAAGGGCCAAGAGAGGATGTACCACCATTCGTGAAGATCCTATAA

>OsCOI7 LOC_Os05g05800.1

ATGGGGCGCGGCGGCTCGCGCGCGGCGTGCGCCGCCGCGGCGCCGCCGTGGCACTCGCTCCCGGACGAGGTCTGGGAGCACGCCTTCTCCTTCCTCCCCGCCGCCGCGGACAGGGGCGCCGCGGCGGGGGCGTGCAGCTCGTGGCTCCGCGCCGAGCGCCGGTCGCGCCGCCGCCTCGCCGTCGCCAACTGCTACGCCGCCGCGCCGCGGGACGCCGTCGAGCGGTTCCCGTCCGTGCGCGCCGCCGAGGTCAAGGGCAAGCCCCACTTCGCCGACTTCGGCCTCGTCCCCCCCGCCTGGGGCGCCGCCGCGGCGCCGTGGATCGCCGCCGCCGCCGACGGGTGGCCGCTGCTCGAGGAGCTCAGCTTCAAGCGCATGGTCGTCACCGACGAGTGCCTCGAGATGATCGCCGCGTCCTTCAGGAACTTCCAGGTGCTCCGCCTCGTCTCCTGCGACGGCTTCAGCACCGCGGGCCTCGCCGCCATTGCTGCCGGTTGCAGACACCTAAGAGAACTTGACCTGCAAGAGAACGAGATTGAGGATTGTTCTATTCATTGGCTCAGCCTCTTCCCGGAATCGTTCACTTCTCTAGTAACTCTAAACTTTTCATGCTTAGAGGGGGAGGTCAATATCACTGTACTTGAACGGTTAGTGACCAGATGTCACAACCTGAAGACTCTTAAGCTCAACAATGCTATCCCCCTTGACAAGCTTGCTAGCCTCCTTCATAAGGCTCCTCAGCTAGTTGAACTCGGAACTGGCAAATTCTCTGCTGATTACCATTCCGATCTGTTTGCAAAGCTGGAGGCGGCGTTTGGAGGTTGTAAAAGCTTGAGAAGGCTTTCTGGGGCTTGGGATGCTGTTCCAGATTATCTGCCAGCATTCTATTGTGTATGTGAAGGCCTCACATCACTTAATCTGAGTTATGCTACTGTGCGAGGTCCTGAGCTCATCAAATTCATTAGTAGATGCAGAAATTTGCAACAATTATGGGTGATGGACCTCATTGAGGATCATGGTTTAGCTGTTGTGGCATCATCTTGCAATAAACTTCAAGAGTTGCGGGTCTTCCCTTCTGACCCTTTTGGTGCAGGATTCTTGACTGAAAGAGGTCTTGTTGATGTCTCTGCAAGTTGTCCAATGTTGGAGTCAGTGCTCTACTTCTGCAGACGGATGACAAATGAGGCACTTATTACCATTGCAAAGAACCGTCCCAACTTCACTTGCTTCCGCCTATGCATCCTTGAGCCACACACTCCAGACTACATCACACGGGAGCCTCTTGATGCAGGTTTCAGCGCCATTGTGGAGTCATGCAGGGGCCTTAGGCGTCTCTCTATCTCAGGCCTTCTCACAGATCTTGTGTTTAAATCCATTGGGGCACATGCTGATCGTCTTGAGATGCTTTCAATCGCCTTCGCTGGGAACAGCGACTTGGGCCTGCATTACATCCTCTCAGGCTGCAAGAGCCTGAAGAAACTGGAGATCAGGGACTGCCCATTTGGTGATAAGCCATTGCTGGCGAACGCAGCAAAGCTGGAGACAATGCGATCCCTTTGGATGTCGTCGTGCTTGTTGACCCTGGGCGCATGCCGACAGCTTGCACGCAAGATGCCCCGCCTTAGTGTGGAGATCATGAACGATCCTGGAAGGTCATGCCCCTTGGATTCGCTTCCGGATGAAACACCTGTTGAGAAACTGTACGTCTACCGGACGATCGCAGGTCCAAGGTGTAGAATGAAAATTCACAGCCTTTTTCAGGACATGGAAATGCATGGACTCTAG

>PpCOI1 PP1S277_20V6.1

ATGGAGTTTGAGAGGCGACGCGCTCCAGGAGCTGGATCTGGTATTCCTTTGTCAGACGAAACCTTAGCATGCGTGTTGAAGTATGTCGAGAATTGGCAGGACAGGGCCGCGGTCTCCCTCGTATGCCAGCAATGGCGGCGAGTGGATGGCGCTACTCGCAAGTTTGTCACGATTTCCTACATGTACTCCACTAATCCTGAGCTACTTACTCGGCGGTTCAAGCGCTTGGAAGGAGTGAAGATCAAGGGGAAGCCAAGGGCGGAAGAGTATGGTCTCCTCGTACCCAACTGGGGTGGCTATGCGGAACCATGGATTCGAGAGCTGGGAAGAGTTTACAGAGGTTTGCAGACCCTCCTGCTGCGGAGATGTCAGGTTTCTGACTCAGATTTGGAATTGATTGCTTCCTCGCCCTTCCATTCTGTCCTGCAAGTATTGCATCTGCACAAATGCGCGGGGTTCTCGACTTCCGGACTTCTTCCCGTTGCGAAAGCCTGCAGATCTTTAAGGACGCTGAGCATCGAGGATAGCAACGTAAACGATGAAGGTGGGGAGTGGCTGCATGTACTCGCCCGCCACAACACAGTCCTGGAGGTCCTCAACTTTGCAGTACTCGGTCTGGAAGACGTCGATGTAGCAGACTTAGCCCTGCTTCTGGAGAAGTGCAAATCTCTGGTGTCGCTCAAAGTCGGGGAGATTGAGCTTGTCGACATGGTTGGTGCTCTTGGAAAATCTTCTTCTCTCCTAGAACTCGGTGCCGGCTCTTGCAATTATCTCAACGACGAGGACAGCAGGGTATATGCTTCTATTTCGTTACCTTTGCAATTGACGTCCCTGTCGGGCCTGTGGTCAATGGGCGACTTCGGATTGTGCATGATTCTCCCCATCGCGCCAAACTTGAAGAAGCTCGACCTGAAGTTCACGTTCTTGAGCCGCAAAGCATACTGCCAGCTTTTCAGTCAGTGCCATTCGCTCGAAGAGCTTCAGATACGGAATGGAGTGGGCGACGAAGGCTTGGAAGTCCTCGGCAAGTCCTGCAAGTCCCTCCGCCGGCTCCGCATCGAGCACGACGAAGCAGGCGCTATTACACAACGGGGGGTTGTTGCAGTAGCTCAAGGCTGTAACAACTTGCAGCAGCTCGTCCTCTACGTCTCAGACATCTCCAACGCAGCCCTCGCCATGGTGGGACAAGGATGCCCTCACCTGACAGACTTCAGGCTTGTACTCACTGGAACCCAGCACGTCGTCGATCTTCCCCTCGACGACGGCTTCAAGCTACTCCTCAAAGGTTGTCCAAACATCTCCAAGCTCGCTGTTTATCTTCGTCATGGTGGCTTGACAGATAAAGGCATGAGCTACATGGGTGACTTCGGCAAAAACCTCAAATGGGTCCTGCTTGGTTGCACCGGTGAATCTGACATCGGGTTAGCGAATTTTGCCTACAAAGCTCAGAAGTTAGAGCGTTTGGAGATCAGGGATTGTCCGTTTGGTGAGGCGGGGCTTGTTGCTGCAGTCGTGGCAATGAGCTCGTTGAAATTCTTGTGGGTGCAAGGGTACAGAGCTCCTGAAGCGGGGTATCAGTTACTAGGATTGGCGCGGCCTTGGTTAAATATTGAGATTAGTTTACCGTCTGGGACTATGCCGGGGCAGTTGATCGCGCACTATGCGATCGTGGCGGCGAGGAATGATTATCCTCCTGATGTGAAGGTGCTGGTGGAGGAGACGGAGGAGTTAGAGGGCATGCTGCCGCCTTGTACGAACCGGAGGACTGTTGATCCTTGA

>PpCOI2 PP1S211_131V6.1

ATGGAGATGGGGCGAGAGAAGAGACCATCAGGATCTGGGACGGGCTTATCCGACGAGACCCTGGCGTGTGTGTTGAAGTATGTGGAGAGTGCGGAGGATAGAGCGTCAGTCTCCCTGGTGTGCAAGCAATGGCGACTCGTGGATGGTGCCACGAGGAAGTTTGTAACGATAGCTTACATGTACTCCACTAGCCCTGAGATGCTCACCCGACGCTTCAAGCGCCTGGAAGGGCTTAAGCTGAAGGGGAAGCCTCGCGCTGCGGAATATGATTTACTAGTACCCGATTGGGGTGGATATGCTGAGCCCTGGATTCGGGATCTGGGGCGCGCATATACAAGTCTGCAAACGCTGCAACTGCGTCGGTGCCAGGTTTCTAATGCGGATTTGACCTTAATTGCGTCTTCTCCCTGTCAAGCGTCTCTGCAAGTTTTGTATTTACATAAATGCGCTGGGTTTTCCACCGCTGGCCTCCTCCCTGTTGCTAAGTCCTGCCGGTCTCTGAAGTCTTTGAGCGTAGAGGACAGCGATGTAACTGATGAAGGTGGAGAGTGGCTATTCGAGCTGGCCCGCAACAATTCCGTGTTGGAGGTCCTGAATTTTGCTGTACTTGGTCTTGAGGATGTTGATGCAGCTGACTTGGTGTTGCTAGTGGAGAGGTGCAAATCACTGGTTTCTCTAAAAGTTGGTGAAGTTGAAATGGTGGACATGATAAGTGCCATTAGCAGAGCGTCTTCTTTGACTGAATTCGGCACAGGCTCTTGCAATTTCTTCGGGGACGAGGACAGCAGGACACATGTATCTATATCTTTACCTTCAAGCTTGACGGGTTTGTCAGGTTTGTGGGCCATGTCCGACCCTGGATTGGCTATGGTTCTTCCCATAGCACCAAACTTGAGAAAACTGGACCTGAAGTTCACGCTTTTGAGCAGAAAAGCTTACTGCCAACTTTTCAGTCAGTGCCATGCTTTGGAAGAGCTTCAGGTTCGCAACGCAGTTGGGGACGAGGGCATGGAAGTTATCGGCAAGACATGCAAGAGCCTCAGGCGATTACGCGTGGAGCACGATAATGCAGGAGCTATCACTCAACGAGGCGTTGTTGCTGTTGCCCAAGGGTGTGCACGAATGCAGCAGTTGATCGTGTACGTGTCCGACATCACCAACGCCGCGCTGGCGATGCTGGGACAATGCTGCGCACAGCTGACGGACTTCCGTCTCGTGCTGGAGACCGCTGCAAGACGCGTCGTCGACCTGCCGTTGGACGATGGAATCAAGCTCCTGCTCAAAGGCTGCCGAAAAATATCCAAGCTTGCTGTATATCTTCGGCACGGGGGCTTGACAGACAGAGGAATGGGTTACATCGGGGAGTTTGGCACGAATTTGAAATGGTTATTGTTGGGATGCACAGGCGAATCCGACATTGGATTGGCCAGTTTGGCATACAAAGCGCAGCGCATTGAAAGGTTAGAGTGTCGGGATTGTCCGTTTGGGGAGGCAGGTCTTGCGGCAGCAGTAGTGGCGATGAGCTCGCTCAAGTTTATATGGATTCAAGGCTATAGGGCTCCATGGGCAGGAGAGCATCTACTGGCCTTATCACGACCGTATCTGAACATAGAAGTTATCTCCTCAACAGACACCCAACCAGGCCAGCTCATAGCCCACTATACCACTGTCGGGCCTCGCACTGATAACCCTTTGGAGGTAAAGCAGCTGACGTTAAACCCGGACGATCACCTGCAGGAAATGCGACCGAGTTTACACTCACCTGGATCTACGCGGCACTAA

>PpCOI3 PP1S24_60V6.1

ATGGTTATGGGGGTTGTTGACGCTGAGAAGCTGGAGTCCAGGAAATCGCTCCCCTGTGGGATGTCGGACGAGACCCTGGCGTGCGTGTTAAATCACATTGAGAGTCCCCAAGATAGGGCAGCTGTGTCCATGGTGTGTCAGCAATGGCGGCGAGTGGATGGCATGACTCGGAAGTTCGTGACGATAGCGAATATGTATGCTACCTCTCCTGCTTCATTGACGAGGAGGTTTAAGGGCTTGGAGGGGATCAAGTTGAAGGGGAAGCCGAGAGCGGCTGAGTATAACCTGGTGCGGAGTGACTGGGGAGGCTACGGGGAGCCGTGGCTAAAGGTGCTTGGGCGACAGTATGCTGATCTTCACATTCTGCAGCTGCGGCGGCTGACGGTGCTAGACTCTGACTTGGAGCTCATAGCGTCGTCGACCTTCAGCTCCGCGTTGCATGTTTTGCATCTGCACAAGTGCGTTGGCTTTACTACGAAGGGTCTTCTTCCCGTAGTACGCGCGTGTCGATCTCTGAGGAGGTTGAGTCTTGAAGACAGCGAGGTAGAAGACAAAGGAGGCGAATGGCTACACGCCTTGGCCCTCAACGATTCCACCTTGGAAGAACTCCATTTTGGAGTACTTGGAATAGAAGCTATCGACATCGAAGACTTGACTATTCTGGTGGAGAAGTCCAAGTCGTTAGTTTGCTTGAAGGTGGCTGAAATCGAGTTACTGGACATGATTGATGTATTGCAAAGAGTTCCTTCCCTTGAAGATCTTGGTGCAGGATCCTGCAATTACCTTGGTGCCAAGGATGTTGATGATTTTGTCTCCATTCCATGGCCGAAGAAATTGAATGCTTTATCAGGGATGTGGTCTCTCATGGATTCAGGCTTGCCACAGATTCTTCCCATTGCGCCAAATCTGATCAAGCTGGACCTAAAATACACGCTTTTGAGCTGTGAAGGCCACTGCCTGCTTCTCAGCCATTGCTTTTCTCTCCAAGAACTCCAGACTCGAAACACACTGGGTGATGATGGCATGGAAACACTCAGCAGGTCGTGCAAAGGATTAAAGAAGCTGCGTGTGGAAGACGATGAAACTGGAGCAATCACACAGCGCGGCATTGTTGCTGTAGCGCAAGGCTGTGAACAACTGGTGCAGCTGATATTGTACGTTGCCAACATCAGCAACGCAGCGCTAGCGATGGTGGGGCAAGGCTGTCCACACTTGGTAGACGTCCGCATTGTGCTGGAGCCCAGTGCGAGGTATGCTCCAGATTTCCCTTTGGACGATGGCCTCAAGCTCATGCTCAAGGGTTGTGTCAACCTAAGGCGACTGGCTGTGTATCTCCGTTACGGTGGGTTGACTGACAAAGGAATGGAATACATCGGTGTCTATGGGAAAAACCTTCAGTGGTTGCTTGTAGGATGCGCGGGAAACAGCGATGTTGGGCTTGCTAACTTCGCCCACTGGGCACAGCGCATACAACGGCTGGAGATTCGAGACTGTCCGTTCGGAGAGACGGGAATGGCAGAGGCTGTTTCGGCGATGAGTTCATTGAAGTATTTATGGGTACAAGGAAGTCGAGCTTTGGAAGCTGGTGAAAAACTATCTGCGCTGTCTCTCCCTTGTCTAAATGTGGAGGTTTGTCCACCACCCGCAGGGCAGCCAGGAGGCCAACTGTTTGCCTACTATTCACTAGCAGGGCCGAGGAAAGATGGGCCTACAGGACTGAAGACTTTTATTTCAAACACAGTGAAAGATCAGTAA

>PpCOI4 PP1S140_66V6.1

ATGACGATGAACCCCGCCATTGTAACAAAATTCCTTGAAAAGCACTTTAGTATACAACTCACAAGACAAAGTTCAAAAAAAAAATGTCTCTCAGTACTTGACGAGACCCTAGACCTCATCTTTAGCTATTTAGATCCCGAGGACCGGGCTTCCGCCTCCTTAGTATGCAAACACTGGCACCGGGTCGATGGCGAAACACGAGAACAAGTCTCCGTGTCCAATTGCTATTCCGTTTCACCATCAGCTCTCAGCAAAAGGTTTCCCAACATCGAGAAATTTAAAATTAAGGGGAAACCTCGGGCCGTGGAGTTCAATCTTCTCGTAGATGACTGGGGTGGTTATGCAAGCGCATGGGTCGAAGAGATAGTGAGAGCTTACCCGAGACTTCACACACTCCATTTCCGGCGCATGGATGTATCTGACGACGACTTGAAGATACTGGCACAGGGTTGTGGGTCTGCTCTGCAGGTACTGAAATTAGACAAGTGCTCAGGCTTCTCGACCTTAGGCCTTCAGCATATTGCTCGCTCTTGCAGGTCTTTGAAAACGCTGTATTTGGAGGAAAGTGATATTGAAGACGAAGGTCATGAATGGCTACTTGATCTTGGACGAAATGTTCCTGGATTAGAAAGGCTTAATCTGGCTTCAACAGGTATAGAAGAAGGTGATGTCAATGATGTCCTAGTGGTTTTAATGCAGAATTGCAAATCACTAAATTCTCTTAAAGTTGGTGAAATGACGCTTGAGAACTTCAAAGAGATCATGAAGTACTCAACTACACCACTTTTGGAGCTTGGTAATGGGTGCTACAGCATGCGTAATGGCGTACGTGAGGAATTAACGTTTGACGCCGCCTTTATTCCCTGGGTCTCAAGATTGAAGGTTCTTGACTTAAAATTCATGAACCTGAACGCCGCGGGCCACTGCCAGCTACTTGCATGTTGCCCTCTACTTGAAGAACTGGAGGCAAGAATTGAGATCCTTGATGAGGGTCTGGAAGTCGTAGGGAAAACCTGTAAATATCTTAAACGGATTCGAATTGATGACCAGGATAGTCCGGGATTTATAACTCACAGGGGCCTCACTGCAATTGCGAAGGGCTGCAGAGAACTGGAATTTTTAGTAATGTACATGAGAGATGTCACTAACAGCTCCTTAGAGGCTGTTGGGCGCTATTCTGAGAACCTGAACGACTTCCGGATTGTTTTACTGAAGACACTAGCGCATCCGGAAGACCTCCCACTGGACAAAGGAGTGTGTTCTTTACTGCAAGGCTGCCCAAAGCTGACCCGTTTTTCTGTGTACTTGCGTCCGGGAGGCCTTTCTGACATAGGATTGTCATATATTGGGAAGTACGGGGGAAGGCTGAAGTGGATTCTACTGGGCTGTTCTGGTGAAAGCGATCAAGGGCTATTGGACCTTGCATATGGTTGTCAGAACCTTAGAAGACTTGAACTTCGTGGATGTCCCTTTTCCGACGCAGCCTTGGCACAAGGCATGATGAACATGGCGAAAATGAAGTATCTGTGGGTCCAAGGCATTGGCGCAACTGAAATGCTCGGCAGATACTTAGTAGGGTCACATCCTTGCCTCCACGTGGAGTGGATGCCTTCTGAACAGCAGTTACTAGCATACTACTCGCTAGCTAGTCATCGAACGGATACTCCTCCAACTGTAGAAATTTTGTCTCAGAGTCGATTTGACTACGATTCCGAAATTTTCGGTGATTATGATGAAGAATGTGTTGGCAACTTGGAGAGTGGAGTTTTTGACGGAGAATATGGCGAGGGCCCCGATGTTGGTGATTTTGATGGAGAGTATGGTCATGGGACTAATTGGGATTTCTAA

>PpCOI5 PP1S187_72V6.1

ATGTCTGAGGTGGCATCCTGGGGAGATTCTCCGCAGTTAAGCAACAAGAAGGGGAAGAATAGACTGGCGAATGAATCCACGCCTAGTACGGAATGTAAATTCAATCTGCTGCCGGAGCCAATTATTGAGTCGATATTTAATCGGGTAGAGGCTAGGGGCGACCGCAACGCAATGTCCCAGGTGTGCAAGCTGTGGCAGAAAATGGACGGCATGACGAGGAAGAATATCTACATCTCCAACTGTTACTCCATTGCACCATCCGACGTTTCGAGACGGTTCAAAAGCTTGCAGAAAATCAAAATTAAGGGAAAGCCAAGAGCGTATGAATTCGGCCTTCTGGTCGAGCGCTGGGGCGGTCACGCAGGTCCATGGATTGGGGAGATGTCTCGTGCCTACCCCGAACTCCTGGGGCTTTCCATGCGTCGCATGGACGTCACGGACAATGATTTGAGAATTCTCGCCTCGAGATGCCCCAAACTGCAGAAATTAAAGCTGCACAAGTGTTGTGGGTTCAGCACCGGGGGTCTTGAGCACATCACACGGTCCTGCAGAACCTTGCGAGTGCTTGACATAGAAGAAAGTGATGATATAGAAGACACCGGTGGGCCGTGGTTGGAGTTGCTAGAAAATAGTGACGGCAGATTGGAGAGTTTGAACATAGCCTCTGCAGGATTGGAGGAGGAGAACATAAAGGAGGTGCTGCCAGTGGTAGGGCGCTCGCTGAAGTGCATCTCTTCCCTCAAGGTCAGCGACATGGAGCTGGGCAGTTTTTTTAAAATCCTGGATAACAGCAATGTGCCTGTGGTTGAGCTTGGTCTTGGCTGCTATTGTTCAAGCCCGGAAGACCCAAAGGAACTGGCGTCCTCGTTCGCTCTGCGCTTATCAAAGCTGCGAAGTGCGGTAGGGGACAGGGGAATGCAGGTGATTGGCGAGACTTGTAAGCAATTGAAGAGGATCAGAGTTGATCAAGACACTTCAGAGTACATGACAGACTATATTACACAGAAGGGGATGATTGCCATCTGCGAAGGGTGTCGAGAGCTCGACTTCTTAGTCATGTACCTTTCTGACGTTAACAATGAAGCACTTGCAGCAGTTGGACGATGCCTTCCGAAACTCACCGACTTCCGCATTGTGTTGCTTGAGGTTCGGAACGACGTGAAGGACTTGCCGCTTGACGAGGGCGTCCGCTTGCTCCTCCAAGGCTGCCCAATCCTGACACGGTTCTCAGTGTACTTGCGGCAAGGCGGTTTGTCGGACAAAGGCGTAGGATACATAGGGCAATTCGGCACTAAGCTAAAGTGGGTCCTCTTGGGCTGCTCTGGCGAAACTGACAAAGGGCTGCGTCTTATGGCTGAAGGTTGTCGGCAGCTTGAAAGGCTGGAGCTTCGTTGCTGTCCCTTCACTGAGCTGCAACTTGCATCTTCAATCTTAAACAGCTGGCGACATTTGAAATACCTGTGGGTCCAGGGCGTCGGCGCTACGTCGGGTCTAGGTGTGGATCTAGTGACCCATAAGAGTGGGTTTCTTGTTGAGTTCATGGGGGAGACCCAGCAGATTCTGGGGTATTACTCTGCCACCCGCCCTCGCACAGACAACCCTAGGTCTGTGTGCTTGATTAACTATGTACCTCCGGAAGATAGGCCCGAAGCGGATGCAAAGGGATTTCAGGGAAATACTCATGCATCCCAGGGCGATGCTCACAGTTTCTACTCCGACTATGCAGATGGATACGTACGTAATTCGTGCTCTGAAGCAGAGGCATTTTATCCTGGATTTAATGAGAACAACCTCTATCCTGACGCTGATGAGAATGCATTCTACACTGGAGTTGTTGACGCTGGGCTCTACCTTGGAGTCGGCGACAGTTGTGGCTATCCTGAATTTGGTGAGGACGAAGCTTTCATATACTAG

>PpCOI6 PP1S49_196V6.1

ATGGCTGCGGAGGTTGTCTGGAGAGAAGATGGAAACATAAGAAGCAGATTTTTTCGGGGGAATGAACATGGGCCAACTAAAAAATGCATTTTCAATCAGCTGCCGGAGTCAGTTATTGAATTGATATTCGATCGGCTAGGGTCCAAAGGTGACCGCAGAGCAATCTCTCAGGTGTGCAAGCAGTGGCATAGGGTGGACGGTCTGACGAGGAAGAACATTTACATTTTCAACTGTTACTCCATTGCACCCTCCAACCTTTCGAAACGGTTTCCAAACTTGGAAAAGATTAAAATAAAGGGTAAACCACGAGCTTACGAATTTGGTCTACTGGTCGAGAGTTGGGGCGCTCATGCAGGTCCGTGGATTGAAGAGATTGCGAGCGTATACCCTAATCTAGAGGGGCTTGCTTTGCGTCGCATGGACATCACCGACAAGGATTTGATGCTTCTGGCCTCCCGATGCCCCAATTTGCGGAAGTTGAAGCTGCACAAGTGCTCGGGGTTCAGTACTAGGGGGCTTGAGTTCATCACACGGTCCTGCAGAACTTTGCGGGTACTGGATATAGACGAAAGTCACGATATGGAAGACACTGGTGGGCCTTGGCTGCAGTTGCTGGAAAAGGGTGACGGCAAATTAGAAAGCTTGAACATAGCGTCTGCAGGGTTGGAGGAGGAGAGCATAAAGGAGGTGCTGCTGAAGCTCGCACCGTCCCTTAAGTGCATCTCCTCCCTCAGGGTCAGCGATATGGAGCTGGGCAGCTTTTTTAAGATCCTGGATAACAGCGAGGTGCCGGTGGTAGAGCTAGGTCTTGGCTGCTACAGTTTAAGCCAGGAGGATCCAAAGGAATTGGTACCCTCGTTCTCGTCGCGGTTATCAAAGCTCAAAATTCTTGATCTCAAGTTTACCACTTTGAATGCTGAAATTCAGATCGAACTCTTGAGGCATTGCTGTAGTGTCGAAGAGCTTGAGCTGCGAAGTGTTGTAGGGGACTGGGGAATGCAGGTGATTAGCGAGAATTGCAAGCAACTGAAGAAAATTCGAGTCGATCAAGACACTTCACCGTACATGACAAACCATGTGACACAAAAGGGAATGATTAGCATCTGCGAAGGGTGTCGAGAGCTCGACTTCCTTGTCATGTATCTTACGGACGTCAACAACGCAGCACTTGCTGCTGTCGGTCAATACCTGCCGAAACTCAGCGACTTCCGCATTGTGTTGCTTGAGGATCAGGATGACGTTGAGGACTTGCCACTCGATGACGGCATCCGCTTGCTCCTTCAAGGCTGCCCAATGCTGAGTCGGTTCTCGGTGTACTTACGGCCAGGCGGCCTGTCGAACAAAGGGTTGGGATACATCGGAGAATTTGGCTCGAAGCTCAAGTGGGTTCTCTTGGGCTCTTCAGGTGAATCTGACGAAGGGTTCCGCCTCATGGCTGAAGGTTGTCGGCAGCTGGAAAGGTTGGAACTTCGTAATTGTCCCTTTAGCGACAAGCAGCTTGCAATCTCAATCTTAAACAACTTGCCACATTTGAAATACCTATGGGTTCAAGGCTTTGGAGCTACGTCGGGTCTTGGTGTAGCTCTTGTGACCCAGATGCCCGGTTTTGTCGTTGAGTGCTGA

>PpCOI7 PP1S12_347V6.1

ATGTGGTGCAACTGTCTGGTGTCTGTGAAGATCAAATTCTCTGGCACAATGGGTTGGCTGCAACTGCGTGACGGCGTTCAGCGAGGAAACAGCGGTTGGTCTTCGGGCTTAGAAATCGGCTACAGTGGTGTGGGAAATAGTGTCTGCGATGAGAACGTGGAGTTTTTACATCCGGGTGTGATGCTTGAGGCCTCATTCCCATTCGCGGATGGTAGTGTCGCGGCTTGGGGGCGTAGAATTTGCAAGATGGGTAGTTGGAGGGGAGCGAAAGCGACGAGGGTCGACGAGGATCACTTCCCATTGCTTCAATTTCCGGATGAAATTATTGAGAAGGTGATTGGCTTCCTCACAAATCCGGTAGATAGGAACTCCACATCCTTGGTGTGTACGCGCTTGAAGGCTATTGAGGGCGAGTCGCGGGAGACCGTGCTAATCTCCAACTGTTACGCAATCCAGCCGGGTACTCTAAAGTCGCGGTTCCCGAATGCGAAATCCATCACTATCAAAGGCAAGCCTCGCATCGTGGATTTCTCGCTGATTCCTCACGCCGAGGTATGGGGTGCATACGCCACCCCCTGGGTCGACTTGCTGAAGGAACACTACCGGCCTATCAGGCACCTGAAGATGAAGCGCATGACGATTTCAGATTCCGACATCAAACGCTTTGTGAGTGCTTGCGGGTATAGTCTGGAGAGGCTGGAGTTCGAGAAATGTTCTGGGTTTAGTACCACGGGGTTGCAGTACATAGCTGGGGCATGTCGCAACCTGGTTGTCCTGAACCTCTCTGAAGCCGACATTCTTCAGGGTGATGCACCCTACTGGATGACAAGCCTCGTCAACACAGCTAGTTCGCTCCGAGTCCTTGATCTCTACCTTACGGAAGTGGAGGATGTCGAACAGAGTGTGCTTGAGAGGCTAGCTAAGCAGTGTCACACTTTAAGGTTATGCGATGCTCTGAAGATAAATCATGTGTTACCGGTTGTGACGGCCGCTTGCGAAACAGTCCGCCACTTAGGCATAGGGCTGAGTTTTCAGAACGGTGACAGCCCCAATCAGATAGCAGAGGCGCTTGGGAGATGCAAGGAGCTGGAAGGAATCTCAGCGGTGTGGGATCCTGACGAGGTATCGGCGATGATGTTGATGCCCGTTGCTGCTCGGCTTAAGACTTTGGACCTCACCTACGCCCTACTCGAACAGCCTGAGCTCACGGACCTGCTTGGCGCTTGTGTGAATTTGGAGGATCTTCAGTGCACTGATGTCATACGAGATAGGGGACTGCTGGAAGTCGGCACCTGCTGCAAAAAGCTGAGAAGTCTAGTCGTTCAACAAGACGCAGCGGGTTTTGTAACACAGAACGGTTTGACTGCCGTGGCGAAAGGGTGCTTCTTGCTGGAGAAGATCATCATATACGCCGCTGATATGACCAATGAAGCTTTGGAGACTCTTGCAACCAATTGCCCTAATTTATCTGATATCCGCATATGCCTTGTCCAGAAGTACGATGGTTCCCATCCAGTCGTGGAGTTGGAAGGAAACTCCACATTGAATTTGGGAGTCAAGGCACTTCTGATGAAATGTCCAAAAGCTCGGCGGCTGGCGCTCTGTTTCAGCAGATTTGGCTTGACAAATGTAGTGATCACCGACGAGGGCATGAAGCACATTGGCGAATATGGAGGCAACCTTCACATCATCACGCTTACAAATTGTGGCGGCAGCAATGCAGGCCTTGAATACATTGCCAAGGGTTGTAACGAGCTTCGGAAACTCGAGCTTCGTCATTGTCCATTTGGAGATGCAAGTATGGAGGCCCTTGCTCGTGGGTGCAAAAGCTTGAAGCAACTGTGGGTTCAGGCCTGCCAAGTTGAGCTCAGGGGTGTGAGGTTGTTGGCTCAACGACCAGGCTTGACTGTGGAGATTGTCGAGGAGAGCAACAATGACGGCGATATCACTCCATGGCAGCTCATTGCCTACGCTTCCGTTGCACCCCCGCGGAAAGACCTTCCGGACAACATCGACTATGTCCACGAAGGATACTGCAAGCCTCTCTACTGCAAGAATTATCTGTGCCCCAGCACCGTTTAG

>PpCOI8 PP1S3_119V6.1

ATGGCTAATCTGGGGAGAAGAAGGAGGTCGACAGTGGAGAGTGAGCACTGTAGTTTACTTCAATGTCCAGACGAGATTCTCGAGAAGATTGTTGACTTAATCTCGGATACGGCAGACAGGAGTGCCATATCCCTCGTCTGCAAGAGCTTGAAAGTCCTCGAGGGGCACACACGCGGAGTGGTACTTGTCTCCAACTGCTATGCAATTCAGCCGCTGACTTTAAAGGACCGGTTTCCCAACGCATGGTCCATCACTATCAAAGGGAAACCCCGTATTGTGGATTTTGCTCTCATTCCCCATGCGGAGGTATGGGGAGCTTACGCCACGCCATGGATGGAGATCCTAGTGAACTTTGACAGGCCCATCCGGCACCTGAGGATGAAGCGCATGACAGTTTCAGATAGCGACATCCAGCTCTTGGTGAGTAGGTGCGGGGAGGGTTTGCAGAGGTTGGAGCTGGAGAAGTGCTCTGGATTTTCGACTTTCGGTTTGGAGATCATCGCCCGGGCCTGCCGGAACCTTATTGAACTGAACATCTCGGAATCGGAGATTCAGAATGGCGGGCACCGCAGTTGGTTAACAACCCTAGTTAATACAGCAAAGTCGCTGCAAGTGTTGGACCTTTCTCTCACGGATGTGGAGCATGTGGAGCAGTCTGTGCTTGAGAAGTTGGCTGGCCAGTGTCATACCCTAAAGTTAAGTGCGGCTTTGGAGATTGAACGCGTCTTGCCTGTTGTGGAAGCTGCAAACCACAGTATGCGCCACTTGGGCACCAGGTTTTATTCACAGAACATCGAAAACCCTCATCAGATTGCGGAGGCATTTGGGAGATGCAGAGTACTAGAAGGTATTTCGGCTCCACTGGACCTGGACGAAGGGTCCATGATGATGGTGATGCCTATTGCTGGTCGCTTGACTACGTTGGACCTCACCTATGCTAACCTCGGACAACCTGAGCTCAGTGACCTACTTCGCACATGCATAAATTTGGAGGACTTCCAGTGCACGGATGTGATTGGTGACAGAGGACTGCGGGTAATCGGAACGCACTGCCAAAAGTTGAGAAGGTTGGTCGTTCAACAAGACGCACAGGGCTTTGTTACACAACATGGGTTGACGGCGGTGGCAAACGGTTGTTTTTTACTGGAGAAGATCATTATTTATGCGGCAGACATGACCAATGCAGCATTAGAGACTCTTGCAAACAATTGCCCTGGTCTGAGTGATATTCGCATATGCCTTGTTCAGAAATACCATCCCAGCCATCCTGTTATAGAGTTAGAAGGGAACTCTACATTAAACTTGGGAGTTAGAGCACTTCTGATGAGATGTCGAAGGGCACGAAGACTGGCACTCTGCTTCAGCAGATTTGGCCTCTCAAATGTGGTGATCACCGACGAAGGCATACGCTACATTGGAGAGTATGGTGGCAACCTTCACATCATCACACTCACCAATTGCGGCAGCAGTGATGCAGGTCTTGAATCCATAGCCAAAGGGTGTACGAATCTGCGCAGATTCGAGCTTCGTCATTGTCCGTTCGGAGATAGAAGCATGGAATTTCTTGCTACGTCTTGCCATAGCCTGAAGCAGCTATGGGTGCAAGCCTGTCAAGTGGAGCTAAACGGGGTGAGAGTTCTAGCTAGGCGGAAAGACCTGGTGGTGGAGGTTGTAAAAGAGAGCACCAACGAGAATGGCGACCCAATTCCATGGCAGTTCATCGCATATGCTTCTGTAGCATCCCCTCGGAATGATCGTCCCGAAAACATCGACTACGTCCACAGCCAGTATGACACACCTCTTCGCAGTGAGTACTTCATGTGCCCCAGCACTGGCGCTGAGTTGATAGACGACCTCGAGGATGGGCTGCAAAATGTTTGA

>PpCOI9 PP1S44_198V6.1

ATGCCCCCCTCGGTCTTTCCAGACGAGGTTCTCGAGCATGTTTTAGTCTTCCTAGATTCACACAAGGACAGAAACTCTGTCTCGCTAGTGTGCAAATCCTGGTACAAGGCTGAGGGATGGAGCAGGCGGAAAGTCTTCATAGGGAACTGCTATGCAGTGTCTTCGGCCACTCTCATCAGGAGATTTCCCAAGTTGGTTTCCCTTGAGATGAAAGGGCGGCCGAGGTTTACCGACTTCGGCCTGGTTCCACAAAATTGGGGTGGATTCATTCAGCCATGGATCCAGGTCATGGCCGAGTACTATCCTGGGTTGGAAGGGCTTAAATTGAAGCGTATGACCGTTTCTGACGAGAGTCTGAGAATGATCGCCGTCGCTTTCCCCAATTTTCGATCTTTACGTCTCACCAGCTGCGATGGATTCAGTACTGACGGGATCACAGAGATCACAAAAAACTGCAGAAATCTCGCGGTCTTGGATCTCCAAGAGAACTATATGGACATCCGAAATGGAGACTGGTTGAAGGCTTTTCCTGAGTCTCTGACATCTCTTGAGAGCCTCAACTTTGCAACTGTGAAATGCGCGGTTGATGAAGAAGCTTTTCAATGTCTGGAAGCTTTGGTGGCTCGCTGCCGATGTTTGAAGACATTGAAGGTGAACAAAGATATATCTCTGGGGCAATTGAGAAGTCTTCTTCTTCGAGCTCCTCAACTTGAAGAGCTGGGCACAGGAATATATAACCAAAATCTGTCATGGGGTAAACTCCACGAGCTTCAGGGTTCTCTCAAGAGGTGTAAGAACCTTCGGAGCCTCTCAGGGCTTTGGGAAGTAATTCCAATGTGCCTACCTACGATGTACCCAGTCTGTCTGAATCTGACCAGCCTAGATCTTTCCAATGTTACTCTCATGACCACGGATTTCACAAAGTTTATTAGTTATTGCACCAAAGTGCGGCGCCTGTTGGTTCAGGACTTCGTGGGTGATAAAGGACTAGCAGCTGCAGCTTTTAATTGCAAAGAGTTGCAGGAGCTTCGAGTCTACCCTGTCGGCGTTGACGGTTACGTCACTGAGCAGGGCTTCATAGCGATCTCCAAGGGGTGTCCCGAATTGAGAAAGATTCTGTATTTCTGCAAGCAGATGACCAACGCTGCAATGGTGAGCTTTGCCCAGAACTGCCCAAAGATGACCCACTTCCGGTTGTGTATCATGAAGTGCTACATGGAGGATTGCGAGACCGGGCAGCCCCTGGATGAAGGATTTGGAGCAGTTTGCAGGTTGTGTGTAGATCTTCGGCGTTTGTCCCTCTCTGGGAAGATGACCGACAAAACCTTCGAGTATATTGGGCAGTATGCAAAGAAGCTGGAAATGCTGTCCGTGGCATTCGCCGGGGACAGTGACGATGGAATGCAATATGTCCTGGATGGATGCCCAAGTCTGCGCAAACTGGAGGTTAGAGATTGTCCTTTCGGTGACGAGGCCCTCTTGACAGGCATCGAGAAGTACGAGTCCATGCGTTCGTTGTGGATGTCGTCGTGCCATCTCACCCGGGATGGTTGTCAATTTTTGGCATCCCACAATTCATCGCTCAACGTCGAAATTATCAAGGATGTGGACAAAGCTCCTCTTGAGCAAGGTCAGTATGTAGAGAAGTTGTACGTTTACCGCACCATTGCAGGGCCCCGTGCTGATGCCCCGCACTTCGTAGAAACCTTGTAG

>PpCOI10 PP1S196_87V6.1

ATGCTCTCCGTTTTCCCAGACGAGGTGCTAGAGCACGTGTTAGTATTTCTGACTGACCACAGGGATAGAAACTCTGTGTCCCTGGTGTGCAAAGCGTGGTGCAGGACAGAAGGATGGAGCAGGCGGAGTGTTTTCATTGGGAACTGTTATGCAGCCTCCCCAAACCTCCTGTTGAGGAGGTTCCCCAAGTTAACGTCTTTAGAGATGAAAGGGCGGCCTAGGTTCACTGACTTTGGGCTGGTGCCTTCGAATTGGGGGGCATTCATCCAGCCATGGATCGAAGCCTTGGCCGAGCATTACGCTGGATTGGAATGTCTCCGTTTGAAGCGCATGACTGTGTCTGACGAAAGTTTACGGATTATAGCTCTTGCCTTCCCCAATTTTCGTTCTCTGCGCCTTGCCAGCTGTGATGGATTTACTACCGATGGTCTTCAGTGGATAACGAGACACTGCAGGCATCTCAAGGAGCTGGATTTGCAAGAAAATGAAATCCAAGTACGCAGTGTAGGCTGGCTGACGGCCTTTCCTGAGTCCCAAACAACTCTGGAAAGTCTCAGTTTTGCGAACATCCAAACCCCGTTGGATGAATACGATTTTCACTCTTTGTATGCCTTGGTAGCTCGTTGTCCAAGACTGAAAAGGTTGAAGTTGAACAGAGAGGTCACTCTGGAGCAGATGCAGAAGCTTCTTCTGCTAGCTCCTCAGCTTGAAGATTTGGGCACAGGGGCATATAACCAGAAGCTGACGTGGGGTAAACTTCATGACCTTCAGGCTTCATTTAGGAAGGTTAAGAATATCCGGTCGCTTTCTGGGTTTTGGGATGTAAGTCCAAGGTGCTTACCGACATGTTTCCCAATTTGTAACGAGCTGATCACCCTAGATCTCTCAACTGTTGCACTCACCACAGCTGATTTTACAAAGTCTACTACAAATTGCGTCAAGCTCCGGCGCCTACTGGTACAAGATTCTGTGGGAGATGAGGGTTTACTTCACGTAGCACGTTGCTGTAAGCAGCTGACGGAGCTTCGTGTCTATCCTTTTAACAATCAAAGTAACGTTACAGAGAAGGGTTTTATTGCAATCTCCGAGGGATGCCGAGACATGCGTAAGATCCTTTACTTTTGCAAGCAGATGTCAAATGCTGCAATGATTCAGTTCGCCAGGAACTGCCCGAATATGACCCATTTCCGGATGGCCATGGTGACGGTCTACGACCGAGACTGTGTCACCAACGACCCTCTGGACGAAGGGTTCGGCGCGGTGTGCAAGCTGTGCAAAAACCTTCGCCGCCTATCTCTCTCGGGGCTCTTAACCGACAAGACCTTTGAGTATATCGGCATGTATGCGAAGAAGTTGGAGACTTTGTCAGTAGCCTTTGCAGGCGATACTGATTTGGGAATGGTCAACGTTCTGGACGGTTGCCCAGCTCTGCGCAAGTTGGAGGTTCGAGACTGTCCCTTTGGGGACGAGGCTCTATTGTCTGGCATCGAGAAGTACGAGTCCATGCGCGCTTTGTGGATGTCTTCATGCCAACTTACCAGGGATGGTGTCCAGTTCCTGGCTGACAAGAACCCTAACCTGAACGTGGAAATCATCGTGGATGTTGAGAAGTCACATGACCCAGAGTACGTTGAGAAGTTGTATGTTTACCGTTCTATTGCAGGCCCTCGGGAGGATGCCCCGTATTTCGTAGATACATTGTGA

>PtCOI1 POPTR_0004s03400.1

ATGGGTCCAAATCCAAAGATGAAAAGGGAATTTCTTGACTCAACAAGGTCATCACCATTCCCAGATGAAGTACTGGAACGAGTTCTTTCACTCTTGAAATCACACAAAGATCGTAGCGCAGTGTCTTTAGTATGCAAGGACTGGTACAACGCAGAAAGCTGGTCAAGAACTCATGTTTTTATAGGGAACTGTTATTCAGTCTCTCCTGAAATTGTTGCACGTAGATTTCCAATAATTAAGAGTGTTACTCTTAAAGGGAAGCCAAGATTTTCTGATTTTAATCTAGTGCCTGAAAATTGGGGAGCTGATGTTCATCCTTGGCTTGTAGTTTTTGCAACGAAGTATCCGTTTTTGGAAGAGCTGAGGCTTAAGAGAATGGCTGTTAGTGATGAAAGTTTGGAGTTTTTGGCTGTTAATTTTCCTAATTTTAAGGTTCTTTCTCTGTTGAGCTGTGATGGGTTTAGTACTGATGGGCTTGCTGCTATTGCCACTCATTGCAAGAGTTTGACTCAGCTTGACATACAAGAGAATGGCATTGATGACAAGAGTGGTGGTTGGTTAAGCTGCTTCCCTGAAAACTTCACATCATTGGAAGTACTAAACTTTGCCAATCTGAATACTGATGTCAATTTCGATGCACTTGAGAGACTTGTAAGTAGGTGCAAGTCACTAAAGGTTCTGAAGGTCAACAAAAGTATTTCCTTGGAACACCTACAAAGGCTGTTAGTTTGTGCTCCACAATTAACAGAGCTTGGCACTGGTTCATTCACACCAGAGCTCACAACTCGTCAGTATGCGGAGCTTGAAAGTGCATTTAACCAGTGCAAGAATTTACATACCCTTTCAGGTTTATGGGAGGCAACAGCACTATATCTACCAGTTCTGTACCCTGTCTGTTCAAACTTGACTTTCTTAAACCTGAGCTATACTTTTCTGCAAAGTCTTGAACTTGCTAGTCTTCTCCGTCAATGTCCACGACTTAGACGCCTCTGGGTCCTGGACACTGTGGGGGACAAAGGGCTGGAGGCTGTTGGATCCAACTGTCCATTGCTTGAGGAGCTCCGTGTCTTCCCTGCCGATCCCTTTGATGAGGAAATTATCCACGGGGTGACTGAAGCAGGGTTTGTTGCTGTCTCTTATGGATGTCGAAGACTCCACTATGTTCTCTACTTTTGCCGGCAGATGACTAATGCTGCAGTAGCAACCATTGTGCAGAACTGCCCTGATTTCACCCACTTTCGTCTTTGCATAATGAATCCAGGCCAACCGGATTACTTGACAAATGAACCTATGGACGAGGCTTTTGGGGCTGTGGTGAGGACTTGCACTAAACTACAGAGGCTTTCTGTTTCAGGTCTCTTGACAGACCTGACATTTGAATATATTGGGCAGTATGCCAAAAATCTGGAAACTCTGTCTGTGGCTTTTGCGGGCAGCAGTGATAGGGGGATGCAGTGTGTGCTAGAAGGTTGTCCAAAGTTGAGGAAACTTGAGATAAGGGACTGCCCATTTGGAAATGCAGCACTTCTTTCAGGTTTGGAGAAGTACGAGTCTATGAGGTCACTCTGGATGTCAGCCTGCAATGTGACAATGAATGGCTGTCGGTTATTGGCAAGGGAGATGCCCAGATTGAATGTTGAAGTAATGAAAGAGGATGGAAGTGATGACTCTCAGGCTGATAAAGTTTATGTTTACCGTTCTGTTGCGGGGCCAAGAAGGGATGCTCCACCTTGTGTACTCACTCTCTCAGGTTTATAA

>PtCOI2 POPTR_0002s10310.1

ATGATCACCAACAAAAAGCCTAGATCATCAGACACTGACTCTAATTATATGAGAGACGATCGAACTGACATGTCAGAAGACGACGACCGATCTCCGCCGTCGGATTCAATCGCCAACGACTCTTGCCCAACACGGACCTGCACTCCTGGGTCTGGGTCGGGTTCGTCTTCCATCCCCGAATACTCAGCTCCATACCCGGACCAAGTCCTCGAAAACGTCTTAGAAAACGTTCTCTGGTTCTTAACCTCACGTAAGGACCGAAACGCTGCGTCATTGGTTTGTAGGTTATGGTACCGGGTCGAGGCTATGACCCGATCCGATTTGTTTATCGGTAACTGCTACGCGGTGTCTCCAGAACGCGCTACGTCGCGGTTTACCCGAATCCGTTCGGTGACGCTAAAAGGAAAGCCAAGGTTTGCTGATTTTAACCTGATGCCGCCTAATTGGGGAGCCCACTTCGCGCCTTGGGTCTCTGCTATGGCAAAGGCTTACCCTTGGTTAGAGAAGATTCATTTGAAGAGGATGTCAGTGACGGATGATGATCTGGCTTTGCTTGCGGAGTCATTTTCGGGGTTCAAAGAGCTCGCACTTGTTTGCTGTGATGGGTTTGGTACTAGTGGACTGGCTGTTGTGGCTAGTAAGTGCAGGCAACTCAAAGTGCTTGATCTGATTGAATCAGAAGTATCGGATGATGAAGTGGATTGGATTTTGTGTTTTCCAGATACCGAAACATGTCTTGAATCCCTGATTTTAGATTGTGTAGAATGTCCCATTGATTTTGATGCACTGGAGAGGCTGGTGACTAGGTCCCCATCACTTAAGAAACTTAGGCTAAACAGGTTCGTTTCAATTGGGCAACTATACCGTTTAATGGTTCGAGCACCGCAGCTCACACATCTTGGGACAGGCTCATTTAGCCAATCGGAGGATGTGGCTCAAGGTGAACTGGAACTAGATTATGGCTCTGCGTTTGCTGCTTGCAAATCATTAGTTTGCCTATCTGGATTCAGGGAAATCATTCCAGATTATTTGCCTGCAATATACCCCGTCTGCGCCAATCTCACTTCACTGAACTTTAGTTATGCAAATATCAGCGCAGAACAGCTCAAACCAATTATAAGCAATTGCCACAAGCTTCAGACTTTCTGGGTTCTTGATTCAATATGCGATGAAGGACTTCAGGCTGTGGCTACAACTTGCAAGGAACTACGTGAGCTTCGGGTTTTCCCTTTTGAAGCTAGGGAGGACATCGAGGGCCCTGTTTCTGAAGTGGGCCTCCAAGCGATTTCAGAGGGTTGCAGGAAGCTCCAATCTATTTTGTATTTTTGCCCGCGGATGACAAATGCTGCTGTTATAGCTATGTCAAAGAACTGCCCAGACCTTGTGGCCTTCCGTCTCTGCATAATGGGACTCCACCAGCCTGATCATGTCACTGGAGAACCTATGGATGAAGGGTTTGGAGCCATTGTCATGAATTGCAAGAAGCTCACTCGACTTGCAGTATCTGGTTTATTGACTGATAGAGCTTTTGCTTACATTGGAAAATATGGGAAGATTGTAAGGACATTATCGGTTGCTTTTGCTGGTGATAGTGACATGGGGTTGAAGTATGTGCTTGAGGGCTGTCCCAAATTACAGAAGCTTGAGATTAGAGACAGTCCATTCGGGGATGCAGCTCTACTTTCTGGTCTGCACCACTATTACAATATGAGATTCCTTTGGATGTCCGCTTGCAAGTTGTCCCATCAGGGATGCCAACAGATTGCTCAAGCATTGCCTCACCTGGTGGTGGAAGTGATTAAGCATGAAGATAATGTGGACATGGATGAGTATGTTGATACATTGTACATGTATCGGTCTCTTGCAGGGAGAAGACATGATGTGCCACGATTTGTTTCCATCTTGTAA

>PtCOI3 POPTR_0001s33030.1

ATGAATTATTTCCCTGATGAAGTATTAGAGCATATTTTCGATTTTGTAACATCACAAAGAGACAGGAACTCAGTGTCTCAAGTGTGTAAACCATGGTACAAAATCGAAAGTACTAGCAGGCAAAAGGTTTTTGTAGGGAATTGTTATGCAATTAGTCCTGAGAGAGTGATTGAGAGGTTTCCAGGTTTGAAATCTATCACTTTGAAAGGAAAGCCTCATTTTGCTGATTTTAATTTGGTTCCTCATGATTGGGGAGGCTTTGTTTATCCATGGATTGAAGCTTTTGCAAGGAATAATATGGGGTTAGAGGAGCTCAAGTTGAAGAGGATGATAATATCCGATGAGTGCTTGGAGCTGATTTCAAGGTCTTTTGCCAATTTCAAGTCCTTGGTTCTTGTTAGTTGTGAAGGCTTCAGCACTGATGGCCTTGCTGCTATTGCTTCTAATTGTAGGTTTCTGAGGGAGCTGGACCTGCAAGAAAATGATGTCGAGGATCATAGAGGCCATTGGCTTAGCTTCTTTCCTGACACTTGTACATCTCTTGTATCACTTAATTTTGCATGTCTCAAAGGAGATGTCAATTTAGCAGCTCTTGAGAGACTTGTAGCTAGATCTCCTAATCTGAGGAGTTTGAGGTTAAATCATGCCGTGCCACTTGATATACTTCAAAAAATATTGATGAGAGCACCTCATTTAGTGGACTTGGGTGTAGGGTCTTACGTGCATGATCCAGATTCTGAGACCTATAATAAATTAGTGACTGCTCTTCAAAAGTGTAAGTCAGTCAAGAGTTTGTCAGGGTTTCTGGAGGCTGCTCCTCAATGCCTATCGGCTTTTCATTTAATTTGCCCGAACCTGACTTCCTTGAACCTAAGCTATGCTCCAGGAATTCATGGTACTGAGCTCATAAAGCTAATTCGTCACTGCAGGAAACTCCAGCGCTTATGGATACTGGACTGCATTGGAGATGAAGGACTAGAAGTTGTAGCTTCCACTTGCAAACATTTGCAGGAAATAAGGGTCTTTCCTTCTGATCCATTTGTTGGGAATGCAGCTGTGACTGAAGTGGGCTTGGTTGCTCTTTCAAGTGGTTGCCGCAACCTTCACTCAATCCTATACTTCTGTCAGCAGATGACCAATGCAGCCCTCATAACTGTAGCTAAGAACTGCCCCAATTTTACCCGTTTCAGGTTGTGCATCCTTGACCCCACAAAACCGGACGCTGATACCAATCAGCCATTGGATGAAGGTTTTGGGGCTATTGTTCACTCATGCAAGGGGCTCAGGCGGTTGTCAATGTCTGGTCTGCTGACTGATCAAGTTTTCCTCTACATTGGAATGTATGCCGAGCAGCTTGAAATGCTTTCTATTGCATTTGCTGGGGACACTGACAAGGGAATGCAGTATCTATTGAATGGTTGCAAGAAACTTCGCAAGCTTGAGATAAGGGACTGCCCTTTTGGTAATGCAGCACTTTTAATGGACGTGGGAAAGTATGAAACAATGCGATCCCTTTGGATGTCATCCTGCGACATTACCCTTGGAGGCTGCAAGTCCCTTGCGAAGAAGATGCCAAGGCTCAATGTGGAGATCATAAATGAAAGTGACCAGATGGATATTACCGCTGATGATGGGCAAAAGGTAGAGAAGATGTTCTTGTATCGGACTTTGGCAGGGCGAAGGAAAGATGCACCAGAGTTCGTGTGGACTTTATAG

>PtCOI4 POPTR_0020s00280.1

ATGCCGAACAAGGCGAGTACGTTCCCGGAGGAAGTGCTAGAGCATGTGCTCTCGTTTATTACAAACGACAAAGACAGGAACGCCGTATCGGTGGTGTGCAAGTCGTGGTACGAGATAGAGCGGTGGTGCAGGAAGAGAATATTTGTTGGTAACTGTTATGCGGTGAGGCCTGATATGGTTATAAGGAGGTTTCCGGAGCTAAGATCGGTGGAGCTCAAAGGGAAGCCACACTTTGCGGACTTTAATTTGGTGCCTGACGGTTGGGGAGGTTACTTTTACCCATGGATAGCGGCGTTGGCTACCGCCTATCCTTGGTTGGAAGAGATTAGGTTGAAGAGGATGGTGATTTCCGACGAGAGTTTGGAGTTTATTGCTAAGAGTTTTAAAAATTTTAAGGTTTTGGTGCTTTCGTCTTGTGAGGGTTTCTCTACGGATGGACTTTCTGCCATTGCAGCCGATTGCAGGAATCTGAGGGAGCTGGATTTACGAGAGAGTGAGGTGGATGATCCAAGTGGGCAGTGGTTGAACAGTTTTCCTGACTCGTTTACATCACTGGTCTCTCTTAATATTTCCTGCTTAGGGGCTGAGCTGAGTTTCTCAGCTCTGGAGCGCCTGGTTGGCCAGTGTCCTGATCTAAAGAATCTTCAGCTCAACCATGCTGTGCCCGTTGAGAGGCTTGCTAAACTTATTCGCCAAGCACCACAACTTGTTGAATTGGGCACAGGTGAGTTCTCGGCTAAGTTGCAGCCTGAAATCTTCTCAAACCTGGCTGGAGCTTTTTCTGTGTGTAAAGAACTGAGGAGTCTATCTGGGTTTTGGGATGTAAATCCAGCTTACCTTCCAGCAGTTTATCCTGTCTGTTCTGGCTTAACATCACTGAACTTGAGATATGCCAATATACAAAGCGCTGATCTCATTAAACTTGTCAGTCAATGTTCGAATCTGCAGCGTTTATGGGTGCTGGATTACATTGAAGACGTTGGACTTGAAGCTCTTGCAGCATGTTGCAAGGACCTGACAGAGTTGAGGGTGTTTCCATCTGACCCATTTGCTGCAGAACCAAATGTATCCTTGACAGAAAGGGGCCTTGTCTCTGTCTCTGAAGGCTGTCCTAAGCTTCAGTCAGTTCTGTACTTTTGCCGTCAAATGACTAATGCTGCTCTAGTTACAGTAGCAAAGAACCGTCCAAGCATGACCTGCTTCCGTCTTTGTATTATTGAACCTCAGGCTCCTGATTACCAAACACTTCAGCCTCTGGATTTGGGTTTTGGAGCCATTGTTGAAAACTACAAGGATCTCCGGCGTCTTTCCCTCTCAGGTCTACTGACTGATCGTGTGTTTGAGTACATTGGAACTTATGGAAAAAAAATAGAGATGCTATCTGTGGCATTTGCTGGCGATAGTGATCTGGGACTCCACCATGTGCTGTCTGGGTGTGAAAGGCTTTGCAAACTTGAAATTAGGGACTGTTCCTTTGGGGATAAGGCTCTTTTGGCCAATGCTGCAAAGCTGGAGACAATGCGATCCCTTTGGATGTCTTCTTGCTCAGTGAGTTTTGGAGCATGTAAGCTGCTAGGTCAGAAGATGCCTAGGCTCAATGTTGAAGTTATTGATGAGAGGGGACCTCCAGAATCGAGGCCGGAAAGCTGCCCTGTTGAGAAGCTTTACATATACAGAACCATTGCAGGACCTAGGCTTGACATGCCTGGATTTGTTAGGACTATGGATGCAGATTCTGTGTCAAGGTTTTGTTGA

>SbCOI1 Sb09g022040.1

ATGGGCGGTGAGGCGCCGGAGCCCCGGCGGCTGACCCGCGCGCTGAGCATCGGCGGCGGCGACGGCGGCTGGGTCCCCGAGGAGATGCTGCACCTGGTGATGGGGTTCGTCGAGGACCCGCGCGACCGGGAGGCCGCGTCGCTGGTGTGCCGCCGGTGGCACCGCGTCGACGCGCTGTCGCGGAAGCACGTCACGGTGCCCTTCTGCTACGCCGTGTCCCCGGCGCGCCTGCTCGCGCGGTTCCCGCGGCTCGAGTCGCTGGCCATCAAGGGGAAGCCCCGCGCGGCCATGTACGGCCTCATACCGGACGACTGGGGCGCCTACGCCCGCCCCTGGGTCGCCGAGCTCGCCGCGCCGCTCGAGTGCCTCAAGGCGCTCCACCTCCGACGCATGGTCGTCACGGACGACGACCTCGCCGAGCTCGTCCGTGCCAGGGGACACATGCTGCAGGAGCTCAAGCTCGACAAGTGCACCGGCTTCTCCACGGATGGACTCCGCCTCGTTGCGCGCTCCTGCAGATCACTGAGAACTTTGTTTCTGGAAGAATGTCAAATTAATGATAAAGGCAGTGAATGGATCCACGATCTTGCAGACGGTTGTCCTGTTCTGACAACATTGAATTTCCACATGACTGAGCTTCAAGTGATGCCAGCTGACCTAGAGTTTCTTGCAAGGAGCTGCAAGTCACTGATTTCCTTGAAGATTAGCGACTGTGATGTTTCAGATTTGATAGGGTTCTTCCAATTTGCCACAGCACTGGAAGAATTTGCTGGAGGGACATTCAATGAGCAAGGGGAACTCACCATGTATGGGAATGTCAGATTTCCATCAAGACTATGCTCCTTGGGACTTACTTTCATGGGAACAAATGAAATGCCTATTATATTTCCTTTTTCTGCAATACTGAAGAAGCTGGATTTGCAGTACACTGTCCTCACCACTGAAGACCATTGCCAGCTTATTGCAAAATGTCCGAACTTACTAGTTCTCGCGGTGAGGAATGTGATTGGAGATAGAGGATTAGGAGTTGTTGCAGATACATGCAAGAAGCTCCAAAGGCTCAGAATTGAGCGAGGAGACGATGAAGGAGGTGTGCAAGAAGAGCAGGGAGGGGTCTCTCAAGTGGGCTTGACGGCTATAGCCGTCGGTTGCCGTGAACTGGAATACATAGCTGCCTATGTGTCTGATATAACCAATGGGGCCCTGGAATCTATCGGGACATTCTGCAAAAAACTCTATGACTTCCGGCTTGTTCTGCTTGATAGAGAAGAGAGGATAACAGAATTGCCACTGGACAATGGTGTCCGAGCTTTGTTGAGGGGCTGCACCAAACTTCGGAGGTTTGCTCTGTACTTGAGACCAGGAGGGCTCTCAGATGCAGGTCTCGGCTACATTGGACAGTGCAGTGGAAACATCCAATACATGCTTCTCGGTAATGTTGGGGAAACTGATGATGGATTGTTCAGTTTCGCATTGGGATGCGTAAACCTGCGGAAGCTTGAACTCAGGAGTTGTTGCTTCAGCGAGCGAGCTCTGGCCCTCGCCATACTACGCATGCCTTCCCTGAGGTACGTATGGGTTCAGGGCTACAAAGCGTCTCAAACCGGCCGAGACCTCATGCTCATGGCGAGGCCCTTCTGGAACATAGAGTTTACACCTCCCAGTTCCGAGAACGCAGGTCGGTTGATGGAAGATGGGGAACCTTGTGTAGATAGTCATGCTCAGATACTCGCATACCACTCCCTCGCCGGTAAGAGGTTGGACTGCCCACAATCCGTGGTCCCTTTGTATCCTGCCTGA

>SbCOI2 Sb03g040150.1

ATGGGCGGCGAGCTGCCGGAGCCGAGCCGGCTGAGACGCGCGCTCAGCTTCGGCTGCGGCGCCGTCCCCGAGGAGGCGCTGCACCTCGTGTTCGGCTACGTGGACGACCCGCGCGACCGGGAGGCGGCCTCGCTCGTGTGCCGCCGCTGGCACCGCATCGACGCGCTCTCGCGCAAGCACGTCACCGTCGGCTTCTGCTACGCCGTGGAGCCCGCGCGGCTGCTCGCCAGGTTCCCCAGGCTCGAGTCGCTCGCGCTCAAGGGGAGGCCCCGCGCCGCCATGTACGGCCTCATCCCCGAGGACTTCGGCGCCTACGCCGCGCCCTGGGTCGCCCAGCTCGCCGCGCCGCTCGACTGCCTCAAGGCGCTCCACCTGCGCCGCATGACCGTCACCGACGAGGACATCGCCGTGCTCGTCCGCGCGCGCGGCTACATGCTACAGGTGCTCAAGCTCGACAAGTGCTCCGGCTTCTCAACCGACGCCCTCCGCCTCGTCGCCCGCTCCTGCAGATCTCTGAGAACTTTGTTCCTGGAAGAATGTACAATTGCCGATGAAGGGAGTGAATGGCTCCATGAACTCGCTGTCAACAATTCTGTTCTGGTGACACTGAACTTCTACATGACAGATCTCAGAGTGGAGCCTGCTGATCTGGAACTTCTTGCAAAGAACTGTAAATCATTGATTTCTTTGAAGATGAGTGAGTGTGATCTTTCAGATCTGATTGGTTTTCTCCAAACCTCCAAAGGATTGCAAGAATTCGCTGGAGGTGCATTTTCTGAAGTTGGAGAGTACACCAAATACGAAAAGGTCAAGTTCCCACCTAGACTATGCTTCTTGGGGGGTCTTACCTTCATGAGTAAAAATGAGATGCAAGTTATCTTTCCATATTCTGCAATGCTTAAGAAACTGGACTTGCAGTACACTTGCCTCACCACTGAAGATCACTGTCAGCTTATTGCTAAATGCCCCAACCTACTGGTTCTCGAGGTGAGGAATGTGATTGGGGATAGAGGACTAGAAGTTGTTGGTGATACATGCAAGAAGCTACGAAGACTCAGAATTGAGCGGGGGGATGATGATCCCGGTCAAGAAGAGCAGGGAGGAGTCTCTCAGATAGGTTTGACAGCCGTAGCTGTTGGCTGCCGTGAACTGGAGTACATAGCTGCCTATGTATCTGATATCACAAATGGGGCGCTGGAATCCATTGGCACGTTCTGCAAGAATCTCTATGACTTCCGGCTTGTTCTGCTTGACAAACAGAACAAAATAGCAGATCTGCCACTTGACAACGGCGTCCGTGCTCTATTGAGGAATTGCACAAAACTTCGGAGGTTTGCCTTCTACCTGAGACCGGGAGGGCTTTCAGATGTAGGCCTTGGTTACATTGGACTGTACAGTGGAAACATCCAATACATGCTGCTCGGCAACGTTGGCGAATCTGACAATGGATTGATCCAATTTGCAATGGGATGCACCAACCTGCGGAAGCTCGAGCTGAGGAGCTGCTGCTTCAGCGAGCGAGCTCTGGCAGTGGCCGTGCTCCAGATGCCCTTGCTGAGGTACATATGGGTGCAGGGGTACAGAGCCTCTCAAACAGGTCAGGACCTCATGCTCATGGCCAGGCCATACTGGAACATTGAATTTGTGCCTCCCGGTCCCGAGAGCGCCTATCGTGTGATGGCAGATGGACAGCCTTGTGTTGACACCCATGCCCAGGTTCTTGCGTATTACTCCCTTGCTGGAAGGAGGCCGGACTGTCCTCAGTGGTTGGTTACTTTGCATCCTGCGTGA

>SbCOI3 Sb01g040110.1

ATGGGCGGCGAGGCCGAGGGCGGGGAGCGGCGGCTGGGGCGGGTGCTGAGCTTTGGGATCCCGGACACGGCGCTGGGGCTAGTGATGGGGTACGTGGAGGACCCCTGGGACCGCGACGCCATCTCGCTGGTGTGCCGCCACTGGTGCCGCGTCGACGCGCTGAGCCGCAAGCACGTCACCGTGGCCATGGCCTACTCCACGACACCCGAGCGCCTGTTCCGCCGCTTCCCGTGCCTCGAGTCACTCAAGCTCAAAGCAAAGCCCCGCGCGGCCATGTTCAACCTCATCTCCGAGGACTGGGGCGGGTCTGCTTCGCCGTGGATCCAACAGCTCTCGGCCACCTTCCACTTCCTCAAGAAGCTCCACCTGCGCAGGATGATAGTATGCGACGACGATATCAACATCCTCGTCCGCGCCAAGGCGCACATGCTCGTCGCGCTGAAGCTCGACCGCTGCTCCGGCTTCTCCACGGCCTCCATCGCACTCATCGCCCGCTCCTGCAAGAAACTGGAAACACTTTTCCTGGAAGAAAGCACGATTGATGAGAGAGACAATGATGAATGGATCCGTGAGCTTGCTACGAGCAATTCTGTTCTTGAGACACTGAATTTCTTTCTGACAGATCTCAGGGCATCCCCAGAGTATCTTACCCTCCTTGTGCGCAACTGTCAAAGGCTGAAAACCCTGAAGATTAGTGAATGTTTCATGCCTGATCTGGTTAGTTTGTTCCGAACTGCACAAACACTACAAGAGTTTGCTGGTGGTTCCTTTGAAGAGCAGGGTCAACCTGTGGCAAGTAGAAATTATGAGAACTACTATTTTCCTCCTTCGCTGCACCGCTTGAGTTTGCTGTACATGGGAACAAATGAGATGCAGATACTGTTTCCATATGCTGCTGCACTTAAGAAGTTAGACCTTCAGTTTACATTCCTTTCCACAGAGGAGCACTGTCAGATAGTTCAGCGCTGCCCCAATCTGGAAACCTTAGAGGTGAGGGATGTCATAGGGGATCGTGGACTACAAGTTGTTGCGCAGACCTGCAAGAAACTGCAGAGGCTCAGAGTAGAGAGAGGAGATGATGATCAAGGAGGTCTTGAGGATGAACAAGGTAGGATTTCACAGGTTGGGTTGATGGCTATAGCCCAAGGCTGCCCTGAGTTGACATACTGGGCAATACATGTATCGGACATTACAAATGCAGCTCTAGAGGCGGTTGGTACATGCAGCAAAAATCTTAATGATTTCCGCCTTGTCCTTCTTGATAGAGAAGCACATATAACTGAATTGCCACTGGACAATGGGGTTCGTGCTTTGCTTAGAGGTTGCACCAAACTCCGGAGGTTTGCATTTTATGTGAGACCTGGGGCCCTATCTGATGTTGGTCTTGGCTATGTTGGAGAATTTAGCAAGAGCATTCGTTATATGTTGCTTGGTAATGTTGGTGAATCTGATAATGGAATTATACAATTATCAAAAGGCTGCCCAAGCTTGCAAAAACTGGAGCTGAGGGGTTGTTTCTTTAGTGAGCATGCTTTAGCTATGGCTGCACTAGAGCTCAAGTCACTGAGGTATCTGTGGGTGCAAGGATTCAGGACATCTCCAACTGGAACTGATCTTATGGCAATGGTACGCCCCTTCTGGAACATTGAGTATATTGTTCCCGACCAAGATGAACCTTGCCCAGAGCATCAGAAACAGATTCTGGCATACTACTCCCTTGCTGGAAGGAGGACAGATTGTCCTCCATCGGTAACTCTGCTTTACCCGGCATTTTGA

>SbCOI4 Sb04g033850.1

ATGAGCACTCCCCACTCGTCCTCCACCTCCTCCGTCCCCCACCGCGCCTCCCCTCCCCAGATCCCCCCATCCCTAACCCTAGCTCCCACGGCGTCCTCTGCCTCGCCGTCCTCCTCCTCCGCGTCCGGCATGCGGGACGCGGCGGAGGACGACTCCGACTCGCCGCCGTCGCAGATGTCGGAGGACGACCCCGGCGGGGGCGGGGACAGGTGGGAGCCGGATCTGAGGGGCGGCAACAACGGCGGGGGCGGGAGGTGGGCGCCGCCGGACCAGGTGCTGGAGAACGTGCTCGAGACCGTGCTCGAGTTCCTCACCGCCGCGCGGGACCGCAACGCCGCCTCGCTCGTCTGCCGTTCCTGGTACCGCGCAGAGGCGCAGACGCGCCGCGAGCTCTTCATCGGCAACTGCTACGCCGTCTCGCCGCGACGCGCCGTCGAGCGCTTCGGCGGCCTGCGCGCCGTCGTGCTCAAGGGCAAGCCCCGCTTCGCGGACTTCAGCCTCGTGCCATACGGCTGGGGCGCCTACGTCTCCCCCTGGGTGGCAGCGCTCGGGCCCGCGTACCCGCGCCTCGAGCGCATCTGCCTCAAGCGGATGACCGTCTCCGACGACGACCTCGCACTCATCGCCAAGTCCTTCCCCTTGTTCAGGGAGCTCTCGCTAGTGTGCTGCGATGGCTTCAGCACGGTCGGGCTCGCCGTCATCGTCGAGCGCTGCCGGCATCTTCGCGTGCTGGATCTGATTGAAGATTACCTTGAGGATGAAGAGGACGAGCTGGTGGATTGGATCTCCAAGTTTTCAGAGTCCAACACCTCTCTGGAGTCACTTGTCTTCGACTGCGTCAGTGTGCCCTTCAACTTTGAGGCCCTCGAGGCACTTGTTGCACGCTCACCGGCTTTGCGTCGGTTGCGTGTCAACCACCACGTGTCAGTAGAGCAGCTGCGCCGTCTCATGGCAAGAGCACCCCAGCTCACACACTTTGGAACCGGTGCATTCCGATCTGAGGGTGCCCCTGATGGGGGTCTGGCTGTGACTGAGCTTGCTACATCTTTTGCTGCAGCCAGGTCTCTCGTTTGTCTATCAGGTTTTCGGGAGGTCGATCCACAATATCTCCCAGCGATCTACCCTGTTTGTGCCAAGCTCACCTCACTAAACTTCAGCTTTGCAAGCCTAACGGCTGCGGAACTCAAGCCAGTTATTCGCAACTGCACCAATCTTCGCACTTTCTGGGTCCTTGATACTGTGGGTGATGAAGGCCTACGTGCTGTGGCTGATACATGCTCTGATCTCCGTGAGCTGAGAGTTTTTCCTTTGGATGCCTCTGAAGATTCTGAGGGTTCGGTCTCAGATGTTGGTCTTCAGGCAATATCAGAAGGCTGCCGGAAGCTAGAATCAATCCTCTACTTTTGCCAGCGGATGACAAATGAAGCAGTAATTGCTATGTCGAAGAACTGTCCTGAGCTTGTAGCGTTCCGCCTCTGTATTATGGGCCGCCACCGCCCTGATCGAGTCACTGGGGACCCAATGGATGAAGGTTTTGGCGCAATTGTGATGAACTGCAAGAAACTCACCAGGCTTTCAGTCTCTGGCCTGCTTACTGATAAAGCCTTTGCATACATTGGAAAATATGGGAAGCTAATAAAGACTCTGTCTGTTGCCTTCGCTGGGAATAGTGACATGTCCCTCCAATATGTATTTGAGGGATGCACTAAGTTGCAGAAGCTTGAGGTCAGAGATAGCCCTTTTACTGACAGGGGGTTACTCTCCGGACTGAACTACTTCTACAATATGAGGTTCTTATGGATGAACTCATGCAGGCTAACTATGAGAGGATGTAAAGATGTAGCTCAGCAAATGCAAAATTTGGTGGTTGAAGTAATTAAGGACCACTCTGAAGATGAAGGGGAGGCTGAGATTGTTGACAAGTTGTACTTGTATCGGTCACTGGCAGGACCAAGGAATGATGCTCCACCATTTGTTACCCTCTTGTAG

>SbCOI5 Sb06g014420.1

ATGACCTACTTCCCTGAGGAGGTGGTGGAGCACATTTTCAGCTTCTTGCCATCGCACAGCGACCGGAACACGGTCTCCCTCGTGTGCAAGGTGTGGTATGAGGTCGAGAGGCTGAGCCGACGAGCTGTGTTCGTGGGGAACTGCTACGCTGTGCGCCCCGAGCGTGTGGTGCTGCGGTTCCCCAATGTGAAGGCGCTCACGGTGAAGGGGAAGCCTCACTTTGCGGACTTCAACCTTGTGCCGCCTGACTGGGGTGGCTACGCGGGGCCATGGATCGAAGCAGCAGCGAGGAGCTGCGTGGGTCTTGAGGAGCTGCGCATGAAGCGGATGGTTGTGTCTGACGAGAACCTTGAGCTGCTAGCTCGGTCATTCCCAAGATTCAAAGTCCTCGTTCTTATCAGCTGCGAGGGGTTTAGCACTGATGGTCTAGCTGCTGTTGCGAGTCACTGCAAGCTCCTGAGGGAGTTAGATTTGCAGGAAAATGACGTGGAGGACCGTGGGCCCAGATGGCTTTCCTTCTTCCCTGATTCCTGCACTTCTCTGGTCTCCTTGAATTTCGCCTGCATCAAAGGGGAGGTGAACTCTGGTGCATTGGAGAGACTTGTCGCTAGGTCTCCGAACCTACGCAGTTTAAGGTTGAATCGTTCTGTTTCAGTAGACACGCTCTCAAAGATACTAGCGCGAACCCCAAATTTGGAAGACTTGGGGACAGGTAATTTGACAGATGAGTTCCAAGCTGAGTCCTACGCCAGGCTTACCAGTGCTCTGGAGAAATGCAAAATGCTAAGAAGTTTGTCTGGATTTTGGGATGCTTCTCCTATCTGTGTTCCATATATCTATCCCCTCTGTCATCAACTAACAGGCCTAAACTTGAGCTATACTCCTACACTGGATTATTCTGATCTGACTAAAATGGTTAGCCGCTGTGTGAAACTCCAGCGTCTTTGGGTATTGGACTGCATTTCGGATAAGGGTTTGCAAGTGGTGGCCTCCAGTTGCAAGGATCTACAAGAACTCAGGGTGTTTCCATCAGATTTTTATGTTGCTGGTGCTTCGGCGGTGACAGAGGAGGGACTTGTTGCAATATCATCAGGCTGCCCAAAACTAAGCTCTTTGCTGTATTTCTGTCACCAGATGACCAATGAAGCACTAATTACTGTAGCTAAGAACTGCCCAAATTTCATCAGATTTAGACTCTGTATCCTTGAGCCAAAGAAGCCGGATGCCATGACAGGCCAGCCGTTAGATGAAGGCTTTGGTGCAATTGTCCGTGAGTGCAAAGGGCTAAGACGACTGTCAATGTCAGGTCTTCTCACAGACAGGGTTTTCATGTATATTGGAAAGTATGCAAAATACCTTGAGATGCTTTCTATAGCATTTGCTGGAGATAGTGATAAGGGTATGATGGACGTGATGAACGGATGTAAGAATCTGAGGAAGCTTGAGATTAGAGACAGTCCATTTGGTGATGTTGCACTCTTGGGAAATGTTGCCAAGTATGAGACAATGCGATCCCTTTGGATGTCATCATGTGATGTCACATTAAAGGGGTGCCAAGTCCTTGCATCGAAGATGCCGATGCTCAATGTGGAGATCATGAATGAACTAGATGGAAGTAGTGAAATGGAGGAGAACCACACAGACATCTCTAAAGTGGATAAGTTACATGTTAGTGAAATGGAGGAGAACCACACAGACCTCTCTAAAGTGGATAAGTTATATGTTTACCGCACAACTGCTGGAGCGAGGGATGATGCACCAAATTTTGTTAAAATCTTATAG

>SbCOI6 Sb05g018860.1

ATGGCATACTTCCCTGAGGAAGTAGTGGAGTACATCCTTGGCTATGTAACCTCACACCGGGACCGCAACGCCGCGTCCTTGGTGTGCCGGGTATGGTACGACATTGAGCGCCGTGGCCGCCGTTCGGTGCTTGTAAGCAACTGCTACGCGGTGCACCCAGAGCGTGTACATATGCGGTTTCCCAACATGCGTGCACTGAGTGTGAAGGGTAAACCGCACTTTGCTGACTTCAACCTTGTCCCGGCGGGTTGGGGTGCCAGTGCAGAGCCATGGGTGGATGCGTGCGCCCGTGCATGCCCAGGTCTTGAGGAGCTCCGGCTGAAGCGTATGGTTGTGACTGATGAATGCCTCAAGTTGCTTTCTTGCTCTTTTACCAACTTTGAATCACTTGTCCTTGTCTGCTGCGAGGGGTTCAGTACTGCTGGGCTTGCTAACATTGCCACCAATTGCAGGTTTCTTAAGGAACTCGACTTACAAGAGAGTTGTGTGAAACATCAAGGCCATCAGTGGATTAATTGTTTTCCCAAGCCTTCAACATCACTAGAATGCTTGAATTTTTCTTGCTTGACTGGGGAGGTAAATGCCGTTGCATTGGAGGAACTTGTTGCAAGGAGTCCAAATCTTAAAAGTTTAAGGCTGAATCCTTCAGTTCCAATTGATGTCTTGCCCAGAATCCTTTCTCACACACCTATGCTAGAGGATTTAGGTACAGGATCTTTTGTACTAGGCAATAACGCTGGTGCATATATCAGTCTATACAGAGCTCTTGGAAAGTGCACTTTGCTGAAGAGTTTATCTGGTTTTTGGGATGCTCCGGGCTTGTATGTTCGAGGAATGTTGTTGCCGATTTGCAGGACCAGGGCCCTTACATGCCTGAATCTCAGCTATGCTCCTTTGATTCAGAGTGACCAGCTTATCAGTATTGTTCGTCAGTGTACAAGGCTCCACGTCTTATGGGTATTAGATCACATTGGTGATGAAGGATTGAAGGTTTTGTCCTATTCTTGTCCTGATCTTCAGGAGTTGAGGGTATATCCAAGTGATCCAAATGCTGCAGCAAGAACTAGTGTGACGGAGGAAGGGTTGGCAGCCATATCTTTCTGTCGGAAGTTAGAGTGTGTGCTCTTCTTCTGTGATCGAATGACAAATACTGCGCTCATCACTATAGCAAAGTACTGTCCACTGCTAACATCCTTCAGACTATGCATTCTGGAGCCTAGGTCAGCAGATGCTGTGACAGGGCAGCCACTGGACGAAGGCTTTGGGGCAATAGTGCAGTCCTGCAAAGGCCTGAGGCGTTTCGCCATGTCGGGCCTCCTCACGGACAGTGTGTTCCTGTACATCGGCATGTACGCGGAGAAGCTGGAGATGCTCTCCGTAGCATTCGCAGGGGATACTGACGACGGCATGGTCTACGTGCTCAACGGCTGCAAGAACCTCAAGAAGCTGGAAATCAGGGACAGCCCCTTTGGTGATGCGGCTCTCCTTGCGGGCGCGCACAGGTACGAGTCGATGCGCTCTCTCTGGATGTCGTCCTGCGAGATCACCCTGGGGGCCTGCAAGACCCTTGCGGCGGCCATGCCAAACATCAATGTCGAGGTCATCAGTGAGGCGGGGGCGAGCGTTGGTGCGACGGATGATGGCATCAGCAATGCGAGGAAGGTGGATAAGCTATACCTCTACCGGACTATCGCCGGACCCAGGAGCGATACGCCAGGATTCGTTTCGATATTGTGA

>SbCOI7 Sb01g044720.1

ATGTCAGAGGAGGACGAGGACCAGCCGCCGCCCAAGCGTCCCACCAGCGCGTCGCCCTCGCCGACCGCGGACCAGGTGCTCGACAACGTGCTCGAGACGGTGCTCCAGTTCCTGGACGCCCCGCGGGACCGGAGCGCCGCCTCCCTCGTCTGCCGCTCCTGGCACCGCGCCGAGTCCGCCACCCGCGACTCCGTCGCCGTCCGCAACCTCCTCGCCGCCTCGGCCACCCGCACCGCGCGCCGCTTCCCCAACGCCAGGAGCCTCCTCCTCAAGGGCCGCCCGCGCTTCGCCGACTTCAACCTCCTCCCGCACGGATGGGACGCCTCCGCCTTCCGACCCTGGGCCGCCGCCGTCGCCGCCGGGTCCTTCCCCGCGCTCGCCTCGCTCTACCTCAAGCGCATCCCCGTCACGGACGCCGACCTCGACCTCCTCTCCCGCTCCCTCCCGGCGTCATTCCGCGACCTCACCCTGCACCTCTGCGACGGCTTCACCTCGCGCGGGCTCGCCTCCATCGCCTCCCATTGCAGCGGCCTGCGAGTGCTCGACGTGGTGGAGTGCGACATGGCCGAGGAGCAGGAGGGGGTCGTGGACTGGGTGGCCGCGTTCCCGCCGGAGCCCACCAACCTTGAGTCGCTCTCCTTCGAGTGCTACGAGCCGCCCGTGGACTTCGACGCGCTCGAGGCGCTTGTGGCGCGGTCCCCGCTCCTCAACCGCCTGGGGGTCAACATGCACGTCTCGCTCGGCCAGCTGCGCCGCCTCATGGCGCTCGCGCCGCGCTTGTCGCACCTGGGCACCGGGTCCTTCCGCCCGGCGGATGGCGGCGAGGAGGGGGCAGGATTCGGGGAGGTTTTCTCTGCATTCGTGTCCGCTGGACGAGCGCGCACGCTTGTTTCTCTCTCCGGCTTCCGTGACCTCGCGCAAGAGTACCTGCCGACCATCGCCGTGGTGTGCGCCCACTTGAAGAGCCTTGACTTAAGCTACACCGCGGTCACCCCGAATCAGATTCTCATGTTCATTGGCCAATGCTACAACCTTGAGACACTATGGGTACTTGACTCGGTGCGTGACGAAGGGCTCGAGTCCGTGGGAATGTCTTGCAAGAAGCTCCAATCTCTTCGTGTGCTCCCGTTGAATGCACGTGAGGATGCCGACGAGTTGGTATCAGAGGTTGGCCTTACTGCCATCTCACGGGGCTGCCCTGCTCTCCGTTCGATATTATATTTTTGCCAGACGATGACCAATGCTGCTGTTATCGCCATGTCGCGCAACTGCCCGGAGCTCAAGGTATTCCGGTTATGTATAATGGGACGGCACCAGCCGGACCACGCGACTGGGGAGCCTATGGATGAAGGATTCGGTGCCATTGTTCAGAATTGCAGCAAGCTTACCAGGCTATCCACGTCGGGGCAACTTACTGACCGGGCATTTGAGTACATTGGCAGGTATGGCAAGTCCCTGCGGACGCTCTCTGTAGCGTTTGCTGGAAACAGCGATGTGGCGCTGCAATACATCCTCCAGGGCTGTTCAAAGCTAGAGAAGCTTGAGATAAGGGACTGCCCTTTCGGTGATGCTGGCCTCCTCTCTGGGATGCATCATTTCTATAACATGCGGTTCGTCTGGATGTCAGGCTGCAACCTGACCCTGCAAGGGTGCAAGGAGGTGGCTCAGGGTCTACCACGAATGGTGGTGGAGTTGATAAATGGCCAGCCTGATGAGAAAGAAAGGAATGAAAGTGTGGACATCTTATACATGTATCGTTCACTCGATGGTCCAAGGGAAGATGTGCCACCATTTGTGAAGATCCTGTGA

>SbCOI8 Sb09g003870.1

ATGGGCCGCGGCGGCGGCTCGCGCGCGGCCCCGGCCGCCACGGCGCCGCCGTGGCACGTGCTCCCGGACGAGGTCTGGGAGCACGCCTTCTCCTTCCTCCCCGCGGACTCGGACCGGGGCGCCGCCGCCGCCGCCTGCCGCTCCTGGCTCCGCGCCGAGCGCCGCTCGCGCCGCCGCCTCGCCGTCGCCAACTGCTACGCCGCGTCCCCGCAGGACGCCGTCGACCGCTTCCCGGCCGTACGCGCCGTCGAGGTCAAGGGCAAGCCCCACTTCGCCGACTTTGGGCTCGTCCCGCCAGCCTGGGGCGCCGCCGCCGCGCCATGGGTCGCCGCAGCCGCCGACGGCTGGCCGCTGCTCGAGGAGATTAGCTTCAAGCGCATGGTCGTCACCGACGACTGCCTCGAGATGATCGCCGCCTCCTTCAGAAACTTCCAGGTGCTGCGCCTCGTCTCCTGCGAGGGCTTCAGTACCGCCGGGCTCGCTGCCATTGCCGCCGGTTGCAGAAATCTAAGAGAACTTGACCTTCAGGAGAATGAGATTGAGGATTGCTCTATTCATTGGCTCAGTCTCTTTCCAGAATCCTTCACTTCTTTGGTAACTCTAAATTTTTCATGCCTAGAGGGGGATGTCAATATCACTGTACTTGAAAGACTAGTAACCAGATGTTGCAATCTCAAGACTCTCAAGCTCAACAATGCTATACCTCTTGACAAGCTTGCTAACCTCCTTCGCAAAGCTCCTCAGATTGTAGAACTTGGAACAGGAAGATTCTCTGCGGATTACCATCCGGATCTCTTTTCAAAGCTTGAAGCAGCATTTGCTGGTTGTAAAAGCTTAAGAAGGCTCTCTGGGGCTTGGGATGCCGTTCCAGAATACCTGCCAGCATTTTACTGTGTATGTGAGGGCCTCACATCACTAAATCTGAGCTACGCTACTGTACGAGGCCCTGAACTGATTAAATTCATTAGCAGATGCAAAAATTTGCAGCTCTTATGGGTGATGGACTTGATTGAAGACCATGGTCTAGCTGTTGTGGCATCATCTTGTAATAAACTGCAGGAGTTGCGGGTCTTCCCTTCTGCTCCTTTTGATGCAGCTGAGCAGGTTTCTTTAACTGAAAGAGGTCTTGTTGACGTCTCTGCTAGTTGCCCAATGCTGGAGTCAGTCCTCTACTTCTGCAGACGGATGACCAATGAGGCCCTTATTACCATTGCAAAGAACCGGCCCAACTTTACTTGCTTCCGCCTATGCATCATTGAGCCTCACACTCCAGACTACACTACACACCAGCCTCTTGATGCAGGTTTTAGTGCCATTGTGGAATCATGCAAGGGCCTTAGGCGGCTTTCTGTTTCAGGCCTTCTCACAGATAGTTTATTTAAATCCATCGGGGCTCATGCTGATCGTCTTGAGATGCTCTCGATTGCCTTTGCTGGGAACAGTGATTTGGGCCTGCATTACATCCTATCGGGCTGCAAGAGCTTGAAGAAGCTAGAGATCAGGGACTGCCCATTTGGTGATAAGCCCTTGCTGGCAAATGCTGCCAAGCTGGAGACAATGCGATCCCTTTGGATGTCGACGTGCTCACTGACCCTGGGCGCATGCCGACAGCTTGCACGCAAGATGCCCCGCCTTAGTGTGGAGGTCATGAATGATCCTCGACGGGGATGCCCCTTGGACTCTCTCACAGATGAGAGCCCTGTCGAGACATTGTATGTCTACCGGACAATTGCAGGTCCAAGGTCTGACACACCAGCCTGTGTCCAGATTGTCTAA

>SmCOI1 EFJ04886

ATGGTTGAATCCACCAGGAGGCGGGTGCCTGACGATTCCAGGGGCCTGCTGTGCTGCATCGACGACGTCCTGGAGAAGATCTTTGGCTACATCAAGAAGCCCGTGGAGAGAAATGCTATCTCGGCTGTGTGCAAGCGGTTTCACGAGCTGGAAGCTCGGACGAGGCATCATGTCCTGGTCTACAACATGTACGCTGTCAACCCCATGAAGCTGTTCGAGCGATTTCCCAGCGTGAGATCGATTACCATCAAAGGGAATCCTCGGCTCGTGGATTTCGACATACTCCCCAGGGATTGGGCCGGCCACGCAGGACCTTGGATTGCAGCTATCAAAGCTCATCCCCAGCTTAACCGCTTCCGGATCAAGAGGATGACGATCACCGACTCTCAGATCGAGGAGCTCTGTGCTGCCTGTGGTCCCAACCTCAAGATCATGCAGTTTGACAAGTGCTCCGGCTTTAGCACCCAGGGTCTCCAAGCACTCGCCAAGTTTTGCAAGAATCTTACCCACTTGGGACTTGCACAGTCGATGATTGATAGCACTAGTGATACCAAATGGCTCAAGGACCTGGTAAACTCGTGTCCCGCTCTCGAATACCTGGACTTGTCACTTATAGAGATGGGCGACGTGGATGAAGCGGTGCTGGTGAAGTTGGCCGAGCGTTGCAAGCTGCTTAAGCTGTGGGAGTCCGAGACTCAAAACTCCGAGCGGTTTTTGCCTGTGCTGCAAAAGTGCTCTTCCAACTTAAGTGACCTTGGGATCGAGCGGATCAACAGCAACTCCGAGACGTCGTTACTGGCGAAGTGTACTGCACTGGAGGGGCTGTCCGGCATCTTCGATCTAGTGGACGATGGAATGCACGCTTTTATGTCGGTCAGTTCTCGTTTGACGAGGCTCGACCTTTCTTACTCCAACCTTACCGAGGTGGAGATAGCGGAGGTGCTTCGAGCCTGTCCTAACCTGCAGTATCTTCGGGTGTTAGACTTGGCTGGAGACCACGGGTTGCAAGCGCTGGGGAACTCTTGCAAAGACTTACACAGGCTTGTCGTTGAGAGTCCTTCTGCAATTGACGGTGGGGTGGTGACACACGCCGGCCTTATGGCTGTTGCACAAGGCTGTAGAAACCTGCAGAAGCTCATTTTCTATCCATCTTTCATCACCAACGAAGCGTTTTATGCGCTCGCTTACAACTGCCCGAACTTGATGGACGTGCGAATATGTCTCATCCAGTCAAGTAGCACTGGCGAGAACATGCCATGGGAGTGCCTGGACGAAGGGGTGACCGCACTCGTCCGGGAATGCAGATCGCTCTACCGATTGACGCTCTGCTTCGACGTCCAGGCGGATGTTGAGTTCCTCACCGACGCGGGCGTGGCTGCAATTGGCGAATATGGAAAGAAGATCAGGGTGCTGACCCTTGTCCACTGTGGCAGCAGCGACATGGGATTGGTACCAGTTCTTCGCGGCTGCAACAAACTTCAGAGGCTTGAAATCCGGAAGTGCCGTTTCGGTGATGAAAGCATGCAAGAAATCGCTTTGAACAGCGAGCTGCATCTCAAGCACTTGTTCGTCCAAGGGTGTGAAGTGACGATAGATGGGCTCAGCAGCCTGGCTTACCGGGCCAAGCACACAAACTCGAGGTTCTACGTGGAAGTGATTGGCTGTAAAGACGGGCGGTGCCTGGAGGAGCACAGGTACTCGTGCACGGACGAGTCTTGCGAGAACCACCACGCCTTCTCGCTCAGCTGCTCCCACTGGCAGATCCTGGCATACCATTCACTCACAGAGCCCCGCGACGACACTCCATGGTTTATCCACCGCTTCGACTCGATTCTCTCCGAGGCTTCTTCGCTGCCTTTCACAAGAGCTGAACCAGGAGGCGCTCTTCAGCTCATGCCAGAGAGCAGTAGCGGTGGGATTGATCTTGATCTGAGCACCGGCGGGATCGATCTAAACGAAGACTCGTCGGAGCCGCTCCAGCTGATGCCGGAGAGCAGTGGCAGTGGCGGCGGTGGTGGCAGTGGTGGGATTGATTTGAACGAGCCAGCGGAGGAGAGTTGCTCGTCATTCCCAGGATTTGAGAGGAAGGAAGTCTTCCACTTTCCCGACATTCTGGAGCGCGTAGCGGCAGAGCCAACGTTGCACTTGGGAGCTCCATTGGTGCTCCAGGCCTCCTTACTGTGA

>SmCOI2 EFJ07502

ATGGAGGAGCGAAGCAATAACAAGAAGAAGAAGAAGAGCACAGTGCTGTGGCTGTGCGATGAGCTGCTGGAGAGAATCCTCGCGCTTATCGCCGATCCCTGCGACCGCGCGGCGGTCTCCGAGGTGAATCGGCAGTGGTACCGGGTGGAGGCGAGGACGCGATCGAGATTGGTGGTCAAGTGCTCGTACGCAGTGCATCCATGGCGGCTGGCCCAGCGATTTACCGGCCTCGCCAGCGTGACCATCAAGGGGCGGCCACGGATCTACGACTGGGGCTTGCTCGGGGACGACTGGGGCGGCGCCGCCGACACCTGGATCCGCGTCCTCGTCGCCTGCTGCCCCTCTCTCGCTGCCATCCACCTCCGCCGCTTCGACGTCCCCGACTCCGCCATCGCCGCCATCGCCACCGCCGCGTTTGCGAGCTCGCTGCAGGTTCTCAAGCTCGACCGCTGCTCGGGCTTCTCCACCCGGGGCCTCCTCGAGATCGCGCGCCACTGCAAGAACCTCAGAGTGTTGAGCTTGGACGAGAGCATCGTGGATGGTGGCGGCGAGCAGTGGCTGCGAGCTCTGGCCGACACCGCCACCAAGCTCGAGGTGCTCAGCTTTAGCCTGACGGGGATCGAGGTTCGGGGCCTGGACGATGTGGCGGCGATTGTGAGCAGGAACAAGCGACTCGCCTCTCTTCGGCTTGACGAAGTTCGCACTACCAATGACGCCATTTCCAGGGCCCGGGGGATCCTGCGCGATGCCGCATCGCTCCAGGAGATGCTGCTGCTCTATCGCTCCGTGGATGAGAGCAGCATCATCGAGAAGCTGGAGCTGCCCAAGACGGTCACTTCGCTCGCCGGGGATATCAGCATTCCTCTCGATTGCGGGCTCGCCTCCCGGCTGCTCAAGCTCGACTTGATGCTCACCACCCTCGATAGCTCGCAGCTCTCGCTTCTCCACCAGACTTTCCAAGCGTGTCCCAACCTGGAAGAACTCAAGGTGAGGAACAGCATCGGGGACGAGGGAGTCGAGGCCATCGCTAAGCACTGTCGGAAGCTCAAGCGCATCCGAATCGAGAACCTCGAGGACAACCATCACTCAGTCTCGCAGCGCGGCCTCATCACGCTCGCCTCCAGCTGCCCACACCTCCGCACAGTAGCCATCTACGCCTCAGACGTGAGCAACGCAGCATTTGCCGCGTTTGGGCATTGCTGCCGGGACCTCTACGACTTCCGGATCGCGGTGCTCGACTCCCCGACCCCGCTCACCGATACCCCGCTCGACGCCGGCGTCAAATCCCTCCTCCAGGGCTGCCGGGGCCTCCGCAAGCTCGCGCTCTACCTCAAGCGCGGCGGCCTCTCGGATCACGGCCTGGCAGAGATGGGGGTGCTCGCGGGCAATCTCAAGTGGCTGCTCCTGGGCTGCGCGGGCTACTCGGACGCCGGATTCGTGGGCCTCGCGGCCGGGTGCGCGAGGCTGACCAAGCTGGAGCTCCGGCACTGCCCGTTTAGCGAGGCCGGCATGGCGGCCGGGGTGGCGCGGATGGAGCGGCTGAGGTATGTGTGGAGCCAGGGGTACCGCGAAGTGGACGCCAGAGAGTTACTGGCGCTGGGGCCGGCGTGGAACATCGAGTACATGCCGTCCCGCGATGCCGCTGTGACACAGTTCGTAGCATATCGGTCGCTGCTTGGGCCCCGGATGGATTGCCCGCCTCGCGTCATGCAACTGGTTGGCTAA

>SmCOI3 EFJ09817

ATGAAGGGCCACGATCTTCTCAACGATGTGCTGCCGGACGAGGCGCTCATTCACATCCTGAGCTATCTGGACGTTCCCAGCGACCGGGGCTCGTGCTCGCTGGTTTGCAAGCGATGGTGGCAGCTGGAGAGCGAGACCCGGCACTCGATCCGGATTGGCGCGTCCGGGAACCCGGACGCTTGCGTCACGGCGGTGGTGAGACGATTCACGGGGCTGCGAGACGTGAGCTTCGATGAGAGGTTTGGTTTTTCCTTGATCCAGAATGGCGACGCGACCAGCCGGAGGGGACGAAAGCGTCGCAGAGGTACTGACGAGCTATCACCACTCTTGACGGAGAGTCTGTGGAGCAGCCTCTCGGACAGCGGGTTGATGCTGCTGGGACAAGGATGTCCGAGGCTGGAGAAGCTGACGCTCGTCTGGTGCTCGGCCATTAGCAGCACCGGATTCAAGTCCCTGGCCGAGAATTGCTGCGGCTTGAAAAATCTAGAGCTCCAGGGATGTTACGTAGGAGACGACGGGCTCAAGGCCATCGGTCAGTTTTGCAAGCTCGAGGATCTCAACCTTCGCTTCTGCGATGGAGTAACGGACTTGGGACTTATGGCGATCGCCACGGGCTGCGCAAAGTCACTCAAGGCGCTCATCATATCCGTGTGCCCCCGGGTAACGGACGCGACTCTGGCAGCCGTGGGAAAGAACTGCTCGCTGCTGGAGAGGCTCACGCTCGACTCGGAGGGATTCAAGAGTGACGGCGTCCAGGCCGTGGCCAGGGGCTGCCCGAGGCTCAAGTACCTGAGGATGCTGTGCGTGAACGTCGAGGACGAAGCCTTGGACTCCGTGGGACGCTACTGCCGGTCGCTGGAAACGCTAGCGTTGCACAGCTTCCAAAAGTTTGACAAAGGCTTCTTGGCGATCGGGCATGGCTGCAAGCAGCTCACAAGCCTCACGCTGAGTGACTGTTACTTCCTCACGGACACGACGCTGGCGGCGATTGCCAGCGGCTGTACTGAGCTCTCGTCGCTGGAGATAAATGGCTGCCACAACATTTCCACTTCTGGAGTCCGAGCAGTGGGACGCTCCTGCCGAAAGCTCACGGAGGTAGTGCTCAAGTACTGCCAAAAGATTGGAGACGATGGCTTGTCCGAGATTGGCAGAGGCTGCAAGCTTCTACAGGCGCTCATTCTCGTCGACTGCTCGGCCATTGGAGACTCCTCCATTCGTAGCATCGCTGGAGGCTGTCCCGGTCTAAAGAGGCTCCATATAAGACGCTGCTACAAGATTGGAGACAAAGCCATCGTCGCCGTTGGCCAGCACTGCGAGAGACTGACCGACTTGAGTATGCGATTCTGTGACCGAGTTGGAGACGATGGCCTGGCAGCAATTGGAGCAGGCTGCCCGGAGCTCAAGCATCTAAACGTAAGTGGTTGCCACCGCGTTGGAGACGCTGGCATTTCTGCGATTGCGAAAGGATGCCCAGAGCTGATACATTTGGACGTGAGCGTTTGCCAGAGCGTAGGCGACGAGGGACTCGCAGCGCTGGCCGGTGGATGCCGGAGCCTCAGAGAAATCATACTGTCACATTGTAGAAGTATCACGGACGCCGGACTGGGTTTCCTTGTGGCGTCTTGTACCAAACTCGAAGCTTGTCACATGGTTTATTGTCCATACGTAACTGCTGCAGGAGTCGCGACCGTGGTGACCGGTTGCTTGAGCATCAAAAAGGTGCTGGTCGAGAAGTGGAAAGTTACCCCCAGGACGAGGCGCCGGGCCGCGTCAATTTTAACAGAGCTCTGTATGGATCTATAG

>SmCOI4 EFJ14966

ATGGGCGAACGTAGCTGCTGCAGCAGGACCAGGCAGAGGAACAGCAGGGATGGCGATGCGAGCATCATCGATCAAGTGTGCGAGCATCCGTTGCTGCTGGAGAGCGTGCTTAGCATCATCTTTGGAATGGTGGACAGCCCCGCGGAGAGGCGGGCGATGTCCGAGGTGTGCCGCCAGTGGCACGCCATGGACAGGGAGACGCGCAAGCACGTTTATGTGGCCTTCGTCTACTCGGTGTCGCCCGCTACGCTCACTCGCCGCTTCCCCAACCTCCGGAGCCTCAAGCTCAAGGCCAAGCCGAGGGCCTACGAGTTTGATCTCCTCCCCCACAACTGGGGGGGTCATGTCCACCCCTGGCTTGAAAACATTGGGCCCGCCTACCCGCAGCTCTCTGCGCTCCATCTCCGCCGCATGGAAGTCCGCGACCAGGACCTCTCCGCCGTCGCCACCGCCTACGCCGCCAGCCTCGAGACGCTCAAGCTCGACTTTTGCTCCGGCTTCTCCACCACCGGCCTGCGAGCCATCACTGGATCCTGCAAGTGCCTCAAGGTGCTCTACGTGGAGAACAGCTACGTCTCGGACGAGGGCGGGCAGTGGCTCAACGAGCTGGCGCTCCACAACCGGGTGCTCGAGGTGCTCGATTTCCAGCTCGCCATTGGCATCTCCAAGGTGAACGTGGAGGACGTGAGAACCATCATCGAGAAGTGCCCAAACCTGACGTCCCTCAAGCTGGTGGAGGGCGAGGACGGGCTGGGCGATGGGCTGAGGAAGGCCCTGGCGTCGTCCACATCGCTCCGGGAGCTGGGCATCTTCCTCACCGCGCAAGAGGAAGACGATCAGGAAGAGATCGATCAGGGCACCAGCAGCACAGGCCAGCAAACAATGAGAGCGCTGCTACCCAGAAATCTCACCAGCATCTCGGGGGATATCCCTCTCCCGCTCTACACCTCCGTGGCGGCGCAGCTCCTCAAGCTGGATCTCATGACCACTACCTCCATCGAGGCGGAACAGCACCACGCCCTCCTCCGCTGCTGTACCAGGCTCCAAAACCTTCAGGTGCGGACAGTCATCGGCGATGAAGGCCTGGCCATTGTGGGCGAGTGCTGCAAGGATCTTCGCAAGGCCCGGATCGAGGACCACAACGACGAGGGGACCTCGGTGTCACACACGGGTCTCATGGCCCTGGCTCGCGGCTGCTCCAAGCTCGAGAAGCTGGCCATCTACGTCGCGGACATGTCCAACCAGGCGCTGGCCGCGGTGGGCAGCGGCTGCCCCGACCTCCGCGACTTCCGCCTCATCCTCACCGAGGCCAACGATCTGAGCAGCATGACCGAGCTGCCGCTGGACGCCGGCTTCGACGAGCTCATGCGCGGCTGCCACCGGCTATCGCGCCTGTGCATCTACGTCCGCCCCGGCGCGCTGTCCGACCACGGCCTCGTCCGGATCGGCCACCGCGGCGCAAACCTCAAGGCCTTGCTCCTCGGCTGCTGTGGCGACAGCGATGCCGGCTTCCTGGCCATCGCGCGGGGCTGCAAGCGCCTCCAGCGGCTGGAGATCCGCGACTGCCCCTTCACCGACGATGGGCTGCTGCGCGGCGTGGGATGCATGGAGGACCTCAAGCTCATGTGGATCCAGGGATTCAGGATGGACGACTACGGGAAATTGGACGTGCTGGGCGGGGAGAAGTACCGCAATGTCGAGTGCACGCGCCGGGATCCAATCCAGTGCTTGATCTACCGCTCGCTCGCCGGGCCGAGACTAGATTGCCCCGAGGAGGTGGTGTCGCCGCAGGGAAATGGGATGCTCCGCCACAACTGGCTCACTCCAAGCGGTGGGCTCGTCGTCGGCTAA

>SmCOI5 EFJ15060

ATGGCGGATCGGAAGATGAGCAAGAACAGCGGCGACAGTGTCATCGATCTCCTGGACGACAATATTCTGCTCCAGATCCTGGAGCGCCTTGAGGATCGATTCGACCGCCAGGCGTGGTGCCTCAGCTGCAAGCACTTCCTGCGCCTCGAGGCCAGCACCAGGAACCGGATCCAGCTCATGCGCCACGAAGTTCTCGAAGGGATCCTCCACAGGTACACGCGGCTGGAGCATCTGGATCTGTCGCATTGCATCCAGCTGGTGGACGAGAATCTGGCGCTCGTAGGGCAGATCGCGGGAAATCGTCTCGCCTCGATCAACCTGTCGCGAGTTGGGGGTTTTACCAGCGCTGGGCTGGGGCTGCTGGCGCGATCCTGCTGCGCCTCCCTCACCGACGTGGATCTGAGCTACTGCTCCAATCTCAAGGACTCGGACGTGCTTGCGCTGGCGCAAATCTCCAATCTCCAGGCTCTCCGCCTCACGGGCTGCCACTCCATCACAGACATTGGGTTGGGCTGCCTGGCCGCTGGCTGTAAGATGCTCAAGCTTCTCACGCTCAAGGGGTGCTTGGGGATCACGGACATTGGCATTGCTCTCGTTGCCGTGAATTGCAAGCAGCTAAGAACACTGGATCTCTCGTACACCGAGGTGACCGACGAAGGCCTGGCATCCATAGCGACTTTGCACTCGCTGGAGGTTCTGAATCTGGTTTCTTGCAACAATGTTGACGATGGAGGATTGAGATCGTTGAAAAGGAGCTGCAGATCGTTGCTGAAGCTGGATGTTTCTCGATGCTCCAATGTCAGTGACGCAGGTCTCGCAGCTCTGGCCACCAGTCACCTCTCCTTGGAACAGCTTACGCTTTCCTACTGCTCTATCATCACGGACGATCTACTGGCCACCTTTCAAAAGTTTGATCACCTGCAATCAATTGTCTTGGACGGGTGTGAGATTGCTCGCAACGGTCTCCCTTTCATCGCCAGGGGTTGTAAGCAGCTCAAGGAGCTCAGCTTAAGCAAGTGTAGAGGTGTAACGGACAGAGGCATAGCTGCTGTTGCTCAAGGATGCACTGCTCTTCACAAGCTCAACCTGACGTGCTGCCGGGAGCTGACGGACGCGTCTCTGTGCCGGATCTCCAAGGACTGCAAGGGTCTCGAGAGCTTGAAAATGGAGTCGTGCAGCCTCATCACCGAGGATGGGCTGTGTGGTCTCGGCGAAGGCTGTCCTCGTCTCGAAGAACTCGATTTCACCGAGTGCAACATGAGTGATACCGGCCTCAAGTATATTTCCAAGTGCACGGCACTGAGGTCCCTCAAGCTGGGATTTTGCTCCACCATCACGGACAAAGGCGTTGCTCACATTGGTGCCAGGTGTTGTAACCTTCGAGAGCTTGACTTCTACAGGTCCAAAGGTATAGGTGACGCTGGAGTGGCAGCAATTGCTTCCGGGTGTCCAAAACTCAAGCTTTTGGACCTTTCGTATTGTAGCAAGATCACAGACTGCTCGTTGCAATCGCTGTCGCAGCTGAGAGAGCTGCAACGCGTCGAGCTCCGCGGCTGCGTGCTTGTTTCGTCGACAGGCCTGGCCGTGATGGCATCGGGCTGCAAGAGGCTCACGGAGATCGATATAAAGCGCTGCTCCCAGATCGGGAACGCTGGAGTCTCGGCCTTGTCCTTCTTTTGCCCCGGCCTCCGAATGATGAACATCTCATATTGCCCGATCTCAAACGCGGGATTGCTGAGCCTTCCCCGGCTCAGCTGCTTGCAAAGCGTCCGGCTTGTCCACCTCAAGAATGTCACAGTGGATTGCTTCGTGACAGTGCTCCAGAATTGCAAGAGCTTGAAGAACGTCAAGCTTCCATCGTATTTGAGAACACTCCTCCCTCCCGGGATCGCGGAAGAGATGGAATCGCGGGGCTGCCGAATTCGATGGATGGACAAAGCCTTGGAAGAAGACGATGTCCTCGAGCCTCACTGA

>SmCOI6 EFJ20341

CTTCCCGAGGAATGCCTAGGGCTCATCTTCGATCGCCTCGACACGCGCGGCCGCAACGTCGCGTCGCTCGTCTGCCGCCGCTGGCTGGTCGCCGAGGCGAATTCTCGCAAGATCCTCTCGCTATCGGCGCCACTGTCGCTGCCGGTGTCGTGCCTGGAATCCAGCCTTATGCGATTTACAGTGCTCTCCAAGCTGGGTCTCAAGTGTGAGCGAGGCGTCCCTTCCATCACCGACGAGGGGCTGGTGCTCATCGCCACGCACTGCCGCCGCCTCAGCAAGCTCAAGCTCAAGAACTGTACAGGGCTCCAAGACGATGGGCTCGTCGCCTTCGCCGCCGCGGTGTGCCGGGCATCCTTTCGATCCTTCTCGTGCTGCTCGTGTGGATTCGGCTCGCGGGGGCTCAACGCGATCATCAAGAACTGCGTGGCTCTCGAGGATCTCTCCGTGAAGAGGCTCCGGATGGGTGGCGAGCCGGGGCAGCTGGTCGAGGGGCCGAGCAAGCTCAAGCGGCTGTCCATCAAGAACATCCTGGATGGCGGCCATGCTTTCACGCCGCTCATCGCGAGCTCCAAGCATCTTCACACGCTGATCATCTTCAAGGCTACCGGTCAGTGGGACAAGCTCTTGGAGCTGTCCGTGGAGGGGCTGAGCGAGCTGACGGAGCTGAGGATCGAGAAGCTCCATCTCGGCGATCAAGGGCTGGTAGCTCTCGCAAAATGCCGCAAGCTCCAGGTGCTCTTCCTCGCCAGAACTCCTGAGTGTAGCAACACGGGGTTGTCAGCCATAGCCAACGGATGCCGGTCGCTTCGCAAGCTTCACGTTGATGGTTGCTTTACGGGGAGAATCGGGGACAAGGGGCTGCTTACCGTCGGCGAGAGGTGTCCGGAGCTCAAGGAGCTCGTTCTCATCGGCGTGTCCGTCACCTCCAACAGTCTGGGAACTGTTTTTACGAACTGCATGGGGCTGGAGCGTCTCGCAGTGTGGAACAGCGAGACTTTTGGGGACGGGGAGCTGGCTTGCATCGGAAGCAAGTGCCAGGCTCTACGCAAGCTGTGTATCAAGTGCTGTCCCATCTCGGACCAAGGACTGGAGGCGCTGGCCAGCGGGTGTCCGAGCTTGACCAAGGTGAAGATCAAGAGGTGTAGGAGTGTATCGGCTTCTGGAGCTGCGTCGCTGATGATGGCTCACGACGGACTGGTGGTGACTTTGGAGGCTGATCAG

>SmCOI7 EFJ24298

ATGTCCATGGAGCTGGAAGATGTTCGTTTCAACCATTTGTTCCAGAAGTCTTTCCTTCACGAGAACTCGTATGCGTTCTTGAGCAAGGAGGAGCTTTGCTCGCAGCTGATCCGGGGCCTTCAGAGCCTCCAGACCGGGCGGAGGAGGAAACTGAGCGAGATTGGCAACACTGAAATTTACAATGCTCCCAAGAGGTTCCTTCCAACTCCACCACCAACAGCGGTCCCGGCAGCAAAGAAGTACCAGCCCAAGGATCTCATTAATGCGCTTCCGGACGAGCTCCTTGTCGAAGTCTTTCGGTACGTGGCCGCTCCAGCCGATCGCTACGCGTGTGCTTCCGTGTGCACACGCTGGCTGATGCTCCAGAGCCACTTACACTCGAGCGAGATCAAGGACGACGAGCAAGAGCTTTCTCTTGGCAGTGGCGACGAGCTAAAGAGGAGCCTGGAAGGAAAGCGAGCTACAGATGTTAGACTGGCTGTGGTCGCTCTCGGAACCCAGTCTAGAGGTGGCCTTGGGAAACTTATCATCAAGGGTGGTCCTCGGCAGAAGCTGTCCAAGGCCGTGAGCAACGTTGGCATGTCCTCTGTTGGCATCTGCTGCGGGAACCTCAAGGTTCTTTCCGTGTGGGACTGCCCAAACATCGACGACGTGGGATTCAGCTGGATTGGCAAGGGATGCCCGCAGCTCAAAGTTCTCAACATCATGAACTGTCCTGGCTTTGGAGATGCTGCTCTTCGGGCAATTGCGGCCGGTTGTCCTCTCCTCTCAAGTTTGACCCTGGATGGCTGCGACAAAGTTGGCGACGAAGGACTGCAGGCCGTGGGGAAGCGCTGCTCTCAGCTTTCGTGCCTCTCGGTCTCCAGGTGCAACAAAGTCGGGGACGTTGGCGTCACTGCGGTTGTTTCGAGCTGCAAGGTGTTGAAGGCCATGAAGCTCGAGAAGCTCAGTATCAACGACGAAGGTCTCGTTGCGGTTGGGGAACACGGCGGCTCACTCCAGAAGCTCAAGCTGTTGCAGCTCGAGAAGATAAGCTCGGAAGGCTTCTTTTTGTTTGGCAAGAGTTCCGGCATGGGGCAGCTCAAGCATCTCCAGATCTCGGCATGCCCCGGCCTCACTGACAGTCTCCTAGACTCCGTTGGGAAAACGAGCAAGGAGATCAAGTTTTTGTCTCTTGCCAACTGCACGTCGCTGGACGAGAGCAAGCTTCTCACTTTCGTGAAGGACTGCACTTTCCTGGAGGGCCTCCACCTAGAGAAGTGCGCCTTTACGGCATCGGCTGCGACGATGACGACGACACTGTTATCGAGCGGGTCCCGGTCTCTTAAAGTTCTGGGCATCGTCAACTGCACTGGAGTGGGAGCCGGGCTTTTGGCTAGCCTCTCTGGATCTGGAAGCTCCTGTCTCCTGGAGCTCAACGTCAGCGGCCTTTCGGCTCTCTCGGACGAGTCCCTGGTCCCTTTCCTGTCAGCGAGCGGGTCCGGCTTAACTTCTCTCAACCTGTCCGGTTGTACGAGGCTGACGAACCGGGCACTCGCTGCGGTAGCGAGTTTTTGCCCCTCTCTCGGGTTGCTCACGCTGGACGGGTGTGCGAGCGTCACGGACCAGGGGATTCGATACGTTGCTCAGGGGCCGCAGGCAGTGCAGGAGCTCAGCCTCGCCGGCTGCGACGTGACGGATGACGGCATGGTGGCTCTAGTACTCGCCAAAGGCTCGTCTCTGAAGACGCTGTCTCTGGCCGGGTGCGGGCGCGTGACGGACCGGAGCCTGCTGGCTATGAAGACGGCTTGCAATACTCTGGAGGCGCTCAACGTCAAGGACTGCAAGGGGCTTAGCCGGGCCAAGCTCGAGTGGTTTGAGGCGGGCCTGTGGAGGTGCCACCTTTGCTACTGA

>SmCOI8 EFJ29076

ATGGAGCTCGAACAAGAGAAAGAGGAGGACGAAAGAGAGGAGGAGGAAGAACAAGGGAGGCTTACAACTTTCCCAGACGAAGTCTTGGAGAACGTCTTAAAGTTCGTCACGGGACACAAGGATCGAAACGCCGTGTCCGTGGTTTGCAAGGCGTGGTACAAGGCCGAGGGGTGGAACCGAGAAGCAGTGTTCATCGGGAATTGCTACGCGGTGTCTCCAGACATTCTCACCCGGAGGTTTCCACGGCTCAAGTCGATGACTCTCAAGGGGAAGCCTCGCTTCGCCGACTTTAGCTTGGTTCCTCCAAACTGGGGGGCCTTCTTCCACCCCTGGATGCCCGTCATCGTGGAGAGCTACCCGTGGCTGGAAGCGCTCAGGCTCAAGCGCATGACCGTGTCGGACGAGAGCCTCTTCATGATCTCGCAGTTGCTGCCAAACTTTCGAGCTCTCAACCTCGTCAACTGCGATGGTTTTAGCACCGAGGGCATTGCAGCCATCACCAGCCACTGCAGGTACTTGCAAGAGCTAGACCTTCAGGAGTGCCTGGTAGACGACCGCGGTGGAGAGTGGCTAAGCTACTTCCCGGAGAGCTGCAACACGCTCGTCACTCTAAACTTCTCGTGCCTGGAGAGCGATGTCAACTTTGAGTGCCTGGAGAAGCTCGTCTCGCGCTGCCGCTCCCTCAAGAAGCTCAATCTCAACAAAGGCGTCACTCTGGAGCAGCTCCTGCGCCTCCTCGTCAAGGCCCCGCAGCTCACCGATCTCGGCACCGGGACTTACTCGCAGATGCAAAACTGGTCGCAGTACGTGGAGCTCCGCACCGCGCTCTCAAACTGCAAGGATCTCCGCCACCTCTCGGGCTTCTGGATGGTGGAGCCGATCTTCATCCCGCTGATCTATCCCCTGGCGCAGAACCTCCTGTCGCTCAACTTGAGCTACGCCACCATCCGGGCCACCGAGTTTGCCAAGCTCATCCAGCGCTGTCCCAAGCTCGAGACTCTCTGGGTTCTGGATTCCGTCGAGGACCGGGGCTTGCAAACCGTGGGCGAGACCTGCAAGAACCTGGTGGAACTCCGCGTCTTCCCGACCGATCACGGTGGCCAGGGCTCGGTGACCGAGGCTGGCCTGGTGGCCGTCTCGCAGGGCTGTCCAAACCTCTCCTCCGTGCTCTACTTCTGCAAGCAGTGCACAAACCAGGCCATCGAGACCGTCGCCACCAATTGCCCGATGCTCACTCGCTTCCGCCTGTGCATCATCACCCCCCGGCAGCGGGACTACATCACCGGCGAGACCATGGACGAAGGCTTCGGCGCCATCGTCAAGAACTGCAAGAACTTGTCCCGCCTCGCCGTCTCGGGCTGGCTCTCGGACAGGGCCTTCGAGTACATCGGCCACTACGCCAAGAAGCTGGAGACGCTCTCGGTGGCCTTCGCCGGGGAGTCGGACGCGGCCATGCAGCACGTACTCAGCGGCTGCCCGAGGCTCCGGAAGCTGGAGATCCGCGACAGCCCGTTCGGAGACTCGGCGCTGCTCGCCGGGCTTCACCAGTACGAGTCCATGAGGTTCTTGTGGATGTCGGCTTGCAGGGTGAGCCTCGCCGGCTGCGGCTGGCTGGCGGGGGCGATGCCCAGGCTCAACGTCGAGGTGATCCGCGAGCAAGGTGACGCTGAAGGTGGCGAAGGTGGCGAGAAAGGTGGCGGAGAGCTTGTCATGGATTGCAGCGAGCCGGTTGAGAAGTTGTACGCGTACAGGACGCTGGCTGGGTGCCGAAGTGATGCGCCCAGCTGGGTGATCACTCTCTAACTCCGCCCAGGTTCTAAAAACTCTCTTCCTTTTAATAAGATGAAATCAAGTTTTAGAC

>SmCOI9 EFJ30612

ATGAAGATCTCCAGGCGGTGGATTCACGCCGATGATGATCCAGACAGCATCAACAGCCGCCTTCCAGACGATCTCCTCAAGATCATCTTCTCGCGCCTCGGCGACGATCAAGACCACGCCAGCGTCGCGCGGGTGTGCCGGCAGTGGCGCGATGCCGAGAGCGCCACCAGGGAGAAGATTACTGTGAATTTCTCCTACGCCGTGAGCCCGGGCTACGTGATCGATCGATTCGGGCAGCTGCGCGCGCTCAAGATCAAAGGCAAGCCTAGGGCTAGCGACTTCGGGCTCATTCCAGTCGACTGGGGAGGCTATGGCGGCCCCTGGATCGCCGCGTTGGCGCTGGCGAGGGCGAGATCGCTGTTTGGCGCTCTCGCCTCGCTCCATTTCAAGCGCATGGAGATTTCCGACGAGGATCTGGCGCTCCTGGCCGAGACGTTCCGCGACGCGCTACAGGTTCTCAAGCTCGAGAAATGCTCCGGATTCACATCGCTGGGCCTGGAATCCATCGCTCGATCGTGCAGGGATTTGAGGGTGCTCTCGCTGGACGAGAGCGATATCGAGGACAAAGGATCACAGTGGCTGCGTGAACTTATCCATTCGTGCGCTAGCCTCGAGGCCTTAAACCTGTCCATGACAGGCTTGGAACTTAGAGATATCCGGCTGGTCGAGGAGATCGTGAGCAGCTCCAAGCTCAAGAGCTTGAAACTCAACGATCTGGAGGACCCTTCTCGCAATCGCAGGCTGGATTTGCGACAAAGCTCCCTCCAGGAGCTCGGTTTCTGCGGCCTCATTCAAGTCTCTTTACCATCGTCACTGAGCTCTTTCTCTGGTGATCTCCAGCTGGCAATGGAGCCGAATCTCGCGAGCGCGCTCACCAGTCTGGATCTCTTGTACACCACTGCCAATCACGAGCAACACCTGGAGATCATCAAGGGCTGTCGCAATCTCCAAGTCTTTAAGGCAAATATAATCGGCGACATCGGGCTGGAGCTCCTCGCAAGCCACTGCAAAGGCCTGCAACGCATCCGGATCGAGAACATGAGGCAGCAAGAACAGCACGGCTTCTCGATCTCTAACTCCGGCATGCTGGCGCTGGCCAAGAGCTGCGTCCATCTCCAGAGCTTCTCCATGTACGTCCACGACGCCGCCAACTCGTCCCTGGAGGCCTTAGCGGAGTCGTGCCCGGGCCTGCTCGACTTCCGGCTGGGGATCCTCGAGACCGCGCCGGACATGGCCGAGCCACTGGACGCCGGCGTCCAGTCCCTGCTCCAGCGCTGCCCGAGCATCACCAAGTTGGCACTCTACCTCAAGGAAGGCGGACTCACGGACAGGGGCCTGGAGAGCATCGGGCGACTCGGGCAGCAGCTCAAGTGGATCCTTCTCGGCTGCCTGAGCGACTCGGACACCTCGGACCGCGGGCTCGTCAGTCTGGCAAGAGGGTGCTCGAATCTCCGCAAGCTGGAGGTGAGGAACTGCCCGTTTAGCGACGCCGCGATCGTGTGTGGCATCCGGGGCTTACCGCTGCTGCGCTACCTCTGGTTCCAGTGCTACCATCGCGTTAGCGATCGCCACTTTGCGCTGCTGGAGCCGGAGTGGAGGATCGAGCTCATGCCCGAGTTCTACTCGGTGCTTTGCTACCGGGCGCTGGTATCGGGTAGCCGAGGTGACCACCCGCCGAGTGTCCGGCCAATGCTCGGTGGTGGTGCTGGTGGTGGCTAG

>SmCOI10 EFJ31715

ATGAGACTCGAGGCCGACATCTGGAGCTTGAACAACCATCTCCTCGTCAAGATCCTGGAGAAGCTGGACGAGGTGGTGGACAGGAAATCGTGGCGTCTCACTTGCAAGCGCTTCTACGCGGCCGGGGCCGAGTCCCAGAAAACCATGCGCCTCTTCAACTCGGAGCTCCTCCCGAGAGCTCTCGCGCGCCACACCGGCATCGAGAGCCTCGACTTGTCCTCGTGCATCAAGATCACGGACGAGGATCTCGCCCTTGTCGGGGAGCTCGCTGGGACGCGCCTACGATCGCTCGGCCTGGCTCGCATGGGAGGATTCACGGTCGCCGGGATCGTTGCTCTGGCGAGGAACTGCTCCGCGCTGGTGGAGCTCGACTTGCGGTGCTGTAACAGCCTTGGAGACTTGGAGCTGGCGGCCGTGTGTCAGCTGGGAAGCTTGCGAAAGCTCGATCTCACGGGATGTTACATGATCTCGGACGCTGGATTGGGATGCCTGGCAGCCGGTTGCAAGAAGCTCCAAGTCGTCGTGCTCAAAGGCTGTGTTGGTATTTCGGATGCCGGGCTTTGCTTCCTTGCTTCCAACTGTAAAGAGCTCACCACCATCGACGTCTCCTACACAGAGATCACGGATGACGGCGTCAGGTGTCTATCAAACTTGCCGTCTCTGAGAGTGTTGAATCTGGCTGCGTGCTCCAACGTTGGAGACGCGGGATTGACCCGTACAAGTACATCATTGCTGGAACTGGATCTCTCTTGCTGCAGAAGCGTGACCAATGTGGGAATCTCGTTTCTCTCGAAGCGAAGCTTGCAGTTTCTAAAGCTCGGGTTTTGCTCGCCTGTAAAAAAACGATCGCAGATAACTGGCCAGCTCTTGGAAGCAGTTGGAAAGCTCACACAGATCCAAACACTTAAGCTAGCTGGCTGCGAGATCGCCGGCGATGGGCTTCGCTTCGTGGGCTCTTGCTGTCTTCAGCTAAGCGACTTAAGTTTGAGCAAGTGTAGAGGGGTCACGGATTCTGGCATGGCTTCCATTTTCCATGGCTGCAAAAACCTCCGGAAGCTTGATCTTACTTGCTGCCTCGACCTCACGGAGATTACTGCTTACAACATCGCAAGGTCGAGCGCGGGACTTGTCAGCTTAAAGATCGAAGCTTGCCGCATTCTAACGGAGAACAACATTCCACTGTTGATGGAGCGGTGTAGCTGCTTGGAAGAACTGGACGTGACCGATTGCAACATCGATGACGCAGGACTGGAATGTATTGCGAAATGTAAGTTTTTGAAGACTCTCAAGCTCGGGTTTTGCAAGGTCAGTGACAATGGCATCGAGCATGTTGGAAGGAATTGCTCCGACTTGATCGAGCTCGATTTATACAGGTCGGGGAATGTTGGTGATGCGGGAGTTGCGTCCATAGCCGCTGGCTGCCGCAAGTTGAGAATTCTAAACCTCTCGTACTGTCCAAACATCACGGACGCTTCCATCGTCTCCATTTCGCAGCTCAGCCATCTCCAGCAGCTCGAGATCCGGGGTTGCAAACGAGTTGGCCTGGAGAAAAAACTCCCGGAATTCAAGAACCTTGTAGAGCTCGATCTCAAACACTGCGGGATTGGCGATCGGGGAATGACCTCCATCGTCTACTGCTTTCCAAATCTCCAGCAGCTAAATCTATCGTATTGCCGGATCTCCAACGCTGGACTCGTGATGCTGGGAAATCTTCGCTGCTTGCAAAACGTGAAGCTCGTCCAGATCGGGGATGTTTCCATCGAAGTTCTCGCGGCAGCACTCCTTTCGTGTGTTTGCCTCAAGAAAGCAAAGCTATTCTGCAATGCCTTGCTGAACGATTCCATCAACGCTCGCTATCAGCAACTGGAAGATCGTGGCTGCCGAATTAGATGGATGATCAAACCAGAGAGATAG

>SmCOI11 EFJ32115

ATGGAATGGATGTCCGACGAGCTCCTCGACTCCATCTTCTCGTTCATCGATCACCCCATGGACCGGCGAGCGCTCTCCGAGGTTTGCAAGCGCTGGTACCTTGCAGACGCCAGGACTCGCAAGAGCATCACTGTTGGCTTCTCCTACGCCATCGAGCCATCCAACTTGAGCCGAAGGTTCCGCAACATCCAGGCTCTCAAAATCAAAGGCAAGCCTCGAGTGTCCGAGTTTGGGATGGTTGTCAAGGACTGGGGTGCCTACTGCGAGCCGTGGATCCAAGAGCTCGTCAGCCAAAGGCATCCCAGCAGCGCTACAGCATTCGCCTCTCTAACGTCTCTCCACTTTCGACGCATGGAAGTCTCGGACACGGCCTTGAGATTGCTCGCTCGGGGCTTCGGCTCCTCGCTCCAAGTTCTCAGGCTGGACAAGTGCTCCGGGTTCTCCACCGCGGGATTGGAAGCCGTGGCTCGAGAGTGCAAATCCCTGCGAGTTCTTTATCTGGAAGAGAGCGTGATCGAGGACGATGGAAGCCAGTGGCTCCACGAGCTGGCAGTGAGCAACAGCGCCCTGGAAGTCCTCAACTTCTTCCTCACTGGCTTGGATCTTTCGAATCTCTCCGACCTGGCGCACATTATCGCGAACTGTAAGTCGCTGACATCCCTGAAACTTGGCGAGATCAGTAGGGGCGTGGTGGACCTCCCGGCGGACATCTTCATCGCGGCCAAGTCGCTCAAGGAACTGGCCGTGATCTTCGCTCGCAACAACATCAGTGTCAACTTGCCCAAGACCCTCACTTCTTTCGCCGGTGACCTCTTGTTCCCGCTGGATCCACTCGTGTGCTCAAATTTTCGCGAGCTGGATCTCATGTCCACCACTCTCACCGCCGAGGAGCATATGCAAGTGATCCAGTGCTGTCCAAATCTCGAAGTCTTGAAGGTCCGGAACATAATCGGCGATGCCGGCGTCGCCACGCTCGCCAGCCTCTGCCCAAAGCTCCGCCGCATCCGCATTGAGAATCTCGAGGACGCGTACGGCTTCTGCTCTTACAAGGGCCTCATTACCCTCGCCTCGCGCTGCGTCAACCTCCAACACGTAGCCATTTACGTCTCCGACATCGCAAACTCGGCCCTCCGAGCTTTCGGCACGCACTGCCCACACATGCTCGACTTCCGAATCGTCCTCCTGGAATCCACCTTGCCCGTCACGGAGCTGCCGCTTGACTCCGGCGTCCGGGCACTGCTCCAAGGCTGCCGCAAGATCACCCGCCTCGCCATCTATCTCCGCAATGGCGGCCTCACCGACGCTGGCCTGGCCGCAATTGGAAGCCTCGGCGAGCACTTGACGTGGCTGCTGCTGGGCTGCGTGGGGACCTCCGATCGGGGCCTCATTGATCTCGCTTCCGGCTGCCGGAGCTTGCAGAAGCTGGAGCTCCGCGATTGCCCCTTCACTGAGGGCGGGATTGCGGTCAGCGTCCGCCTCCTCGCGTCGCTGCGCTTCTTGTGGATCCAAAAGTACCGCGAGTCCAATCCCTATGATCTCCTCCAGATGGGTGACTGGGTGGTAGAGTACATCGTACCCAGTAGCGACACTACACCATCACAGGTGGTGGCGTATCGATCCACTGTGGGGCACCGCAGCGATTTCCCTGAGGAGGTGATCCCTCTCAGCCAGATTGCCTGGGCATTTGGCGGGGGATTTCATATTTGA

>TuCOI1 TRIUR3_17029-P1

ATGCCCTACTTCCCAGATGAAGTGGTGGAGCACATCCTTGGCTTCGTATCGTCGCACCGTGACCGCGATGCTGCATCTCTCGTGTGTCACGCGTGGTACCGCATCGAGGGCCTCACTCGCCGCTCCGTGTTCATTTCCAACTGCTATGCCGTGCGCCCAGAGCGTGTGCACGCGCGTTTCCCCTGCCTGCGCTCGCTGACCGTGAAGGGCAAGCCACGCTTTGCTGACTTCAACCTTGTCCCTGCGGGGTGGGGTGCCTCAGCGGAGCCATGGGTGGATGCGTGTGCCCGTGCGTGCCCTGGCCTTGAAGAGCTCCGCCTGAAGCGGATGGTTGTCACTGATGGTTGCCTCAATCACCTTGCTCACTCATTTCCCAATTTGAGATCACTAGTCCTTGTTAGCTGTGAGGGGTTCAGCACTGATGGCCTTGCTACTATTGCCACCAATTGCAGGTTTCTGAAGGAACTTGACTTACAAGGGAGTCAGGTGGAGTTTCGAGGCCGTCATTGGTTTAGTTGTTTCCCCAAGCCTTCGACATCATTAGAATCCTTGAATTTTGCTTGCTTGGATGGAGCAGTGAGTGCTAATGCATTGGAAAGTCTTGTTGCAAGGAGTCCAAATCTTAAAAGCTTAAGGTTAAATCGTGCAGTTCCACCAGCTGTTTTAGCCAAAATTCTTACTTCCGCTCCTAAGCTGGTGGATTTAGGTACAGGATTGGTTGCTCAAAGCAATAATGCTGGTGCACTCCCCAGTCTCTACAGTGCTATTCAACAATGCAGTTCTCTGAATAGTTTATCTGGCTTTTGGGATTCTCCACGTTGGATTACTCCAATAATACAATATATTTGCAAGAACCTAACATGCTTGAACCTTAGCTATGCTCCAATGTTTCGGACAGTTGATCTTATTGGAATTATTCGCCAATGTCAGAATCTCCGACACTTGTGGGTACTAGATCACATTGGTGATGCAGGATTAAAGGTTGTAGCCTCTTCTTGCCTGGAGCTGCAAGAGTTGAGGGTATTTCCTGCGAATGCAAATGTGTTAATAAGCACTGGTGTGACAGAGGAAGGGCTGGTTGCAGTATCTTCAGGCTGTCGGAAGCTAAACTCTGTTCTCTATTCCTGCCGTCGAATGACTAATTCTGCTCTGATCACGGTGGCAAAGAACTGCTCGCGAATCACGTCCTTCAGACTGCATATCTGCCTGCATGGGTCAGTAGATGCCGTGACAGGCCAGCCACTGGACGAGGGTTTTGGGGCAATCGTCCGGTCATGCAAGGGCCTCAGGCGCTTATCTATGTCTGGCCTTCTCACGGACAGTGTGTTCCTGTACATCGGCATGTACGCCGAGAGGCTGGAGACGCTCTCTGTCTCGTTTGCAGGAGATAGTGATGATGGCATGATCTATGTGCTCAATGGCTGCAAGAATCTCAGGAAGCTGGAGATCAGGAACTGCCCATTTGGCAACACCGCGCTTCTCGCAGGCATGCACCGGTCCCTCGCGGCAGCCATGCCAGGCCTCAACGTCGAGGTCATCAGCCAGGCGGATGGAGGCACCAACGATGCAAAGAAGAAGAAGTGGAACGCAATGTTATGGTTTCATCTTGGTGTTTGGTTGGAGCCAATGAAAAGAATGAAACGGTTTCATTTCAATGTTCTTGACGATGGTTTTGCTATTTCACATGGGTCTGAATTTTCTGGTGGGCACCGTCAGGAGTGGCGGGGGACGGGAGCTGGCACTTCAGCCTGGGTTGGGTTGGGAGGTGGGTGTACCGGAAGGAAGAAGAGGAGCGGCCGTTCTTGCAGGCATGCACAGCCGGTGGCCACTCCGGCCCCAGCGTCCACTCGCCACCACACCTCCTGCTCTGATCCCAAGCCTTTCGATCCACTATCTCACGCCATGGCCCTAGCTGATCTCAACTTCGAAATTGGTCAAGATTTTGTCGTGGAAGTCTGGAGAAAATGGGGAGTGCCAATCAACTACGAGGAAGGCAAAGACATGGAAGAGTTCCTCCTGGTTGCGGAGTTCACTCGATCACAAATCCGACTCACGGAAGAATCATTAATCACTATTCTCTTATCTTGTTTTGGAGGTAGAGCATCACTTTTTAAGGTCCAATTTCTCAGAATTGGCCGTTTAAATTCTTTGTCTCCTCCAAAGAAGTGGGGTTTTCGATCATCGAGGGAGGCAACATCTCTCTTCCTCTACCGAATATCAACTTCCTACTTTGGGGCAATGGTGGTCCTAATTCCAGTTGGGAATTGGATCAATACCTCAAAGAAAAAGAAGATGCTTGGACGCACATCTTTCATCGTCACCCTAGGAAGTCCTACATCCAGGCTCTGCAATCTCCAAGGATCTATCTAG

>TuCOI2 TRIUR3_18749-P1

ATGACCGTCTCCGACGACGAGCTCGCGCTCATCCCCAAGTCCTTCCCGCTCTTCAAGGAGCTCTCGCTCGTCTGCTGCGACGGCTTCACCACCCGCGGCCTCGCCGTCATCGCCGAGGGCTGCCGGCATCTTCGGGTACTGGATCTGACTGAAGATTATTTCCATGAGGAGGAGAGCGAGGTGGTGGATTGGATCTCCAAGTTTCCAGAGAGCAACACGTCGCTGGAATCGCTTGTATTTGATTGTGTTAGTGTCCCATTCAACTTTGAGGCCCTGGAGGCACTTGTTGCACGCTCACCAGCTCTGCGTCGTCTGCGCGTCAATGATCATGTGTCGATAGAGCAGTTGCGTCGTCTCATGGCAAGGGCACCCCATCTAACTCACCTTGGCACTGGATCATTCCGATCTGAGCCAGGCCCTGGGGGTGCTTTGTCTGTGTCTGAGCTCGCTACCTCTTTCGCAGCGTCCAGATCACTTGTTTGTTTGTCAGGTTTCTTGGATGCCAATGCAGCATACCTCCCAGCAATCTACCAAGTTTGTGCCAATCTCACTTCCCTCAATTTTAGCTTTGCGGGTCTAACTGATGAAGAGTTCATACCAGTTATTCGCCACTGCGTCAATCTTCGCACTTTATGGGTTCTTGATACTGTGGGTGATGAAGGCCTTAGGGCTGTGGCTGAAACATGCTCAAATCTCCGTGAGCTACGTGTTTTTCCTCTGGATGCCACTGAGGATTCTGAGGGCTCAGTCTCAGATATTGGTCTCCAGGCAATCTCAGAAGGCTGCCGAAAGCTCGAATCAATTCTCTACTTTTGCCAGCGCATGACAAATGCAGCAGTAATTGCTATGTCCGAGAACTGCCCTGACCTTTTGGTGTTCCGCCTCTGTATTATGGGCCGCCACCGCCCTGATCGGATTACCGGGGCGCCCATGGATGAGGGTTTTGGGGCGATTGTGATGAACTGCAAGAAGCTCACCAGACTCTCAGTCTCTGGCCTGCTCACTGATAAGGCGTTTGCATACATTGGGAGACACGGAAAACTCATAAAGACTCTGTCTGTTGCCTTTGCTGGGAATAGTGACATGTCTCTTCAGCATGTGTTTGAGGGGTGCACTAGGTTGCAGAAGCTCGAGGTCAGAGATAGCCCTTTTGGCGACAAAGGATTGCTCTCTGGCCTGAACTATTTTTACAACATGAGGTTCTTTTGGATGAACTCATGCAGGCTAACTGTGAAGGGTTGTGGGGATGTAGCTCAGCAAATGCCTAATCTGGTGGTTGAAGTAATGAAGGAAAATGAAGGGGAAATGGATACCGTTGATAAGCTGTACCTGTATCGATCGTTGGCAGGACCAAGGGAAGATGCTCCATCATTTGTCAACATCTTGTAG

>TuCOI3 TRIUR3_21512-P1

ATGACGGACATGGGACTCGGCTGCATCGCCGTCGGCTGCCCGGACCTGCGGGACCTCACGCTCAACTGGTGCCTCGGGATCACGGATTTGGGGGTCCAGCTCCTCGCCCTCAAGTGCAAGAAACTCAGGACCCTGAATCTATCCTACACCATGATCTCCAAAGACTGCCTTCCAGCCATCATGAAGCTACCCAATCTTGAGGTGTTGGCACTGGTGGGATGTGTTGGAATAGATGATGATGCCCTTAGTGGTCTTGAGAATGAATGCAGCAAATCACTACGGGTGCTCGATCTGTCAACCTGTCGAAATGTCACTCATACGGGAGTTTCATCAGTTGTGAAGGCAGTGCCAAATCTCTTGGAGTTGAATCTGTCGTACTGCTGTAATGTTACTCCATCTATGGGAAAATGCTTCCAAATGCTTCCTAAGTTGCAGACCCTGAAATTGGAAGGCTGCAAGTTCATGGCTGATGGACTAAAATACATTGGAATTTCTTGCGTCTCTTTAAGAGAGTTGAGCCTGAGCAAGTGTTCAGGAGTGACAGATACTGATCTTTCTTTTGTTGTGTCAAGACTAAAGAATTTGCTGAAGCTGGACATTACTTGCAATCGCAATATCACTGATGTTTCGTTAGCTACCATCACTAGCTCATGCCCTTCCCTCGTCTCTCTAAGAATGGAGTCCTGTAGCCATTTTTCTAGTGAAGGGCTCCGACTGATTGGGAAGCGATGTTGCCATTTGGAAGAGTTGGACATCACCGACAGTGATTTGGACGATGAAGGTTTGAAAGCTCTGTCTGGATGCAGAAAACTATCAAGCTTAAAAATTGGAATATGCATGAGGATAAGTGATGAAGGCCTTATCCACATTGGGAAGTCTTGTCCAGAACTCCGAGATATTGATTTGTATAGGTCTGGGGGTATTAGTGATGAGGGGGTTACTCAAATTGCTCAAGGTTGCCCAATGTTAGAGTCTATCAACCTGTCCTACTGTACAGAAATAACAGATGTTTCGTTGGTGTCGCTCTCAAAATGCGCAAAGCTGAACACACTGGAGATCCGTGGTTGCCCCAGTGTTTCATCTGCTGGGCTCTCAGAAATAGCAATCGGATGCAGGCTACTTGCCAAGCTTGATGTCAAGAAATGCTTTGCGATCAATGATGTGGGGATGCTTTTTCTTTCCCAGTTCTCTCATAGCCTCCGTCAGATAAACTTGTCATACTGTTCAGTCACCGACATTGGACTTCTGTCCCTCTCTAGCATATGTGGGCTTCAGAACATGACGATTGTACACTTGGCGGGTATTACACCTAATGGCTTGATGGCTGCTCTTATGGTCTCTGGTGGTTTGACAAGGGTGAAGCTTCACGCAGCGTTCAGATCTATGATGCCTCCCCATATGCTCAAAGTCGTTGAGGCTCGCGGCTGTGCTTTCCAGTGGATTGATAAACCATTCAAGGTATTCTACTTGCCCCCTTACAAGCCTAGGTCGAGCAAGAGCGATGCGACATATGGCAACAACAGTCTCGAGATGTGCTTGTACGATGAGATTATGAGAAATGCTTGTTATCGTCGTCTTGACTGTCAGCCTTACATGAACATGATGGTGTAG

>TuCOI4 TRIUR3_22304-P1

ATGTCCCTGCCCGACGTCGTGTACAGCTGTGGATCCTGTGGATATGCTCTAAATCTTTCATCTTCTAACAGAAGCACATCTGACGTAGGATCCTCGTACCAAAAATCTCTGAAGAAGGGTCTGATTTCATTCACTTCTATTGATCTCAGCCGTTTTACTCAGGTTGACGAGATATCTTGTTTTCCTTTCCTTACCTGGCGTAGCTACCGGCCGAAAACTAAGCTTCTGTGCCGAAAATGTGGATCATCTATTGGCTATGGATACGCATACAAGGAGCATGCCCAGTGCCTTCCAGAGATGTTGGCTCGTTCTCCATATCTTCAGTTGATATCCCTTGCGGGGCTTAATGAGCTATCTGATTCAGCTCTGTACGAAGTGGGAGTTTCTGGAACATCTTTGCAGTCCTTCTCATTGTACTCCTGTTCTGGTATAACAGATGATGGTCTAGCACAAGTGTCAATCGGATGTCCTAATTTGGTTATAGTGGAACTTTACCGCTGCTTAAATATCACAGATCTTGGTTTGGAAAGTCTTTCCCAGGGCTGTCATTCTTTGAAGAGTCTTAACCTTGGTTACTGCACAGCCATTTCAGATCGAGGGATTAGTTCAATCTTTAGGAATTGCCGGAACATTTGTGCACTCATCATATCATATTGCAGAGGTGTATCTGGTGTTGGATTTAGAGGTTGCCCAAGTACACTTTCTTACCTAGAAGCCGAGTCCTGCATGCTTTCTTCAGAGGGAATGCTAGACATATCGAGTGGTGGTGGACTCCAGTACATAAATTTGTATAATCTAAGGAGTTCAGCTGGGCTGGATTGCCTAGGTGGAGTTGGCAGTATGAAGAAACTCCGAGTTCTGAATCTGAGGATGTGCCGCTATCTTACTGATGATTCTGTGGTGGCAATAGCTAGTGGGTGTCCGTTGATCGAGGAGTGGAGCCTTGCTGTCTGTCATGGCGTCCGCTTACCTGGTTGGTCGGCGATTGGATTGAACTGCAACAAGCTAAGAATTCTTCATGTGAACCGTTGCCGAAACATATGTGACCAAGGATTGCATGCTCTCAAGGATGGATGTGTGCGCCTTGAGGTCCTGCATATACATGGTTGTGGCAAGATTACAAATAATGGCCTGGCACTATTTAGCATTGCTAGGCCCAGTGTGAAGCAAATGGTGGACGAAGCCATGTCCATTGGCCCCTCAATCGAGGATTTATTCCGGTTGCAGTGA

>TuCOI5 TRIUR3_23066-P1

ATGAGCTTCGGGATCCCGGACGTGGCGCTGGGGCTGGTCATGGGGTGCGTGGAGGACCCCTGGGACCGCGACGCCATCTCGCTCGTCTGCCGCCACTGGTGCAAGGTCGACGCGCTCAGCCGCAAGCACGTCACCGTCGCCATGGCCTACTCCACCACCCCCGACCGCCTCTTCCGCCGCTTCCCCTGCCTCGAGTCGCTCAAGCTCAAGGCCAAGCCCCGCGCCTCCATGTTCAACCTCATCCCCGAGGACTGGGGAGGCTCCGCCTCGCCCTGGATCCGCGAGCTCTCCGCCTCCTTCCACTTCCTCAAGGTGCTGCACCTCCGCCGGATGATTGTCTCCGACGACGACGTCGCCGTGCTCGTGCGCGCCAAGGCCCACATGCTCGTCTCCCTCAAGCTTGACCGCTGCTCCGGCTTCTCCACCTCCTCCCTCGCTCTCCTCGCCCGCTGCTGCAAGAAACTGGAAACGTTGTTTCTTGAAGAAAGTTCTGTTGCTGAGAAAGAAAATGATGAATGGCTCCGTGAGCTTGCTACCAGCAATACTGTCCTTGAGACGCTGAATTTCTTTCTGACGGATCTCAGGGCATCCCCTGCACATCTTCTCCTCCTTGTGCGAAATTGCCGAAGGCTGAAAACTCTCAAGATTAGCGACTGTTTCATGTCTGACCTGGTCGACCTGTTCCGTACAGCAGAAACACTACAAGACTTTGCTGGTGGTTCCTTTGATGATCAAGATCAAGGTGGGAATTATGCTAACTACTATTTCCCTCCTTCAGTACAGCACTTGAGTTTGCTCTACATGGGAACAAATGAGATGCAGATATTATTTCCATATGGTGCCACACTCAAGAAGTTGGACCTTCAGTTCACATTCCTTACCACAGAGGATCACTGTCAATTAGTCCAGCGCTGCCCAAATCTAGAAGTTTTGGAGGTGAGGGATGTGATAGGAGATCGAGGGTTAGAAGTTATTGCGCAGACCTGCAAGAAATTACAGCGACTCAGAGTCGAGAGAGGAGATGATGACCAAGGAGGTCTTGAGGACGAACAGGGTAGAGTGACACAAGTAGGATTGATGGCTGTAGCTGAAGGCTGTCCTGATTTGGAGTACTGGGCAGTACATGTGTCTGACATTACAAATGCAGCTCTTGAGGCTATTGGCGCATTCAGCAAAAACCTGAACGATTTCCGACTTGTCCTGCTTGATAGAGAGGTGCATATAACTGAACTGCCCCTTGACAACGGGGTTCGGGCTTTGCTGAGAGGTTGCACCAAACTCCGGAGGTTTGCATTTTATGTGAGACCTGGAGCTCTATCAGATATTGGCCTTTCTTATGTTGGCGAATTTAGCAAGACCGTCCGCTACATGTTGCTTGGGAATGCCGGGGGGTCTGATGATGGACTGCTGGCATTTGCACGAGGATGCCCAAGCTTGCAGAAATTGGAGCTAAGGAGTTGCTGCTTTAGTGAACGTGCATTGGCAGTTGCAGCCTTACAGCTGAAGTCACTCAGATATCTTTGGGTGCAGGGATACAAGGCATCTCCTACTGGCACCGATCTCATGGCAATGGTACGCCCCTTCTGGAACATTGAGTTTATTGCACCAAATCAAGATGAGCCTTGCCCAGAGGGTCAGGCACAGATTCTGGCATACTACTCTCTGGCTGGGGCAAGGACAGATTGTCCTCAGTCAGTAATTCCCCTCCATCCGTCAGTGGGAAGCTAA

>TuCOI6 TRIUR3_23956-P1

ATGACCGTCACCGACGACGACCTCGGCCTCGTCGCGCGCTCCTTCCCGGGCTTCAAGGAGCTCTCCCTCGTCTGCTGCGACGGCTTCAGCACCCTCGGCCTCGCCGTCATCGCCGAGCGTTGCCGGCACCTGAGGGTTCTGGATCTGATCGAGGACTATGTGGATCAGGAGGACGAAGCAGTGGACTGGATCTCCAAGTTCCCAGTGTCAAACACATCCCTAGAATCCCTCATGTTTGACTGCGTTGCTGTCCCATTCAACTTTGAGGCCCTAGAGGCCCTTGTGGCACGCTCACCCTCTCTTCGCCGGCTGCGTGTGAACCACTACGTTTCGGTGGAGCAGCTACGCCGTCTCATGGCACGGGCTCCGCAGCTCACACACCTTGGCACTGGGGCCTTTCGTCCTGAGGCTCCACAAGGTGGAGGCATGTCGGTGTCTGAACTCGCACCCTCTTTTGCAGCCTCAACGTCTATAGTCTGCCTCTCTGGGTTTCAGGAGGTCAACCCTGAATACCTTCCAGCAATCTACCCAGTGTGTGGTAATCTCACCTCCCTTAACGTTAGCTTCGCGAGCCTAACTGCTGAGGATCTGACACCAGTCATTCGCCAATGCCACAAACTTCAGACATTCTGGGTTCTTGATACTGTGGGTGATGAAGGCCTCAGGGCTGTGGCTGAGACATGCTCTGATCTTCGTGAGCTGCGAGTATTTCCGCTGGATGCCACCGAAGACTCTGATGGGTCTGTGTCAGATGTTGGGCTTCAGGCTATATCCGAAGGTTGCCGAAAGCTTGAATCCATACTTTACTTTTGCCAGCGGATGACAAATGCTGCGGTCGTAGCTATGTCCAATAACTGCCCTGACCTTGTGGTATTCCGTCTTTGCATCATGGGTCGCCACCGCCCTGATCGTATTACTGGGGAGCCCATGGATGACGGCTTCGGCGCGATAGTGAAGAACTGCAAGAAACTTACTAGGCTCTCAGTCTCTGGCCTGCTCACTGATACGGCATTTGCATACATTGGGCAATATGGCAAACTAATAAAGACTTTGTCTCTTGCCTTCTCGGGGAACAGTGACCTGTCGCTTCAGTTTTTGTTCGAGGGATGCACTCGGTTACAGAAGCTTGAGGTCAGAGATAGCCCATTCAGTGATAGGGGACTGCTTTGTGGTTTGGATTATTTCTACAACATGAGGTTCCTGTGGATGAATTCATGCAGGCTAACCATGAGGGGTTGTAGAGAGGTAGCTCAGCGGATGCCTAACTTGGTTGTTGAAGTAATGGAGGAGCAAAATGAGGATAAGGTGGAAACAGAGACTGTTGATAAGTTGTACCTGTACCGCTCACTAGCAGGGCCAAGGGGTGATGCACCGCCATTGGTGAAAATTTTGTAG

>TuCOI7 TRIUR3_24621-P1

ATGGCTGCCATGCGTGCCGATCGCCAGATCTTGGAGGAGCACCTCGACTTCGAGGCCACCGTCGACACCGCCACATGGTTGTGTACTTTAAAATACAGTTCGTGCTGTTTGAATATACCCACGGGACGTGCTAGTCCTACTGGGCATCATTGCTGCAATGGGAGTATCATACAAGCACGTATCTGTCAGAATCGGGGCTGCGGGGAATCAAGAGGATTGCTTGGGGGAGAATTCAATAAGCCAATTTTGATGAAGCTTGCCAATACATTCAATGTAGTGACCCTTGTGGCTGCAACGTATCATGTTTGCAGGTTTACTGTAATCGAATGGAAGGTTTTGGGTGGAGGAATCGGGACTGGCGGTCGCAATCCTCCTAGCCATTTTGGTGCTGTCCGTTCTGGGTTACTAGGGGGTTTGCGTGTTCTAAATAGGTGTTGGTTGCCAGTTTTAGCTCAACAAATCTGGCTGGAGCCTGATCTGCCCAAGCCAGCATTAGTTGTGTGCTCTCTGTGTCTGGTTGAGCAAGGTTGCAGCTTCCATTTGTTGATTTCGTCGGCTTTGTTTAGTAGCTTTGATAACCTGGAGACTGCAAGCAAACAGATCCCGTCCTGCAGCTCTAGTAATAAGGTGGTGCTGGGGGAAGGCACCATGACCTACTTTCCGGAGGAGGTGGTGGAGCACATATTCAGCTTCTTGCCCGCGCAATGTGACCGGAACACCGTTTCACTTGTTTGCAAGGTATGGTATGAGATTGAAAGGCTGAGCCGGCGAACTGTCTTTGTGGGTAACTGCTATGCTGTGCGCCCTGAGCGCGTGGTGCTTCGGTTCCCCAATGTGCGGGCACTGACAGTGAAGGGGAAACCACACTTCGCTGATTTCAACCTTGTGCCACCTGATTGGGGTGGGTACGCCGGACCATGGATCGAGGCAGCAGCCAGGGGCTGCGTGGGTCTTGAGGAGCTGCGGATGAAGCGGATGGTGGTGTCAGATGAGAGCCTGGAGCTGCTTGCCAAATCATTCCCACGATTCAGGGCCCTAGTTCTTATCAGCTGTGAGGGGTTCAGCACCGATGGACTAGCAGCTATTGCAAGTCACTGCAAGCTCCTGAGGGAGTTAGATTTGCAGGAAAATGAGGTGGATGATCGAGGGCCAAGGTGGCTCTCCTGCTTCCCTGATTCCTGCACGTCCCTTGTCTCCTTGAATTTCGCCTGCATCAAAGGGGAGGTTAATGCTGGTTCATTAGAGAGACTTGTTGCTAGGTCCCCAAGTCTTCGGAGTTTGAGGTTGAATCGATCTGTGTCAGTAGATACACTCTCGAAGATATTAATGCGCGCCCCTAATTTGGAGGATCTAGGGACTGGGAACTTGACAGATGACTTCCAAGCTGAATCGTATCTCAGGCTGACCCTTGCATTGGAGAAATGCAAACTGCTGAGGAGTTTATCGGGCTTTTGGGATGCTTCGCCTTTGTGCCTTCCATTCATCTATCCTGTATGTGGGCAACTAACAGGTTTAAACTTGAGCTATGCTCCGACACTTGATTCTTCTGATCTCACCAAAATGATCAGCCACTGTGTGAAACTCCAACGTCTTTGGGTACTGGATTGCATCGCGGATAAGGGCTTGCAAGTGGTGGCCTCCAGTTGCAAGGATCTACAAGAACTCAGGGTATTCCCATCAGACTTCTATATCGCCGGGTATTCCCCAGTAACAGAGGAGGGACTTGTTGCAATATCCTTGGGCTGTCAAAAACTGAGCTCATTGCTATATTTTTGTCATCAAATGACGAATGCCGCACTGCTTACTATAGCTAAGAACTGCCCAAATTTCACGCGGTTCAGACTCTGTATTCTTGAGCCTGGGAAGCCTGATGCCATGACAAACCAACCATTAGATGAAGGTTTTGGTGCTATTGTTCGTGAATGCAAAGGGCTAAGGCGATTGTCAATATCGGGTCTTCTCACCGACAAGGTTTTCATGTATATTGGTAAATTCGCGAAACAACTTGAGATGCTTTCAATAGCATTTGCTGGAGATAGTGATGCGGGAATGATGCATGTTATGGAAGGATGCAATAATCTGAGGAAGCTGGAGATTAGAGATAGCCCATTTGGTGATGCTGCACTCCTGGAGAATGTTACCAAGTATGAGACAATGCGATCCCTTTGGATGTCATCATGCAATGTCACAGAAAAGGGGTGCCAAATCCTTGCATCAAAGATGCCAATGCTTAATGTGGAGGTTATAAATGAGGTAGATGAGAGCAATGAAATGGATGAGAACCATGGAATCCCCAAAGTTGACAAGTTATATGTTTACCGCACAACTGCTGGGGCAAGGGATGATGCGCCAAATTTTGTTAAAATCCTATAG

>TuCOI8 TRIUR3_31653-P1

ATGGTCGTCACCGACGACGACCTCGCCGCCCTCGTCCGCGCCCGCGGCCACATGCTGCAGGAGCTCAAGCTCGACAAGTGCTCCGGCTTCTCCACCGACGCCCTCCGCCTCGTCGCCCGCTCCTGCAGATCACTGAGAACTCTGTTTCTGGAAGAATGTACAATTACTGATAATGGCACCGAATGGCTCCATGACCTTGCTGCCAACAATCCTGTTCTGGTGACCTTGAACTTCTACTTGACTTACCTCAGAGTGGAGCCAGCTGACCTCGAGCTTCTCGCCAAGAATTGCAAGTCACTAATTTCGTTGAAGATTAGCGACTGTGACCTTTCAGATTTGATTGGATTTTTCCAAATAGCTACATCTTTGCAAGAATTTGCTGGAGCGGAAATCAGTGAGCAAAAGTATGGAAATGTTAAGCTTCCTTCCAAGCTTTGCTCCTTCGGACTCACCTTCATGGGGACAAATGAGATGCACATAATCTTTCCTTTTTCTGCTGTACTCAAGAAGCTGGATTTGCTGTACAGTTTCCTCACCACTGAAGATCATTGCCAGCTCATTGCAAAATGTCCAAACCTACTCGTTCTTGCGGTAATGGCGGTGCTTGTACATTACTACATTATGAAGGTGAGGAATGTGATTGGAGATAGAGGATTGGGGGTTGTCGGAGACACATGCAAGAAGCTACAAAGGCTCAGAGTTGAGCGAGGGGAAGATGATCCTGGCATGCAAGAAGAGGAAGGCGGAGTTTCCCAAGTAGGCCTAACAGCGATAGCCGTAGGCTGCCGTGAACTGGAAAACATAGCTGCCTATGTGTCCGATATCACAAATGGAGCCCTGGAATCCATCGGAACGTTCTGCAAAAACCTCCATGACTTTCGCCTTGTCCTGCTCGACAGACAAGAGACGATAACAGAATTGCCGCTGGATAACGGTGCCCGCGCGCTGCTGAGGGGCTGCACCAAGCTTCGGAGGTTCGCTCTGTACCTGAGACCAGGGGGGCTTTCAGATGTAGGCCTCGGCTACATCGGGCAGCACAGCGGAACCATCCAGTACATGCTTCTGGGTAACGTCGGGCAAACGGATGGTGGGTTGATCAGTTTCGCAGCCGGGTGCCGGAACCTGCGGAAGCTTGAACTGAGGAGCTGCTGCTTCAGCGAGCGGGCTCTGGCCCTCGCCATACGGCAAATGCCTTCCCTGAGGTACGTGTGGGTGCAGGGGTACAGGGCCTCTCAGACCGGCCGCGACCTCATGCTCATGGCGCGGCCCTTCTGGAACATCGAGTTCACGCCTCCCAGCACGGAGACCGCGGGCCGGGTGATGGAAGACGGGGAGCCCTGCGTTGACAGGCAAGCTCAGGTGCTGGCGTACTACTCCCTCTCTGGGAAGAGGTCCGACTACCCGCAGTCTGTTGTTCCTCTGTATCCTGTGTGA

>TuCOI9 TRIUR3_32567-P1

ATGGCGGAGTGCCGGAGTGCGGGCGCAGCGTCCGACCTTGGGCAGAGCGCCGGAGTGGGAGAGGGAGGAGCTGGATGGGCTCGAACTGGCGGTGCAGAGAGGGAGAATGGGGGCAAGCCCCACTTCGCGGACTTCGGGCTCGTCCCGCCCGCCTGGGGCGCCGAGGCCGCGCCCTGGGTCGCCGCCGCCGCCGAAGGCTGGCCGCTTCTCGAGGAGCTCAGCTTCAAGCGCATGGTCGTCACCGACGAGTGCCTGGAGATGATCGCCTCCTCCTTCCGGAACTTCCAGGTCCTGCGCCTCGTCTCATGCGAGGGATTCAGCACCGCCGGCCTCGCCGCCATTACCGAAGGTTGCAGAAATTTAAGAGAACTTGACCTGCAAGAGAACTACATTGAGGATTGTTCCAGTCATTGGCTCAGTTACTTTCCGGAATCCTTTACTTCTCTGGAAACTCTGAACTTTTCATGCTTAGAAGGGGAGGTCAATTTCGCTGTACTCGAGCGGCTAGTGAGCAGATGCCGCAACCTCAAGACTCTGAAGCTCAACAATGCTATCCCTCTTGACAAGGTTGCTAGCCTTCTTCGTAAGGCTCCGCAACTAGTAGAACTCGGAACTGGCAAATTCTCTGCTGAGTATCATTCAGATCTCTTTGCAAAGCTCGAAGCGGCATTTGCAGGTTGTAAAAGCCTGAGAAGGCTCTCTGGGGCTTGGGATGCTGTGCCAGATTACCTGCCAGCATTCTACTGTGTATGTGAAGGCCTCACATCACTCAATCTGAGTTATGCTACTGTGCGAGGCCCTGAGCTAATAAAATTTATTAGCAGATGCAAGAATCTGCAGCAATTATGGGTGATGGACCTAATCGAAGACCATGGTCTAGCTGTCGTGGCAGGCTCTTGCAGTAAACTGCAAGAGCTGCGGGTCTTCCCGTCTGACCCTTTTGGTGCCGGGCAAGTTTTATTGACTGAAAGAGGCCTTGTTGATGTTTCTGCCAGCTGCCCCATGTTGGAGTCAGTTCTCTACTTCTGTGGCCAGATGACGAATGAGGCCCTCATTACAATCGCAAAGAACCGTCCCAACTTCACTTGCTTCCGGTTATGCATCCTTGAGCCCCGTACTCCAGATTACGTCACACGGGAGTCTCTTGATGCTGGCTTTAGTGCCATTGTGGAATCATGCAAGGGCCTTAGGCGCCTCTCTGTGTCTGGCCTTCTCACAGATCTCGTGTTCAAATCAATCGGTGCAAATGGTAATTGTCTTGAGATGCTGTCAATCGCCTTTGCTGGGAACAGTGATCTTGGCCTGCATTATATCCTCTCTGGCTGCAAGAGCCTGAAGAAGCTGGAGATCAGGGACTGCCCATTTGGAAATAAGCCGTTGCTGGCAAATGCTGCCAAGCTGGAGACAATGCGATCCCTTTGGATGTCGTCGTGCTCATTGACCCTGGGCGCATGCCGACAGCTTGCAGAGAAGATGCCCCGCCTTACCGTGGAGATAATGAACGATCCTGGAAGAATATGCCCTGTGGAGTCACTTCCGGATGATAGCCCGGTCGAGACATTGTATGTGTACCGGACAATTGCCGGTCCAAGGTCCGACACACCAGATTACGTCCAGATTGTTTAA

>TuCOI10 TRIUR3_34918-P1

ATGGCTGGAGAGGATGTCGACCCCAAGTCCAGTGCTTCTGCTGCTGCTGTCCCAGGGCTGCGTGTGCTCGACGTGGTGGACTGCGAGCTCAACGAGGAGGAGGACGACGAGGTGTCGGACTGGGTGGCGGCGTTTCCGCGCGGCCACACCGACCTGGAGTCTCTCTCCTTCGAGTGCTTCACCCCGCAGGTACCCTTCGCCGCCCTCGAGGCTCTCGTGGCGCGCTCGCCGCGCCTCCGCCGCCTGCGCGTCAACCAGCACGTCTCGCTCGGCCAGCTGCGCCGGCTCATGGCGCTCACGCCTCGCCTCACGCACCTCGGCACGGGCTCGTTCCGGCCGGGGGACGGCGCCGAGGACGAGGGGCTCGACTTTGGTCAGATGTTGACCGCCTTCGCGTCGGCCGGCCGGGCTAACTCGCTGGTGTCGCTGTCCGGCTTCCGTGATCTCGCGCCGGAGTACCTGCCGACCATTGCCACGGTCGCCGCCAACTTAACCAGCATGGACCTGAGCTACGCCCCTGTCAACCCCGACCAAGTCCTGCTCTTCATCGGGCAATGCCGCAGCCTCGAGACGCTATGGGTGCTCGACTCAGTGCGCGACGAGGGGCTCCAAGCCGTGGCCATGTACTGCAAGAAGCTCCAGGTTCTCCGTGTGCTCCCATTGGACGCGCACGAGGACGCCGACGAGCTGGTGTCGGAGGTCGGGCTCACCGCCATCTCGGAGGGCTGCCGTGACCTTCGCTCCATCCTCTACTTCTGCCAGAGGATGACCAACGCCGCCGTCATCACCATGTCGCAGAACTGCCCCGAGATGACGAACTGCCCCGAGATGAAGGTGTTCCGGCTATGCATAATGGGGAGGCACCGGCCCGACCACGTGACGGGGGAGCCGATGGACGAGGGGTTCGGCGCCATTGTCCGCAACTGCAGCAAGCTCACCAGGCTCTCCACGTCCGGGCACCTGACGGACCGAGCATTCGAGTACATCGGCAACTACGGCAGCTCCCTGCGGACGCTCTCCGTGGCGTTCGCCGGGGACAGCGATCTGGCGCTGCAGCACATCCTCCAGGGCTGCTCCAAGCTGGAGAAGCTGGAGATAAGGGACTGCCCGTTCGGCGACGCCGGCCTGCTCTCCGGTATGCACCATTTCTACAACATGCGGTTCGTCTGGATGTCGGGCTGCAGCCTGACGCTGCAAGGCTGCGAGGAGGTGGCCCGGCAGCTCCCACGGATGGTGGTGGAGCTGATAAACAGCCAGCCTGAGATCGAGAAGACAGACGGCGTCGACATCCTATACATGTATCGGTCGCTGGAGGGGCCAAGGGAGGATGTTCCACCATTTGTGAAGATCCTGTGA

>TuCOI11 TRIUR3_35106-P1

ATGTACGGCCTCATCTCCGACGACTGGGGCGCCTACGCCGCGCCCTGGGTCGCCCGGCTCGCCGCGCCGCTCGAGTGCCTCAAGGCGCTCCACCTGCGACGCATGACCGTCACCGACGACGACGTCGCCGCGCTCATCCGCTCCCGCGGCCACATGCTGCAGGAGCTCAAGCTCGACAAGTGCTCCGGCTTCTCCCCCGCCGCGCTCCGCCTCGTCGCCCGCTCCTGCAGATCCTTGAGAACATTATTTCTTGAAGAATGTGTGATTACTGATGAAGGTGGTGAATGGCTTCATGAACTTGCTGTCAACAATTCTGTTCTTGTGACACTGAACTTCTACATGACTGAGCTCAAAGTGGTGCCAGCTGATCTGGAGCTTCTAGCAAAGAACTGCAAATCATTACTTTCTTTAAAGATCAGTGAGTGTGACCTTTCAGACCTGATTGGTTTTTTCGAAGCAGCCAATGCATTGCAAGATTTTGCTGGAGGATCGTTCAATGAGGTAGGAGAGCTAACAAAGTATGAAAAAGTCAAGTTTCCACCAAGAGTATGCTTCTTGGGGCTTACGTTCATGGGGAAAAATGAGATGCCTGTTATCTTCCCTTTTTCTGCTTCATTAAAGAAACTGGACTTGCAGTACACTTTCCTCACCACTGAGGATCATTGCCAGCTTATCTCAAAATGCCCGAACCTATTTGTTCTCGAGGTGAGGAATGTGATAGGAGACAGAGGGCTAGAGGTTGTTGGTGATACATGCAAGAAGCTACGAAGACTTCGAATTGAGCGAGGGGATGATGATCCAGGTCTCCAAGAAGAGCAAGGAGGAGTTTCTCAGTTAGGCCTGACAGCGGTAGCTGTTGGTTGCCGTGACCTGGAGTACATAGCTGCCTATGTATCTGATATCACCAACGGCGCTCTCGAATCCATCGGGACCTTCTGCAAAAATCTCTACGACTTCCGGCTTGTCCTGCTCGACAGACAAAAGCAGGTAACCGATCTGCCGCTCGACAACGGTGTTCGAGCTCTGTTAAGGAGCTGCACCAAGCTCCGGAGATTTGCTCTCTACCTGAGACCTGGAGGGCTCTCAGACACAGGCCTCGACTACATCGGGCAGTACAGCGGCAACATCCAGTACATGCTACTGGGCAACGTCGGCGAATCGGACCACGGGTTGATCCGCTTCGCAATAGGATGCACCAACCTGCGGAAGCTTGAGCTTCGGAGCTGCTGCTTCAGCGAGCAAGCCCTGTCCCTCGCGGTGCTCCACATGCCCTCGCTCAGGTACATATGGGTGCAAGGCTACAAGGCCTCTCCAGCAGGCCTGGAGCTCCTCCTCATGGCGAGGCGATTCTGGAACATCGAGTTCACGCCCCCCAGCCCCGAGGGCTTGTTCCGCATGACGCTCGAAGGAGAACCCTGCGTGGACAAGCAGGCCCAGGTTCTTGCCTACTACTCCCTTGCCGGGCAGAGGCAGGACTGCCCTGACTGGGTGACCCCGTTGCATCCAGCCGCATGA

>ZmaCOI1 ZM01G10730

ATGGGCGGCGAGGCCGAGGGCGGGGAGCGGCGGCTGGGGCGGGACCCCTGGGACCGCGACGCCATCTCGCTGGTGTGCCGCCACTGGTGCCGCGTCGACGCGCTCAGCCGCAAGCACGTCACGGTGGCCATGGCCTACTCCACAACACCCGAGCGCCTGTTCGGCCGCTTCCCGTGCCTCGAGTCACTCAAGCTCAAAGCAAAGCCCCGCGCGGCCATGTTCAACCTCATCTCCGACGACTGGGGCGGGTCTGCGTCGCCGTGGATCCGACAGCTCTCGGCCACTTTCCACTCCCTCAAGAAGCTCCACCTGCGCAGGATGATAGTATCCAATGACGACATCAACACGCTCGTCCGCGCCAAAGCCCATATGCTTGTGTCGCTGAAGCTCGACCGCTGCTCAGGCTTCTCCACGCCCTCCATCGCACTCATCGCCCGCTCTTGCAGGAAACTGGAAACACTTTTCCTGGAAGAAAGCATGATTGATGAGAAAGAAAATGATGAATGGATCCGTGAGCTTGCTACGAGCAATTCTGTTCTTGAGACACTGAATTTCTTTCAGACAGATCTCAGGGCATCCCCAGAGTATCTTACCCTTCTTGTGCGCAACTGTCAAAGGCTGAAAACCCTGAAGATTAGTGAGTGTTTCATGCCTGATCTGGTTAGTTTGTTCCGAACTGCACAAACACTACAAGAATTCGCTGGTGGTTCCTTTGAAGATCAGGGTCAACCTGTGGCAGGTAGAAATTATGAGAACTACTATTTTCCTCCTTTACTGCACCGCTTGAGTTTGCTCTACATGGGAACAAATGAGATGCAGATACTGTTTCCATATGCTGCTGCACTTAAGAAGTTAGACCTTCAGTTTACATTCCTTTCCACAGAGGATCACTGTCAGATAGTTCAGCGCTGCCCCAATCTGGAAACCTTAGAGGTGAGGGATGTCATAGGGGATCGTGGGCTACAAGTTGTTGCAGAGACCTGCAAGAAATTGCAGAGGCTCAGAGTAGAGAGAGGAGATGATGATCAAGGAGGTCTTGAGGATGAACAAGGTAGGATTTCACAGGTTGGGGTGATGGCTATAGCCCAAGGCTGCCCTGAGTTGACATACTGGGCAATATATGTATCGGACATTACAAATGCAGCTCTAGAAGCGGTTGGTACATGCAGCAGAAATCTTAATGATTTCCGCCTTGTCCTTCTTGATAGAGAAGCACATATAACTGAGTTGCCACTGGACAATGGGGTTCGTGCTTTGCTTAGAGGTTGCACCAAACTCAGGAGGTTTGCATTTTATGTGAGACCTGGGGTCCTATCTGATGTTGGTCTTGGCTATGTTGGAGAATTTAGCAAGAGCATTCGATATATGTTGCTTGGCAATGTTGGTGAATCTGATAATGGAATTATACAATTATCAAAAGGCTGCCCAAGCTTGCAAAAACTGGAGCTGAGGGGTTGTTTGTTTAGTGAGCATGCTTTAGCTATGGCTGCACTAGAGCTCAAGTCACTGAGGTATCTGTGGGTGCAAGGATTCAGGTCATCTCCAACCGGAACTGATCTTATGGCAATGGTACGCCCCTTCTGGAACATTGAGTATATTCTTCCAGACCAAGATGAACCTTGCCCAGAGTATAAGAAACAGATTCTGGCATACTACTCCCTTGCTGGAAGGAGGACAGATTGCCCTCCATCGGTAACTCCACTTTACCCAGCAGTTTGA

>ZmaCOI2 ZM03G26700

ATGGGCGGCGAGGTGCCGGAGCCGCGCCGGCTGAGCCGCTCGCTCAGCTTCGGCTGCGGCGGCGTCCCCGAGGAGGCGCTGCACCTCGTGCTCGGCTACGTGGACGACCCGCGCGACCGGGAGGCGGCCTCGCTCGTGTGCCGCCGCTGGCACCGCATCGACGCGCTCTCGCGCAAGCACGTCACCGTCGGCTTCTGCTACGCCGTGGAGCCCGCGCGGCTGCTCGCCAGGTTCCGGCGGCTCGAGTCGCTCGCGCTCAAGGGGAGGCCCCGCGCCGCCATGTACGGGCTCATCCCCGAAGACTTCGGCGCCTACGCGGCGCCCTGGGTCGCCGAGCTCGCCGCGCCGCTCGACTGCCTCAAGGCGCTCCACCTGCGCCGCATGACCGTCACCGACGAGGACATCGCCGTGCTCGTCCGCGCGCGCGGCCACATGCTACAGGTGCTCAAGCTCGACAAGTGCTCCGGCTTCTCAACGGACGCCCTCCGCCTCGTCGCCCGCTCCTGCAGATCTCTGAGAACTTTGTTCCTGGAAGAATGTATAATTGCCGATGAAGGGAGCGAATGGCTCCATGAACTCGCCGTCAACAATTCTGTTCTGGTGACACTGAACTTCTACATGACAGAACTCAAAGTGGAGCCTGCCGATCTGGAGCTTCTTGCAAGGAACTGTAAATCATTGATTTCTCTGAAGATGAGTGACTGCGATCTTTCGGATTTGATTGGTTTTCTCCAAACCTCCAAGGCACTGCAAGAATTCGCTGGAGGCGCTTTTTTCGAAGTCGGAGAGTACACCAAGTACGAAAAGGTCAAGCTCCCACCTAGGCTATGCTTCTTGGGGGGTCTTACCTTCATGGGTAAAAACGAGATGCCCGTTATCTTTCCGTATTCTGCGTCGCTTAAGAAACTGGACCTGCAGTACACTTTCCTCACCACTGAAGATCACTGTCAGCTTATCGCTAAATGCCCCAACCTACTGGTTCTCGAGGTGAGGAATGTGATTGGGGATAGAGGACTGGAAGTTGTTGCCGATACATGCAAGAAGCTACGAAGGCTGAGAATCGAGAGGGGGGACGATGACCCCGGCCAAGAAGAGCAGGGAGGAGTCTCTCAGATAGGCCTGACAGCCGTAGCTGTCGGCTGCCGGGAACTGGAGTACATAGCGGCCTACGTATCCGACATCACAAACGGGGCGCTGGAGTCCATCGGCACCTTCTGCAAGAACATGTACGACTTCCGGCTCGTTCTACTCGACAAGCAGAACAAAATAACGGATCTGCCGCTGGACAACGGTGTCCGAGCTCTACTGAGGAGCTGCACCAAGCTTCGGCGGTTCGCCTTGTACCTGAGACCGGGAGGGCTCTCGGATGCAGGCCTCGGCTACGTCGGACAGTACAGCGGGAACATCCAGTACATGCTGCTCGGCAACGTCGGCGAATCGGACAACGGGCTGATCCGGTTCGCGATGGGGTGCGCCAACCTGCGGAAGCTGGAGCTGAGGGGCTGCTGCTTCAGCGAGCGGGCCCTGGCGGTGGCCGTGCTCCAGATGCCCTCGCTGAGGTACGTATGGGTGCAGGGGTACAGGGCCTCTCAGACGGGCCGGGACCTCATGCTCATGGCCAGGCCGTACTGGAACATCGAGTTCGTGCCTCCCCGTCCCGAGAGCGCGTGTCGGGTGATGGCGGATGGACAGCCTTGTGTCGACACCCATGCTCAGGTTCTCGCGTACTATTCCCTCGCTGGAAGGAGGCCGGACTGTCCGCGGTGGCTGGTTACTTTGCATCCTGCGTGA

>ZmaCOI3 ZM06G24870

ATGGGTGGGGAGGCGCCGGAGCCGCGGCGGCTGACCCGCGCTCTGAGCATCGGTGGCGGTGGCGGCGGCTGGGTTCCCGATGAGATGCTGCAACTGGTGTTGGGGTTTGTTGAGGACCCGCGCGACCGGGAGGCCGCGTCGCTGGTGTGTCGCCGGTGGCACCGCGTCGACGCGCTCTCGCGGAAGCACGTGACGGTGCCCTTCTGCTACGCCGTGTCCCCGGCACGCCTGCTCGCGCGGTTCCCGCGGCTCGAGTCGCTCGCGGTGAAGGGGAAGCCCCGCGCGGCCATGTACGGGCTCATACCCGACGACTGGGGCGCCTACGCCCGCCCGTGGGTCACCGAGCTCGCCGCGCCATTCGAGTGCCTCAAGGCGCTCCACCTGCGACGCATGGTCGTCACAGACGACGACCTCGCCGAGCTCATCCGTGCCAGGGGGCACATGCTGCAGGAGCTGAAGCTCGACAAGTGCACCGGCTTCTCCACGGATGGACTCCGCCTCGTCGCCCGCTCATGCAGCTCACTGAGAACTTTGTTTTTGGAAGAATGTCAAATTAATGATAAAGGCAGTGAATGGATCCGCGATCTTGCAGTCAACTGTCCTGTTCTGGCGACATTAAATTTCCACATGACTGAGCTTGAAGTGATGCCAGTTGACCTAGAGCTTCTTGCAAAGAGCTGCAAGTCACTGATTTCCTTGAAGATTGGCGACTGTGATCTTTCAGATTTGATAGGGTTCTTCCAATCTGCCACATCACTGGAAGAATTTGCTGGAGGGACATTTAATGGGCAAGGGGAACTCACCAAGTATGGGGATGTTAAATTTCCATCAAGAATTTGCTCCTTGGGACTTACTTTCATGGGAGCAAGTGAAATGCCTATTATATTTCCTTTTGCTGCTATACTAAAGAAGCTGGATTTGCAGTACACTTTCCTCACCACCGAAGACCATTGCCAGCTCATTGCAAAATGCCCGAACTTACTAGTTCTCGCGGTGAGGAATGTGATTGGGGATAGAGGATTAGGTGTTGTTGCAGATACGTGCAAGAAGCTCCAAAGGCTCAGAATTGAGCGAGGAGACGATGAAGGAGGTGTGCAAGAAGAGCAGGGAGGGGTCTCTCAAGTGGGCTTGACAGCTATAGCGGTAGGCTGTCGTGAACTGGAATACATAGCTGCCTATGTGTCTGATATAACCAATGGGGCCTTGGAATCTATCGGGACATTCTGCAAAAAACTCTATGACTTCCGGCTTGTTCTGCTCGATAGAGAAGAGAGGATAACTGATTTGCCACTGGACAATGGTGTCCGAGCTTTGTTGAGGGGCTGCACCATGCTTCGGAGGTTTGCTCTGTACTTGAGACCAGGAGGGCTCTCAGATGCAGGTCTCGGCTACATTGGACAGTGCAGTGGAAACATTCAATACATGCTTCTCGGTAACGTTGGGGAAACCGATGATGGATTGATCAGTTTCGCACTGGGATGTGTAAACCTGCGAAAGCTTGAGCTCAGGAGCTGTTGCTTCAGCGAGCGAGCTCTGGCCCTCGCAATACTAAGTATGCCTTCCCTGAGGTACGTATGGGTTCAGGGCTACAAAGCGTCTCAAACAGGCCGAGACCTGATGCTCATGGCGAGGCCCTTCTGGAACATAGAGTTTACACCTCCCAGTTCCCAGAATGCTGGTCGGTTGATAGAAGATGGGGAACCTTGCGTAGATAGTCATGCTCAGATACTGGCATACGGCTCTCTTGCTGGTAAGAGGTTGGACTGCCCACAATCCGTGGTCACTTTGTATCCTGCGTGA

>ZmaCOI4 ZM08G14420

ATGGGTGGGGAGGCGCCGGAGCCGCGGCGGCTGACCCGGGCGCTGAGCATCGGCGGCGGTGACGGCGGCTGGGTTCCCGAGGAGATGCTGCAACTCGTGATGGGGTTCGTCGAGGACCCGCGCGACCGGGAGGCCGCGTCGCTGGTGTGTCACCGGTGGCACCGCGTCGACGCGCTCTCGCGGAAGCACGTGACGGTGCCCTTCTGCTACGCCGTTTCCCCGGCACGCCTGCTCGCGCGGTTCCCGCGGCTCGAGTCGCTCGCGGTGAAGGGGAAGCCCCGCGCGGCCATGTACGGGCTCATACCCGACGACTGGGGCGCCTACGCCCGCCCGTGGATCACCGAGCTCGCCGCGCCGCTCGAGTGCCTCAAGGCGCTCCACCTCCGACGCATGGTCGTCACAGACGACGACCTCGCCGAGCTCGTCCGTGCCAGGGGGCACATGCTGCAGGAGCTGAAGCTCGATAAGTGCACCGGCTTCTCCACTCATGGACTCCGCCTCGTTGCCCGCTCCTGCAGATCACTGAGGACTTTATTTTTGGAAGAATGTCAAATTGATGATAAGGGCAGTGAATGGATCCACGATCTCGCAGTCTGCTGTCCTGTTCTGACAACATTGAATTTCCACATGACTGAGCTTGAAGTGATGCCAGCTGACCTAAAGCTTCTTGCAAAGAGCTGCAAGTCACTGATTTCATTGAAGATTAGTGACTGCGATCTTTCAGATTTGATAGAGTTCTTCCAATTTGCCACAGCACTGGAAGAATTTGCTGGAGGGACATTCAATGAGCAAGGGGAACTCAGCAAGTATGTGAATGTTAAATTTCCATCAAGACTATGCTCCTTGGGACTTACTTACATGGGAACAAATGAAATGCCCATTATGTTCCCTTTTTCTGCAATACTAAAGAAGCTGGATTTGCAATACACTTTCCTCACCACTGAGGACCATTGCCAGCTCATTGCAAAATGCCCGAACTTACTAGTTCTCGCGGTGAGGAATGTGATTGGAGATAGAGGATTAGGAGTTGTTGCGGATACGTGCAAGAAGCTCCAAAGGCTCAGAATAGAGCGAGGAGATGATGAAGGAGGTGTGCAAGAAGAGCAGGGAGGGGTCTCTCAAGTGGGCTTGACGGCTATAGCCGTAGGTTGCCGTGAGCTGGAATATATAGCTGCCTATGTGTCTGATATAACCAATGGGGCCTTGGAATCTATCGGGACATTCTGCAAAAAACTATACGACTTCCGGCTTGTTCTACTTGATAGAGAAGAGAGGATAACAGACTTGCCACTGGACAATGGTGTCCGAGCTTTGTTGAGGGGCTGCACCAAGCTTCGGAGGTTTGCTCTGTACTTGAGACCAGGAGGGCTCTCAGATGCAGGTCTCGGCTACATTGGACAGTGCAGCGGAAACATCCAGTACATGCTTCTCGGTAATGTTGGGGAAACTGATGATGGATTGATCAGCTTCGCATTGGGTTGCGTAAACCTGCGAAAGCTTGAACTCAGGAGTTGCTGCTTCAGCGAGCGAGCACTGGCCCTTGCAATACTACATATGCCTTCCCTGAGGTACGTATGGGTTCAGGGCTACAAAGCGTCTCAAACCGGCCGAGACCTCATGCTCATGGCAAGGCCCTTCTGGAACATAGAGTTTACACCTCCCAATCCTAAGAACGGAGGTTGGCTGATGGAAGATGGGGAGCCTTGTGTAGATAGTCACGCTCAGATACTTGCATACCACTCCCTCGCCGGTAAGAGGCTGGACTGCCCACAATCCGTGGTTCCTTTGTATCCTGCGTGA

Genomic sequences:

>TaCOI1-A Traes_1AS_1E22B5174.1

ATGGAGGACCTACAGGAGGCTCTGATGACAGAGATTCTCAAGAGGATCACCACGATAAGTGATCTGAATTCTCTTTCCCTTGTGTCAAAGCAGCTCTGCAAGATAGAGGGGAATCAGAGGGGTGCTATCCGTGTTGGTTCCCGTCTTTGCACTGCTACAGAAGCACTGACATCATTGTGCGCCCGCTTCCCAAATCTGCGAAGAGTGGAAATCGATTACTTTGGTTGGATACCTGGACATGGAAGGCAGTTGGACAACAAAGGCTTTTCTGTGTTTTCATCTCACTATTCCTCACTGATTGACCTAACCTTAAGCTTCTGCTCATGCATCGATGACTCTGGGCTTGCTTGTTTAGCTTATTGCAAGACATTGGTGTCTCTCAGGCTCAACTCCGCACCAAAAATAACGTCAGTTGGGCTTTTCTCGGTTGCAGTTGGTTGCAGAAGTCTATCTGCTCTCCACCTTATTGATTGCGAGAAAATCGACACTGTAGAGTGGCTGGAATACCTTGGTAGGGATGGATCGTTGGAAGAGCTTGTAGTGAAGAATTGCCAAGGAATCAATCATCATGACTTTCTAAAGTTTGGTTCAGGATGGATGAAGCTCCAGAAGTTTGAGTTTGAGAGTAAAAGAGAAAGATATGATCGTCTTCCAGGTGATGTGGTCTATGACTCCTCGTACCATGCTCACAGCACGGATATATATGATTTCTGCTGTGAGAGTTTGAAGGATTTAAGGTTGGCTCATATTAAAACTTGGCCAGAAGTAGGTCTTCGTCTTGTCCTAGGGAAGTGTAAAGCATTGGAGAAGCTTTGCCTTGAGTATGTTCGTGCCCTAAATGACAATGACATTATTGCATTATCTCGGAGCTGCAGCAACCTTAAAAGCATCTCACTTTGGCTCAACCTGCAGCGCTACTCTAGTGATGTCAGCTATTGTGAGACGAGGACGTCATTTACTGATAACAGCCTTTACGCTCTAGCACGAAACTGTCGTATGCTTCAGATGGTAGACCTCAGCTTTATAGGATGTTCCCGTGACTGGCCATCAGAAATAGGATTCACACAAGAGGGTTTTCTGGTGCTCATTCAGTCCTGCCCGATTCGTGTTCTCGTGCTAAACACCGCCAACTTCTTTGATGACGAGGGGATGAAGGCCCTCTCATCCTCACCACATCTGGAGACACTCGAGCTTATATTGTGCCATGCGGTAACTGATGCTGGGATGCACTTCATTGCGCACACCCCATGCTTGAGTAATCTCACACTTCGGTTGTGTCATAACGTTACTGATGTGGGAGTGGCTGAACTGGGACATGCACATAAGTTAGAATCTTTGGTTGTCGAGTATTGTGGTGAGGTCTCTCTGCAAGCTGTGCAGGGTGTTGCCAAGTTAGTTCACTACAGTGACTATTCATATTTCTTTATGAAGAAAATTGGTCTTGGCGCCTATTGA

>TaCOI2-A Traes_2AL_9EC359B65.2

GTGAGGGCTGGTTTGGATGCGTGCATCTCCAGCCTCCTCCCAAGTCCTACTCCTACTACTGAAGAGAGAGAGAGAGAGAGAGAGAGGGAGAGCATTCCCCTCCTCCAACCTCCCCCCCAGCTGCCTCCCCTCCCAAAGCTCCCCACTTCTCCCGCTCCGGCGTCGCATTCGCCCCGCCCGCCCCGCCGATTCCCTCGGGTCTTATCCTCGCTTGCGGCCTGCCGACAGCGGGGCTCCCCTCCGCTCGGAGTCTCCCCGCGCCCCGCCGAATTTCTCGGGTCATTCTCTCCGGCGGCTCCGGGTGCGGGCTTTCGGTCCGGGGCGCCGCGGCCCGCGATCATTGCTGGAGGAGAGCTTTTTTCGCGGAGGGGTCTGGTGCGGAGTCTGCAGTACCGGCGGCGTCCATAGGTTAGATCTGGAGGTTTCGGCGCTCTGGATCTGAGCTGTTGGTAGGCTGCATTTTTTTTTTCGTTTCCTTCTCCCCGTCGCTATTTGTTTCCTTTTTCTTAGCAAGTTCCCGGAGGCGGCAAGAACTCAGCGGTCGCTGATCTTTCTTTGCAGGTTTTGGGTGGAGGAATCGGGACTGGCGGTCGCAATCCTCCTAGCCATTTTGGTGCTGTCCGTTCTGGGTTACTAGGGGGTTTGCGTGTTCTAAATAGGGTATGGACGGCAGATGCAGTAACTACTAGTATCTCTTGAAGAAACTTCAGCTGCAGATCTGAAACCCATTTTTGTGCCCCTTGCTGTAGTTGTAGCCCTGCCAGATGAGGTGAACCAGGCCGCTTTCTAGTGCCCCCTTGCCATTGCTTGTTGCCAGTGTTGGTTGCCAGTTTTAGCTCAACAAATCTGGCTGGAGCCTGATCTGCCCAAGCCAGCATTAGTTGTGTGCTCTCTGTGTCTGGTTGAGCAAGGTTGCAGCTTCCATTTGTTGATTTCGTCGGCTTTGTTTAGTAGCTTTGATAACCTGGAGACTGCAAGCAAACAGATCCCGTCCTGCAGCTCTAGTAATAAGGTGGTGCTGGGGGAAGGCACCATGACCTACTTTCCGGAGGAGGTGGTGGAGCACATATTCAGCTTCTTGCCCGCGCAATGTGACCGGAACACCGTTTCACTTGTTTGCAAGGTATGGTATGAGATTGAAAGGCTGAGCCGGCGAACTGTCTTTGTGGGTAACTGCTATGCTGTGCGCCCTGAGCGCGTGGTGCTTCGGTTCCCCAATGTGCGGGCACTGACAGTGAAGGGGAAACCACACTTCGCTGATTTCAACCTTGTGCCACCTGATTGGGGTGGGTACGCCGGACCATGGATCGAGGCAGCAGCCAGGGGCTGCGTGGGTCTTGAGGAGCTGCGGATGAAGCGGATGGTGGTGTCAGATGAGAGCCTGGAGCTGCTTGCCAAATCATTCCCACGATTCAGGGCCCTAGTTCTTATCAGCTGTGAGGGGTTCAGCACCGATGGACTAGCAGCTATTGCAAGTCACTGCAAGTGAGTGTACTTAAAATTGCAGTTTGTTAAGGCATCATAGTTTTCTGATTTGCTGTTTTAGTTTCTGCGTGCATCTAGATCTTATGGTTTAACTTGGTGAGGTATTGAAATATGAAATGCCTTTCTTGCTCTTTTCTGATTAGCTACTAGCCTTCTTGCTTCGGTCCTGCCAGTTATTACTTGTTTGAAGCCTAGATCCAGCACTTGTTCCAATGCTATGTGCAAGTAAAGTTGAAAGGATCAAATTTGATCTGCTGCACAATTCTGTAGCTACAAAATCTCCCGAATTGCATGAGCACTCTTTCAGGGTCTGAATGATCCAGTTGCATCTGATGTCCTTAGTTAGTTATCCTGATGTTCTGTCCAAATTTTGTACCTGCCATTTTTTGACGGACCATTCTTGTGTTAGATTCGTGTATAGTATGTCTGTTATCTTTTTATTTAGCTCGTTAGACTTTACATTTTATCAGTTAATTTTGTCTATACTTCCTTTTTGGTAGGATGAATTGGTATTGTTATGCTCATGGTGCACACAATCTGCTAGGGCAGGATGCCAGTTCTGATTAATTTCATTAATGCATTATTATTATCTCATTAAAGGCACCAATCTACTCTTGTTACATGCATTTAATGGTCAATACACTGACATTGTTTTTTATATTGTTATCTGGTCTAGGCTCCTGAGGGAGTTAGATTTGCAGGAAAATGAGGTGGATGATCGAGGGCCAAGGTGGCTCTCCTGCTTCCCTGATTCCTGCACGTCCCTTGTCTCCTTGAATTTCGCCTGCATCAAAGGGGAGGTTAATGCTGGTTCATTAGAGAGACTTGTTGCTAGGTCCCCAAGTCTTCGGAGTTTGAGGTTGAATCGATCTGTGTCAGTAGATACACTCTCGAAGATATTAATGAGCGCCCCTAATTTGGAGGATCTAGGGACTGGGAACTTGACAGATGACTTCCAAGCTGAATCGTATCTCAGGCTGACCCTTGCATTGGAGAAATGCAAACTGCTGAGGAGTTTATCGGGCTTTTGGGATGCTTCGCCTTTGTGCCTTCCATTCATCTATCCTGTATGTGGGCAACTAACAGGTTTAAACTTGAGCTATGCTCCGACACTTGATTCTTCTGATCTCACCAAAATGATCAGCCACTGTGTGAAACTCCAACGTCTTTGGGTGAGTTCCCTTGCTGCCGCACTCACCTTACTATTTTTATGCTTGATTAAGACAATTCAGTACAGGGTTTGAGCCTTTATCCTGTACACTCCAGGTACTGGATTGCATCGCGGATAAGGGCTTGCAAGTGGTGGCCTCCAGTTGCAAGGATCTACAAGAACTCAGGGTATTCCCATCAGACTTCTATATCGCCGGGTATTCCCCAGTAACAGAGGAGGGACTTGTTGCAATATCCTTGGGCTGTCAAAAACTGAGCTCATTGCTATATTTTTGTCATCAAATGACGAATGCTGCACTGCTTACTATAGCTAAGAACTGCCCAAATTTCACGCGGTTCAGACTCTGTATTCTTGAGCCTGGGAAGCCTGATGCCATGACAAACCAACCATTAGATGAAGGTTTTGGTGCTATTGTTCGTGAATGCAAAGGGCTAAGGCGATTGTCAATATCGGGTCTTCTCACCGACAAGGTTTTCATGTATATTGGTAAATTCGCGAAACAACTTGAGATGCTTTCAATAGCATTTGCTGGAGATAGTGATGCGGGAATGATGCATGTTATGGAAGGATGCAATAATCTGAGGAAGCTGGAGATTAGAGATAGCCCATTTGGTGATGCTGCACTCCTGGAGAATGTTACCAAGTATGAGACAATGCGATCCCTTTGGATGTCATCATGCAATGTCACAGAAAAGGGGTGCCAAATCCTTGCATCAAAGATGCCAATGCTTAATGTGGAGGTTATAAATGAGGTAGATGAGAGCAATGAAATGGATGAGAACCATGGAATCCCCAAAGTTGACAAGTTATATGTTTACCGCACAACTGCTGGGGCAAGGGATGATGCGCCAAATTTTGTTAAAATCCTATAGAGCTAGTTGAGAATTGCCTGGTATCTCCTTGCTGTATAAGGTAATCCTTCTCAAATTATCTACTTTCATTCAGAAGGTATCTTGTTTGTTTGAAGAATCTAAACTTTGAAGTTGCATTCTCTTGTTTCTCCGCAGCTCGTATGTTCCTCTAAATATGTTGTGCATGCAACACGCAACTTGCAAATGATTTTTCAAAAGATGAAAATATAGTACATAGTTGAAAATGATGTCGAACCAACTTTATTCGTCCAAATCTCCCATCTACTTCCTCACTAGATTATAGCTGCTCAATCATAGTGCCGACGCATAGAATGCTGCTGATATATCGCATGATATGATCCCCATTCCATCTGACCTCCCTATACTGGACTGCTTTTACAGATGGTCGGCGGAATGGAATCCGGCGGGGGTCTGTGGACAGTGGCAAGTCTCCCGCTGCCCCCCTCGGCTTTGCAGCGCCAGGAAAAGCCACGTCAGTGTGCTCCTTATCCTGGAGCATCTCTGTCCAGGGATCCATCATTCCCTTTTTAGCGTCTGGTCATATGGCCACTGCCTTTATCCTGTGCATTTTTCTTTTTCTTAAGAGGACCACCAACCAACCAATCCTTTCTTTTCCTTTTTCCTCTACTATGCTGTACTCCGTAGCCATTTGCTTTTGACATTGCCACAGTGGAATGTGCCTCAGCTGTCGCTGTTGCTCTTGTTGTAAACGTTTGTTTGAACCTGACCGAAACGACGATGGCCAATGATCTTGCTCCCGTGAAATCATGCAGCAGAGGCTGGAATAATTTGTTGCTCATCAGCCCATTACTTCATTTTCGGCACTTGTTACCGTCCCTTTTTTTTTAGCTAATGACATTGTGTG

>TaCOI2-B Traes_2BL_370AE211F.1

GGATGCGTGCATCTCCAGCCTCCTCCCAAGTCCTACTCCTACTACTGAACAGAGAGAGGGACAGAGCATTCCCCTCCTCCAACCTCCCCCCCAGCTGCCTCCCCTCCCAAAGCTCCCCACTTCTCTCGCTCCGGCGTCGCATTCGCCCCGGCCGCCCCGCCGATTCCCTCGGGTCTTATCCTCGCTCGCGGCCTGCCGTCAGCGGGGCTCCCCTCCGCTCGGAGTCTCCCCCGCGCCCCGCCGAATTTCTTGGGTCATTCTCTCCGGCGGCTCCGGGTGCGGGCTTTCGGTCCGGGGCGCCGCGGCCCACGATCATTGCTGGAGGAGAGCTTTTTCCGCGGATGGGTTTGGTTCGGAGTCTGGAGTACCTGCGGCGTCCATAGGTTAGATCTGGAGGTTTCGGCGCTCTGGATCTGAGCTGTTGGTAGGCTGCATTTTTATTTTCGTTTCCTTCTCCCCGTCGCTATTTGTTTCCTTTTTCTTAGCAAGTTCCCGGAGACGGCAAGAACTCAGCGGTCGCTGATCTTTCTTTGCAGGTTTCGGGTGGAGGAGTCGGGACTGGCGGTCGCAATCCTCCCAACCATTTTGGTGCTGTCCGTTCTGGGTTACTAGGGGGTTTGCGTGTTCTAAATAGGGCATGGATGGCAGATGCAGTAACTACTAGTATCTCTTGAAGAAACTTCAGCTGCAGATCTGAAACCCATTTGTGCCCCTTGCTCTAGTTGCAGCCCTGCCAGATGAGGTGAACCAGGCCGCTTTCCAGTGTCCCCTTGCCATTGCTTGTTGCCAGTGTTGGTTGCCAGTTTTGGCTCAACAAATCTGGGTGGAGCCTGATCTGTCCAAGCCAGCATTAGTTGTGTGCTCTCTGCGTCTGGTTGAGCAAGGTTGCAGCTAATTTTGTTGATTTCGTTGGCTTTGTTTAGTAGCTTTGATAACCTGGAGACTGCAAGCAAACAGATCCCGTCCTGCAGCTCTAGTAATAAGGTGGTGCTGGGGGAAGGCACCATGACCTACTTTCCCGAGGAGGTGGTGGAGCACATATTCAGCTTCTTGCCTGCGCAGTGTGACCGGAACACGGTTTCACTTGTTTGCAAGGTATGGTATGAGATTGAAAGGCTGAGCCGGCGAACTGTCTTTGTGGGTAACTGCTATGCTGTGCGCCCTGAGCGTGTGGTGCTTCGGTTCCCCAATGTGCGGGCACTGACAGTGAAGGGGAAACCACACTTCGCTGATTTCAACCTTGTGCCACCTGATTGGGGTGGGTATGCCGGACCATGGATCGAGGCAGCAGCCAGGCGCTGCGTGGGTCTTGAGGAGCTGCGGATGAAGCGGATGGTGGTGTCAGATGAGAGCCTGGAGCTGCTTGCCAAATCATTCCCACGATTCAGGGCCCTAGTTCTTATCAGCTGTGAGGGGTTCAGCACCGATGGACTAGCAGCTATTGCAAGTCACTGCAAGTGAGTGTACTTAAAATTGCAGTTTGTTAAGGCATCATAGTTTTCTGATATGCTGTTGTAGTTTCTGCGTGCATCTAGATCTTATGGTTTAAAATGGTGAGGTATTGAAATATGAAGTGCCTTTCTTGCTCTTTTCTGATTAGCTACTAGCCTTCTTGCTTCGGTGCTGCCAGTTATTACTCGTTTGAAGCCTAGATCCAGCACTTGTTCCAATGCTATGTGCAAGTAAAGTTGAAAGGATCAAATTTGATCTGCTGCACAATTCTGTAGCTACAAACTCTCCCGAATTGCATGAGTACTCTTTCAGGGTTTGAATGATCCAGTTGCATCTGATGTCCTGAGTTAGTTATCCTGATGTTCTATCCTAATTTTGTACCTGCCATTTTTTGACGGACCATTCTTGTGTTAGATTCATGTATAGTATGTCTGTTATGTTTTTATTTAGCTTGTTAGACTTTACATTTTATCAGTTAATTTTGTCTATACTTCCTTTTTGGTAGGATGAATTGGTATTGTTATGCTCATGGTGCACACAATCTGCTAGGGCAGGATGCAAGTTCTGATTAATTTCATTAATGCATTATTATTATCTCATTAAAGGCACCAATCTACTCTTGTTACGTGCATTTAATGGTCAATACACTGACATTGTTTTTTATATTGTTATCTGGTCTAGACTCTTGAGGGAGTTAGATTTGCAGGAAAATGAGGTGGATGATCGAGGGCCAAGGTGGCTCTCCTGCTTCCCTGATTCCTGCACGTCCCTTGTCTCCTTGAATTTCGCCTGCATCAAAGGGGAGGTTAATGCTGGTTCATTAGAGAGACTTGTTGCTAGGTCCCCAAGTCTTCGGAGTTTGAGGTTGAATCGATCTGTGTCAGTAGATACACTCTCGAAGATATTAATGCGCGCCCCTAATTTGGAGGATCTAGGGACTGGGAACTTGACAGATGACTTCCAAGCTGAATCGTATCTCAGGCTGACCCTTGCATTGGAGAAATGCAAACTGCTGAGGAGTTTGTCGGGCTTTTGGGATGCCTCCCCTTTGTGCCTTCCATTCATCTATCCTGTATGTGGGCAACTAACAGGTTTAAATTTGAGCTATGCTCCGACACTTGATTCTTCCGATCTCACCAAAATGATCAGCCACTGTGTGAAACTCCAACGTCTTTGGGTGAGATCCCTTGCTGCCACACCCACCTTACTATCTTTATGCTTTATTAAGACAATTCAGTACAGGGTTTGAGCCTTTATCCTGTACACTGCAGGTACTGGATTGCATCGCGGATAAGGGCTTGCAAGTGGTGGCCTCCAGTTGCAAGGATCTACAAGAACTCAGGGTATTCCCGTCAGACTTCTATATCGCCGGGTATTCCCCAGTAACAGAGGAGGGACTTGTTGCAATATCCTTGGGCTGTCAAAAACTGAGCTCATTGCTATATTTTTGTCATCAAATGACGAATGCCGCACTGCTTACTATAGCTAAGAACCGCCCAAATTTCACGCGATTCAGACTCTGTATCCTTGAGCCTGGGAAGCCTGATGCCATGACAAACCAACCATTAGATGAAGGTTTTGGTGCTATTGTTCGTGAATGCAAAGGGCTAAGGCGATTGTCAATATCGGGTCTTCTCACCGACAAGGTTTTCATGTATATTGGTAAATTCGCGAAACAACTTGAGATGCTTTCAATAGCATTTGCTGGAGATAGTGATGCGGGAATGATGCATGTTATGGAAGGATGCAATAATTTGAGGAAGCTGGAGATTAGAGATAGCCCATTTGGTGATGCTGCACTCTTGGAGAATGTTACCAAGTATGAGACAATGCGATCCCTTTGGATGTCATCGTGCAATGTCACAGAAAAAGGGTGCCAAATCCTTGCATCAAAGATGCCAATGCTTAATGTGGAGGTTATAAATGAGGTGGATGAGAGCAATGAAATGGACGAGAACCATGGAATCCCCAAAGTTGACAAGTTATATGTTTACCGCACAACTGCTGGGGCAAGGGATGATGCGCCAAATTTTGTTAAAATCCTATAGAGCTAGTTGAGAATTGCCTGGTATCTCCTTGCTGTAAAAGGTAACCCTTCTCAAATTATCTACTTTCATTTAAAAAATATCTTGTTTGTTTGAAGAATCTAAAATTTGAAGTTGCATTCTTTTGTTTCTCCTCAGCTCATATGATCCTCTAAATATGTTGCGCATGCAACACACAACTTGCAAATGATTTTTCAAAAGATGAAAATATAGTACATAGTTGAAAATGATGTCAAACCAACTTTATTCGTCCAAATCTCTATCTACTTGCTCACTAGATTGTAGCTACTCAATCATAGTGCCGATTGCATGATATGATCCCCATTTCATCTGACCTCTCCCTATACTGGACTGCTTTTACAGATGGTCGGCGGAATGGAATCCGGCGGGGGCCTGTGGACAGTGGCAAGTCTCCCGCTGCCCCCCTCGGCTTTGCAGCGCCAGGAGAAGCCACGTCAGTGTGCTCCTTATCCTGGAGCATCTCTGTCCGGCGATCCATCATTCCCTTTTTAGCGGCTGGTCATATGGCCACTGCCTTTATCCTGTGCATTTTTCTTTTTCTTAAGAGGACCACCCACCAACCAATCCTTCCTTTTCCTTTTTCCTGTACTATGCTGTACTCCGTAGCCATTTGCTTTTGACATTGCCACAGTGGAATGTGACTGCTGTCGCTGTTGCTCTTGTTGTAAACGTTTGTTTGAACCTGACCGAAAGGACGATGGCCAATGATCTTGCTCCCGTGAAATCATGCAGCAGAGGCTGGAATAATTTGTTGCTCATCAGCACATTACTTCATTTTCGGCACTTGTTACCGCCCCTTGTGTTTTTTAGCTAATGATATTGTGTGCGTCCGG

>TaCOI3-B TRAES3BF018800040CFD_t1

GCGCAAGTGAAATCGAATCAATAATCAATAATCGTGTGAAAATGGCGTACGTGATCGTCGGCGGCGGCACGCTGATGAGCGTTGAGCCGCGGCACGTACAGTGCTAGGGAGCAGAGCGGAGCTGCCCGTACTCGTAGACTGGTACCGTGCTGGCTGTACCCGGTGGGCGAGGCCATTTATCGCTCTCGTCCACCGGCGGGGCGTACGTGGCGCGGCACCACCAGACGGGACGGGACGAGCCGCGCAGGTAAAAGCTGCTGCCTCCCCATGTCACGTCCGCCCCTGCTCCTATCCCTCCCTTCTCCCTCCAATAATTTCCTTCCCTTCACTTCTCCCGTCCTCCCGCAAAGCCCACCCCCGTAGCAGAAAGGGAGGGAGGGAGGAGGAATCTCCGTCTCCACCTCCACCTCCATGCCCCCGCCCCCCGCCGGGCCCGGCCCAGATCTCCCGCGNNNNNNNNNNNNNNNNNNNNNNNNNNNGATCCGGCCCGATGGGCGGGGAGGTGCCGGAGCCGCGGCGGCTCAGCCGCGCGCTCAGCTTCGGCGTGCCCGACGAGGCGCTGCACCTCGTCATGGGCTACGTCGACGCCCCGCGCGACCGGGAGGCCGCCTCGCTCGTCTGCCGCCGCTGGCACCGCATCGACGCGCTCACCCGCAAGCACGTCACCGTCGCCTTCTGCTACGCCGCCGACCCCTCGCGCCTCCTCGCCCGCTTCCCGCGCCTCGAGTCGCTGGCCCTCAAGGGCAGGCCGCGCGCCGCCATGTACGGCCTCATCTCCGACGACTGGGGCGCCTACGCCGCGCCCTGGGTCGCACGGCTCGCCGCGCCGCTCGAGTGCCTCAAGGCGCTCCACCTGCGACGCATGACCGTCACCGACGACGACGTCGCCACGCTCATCCGCTCCCGCGGCCACATGCTGCAGGAGCTCAAGCTCGACAAGTGCTCCGGCTTCTCCACCGACGCGCTCCGCCTCGTCGCCCGCTCCTGCAGGTAGTCCAGCAGCTCTACTCACCTGCGTCCGTGCACCTCTTTTTTTTTTTGCCCTCTTGCCTGTTGATTTGTGGTGGTGAGTTCCAGTAATTGTGACGGTTGATCTGCTCATTGATGAATGAGGATTGATCTGGTCTGTGTGTTGGTTGGTTTCGCCAAATGGAGTATATACTGGATAAGGATGAGTTAAGTGCTAGTTTCTATAATTAGATATTTGTTTGCTCTATCATGAGCTGTAACAAATACGGTCATGTGAGGGAAGGTGTCAAGTTAGCTTCCACACACTTTGTTTGGGCGCCATGATACATCATGGCACTAGTTTTCTACGACAAATTGATATGAGCAGTATAAGCAAATGACTGCCGAAGGGAGATATATGAGATTTGGTGTAGTTCTATGGTTTACCTGTCGTTTCTGTCCAGCACATGACCGATCCGGTCTCTAGTTTGTAATATTGACTCACAACAGCTCACAAGTCCCAACCAAGTCAGGCTTAAGCAAACTATAATTTGAGGTTGCACCAGTCAGGTGATCTATCTTCCCCAGGAGTGCTATCGTACTACTTTGATTGGACTTGAACAAGACTACATCCGCCCCTATGGGACGTATTTTGAACCAAAATAATGTTGCAAGTTGGTGGTTTCTGTTACCAGTCAATTCTATCATGTTCAGATTAGTGTGGCACTATGTCATACCGTCATATCGATTTTCGAGCATCATCTTGTCCTTCCTTTTTACTCTTTTGGCAGAAGCCTTTCCATGTGTCCGTGTTAACTTTGCTTTGTCTGAATTCTGCAACTACGAAGTTGTGTGAGGGAAATTATTTTCCCTTAGAATGCAAGTATGATTTGTGTGAGCTTGTCTAGTTCACATATGGAGCTGGGAGACTAAAGGTTAGCATGCTACAAAGGATCTGTAGTGCTTTAGGTAAAAATGCATGTACAGTGTCCTCCTTTGTGCCTGGAAATGCCTCGTATGCAACCATGAATTGAAAAAAAAACTGACTTGACTTCAATGGCTGTTCCGCTGCCCCACTATTAATGAAAGGGGACAGAACATGGGGACATCGGGGCATGTTCATGAAGTTTGAGACGTGTCTTTATAGGTTTCCTGGTTCACCAGTAATCCACTTTTCATCTCACTTTCCATTCTTCCTTAGCTTACAACTATTTTTGTGCTCCTTTAGCAAGACATTCGACTGTGGTACATTAATTCAAAGTGATTTTACTGTCACTGCATGATTGCGTGCTATCACTGCAGTAACATGCTATTCTAAATTATCTTGGCAGATCCTTAAGAACATTATTTCTTGAAGAATGCGTGATTACTGACGAAGGTGGTGAATGGCTTCATGAACTTGCTGTCAACAATTCTGTTCTTGTGACACTGAACTTCTACATGACTGAGCTCAAAGTGGTGCCGGCTGATCTGGAGCTTCTAGCAAAGAACTGCAAATCATTACTTTCTTTAAAGATCAGTGAGTGTGACCTTTCAGACCTGATTGGTTTTTTCGAAGCAGCCAATGCATTGCAAGATTTTGCTGGAGGATCGTTCAATGAGGTAGGAGAGCTAACAAAGTATGAAAAAGTCAAGTTTCCACCAAGAGTATGCTTCTTGGGGCTTACGTTCATGGGGAAAAATGAGATGCCTGTTATCTTCCCCTTTTCTGCTTCATTAAAGAAGCTGGACTTGCAGTACACTTTCCTCAGCACTGAGGATCATTGCCAGCTTATCTCAAAATGCCCGAACCTATTTGTTCTTGAGGTAATGTTTCTCGTACTCATGTATTTTGGGGGTGTGAAATTTGTCGTACTCTCATTTTTACTGACAGTAGATTCTGTCAACAGGTGAGGAATGTGATAGGAGACAGAGGGCTGGAGGTTGTCGGCGATACATGCAAGAAGCTACGAAGACTTCGAATTGAGCGAGGGGATGATGATCCAGGTCTACAAGAAGAGCAAGGAGGAGTTTCTCAGTTAGGCCTGACAGCGGTAGCTGTTGGTTGCCGAGACCTGGAGTACATAGCTGCCTATGTATCTGATATCACCAACGGTGCTCTTGAATCCATTGGGACCTTCTGCAAAAATCTCTACGACTTCCGGCTTGTCCTGCTCGACAGACAAAAGCAGGTAACTGATCTGCCACTCGACAACGGTGTTCGTGCTCTGTTAAGGAGTTGCACCAAGCTCCGGAGATTTGCTCTCTACCTGAGACCTGGAGGGCTCTCAGACATAGGCCTCGACTACATCGGGCAGTACAGCGGCAACATTCAGTACATGCTGCTGGGCAACGTCGGTGAATCTGACCACGGGTTGATCCGCTTTGCGATAGGATGCATCAACCTGCGGAAGCTTGAGCTTCGGAGCTGCTGCTTCAGCGAGCAAGCCCTGTCCCTCGCGGTGCTCCACATGCCCTCGCTCAGGTACATATGGGTGCAAGGCTACAAAGCCTCTCCAGCAGGCCTCGAGCTCCTGCTCATGGCGAGGCGATTCTGGAACATCGAGTTCACGCCCCCCAGCCCCGAGGGCTTGTTCCGCATGACGCTTGAAGGAGAACCCTGCGTGGATAAGCAGGCCCAGGTTCTTGCCTACTACTCCCTTGCTGGGCAGAGGCAGGACTGCCCTGACTGGGTGACCCCGTTGCATCCAGCTGCATGATTGATTGTAAATACAGTGTACTACATCAAGTTGTGTGTACGTAGGTACTCTTACCTTATTGCCCCTCGTCCCTTGGGCAACGATCGTGTCCGAATATGGTAGTAATTTGTATGGATGTAGATCATTAGCTAGCTGCTTTGGTGCCCTAATAAGCTAGTGCTACTGTAGTGCTGTAGCTGAGGTGTAGTGCAATAAGTTGCTGTTGTCGCTTGTACTACTATGTATGTAATCCTGGGAAGTTGTATGCTAAAGTTGCTCCGTGCTTTGATCCTGAGAGTTGAGACTTATCTGAAATCTTGGTCGGTTGCTG

>TaCOI3-D Traes_3DL_845220DFC.1

TTTTATTTTCTCTTTTTTTTGCCCTCTTGCCTGTTGATTTGTGGTGGCGAGTTCCAGTAATTGTGACGGTTGATCTGCTCATTGATGAATGAGGATTGATCTGGTCTGTGTGTTGGTTGGTTTCGCCAAATGGAGTATACTGGATAAGGATGAGTTAAGTGCCAGTTTCTATAATTAGATATTTGTTTGCTCTATCATGAGCTGTAACAAATACGGTCATGTGAGGGAAGGTGTCAAGTTAGCTTCCACACACTTTGTTTGGGCGCCATGATACATCATGGCACTAGTTTTCTACGACAAATTGATATGAGCAGTATATGCAAATGACTGCCGAAGGGAGATATATGAGATTTGGTGTACTTCTATGGTTTACCTGTCGTTCCTGTCCAGCACATGTCCGATCCGGTCTCTAGTTTGTAATAAGTATTGACTCACAACAGCTCACAAGTCCCAACCAAGTCAGGCTTAAGCAAACTATAATTTGAGGTTGCATCAGTCATGTGATCTATCTTCCCCAGGAGTGCTATCGTACTACTTTGATTGGACTTGAACAAGACTACATCCGCCCCTATGGGACATATTTTGAACCAAAATAATGTTGCAAGTTGGTGGTTTCTGTTACCAGTCAATTCTATCATGTTCAGATTAGTGTGGCACTATGTCATACCGTCTTGTCCTTCCTTTTTACTCTTTTGGCAGAAGCCTTTCCATGTGTCCGTGTTAACTTTGCTTTGTCTGAATTCTGCAACTACGAAGTTGTGTGAGGGAAATTATTTTCCCTTAGAATGCAAGTATGATTTGTGTGAGCTTGTCAAGTTCACATATGGAGCTGGGAGACTAAAGGTTAGCATGCTACAAAGGATCTGTAGTGCTTTAGGTAAAATGCATGTACAGTGTGCTCCTTTGTGCCTGGAAATGCCTCATATGCAACCGTGAATTGAAAAAAAAACTGACTTGACTTCAATGGCTGTTCCGCTGTCCCACTATTAATGAAAGGGGACAGAACATGGGGACATCGGGGCATGTTCATGAAGTTTGAGACGTGTCTTTATAGGTTTCCTGGTTCACCAGTAATCCACTTTTCATCGCACTTTCCATTCTTCCTTAGCTTACAACTATTTTTGTGCTCCTTTAGCAAGGCATTCGGACTGTGGTACATTAATTCAAAGTGATTTTACTGTCACTGCATGATTGCGTGCTATCACTGCAGTAACATGCTATTCTAAATTATCTTGGCAGATCCTTGAGAACATTATTTCTTGAAGAATGCGTGATTACTGGTGAAGGTGGTGAATGGCTTCATGAACTTGCTGTCAACAATTCTGTTCTCGTGACACTGAACTTCTACATGACTGAGCTCAAAGTGGTGCCGGCTGATCTGGAGCTTCTAGCAAAGAACTGCAAATCATTACTTTCTTTAAAGATCAGTGAGTGTGACCTTTCAGACCTGATTGGTTTTTTCGAAGCAGCCAATGCATTGCAAGATTTTGCTGGAGGATCGTTCAATGAGGTAGGAGAGCTAACAAAGTATGAAAAAGTCAAGTTTCCACCAAGAATATGCTTCTTGGGGCTTACGTTCATGGGGAAAAATGAGATGCCTGTTATCTTCCCCTTCTCTGCTTCATTAAAGAAACTGGACTTGCAGTACACTTTCCTCACCACTGAGGATCATTGCCAGCTTATCTCAAAATGTCCAAACCTATTTGTTCTCGAGGTAATGTTTCTCATATACTCTTGTATTTTGGGGGTGTGAAATTTGTCATACTCTCATTTTTACTGACAGTAGATTCTGTCAACAGGTGAGGAATGTGATAGGAGATAGAGGGCTAGAGGTTGTCGGCGATACATGCAAGAAGCTACGAAGACTTCGAATTGAGCGAGGGGATGATGATCCAGGTCTACAAGAAGAGCAAGGAGGAGTTTCTCAGTTAGGCCTGACAGCGGTAGCTGTTGGTTGCCGTGACCTGGAGTACATAGCTGCCTATGT

>TaCOI4-A Traes_3AL_2240B0C5F.1

TGTCCACCTTGATTCTAGAAAGAACAAAAAAAAAATGTTGTAGTTATACAGCAATTAAAACTGCTCAATCAGATGATATATTTGTCCCAAGAAACTTTAATTTCTTAGTTCGAAATAAATAAATGGTTTGTTCAGGGCCTTATGTGACAACTACAAAATCTTGTCTTATTAGCAATGTTTGAAGTGCAGTCCAGATATTGATTGACCAAAGTGTCATTTTCAGGTGCCAGGACCACATGAATGAGTTGGTGATATCACTGGCCCACAAATTTACAAAGCTTCAAGTCCTTTCTCTAAGGCAAATCAAGCCTCAGCTTGAAGACAGTGCAGTGGAGGCTGTAGCAAATTCTTGTCATGATTTGCGCGAGTTGGATCTGAGCAGAAGCTTCAGGCTTAGTGATCGGTCCTTGTATGCTCTGGCACATGGGTGCCCTCACCTTACAAGGCTGAACATCAGTGGATGTTCCAATTTCAGTGATGCTGCTTTGATCTACCTCACTAGTCAGTGCAAGAACTTGAAATGCTTGAATCTGTGCGGGTGTGTGCGGGCAGCATCCGACAGAGCATTGCAGGTACTAAAGCTGCGACAGCATATTTTGCATGAACCTTTATTTAATCTCTATTATCTTGGTGCAGGCCATAGCCCGCAACTGTAGTCAGCTGCAATCTTTGAACCTTGGTTGGTGCGATACTGTCACTGACGGGGGAGTCACTAGCTTGGCATCAGGATGTCCTGAACTTAGGGCCGTCGACTTGTGTGGCTGTGTTCTTATAACAGGTAATTTGTTTGATCGATCTTATGTTCCCAGAGTCTGCACAATCTGGTGCTCTGTTGATTGCTAAAACAAAATATACAAGGATATTAGCACTTCGTTTCTCTGCTCTGCTAAGGTAGTTTAACTATTTAGTTAATATCAGACATGCTGACAGACACAAACAATATCAGAACTGAGACTGGCTTTCTGCCAAGTCTTATGCTTTGCAATCATATTGTAATTTTAGGGCAAGGCATTTGCAACTCGCATTATTTATTGGAATGCACAGTCTCAAACATTACATTACACGCATCCTTGGACTTTTTTAGTTTGCTATATATGTCTTCTTACATAATGTGCAGTCTTCCTGATTTTGTTAAGTTGCTTCTGCATCCTTTACCCTTGTACACTGTTCTCTGAGGTGCAACCTTCATACTTATCCAAGATCAAGTCAAGTGTGCTCATCCAAATTTGTTTCGGTTGTTGATGATGTTCACCATGTACGGCATTGTTATCTAAGTTCAGCATCGTTTACAGTGCTGTAGTTGCTTGTCAGTCGCATGCAGAGCCATGCTCATTGCCTGAAATCTGACTCCTGTCCGTTTCTCCTTCTGCAGATGAGAGCGTGGTTGCTCTTGCAAACGGCTGCCCACACCTGCGTTCCCTGGGGCTGTACTACTGCCAGAACATCACCGACCGGGCCATGTACTCGCT

>TaCOI4-B TRAES3BF021600080CFD_t1

CTTGCTTGCCGTTTCCATTTTATTTTGGCTTTGTGCTTTCCACGGCTGCAGACCCCAAAGCTCCTTTTGAGAAGGTCTGCGTTGTGCAGAGTTCACTCTCCCGGGATTTTTGCGCAAGTTCCTCGATTGGAAGTTGCTAGTTGCCACCCGCAAATATATTTGATTCTAGGTGATCTCATAGATTGGATTGTTGATACAGAGGTGCTGATCCTAAGGCTGTTCATTCTTACGCCACTTCTAGCTGACTTTTGGTTCCTTCTGAACGCTTTGATTGACTTGAGAGATTAGTGCAGTAGTCGTTGCTTCTTGAAGAATTCTTTTCTCGAACACGCATCTAAATGCGTGACATTTTGTATTACAAGGAACTCTGCCGTGGAAGAGATCTCCATGGTTAACAACGGCTTATTGGCATAAGCCAACCAAAAAAATTAAAGCACAGGGCAAGAAAACGAAACAAAGCAAAAGCAGAAAAGAGAAATCAAGTACAGGGCAAGAAACGAAACAAAGCAAAGAGACAAAGGGTGGCACTACTTATCGAGGCAATAGCGTCTAGGTTCTCGATTGTATTATTGCTCCATTCCTCTGTTTGTGTTCCCTACCTAATTTTGCGGGTATTTATCTTCCGTTCACTAGATGTGACGTGTTTAAGTAAAAGTAGTAAGAACCCTACCTTGATCAAATAATTGCTAGATCTCGGTGAGGGCTTCTGACAGCGACATTCCAGTATGGTCTAGCGCATTCCTGGGAGGTTACTTGGGTGCATTTACGTTTTTGTTGCGGATACTTATTACTAATCTAACCTATTGACTCTGTTTATATAAGTTTGCTAATAGCTGCACTACTGAATGCAGGTTTATCTGTGAAAATGGTCAATGCACAGATGGTGAGTGGGTACTTGGACAATTCGTTCAATGCACTCGTGGTTTCTGGTGGCGGTGAGAGTGGTGGACAGACACAGAATGGTGTCACAGACACCACCTTGTCAGGTTGGAAGGACCTCCCCATGGAGCTTCTGCTGAGGATCATATCAGTCGCTGGAGATGACAGGATGGCCATTGTAGCCTCCGGTGTTTGCACCGGGTGGCGTGATGCACTAGGATGGGGAGCCACTAGTCTCTCCTTCTCGTGGTGAGTGATCTTCAGTTCTGCATTTCTTTCTTATGTGAATATGACCAATATTAGATTCTTCAGTTAACAAGATGGTTCAAATTATAATTATGAAATGTCACTTGCAGGCTGTAGCACAACTTACCACTGGGCTTGTAGTTTAGATCATTTTGTATATTATATAATTCAATTGTATGATTTATTGGAAGTCCACTGCAAATGTGAAAAAAAAATTGTCAATCAATTTGGTTCTTCAGCTGTCCAAAAACATAAAATGTTGTAGTTATACAACCATTAAAACTGCTCAATCAGATGATATATTTGCCCCAAGAAACTTTAATTTCTTAGTTCGAAATAAATAAATTGTTTGTTCAGGGCCTTATGTGACGACTACTTGTCTTATTAGCAATGTTTGAAGTGCAGTCCAGATGAAAGGTTATTGATTGACCAAAGTGTCATTTTCAGGTGCCAGGACCACATGAATGAGTTGGTGATATCACTCGCCCACAAATTTACAAAGCTTCAAGTCCTTTCTCTAAGGCAAATCAAGCCTCAGCTTGAAGACAGTGCAGTGGAGGCTGTAGCAAATTCTTGTCATGATTTGCGCGAGTTGGATCTGAGCAGAAGCTTCAGGCTTAGCGATCGGTCCTTGTATGCTCTGGCACATGGGTGCCCTCGCCTTACAAGGCTGAACATCAGTGGATGTTCCAATTTCAGTGATGCTGCTTTGATCTACCTCACTAGTCAGTGCAAGAACTTGAAATGCTTGAATCTATGCGGGTGTGTGCGGGCAGCATCCGACAGAGCGTTGCAGGTACTAAAGCTGCGACAACATATTTTGCGTGAACCTTTATTTAATCTCTGTTATCTTGGTGCAGGCTATAGCCTGCAACTGTAGTCAGCTGCAATCTTTGAACCTTGGTTGGTGCGATACTGTCACTGACGGGGGAGTCACTAGCTTGGCATCAGGATGTCCTGAACTTAGGGCCGTCGACTTGTGTGGCTGCGTTCTTATAACAGGTAATTTGTTTGATCGATCTCATGTCCCTGCAGTCTGCACAATCTGGTGCTCTGTTGATTGCTAAAACAAAATATACAAGGATATTAGCACTTTTTTCTCTGCTCTGCTAGGTAGTTTTAACTTTTTAGTTACTATCAGACATGCTGACAGAAACAATATCAGCACTGATCCTGGCTTTCGGCCAAGTTTTATGCTTTGCAATCATATTGTAATGTTAGGACAAGGCATTTGCAACTTGCATTATTTATTGGAATGCACAATCTCAAACATTACATTACACGCATCCTTATATGTCTTCTTACATAACGTGCTCTCTTCCTGATTTTGTTAAGTTGATTCTGCATCCTTTTACCCTTATACACTGTTCTCTGAGGTGCAACCTCCATACTTCTCCAAGATCAAGTGTGCTCACCCAAGTTTATTTCGGTTGTTAATCATGTTCACCATGTAGGCATGTACGGCATTGTTATCCAAGTTCATCATCGTTTACAGTGCTGTGGTTGCTTGTCAGTCGCACGTAGAGGCATGCTCTTTGCCTGAAATCTGACTCATATCCGTTTCTCCTTCTTCAGATGAGAGTGTGGTTGCTCTTGCAAACGGCTGCCCTCACCTGCGTTCCCTGGGGCTGTACTACTGCCAGAACATCACCGACCGTGCCATGTACTCGCTGGCAGAGAACAGCCGCATCAGGAGCAAGGGCATGAGCTGGGACACGGCTAAGAACAGCCGCGGCCGCGACGACAAGGACGGCCTCGCCAGCCTGAACATCAGCCAGTGCACCGCGCTGACGCCCCCGGCAGTGCAGGCGGTGTGCGACTCCTTCCCGGCGCTCCACACCTGCCCGGAGAGGCACTCCCTCATCATCAGCGGCTGCCTGAGCCTCACCACCGTGCACTGCGCGTGCGCCCACCACCGGCACCGCGCCGGGGCCGGGAGAGCCATCCTGTCTAACCATGCCTACTGAGGTCCATCCATGTGTGGATCATGGCAGGGTCTGAGATCCCGGTGGGGCTGAGTTTGTATCGTGTTGTATCACCGAATGTGAAGTGGAACCCAAGGGTGCGGTGGAAGTCTGTGTGTCTGTACTTGGACATTAACAAAATGAGTTCTCTGGAGAATATGTGAGAG

>TaCOI4-D Traes_3DL_3DB9F1EC5.1

CTTTTAGGTGCTCTCGTAAGGAGAGATTGGATTGTTAATACGGAGGTACTGATCCTAAGGCCATCCGTAGCAGTTTTTCTTACGCCACTTCTATCTGACTTTTGGTTCCTTCTGAACGCTCTGATTGACTTGACAGATCAGTGCAGTAGTCTTTGCTTCTTGAAGAATTCTTTTCTCGAACACGCATCTAAATGCGTGACATTCTGCATCAGAAGACACTCTACCTCGGAAGAGAACTCCATGGTTAACAACGGCTCATTAGCATAAGCCAACCAAAAATATTAAAGCGCAGGGCAAGAAAACCAAAACAAAGCAAAAGCAGAAAAGAGAATATAAGTACAGGGCAAGAAACGAAACAAAGCAAAGAGACAAGGACAGCACTACTTATCTAGGCAATAGCGTCTAGGTTCTCGATTGTATTATTGCTCCATTCCTCTGTTTGTGTTCCCTACCTAATCTTGCGGGTATTTATCTTCAGTTCACTAGATGTGACGTGCTTAAGTAAAAGTAGTAAGAACCCTACCATGATCAAGTAATTGCTAGATCTCGGTGAGGGCCTCTGACAGCGACATTCCAGTATGGTTTAGCGCATTCCTGGGAGGTTACTTGGATGCATTTACGTTTTCGTTGCGGATACTTATTACTAATCTAACCTATTGACTCTGTTTATATAAGTTTGCTAATAGCTGCACTACTGAATGCAGGTTTCGCTGTGAAAATGGTCAATGCACAGATGTTGAGTGGGTACTTGGACAATTCGTTCAATGCACTCATGGTTTCTGGTGGCGGTGAGAGTGGACAGCCACAGAATGGTGGCACAGACACCACCTTGTCAGGTTGGAAGGACCTTCCCATGGAGCTTCTGCTGAGGATCATATCAGTCGCTGGAGATGACAGGNNCATTGTAGCCTCCGGTGTTTGCACCGGGTGGCGTGATGCACTAGGATGGGGAGCCACAAGTCTCTCCTTCTCGTGGTGAGTGGTCTTCAATTCTGCATTTCTCTTGTATGCGAAGATGACCAATATTTGATTCTTCAGTTTACAAGATGGTTCAGAATTTTGGTTATAATTATGAAATGCCACTTGTAGGCTGTAAGCACAGCTTATCACTGGGTTTGTAGTTTAGATCATGTTATATATTATATTATTCAATCGTATGATTTATGTTGGAAGTCCACTGCAAATGTGAAATTTTCTTGGTCAATCAATTTGGTTCTTCAGCTGTCCAATAACGTAAGTAAAATGTTGTAGTTATACAACCATTAAAACTGCTCAATCAGATGATATATTTTCCCCAAGAAACTTTAATTTCTTAGTTCGAAATAAATAAATGGTTTGTTCAGGGCCTTATGTGACAACTACAAAATCTTGTCTTATTAGCAATGTTTGATGTGCATCGCAGATGAAAGGTTATTGATTGATCAAAGTGTCATTTTCAGGTGCCAGGACCACATGAATGAGTTGGTGATATCACTGGCCCACAAATTTACAAAGCTTCAAGTCCTTTCTCTAAGGCAAATCAAGCCTCAGCTTGAAGACACTGCAGTGGAGGCTGTAGCAAATTCTTGTCATGATTTGCGCGAGTTGGATCTGAGCAGAAGCTTCAGGCTTAGCGATCGGTCCTTGTATGCTCTGGCACATGGGTGCCCTCACCTTACAAGGCTGAACATCAGTGGATGTTCCAATTTCAGTGATGCTGCTTTGATCTACCTCACTAGTCAGTGCAAGAACCTGAAATGCTTGAATCTGTGCGGGTGTGTGCGGGCAGCATCCGACAGAGCGTTGCAGGTACTAAAGCTGCGACAACATACTTTGCGTGAACCTTTATTTAATCTCTATTATCTTGATGCAGGCCATAGCCTGCAACTGTAGTCAGCTGCAATCTTTGAACCTTGGTTGGTGCGATACTGTCACTGACGGGGGAGTCACTAGCCTGGCATCAGGATGTCCTGAACTTAGGGCCGTCGACTTGTGTGGCTGTGTTCTTATAACAGGTAATTTGTTTAATTGATCTTATGTTCCCTCAGTCTGTACAACCTGGTAGTTGGTTGATTGCTAAAACAAAACATACAAGGATACTAGCACTTTGTTTCTCTGCTAGGTAGTTTAACAGTTTAGTCAATATCGAAGACGCTGACTGACACAAACGATGATATCAGAACTAGTCCTGGCTTTCTGCCAGGTCTATGCTTTGCAATCATATTGTATTTAGGACAAAGGCATTTGCAACTTGCATTATTTATTGGAATGCAACATCTCTAACATTACATTACATGCATCCAATTACTTAGTGCATCCAAAATTTTGTTAAGTTGCTTCTGCATCCTTTTTACCCTTATACACTGTTCTCTGAGGTGCAACCTTCATACTTCTCCAAGATCAAGTGTGCTCACCCAAGTTTATTTCGGTTGGTAATGATCTTCACCATATACGGCATTGTTATCTAAGTTCAGCATCGT

>TaCOI5-A Traes_4AS_AD3F2B991.1

CCATGAGCTTCGGGATCCCGGACGTGGCGCTGGGGCTGGTCATGGGGTGCGTGGAGGACCCCTGGGACCGCGACGCCATCTCGCTCGTCTGCCGCCACTGGTGCAAGGTCGACGCGCTCAGCCGCAAGCACGTCACCGTCGCCATGGCCTACTCCACCACCCCCGACCGCCTCTTCCGCCGCTTCCCCTGCCTCGAGTCGCTCAAGCTCAAGGCCAAGCCCCGCGCCTCCATGTTCAACCTCATCCCCGAGGACTGGGGAGGCTCCGCCTCGCCCTGGATCCGCGAGCTCTCCGCCTCCTTCCACTTCCTCAAGGTGCTGCACCTCCGCCGGATGATTGTCTCCGACGACGACGTTGCCGTGCTCGTGCGCGCCAAGGCCCACATGCTCGTCTCCCTCAAGCTTGACCGCTGCTCCGGCTTCTCCACCTCCTCCCTCGCTCTCCTCGCCCGCTGCTGCAAGTAATTTCTCCCCTCCTTGTTCCGGTTCACAAATGATGTGCATGCATTAGTTCGTTTTAATTACTGCGGTATCTCTTTCTTGTCCTACGAATTAATCTGTAGTTTCAATTGTTCTAGTTAGTTTTTCTCTTCAGAATTGTGTTTTGTTCTATTCGGAAAGCGTTGGTAGATGGGATGTTTGTAAAGCGAGTATATATAAGATGGTTCTTTTGTTCATTTGGAAAGGGGAAAAGTAAGTGTAACGTCTCACCTTTTCATGATGTAAAACATCATGCTACTCTTTGCAATTTGGGAATAATTCTAGCTTTGGAGCAGAAATTAGGAAATGTGGACTTTAACTGCCTTACTGAGTTGGGGACTTTGCTTAGTTGTTTATACTTTCTTAGAGAACTATGATACTTGGTGGAGATAGTTTTGAAAAGCAGATTATCAAAATGGTGCTGTAGAAGTTTAGTAAGAATGTAAGAGCATGCATTCATAGGTTACTAGTTTAATTAATCATATTTCTTGTCATGTGCAGGAAACTGGAAACGTTGTTTCTTGAAGAAAGTTCTGTTGCTGAGAAAGAAAATGATGAATGGCTCCGTGAGCTTGCTACCAGCAATACTGTCCTTGAGACGCTGAATTTCTTTCTGACGGATCTCAGGGCATCCCCTGCACATCTTCTCCTCCTTGTGCGAAATTGCCGAAGGCTGAAAACTCTCAAGATTAGCGACTGTTTCATGTCTGACCTGGTCGACCTGTTCCGTACAGCAGAAACACTACAAGACTTTGCTGGTGGTTCCTTTGATGATCAAGATCAAGGTGGGAATTATGCTAACTACTATTTCCCTCCTTCAGTACAGCACTTGAGTTTGCTCTACATGGGAACAAATGAGATGCAGATATTATTTCCATATGGTGCCACACTCAAGAAGTTGGACCTTCAGTTCACATTCCTTACCACAGAGGATCACTGTCAATTAGTCCAGCGCTGCCCAAATCTAGAAGTTTTGGAGGTATGTCCTGGCAAATTATTTTAAATGTTTTTCCCATCTTTCCTGTCTGGAAGTCTGATAGTGAATAAGCTATCTCAAACGTGTTTTGTTGATAATTTGTCTTCCAACAGGTGAGGGATGTGATAGGAGATCGAGGGTTAGAAGTTATTGCGCGGACCTGCAAGAAATTACAGCGACTCAGAGTCGAGAGAGGAGATGATGACCAAGGAGGTCTTGAGGACGAACAGGGTAGAGTGACACAAGTAGGATTGATGGCTGTAGCTGAAGGCTGTCCTGATTTGGAGTACTGGGCAGTACATGTGTCTGACATTACAAATGCAGCTCTTGAGGCTATTGGCGCATTCAGCAAAAACCTGAACGATTTCCGACTTGTCCTGCTTGATAGAGAGGTGCATATAACTGAACTGCCCCTTGACAACGGGGTTCGGGCTTTGCTGAGAGGTTGCACCAAACTCCGGAGGTTTGCATTTTATGTGAGACCTGGAGCTCTATCAGATATTGGCCTTTCTTATGTTGGCGAATTTAGCAAGACCGTCCGCTACATGTTGCTTGGGAATGCCGGGGGGTCTGATGATGGACTGCTGGCATTTGCACGAGGATGCCCAAGCTTGCAGAAATTGGAGCTAAGGAGTTGCTGCTTTAGTGAACGTGCATTGGCAGTTGCAGCCTTACAGCTGAAGTCACTCAGATATCTTTGGGTGCAGGGATACAAGGCATCTCCTACTGGCACCGATCTCATGGCAATGGTACGCCCCTTCTGGAACATTGAGTTTATTGCACCAAATCAAGATGAGCCTTGCCCAGAGGGTCAGGCACAGATTCTGGCATACTACTCTCTGGCTGGGGCAAGGACAGATTGTCCTCAGTCAGTAATTCCCCTCCATCCGTCAGTGGGAAGCTAAAAAGACCACCACCAGTTTGACTGTACATACATGTTTGATGCCAGCAAAACCCACAATGCGGTATAGGGACATTCCACCTTACAGTGCCAATTACGGGACTGAAAGCTCAAGTAAAAGCGACCCACTCTGAACTGCCTTGGTATCTTAGGGGCAACATTTTTGGGTAAGCTGTTCATCTGGCCAACATGGATATCTTTGTGTACTACACCATTTTGACATGGCTCGGACACGCATTTTTGTAATAATGTGCCCAGTTGTAATGGCATTTTTCTGTTCTTGAGCTTTGCCCACTGTATTGTTGTTCTACAAACAGTATTGGATTAGTTGTTGTACCATCTGTGAAACAATCTGCACAATGTTATGTTTAACCCATGAATATCTTGAAATTTTGCTGCGGCT

>TaCOI5-B Traes_4BL_499E7F095.1

CCCCCGTTGAAATCCGCGGCGGGACAGATCGGGGCGCAGATCGGGGGCCGGCTCCCGGCGATGGGCGGCGACGAGCGGCACCTGGGGAGGACCATGAGCTTCGGGATCCCGGACGTGGCGCTGGGGCTCGTCATGGGGTGCGTGGAGGACCCCTGGGACCGCGACGCCATCTCGCTCGTCTGCCGCCACTGGTGCAAGGTCGACGCGCTCAGCCGCAAGCACGTCACCGTCGCCATGGCCTACTCCACCACCCCCGACCGCCTCTTCCGCCGCTTCCCCTGCCTCGAGTCGCTCAAGCTCAAGGCCAAGCCCCGCGCCTCCATGTTCAACCTCATCCCCGAGGACTGGGGCGGCTCCGCTTCGCCCTGGATCCGCGAGCTCTCTGCCTCCTTCCACTTCCTCAAGGTGCTGCACCTCCGCCGAATGATTGTCTCCGACGACGACGTCGCCGTGCTCGTGCGCGCCAAGGCCCACATGCTCGTCTCCCTCAAGCTTGACCGATGCTCCGGCTTCTCCACGTCCTCCCTCGCTCTCCTCGCCCGCTGCTGCAAGTAATTTCTCCCCTCCTTGTTCCGGTTCACAAATGATATGCATGCATTAGTTCGTTTATTAATTACTGCGGTATCTCTTTCTTGTCCTACAAATTAATTTGTAGTTTCAATTGTTCTAGTTAGTTTTTCTCTTCAGAAATTGTGTTTTGTTCTATTTGGAAAGGGTTGGTAGATGGGATGTTTGTAAAGCGAGTATATATAAGATATTCTTTTGTTCATTTGGAAAGGGGAAAAGTAAGTGTAACGTCTCACCTTTTCATGTAGTAAAACATCAGGCTACTCTTTGTAATTTGGGAATAATTCTAGCTTTGGAGCAGAAATTAGGAAATGTGGACTTTAACTGCCTTACTGAGTTGGGGACTTTGCTTAGTTGTTTATACTTTCTTAGAGAACTATGATGCTTGGTGGAGATAGTTTTGAAAAGCAGATTATCAAAATGGTGTTGTAGAAGTTCAGTAAGAATGTAAGAGCATACATTCATAGGTTACTAGTTTAATTAATCATATTTCTTGTCATGTGCAGGAAACTGGAAACCTTGTTTCTTGAAGAAAGTTCTGTTGCTGAGAAAGAAAATGATGAATGGCTCCGCGAGCTTGCTACCAGCAATACTGTCCTTGAGACGCTGAATTTCTTTCTGACAGATCTCAGGGCATCCCCTGCATATCTTCTCCTCCTTGTGCGAAATTGCCGAAGGCTGAGAACTCTCAAGATTAGCGACTGTTTCATGTCTGACCTGGTTGACCTGTTCCGTACAGCAGAAACGCTACAAGACTTTGCTGGTGGTTCCTTTGATGATCAAGATCAGGGTGGGAATTATGCTAACTACTATTTCCCTCCTTCAGTACAGCGCTTGAGTTTGCTCTACATGGGAACAAATGAGATGCAGATATTATTTCCATATGGCGCCACACTCAAGAAGCTGGACCTTCAGTTTACATTCCTTACCACAGAGGATCACTGTCAATTAGTCCAGCGCTGCCCAAATCTAGAAGTTTTGGAGGTATGTCCTGGCAAATTATTTTAAATGTTTTCGCCATGTTTCCTGTCTGGAGGTCAGATAGTGAATATGCTATCTCAAACGCGTTTTGTTGACAATTTGTCTTCCAACAGGTAAGGGATGTGATAGGGGATCGAGGGTTGGAAGTCGTTGCACAGACCTGCAAGAAATTACAGCGACTCAGAGTCGAGAGAGGAGACGATGACCAAGGAGGTCTTGAGGACGAACAGGGTAGAGTGACACAAGTAGGACTGATGGCTGTTACTCAAGGCTGTCCTGATTTGGAGTACTGGGCAGTACATGTGTCTGACATTACAAATGCAGCTCTTGAGGCCATTGGTACGTTCAGCAAAAACCTGAATGATTTCCGACTTGTCCTGCTTGATAGAGAGGTGCATATAACCGAACTGCCCCTTGACAACGGGGTTCGGGCTTTGATGAGAGGTTGCACCAAACTCCGGAGGTTTGCATTTTATGTGAGACCTGGAGCTCTATCAGATATCGGCCTTTCCTATGTTGGTGAATTTAGCAAGACCGTCCGCTACATGTTGCTTGGGAATGCCGGGGGATCTGATGATGGACTGCTGGCATTTGCACGAGGATGCCCAAGCTTGCAGAAATTGGAGCTAAGGAGTTGCTGCTTTAGTGAACGTGCATTGGCAGTTGCAGCCTTACAGCTGAAGTCACTCAGATATCTTTGGGTGCAGGGATACAAGGCATCTCCTACTGGCACCGATCTCATGGCAATGGTACGCCCCTTCTGGAACATTGAGTTTATTGCACCAAATCAAGATGAGCCTTGCCCAGAGGGTCAGGCACAGATTCTGGCATACTACTCTCTGGCTGGGGCAAGGACAGATTGTCCTCAGTCTGTAATTCCCCTCTATCCATCAGTCGGAGGCTAAAAAGACCACCACCAGTTTGACTGTACATACATGTTTGATGCCAGTGAAACCCACGATGCGGTATTAGGGACATTCCACCTTACAGTGCCAATTACGGGACTGAAAGCTCAAGTAAAAGCGACCCACTCTGAACTGCCTTTGTATCTTAGGGGCAACATTTTTGGGTAAGCTGTTCATCTGGCCAACATGGATATCTTTGTGTACTACACCATTTTGACATGGCTCGGACACGCATTTTTGTAATAATGTGCCCAGTTGTAATGGCATTTTTCTGTTCTTGAGCTTTGCCCACTGTATTGTTGTTCTACGAACACTATTGGATTAGTTGTTGTACCATCTGTGAAACAATCTGCACAATGTTATGTTTAACCCACGAATATCTTGAAATTTTGCTGCGGCTTGGCCTCAGCTTTACTGGTCGTGATTTTCC

>TaCOI5-D Traes_4DL_8C2C2ADD4.1

CACACCCCCGTTGAAATCCGCGGCGGGACAGATCGGGGCGCAGATCGGGGGTCGGCTCCCGGCGATGGGCGGCGACGAGCGGCACCTGGGGAGGACCATGAGCTTCGGGATCCCGGACGTGGCGCTGGGGCTGGTCATGGGGTGCGTGGAGGACCCCTGGGACCGCGACGCCATCTCGCTCGTCTGCCGCCACTGGTGCAAGGTCGACGCGCTCAGCCGCAAGCACGTCACCGTCGCCATGGCCTACTCCACCACCCCCGACCGCCTCTTCCGCCGCTTCCCCTGCCTCGAGTCGCTCAAGCTCAAGGCCAAGCCCCGCGCCTCCATGTTCAACCTCATCCCCGAGGACTGGGGCGGCTCCGCCTCGCCCTGGATCCGCGAGCTCTCCGCCTCCTTCCACTTCCTCAAGGTGCTGCACCTCCGCCGGATGATTGTCTCCGACGACGACGTCGCCGTGCTCGTGCGCGCCAAGGCCCACATGCTCGTCTCCCTCAAGCTTGACCGCTGCTCCGGCTTCTCCACCTCCTCCCTCGCTCTCCTCGCCCGCTGCTGCAAGTAATTTCTCCCCTCCTTGTTCCGGTTCACAAATGATATGCATGCATTAGTTCGTTTATTAATTACTGCGGTATCTCTTTCTTGTCCTACGAATTAATTTCTAGTTTCAATTGTTCTAGTTAGTTTTTCTCTTCAGAAATTGTGTTTTGTTCTATTTGGAAAGGGTTGGTGGATGGGATGTTTGTAAAGCGAGTATAGATAAGATGTTCTTTTGTTCATTTCGAAAGGGGAAAAGTAAGTGTAACGTCTCACCTTTTCTTGATGTAAAACATCATGCTACTCTTTGTAATTTGGGAATAATTCTAGCTTTGGAGCAGAAATTAGGAAATGTGGACTTTAACTGCCTTACTGAGTTGGGGACTTTGCTTAGTTGTTTATACTTTCATAGAGAACTATGATACTTGGTGGAGATAGTTTTGAAAAGCAGATTATCAAAATGCTGTTGTAGAAGTTTAGTAAGAATGTAAGAGCATACATTCATAGGTTACTTGTTTAAATTAATCATATTTCTTGTCATGTTCAGGAAACTGGAAACGTTGTTTCTTGAAGAAAGTTCTGTTGCTGAGAAAGAAAATGATGAATGGCTCCGCGAGCTTGCTACCAGCAATACTGTCCTTGAGACGCTGAATTTCTTTCTGACGGATCTCAGGGCATCCCCAGCATATCTTTTCCTTCTTGTGCGAAATTGCCGAAGGCTGAAAACTCTCAAGATTAGCGACTGTTTCATGTCTGACCTGGTCGACCTGTTCCGTACAGCAGAAACACTACAAGACTTTGCTGGTGGTTCCTTTGATGATCAAGATCAAGGTGGGAATTATGCTAACTACTATTTCCCTCCTTCGGTACAGCGCTTGAGTTTGCTCTACATGGGAACAAATGAGATGCAGATATTATTTCCATATGGTGCCACACTCAAGAAGTTAGACCTTCAGTTTACGTTCCTTACCACAGAGGATCACTGTCAATTAGTCCAGCGATGCCCAAATCTAGAAGTTTTGGAGGTATGTCCTGGCAAATTATTTTAAATGTTTTTGCCATCTTTCCTGTCTGGAAGTCTGATAGTGAATATGCTATCTCAAACGTGTTTTGTTGATAATTTGTCTTCCAACAGGTGAGGGATGTGATAGGAGACCGAGGGTTAGAAGTTGTTGCGCGGACCTGCAAGAAATTACAGCGACTCAGAGTCGAGAGAGGAGACGATGACCAAGGAGGTCTTGAGGACGAACAGGGTAGAGTGACACAAGTAGGATTGATGGCTGTAGCTGAAGGCTGTCCTGATTTGGAGTACTGGGCAGTACATGTGTCTGACATTACAAATGCAGCTCTTGAGGCCATTGGTGCGTTCAGCAAAAACCTGAACGATTTCCGACTTGTCCTGCTTGATAGAGAGGTGCATATAACTGAACTGCCCCTTGACAACGGGGTTCGGGCTTTGCTGAGAGGTTGCACCAAACTCCGGAGGTTTGCATTTTATGTGAGACCTGGAGCTCTATCAGATATTGGCCTTTCTTATGTTGGCGAATTTGGCAAGACTGTCCGCTACATGTTGCTTGGGAATGCTGGGGGATCTGATGATGGACTGCTGGCATTTGCACGAGGATGCCCAAGCTTGCAGAAATTGGAGCTAAGGAGTTGCTGCTTTAGTGAACGTGCATTGGCAGTTGCAGCCTTACAGCTGAAGTCACTCAGATATCTTTGGGTGCAGGGATACAAGGCATCTCCTACCGGCACCGATCTCATGGCAATGGTACGCCCCTTCTGGAACATTGAGTTTATTGCACCAAATCAAGATGAGCCTTGCCCAGAGGGTCAGGCACAGATTCTGGCATACTACTCTCTGGCTGGGGCAAGGACAGATTGTCCTCACTCAGTAATTCCCCTCTATCCGTCAGTCGGAAGCTAAAAAGACCACCACCAGTTTGACTGTACATACATGTTTGATGCCAGTGAAACCCACGATGCGGTATAGGGACATTCCACCTTACAGTGCCAATTACGGGACTGAAAGCTCAAGTAAAAGCGACCCACTCTGAACTGCCTTCGTATCTTAGGGGCAACATTTTGGGGTAAGCTGTTCATCTGGCCAACATGGATATCTTTGTGTACTACACCATTTTGACATGGCTCGGACACGCATTTTTGTAATAATGTGCCCAGTTGTAATGGCATTTTTCTGTTCTTGAGCTTTGCCCACTGTATTGCTGTTCTACGAACAGTATTGGATTAGTTGTTGTACCATCTGTGAAACAATCTGCACAATGTTATGATTTACTGGTTGTGATTTTCCACCATCTGAACCCACGAATATCTTGAAATTTTACTGCGGCTTGGCCTCAGCTTTACT

>TaCOI6-A Traes_5AL_27F194099.2

CGATCCGTCCGGACGGGGGCTGGTGACGAACCCTAACTTGCTTGTACTGTCCGGATGGTTTTGATCTCGTATTTTCTTTCCTTTCTGTGAGCTGTGAGGAGTTTAATCCATCTAGCTTCATGCGGATTCGTTCGCTGCTTGTTGAGCTGATCATAGCTGTGGGTTCCTTTTCCGGAAGAAAAAGAAGAGGAAATTTTGGGGTTCCAGTCCAGCCCAACCCCATGGCACTCTTTGGGTGTGCGGTCGGAGGACATCTCTGGGTTTTCTTGCGAACACTTCTACTTATGGAAAGATGCAAAAAATTCGTGCGACTGTTGCTTAATTAACTGTGTTAATTCTTGGAAATTTATGATCAAGTACTTATTTGAGTATGAATATATATCATATATCAGCCTGTACAGTACCAATTTCAAGCAAATGGTAGTTAATTGGTACCCAGTGCTAACACAATGTTTTGTTTCTTGTGCTGGTGTGACTGTGAACAGGTCACGGCCGGAAACTGTAAAAGTTAATTGTGGTTTGTTAGGATTCTGGGAGGCGGCAACATGCCCTACTTCCCAGATGAAGTGGTGGAGCACATCCTTGGCTTCATATCGTCGCACCGTGACCGCGATGCTGCATCTCTCGTGTGTCACGCGTGGTACCGCATCGAGGGCCTCACTCGCCGCTCCGTGTTCATTTCCAACTGCTATGCCGTGCGCCCAGAGCGTGTGCACGCGCGTTTCCCCTGCCTGCGCTCGCTGACCGTGAAGGGCAAGCCACGCTTTGCTGACTTCAACCTTGTCCCTGCGGGGTGGGGTGCCTCAGCGGAGCCATGGGTGGATGCGTGTGCCCGTGCGTGCCCTGGCCTTGAAGAGCTCCGCCTGAAGCGGATGGTTGTCACTGATGGTTGCCTCAATCACCTTGCTCACTCATTTCCCAATTTGAGATCACTAGTCCTTGTTAGCTGTGAGGGGTTCAGCACTGATGGCCTTGCTACTATTGCCACCAATTGCAGGTCAGAATGCTACAAGCTTGGAAGTCTTTCACATTTCATGTTATGATAATAGTACTGACTACTGAATGTGGATGTCTTCCTTGTACAATTGTTTGAATCTTGGATACCGAATTTATGTTATGTTTGTTTTAGAATGGAAACCGAGAAACTATCAATGACATTTGAATGATCCTACAGAAAAACCTTGATAAGTCCTTGTAAGTTGGCATTGCTCTATCTCCATTTCCATGAGTTCTGATAAACAATAATGATTGAGATTGTGGATGCACTTCAGTTTATTAAAGAAAGGTCTTATTTTAAGGGATGTTTCTTCAAACCAGTTTGTGCGAACTTATTTAAGAGATTGTGAATGCATTGCATATTGGGGCAATCCATGGTTCCAGTGTTATATAATCTTCATGACTGCATATACATTTTGAGATGTGATCAATATAAAAAAGTTTGGTATGCTGCATTTATATAAGTGGAATCTTGAGGGCTCTTTCACCAGTAACTTTAAAAGACATATGTGGACTGTCGGTGGTTTACATATAGCAATGTGGCATTACCAGAACATCGGAAAGAGAGGAAGGCACTAATGTAGTAATGTTGGCTATGTCATATCATTCCAGTTTAAAGGAGGATTAGAATACTCGACGGTTCTTTCACCAGTAATTTATTACCAATTTCAAATATGCCTGGTCTAGAGTCTCTCGATCCTCCCATCAATATAATTGAAGTGCTCGCCTTTTTTTTGAAAGAAAACTATCATGGAAACCCCTACAGTATATATAGGAATATAAATTCACGTCTGTGAGGACTTGAACCTAGGTGGTGGGTTGTACGTCCACTCCCACAACCTCTTGAAGGGCTCGCATTTGAAGTGTTACCAACTCTTAATTAGGCAAATAATGGTAATTATTCACTATTCTTAACCATAATCATCGTACATATTTTTTTACTAAGTTTTGAAACTCACCACAAACTTGTTACTGTAGTTGCATAATTTGGTACGTATCTCTGTGGGTGGATTCCATGTGCCCACCGATCGGTATTTATTTAAGGCTAGGGAATTTCAGCAATATAAACTTAAAACCTTAGTACCAATGTACGCAACAGGGTAAACACTAACGAAGGGTAGCTTGTACTGGCAGTGTCGGCTTGGTTGGGGCAGAGTCAGATCCTAGGTGCTGGCGATCGACTTTGTAGGAGATAACAAATTTGGTTCTTCATTCTTTAGTTGCTGCGTCATACGGCACGCCGTATATTGGTGAGCCTGCCCTCTTAAACAATGAAACTCCTTAGTCTCACACAACGAAACCTGGAGATCACAACTTGAAAGATAAAAAGGAGTGGTTAAAGGGGGCAGTTCGCTCGCTAAACTGTGCGTTGTTGAGGTAAAAGTGAGGCACCTGCGGAGGCTGCGCCTCAAACGTGGGTGGAGGAGCTCACAGGTGTGTGCTCTAGCCAACCGAACACCGCTCGGTTCTCACAGCTTGAAAGATAAGATAATGGAAAATATCTTCTCATTAATCTTAGGTGTACATGGTTGTTACTAGCATTGTGCAGCCCACTTAGTGTAGGTCCAAGGCCCCACGTTCTGATCTCCACCCATAACATTTGTAAAGCCCATACGTAAGGCACTTAAAATTTGTTTCATATTTATTATTAACAGTTACAGCTATTATTACACTTCCAGTCATATTTACATATTTTCTCATGTCTATTCATAAAGCATCATTTCCATGTGACTCCAGATTGGATTATTATAATTTATGTGACATATATTTGGTGATACCATCTTTAAGGAAATTCAATAGTTTGCGCACTGTCCGTTTGTTTTCTGTAGAACTGGTAGGTCGTTTGCATCAGTAGTCACCAACAATGATGTCATTCGTAAACCGGATTATTTTGATAACAATTAGAATGTTAATACCTGGCAAAATTGTGTATCTTAGAAGTTATTGTCTCATACACATGTTCTGTTCTATTAAGATTAATTAATCTCGACCTCTCTTCAGTTTTTTAAGTGTTGCTTCAGAAAAGTTCTTTCGGTAATTGCAAGTCATATTACTTAATTTGTAAACTCTTCATTATAGGTTTCTGAAGGAACTTGACTTACAAGGGAGTCAGGTGGAGTTTCGAGGCCGTCATTGGTTTAGTTGTTTCCCCAAGCCTTCGACATCATTAGAATCCTTGAATTTTGCTTGCTTGGATGGAGCAGTGAGTGCTAATGCATTGGAAAGTCTTGTTGCAAGGAGTCCAAATCTTAAAAGCTTAAGGTTAAATCGTGCAGTTCCACCAGCTGTTTTAGCCAAAATTCTTACTTCCGCTCCTAAGCTGGTGGATTTAGGTACAGGATTGGTTGCTCAAAGCAATAATGCTGGTGCACTCCCCAGTCTCTACAGTGCTATTCAACAATGCAGTTCTCTGAATAGTTTATCTGGCTTTTGGGATTCTCCACGTTGGATTACTCCAATAATACAATATATTTGCAAGAACCTAACATGCTTGAACCTTAGCTATGCTCCAATGTTTCGGACAGTTGATCTTATTGGAATTATTCGCCAATGTCAGAATCTCCGACACTTGTGGGTAAGACTTAGTAGCACTGGTGGTAGGGAAATTTAGTCTGTTTCTTGTTATGTTAAATTACTTTATGCAGTTACTAGAGCTTTACTTTGTTGGCTGTGTTGTTTCACTTCAAGCCTATGAAATGAGATGTTTACGTATATGGAAGTTCTACTTTTGTCAACCCTGCTGTAGTGTAATTAGCAAGTAATGTTGCCCAATATGCTGTTTTAAGTTGTACTTATTAGGCAAGAATTTTGTAAATTTAACACACAAAATACATTTAGTTCAACTTCTTATGTGTTAGGATGTGTTTGGTTCAAGGGATCAGTTTGGGTTGTTGAGGGTATCCCCAACCACCCGGTTAGGGATAGCCATTTTTTCTGTTTGGTTGTATAGGGATGAAAAAGGGTTGAGAATAGCCATTTGATGTTTGGTTTGAGGGATGATGGATGGATAGGTTGTTCCAAGAATCTTATTTCTTTGAGGTGGTCAGTTATTTTTTTATGGAAAACAATCATATTTCTGAAGGTGAATAAGTATACAGGGCACATAATTCACGAACAATCCATCTAAATTTGATAGTATATTCATCAAGTAAAATTCAGAGCAAATGCTTGCCGCCTCTGTGCGCTCTGTTCGCTGCTCCTGTAAAGAGAAAATCAACAGAAAACGAGAACACAAGGAGCAGCTCGTGTTCACTGTAAACAAATATATGCATGGCAGCTCGTGGAGTACTAACTATGCAAGATTGCCTAAAGAAAAACAATGCAAGATAGGAGAGCAAGGAGCAGAGGAGCAGCTCCTGTGCCCCGACCTCAGGCCGTCAGGCGGATGCACGGCGTCCCTGTCGGCGGCTGCGCACTCCTGTACAGCACCACCAGTCCACCACTCAGCTTCCCTGTCCACGCCCGAGCTTCACCGCTGGCCAAATCCAGCTGCCCCAACGCGCTCTACCACGGCCGTGCGCTCCGCCATCGCACACGACTTGCCGGCCACCACCAGCAGCAGCCATGGCGTCGTTAGATCTTGTGCAGNNNNNNNNNNNNNNNNNNNNNNNNNNNNNNNNNNNNGCGAGAGAGAGAGAGAGAGGAAAAATGAGATGGGGTGTACCACTTCGAGCGAGAGCGAGGGTGGTTGCCCATATCCGCCACTTTTCGGCGGCTCCGCTCAGCCCGTTTTTCAGGGAATATTCTTCGAATCTGGCTATCCCCAACCACCCAAAACTCTGAACCAAATGCCTGATAGCCATACAAAAAAAGGGCTATCCCTAGCCTCTCGAGGATATTCCTTGAACCAAACGCACCCTTACTCTTTGACAGAGGTTGCATAAGAAGTTAAGAACACACGTATCTGTTGACTTGTTTAACGATCTACTAGTTTGTACCAGTATAGACATTTGAAAACATCAGACCCTGCAAATGTGGCAAGATATAATAATCTTAAAAATGTCAGCGAACTTTGGAAGTACACCCTTTTTTATCTCTCTTGGCATCTTTGGTAATGCTACAGCGGTTTATACTTTATAGTATGCATCTCTGGCCATGCTGTGCACAAGGAAAAGTTGTAGGCGTGAAAATTCTGTCAACGTAGTACTGTATTTTGAGAATGACCAAAGACAAAATGATGATTTAGTAATAACTGATATGGGATTGATTGATGTTAGGGAAACATTGTCTAATAGCTATTGTACTTAATTGTCAGTGTTACTTAACGCGCTCACCCACACAAACCACACACAAAGAGGTGACATACTTTTGGGTGTTTCAACGTATCTTTGTGTGAGTGTTTCTAATCCCAGAGAAGGTCCTTCTGTGGGTGTTTTAACAGCTCTGTAACTAGTATTGGAAGGTTACTTCAATAGTTATAGTGGAACACTTCTGGTAAAAGGATTCTTAGAGTATACCTTTGTTTTTCTTATGTTTCCTTCATTTGTGTTTCATCCTTGCTGGTTGTTTCTTGTTATAAAATGTTATAATTCACTTCAGCTTAATGGTTGTAGGTACTAGATCACATTGGTGATGCAGGATTAAAGGTTGTAGCCTCTTCTTGCCTGGAGCTGCAAGAGTTGAGGGTATTTCCTGCGAATGCAAATGTGTTAATAAGCACTGGTGTGACAGAGGAAGGGCTGGTTGCAGTATCTTCAGGCTGTCGGAAGCTAAACTCTGTTCTCTATTCCTGCCGTCGAATGACTAATTCTGCTCTGATCACGGTGGCAAAGAACTGCTCGCGAATCACGTCCTTCAGACTGCATATCTGCCTGCATGGGTCAGTAGATGCCGTGACAGGCCAGCCACTGGACGAGGGTTTTGGGGCAATCGTCCGGTCATGCAAGGGCCTCAGGCGCTTATCTATGTCTGGCCTTCTCACGGACAGTGTGTTCCTGTACATCGGCATGTACGCCGAGAGGCTGGAGACGCTCTCTGTCTCGTTTGCAGGAGATAGTGATGATGGCATGATCTATGTGCTCAATGGCTGCAAGAATCTCAGGAAGCTGGAGATCAGGAACTGCCCATTTGGCAACACCGCGCTTCTCGCAGGCATGCACCGGTACGAGGCAATGCGCTCGCTTTGGATGTCGTCGTGCGACATCACCCTGGGTGGCTGCAGGTCCCTCGCGGCAGCCATGCCAGGCCTCAACGTCGAGGTCATCAGCCAGGCGGATGGAGGCGCCAACGATGCAAAGAAGGTGGAGAAGCTGTATGTCTACAGGACACTCGCCGGCCCAAGGGTTGATGCCCCTGGATTCGTCTCGGCACTGTAAATTACTTATACCGTCGTAGTTGCTGGGTTTTCCTAGACCCTGCTATTTGTGTGAACTTAATTCGGTGCTGTCGCACTTTTATGCTACGTCTGCTACTCTGAAGCGGGCACCAGTCTTGTGAATTTGGTTAGTCTTCCGAAGCGCTATTTGTGTGGCCAACACAGCATAATATATGGATCTAGGCCTTATTTCCAAGGTTGCACTGCGGTATAGCTGAACTGTTTGTGACGCAACGAGCTGATCTGTTCTGTTACTGTGTGCTTGGTTACTGAACCAAGTGGAACGGAATGCTATTTTTATTTTTTATTTTCTTTATTAGAAGAAGTGGAACGCAATGTTATGGTTTCATCTT

>TaCOI6-B Traes_5BL_7AE04C6B2.1

CTTCATTCCCCTCTGACGGTCATCGCGCTCCACTCCGCCGGCCGAGCACCCGCCCCCGGGTCCAGGCCTCCTTCCACCGAGGAACGCCACCTCCAAGGGATTCTCCTCTCAATCCACGGTCGCCGCCGTCCTCCTCCTCATCCGCAGCTTCTCCGCTCCACTGGACTGCCGATCCGTCCGGACGGGGACTGGTGACGAACCCTAACTTGCTTGTGCTGCCCGGATAGTTTCGATCTCGTCTTTTCTTTTCTTTCCTTGCCGTGATGTGTGAGAAGTTCAATCCGTCGAGTTTCCTGCGGATTCTGTCGCTGCTTGGTGAGCTGATGATAGCTGTGAGTTCCTTTTCCGGAAGAAAAAAAAAGAAGAGGAAATTTTGGGGTTCCAGTCCAGCCGAACCCCATGGCACTCTTCGGGTGTGCGGTCGGAGGACGGCGCCGGTCGTGCTCGGTGCTTTTCTTGCGAACGCTTTTACTTTTGGAAATATGCAAAAATTCGTGTGACTGTTGCTTAGTTAACTGTGTTAATTCTTGGAAATTTATGATCAAGTACTTCTTTGAGTATGAATATATATCGCATATCAGCCAGTACAAGTACCAATTTCAAGCAAATGGTCGTAAATTGGTGCCCTGTGCTAATACAATCTTTTGTTTCTTTGCTTGTGTGACTGTGAACAGGTCATAGCCGTAAACTGTAAAAGTTAATTGTGGCTTGTTAGGATTCTCGGAGGCGGCAACATGCCCTACTTCCCAGATGAAGTGGTGGAGCACATCCTTGGCTTCGTATCGTCGCACCGTGACCGCGACGCTGCATCTCTCGTGTGCCACGCATGGTACCGCATCGAGGGCCTCACTCGCCGCTCCGTGTTCATTTCCAACTGCTATGCGGTGCGCCCGGAGCGTGTGCACGCCCGTTTCCCCTGCCTGCGCTCGCTGACCGTGAAGGGCAAGCCACGCTTTGCTGACTTCAACCTTGTCCCTGCGGGGTGGGGTGCCTCAGCGGAGCCATGGGTGGATGCGTGTGCCCGTGCATGCCCTGGCCTTGAAGAGCTCCGCCTGAAGCGGATGGTTGTCACTGATGGTTGCCTCAATCACCTTGCTCACTCATTCCCTAATTTGAGGTCACTAGTCCTTGTTAGCTGTGAGGGGTTCAGCACTGATGGCCTTGCTACTATTGCCACCAATTGCAGGTCAGAATGCTACAAGCTTGGAAGTCTTTTACTTTTCATGTTATGATAATGGTACTGAGTGTGGGTGTCTCCCGTTAGACATTGAATGTAAATGGGTTATCACCAGCATCGCCTCATAAACTGCAATTCGTATTCAACCTTCTTGCCATACCCAGTTTATCTCGATGCTCTACAATTTTGTACCAGATCATGGAAGCTTGTTTATCCTTGTACAATTGTTTGAATCTTGGATACCTAGAATGGAAACCGAGAAACTATCAATGACATTTAAATGATCCTACAGAAAAACCTTGATAAGTCCTTGGCAGTTGGCATTGCTCTATCTCCCTTTCCATGAGTTCTGATAAACAATAATGATTGAGATTTTAGATGCATTTCAGTTTATTAAAGAAGGTCTTATTTTAAGGGATGTTTCTTCAAACCAGTTTGTGAGATTGTGAATGCATTGCATACTGGGTCAATCCATTGTTCCAGTGTTATATAATCTTCATGACTGCATGTATACATTTCAAGATCTGATCAATATAAAAAAGTTTGGTATGCTGCACTTATATAAGTGGAATCTTGAGAGCTCTTTCACCAGTAACTTTAAAAGACATATCTGGACTGTCGGTGATTTACATATAGCAATGTGGCATTACCATAACATCGGAAAAAGAGGGAGGCACTAATGTAGTAATGTTGGTTATGTCATATCATTCCAATTTAAAGGAGGATTAGAATACTCGAGGGTTCTTTCACCAGTAATTTATTGCTAATTTCAAATATGCCTCTAGAGTCTCTCGACCCTCCCATCAATATAATCAAGCATGAATTTCGAGTTGCGCGACTAGTCGTCGACTAGTCGATGAGTTGCAAAAAAATGTCGACTCAACTTAGTGTCGACTTGAGTCGTGAGTCGGGACTAGTCGCAACTTCAGGCTCGACTTTTTCCGAGTCGCTACCCCAGAGCGACTCGACTCGGATGAGTCGCGACTCGAAAACCATGAATCGAAATGCTTGCCTTTTAAAAAAAAAGAAAACTGTCAGAAACCCTTACAGTATAATATAGGAATATAAAATCACGTCCATGAGGACTTGAACCTAGGTGGTGGGTTGTACGTCCACTCCCATAGCGTCTTGAAGGGCTCACATTTGAAGTGCTACCAACTCTTAATTAGGTGAATAATGGTAATTATTCACTATTCTTACCCATAATCATCGTACGTATTTTTTGCTCTTCAGTTTTGAATCTCACCACAAACTTGTTACTGTATTTGCATAATTTCGTAGCTCTGCGGGTGGATTCACGTGCCCACGGATTGGTATTTACGTAAGGCTAGGGAATTTCAGCAATATAAATTGCAAATCCTTGTTACCAATGTACGCAACAGGGTAAACACTAACGAAGGGTAGCTTGTACTGGCAGTGTTGGCTTGGTAAGGGTAAAGTCAGATCCTAGGTGCGGGCGATGAACTTTGTAGGAGATAACAAATTGGTTCTTCATTTTAAGTTGCTGTGTCATACGGCATGCCTTATATTGGTGAGCCTGCCCTCTTAAACAATGGAACTCCTTAGTCTCACACAACGAAACCTGGAGATCGCAACTTGAAAGATAAGAAAAAGTGGTTAAAGGGTTCCCTCTCTAAACTGTGCGTTGTTGAGGTAAAAGTGAGGCACCTACGGAGGCTGTGCCTCAAATGCGGGTGGGGAGCTCACAGGCGTGTGCTCTAGCCAACCAAACACCGTTCGGTTCTCGTGACTTGGAAGGTTAAAATAGTGGAAAATATCTTCTCATTAATCTTAGGTGTACATGGTTGTTACTTGCATTGTGCAGCCCACTTAATGTAGGCCCAAGGCCCCACGTTCTGATCTCCACCCATGACATTTGTAAAGCCTGTATGTACGTAAGGCACTCAAAAGTTTCTCTCTCAAAAAAAGGCACTCAAAAGTTGTTTCATATTTATTATTAACAATTACAGCTATTATTACATTCCCAGTCATATTACATATTTTCTCATGTCTATTCATAAAGTATCATTTCCATGTGACTCCAGATTGAATTATTATAATTTATGCGACACATATTTGGTGATACCATCTCATGTCTATTCATAAAGTATCATTTCCATGTGACTCCAGATTGAATTATTATAATTTATGCGACACATATTTGGTGATACCATCTTTAAGGCAATTCGATAGTTTGCGCACTGTCCGTTTGTTTTCTGTAGAACTGGTAGGTTGTTTGCATCAGGAGTCACCAACAATGATGTCATTCGTAAACCAGATTATGTTGATAACAATTAGAATGTTACCTGGCAAATTTTGTGTATCTTAGAAGTTATTGTATACACATGTTCTGTTCTATGAAGATTAATTCATCTCGGCCTCTTTTCAGTTTGTTAAGTGTTGCTTCAGAAAAGTTCTTTCGGTAATTGCAAGTCATATTACTTAATTTGTAACTCTTCATTGCAGGTTTCTCAAGGAGCTTGACTTACAAGGGAGTCAGGTGGAGTTTCGAGGCCGTCATTGGTTGAGTTGTTTCCCCAAGCCTTCGACATCATTAGAATCCTTGAATTTTGCTTGCTTGGATGGAGCAGTGAGTGCTAATGCATTGGAAAGTCTTGTTGCAAGGAGTCCAAATCTTAAAAGCTTAAGGTTAAATCGTGCAGTTCCACCAGCTGTTTTAGCCAAAATTCTTACTTCCGCTCCTAAGCTGGTGGATTTAGGTACAGGATTGGTTGCTCAAAGCAATAATGCTGGTGCACTCCCCAGTCTCTACAGTGCTATTCAGCAATGCACTTCTTTGAATAGTTTATCAGGCTTTTGGGATTCTTCACGTTGGATTACTCCAATAATACATTGTATTTGCAAGAACCTAACATGCTTGAACCTTAGCTATGCTCCAATGTTTCGGACAGTTGATCTTATTGGAACTATTCACCAATGTCAGAATCTCCGACACTTGTGGGTAAGGTTTAGTAGCACTGGTGTTATGGAAATTTAGCCTGTTTCTTATTATGTTAAATTACTAAATGCAGTTACTAGATCTTTACTTTGTTGGATGTTGTTCCACTTCAAGCCTATGAAATGAGATGTTTACGTATTTGGAAGTTCTGCTTTTGTCAACCCTGCTGTAGTGCAATTAGCAAGTAATGAAAATGTTGCCCAATATGCTGTTTTAAGTGGTACTTACTAGGCAAGAATTTTGTATATTTAACATACAAAATGCGTTTAGTTCAACTTCTTATGCTTTACTCTTTAACAGAGGCTGCATAAGAAGTTAAGAACACATGTTTATCTATTGACTTGTTTAACAAATTAGTTTGTACCAGTATAGACATTTGAAAATACCAGACCCTGCGAATGTGGAAGATATGATAATCTTAAAAATGTCAGCGAACTTTGGGAGTAGACCCTTTTTACCTTTCTTGGCATCTTCAGTAATGCTACAATATACTTTATAGTATGCATTTCTAGCCTTGCTGTGCACAAGGAAAAGTTGTAAGCATGAAAATTCTGTCAACGTGGTACTGTATTTTGAGAATGACTAAGACAAAATGATGATTTAGTAATAACTGATATGGGGTTCATTGATGTTAGGGAAACATTGTCTAATAGCTATTGTACTTAATTGTCAGTGTTACTTAATGCGCTCACCCACACAAACCACACACAAAGAGGTGACATACTTTTGGGTGTTTTAACTAGTATTGGAAGGTTACTTCAATAGTTATAGTGGAACACTTCTGATTAAAGGATTCTTAGAGTAAACCTTTGTTGTTCATATTGTTTCTTTCATTTGCGTTTCGTCCTTGCTGGTTGTTTCATGTTATAAAATGTTTTATTCACTAAGGCTTAATGGTTGTAGGTACTAGATCATATTGGCGATGCAGGATTGAAGGTTGTAGCCTCTTCTTGCCTGGAGCTGCAAGAGTTGAGGATATTTCCTGCGAATCCTGCGAATGCAAATGTGTTAGCAAGCACTGGTGTGACAGAGGAAGGGCTGGTTGCAGTATCTTCAGGCTGTCGGAAGCTAAACTCTGTGCTCTATTCCTGCAGTCGAATGACTAATTCTGCTCTGATTACGGTGGCAAAGAACTGCTCGCGAATCACGTCCTTCAGACTGCATATCTGCCTGCATGGGTCAGTAGATGCCGTGACAGGCCAGCCACTCGACGAGGGCTTTGGGGCAATTGTCCGGTCATGCAAGGGCCTCAGGCGTCTATCTATGTCTGGCCTTCTCACGGACAGCGTGTTCCTGTACATCGGCATGTACGCCGAGAGGCTGGAGACGCTCTCTGTCTCGTTTGCAGGAGATAGTGATGATGGCATGATCTATGTGCTCAACGGCTGCAAGAATCTCAGGAAGCTGGAGATCAGGAACTGCCCATTTGGCAACACGGCGCTTCTTGCAGGCACTCACAGGTACGAGGCGATGCGCTCGCTTTGGATGTCGTCGTGCGACATCACCCTGGGTGGCTGCAGGTCCCTCGCGGCAGCCATGCCAGGCCTCAACGTCGAGGTCATCTGCCAGGCGGATGGAGGCGCCAACGATGCAAAGAAGGTGGACAAGCTGTATGTCTACAGGACACTCGCCGGCCCGAGGGATGACGCCCCTGGATTCGTCTCGGCGCTGTAAATTACTTGTACCGTCGTAGTTGGTGGGTTTTCCTAGACCCTGCTATTTGTGTGAACTTAATTCGGCGCTGTCGCACTTTTATGCTACGTCTGCTACTCTGAAGCGGGCACCAGTTCTGTAAATTTGGTTAGTCTTCTGAACTGCAAATCTTAGCCCGGGCTGTGGCCAACACAGCATAATGCATATGGATCCAGGCCTTATTTCCAAGGTTGCACTGCAG

>TaCOI6-D Traes_5DL_4E8DD7C5F.3

CCGAGCACCCCCAGGTCAAGGTCTCCTCCCTCCGAGGAACGCCGCCTCCAAGGAACTCTCCTCTCACTCCACGGCCGTCGCCGTCCTCCTCCGCCGCCATCTTCTCCGCTCCGCTCGACTGGCGATCCGTCCGGCCGGACGGGGACCGGTTCCTTGCTGGTGACCAACCCTAACTTGCTTTTATTACTGTCCGGATGATTTCGATCTCGTCTTTTCTTTCCTTTATGTGATCAATCTAACCCAGACACTCGAATCCACCCGCGGATTCTTTTGCTTCCTGGTGAGCTGATGATAGATAGCAGCGAGTTCCTTTCCCCAAAAGAAAAACAAAAAATACAAGGAAATTTGGGGTTCCAGTCCAGCCGAACCCCACAGAGGACATCTTTCTGCGGTTCGGTCCGTCGATTTCGATCTTGGAGCTGACAACTTCTATCCCTTTCCCGGGTTTTGGATTCGTCTTCAGTGTTCTGATTTCTCAACGACCTTGGGTATTTTCTCTCAGTTTCATGGCGTGCTTTTTCTTCCGACTACTTTTCTATTTATAGAAAGACGCAAAATTTCATGTGACCGGTTGCTTAGTTTACTGTACTCTGTCCGGAACCCGCTCTAACTTGTGTTAATTCTTGGGAATTTATGATCAAATACTTCTTTGAGTGAGTATATAGCACATAGCAAAGAGTCGGGTAACAATTCAAGCAAATGGTCCTAAAGTGATACCCAGTCCTAACACAATGTTCCGTTTCTTGTGTGTGTGTGACTGTGAACAGGTCACAACCGGAAGCTGCAGAAAATAAGGGCGGCTTATTAGGATTCTGGGAGGCGGCAACATGCCCTACTTCCCAGATGAAGTGGTGGAGCACATCCTTGGCTTCGTATCGTCGCACCGTGACCGCGATGCTGCATCTCTCGTGTGCCACGCGTGGTACCGCATCGAGGGCCTCACTCGCCGCTCCGTGTTCATTTCCAATTGCTATGCGGTGCGCCCAGAGCGTGTGCACACACGTTTCCCCTGCCTGCGCTCACTGACCGTGAAGGGCAAACCACGCTTTGCTGACTTCAACCTTGTCCCTGCGGGGTGGGGTGCCTCAGCGGAGCCATGGGTGGATGCGTGTGCCCGTGCATGCCCTGGCCTTGAAGAGCTCCGCCTGAAGCGGATGGTTATCACTGATGGTTGCCTCAATCACCTTGCTCACTCATTTCCCAATTTGAGGTCACTAGTCCTTGTTAGCTGTGAGGGGTTCAGCACTGATGGCCTTGCTACTATTGCCACCAATTGCAGGTCAGAATGCTACAAGCTTGGAAGTCTTTTACTTTTCATGTTATGATAATGGTACTGAGTGTGGGTGTCTCCCATTAGACGTTGAATGTAAAATGGGTTATCACCAGCATCGCCTCATAAACTGCAATTCATATTCAACCTTCTTGCCATACCCAGTTTATCTCGATGCTCTACAATTTTGTACCAGATCATGGAAGCTTGTTTATCCTTGTACAATTGTTTGAATCTTGGATACCTAGAATGGAAACCGAGAAACTATCAATGACATTTAAATGATCCTACAGAAAAACCTTGATAAGTCCTTGGCAGTTGCCATTGCTCTATCTCCCTTTCCATGAGTTCTGATAAACAATAATGATTGAGATTGTGGATGCATTTCAGTTTATTAAAGTTAAGGGATGTTTCTTCAAACCTGTTTGTGAGATTGTGAATGCATTGCATACTGGGTCAATCCATGGTTCCAGTGTTATATAATCTTCATGACTGCATGTATACATTTCGAGATCTGATCAATATAAAAAAGTTTGGTATGCTGCACTTATATAAGTGGAATCTTGAGAGCTCTTTCATCAGTAACTTTAAAAGACATATGTGGACTGTCGGTGATTTACATATAGCAATGTGGCATTACCATAACATCGGAAAGAGAGGGAGGCACTAATGTAGTAATGTTGGTTATGTCATATCATTCCAATTTAAAGGAGGATTAGAATACTCAAGGGTTCTTTCACCAGTAATTTATTGCCAATTTCAAATATGCCTCTAGAGTCTCTCGACCCTCCCATCAATATAATCGAAGCATGGATTTCGAGTTGCGCGACTAGTCGTCGACTAGTCGATGAGTCGCAAAAAAATGTCGACTCAACTTCGTGTCGACTCGGGTCGTGAGTCGCGACTAGTCGCAACTGCAGGCTCGACAAGCGCTCGCCTTTTTTTTTAAGAAAACTGTCGGAAACCCTTACAGTATACATAGGAAATTAGGAATATAAATTCACGTCCATGAGGACTTGAACCTAGGTGGTGGGTTGTACGTCCACTCCCATAGCCTCTTGAAGGGCTCACATTTGAAGTGCTACCAACTCTTAATTAGGTGAATAATGGTAATTATTCACTATTCTTACCCATAATCATCGTACGTATTTTTTACTCTCAGTTTTCAATCTCACCACAAACTTGTTGCTGTATTTGCATAATTTGGTAGCTCTGCGGGTGGATTCACGTGCCCACGGATTGGTATTTACTAAGGCTAGGGAATTTCAGCAATATAAATTGCAAATCCTTGTTACCAATGTATGCAACAGGGAAACACTAACGAAGGGTAGCTTGTACTGGCAGTGTCGGCTTGGTAAGGATAAAGTCAGATCCTAGGTGCGGGCAATGAACTTTGTAGGAGATAACAAATTGGTTCTTCATTTTTAGTTGCTGTGTCATACGGCATGCCTTATATTGGTGAGCCTGCCCTCTTAAACAATGGAACTCCTTAGTCTCACACACAACGAAACCTGGAGATCGCAACTTGAAAGATAAGAAAAAGTGGTTAAAGGGTTCCCTCTCTAAACTGTGCGTTGTTGAGGTAAAAGTGAGGCACCTACGGAGGCTGTGCCTCAAATGCGGGTGGGGGAGCTCACAGGCGTGTGCTCTAGCCAACCAAACACCGCTCGGTTCTCGCGACTTGGAAGGCTAAGATAGTGGAAAATATCTTCTCATTAATCTTAGGTGTACATGGTTGTGACTTGCATTGTGCAGCCCACTTAATGTAGGCCCAAGGCCCCACGTGCTGATCTCCACCCATGACATTTGTAAAGCCTGTATGTACGTAAGGCACTCAAAAGTTGTTTCATATTTATTATTAACAATTACAGCTATTATTACATTCCCAGTCATATTTACATATTTTCTCATGTCTATTCATAAAGCATCATTTCCATGTGACTCCAGATTGAATTATTATAATTTATGTGACACATATTTGGTGATACCATCTTTAAGGCAATTCAATAGTTTGCGCACTGTCCGTTTGTTTTCTTTAGAACTGGTAGGTCGTTTGCATCAGGAGTCACCAACAATGATGTCATTCGTAAACCAGATTATGTTGATAACAATTAGAATGTTAATACCTGGCAAAATTTTGTGTATCTTAGAAGTTATTTTCTCATACACATGTTCTGTTCTATGAAGATTAATTCATCTCAGCCTCTTTTCAGTTTGTTAAGTGTTGCTTCAGAAAAGTTCTTTCAGTAATTGCAAGTCATATTGCTTAATTTGTAACTCTTCATTGCAGGTTTCTCAAGGAACTTGACTTACAAGGGAGTCAGGTGGAGTTTCGAGGCCGTCATTGGTTGAGTTGTTTCCCCAAGCCTTTGACATCATTAGAATCCTTGAATTTTGCTTGCTTGGATGGAGCAGTGAGTGCTAATGCATTGGAAAGTCTTGTTGCAAGGAGTCCAAATCTTAAAAGCTTAAGGTTAAATCGTGCAGTTCCACCAGCTGTTTTAGCCAAAATTCTTACTTCCGCTCCTAAGCTGGTGGATTTAGGTACAGGATTGGTTGCTCAAAGCAATAATGCTGGTGCACTCCCCAGTCTCTACAGTGCTATTCAGCAATGCACTTCTCTGAATAGTTTATCAGGCTTTTGGGATTCTCAACGTTGGATTACTCCAATAATACATTATATTTGCAAGAACCTAACATACTTGAACCTTAGCTATGCTCCAATGTTTCGGACAGTTGATCTTATTGGAACTATTCACCAATGTCAGAATCTCCGACACTTGTGGGTAAGGTTTAGTAGCACTGGTGTTATGGAAATTTAGCCTGTTTCTTATTATGTTAAATTACTTCATGCAGTTACTAGATCTTTACTTTGTTGGATGTTGTTTCACTTCAAGACTATGAAATGAGATGTTTACGTATTTGGAAGTTCTGCTTTTGTCAACCCTGCTGTAGTGCAATTAGCAAGTAATGAAAATGTTGCCCAATATGCTGTTTTAAGTTGTACTTACTAGGCAAGAATTTTGTATATTTAACACACAAAATGCATTTAGTTCAACTTCTTATGCGTTACTCTTTAACAGAGGCTGCATAAGAAGTTAAGAACACACGTTTATCTGTTGACTTGTTTAACGATCTAGTTTGTACCAGTATAGACATTTGAAAATATCAGACCCTGCGAATGTGGCAATATATGATAATCTTAAAAATGTCAGCGAATTTTGGGAGTAGACCCTTTTTACCTCTCTTGGCATCTTCAGTAATGCTACAATATACTTTATAGTATGCATTTCTAGCCTTGCTGTGCACAAGGAGAAGTTGTAGGCGTGAAAATTCTGTCAACATGGTACTGTATTTTGAGAATGACCAAGACAAAATGATGATTTAGTAATACTCCCTCCGTCCCAAAATTCTTGTCTTAGATTTGTCTAGATACGGATGTATCTAACACTAAAACATAACTAGATACATCCGTATTTAAACAAATTTAAGACAAGAATTTTGGGACGGAGGGAGTAACTGATATGGGGTTCATTGATGTTAGGGAAACATTGTCTAATAGCTATTGTACTTAATTGTCAGTGTTACTTAACGCGCTCACCCACACAAAGAGCTGACATAATTTTGGGTGTTTTAACGTCTCTTTGTTGGGTGTTTCTAATCCCAGAGAAGGTCCTTGTGTGGGTGTTTTAGGCCTTGTACAATGCAAGGTGCTTAGGGGAGGTGCTTAGTAAAATAATCCGAGCTTTTCTCAAGCACCAGTGCTTATTTCTGTAGGAGGGGTGCCTAATTAAGCGTCTACCCTCTATAAATAAGCGCCGGTGCTTAAGAAAAACCAATTTATTTTTCTAAGCACCACCTCTAAGCACCTTGCATTGTACAAAGGCCTTAACAGCTCTGTAACTAGTGTTGGAAGGTTACTTGAATAGTTAGAGCAGAACACTTCTGATAAAAAAATTCTTAGAGTAAACCTTTGTTGTTCATATGTTTCTTTCATTTGCGTTTCGTCCTTGCTGGTTATTTCATGTTATAAAATGTTTTATTCACTTCGGCTTAATGGTTGTAGGTACTAGATCACATTGGCGATGCAGGATTGAAGGTTGTAGCCTCTTCTTGCCTGGAGCTGCAAGAGTTGAGGATATTTCCTGCGAATGCAAATGTGTTAGCAAGCACTGGTGTGACAGAGGAAGGGCTGGTTGCAGTATCTTCAGGATGTCGGAAGCTAAACTCTGTGCTCTATTCCTGCAGTCGAATGACTAATTCTGCTCTGATTACGGTGGCAAAGAACTGCTCGCGAATCACGTCCTTCAGACTGCATATCTGCCTGCATGGGTCAGTAGATGCCGTGACAGGCCAGCCACTGGACGAGGGTTTTGGGGCAATCGTCCGGTCATGCAAGGGCCTCAGGCGTCTATCTATGTCTGGCCTTCTCACGGACAGCGTGTTCCTGTACATCGGCATGTACGCCGAGAGGCTGGAGACTCTCTCTGTCGCGTTTGCAGGAGATAGTGATGATGGCATGATCTATGTGCTCAACGGCTGCAAGAATCTCAGGAAGCTGGAGATCAGGAACTGCCCATTTGGCAACACCGCGCTTCTTGCAGGCACGCACAGGTACGAGGCGATGCGCTCGCTTTGGATGTCGTCGTGCGACATCACCCTGGGTGGCTGCAGGTCCCTCGCGGCAGCCATGCCAGGCCTCAACGTCGAGGTCATCAGCCAGGCAGATGGAGGCGCCAACGATGCAAAGAAGGTGGAGAAGCTGTATGTCTACAGGACACTCGCTGGTCCGAGAGATGATGCCCCTGGATTCGTCTCGGCACTGTAAATTACATATACTGTCGTGGTTAGTGGATTTCTATTTGGCTTGTAATTTTGTACCGTGCCTGAACCGTGGAAGAATCTGCACGTGCTAAGACTATAATTTCGATCATCCTGCTATTTCACTTACCATGTCGATGGAAGCAGTGTATGCTACATTTGTTGGTGATCAGATTCTGTGTTCACAGATGCATTTGATACAGAATGCAATAGGCAATTAAAATGTGAC

>TaCOI7-A Traes_6AL_0F53490CA.1

GGCGCCTACGTCTCCCCCTGGGTCGCCGCGCTCGGCCCCGCCTACCCGCGCCTCGAGCGCATCTGCCTCAAGCGGATGACCGTCTCCGACGACGAGCTCGCGCTCATCCCCAAGTCCTTCCCGCTCTTCAAGGAGCTCTCGCTCGTCTGCTGCGACGGCTTCACCACCCGCGGCCTCGCCGTCATCGCCGAGGGCTGCCGGTACGCTTCCCTTCCCTTTCTCGTCTTTGGGTATCAACGAACGTATCAACTTTACGGGCTGGGACAGTATCGTACTGCCATTGGCGCATTTGTTCATTTCTCAGCTGCATCATGCCATTCCCCGCTTTATTTAATGTGCACGCCTACTCCTTTATGCTTTTTTCGGTTTTATTGGTCCGACTTTGCCCTGGATCAGAATCTGATCTAGGCTACACTAGCTTTTATTGCCGCGCCGGGTTCTCATCCCTACACGTTCTCTCAAAGCTAGATCTTTATTGTTCTTGAGCTCTACTCAGTTCGTGACACTTGGTAGTGCCACACTCGAATCTTTGCCGTTAGTAGCACTCAACTTTTGATCGTTTTCTTGTCATGGTTATGGCAAATAGTTCTAGCAAACAAATGTCTCAAACTATGATATACTAGTACTCAAGAGTGGTATCATCTTATGGATCGTTGCATTTATTTTTTAGGCATCTTCGGGTACTGGATCTGACTGAAGATTATTTCCATGAGGAGGAGAGCGAGGTGGTGGATTGGATCTCCAAGTTTCCAGAGAGCAACACGTCGCTGGAATCGCTTGTATTTGATTGTGTTAGTGTCCCATTCAACTTTGAGGCCCTGGAGGCACTTGTTGCACGCTCACCAGCTCTGNTCTGCGTCGTCTGCGCGTCAATGATCATGTGTCGATAGAGCAGTTGCGTCGTCTCATGGCAAGGGCACCCCATCTAACTCACCTTGGCACTGGATCATTCCGATCTGAGCCAGGCCCTGGGGGTGCTTTGTCTGTGTCTGAGCTCGCTACCTCTTTCGCGGCGTCCAGATCACTTGTTTGTTTGTCAGGTTTCTTGGATGCCAATGCAGCATACCTCCCAGCAATCTACCAAGTTTGTGCCAATCTCACTTCCCTCAATTTTAGCTTTGCGGGTCTAACAGATGAAGAGTTCATACCAGTTATTCGCCATTGCATCAATCTTCGCACTTTATGGGTAAGTATTTTGTGATTTCATTTGTCGTCACATTGATAGATATATTGGTTTATGTACTTTGACATCAGGTGTTGGTGTTCCAATTTCTTATTGTAGGTTCTTGATACTGTGGGTGATGAAGGCCTTAGGGCTGTGGCTGAAACATGCTCAAATCTCCGTGAGCTACGTGTTTTTCCTCTGGATGCCACCGAGGATTCTGAGGGCTCAGTCTCAGATATTGGTCTCCAGGCAATCTCAGAAGGCTGCCGAAAGCTCGAATCAATTCTCTACTTTTGCCAGCGCATGACAAATGCAGCAGTAATTGCTATGTCCGAGAACTGCCCTGACCTTTTGGTGTTCCGCCTCTGTATTATGGGCCGCCACCGCCCTGATCGGATTACCGGGGCGCCCATGGATGAGGGTTTTGGGGCGATTGTGATGAACTGCAAGAAGCTCACCAGACTCTCAGTCTCTGGCCTGCTCACTGATAAGGCGTTTGCATACATTGGGAGACACGGAAAACTCATAAAGACTCTGTCTGTTGCCTTTGCTGGGAATAGTGACATGTCTCTTCAGCATGTGTTTGAGGGGTGCACTAGGTTGCAGAAGCTCGAGGTCAGAGATAGCCCTTTTGGCGACAAAGGATTGCTCTCTGGCCTGAACTATTTTTACAACATGAGGTTCTTTTGGATGAACTCATGCAGGCTAACTGTGAAGGGTTGTGGGGATGTAGCTCAGCAAATGCCTAATCTGGTGGTTGAAGTAATGAAGGAAAATGAAGGGGAAATGGATACCGTTGATAAGCTGTACCTGTATCGATCGTTGGCAGGACCAAGGGAAGATGCTCCATCATTTGTCAACATCTTGTAGTACTGCCATTGAAGAAGTTTGTTGTACGGTCATGTTTGTTCTACTATTCCCGATCATTGCTGTTGTATTCCTGTTCAAGTATTCCAGAGCCAGAGAGGAAGCTCCCCGCGGATGATTTTGAATTGTGGGGAATATGAATGCATCATTTAATTAAGCAAACCATCAATTATTCTTCTCTGGGCTGATTGTGTCGAGACTTGAACCTGTACGAGGTTTCTTTGATGTATGTACCTTGGTGAGGCATCTCATCTTGTGAGTCACTGTATTCCCAATGGAATCTGAGTTATTTTCCTGGCCAGCACCAGACTGATGCCTTTGTTGTACCGTAGTTGAGTTGTCGGAACCGATTAGTGATTGCACAGAGATTGGCTGCAGGTTGACTCCAATTCGGCAGCAGAGATTTTACATGGTTCCTTTTGCCCTCAAGTCTCCAGCCATTGATGTTTTGCCACGTACTGTTCAGTCTATCTGCTCAGACGAAGGAACGGCATTATCTATGTTGCAATGTTCCATCAACTTTTTTGCCTTGTATTTTAGATTATCCCAGCTACGATAAATTTTCTCTTCCCATTTTTTATAGTTTCCACAACTTATTTTTGTAGATCAATGTAGACCACTATCACTTTTCCTGGTCACAACCTTCTGCTGTTATTAATCAAGAAGATATTGGATGATCAAATTCAGTCAGCATCAATTTGTTACCACGTTCTCC

>TaCOI7-B Traes_6BL_9E82CDBD1.1

GTTCACTTCTCAGCTGCATCATGCCATTCCCACTTTATTTAATGTGCACGCCTACTCCTTTATGCTTTTTTCGGTTTTATTGGTCCGGCTTTGCCATGGATCAGAATCTTATCTAGGCAATAGCTTTTATTGCCTTGCTGGGTTCTCATCCCTACACGTTCTGTCAAAGCTAGATCTTTATTGTTCTTGAGCTCTACTCAGTTCGTGACACTTGGTAGTGTCACACTCGAATCTTTGCCGTTACTCGCACTCAGCTTTTGATAGTTTTTCTGTTATGGTTATGGCAAATAGTTCTAGCAAACTGTGATATACTAGTACTCAAGTGTGTATCTTATGGATCGTTGCATCTATTTTTTTAGGCATCTTCGAGTACTGGATCTGACTGAAGATTATTTCCATGAGGAGGAGAGTGAAGTAGTGGATTGGATCTCCAAGTTTCCAGAGTGCAACACGTCGCTGGAGTCGCTTGTATTTGATTGTGTTAGTGTCCCATTCAACTTTGAGGCCCTGGAGGCACTTGTTGCACGCTCACCAGCTCTGCGTCGTCTTCGCGTTAATGACCATGTGTCGATAGAGCAGTTGCGTCGTCTCATGGCAAGGGCACCCCATCTAACTCACCTTGGCACTGGATCATTCCGATCTGAGCCAGGCCCTGGGGGTGCTTTGTCTGTGTCTGAGCTCGCTACCTCTTTCGCAGCGTCCAGATCACTTGTTTGTTTGTCAGGTTTCTTGGATGCCAATGCAGCATACCTCCCAGCAATCTACCAAGTTTGTGCCAATCTCACTTCCCTCAATTTTAGCTTTGCGGGTCTAACTGCTGAAGAGTTCATACCAGTTATTCGCCACTGCGTCAATCTTCGCACTTTATGGGTAAGTATTTTGTGATTTTTTTTGCCGCCACATTGATAGATATATTGGTTTATGTACTTTGACATCAGGTGTTGGTGTTCCAATTTCTTATTGTAGGTTCTTGATACTGTGGGTGATGAAGGCCTTAGGGCTGTGGCTGAAACATGCTCAAATCTCCGTGAGCTACGTGTTTTTCCTTTGGATGCCACCGAGGATTCTGAGGGCTCAGTCTCAGATATTGGTCTCCAGGCAATCTCAGAAGGCTGCCGAATGCTTGAATCAATTCTCTACTTTTGCCAGCGCATGACAAATGCAGCAGTAATTGCTATGTCCGAGAACTGCCCTGACCTTTTGGTGTTCCGCCTCTGTATTATGGGCCGCCACCGCCCTGATCGGATTACCGGGGCGCCCATGGATGAGGGTTTTGGGGCGATTGTGATGAACTGCAAGAAGCTCACCAGACTTTCAGTCTCTGGCCTGCTCACTGATAAGGCGTTTGCATACATTGGAAGACACGGAAAACTCATAAAGACTCTGTCTGTTGCCTTTGCCGGGAATAGTGACATGTCTCTTCAACACGTGTTTGAGGGGTGCACTAGGTTGCAGAAGCTTGAGGTCAGGGATAGCCCTTTTGGCGACAAAGGATTGCTCTCTGGCCTGAACTATTTTTACAACATGAGGTTCTTTTGGATGAACTCATGCAGGCTAACTGTGAAGGGTTGTGGGGATGTAGCTCAGCAAATGCCTAATCTGGTGGTTGAAGTAATGAAGGAAAATGAAGGGGAAATGGATACCGTTGATAAGCTGTACCTGTATCGATCGTTGGCAGGACCAAGGGAAGATGCTCCATCATTTGTCAACATCTTGTAGTACTGCCATTGAAGAAGTTTGTTGTATGGTCATGTTTGTTCTACTATTCCTGATCACTGCTGTTGTATTCCTGTTCAAGTATGCCAGAGCCAGAGAGGAAGTTCCCTGCGGATGATTTTGCGGGAAATATGAATGCATCATTTAATTAAGCAGATCATCAATTATTCTTTTCTGGGCTGATTGAGTCGAGACTTGAACCTGTACGAGGTTTCTTTGATGTATGTATCTTGGTGAGGCATCTCACCTTATTCTCAATGGAATATGAGTTATTTTTCTGGCCAGCACCAGACCGATGCCTTTGTTGTATCATAGTTGAGTTGTCGGAACCGATTAATGATTGCACAGAGACTTGCTGAAGGTCGGCTCCAATTCGGCAGCAGATTTTACATGGTTCCTTTTGCCCTCAAGTCTCCAGCCTTTGGTGTTTTGCCATGTACTGTTCAATCTATCTGCTCAGACGAAGGAACGGCATTATCTATGTTGGAATGTTCCATCAACTTTTTTGCCTTGTATTTTAGATTATCCCAGCGACGATAAATTTTCTCTTCCCATTTTTTTATAGTTTCCACAACTTATTTTTGTAGATCAATGTAGACCACTATCACTTTTCCTGGTCACAACCTTCTGCTGTTATTAATCAAGAAGATATTGGATGATGAAATTCAGTCAGCATCAATTTGTTATCACGTTCTCCTTGCTTTCTTGCCTGTTCATTTGCCATGATCATGCTACTTTGATTATGACTGCTCTGTTGCTACCACCATAGCAAATAAATGCCCTCTGCCTGTGTATTGGCACATGATAAGGACATGACCTACTAATGATTTGATCTCAAGAGTGGTCCTTGGTCCTTGTTTCGTGTAGATCTGCATGACATCTACTGTCAGATAGGGATTTGCCTTTTCATCCTTCTCCTGTGTACATCCCTGGAGGCGTCAAAAGCTCAAAGCTCCCAATTGGCAGAGTTGCACCTTCCACAAGGCAAGAGGAAAAAGGTAATGGTTTTACTTACTGACATAGTAACATGTACGCTTATGTTCCAAAATAATTGGCTAGACAAGGTGTGTGCTGATGATTTTTAGATCTCTGTCTTCTCTGTGGGGCTGGTTATCACTGGATTCTAGCTTGTTATGACTAATCTGACTCTTATCCTGGTTCCAAAAATACCCACAACTTCAATCAAAGCCAGATATTTGTGCGCAAAAAGGTTTCTTCAGCTTCTGTTGAAGCATTGCCCAGTTGGTGCTAATTCAACGGTCACTACTCCCTCACTTTACTGGGAGTACTAAATATTCATCACATATCCTTAAGCATCT

>TaCOI7-D Traes_6DL_AD0DAD6D1.2

GTCCGGCCTTGCCCTGGATCAGAATCTGATCTAGGCCATAGCTTTTTATTGCCGTGCTGGGTTCTCATCCCTACACGTTCTGTCAAAGCTAGATCTTTATTGTTCTTGAGCTCTACTCAGTTCGTGACACTTGGTAGTGCCACACTCGAATCTTTGCCGTTAGTGGTACTCAACTTTTGATCGTTTTCTTGTTATGTTATGGCAAATAGGTCTAGCAAACAAATGTCTCAAACTATGATATAGTAGTACTCAGGTTGTGTTATCTTATGGATCGTGACATTTATGTTTTAGGCATCTTCGAGTCCTGGATCTGACTGAAGATTATTTCCATGAGGAGGAGAGCGAGGTGGTGGATTGGATCTCCAAGTTTCCAGAGTGCAGCACGTCGCTGGAATCTCTTGTCTTTGATTGTGTTAGTGTCCCATTCAACTTTGAGGCCCTGGAGGCACTCGTTGCACGCTCACCAGCTCTCCGTCGTCTGCGCGTGAATGACCATGTCTCGATAGAGCAGCTGCGTCGTCTCATGGCAAGGGCACCCCATCTGACTCACCTTGGCACTGGATCATTCCGATCTGAGCCAGGCCCTGGTGGTGCGTTGTCTGTGTCTGAGCTCGCTACCTCTTTTGCGGCGTCCAGATCACTTGTTTGTTTGTCAGGTTTCTTGGATGCCAATGCAGCATACCTCCCAGCAATCTACCAAGTTTGTGCCAACCTCACTTCCCTCAATTTTAGCTTTGCGGGTCTAACTGATGAAGAGTTCATACCAGTTATTCGCCACTGCGTCAATCTTCGCACTTTATGGGTAAGTATTTTGTGATTTTTTTTGTCGTCACATTGATAGATATATTGGTTTATGTACTTTGACATCAGGTGTTGGTGTTCCAATTTCTTATTGTAGGTTCTTGATACTGTGGGTGATGAAGGCCTTAGGGCTGTGGCTGAAACATGCTCAAATCTCCGTGAGCTACGTGTTTTTCCTCTGGATGCCACCGAGGATTCTGAGGGCTCAGTCTCAGATATTGGTCTCCAGGCAATCTCAGAAGGCTGCCGGAAGCTTGAATCAATTCTCTACTTTTGCCAGCGCATGACAAATGCAGCAGTAATTGCTATGTCCGAGAACTGCCCTGACCTTTTGGTGTTCCGCCTCTGTATTATGGGCCGCCACCGCCCTGATCGGATTACCGGGGCGCCCATGGATGAGGGTTTTGGGGCGATTGTGATGAACTGCAAGAGGCTCACCAGACTTTCAGTCTCTGGCCTGCTCACTGATAAGGCGTTTGCATACATTGGAGGACACGGAAAACTCATAAAGACTCTGTCTGTTGCCTTTGCCGGGAATAGTGACATGTCTCTTCAGCACGTGTTTGAGGGGTGCACTAGGTTGCAGAAGCTTGAGGTCAGAGATAGCCCTTTTGGCGACAAAGGATTGCTCTCTGGCCTGAACTATTTTTACAACATGAGGTTCTTTTGGATGAACTCATGCAGGCTAACTGTGAAGGGTTGTGGGGATGTAGCTCAGCAAATGCCTAATCTGGTGGTTGAAGTGATGAAGGAAAATGAAGGGGAAATGGATACCGTTGATAAGCTGTACCTGTATCGATCATTGGCAGGACCAAGGGAAGATGCTCCATCATTTGTCAACATCTTGTAGTACTGCCATTGAAGAAGTTTGTTGTACGGTCATGTTTGTTCTACTATTCCCGACCATTGCTGTTGTATTCCTGTTCAAGTATGCCAGAGCCAGAGAGGAAGCTCCCCGTGGATGATTTTGAATTGTGGGGAATATGAATGCATCATTTAATTAAGCAGGCCATCAATTATTCTTTTCTGGGCTGATTGCGTCGAGACTTGAACCTGTACGAGGTTTCTTTGATGTATGTATCTTGGTGAGGCATCTCATCTTGTGAGTCATCGTATTCCCAATGGAATCTGAGTTATTTTCCTATGCCTTTGTTGTACCATAGTTGAGTTGTCGGAACTGATTAGTGATTGCACAGAGAATGGCTGAAGGTCGACTCCAATTTGGCAGCAGATTTTACATGGTTCCTTTTGCCCTCAAGTCTCCAGCCATTGATGTTTTGCCATGTACTGTTCAATCTATCTGCTCAGACAAAGGAACGGCATTGTCTATGTTGGAATGCTCCATCAACTTTTTTGCCTTGTGTTTAGATTATCCCAGCTACGATAAATTTTCTCTTCCCATTTTTTATAGTTTCCACAACTTATTTTTGTAGATCAAGGTAGACCAATATCAACTTTTCCTGGTCACAACCTTCTGCTGTTATTAATCAAGAAGATATTGGATGATGAAATTCAGTCGGCATCAATTTGTTACCACGTTCTACTCTACTTCCTTTGTTGCCTGTTCATTTGCACCCATGATCATGCTGCTCTGATTATGACTGCTCTGTTGCTACCACCACAGCAATAAATGCCCTCTGCCTGTGTATTGGCACATGATAAGGACATGACCTACTAATGATTTGATCTCAAGAGTGGTCCTTGGTCCTTGTTTCGTGTAGATCTGCATGACATCTACTGTCAGATGGGGATTTGCCTTTTCATCCTTCTCCTGTGAACATCCCTGGAGGGGCCAAAAGCTCAAAGCTCCCAAATGGGCAGAGCCTGCACCTTCCACACGGCAAGAGAAAAAAGGTAATGGTTTCACTCCCTGTCATAGTGGCATGTACTCTTATGATCCAAAATAATTGGCTAGACAAGGTGTGTGCTGGGGATATTTAGATCTCTGCCTTCCCTGTGGGGCTGGTTATCACTGGATTCTAGCTTGTTATGACTAATCCAACTCTTGTCCTGGTTCCAAAAACACCCACAACTTGAATCAAAACCAGATATTTGTGTCCAAAAAAGTTTCATTAGCTTTTGTTGAACCATTGCCCAGTTGGTGCTAATTCAACATACACTACTCGCTAACTTTATTGGCAGTACTACTAAATATCCATATATCCTTAAGCATCTTATGAAGTCAGACCTGATGTAACCCAAGTCTAGATAAACACAAACCACAGTTCTTGTCCATGTGCAGTGGACTCTTATCCAAAATTCTCTGCTTTTTTCCTCTCAACTCAATCGGTCGCGGAGTAGGAGCCACAATGATTTTGTTCCTAAACCCTTTTAGGTTTACTTGGTAGTTTGGCAGTGTCAACCATTAATCTATCCTCAGCATCTTATAAGTCAGCCTTAACACTACGCAAACACAATTCACCTTTTCTTGTCGATGCGACACGAGGCCCTTATCCACTAATTGTTTCTCCCTGCCTTTTGCTCGACTGAGAGCCGGAAAATCAACCGGGGAGTAGGAAAACAGTGTAGCCATGATCATTGTGCTCCTAAACCTTTTTAGGTTTCCCTGGTAGTGTCAACTGTTAAATCTATCCCCAACACTATGTAAACACAAGGCACCTTTTCTTGTCTAATGTGAAATCAAATGAGATCTTTATCCATTAATTTTCTAGAATTTCCTCAACTCGACAACTCGAAGCCAGGAAATCATTTGGGAAGTAGAAAAACTGTGTCACCATGATCATTTTGTTCCAAAATCCATGCAGGTTTACTTACCAAACGAACAAATGTTGCTGACATGATATTTGCTCCTATATCTTCCTTGATCGTGCGTAAATATCCACTGCTCTCTCCGTAAAGAAAATATAAGAGCGTGGGGTTGGGTTTTCTCCCTCGGCTAAAGCTGTGGATGAAAACCAGAACCCTGTTCTTCCATGATATTTCCACCATATTGATTCTCCAAAAGAAAGAAATGATACGTCGTCGAAAGATCACGTCAACTTCTCCAATGTTCTCTTACGGCACGTCGCATATCCAACTCGCTTTTCACTTTGTAGTATGATAGGGAAAGAAAAATCTCGCAGGTAGCCATCCGAAAATCAAGAGCGCAAATCTTTT

>TaCOI8-A Traes_7AS_78FEAE00C.1

CTTCAGTTTATAAAGGAGAGGAGTCGAGTGTGGCATCTAATCGACTGATGTACTTAATACTTGTGTAGGCACCTGAGGGTTCTGGATCTGATCGAGGACTATGTGGATCAGGAGGACGAAGCAGTGGACTGGATCTCCAAGTTCCCAGTGTCAAACACATCCCTAGAATCCCTCATGTTTGATTGCGTTGCTGTCCCATTCAACTTTGAGGCCCTAGAGGCCCTTGTGGCACGCTCACCCTCTCTTCGCCGGCTGCGTGTGAACCACTACGTTTCGGTGGAGCAGCTACGCCGTCTCATGGCACGGGCTCCGCAGCTCACACACCTTGGCACTGGGGCCTTTCGTCCTGAGGCTCCACAAGGTGGAGGCATGTCGGTGTCTGAACTCGCACCCTCTTTTGCAGCCTCAACGTCTATAGTCTGCCTCTCTGGGTTTCAGGAGGTCAACCCTGAATACCTTCCAGCAATCTACCCAGTGTGTGGTAATCTCACCTCCCTTAACGTTAGCTTCGCGAGCCTAACTGCTGAGGATCTGACACCAGTCATTCGCCAATGCCACAAACTTCAGACATTCTGGGTAGGTCCATACTCTTATTTGTTTTACCATGGTAGTCTAATTCATGTTACTTTTTCTTTTCATTTGCACTAAACTGCATCTGGTTTAGGTTCTTGATACTGCGGGTGATGAATGCCTCAGGGCTGTGGCTGAGACATGCTCTGATCTTCGTGAGCTGCGAGTATTTCCGCTGGATGCCACCGAAGACTCTGATGGGTCTGTGTCAGATGTTGGGCTTCAGGCTATATCCGAAGGTTGCCGAAAGCTTGAATCCATACTTTACTTTTGCCAGCGGATGACAAATGCTGCGGTCGTAGCTATGTCCAATAACTGCCCTGACCTTGTGGTATTCCGTCTTTGCATCATGGGTCGCCACCGCCCTGATCGTATTACTGGGGAGCCCATGGATGACGGCTTCGGCGCGATAGTGAAGAACTGCAAGAAACTTACTAGGCTCTCAGTCTCTGGCCTGCTCACTGATAAGGCATTTGCATACATTGGGCAATATGGCAAACTAATAAAGACTTTGTCTCTTGCCTTCTCGGGGAACAGTGACCTGTCGCTTCAGTTTTTGTTCGAGGGATGCACTCGGTTACAGAAGCTTGAGGTCAGAGATAGCCCATTCAGTGATAGGGGACTGCTTTGTGGTTTGGATTATTTCTACAACATGAGGTTCCTGTGGATGAATTCATGCAGGCTAACCATGAGGGGTTGTAGAGAGGTAGCTCAGCGGATGCCTAACTTGGTTGTTGAAGTAATGGAGGAGCAAAATGAGGATAAGGTGGAAACAGAGACTGTTGATAAATTGTACCTGTACCGCTCACTAGCAGGGCCAAGGGGTGATGCACCGCCATTGGTGAAAATTTTGTAGTCATCGCTTATTTTGTGGGAGTTTGGTGGTGATCCGAAGCAGAACTTTTCTTGTCCTACTGGACCTGCTCATCCTTGTTCTGTTATTCCTGAACGAGAGCTGGTCGTTTTGTGTTCTTGATCAAGTTAACTGGAGCCAGTGGACGGAAGTTTCCTGTGGAGGGTTTTCATGATTTCACAGGACGCAACAATGTATTATTCACTGGACCGAAGGAGCACCGGCATTGCTTACGCGTCGCAACGAATTCACAATTGTTCTCATGATGAGGCGGCATTTGCTGTTGAAGCAGTTTGGGTACCTCACTACATGTGTATCATCTTGATCAGCATCTGCTATGCCGGGAGCAGTTGAAGACAAGGGAATCAGATTCCGGCCTCGCGTCGATCCCAGTATTGCAGCACAGATGAGCAGGGGCTGATTGCACTCAAGAGTCCAGTTAGCGCTTCTGTTTCATCCATCCAGACAGAGGAGTGGGCATGCCAATGTGAATTCCACTCTGCTACCATCCATGTACTTGTGCTTAGAAGATGACTATGGTAAAAGAAAGCCTCCCCTTTGTTTGTACTTTGTAGCCTCCCTCGAGACATTGTTGTTTTTGTAGACCATTATTATTATTATCTCTTGTTACATTCAACTTTGCCTCGCCTTTATTATGCTGAGAACTACCCAAGGAGTTGAAACTCTATTATCAGCAAATTTTGTTCTTTC

>AetCOI1 EMT18237

ATGGGGTGCGTGGAGGACCCCTGGGACCGCGACGCCATCTCGCTCGTCTGCCGCCACTGGTGCAAGGTCGACGCGCTCAGCCGCAAGCACGTCACCGTCGCCATGGCCTACTCCACCACCCCCGACCGCCTCTTCCGCCGCTTCCCCTGCCTCGAGTCGCTCAAGCTCAAGGCCAAGCCCCGCGCCTCCATGTTCAACCTCATCCCCGAGGACTGGGGCGGCTCCGCCTCGCCCTGGATCCGCGAGCTCTCCGCCTCCTTCCACTTCCTCAAGGTGCTGCACCTCCGCCGGATGATTGTCTCCGACGACGACGTCGCCGTGCTCGTGCGCGCCAAGGCCCACATGCTCGTCTCCCTCAAGCTTGACCGCTGCTCCGGCTTCTCCACCTCCTCCCTCGCTCTCCTCGCCCGCTGCTGCAAGTAATTTCTCCCCTCCTTGTTCCGGTTCACAAATGATATGCATGCATTAGTTCGTTTATTAATTACTGCGGTATCTCTTTCTTGTCCTACGAATTAATTTCTAGTTTCAATTGTTCTAGTTAGTTTTTCTCTTCAGAAATTGTGTTTTGTTCTATTTGGAAAGGGTTGGTGGATGGGATGTTTGTAAAGCGAGTATAGATAAGATGTTCTTTTGTTCATTTCGAAAGGGGAAAAGTAAGTGTAACGTCTCACCTTTTCTTGATGTAAAACATCATGCTACTCTTTGTAATTTGGGAATAATTCTAGCTTTGGAGCAGAAATTAGGAAATGTGGACTTTAACTGCCTTACTGAGTTGGGGACTTTGCTTAGTTGTTTATACTTTCTTAGAGAACTATGATACTTGGTGGAGATAGTTTTGAAAAGCAGATTATCAAAATGCTGTTGTAGAAGTTTAGTAAGAATGTAAGAGCATACATTCATAGGTTACTTGTTTAAATTAATCATATTTCTTGTCATGTTCAGGAAACTGGAAACGTTGTTTCTTGAAGAAAGTTCTGTTGCTGAGAAAGAAAATGATGAATGGCTCCGCGAGCTTGCTACCAGCAATACTGTCCTTGAGACGCTGAATTTCTTTCTGACGGATCTCAGGGCATCCCCAGCATATCTTTTCCTTCTTGTGCGAAATTGCCGAAGGCTGAAAACTCTCAAGATTAGCGACTGTTTCATGTCTGACCTGGTCGACCTGTTCCGTACAGCAGAAACACTACAAGACTTTGCTGGTGGTTCCTTTGATGATCAAGATCAAGGTGGGAATTATGCTAACTACTATTTCCCTCCTTCGGTACAGCGCTTGAGTTTGCTCTACATGGGAACAAATGAGATGCAGATATTATTTCCATATGGTGCCACACTCAAGAAGTTAGACCTTCAGTTTACATTCCTTACCACAGAGGATCACTGTCAATTAGTCCAGCGCTGCCCAAATCTAGAAGTTTTGGAGGTATGTCCTGGCAAATTATTTTAAATGTTTTTGCCATCTTTCCTGTCTGGAAGTCTGATAGTGAATATGCTATCTCAAACGTGTTTTGTTGATAATTTGTCTTCCAACAGGTGAGGGATGTGATAGGAGACCGAGGGTTAGAAGTTGTTGCGCGGACCTGCAAGAAATTACAGCGACTCAGAGTCGAGAGAGGAGACGATGACCAAGGAGGTCTTGAGGACGAACAGGGTAGAGTGACACAAGTAGGATTGATGGCTGTAGCTGAAGGCTGTCCTGATTTGGAGTACTGGGCAGTACATGTGTCTGACATTACAAATGCAGCTCTTGAGGCCATTGGTGCGTTCAGCAAAAACCTGAACGATTTCCGACTTGTCCTGCTTGATAGAGAGGTGCATATAACTGAACTGCCCCTTGACAACGGGGTTCGGGCTTTGCTGAGAGGTTGCACCAAACTCCGGAGGTTTGCATTTTATGTGAGACCTGGAGCTCTATCAGATATTGGCCTTTCTTATGTTGGCGAATTTGGCAAGACTGTCCGCTACATGTTGCTTGGGAATGCTGGGGGATCTGATGATGGACTGCTGGCATTTGCACGAGGATGCCCAAGCTTGCAGAAATTGGAGCTAAGGAGTTGCTGCTTTAGTGAACGTGCATTGGCAGTTGCAGCCTTACAGCTGAAGTCACTCAGATATCTTTGGGTGCAGGGATACAAGGCATCTCCTACCGGCACCGATCTCATGGCAATGGTACGCCCCTTCTGGAACATTGAGTTTATTGCACCAAATCAAGATGAGCCTTGCCCAGAGGGTCAGGCACAGATTCTGGCATACTACTCTCTGGCTGGGGCAAGGACAGATTGTCCTCACTCAGTAATTCCCCTCTATCCGTCAGTCGGAAGCTAA

>AetCOI2 EMT11181

ATGGCGATGACTGCAGTTTCAGGCCGCTGGTCCCTTGCAGTCATCGACAAGGTCAGGACGGCTCTAATAAGAAAGCGACGACGATGGCTAGTTCTTTATTATGTTTGAGGTGCTTTGTATTTCCATTGTACCTTTATAAAGGATCTGGATCTTTTTCGCAAAACTATTATTTTTAGATAAAATCTGTCGTACAGTAAACTCTTCTCGAAAAGGATAGGAAAGCGGGCTCCCAACCCTTCGTAGATCAGCTGTGGAATATATTTCTCAAAGGAAAAAAATGTGGAAGACTGTAACCAATTTTTTTTTGCATGGTAAGACGTGTCTCGTTCATATCATAAAGATCAAAATACAAGTCACGTAAGGACCGACATGACAAAACTGAAAAATAGCAGAACATCTCTGAGTTTGACATCAACGCCCGTCACCCGTCTCCAGCACCACAACAGCAGGCGCCGAAGAAAAGAATGACGGATCACCTCCTCACCCGACCTCGACGCGGCTCCATCGCTGATATGCAGCTTTGCGGACCTCCAAGGCGGCTCACAAAAAGTGAAGCCCTTGCCGTTGAACGAATCAGACCGGGGCAACACCCCGGGCACGTCAGCGAACTCCAGATCTGGCACCCGACCACGACTAAGACGCCGAAGAGGGAAACCATACCTGTCATCCACGCAACACGAACCCAGCGCACGTTCCGTCTTCCAGATATCGTCGATGCAGACTACAATCTGCATCCGCTCCTGGACTGTAACCAATTCTACTACTCTTTATTTCTTTTGCGGGTGAGAATTCTACTTCACTCGCTGAATGTGCTTTGGGATCTTATGTTAGAGTTTTATAGGTTTTTGTTTTAGTGTGAGATTTTGGCAGTGAGAATATATGCCACTCTCCCTGTCCAACGTTAATTTTCGGGATAAACCAGTCCTATAGTAAACTCTTCTGACCCCGCAAAAAAAATACAGTAAACTCTTCTGTACAGTACTGTAAAAAAGCCACAAAGGTACATAGAAGGTACACAAAGGTACGCCTCCACTTGGTAACCGTAGCGTTACCCTCTGCCGAAATACTTTTACTCTGCTGGAAGAGAAAAAAAAAAGAAAAATGGTTCCTCCCTCCGTCCCCCGTCCACCTCCCTTCTCCGATCTAATCATTATTTAATCCTTTTCCAGTCTCACATGAGCGCTTCCCCCCAGCCCAAAGTCTATCTTCTTCCACCTCACTGACACTTCACCCCACCACCACCACCACCACCACCATGCATCAGCATGAAGGGGCAGGTGACACATCCTTTTTGCTCACACAAACGCGTATANNNNNNNNNNNNNNNNNNNNNNNNNNNNNNNNNNNNNNNNNNNNNNNNNNNNNNNNNNNNNNNNNNNNNNNNNNNNNNNNNNNNNNNNNNNNNNNNNNNNNNNNNNNNNNNNNNNNNNNNNNNNNNNNNNNNNNNNNNNNNNNNNNNNNNNNNNNNNNNNNNNNNNNACCGCGGCCCGCGACCGCAACGCCGCCTCGCTCGTCTGCCGCTCCTGGTACCGCGCCGAGGCGCAGACGCGCCGCGAGCTCTTCATCGGCAACTGCTACGCCGTCGACCCGCGCCGCGCCGTCGCCCGCTTCCGCGCCGTCGCCGCCGTCGTGCTCAAGGGCAAGCCGCGCTTCGCCGACTTCAGCCTCCTCCCCGACGGCTGGGGCGCCAACGTCAAGCCCTGGCTGGAGGCGCTCGGCCCCGCCTACCCGTGCCTCGAGCGCATCTGCCTCAAGCGCATGACCGTCACCGACGACGACCTCGGCCTCGTCGCGCGCTCCTTCCCGGGGTTCCAGGAGCTCTCCCTCGTCTGCTGCGACGGCTTCAGCACCCTCGGCCTCGCCGTCATCGCCGAGGGTTGCCGGTACTATGTCCCAGCCAAACTTTCTTTTTCTCTCGTAGTATATAGCTTGCTGTCGACGCATTCCCGTTGACTTCGGGTTGACACTGCGGTTGGAATTGAAAGCTGCCGAAGGTTGAATCTTTGCAGCCTTCTCTCTCTCTCTCTCTCTCTCTCTCTCTCTCTCGCGAGGTCCTCTAGTTCTCAACCTATGCACGTGTGCCATTCTTTAGCTTGTTGTCGCACTTGCTATGTTTTGCTGCTAGATTTACTCGCATATTTCTCCGTTGACGGCCCCATGAATGAACTCAAAATATATCTTGACGTGCGAAGTACTTTGATACGTGTTCGTACTAATGATGCTTACATTTGTGGTTTATTCCACTGACTCAAGGCGTTAAAACATAATCGCTTTATTTCGTTAGTGCCCGTTGACGAATGAAAATTAAATCCTGGGTAGGGAATATTGCAGTTCATATATTTAGTACTAACGAAACTTTTAATGGGTCCGGGTTAACTAACTCCCACTCTCTGTAATAGGCAATTTGGTATTCCGTTGCATTTGTGGCAATCCAATTGTACTCCCTCCTTCCAAATATGTAAAGCCCCTCAAATTTTTAGCCTAACTTTTGTCCACTTATTTGACCAACATAATATGAGTTATATGTTACAGAGAGTATACCATTTGATTCGTATCTGAAAGTTCTTGTGGTATAATATTCGGGGCATACCACTTATACTTTCTTACTCAAATTCATGGTCAAAGTTAGGCTCAAAGTTTGATGGGTGCCTTACATATTTGGAAAGAGGGGGTAGCTTAATGCTTATCTACTTTGGCAACTCCCAGACTTACGTCTCCAGGCTCCAGATGTGCTTGTTCCTCTTGCTTTGCTTCAGTTTATAAAGGAGTGGAGTACGAGTGTGGCATCTCATCGACTAATGTACTTAATACTTGTGTAGGCACCTGAGGGTTCTGGATCTGATCGAGGACTATGTGGATCAGGAGGACGAAGCAGTGGACTGGATCTCCAAGTTCCCAGTGTCAAACACATCCCTAGAGTCCCTCATGTTTGACTGCGTTGGTGCCCCATTCAACTTTGAGGCCCTGGAGGCCCTTGTGGCACGCTCGCCCTCTCTTCGCCGGCTGCGTGTGAACCACCACGTTTCAGTGGAGCAGCTCCGCCGTCTCATGGCGCGGGCTCCGCAGCTCACACACCTTGGCACTGGGGCCTTTCGTCCTGAGGCTCCACAAGGTGAGGGCATGTCGGTGTCTGAACTCGCGCCCTCTTTTGCAGCCTCGACGTCTATAGTCTGCCTCTCTGGATTCCAGGAGGTCAACCCTGAATACCTTCCAGCAATCTACCCAGTGTGTGGTAATCTCACCTCCCTTAACGTTAGCTTTGCAAGCCTGACTGCTGAGGATCTGACACCAGTCATTCGCCAATGCCACAAACTTCAGACATTCTGGGTAGGTCCATACTCTTATTTGTTTGCCATGGTAATCTAATCCATGTTACTTTATTTTCATCTGTACTAAACTGCATCTTGTTTAGGTTCTTGATACTGTGGGTGATGAAGGCCTCAGGGCTGTGGCCGAGACATGCTCTGATCTTCGTGAGCTGCGAGTATTTCCGCTGGATGCCACCGAGGACTCTGATGGGTCTGTGTCAGATGTTGGGCTTCAGGCTATATCCGAAGGTTGCCGAAAGCTTGAATCCATACTTTACTTTTGCCAGCGGATGACAAATGCTGCGGTCGTAGCTATGTCCAATAACTGCCCTGACCTTGTGGTGTTCCGTCTTTGCATCATGGGTCGCCACCGCCCTGATCGCATTACTGGGGAGCCCATGGATGACGGCTTTGGTGCGATAGTGAAGAACTGCAAGAAACTTACTAGGCTCTCTGTCTCTGGCCTGCTCACTGATAAGGCATTTGCATACATTGGGCAATATGGCAAACTAATAAAGACTTTGTCTCTTGCCTTCTCGGGGAACAGTGACCTGTCGCTTCAGTTTTTGTTCGAGGGATGCACTCGGTTACAGAAGCTAGAGGTCAGAGATAGCCCTTTCAGTGACAGGGGATTGCTTTGTGGTCTGGATTATTTCTACAATATGAGGTTCCTGTGGATGAATTCATGCAGGCTAACCATGAGGGGCTGTAGGGAGGTAGCTCAGCGGATGCCTAACTTGGTCGTTGAAGTAATGGAGGAGCAAAATGAGGACAAGGTGGAAACAGAGACCGTTGATAAGTTGTACCTGTACCGATCACTAGCAGGGCCAAGGGGTGATGCGCCGCCATTGGTGAAAATTTTGTAG

>AetCOI3 EMT22616

ATGTGCTACAAAAATTTCGTCAGGTAAGATGAAAATATATGTACTATTGTTACTAACGAGCTACTCGTGAAACTCATTAGCTCGTTCGTTAAGTTCGTTAAGCTTAACAAGCTGAAATCCATGATTGGCTCTGTTCATTAAGAAGCGAACCACAAGCTTAACGGGCTACTCATGAAACTCGTTAGCTCGTTCGTTAAGCTTAACGAGCTGGAATCCATGATTGGCTCTGTTCATTAAGAAGCAAACCACGAGCTTAACGAGCCGAACCATCGAGCGCTCAGTAAGCTCGCGAGCTTCGACCTTTTGATACAGCCCTATCTGCAGCAGGCGATGCTGTCGGAGCGCATGCAACAGCTAAAATGTTGCCGCCTCATGTAGTAGCGGCGTGGCCCACGCCTCGTCGCGCGTGCAGCAGGACCTGTCGGCCTGGGAGCGGCAGGAGCAGTGGTGGCCGCCTCGTACGGTGGCGGCAGGGCCCACCTGCCCCGTTCCGTCCCGGCTCGAGCCGGTCCAAAAACCTGTTTTACCCGGGCGGCCGCCTTCAGGCGTGGCGGCAGGTGCCACGTGTCGCCTGCCGCGCCTGCCGTCACGGTTGAGGGGGTCCTTTTTAACAATTTTATCCACAAGTAGTCCTTTTTGGCAATAGCGTTCGGCATGGGTTCTTTTGGACAAAAAATCGCATGCTTATGCGTATTCTAGAAAACGAAAAAACCGTGTACCCCGGACGGAGCACTTAACCGGATTTAATAACCCTCGCGTCCGTAGCATCTCTTCCTCCATTCCTTCCTNNNNNNNNNNNNNNNNNNNNNNNNNNNNNNNNNNNNNNNNNNNNNNNNNNNNNNNNNNNNNNNNNNNNNNNNNNNNNNNNNNNNNNNNNNNNNNNNNNNNNNNNNNNNNNNNNNNNNNNNNNNNNNNNNNNNNNNNNNNNCCGCTCCCCGCCACCACCACCGCAAGCCCCCTCCTCCGCCGCCGTCCCCCACCGCGCAGGCAGGGTCCCCTGGTTTCTGCAGCGCCGCAGCCGCCGAGGACGCGGCCGCGGAGGCTGACTACACTTCCGACCTCCCCGAGGAGCTCCTCGCCGTCGTATTCGGGCTCCTCGGCTCCGGCGACCGCAAGCGCTGCTCCCTGGTGTGCCGCCGCTGGCTCGCCACCGAGGCCTCCTCGCGCCTGCGCCTCGCCCTCGACGCGCGGGCGCCCCTCCTCGCCGCGGCCCCGGCCATTCTCGCGCGCTTCTCCGCCGTATCCAAGCTCGCGCTCAAGTGCGACCGCCGCGCGGAGAGCGTCGGCGATCCCGCGCTCGCGCTCGTCGCGCATCGCCTCGGCCCCGGCCTTCGCCGCCTCAAGCTCCGCTCGGTCCGTGCTGTCACCGACCACGGCGTCGCCGCCCTCGCAGCCGCGGCTATAAACCTCCGCAAGCTCTCAGTTGGGTCATGTACCTTCGGCGCCAAGGGGATCGAGGCAGTTCTCCGGTCCTGCCCCCAGCTCGAGGAGCTCTCTGTCAAGCGGCTGCGCGGCCTAGCTGACTCAGAGCCCATCACCGTCTCCAGCCCTCGTCTCCAGTCCCTGGCCCTTAAAGAGCTCTACAACGGGCAGTGCTTCTCCTGTTTAATCACGCGCTCCCCCAACCTCAAAACCCTCAAGATCATCCGCTGCTCCGGTGATTGGGACCCGGTTCTCCAGGCGATCCCACAGGGTGCCTTGCTAGCCGAGCTTCATCTCGAGAAACTGCAGGTCAGTGACCTTGGTGTGGCGGCGCTATGTGGGCTAGAGGTCCTGTACCTTGCCAAGGCGCCGGAGGTCACAGATGTTGGGTTGGCAGCACTTGCCACCAAGTCGCCACGTCTACGCAAGCTGCATGTAGATGGATGGAAGGCGAATAGGATTGGCGACCGTGGGCTTGCAACCGTGGCGCAGAAATGTGCTGCTTTGCAGGAATTAGTCCTCATTGGTGTGAATTTGACATCGGCGAGTCTTGAGTTGATTGCTGCCAACTGCCCCACTCTTGAGCGGCTTGCGCTTTGCGGGTCTGACACATTTGGGGATGCAGAGATATCTTGCGTGGCGACTAAGTGTGCTTCTCTGCGGAAGCTGTGCATCAAGGCATGCCCTGTGTCTGATGCCGGAATGGACAAGCTTGCAGCAGGCTGCCCACGCCTTGTCAAGGTGAAGGTGAAAAAGTGCCGCAGGGTGACGTTTGAGTGTGCTGAGCGGCTTCGTGCTAGTCGGCATGGCGCTCTTGCTGTGAATTTTGACACGCCAGGTGGTGCAGGCGAATTGCAAGATGCTAGTGTGGATGAGAGTGGTGTACTGGAGAATGCAGGGAGTGATGTGGTACAAGATGATTTTGATGATCAGATAGGGGTTCCTGACCTTCTGTGTGGCACCAGTGGCAGACCATCAGGGTGGAAAGCATGGATGAGTACTTTGATTCCAAGGAGCTTGTCGGTTTCCATGTTTCGCAGACGTCCGCGTGGGAGCTCCCATAA

>AetCOI4 EMT31633

ATGGGCGGGCGGGCGGCCGTCCCGGGGACATTTTGGTCATCTTCCTTGGTCTCAGGTGCTACCTCCACCTCGGCCGAGGGCAGGCGCTACTCCGACAGGTGCTACCTATCCACATCTCCATCTTTCTCCTCCATCCTCCCTCTCCCCCCACTCCACACCACGGGGTCCTGTTCATCTAGAGGCAATGCCACCGCTCACTGTTCATCCACCCCCCCCCCCCCCCCCACTCTCACNNNNNNNNNNNNNNNNNNNNNNNNNNNNNNNNNNNNNNNNNNNNNNNNNNNNNNNNNNNNNNNNNNNNNNNNNNNNNNNNNNNNNNNNNNNNNNNNNNNNNNNNNNNNNNNNNNNNNNNNNNNNNNNNNNNNNNNNNNNNNNNNNNNNNNNNNNNNNNNNNNNNNNNNNNNNNNNNNNNNNNNNNNNNNNNNNNNNNNNNNNNNNNNNNNNNNNNNNNNNNNNNNNNNNNNNNNNNNNNNNNNNNNNNNNNNNNNNNNNNNNNNNNNNNNNNNNNNNNNNNNNNNNNNNNNNNNNNNNNNNNNNNNNNNNNNNNNNNNNNNNNNNNNNNNNNNNNNNNNNNNNNNNNNNNNNNNNNNNNNNNNNNNNNNNNNNNNNNNNNNNNNNNNNNNNNNNNNNNNNNNNNNNNNNNNNNNNNNNNNNNNNNNNNNNNNNNNNNNNNNNNNNNNNNNNNNNNNNNNNNNNNNNNNNNNNNNNNNNNNNNNNNNNNNNNNNNNNNNNNNNNNNNNNNNNNNNNNNNNNNNNNNNNNNNNNNNNNNNNNNNNNNNNNNNNNNNNNNNNNNNNNNNNNNNNNNNNNNNNNNNNNNNNNNNNNNNNNNNNNNNNNNNNNNNNNNNNNNNNNNNNNNNNNNNNNNNNNNNNNNNNNNNNNNNNNNNNNNNNNNNNNNNNNNNNNNNNNNNNNNNNNNNNNNNNNNNNNNNNNNNNNNNNNNNNNNNNNNNNNNNNNNNNNNNNNNNNNNNNNNNNNNNNNNNNNNNNNNNNNNNNNNNNNNNNNNNNNNNNNNNNNNNNNNNNNNNNNNNNNNNNNNNNNNNNNNNNNNNNNNNNNNNNNNNNNNNNNNNNNNNNNNNNNNNNNNNNNNNNNNNNNNNNNNNNNNNNNNNNNNNNNNNNNNNNNNNNNNNNNNNNNNNNNNNNNNNNNNNNNNNNNNNNNNNNNNNNNNNNNNNNNNNNNNNNNNNNNNNNNNNNNNNNNNNNNNNNNNNNNNNNNNNNNNNNNNNNNNNNNNNNNNNNNNNNNNNNNNNNNNNNNNNNNNNNNNNNNNNNNNNNNNNNNNNNNNNNNNNNNNNNNNNNNNNNNNNNNNNNNNNNNNNNNNNNNNNNNNNNNNNNNNNNNNNNNNNNNNNNNNNNNNNNNNNNNNGCGGGGCGGGAGCCCCTGGTGCCCGCCTGCCCCGGGCTCGAGGCAGTCGATCTGTCGCACTGCGTCGGTGCTGGGGATAGGGAGGCTGCCGCGCTGGCCGCGGCCTCCGGGCTGAGGGAGCTGAATCTGGAAAAGTGCCTGGGCGTCACTGACATGGGACTCGCCAAGGTAGCCGTGGGGTGCCCCAGACTGGAGAATCTGAGCTTCAAGTGGTGCCGTGAAATCTCTGACATCGGCGTCGATCTGCTTGTGAAGAAGTGCCGCGAGCTCCGCAGCCTTGACATCTCCTACCTAAAGGTTATACTCCCATATGAGCTGTTGAACTCTTCACTTTATGCAATGGAAAGGTTTAGGTTCAGCCTTTCGGGTTAGGACTAGAACCCTTGATAAACAGGAGAAACGATGCCGCGACGAATACAGAGTTAGAGTGGACTTTGATGTTTATTGATTGCTTTTAGACCCAGAAAATGTCAAAGAGATTTGAGGTATTGGACCATTGGACTTATTCGGTGGTTCATCCAGTTTAGAGATTGATGCATTTGTTAGGGTACTATGGGTCAACTTATGGAGACATCTTTGAAAAAATGCAGCTGGAAGAGGAAACATTCTTACATATCTGTTCTGTTGGCTAGTATATAGGCTTTTAGAAAAAACTCGTGACCTCTTGGATCACACTTTAGTTTCATCTTCCTTCCAAGAAAAGTTCAAATTTGGTGCCAGATGGCAAAAGTACATTTGAAAAAATTGTACCGTGTGATAGGTACTGGTATGTATTGTAGGAGTTAGAGGTGGAAAAGATGGATTTTGACCTGGGCTATTTCTTGGTCCTTTTGACTAGGAAATTATGATGCGTTGGTTGAAGTTTACCTCTGTATATGTTGAATCTGAATCTGTTGCTGTGTAGATTAGACTTGACTCATTTTATGGAGTGCACTCATTATATCTTGGTGCAATTTATAATTGTATTTTGGTCAACAACAACAAAGCCTTTTGTCCCAAGCAATTTGGGGTAGGCTAGAGATGAAACTCAACAGAAAACCGTGAGAACCCAAAAGAGAAGAGAGAAAGTAAAAGTAAACAAAATAAATCAAGGTTCTGGTACATGGATTACTGATTTCCATCATTCCATGCAGTCCTGTCAAGAACCAAATCCCTGGGTACATCCCAATCCTTCAAGCCTCTTTTTACCGCCTCCTCCCGTATCAACTTTGGTCGCTCCTACCCCTCCTAGTATTTTCAATACTCTTAAGAATAGTATTTTGGTCACTCAAGAGAAATTGTATTTTGGTACTTCTTCTGTCATGGAACTGAAAGGAACTGCATGTGCCGTGTCATTGTGCCATGTTGTTCAGTATTACTCTCTCCTTATCAAAATATAAGACTTTTTTGACACTATCAAAAAACTTTAGTATACTACCCTTTCCTGCCGACCCTTTTGGCATGATGCTGCTGGAAAGGGGTGAGTTTGGGTAAAAAAAAATCTAGGTAATTGAGTGGGAGAGGCTAGGCAGAACCACAGGATAGACTAATCCAAATATACCTTTGGTCTTCATCTCTTGCAGTTTGTAAGAAATTGCACCATTAAATGGTTTGTCATATTATTGGTGACTTTGATGCATCTTAGGGAATGCTATTGTTATGAGTAGACATGGCTGCATCCATTTGGCATGTGGTGTATGTTTTGGAATCCATGTTAACAAGTTTAATGCACGTCCAACGAAAATCTTAAATCGGCGAGGAAAGTTTTTGCCAACATAATTTTACTTCGACACATGGCACCATTGTAACGTGAGGAGGTCTCCCTTTTAGGGGAAATTGAGAAGTCCTCATGCTTACAGAAAATATACTAATTTCTTGCGCTGTGATAAACTATATAATAATAATATACTACTGAAAGGAAGTAGCATTTGTTAATCAATTGATGGTGCAATTTCTTACAAACTATTTAATAATGTGCATCAAAGTATTAATGTGAGGAGTATAATCTAGTATTTTGTCAGCATTTGGTTAGTTCTGAATTTCATCAACTACTGAAAGGAAGTAGCAGCAGTTTAAACCTGAAAGTGTATATTTGTTTACTGCTGTTGGAAATTATCTCGAGAATGGATTGAACATACTCATGAGCAAATTGCTTTCTACTCAAGTTATTAGTTTGGTTCCATCTAAAATTTTGGAAATCACTTCTTTTTGTGAACTGTACATATGATTTACTTAATTTTTCTTTTCTGTTGCAGGTGAGCAATGAGTCCCTTAGATCAATATCAACACTTGAGAAGCTTGAGGAGTTGGCCATGGTTGCTTGCTCATGTATAGACGATGAAGGCCTGGAATTGCTTAGCAGAGGAAGTAATTCATTGCAGGTATATGGATAATTATTGTAGTCTCATGCAGGGATACTCTTGCATATTATCATGTCTTCCGTCACCTGAAATCATATGGCTTACTCTCTCGTCAAACATGGTATGTAGATAATGATTTTGTCTCATGCGCATTGTCATGTCTTTGATCATCCGAAATTATTGCTTAGTCTCTCGTCACACTTTAAACTGGATAGGTCTAGTGTGCAAGTTTACCTGCAATTTTTACTGTTTGATTATTCTTTTACATCTTACCAAGCATCGATCCATTCTAAATATCGTCTTCCATTGCAGAGTGTTGATGTGTCAAGATGTGATCATGTGACTTCCCAGGGGTTAGCTTCACTGATAGATGGTCACAGTTTTCTCCAGAAGCTATATGCCGCAGATAGTTTGCATGTGAGTTGGTATTTCTAATTTTACTTGCTTTGCTGCACACATTGGCTGTACTTTTCGATAAAATGATGCCATGTTTTTGGGGGAGGCTTGGCCCCCATGATGAATGAAGGAATACATAATTGCTGATGATTATTGACCTTCTACTTGAATTTTGTAGGAGATTGGACAGGATTTTCTATCCAAGTTAGTAACACTGAAGGCAACCTTGGCCGTGTTGAGACTTGACGGCTTTGAAGTGTCATCCTCTCTTCTTTCAGCGATTGGTGAAGGTTGTACCAACTTGGTTGAGATTGGACTAAGCAAATGCAACGGTGTTACAGATGAAGGCATCTCTTCGCTTGTAGCTTGCTGTAGCTACCTAAGGACAATTGATCTCACATGCTGCAATCTAGTCACAAACAATTCCCTTGATTCAATAGCTGACAACTGTAAGCTGCTTGAATGCCTCCGGTTGGAGTCCTGCTCTTCAATAAACGAGAAAGGACTAGAGAGAATTGCGAGCTGTTGCCCCAATCTAAAGGAGATAGATCTCACTGATTGTGGAGTGAATGACGAAGGTTTGTGATACTGCATACTGCATACTGCATCTTTTTTCATCCAAGCATGTCCATGATTTGAGCATTGTCATATGAAGTTAACTCTCTTGCAGCGTTGCATCATTTGGCGAAGTGCTCTGAACTGCTGATATTGAAATTAGGCCTGAGCTCAAGTATTTCGGACAAAGGCCTTGGTTTTATTAGTTCAAAGTGTGGAAAGCTCATTGAACTTGACCTCTATCGGTAATTTGATCTCCTTGTTTGTTCTTTATAGGCTTCCAGCCATCTTCCATGCACAATAATAACTTGCATATTATCTTTCTATCCTAAAGCTGCAGTTCTATCACCGATGATGGGCTGGCAGCCTTAGCCAACGGCTGCAAGAAAATTAAGCTGCCGAACCCTTGTTACTGCAACAAGATAACTGATTGATACTGAACCTTTGTTACTGCAACAAGATAACTGATAGTGGTTTGAGCCACCTGGGCGCTCTGGAGGAGCTCACAAACCTTGAACTGAGATGCCTGGTCCGCATTACAGGCATCGGAATTTCTTCGGTTGTCACTGGCTGTAAGAGCCTGGTAGAACTTGACTTGAAGCGCTGCTATTCTGTCGATGATTCTGGCCTATGGGCTCTTGCTCGATATGCTCTAAACCTTAGACAGGTAAATGGTATGCAGAATTTAGCTTCTTCTTGTACAAGTTCTGACGAGGCAGGAACCTTATTAGATTGTCCATATTCTGCAGCTTACCATATCATACTGCCAAGTTACTGGCCTAGGCTTGTGCCACCTGCTTAGCTCCCTGAGGTGCCTCCAGGACGTGAAGATGGTGCACCTCTCGTGGGTTTCCATAGAAGGGTTCGAGATGGCTCTGAGAGCCGCTTGTGGGAGGCTGAAGAAGCTGAAGATACTCAGCGGTTTGAAGACCGTGCTATCCCCTGATCTGCTCCAGCTGCTGCAAGCCTGCGGCTGCCGCATCAGATGGGTCAACAAGCCTCTTGTCTACAAGGATGCCATCTGA

>AetCOI5 EMT03750

ATGGAGGATCTCCCAGAGCCACTGCTTGCAGAGATCGTCAAGAGGATTACCAAGACAAGGGATCTTAATTCTCTTTCCCTCGTGTCGAAGCAGCTCTGCACCGTTGAGGCGGAGCACAGGGATGCTATCCGTGTTGGCTGTGGACTCAACCCTTCTACAGAAGCCTTGGTATCGTTGTTCTCCCGGTTCCCCAATTTGGCGAAAGTAGAGATAAACTATTCCGGATGGTGGCCCAGTAACGGGAACCAGTTGAACAACCAAGGCCTCTGTGTTCTATCATCTCACTGCCCCTCACTGTCCGATCTTTCTTTAAGCTTCTGCTCTTACATTGGTGACTCTGGTCTTGGTTATCTAGCTGATTGCAAAAAATTGAGGTCGCTCAGGCTGAACTGTGCACCAGCAGTAACTTCTACTGGGATTTTTCGTGTGGCGGTTGGTTGCAGGCATCTTTCGGTTCTTCACCTTGTTGACTGTATGGCAGTAGACAGCAGGGAGTGGCTGGAGTACCTTGGGAGATATGGATCATTGGTAGAACTTGTTGTAAAGGATTGCGATGGAATCAGCCAGTATGACCTCTTAAAGTTTGGCCCAGGCTGGGAGAATCTCCAAAAATTTGTGTTTGAGATTAATGGAAACTACTGGATGTCAGGGGCGTCTGACCCCTCCTACGTGTCTGGCTACCCATATAGGTATGACATCTGCTGTGATAGCTTGAAGGATCTTAGGTTAGCTCATATTATAACCGAGCCAGAAACTGGACTTCGTTTTCTCCTGGGGAAGTGCAAAGCATTGGAGACGCTTTACCTAGAGTATGTGATTGGTCTAAAAGAGAATGAGACGATTGCGCTATTCCAGAGGTGCAGCAACCTTAAAACCATCTCACTTCGGTTCATGCCTCTGCGGTGTGAAGATTATGAGTTTAGGACGCCATTGACTGATGTTAGCCTTAAGGCTCTAGCTCTCAGCTGCCCTATGCTTCAGGTTGTAGAGCTCACATTCACATTTTGCGAACCCATGTATCCAACAGAAATAGGTTTCACACAAGAGGGTATTGTGTCGCTCGTCCAATCTTGTCCAGTCCGCACTCTTTTGCTAAATGGTGCCAGCATTTTGTATGACGAGGGGTTGAAGTGCCTCTCATCCTCACAGTTCCTGGAGAAGCTTGAGCTCGTGGATTGCAGGTCTATAACCGATGCTGGTATGAGTTTCATTATACTGGCTCCTTGCTTCAGCAATCTCACCCTCCGCAAATGCAAGAAAGTGACAGACAATGGGATGGCTGAGCTGGCACGTTCACAGAAGTTGGAGTCACTGACCGTTATAGGCTGCTGCCAGATCTCTCAGGAGGGTGTGCAAGGGGCTGCCAAATCAGTTCACTACTCTGCAGAGACTGAAAGCCACGGCAGTCTAAAAGGAATGAACACGGACAGGGACATGAACAGGAAAAGACGCCGGTCGCCCTGA

>AtCOI1 AT3G62980.1

CACTCTCTCTCTCTCTCTATCTATCTTCTTCATCTTTGTTTCAGCTTCTCCTTTGAAACTAGATTATCTCCGTTAATGGCGCCTCAGATCTAACCTTGGGAGTTATATATATATACTTGTTCAACTCTTCTTCGAACGCTGTCCAACTTCTTCCTCTGGTTCGATCATCGAGATCTCGAGGTTATTTGGCCGCAATGCAGAAGCGAATAGCCTTGTCGTTTCCAGAAGAGGTACTAGAGCATGTGTTCTCGTTTATTCAGCTGGATAAGGATAGGAACTCAGTCTCTCTGGTGTGCAAGTCATGGTACGAGATCGAGCGGTGGTGCAGGAGGAAAGTCTTCATCGGGAACTGCTACGCCGTGAGTCCAGCGACGGTGATTAGGAGGTTCCCGAAAGTGAGATCCGTGGAGCTTAAAGGAAAACCTCACTTTGCTGACTTTAATTTGGTACCTGACGGATGGGGAGGTTACGTGTATCCATGGATTGAGGCCATGTCTTCGTCTTACACGTGGCTTGAAGAGATAAGGCTGAAGAGGATGGTGGTCACCGACGATTGCTTGGAGCTCATAGCCAAGTCTTTTAAGAATTTTAAGGTTCTTGTGCTTTCTTCCTGCGAAGGCTTCTCCACCGATGGTCTCGCTGCTATCGCTGCCACTTGCAGGTATTCTTTTTCTGAATGTTTTTTGTTTCATCAAGAAGACTCCCTTGTGTCTCTAGGAAGAAGCTTTTGTTTGTATTAAACTTATTGGTGTGTGATTTGTATATTCATTATACTACATAAAGTCTCAATTATAGCAAATTCATGTACTTGTTATTTGATCTGGGCATATAGGTTTTGCTGCGTTGTTCCTGCTGATGTTACTCTGGTCTTTGTGAGCTTTTTTTTGGTTTGGTCAAGATTTTGCGTATCTAAAAGATATATGTTTTAATCACGTTTCTGAAGATTCTTATTATAGCCACTCTCTTTTTCTTTCTGGTGGGTCTTATTTAACGGTGGTGTATCTATTTATTCCTGGATTATTCGCTCTCAGTGAATCTCCTGAGGTGGGAAATCTAATCAATTTGGCTTTGTGTGTGTTACTTTTCTTTTTGTAGGGGACTTTCAATTTTCTTTTTTTACCTTGTTGGGAAACTTAGATCTGTTTTCGGATTCCCCTTCTTTACTCGCTTTAGCTCTGTTTTAGCATCTCTCACATTGCTTCCACGTGATAAATTTAGTAATTTGTTATTGTTCTATGCTTAGATAGTTTCCCAAAGTGATCTTTCTACATTGTATCAAATACAACTTTGGACAAATTAATGACAATTATGCTCATGTATTGTAGGCAATGTTTATGTTCTCGAAGTTGTATAATGTGGGTCACTCGATGCTAATTATGTGATTTCTTATGCAGGAATCTGAAAGAGCTTGACTTACGAGAGAGTGATGTTGACGACGTTAGTGGCCACTGGCTTAGCCATTTCCCAGATACATACACTTCTTTGGTATCACTCAATATATCTTGCTTAGCATCTGAGGTCAGTTTCTCTGCTCTGGAAAGGCTGGTGACTAGGTGTCCCAATCTCAAGTCTCTCAAGCTTAACCGAGCTGTTCCACTTGAAAAATTGGCTACTTTACTTCAAAGAGCACCTCAATTGGAGGAATTGGGCACTGGTGGGTACACTGCAGAAGTGCGACCAGATGTTTACTCTGGTTTATCTGTAGCGCTCTCTGGGTGCAAGGAATTGAGGTGCTTATCTGGATTTTGGGATGCTGTTCCTGCCTATCTTCCAGCAGTTTATTCGGTTTGCAGTCGGCTTACAACTTTGAATCTGAGTTATGCAACAGTCCAGAGCTATGATCTTGTCAAGCTTCTTTGTCAATGCCCTAAACTGCAGCGCCTCTGGGTGAGTCCATTTTAACATACATTAACACATGAATTTGGAATAGGTCATTTTACTGTATGGTATGAGAAAGCTTATGTTCATAGTTTTCTTAAAAAATTTGAATGATATAGAGCAATATATCTGAATTATAAGCATTAAAGCCTTTTTCTTTTTGTAACTGTGCTCTAACTTATTATTGACTACAGGTGCTTGACTACATCGAGGATGCTGGTCTTGAGGTGCTTGCTTCAACCTGCAAGGACCTACGCGAGCTGAGAGTGTTTCCGTCCGAGCCTTTTGTCATGGAACCAAATGTGGCATTGACGGAACAGGGGCTTGTCTCCGTCTCCATGGGCTGTCCAAAACTCGAGTCGGTTCTCTACTTCTGCCGTCAAATGACCAATGCTGCATTGATAACCATTGCTAGGAACCGTCCCAACATGACTCGCTTCCGTTTGTGCATCATTGAGCCAAAAGCCCCAGACTATCTGACTCTAGAGCCACTGGATATTGGATTTGGAGCCATAGTAGAGCACTGCAAGGATCTCCGTCGCCTCTCTCTATCTGGCCTCTTGACCGACAAGGTTTTTGAATACATTGGGACATATGCCAAGAAGATGGAAATGCTCTCAGTGGCATTTGCAGGAGACAGTGACTTAGGCATGCATCATGTTTTGTCCGGGTGCGATAGCTTGAGGAAACTAGAGATAAGGGACTGCCCGTTTGGAGACAAGGCGCTTTTGGCCAATGCTTCAAAGCTGGAGACAATGCGATCCCTTTGGATGTCTTCTTGTTCCGTGAGTTTTGGAGCCTGCAAGTTACTAGGACAGAAGATGCCAAAGCTGAATGTGGAAGTCATCGATGAACGGGGTGCACCGGACTCGAGACCAGAGAGCTGCCCTGTTGAGAGAGTCTTCATATACCGAACAGTGGCTGGTCCTCGATTTGACATGCCTGGCTTCGTCTGGAACATGGACCAAGACTCAACAATGAGGTTTTCCAGGCAAATCATTACTACTAACGGATTATAAGACAAAGAAACGAGGTACATTTTTCATTAATCCTTGTTCATCATCCCATATAATCGTGTTCCGGTTTAGGAACATGAGAGAGCTCGGTCGCCAATAATTTGAATCTCGGCTGCTTCATATAACCATCAAGCTCATGAGCAATGAATAAGTGGAGAGAGAGAAAGAGAGATCTAAGAGAGCTTTGTTTTATTTATGTAAGAATTATTGATTGTTGTATGTTTATTTGGCTACTTACACTGAGAGATCCTGGTGGGGTATTTTTCTTAAGAGGATAAGAGATTTTTTTAAAAGACAATGTCAAAAATGTAACTGTTCTACTTGTTTTGTTTGGACTCTTTTATCCTTTTAAATGTTTTCTATCTC

>AtCOI2 AT1G12820.1

ATTACAGAGTTTCTGGTTTTTTTTGGCTTGCGTGATCTCTCTCTTCTCTTCTCTCTCTCACTCATCCAAATCTTTGATCATCTCATCATCATTGGACTTGAAGCTAACTCCAGAGTTTTATCCAGATTCATCATTTTCCAACGTTTTTCTTCAATCGAATCATCAGCGAGTTTGGTTCCGACGACGATCTTAGTAGCTTCATAAGCTCAAGGTCTCGAAACTCTTTCTAGATCTAGCTCATTTACTTTCTTGTAGATCTGAATCTTAATCTCTGTGTTTAGTTTATCGAGTTTCAGGATCTGTTGTTTCTAAATTAGTTTGTATAATTTGAATCTCGCTTTGTTGCAGATTCTTCAGGTTTCGGTGGATTTTCTTGAGCTCTTCTAAGCTTCTCCATTTTGTGAGCCTCCGATTTCGAACCTGGTAGTGTTGTGGATCTCACTGGACCACGAAGCTTTCTTCACTGAGCTAAGCTTGAAGTCAATTCCTCTGTTCGACAATCAATAGAGGAAGATGAATTATTTCCCAGACGAGGTTATAGAGCACGTGTTTGACTTCGTAGCTTCTCACAAAGACAGGAACTCGATATCTCTGGTCTGCAAATCATGGCACAAGATCGAGAGGTTTAGTAGGAAGGAAGTGTTCATCGGAAACTGCTACGCGATTAACCCGGAGAGGTTGATCAGGAGGTTTCCATGTCTCAAATCCTTAACTTTAAAAGGGAAGCCTCATTTTGCAGACTTCAACTTGGTTCCTCATGAATGGGGAGGTTTCGTGCATCCTTGGATTGAAGCTTTGGCTAGAAGCCGTGTGGGACTTGAGGAGCTGAGGTTGAAGCGGATGGTTGTAACAGATGAAAGCTTGGACCTTCTTTCACGTTCTTTTGCAAATTTCAAGTCTTTGGTTCTTGTTAGCTGTGAAGGGTTTACCACTGATGGCTTAGCTTCCATTGCCGCTAATTGCAGGTAAAGGCCTTTCTTTTACTGGCGTTTCTCTAGGTTTTATGTTGTTTTTAAAATGTGTTGTTGTCTTTCTTATTAGGCATCTTCGTGAGCTGGACTTGCAAGAGAATGAGATTGATGATCATAGAGGTCAATGGCTGAACTGTTTTCCAGATAGCTGCACTACTCTTATGTCGTTGAATTTCGCTTGCCTTAAAGGAGAGACCAATGTTGCTGCTTTAGAAAGGCTTGTTGCTAGGTCACCAAACCTGAAGAGCTTGAAGTTAAACCGTGCAGTACCGCTTGACGCACTCGCAAGGTTAATGAGTTGTGCGCCGCAGCTAGTGGACTTAGGAGTAGGGTCTTATGAGAATGAGCCAGATCCTGAATCTTTTGCAAAACTCATGACTGCCATTAAGAAATACACATCGTTAAGGAGCTTGTCTGGCTTTTTAGAGGTTGCTCCACTCTGCCTCCCAGCGTTCTACCCAATTTGCCAAAACCTTATCTCTTTGAACCTCAGCTATGCAGCTGAAATCCAAGGCAACCACCTCATTAAGCTTATTCAGCTTTGCAAGAGACTTCAACGATTATGGGTAAGTTTGTTCTCACTTTCTCTTTTCACTGTCCTGAAGAAAATAAACTAACTTGCTAACCTGACAACAAATGGTTTTTATGGTTCCTACAGATATTGGATAGTATTGGTGACAAAGGACTTGCGGTTGTCGCTGCCACATGTAAAGAGTTACAAGAGCTTAGAGTTTTTCCCTCTGATGTACATGGTGAAGAAGATAACAACGCATCTGTGACTGAGGTTGGACTAGTCGCCATTTCCGCAGGTTGCCCTAAACTTCATTCGATTCTGTACTTCTGCAAACAGATGACAAACGCAGCGCTCATAGCCGTGGCCAAAAACTGTCCAAACTTCATCCGGTTCAGGCTATGCATTCTCGAGCCACACAAACCTGACCACATTACATTTCAATCACTGGACGAGGGCTTTGGTGCAATCGTACAAGCTTGCAAGGGTCTAAGACGGCTCTCTGTCTCCGGTCTCTTAACCGATCAAGTCTTTCTCTACATCGGTATGTACGCGGAACAGCTCGAGATGCTTTCGATAGCTTTTGCGGGGGACACTGACAAAGGAATGCTCTATGTGTTGAATGGATGCAAAAAAATGAGGAAGCTGGAGATAAGGGACAGTCCTTTTGGGAACGCTGCGCTTCTTGCTGACGTGGGTAGGTACGAAACAATGCGATCCCTTTGGATGTCGTCTTGTGAAGTAACACTCGGTGGCTGCAAGAGGCTCGCGCAGAATTCGCCACGGCTTAACGTAGAGATCATCAACGAGAATGAGAATAATGGGATGGAACAGAATGAAGAAGATGAAAGAGAGAAGGTTGATAAACTTTACCTCTACCGAACAGTGGTTGGGACTAGAAAAGATGCACCACCATATGTTAGGATTCTTTAGTCTCTTTGCACCTTCATGTGTTTTCAAACTTTTCTTTGTACTAGTTTTTGCCTTTTATCAAACTTGGAACAAATACAAATGTCGCATTTTATTATCACCGTAATTCAGCCATTGAAAAC

>AtCOI3 AT5G49980.1

AAAAAATAATCCCCAAATAATGGAGACGAAGTGGAGAGAGAAAGCTCCCACTCTCTCACACCCCAAAGCTTCTTCTTCTTCTTCCTCTTCTTCCTCTTCCTCTTCTCTAATCTGAATCCAAAGCCTCTCTCTTTATGACACAAGATCGCTCAGAAATGTCTGAAGATGACGATGACCAACAATCTCCACCGTTGGATCTACCCTCTACCGCCATAGCTGATCCTTGCTCATCTTCCTCTTCACCAAACAAATCTCGTAACTGTATCTCAAATTCTCAAACTTTCCCTGACCATGTTCTCGAAAACGTACTTGAGAACGTTCTTCAGTTCCTAGATTCAAGATGTGACCGTAACGCTGCTTCTCTAGTTTGCAAATCTTGGTGGCGTGTTGAAGCTTTGACTCGATCTGAGGTTTTTATTGGTAACTGTTACGCTCTTTCTCCGGCGAGGTTGACTCAGAGATTCAAGCGTGTTAGGTCTCTTGTGCTGAAAGGGAAACCTAGGTTTGCTGATTTCAATCTCATGCCTCCTGATTGGGGTGCTAATTTTGCTCCTTGGGTTTCTACTATGGCTCAAGCTTATCCTTGTCTTGAGAAAGTTGATTTGAAGAGGATGTTTGTTACTGATGATGATTTAGCTCTTCTTGCTGACTCTTTTCCTGGGTTTAAAGAGCTTATCTTGGTTTGTTGTGAAGGTTTTGGTACTAGTGGTATCTCTATTGTTGCCAACAAGTGCAGGTAAATGTTACAACTTATTGGATATGAAAATGCTATCTTTGAGTAATGTTTTGATAGTTATGAACTTATTGGATATTAATTTGTTTTCAGAAAGCTGAAAGTGCTTGATTTGATTGAGTCTGAGGTCACGGATGATGAAGTGGATTGGATCTCTTGTTTCCCTGAGGATGTAACTTGTTTGGAGTCTTTAGCTTTTGACTGTGTGGAAGCTCCTATCAATTTTAAGGCGCTTGAGGGTCTTGTTGCTAGGTCACCGTTCTTGAAGAAACTTAGGCTAAACAGGTTTGTGTCTCTTGTGGAGCTACATCGTCTGCTACTTGGAGCTCCACAGCTTACTAGTCTTGGGACTGGTTCATTTAGCCATGATGAGGAACCTCAGAGTGAGCAAGAACCAGATTATGCTGCTGCATTTCGTGCTTGTAAATCTGTAGTTTGCTTGTCAGGGTTTAGAGAGTTGATGCCGGAGTATCTTCCAGCTATCTTTCCGGTGTGCGCTAATCTCACCTCCCTGAACTTCAGTTATGCTAACATTTCTCCTGACATGTTCAAGCCCATCATACTCAATTGCCACAAACTCCAGGTGTTCTGGGTATGTATCCTTCTTGATCTATGTCTTCATGGCTTTATTAAGTTATATGTGACTTAACTCTAGTGACCGTATTGTTTGTGTTAGATGGATAGAATGAATAAACTTGATATTAGTTGTGTAAGGAACTTGAGTAAAATGCATGACTTGAACTTTACAATGCAGGCCCTTGATTCAATATGTGATGAAGGACTACAGGCAGTTGCAGCCACTTGCAAGGAACTCCGTGAACTCAGGATCTTCCCTTTTGATCCTCGGGAAGACAGTGAAGGTCCTGTCTCTGAATTAGGCCTCCAAGCAATCTCCGAGGGTTGTAGGAAACTAGAATCTATTCTCTACTTTTGCCAGCGCATGACTAATGCCGCTGTGATAGCCATGTCAGAGAACTGTCCAGAGCTTACTGTGTTTAGGCTGTGCATAATGGGTCGACATAGGCCTGACCATGTAACAGGAAAGCCTATGGACGAGGGATTTGGTGCCATTGTTAAAAACTGCAAGAAGCTAACTCGCCTTGCAGTGTCGGGATTGCTGACAGATCAAGCTTTTAGGTATATGGGTGAGTATGGGAAATTGGTCCGTACGCTTTCAGTAGCTTTTGCAGGGGACAGTGACATGGCTCTGAGACATGTCCTAGAAGGTTGCCCTAGACTGCAGAAACTTGAGATAAGGGACAGTCCCTTTGGAGATGTTGCATTACGGTCTGGTATGCATCGCTATTACAACATGAGGTTTGTTTGGATGTCAGCATGTAGCTTGTCTAAGGGATGCTGCAAGGATATTGCACGAGCAATGCCGAATCTAGTTGTGGAAGTAATTGGATCGGATGATGATGATGACAATAGGGATTATGTCGAGACTTTATACATGTATCGGTCTCTTGATGGTCCAAGGAATGATGCACCAAAGTTCGTCACGATTTTATAGAACGAGGAGCATTTCCGTGTTTCCGTTTTTTAAGACGGGAATGAAATGGGTAAGTGTAGGAAGCTCAGATGATGATGGTGGTGGCATCAGAGGCACACTACATGATTGTTGATGATGAATGATGGTGGTTGGGACTTGGGAGGAATGCAAGATTGTTGTATCATTATTTTCTTTTTTTATTTTTGGGTTCAAAAGATGTTATTTGGGTGGTTAGGTGGAACAGTGGATGTATGTTTTGGGAGCATCGGTGTTAGTTAGACAGCTGAGTTGGTTCTAATAAGCTGCACTTTTGGCTCTCTCTAGTTGTTGTTGTTGAGAAACTAATGAGACATCTCTCTCTTTTGTCTTTCCCATTTTTAATTAATTTTGAGATATTTCATCTTAATCTGTTTAAATTATGTCATTTGTCAGCAAACTATAATGTAACTTCAAGAAAAGATCATTTTGATTCGATTC

>AtCOI4 AT4G24390.1

GAGAGAGTCCAAACACCAAGACCAGCTCCTTTTTCACCTATCTCTCTTCTTCATCTGAATCAGATTTTGTATCAGAAAGAGAGCACCTGAGCTCATAAATTCTGGATAAGATCAAAGTCAGAGTCTTTCTTGTTTTTCATCACTTACTTGTATGACAGAAGAAGATAGCTCAGCTAAAATGTCAGAGGATGTTGAGAAATATCTCAACTTAAATCCACCTTGCTCCTCCTCCTCCTCTTCTTCCTCCGCCGCTACATTCACGAACAAGTCTCGAAATTTCAAATCTTCTCCCCCGCCGTGTCCAGATCATGTCCTTGAGAACGTTTTAGAGAACGTGCTTCAGTTCCTCACTTCCAGATGCGATCGCAACGCAGTCTCATTGGTCTGCAGATCGTGGTATCGCGTCGAGGCTCAGACTCGATTAGAGGTTTTTATTGGAAACTGTTACTCGCTCTCTCCTGCTCGGCTTATTCACCGGTTCAAGCGTGTTAGGTCTCTTGTGCTTAAAGGGAAACCTAGGTTTGCTGATTTTAATCTCATGCCTCCTAATTGGGGAGCTCAATTCTCTCCTTGGGTTGCTGCTACAGCTAAGGCTTATCCTTGGCTCGAGAAGGTTCATTTGAAGCGTATGTTTGTTACGGATGATGATTTGGCTCTTCTTGCTGAGTCGTTTCCTGGGTTCAAAGAGCTTACTTTGGTCTGCTGTGAAGGTTTTGGGACTAGTGGTATTGCTATTGTTGCTAACAAATGCAGGTACTTTGTAGATCTCTTCTCTTTGTTTGTTTGTTTGTTTGTTCTCAGAGTCTTCTAGAAATGCTTATCAAGTGGGACTTTGTTTTCAGGCAGCTAAAGGTCCTTGATTTGATGGAGTCAGAAGTCACAGATGATGAGTTGGATTGGATTTCTTGTTTTCCTGAGGGTGAAACTCATCTGGAGTCTTTGTCTTTTGACTGTGTTGAATCCCCTATCAATTTCAAGGCATTGGAGGAGCTCGTGGTTAGGTCACCATTCTTGAAGAAACTTAGAACGAACAGGTTTGTTTCCCTTGAAGAGCTGCATCGACTAATGGTTCGAGCGCCGCAGTTAACGAGTCTTGGGACGGGGTCATTTAGTCCAGACAATGTGCCTCAGGGAGAACAACAACCGGATTATGCAGCTGCTTTTCGTGCTTGTAAATCCATAGTTTGTCTCTCAGGATTCAGGGAATTTAGACCGGAATACCTCCTAGCCATCTCTTCAGTTTGTGCTAATCTCACCTCTCTTAACTTCAGTTATGCTAACATTTCTCCTCACATGCTCAAGCCCATCATAAGCAACTGTCACAATATCCGAGTCTTCTGGGTATGTATCTTTATTGATCTCATTGTATAGTATAGTGTAATATCCTTTAACCGAGGACTTTGAGTTACATGCGTATCTTGAAACTTTTATAATGCAGGCTCTTGACTCGATACGTGATGAAGGACTACAGGCAGTGGCTGCCACATGCAAGGAGCTCCGTGAGCTTCGGATTTTCCCTTTTGATCCTCGTGAAGACAGTGAAGGTCCTGTCTCGGGAGTAGGCCTCCAAGCAATTTCAGAGGGCTGTAGGAAACTGGAATCTATCCTGTACTTTTGCCAGAATATGACCAATGGAGCTGTGACAGCCATGTCGGAGAACTGCCCGCAGCTTACTGTGTTTAGACTTTGCATAATGGGTCGCCATAGGCCTGACCACGTGACAGGAAAGCCAATGGACGATGGATTTGGTGCCATTGTTAAAAACTGCAAGAAGCTAACCCGACTTGCAGTATCAGGGTTACTAACAGATGAAGCTTTTAGCTATATAGGAGAATATGGGAAATTGATCCGTACGCTATCTGTAGCGTTTGCTGGGAACAGTGACAAGGCTCTGAGATACGTTCTTGAGGGTTGTCCTAAACTACAAAAGCTTGAGATCAGGGACAGTCCCTTTGGAGATGTTGGATTGCGCTCTGGTATGCATCGGTATTCCAATATGAGGTTTGTTTGGTTGTCGTCATGTCTCATATCCCGTGGAGGCTGCAGGGGTGTTTCTCATGCTCTGCCTAATGTAGTCGTGGAAGTATTTGGAGCCGATGGTGATGATGACGAAGACACTGTCACTGGGGATTATGTTGAGACATTGTACTTGTATCGATCCCTTGATGGCCCAAGGAAGGATGCTCCAAAGTTTGTAACAATTTTATGAGAAAAACTTTGGTGCAAATGAGGCCATGATTGGGGAAAAGCAAAACTAGCAAGCAGAAGATAATGTGTTTGTAAACTGTAAGTGCTTCAGGTACACTGCATGATTTGTTTTGCCATTTTCACTCTTCTTTGGATTCGTTATATACACCAAGAAGAGTATTATAGCTCAAAGGAAGACAATGTATGAAAATGAACGAAAGAATCGTCTATTATTGTTTGTGTTGTAATAATTGTTTTTTCGCAAACATTGCCAGATTTATTTACAGTCATTGCACGATTATTATCCAAAGAATTATTCGTTATACATAACATTTTAAGCT

>BdCOI1 BRADI2G23730.2

CCCCCAGCCCCTCTCCACTCTTCTCCCTCCAATAATTCTCTCCTCCAGTCCTCTCTGCCTGCACCACCACCAAGCACCATCAGAAAGGGAAAGGGGGGAAATCCCCATGTCTCCACCGGATCAGATCCCCGGCGGAGAGCCTTAGACGGAGAGCGAGCGGAGGTTATCTGATCCGCTGCTCGATGGGCGGGGAGGCCCCGGAGCCGCGGCGCCTGAGCCGCGCGCTCAGCTTAGATGGCAGCGTCGTCCCGGAAGAGGCGCTGCACCTGGTGATGGGATACGTCGACCACCCGCGCGACCGGGAGGCGGCGTCGCTCGTGTGCCGCCGGTGGCACCGCATCGACGCGCTCACCCGGAAGCATGTCACCGTGCCCTTCTGCTACGCGGCGTCCCCGGCGCGCCTGCTCGCGCGGTTCCCGCGCCTCGAGTCGCTCGCGGTCAAGGGGAAGCCTCGCGCCGCCATGTACGGCCTCATCCCCGACGACTGGGGCGCGTACGCTCGTCCCTGGGTCGCCGAGCTCGCTGCGCCGCTCGAGTGCCTCAAGACGCTCCACCTGCGGCGCATGGTCGTCACCGACGACGACCTCGCTACTCTTGTCCGCGCCCGCGGCCACATGCTGCAGGAGCTCAAGCTCGACAAGTGCTCTGGCTTCTCCACCGACGCCCTCCGCCTCGTCGCTCGCTCCTGCAGGTACTGTTCTGTAATTTCTCGATTTACTAACCCCTTTTTGCTCTGATCTGCGAGTGTGAGAGGTAACCCGGAATTTGAGTTTGGGGATCGGGGGTACAGTGACTTGGTGGCGAATTGAGCCGTTGTTTAGTTGATGTCCCGAAACAGCGGGCGCAATGATAGATAGAGCACCGAGCTCGTTAATCAGTTTCTGTAAATAGAAACTTGTTTTATGACGGTTGATGTTTGCCAAAGGTAGTTCTAGATCTGAACGCCCATTGTAGGGCATATTCTCCTCACATGATGAGACATGATGGTAGATTGGACAGCCGTACAACAGGACAAAAAGACCTGTATCTGCGTGGTTTGAGTTCGTTGCCTTACAAGTGTAGTATATCTATTCATAAGTGATTAATTGCATGCTTTGTATTTTGCTGTTGATCATAATGCACAAGAGTCCGATGCACAGCATTTATTTTTCTGACCAATTCGCAAGAGGTCACCAAATCTGGCAGCAACAAAGTGTAAAAATGCCAAAGCAGTAGTGATTGACTTGACCAAAGCTAGCTCGTCTTCTATTGGAACGTACTAATGTACAGGATAAATAGTCAAGTTGCTGGTTGTTTCTACTATCAGTACATTCTATCATGAACAACATTGTGTACCAAGGATATGCCAGGCTGATCATATTTACCTGTCTCTCTTTTTTTTCGAAAAGGAAGAGGAACCCCCCAGCCCCTGCATCAATTGAGTACCTAAGATAGTTTGTCTTACTAAAATGACATACTCCCTCTGTCCAACAAAGGATGTCTCAACTTTGACCAAATTTGAATGCATCTATACACTATGTCATGTCTAGATACATCCAAATTTTGACAAACTTGTGACATCTTTTGTTGGACGGAGGGAGTACATTTCTTTTCCATGCTGATCACCAAAATTGGGACTATTTATGCTACCATAAGCTAAAATTGCTTCTCTTCAATGCACTCCTTTGTTCCATGATCTTTCTTTTTGGTATATTTTGTTGGTAATGCCGTGATAAGATCACTCCACTAACATGTTGCACTAAATTCAGCAGATCACTGAGAACTTTGTTTCTGGAAGAATGTTCAATTACTGATAATGGCACTGAATGGCTCCATGACCTTGCTGTTAACAATCCTGTACTGGTGACCTTGAACTTCTATATGACTTACCTCAGAGTGGTGCCAGCTGACCTCGAGCTTCTTGCCAAGAACTGCAAGTCATTAATTTCATTGAAGATTAGCGACTGTGACCTTTCTGATTTAATTGGATTTTTCCAAATGGCCACATCATTGGAAGAATTTGCTGGAGCGGAATTTAATGAGCAAGGGGAACTCACAAAGTATGGAAATGTTAAATTTCCTTCAAGGCTTTGCTCCTTAGGACTTACTTGCTTGGGGACTAATGAGATGCATATCATCTTTCCCTTTTCCACTGTACTCAAGAAGCTGGATTTGCAGTACACTTTTCTCACCACTGAAGATCATTGCCAGCTCATTGCAAAATGTCCCAACTTGTTAGTTCTCGCGGTAATGGTGATACATGTACCTTGTAACATATTCAATTAGTGCTCTATTCTTAATTGTAACTAACATCCTAAATATGCTTTACAGGTGAGGAATGTGATTGGAGACAGAGGATTAGGGGTTGTCGCAGACACATGCAAGAAGCTACAAAGGCTCAGAGTTGAGCGAGGGGATGATGATCCTGGTCTGCAAGAAGAGCAAGGAGGAGTTTCTCAGGTAGGCCTGACAGCTGTAGCCGTTGGTTGCCGTGAACTGGACTACATAGCTGCCTATGTGTCTGATATCACAAATGGGGCCCTGGAATCTATTGGGACTTTCTGCAAAAATCTGTGTGACTTTCGGCTTGTCCTGCTTGATAGACAAGAGAGGATAACAGAATTGCCACTAGACAATGGTGTTCGTGCGCTGCTGAGGGGCTGTGCTAAGCTTCGGAGATTCGCGCTATACTTGAGACCAGGGGGGCTTTCAGATGTAGGCCTTGGCTATATTGGACAGCACAGTGGAACTATCCAGTACATGCTTCTGGGTAATGTTGGAGACACAGACAATGGACTGCTCCTTTTTGCGTCTGGGTGTGTGAACCTACGGAAGCTTGAATTAAGGAGCTGTTGCTTCAGTGAGCGGGCTTTGGCTCTTGCTATGCTACAAATGCCTTCTCTGAGGTACGTATGGGTACAAGGTTACAAAGCCTCCCAAACTGGTCGCGACCTCATGCTCATGGCCAGACCTTTCTGGAACATAGAATTTACACCTCCCAGTCCTGAGAATGCAAATAGGCTGACGGTAGATGGGGAGCCTTGTGTAGATCGTTATGCTCAGATTCTTGCATACTACTCCCTTGCCGGAAAGAGGTCGGACTGCCCGCAATCTGTGGTTCCTTTGTATCCTGCGTGACTGTAAATACACTAAGCCGGCACCTTTCTGCTATGTTCTAGCCCTTTGCTCTCTTGGGCCATAAGGATGTTTGTATGTGGTTATTGTATGGACATATGGCTAGCTGGTATGTACTGGAATAACCGAATAAGCGCATGGTATTCTTGCCTGTACTACTATCTAATCCTAGGAAGATGTATACTGAGTAACACTGCATCTGTGACACTACAATGCCTGCTCCCCAGATTTGTACCATTGGAGGTGTAAAAGTACCTGTTTTTGGCAGCCTATGTAATACGTACTTAAGATGTTCTCTGTTGAACACAAATGCTCCCGGATAAACCTCGTTGAGTTGCATCTTGCATGCTAAATTTTCTGTCCTGAGAGCATGATGTGTTTATCAACAACGACCCGATGGAACTTCTGG

>BdCOI2 BRADI3G58320.1

CGCCCATTTCCAAAAATCGAAACAGAAGCGAGGGGAATAAAAGGGGGCGTCCTTCGCTTCGCTCAGGCTTCAGTCCGTCCAACCCCGCGCTTCTCAGATCTAATCATTCTTTAATCCATTTACCTACATACAGTCTCACATGAGCACTCCCCGCTCCTCCGCCTCCTCCCCCGATCCGATCCCCCACAAATCCGTAACCCTAGCACCCTCCCTCGGCATGCGCGATCCGGAGGAGGACTCGGACTCGACGCCGTCGCGGATGTCGGAGGACGACGACTGCTCCGGCGGGGGAGGCGGCGGCGGGTGGACGCCGGATCTGAGGGGCGGCGGCGGCAGGTGGGCGCCGCCGGACCAGGTGCTGGAGATCGTGCTCGAGAGCGTGCTCGAGTTCCTCACCGCTGCGCGCGATCGGAACGCCGCCTCGCTCGTCTGCCGCTCCTGGTACCGCGCCGAGGCCCAGACGCGCAGGGAGCTCTTCATCGGCAACTGCTACGCCGTCTCGCCGCGCCGCGCCGTCGAGCGGTTCGGGGGCGTGCGGGCCGTCGTTCTCAAGGGGAAACCGCGCTTCGCGGACTTCAGCCTCGTGCCCCACGGCTGGGGCGCCTACGTTTCGCCCTGGTTCGCCGCGCTTGGCCCCGCGTATCCCCGCCTCGAGCGCATCTGCCTCAAGCGGATGACCGTCTCCGACGACGAGCTCGCGCTCATTCCCAGGTCGTTCCCGCTCTTCAAGGAGCTGTCGCTGGTGTGCTGCGACGGATTTAGCACCCGCGGCCTCGCCATCATCGCCGAGGGATGCAGGTACGCTTCTCGTTGAAAGTCCTCGCGTTCCCGGATGATGCACCTGAGCTGAATCTGAACCGGCTTATGGTATCAACGTACTACCAGCTTTCCGGACTGGGAGGGTATCATTGGTGCATTTGATCAATTCCTAGCTGCATCATGCAATTTCGACTTTATTTAATGTACACGCCTAGTCCTTTACGCTTTTCAGTTTGATTGACCCGACCCTGCCATGGATCAGAATCTGATCTTAGGCTAGCTTTTATGCCCGTCTTGGGTTCTCATCCCTACACGTTCTGTGGAAATTAGATCTTTATTGTCGTTGTGCTCTACTAAGTTCGTGACACTTGGTAGTGTCAGTCGAATCATGCCCGTTGGGAGTCTCCTGCTTCCAGTTTATGGCCACAATGTTTGATTTCTTTAAGTTTATCGCGAAGTAGTTCTAAGCAAACTTCCCAAACTGATAACTCATGAATCTTCTCATTTCTTTCTTAGGCATCTCCGAGTGCTGGATCTGACTGAAGATTATTTTCATGAGGAGGAGAACGAGGTAGTGGATTGGATCTCCAAGTTTCCAGAGAGCAACACGTCGCTGGAGTCGCTTGTATTTGATTGTGTTAGTGTACCATTCAACTTCGAGGCCCTCGAGGCACTTGTTGCACGGTCACCAGCTCTGCGTCGGCTGCGTGTGAATGACCATGTATCGATAGAGCAGCTGCGTTGTCTCATGGCAAGGGCACCCCGTCTCACGCACCTTGGTACTGGTTCATTCCGATCTGAACCAGGCTCTGGCGGCACATCATCTGTGTCTGAGCTCGCCACCTCTTTTGCAGCATCGAGGTCACTAGTTTGTTTGTCAGGTTTCTTGGATGTCAATGCAGAATACCTCCCAGCAATTTACCCAGTTTGTGCCAATCTCACATCCCTCAACTTTAGCTTTGCGAGCCTAACTGCTGAGGAGATCATACCAGTTATTAACCACTGCGTCAGTCTTCGCACTTTCTGGGTATGTATTTTGTGACCTTTTTGTTGTCACATTGATAGGTATGTCGGTTTACCTACTTCTTCATTAACCGTTGTTGTTCAAATTTCATACTCCAGGTTCTTGATACAGTGGGTGATGAAGGCCTTCGGGCTGTGGCTGAAACATGCTCAGATCTCCGTGAGCTGCGTGTTTTTCCTCTCGATGCCACAGAGGATTCTGAGGGCTCAGTCTCGGATATTGGTCTTCAAGCAATCTCAGAAGGCTGCCGGAAGCTCGAATCGATTCTCTACTTCTGCCAGCGTATGACAAATGCAGCAGTAATCGCTATGTCTGAGAACTGCCCTAACCTTGTGGTGTTCCGCCTCTGTATTATGGGCCGTCACCGCCCTGATCGGATTACAGGGGAGCCCATGGATGAGGGTTTTGGGGCAATTGTGATGAACTGCAAGAAGCTCACTAGACTTTCAGTCTCTGGCCTGCTCACTGATAAAGCGTTTGCATACATCGGGAAGTATGGAAAACTAATAAAGACTCTGTCTATTGCCTTTGCCGGGAATAGTGACATGTCTCTTCAACATGTCTTTGAGGGATGCACTAGGTTGCAGAAGCTTGAGGTCAGAGATAGCCCTTTCGGCGACAAGGGGTTGCTCTCTGGCATGAACTATTTTTACAACATGAGGTTCTTTTGGATGAACTCCTGCAGGCTAACCGCGAAGGGCTGTCGAGATGTAGCTCAGCAAATGCCAAATTTGGTGGTGGAAGTAATGAAGGAGCATCCTGAAGATGAAGGGGAGACAGATACCGTTGATAAGCTGTACCTGTATCGATCACTTGCAGGACCAAGGAATGATGCTCCATCATTTGTGAACATCTTGTAGGAGTGCAATTGAAGTAGTATGTGTATGGACATGTTTGTGCTATTATTCCTGCTAGCTTGTATTCCTGATCAAGCATATTTTAGAGGCTCAGAAGCAGGGATGGTGAAGTTTCCCGCGGATGATTTTGAAAATGTCATGGGTAATATGCACGCATCGTTCAGTTAAGCAGAGCATCGCTTATTCTTTTCTGGGCTGATTCCGTTGAGATTTAACCTGTATGGGAGCAGCCCATGCGATTTACCTGATGGTGAATGGAAATCTGAGTTATGACTGTCCTTGCCAGCATCAGACTGATGGCTTTGTGCACTATAATTGAGTAGTAGGAACTAATTGCACAGGGACTGGCTGCTGGCCTAGTTAAATTCGGCAGCATATTTTAGATGGTTCATGTGCCCTCAATATTCTCCGGACACTGATGGTTTTGTCATCTATTTGTTCAATTTATCTGCTCACATGAAGGAATGACAATGTTCCATGAACAGTTGCCTTGTAATCTCAGCTATGATAAAATTTTCGCCTTCCCATGTTTTTAGTGTCCTTTCCACAAGTTAATTTTTGTTAATCATACTTGATCTCACCTTCTGCTGTCATTAATCCAGAAGAGATTTTGGTCGTTTTCTCCTTGTGTTGCTACTGTTCCATTGCCATGATCA

>BdCOI3 BRADI4G05157.1

GGGCAATGTGGTCACCTTGTCCGTCTCTCTCAGGTGTTACCTTCCTTCCCCGTCGCCTGAGGGCTGAGGCAGGTGCTACTCCGACAGGCACTACCTATCCACATCTCCATCTCTCTCCTCTTTCCCTCACCTCCGTACCCTCCCCCAGTCACCACCGGCCGCCTGCCTCCTCCCCCCTCCGCCGGCCGGATCAGGAAGGATAGCTCCGCGCGCCGCCTCTGCCCTGCGGATCCTACCATGCTGAGACGCCCCGCCTTATACCGACCCCGCAGGGCCACCACACCGCGCCACCCGATCCCCGACGGCCACCCCTTCGTCGCCGTCTTCTCCTTCCTCTTCATCCCCACCTAAGCTCCACGCGTCCGGCGCGTCCTGTCCCCCTGCCGCCGAGCTTTCCTGGTGGAGGAATCGTCAGCTGTTCCTACGGGGTCGATCCTCGATCGATCGATCCCGGAGATGAGCGAGGATGCGCAGAGGTACGGCGGCGGCGGCGGCAGCGGCGGGGGCGGGAGCAGCGGCGGCATCGGGGCGCTGTCCCTGGATCTGCTCTGCCAGGTGCTCGACCGCGTGCAGGAGCGGCGGGACCGCAAGGCGTGCCGCCTCGTCAGCCGCGCCTTCGCGCGCGCCGAGGCCGCGCACCGCCGCGCGCTGCGGGTGCTCCGGCGGGAGCCGCTCCCTCGCCTGCTCCGCGCGTTCCCGGCGCTCGAGCTGCTCGACCTCTCCGCCTGCGCCTCGCTCGACGACGCGTCTCTCGCGGCCGCCGTGGCCGGCGCCGACCTCGGCGCCGTCCGCCAGGTCTGCCTCGCGCGGGCCAGCGGGGTCGGGTGGCGCGGCCTGGACGCCCTCGTCGCCGCCTGCCCCAGGCTGGAGGCAGTCGACCTGTCGCACTGCGTCGGCGCTGGGGACAGGGAGGCCGCGGCTCTGGCGGCGGCCGCTGGGCTGAGGGAGCTGAATCTGGAGAAGTGCCTCGGCGTCACTGACATGGGCCTCGCCAAGGTAGCTGTGGGCTGCCCCAAGCTTGAGAAGCTGAGCTTCAAGTGGTGCCGTGAGATTTCCGACATCGGTGTTGATCTGCTCGTCAAGAAGTGCCGCGAGCTCCGCAACCTTGATATCTCCTACCTAGAGGTTATTCCCATTCGAACTATTCAATTCTTATTTGATTGCTACTCCATTTATTGATTGGTTCGGTTTAGCATTAGTATCATTATCCTTGAGAGAAAGGTGAAAAGCATGAGCTGGCGACGAATATACAGAGATTTTTTTCCTGTAATAACAGAGGCCACCCCTCCGGCTTCATTAAAGGAAACCAGTGTCTTACTTACATAAATACGAGTACTAGTTTAAAGCAAACAAACTGAAGAGCGACCTCAGATCAGGCGAACCCTAAACTAAATCTTCTGGAACCAGGACAATCTGCAGAACTAAAGTTCTGAACAACGACATCTTCCAAGCAGCACAACATCCCCCAGCTCACACATGAACCACTTCTTCAGATTGTTAATTGCTGCGTCACAGGGGCCCAACCTTGCGAATGACTGAACACTTGAGCCGTGACTTGCCATAACCGCCGCGAGCACAACGAATTTACGGAGTAAATGGGGACTTCGTCGTGTACTGATTGCTTTTGAATCAATAAAGCATCAAAACAAAAAATTGCACTTATTGGGCACTCTTCCAATTTAGAAATTGAGGCATTTGAGTGAAAGAGGTGCTTCTCAAGATAGTATGGGTCAAATTTATTGGAGATGTTTTTTGAGGAATGCAATTGGAATACGAAAATTTGCTTAGATCTTTCTTCTGTTTGCTAGCATATAGGTTGCTAGAAAAAAAAAGCCTCCTGACATCTTGGATCACTCTTTGAGTTGCATCTTCCTTCTGAAGAAAAGTTAAATTTTGGTACCAGATGGCAAAAGTACATTTGCGAAAATTGAATCATGTGATAGTAGAGTTATAGGAAGGGTGGATTTTAATCCGGACTGTCTTTGGTCCTTTTGATTAGGTAATTATGATGCACCAGTAGAAGTTTACTTCAGGATATTATGAAACTGTTTCTGTTGAGTCTAGACTCTTTTACAGAGTGAATTGGTATTGAGTGCGATTTAGATAAGTATTTCGGGCACATTAGGAGAAGTTTTATTTTGGTACATTTTCAGTCATGGAACTGAATAGAACTGGATATGTTCATCATTTGGCCATGTTCTTCAGTATTAGATTATAAAACTTTAGCCCACCCTTTCGCATGCTGCTGGAAAAGGATGAGTAGTCAAAATATTGAGTGGTGGCAATTAGGCCGGGCTCAGGGATAGAGTAGACTAAATATTCCTTCGGTCTTAACTAGCACAATATCCATGCGTTGCTATGGACTACACAAAATAATATGTTCAAAAAATGTTATACTATTAATCATCGATTATCCTATCGTATAATCCATATTCATTTGCATCTCAATGATTGTGCCAAATCAACAAGTGAAAGTGATGGAAAATAAAAATCAAGTGACGCAGTCTAAATGTGACAGACCGCTGCCATCACGAGTCTTCTATATCTGTAAAGATGTGGTGCAATTTGTAAGAAAAGCTGTACCAAAGCTATTGGAATGAGTGGACGTAGATGCATCTTTTTGGTATATAGTGTTTGTTTTGAAATCCATGTTATCAAGTTTCATGCACGTTCAACCGAAAGCCTAATCTGGTTAGGAAAGATTGCCAATATAATTCTACTTCGAAACATGATATACCATTCTCATGTTTCAGGTAGGAAGGACTCCTGTTTACAGAAAATACACGTACTATTTTTCTAGCTGTGACAAACGTGCAGTAGGACTATAAGTTAGTATTTCAGCACTATTTCATTACTTCTTAGTTTCATCTACTACTGGCAGGAAGCAGGAACAGTTTAGATCAAAAAGTGTATAGTTATTTACTCTTCCTATTGGAGATTATCCTGACAATGGATTGTATACTCACATGAGGAACCTGCGGAGTACTTAAGTTATTAGTTAGATTCCATCGTGAAGATCTAAAATTTCGAAAAACACCTCTCTTTTTTTCTGCTAAGTTTACTCATGAAGGTACTTACATTTTATTTTTCTGTCGCAGGTGAGCAATGAGTCTCTTAGATCAATATCGACTCTTGAAAAGCTAGAGGAGTTGTCGATGGTTGGTTGCTTATGTATAGATGACAAAGGCCTGGAATTGCTAAGCAGAGGAAGTAATTCACTGCAGGTAATCACTTTGCAGGGATGCTGTTGTTTAGTCTCATGCAATTGTTATGTCTTTGATCATCTGAAGTCATATTGCTTAGTCTCTGTTACTTGGAAATATTTTTGAGTGTTCAAGTTTACCAGCATTTGAATGTTTCATTATTCTTTTCGATATTAGTAAGCATCAGTCCATTCTAAATATTGTCTTATGTTGCAGAGTGTTGATGTGTCAAGATGTGATCACGTGACTTCCGAAGGCTTAGCTTCACTCATAGATGGTCACAGTTTTCTCCAGAAGCTAAATGCTGCTGATAGTTTGCATGTGAGTTAGTACTCCTAATGTTACTTGCTTGGTGCACACGTTGGCTACATCTTTAGATTAAAAAAGCTGGCATGTTTCATTGAGAGAGACTCATCCCTCATGATGAATAAATAAGTAAATTAATAATGTAAAGGACAGAGGACAACTACTTAATTGCCAATGATTGTTGACCTTCTATTTGGTTGAATCTTGTAGGAGATTGGGCAGAATTTTCTATCCAAGCTAGCAACACTGAAGGAAACATTGACAATGTTGAGACTTGACGGCTTTGAAGTCTCATCCTCTCTTCTTTTAGCGATTGCTGAAGGCTGTAACAACTTGGTTGAGGTTGGACTTAGCAAATGCAATGGTGTTACAGATGAAGGCATCTCTTCGCTTGTAGCTCGGTGTGGCTACCTAAGGAAAATTGATCTCACATGCTGTAATCTACTCACTGACAATGCCCTTGTTTCAATAGCTGACAACTGTAAGATGCTTGAATGCCTCCTGTTGGAGTCCTGCTCTTCATTAAGTGAGAAAGGACTAGAGAGAATCGCTACCTGCTGCCCCAATCTTAGTGAGATAGATCTTACTGACTGTGGAGTGAATGATGCAGGTATGTGCTGCCGCTTATTACATGTATGATTATTTTTCATCTAAGCCTGTCTCATGATTTGAATATTGACAAATAAAATCAACTCCATTGCAGCGCTGCAGCATTTGGCTAAGTGCTCTGAACTGCTGATATTGAAGTTAGGCTTGTGCTCAAGTATTTCTGACAAAGGCCTTGGTTTTATTAGTTCGAAGTGTGTAAAACTCACAGAAGTTGACCTCTATCGGTAATTTGATCTCCTGGTCTATTCCTTTATAGTGAGTCCCAGCATCTTCTATGCATGAGAATAACCTGTATTTTATCTTGCTATATGCAGCTGCAACTCTATTACCGACGATGGGTTAGCAACTTTAGCAAAGGGCTGCAAAAAGATTAAGATGCTGAACCTGTGCTACTGCAACAAGATTACTGATGGTGGTTTGAGCCACCTGGGCTCTCTGGAGGAGCTTACAAACCTTGAGCTGAGGTGCCTGGTCCGCATTACAGGTATTGGAATCTCGTCAGTTGCCATTGGTTGCAAGAGCCTGGTAGAAATTGACTTGAAGCGTTGCTATTCTGTTGATGATTCTGGCTTGTGGGCTCTCGCACGATATGCCCTAAACCTTAGACAGGTAAATTTATCCTTAGTTTGGCTTCTTCCTTTACGCAAGTTCTGATGAGACAGGAACCTCAGTAGCTTGTCGTTTTCTGCAGCTTACCATATCATACTGCCAAGTTACTGGCTTGGGATTGTGCCACCTGCTGAGCTCCCTGAGGTGCCTCCAGGACGTGAAGATGGTGCACCTCTCATGGGTCTCCATAGAAGGGTTTGAGATGGCACTGAGAGCTGCTTGCGGCAGGCTGAAGAAGCTGAAGATGCTTAGCAGTCTGAAGTCTGTGCTGTCCCCTGAGCTGCTCCAGCTGCTGCAGGCCTGTGGCTGCCGCATCAGATGGGTCAACAAGCCTCTCGTCTACAAGGATGGCATCTGAGCCTGAGGTTTGAGGAGTGTTAAGATCTACATATCGTCAACATCTGAACCTTAAGTTCCCATGGCTTTATAGCTAATTCTTGATAATAATCGCATGGTTTGCCTGTTCTCATCCCTGAGATCAAGATGGTGTTCGCGTTTTATGATGGTGGGTAAACCTGGCATCTGATGTGAAACATACGATATGTAACACTGAAGCCTCTTATGCTGCGCAACATGGCATCTGATGCCGAACATGTAATAGGTACTCCGTACAACAGAGTGCTCAACATGTGGAAGATGGCATATCAATGACCCAAATTTGTGTATAATCCCAGGTGGAGAGTTGTTGTGTTTCCATTGTTTTGTGATGTAGCTCTGTTGATTTTCATCGGTCCAGGCAATGGCACTAAAACTGGTGATATATGAGTTGCACAGATGAGGAAGAGTTGTTTTATTTCCATGTGAGCTTCAGAGCCTGCACGTGTGCAAGAGGCAGTCAGCATTTATATTTTTTTGTTTGTCAAATAATATCATGTCTGTAAAGTCAGATAGACCAAAACAAAGCAGATGCTTCTACTAAGACAAGTGCGGGTAGGAATTTTCCCGA

>BdCOI4 BRADI4G06170.1

GGGGTCAGCACCGCGCAGGGGCATATAACATGGTATAAGACGACAGAGGAAGCGCTTTCCCATTGAAAAAAAGGGAAATCATCATCCCCTCAAAAGAAATGCAAGCGAGACGAGGGTTCTAGCGCTGCTCCATTGCAGACTTGGGGGGAAAGGGAGGGTCGAGGGCCCGAAGGACCCAAGATCGATCGGAGGCCGAACCGCCATCGCCGCCGTCTTTATCCCCTTCCTCTCACCTGGCCGTGCCTCCGAAGCCGCATCCATCGCCGCCGCCGCCGCCTCCAGGTCCGAGCCAGCCGCGAGCCCGCGACACGCCCTTGTATATCCCCTGTAAGCCCCGCTGCCATGGAGCCTTCTCCCGTGACCGACAGCGAAGAAACCGAAGGCGTTCGTTGTGGATCGGGCTCGTCCGTTCGCCGGCCGTCGCGTCGTCCCCAGCTGGCCGAGGGGGCGAGGGTTCTGACGGAGAACGCGACACGGGTTGCTGCGGCGAATCCAAGGAGAGCCAGGCTCACGGTGTTGATGCCCGTGCGATCTCCGATTCTCAGGATGATGCCTCTCAGGGCGAGGTCTGCGTCAGCGACATTTCGGACATCCCCGTCAGCAAAAGCCCTGCGAGCTCCCGTAGCCATCTCAACAAGTATGGCTCTTCCATTCCTGATAAAAATTACCTGCCTGCTGCATCTATGACTTCGCGTAGCAACTCTCCAAGTTGTAGGCCGTGTGTTTGGAGCAAACAATTTTTTGATTCTGTTAATGCTACTTGCCTAGGAATCATAACACCTCAATACATGCTCTGGTCTTTTCATGTGATGCTGGTCAGTAGCGAGTGTTGCCTTGCGGGCTTGTGTTAATTCTAATTATTAGGTTCTCTGTAGGTACCTTCTTTCGTTCACTATTACAGCATGCTCAGCTTTTGATGATGTGCTTCGTTAATACAGATATGGTTCTAGCTATTTGATGTAAACCAACACTCTTTAATTAATGTTCAGTCTGATGAACACACATATTCTTAACAGATACAGGAAAGAATTCAAATGTTCATTAGTGTACATATATTATTTTCATTGAATGTAATCTCATTATCTATGATCTCATTAACTTTCAGTCCAGCGCTTTGTATATTCGCCGAACCTTCAGATTACCCAACCAGCACAGCACCATTTCTCATTGGAGGATCTCCCAGATGCAATGTTAACAGAGATCATCAAGAGGATTACCCTGACAAGTGATCTGAATTCTTTTTCCCTTGTGTCGAAGCGACTCTACACCATTGAGGCAGATCAGAGGGGTGCTATTCGTGTTGGTTGCAACCTTTGCCCTGCTACAGAAGCCTTGGCATCACTCTGCACACGGTTCCCCAATCTCTGGAAAGTGGAAATTGATTACTCTGGTTGGACGCCAGGACATGGGAATCAGTTGGACAACCAAGGGTTCCTTGAGTTTTCATCTCGCTGTCCCTCGCTTACTGATCTGACCTTGAGCTTCTGCTCACGCATCCATGACTCTGGTCTTGGTTGTCTAGATTATTGCAAGAAATTGATGTCCCTTAGGCTGAAATCCGCACCAAAAATAACTTCACGTGGGCTTCTCTCGGTTGTAGTTAGTTGCAAGAGTCTATCTACTCTCCATATTGTTGATTGTCACAAAATAGGCAGCGCAGATTGGCTGGAGTACCTTGGTCAGAATGGATCACTGGAAGAACTTGTAGTAAAGAATTGCCAGAGAATTAGCCAGTATGACCTTCTAAAGTTTGGTCCAGGATGGATGGAGCTCCAAAAGTTTGAGTTTATGACCGATGCAGGTTTATGGGACCATCTTGAAAGATGTTATGATTCCTCATACAATGCTCACGACCCGAGTAGATATGATTTTCACTGTGATAGTTTGAAGGACTTGACATTTGCACGTTTTACAACTGGGCCAGAAATAGGGCTACGTAATCTTCTGGGCAAATGCAAAGCACTGGAGAAACTGCGCCTTGAATATGTTCATGGCCTAAATGACAATGACTTGATTGCGCTATCCAAGAGCTGCAGCAAGCTTAAAAGCATCTCACTTTGGCTCACACCAATTTTCCATGATTTCTATAAGTGCACAACATCATTTACTGATGACAGCCTTAAGGCCCTAGCCCTCAACTGTCCTATGCTTCAGACTGTTGAACTCACGTTTGGAGACTGCGAGCCCACGTACCCTTCAGAAATAGGCTTCACAAGGAAGGGCCTTGTGATGCTCATGAAGTCTTGCCCAATTCGTGTTCTTGTGCTAAATGGGGCCAACTTCTTTGATGACAAGGGAATTAAGGCCCTTTCATTTGCACCATTGCTAGAGACACTTGAGCTTATTGACTGCAAGGCGATAACGGATATCGGGATGCGTTTCATTGTGCATATCCCACGCTTGAGTAATCTGGCACTCCGGTGGTGTGAACATGTAACTGATGATGGAGTGGCTGAACTGGTACAAGCACACAAGTTAGAGTCTTTGAGCATTGAATGTTGTCTTCAGGTTTCTCTGGAGGCTGTGCAGGGGGCTGCCAGATCAGTACACTACTACACCAAGTGTGTATCCAACAGCCTTTTGGGAAAAAGAATGTTCTTAAAATACTGCTCGTGAAGTGGTCTCCAGATCGTCAACCAGAATCACCTATTTTGTTGTCAAAACCATTGTTTTATGTTACTTCCCATGTACTTTCTGTACTTGTTTGTACTTTCCCCGTCTACTGCTATAATTCTCTTTAATGTTGGGGAAGGCTAGTGTAAGAATATGGAAGGTATCTGATGATTCAAAACTTTTAATGGTAGCTCTGCAGCTCGTTCTGTAAATCTAGCAGCTTAACTTGCAGTTTCTTTACCTTCTCTGAAATCTGCACAAAATGCCCCAAGATACCTGTTACAGATCTTCTGTAGCGTGACAGACACAAATTAGATTCAAGCAGTTTTTGGTAGAAATATCTGGTTGTTAGTTTCTGTTATATGCAAGGAAATGCTGTGATTATGATCATGATTTGTACAAACCGATGAACGGAGCA

>BdCOI5 BRADI4G08400.1

TCCTCCGCCTCCCCCGTACACTAGATCCGCCACCGCCGCCGCCACCCCTCATGGGCCAGTGCCCCTCCGCATCCCGCCATCACCGTCACCATCGCAAGCTGCCGCCGCCGCCGTCCCCCGCCGCGCAGGCGACGTCGCCTCGTTTCTCCTCCGATGGTGCCGCGGCCGCTGCAGAGGACGCCGCCGCCGCGGCGACGGCGGACTACACCTCTGATCTCCCCGAGGAGCTCCTGGCCGTGGTGTTTGGGTTTCTCGGCTCGGGCGACCGCAAGCGCTGCTCCCTCGTCTGCCGCCGCTGGCTCGCCGCCGAGGCTGCCTCGCGCCTCCGGCTCGCCCTCGACGCGCGAGCGCCGCTCCTCGCGGCGGCCCCTGGGATCCTCGCGCGCTTCTCCGCCGTCTCCAAGCTCGCGCTCAAGTGCGACCGCCGCGCCGAGAGCGTCGGCGACCCCGCGCTCGCCCTCGTCGCACAACGCCTCGGCCCCGGCCTCCGCCGCCTCAAGCTCCGCTCCGTCCGTGCCGTCACCGATCACGGCGTCGCCACGCTCGCCGCTGCCGCCGGCAACCTCCGCAAGCTGTCTGTCGGCTCATGTGCCTTCGGTGCCAAGGGGATCGAGGCAGTCCTCCGCTCCTGCCCCCAGCTCGAGGAGCTCTCTGTCAAGCGCCTGCGAGGCCTTGCTAACTCGGAGCCCGTCGCAATCTCTGGCCCTCGTCTCCAGTCTCTGTCCCTCAAGGAGCTCTACAATGGCCAGTGCTTTTCATGTTTAATCACGCAGTCCCCTAACCTCAAAACTCTCAAGGTAATCCGATGCTCTGGCGATTGGGACCCCGTGCTGCAGGCAGTCCCGCAGGATGCTTTGCTGGCCGAGCTTCATCTCGAGAAGCTGCAGGTCAGCGACCATGGCGTCAGTGCTCTATGTGGACTCGAAGTCTTGTACCTTGCCAAGGCCCCAGAGGTCACAGATGTTGGGTTGGCAGCGCTAGCCACCAAGTCGCCACTCTTACGCAAGCTGCATGTTGATGGGTGGAAGGCGAATCGGATTGGTGACCGGGGGCTTGCAACCGTGGCACGGAAATGTGCTGCTTTGCAAGAATTGGTTCTTATTGGTGTAAATTTGACATCAGTGAGCCTTGAATTGATAGCTGCTAACTGTCCCACCCTCGAGCGGCTTGCCCTATGTGGGTCTGACACATTTGGGGATGCGGAGATCTCTTGCGTGGCGACCAAGTGTGCTTCTTTGCGGAAACTCTGCATCAAGGCATGCCCTGTGTCTGATGCTGGAATGGATAAGCTCGCTGAAGGCTGCCCACGCCTTGTCAAGGTGAAGGTGAAGAAGTGCCGTGGGGTGACGTTTGAGTGTGCCGAGCGGCTCCGTGCTAGTCGTCATGGAGCCCTTTCTGTGAATTTTGACACACCGGGCGTTGCTGGTGAATTGCAGGATGCTAGGAGTGTGGATGAGAGTGGTGTGCTGGAAAATGCAGGGAGCGACACTGTACCAGATGATCTTGATGATCAGATAGGGATCCCTGACCTTTCTTGTGGCAGCAGTGGCAGGCCATCAGGGTGGAAAGCACGACTGGGTGCTTTCATGTCAAGGAGTTTGTCAGTTTCCGTGTTCCGGAGGCGTCTGCAGGCGAGCTCGTGTAACTCATAAGTCAGCGCACGAGGATTGTGTGCTATGGTTGCTCCTTTAGCCCTTTGATTCTTTTATGTTTGTATGTCAATTAGTTGGTGCTGTGCTTGTTTGTTAAGATCGATGTTTTTTGTCTTTTTGAATTGTACCATTCTGTTCTTCCAGATTGCTGATGGAAAATTCAGTTTATTTTGCCATTCAGTTTGGTTCGAGGATACTTATTATTGATCTTTCGCTTTGTAGTAGAGGAAACATCCATGTGATCATACAATGTAATCTCTGATAAACAATTTTTTCAATTTAAAAACGTGATATGTGAAGC

>BdCOI6 BRADI4G11900.1

GGCAGCAGGTCCCCTTGGTCTCCGCCCCCTCCAGCTCCTCCAAAACTCCCCGCCGCCCCCCAGAGCATATCCTGCTCCGGCGGCGATGAGGGGCAGGCGCGTCGACCTCATAAACGTGGCCCTCCCGGAGGAGCTCCTGGAGGAAGTGCTCCTCCGCGTGGGCGGCGCCAAGCGCGACCTGGACGCCTGCGCCCTCGTCTGCCGCCGCTGGCGCCGCCTCGAGCGCGGCACGCGCCGGTCCGCCAAGCTCCCGGCCTCCGGGGCTGGAGCCAACGAGCTGGCCCGCCTGGTCGCCGAGACGTTCTCCGCGCTCGTCGATGTCAGAGTGGACGAGCGCCTCTCCGCCGGCACCGGTCCCGGTCTCGTCGCCGTCCCGCCGCCTGGATCGCGCCGCCGCGTGAGTACCTCCGATCTGTTATTAGGGAAATGTGTTTCTTTTGGTCCGCCCTACGCGAATCGTGGGGAATCACGCCCCCTCCCTCGCGGATAATTCCCGGATCTAAATCGTGAAGGACGTTATTTTCATGTTTTTGTTGGGACAGAAATTCGCATGATTTAAGGTTTTGTAGAGGGAATGGAACACGTTATCTACTTGTGCTTGTTTTCCTGTTGCTAGTCTACCTGTGGTTTATTGCCGCTGGCCATCCGTATGCCTGAACTGTACTACCCGATTGTAGGTTGTTTTAGGTGGACTTGTTGCAATACACTAAAAATACTGCTTCTGGATGCAGAGAGTTAGCGGGAGCACATCTGCGCGCCGCAGAAGGATGTCCCGCTCGCGATGGTTATTTCCATCGGACCAGACAGCCAATGGTGATGGGATAGAGGGCAATTTTTTCACCGATGTTGGCTTGACGAATCTCGCAGAAGGTTGCAAAGGGCTTGAAAAACTAAGTCTCAAATGGTGTACTAACATCACTTCGACTGGCCTAGTGAGAATATCAGAGAATTGTAAGAATTTGACTTCTTTGGATATCGAGGTAATGTCTTGCTACCTTCTCTGTACCATTTGATCTGTTTTTGATTTGTGAGCTCCATGTTCTGTCATGTGTGTTTTCAGAAGACTTAAAAAAATGTCTCTGTGTATTTCCCTGTATAAATGGTAAATGCCCGACGTAGAGTTTACATGCAATGCGGTCATTGAATGTAATGATATGTGTGAATTGACAGCAAGCAGGTATTCTGGTTGATTATTGCTTTATATTAATGAAAGGGAAATATTTTTGCACTACAAATTTGTAGTTGATAAAGTCTAGTTATATACTTGAAGTTCCCTCACATTTGTTGACAAAATACATCTATTTCTTTCCTCGAGCAGTCTCAGTAGTCAATGGCTAATTTTGAACAAAATATGAAGTGATATTTATCTGCAGGCTTGCTATATTGGAGATCCAGGACTTGTTGCTATTGGGGAAGGCTGCAAGCGACTTAATAATTTGAACTTGAATTATGTTGAAGGCGCAACAGATGAAGGCTTGATTGGACTAATAAAGAACTGTGGACCATCACTGATTTCTCTTGGTGTTACTATTTGTGCTTGGATGACTGATGCATCTTTACGTGCTGTTGGATCCCACTGTCCTAAACTTAAAATCCTGTCACTGGAAGCAGAACATGTTAAAAATGAGGGAGTGATATCTGTCGCTAAAGGATGTCCCTTGTTGAAATCTTTAAAGCTACAGTGTGTTGGTGCTGGCGATGAGGCTCTAGAAGCTATTGGTTCATATTGTTCGTTCTTAGAAAGCTTTTGCCTCAATAACTTTGAAAGATTTACGGACAGGTACACAGTACAATCGCACTCTAATCTGTAGAGGCACAAACTCCACTATTGTTTTGTCTGTTTGCTTGGTGTTTGTAAATTTCTGTTTGGACTTTCTATGCTAGGGCAATAATATTCCTTTCCTACCTTTTTGTTTGACCCGAATGTATGCTACCCTTTGGGCAAACTTACTTCATTAGGATGTTAGGAATACATATTGTTGTTTCCCAGTGAATTACAAATCCTGCAATTGCAGGTACCAATGACCATCATAAATTCTTGTTATCTCCGACTTTTGCTTCTTCTATTACTTTTGTATGATCTTCTAACCCATTTTGCATTTCCAGGAGCCTTTCTTCCATTGCAAAAGGTTGCAAGAATCTCACAGATCTTGTTCTCAGTGATTGCCAATTGCTAACTGATAAAAGCCTTGAATTTGTGGCACGCAGCTGCAAAAAAATAGCACGTATAAAGATCAACGGTTGTCAGAATATGGAAACTGCTGCACTGGAGCACATTGGACGATGGTGCCCGTATGTCTCTTGTTGAGAACTTGATCTGTTAAAAGTTCCCTTTCTTCAGTTGACATTTATGTAGAATTGATTATCGAATATTAATCCAGTGTTTTTTCAAGTAAGTGAACTAGTGTGTTTATCTTACATTCTTTGTCTTCCTATGAAAGCAGGGGTCTTTTGGAGCTCTCTCTAATTTACTGCCCAAGGATCCGCGATAGTGCATTTTTGGAGCTTGGTAGAGGCTGCTCCCTTCTCCGTTCTCTTCATTTAGTTGACTGTTCAAGAATAAGTGATGATGCTATCTGCCACATAGCTCAAGGTTGCAAGAACTTAACTGAACTTTCCATTCGCCGTGGTTATGAGGTACTCCCTCCGATCCTAAATTGTTGTCAAAATATTACATGTATCTAGACGCTTTTTAAGAATATATCCATCCATATTTGGGCAAATTTGAGTCAACAATTTAGGATCAGAGGGAGTAATTTCCAAGTTTGCACTGATTTACTGCAAGCCTAGCATGTAGAGATAAATTGGAACCTCTTCTGTAAATGGTGCACAACTTTCTGCCCTGATATATTCTATACCATCGCTTGCATCTCAAATATGAAATGAGGTGTGTTTAATAACTTTAGTTATAGAGGGAGAAACATGATTTGGTTGCATGGTTTCGAGAACATGATTTGGTTGCATGGTATTTGAGAAATGATTTGGTTGCATTCACATTTTTAAATTCCGAACTCAGATTTATTTGTTGATTGAATTATTATTTTTTGGTGTGCAGATAGGAGATAAAGCATTGATATCAGTTGCCAAGAATTGTAAATCACTTAAAGTGTTAACACTCCAATTTTGTGAGAGGTAAAAATTATGAAGCATCTATCCTGTCTTTTTTCTTATCTTGCCCATGCATATCAATTCTTGACTGAAATACATGTGTATGATATGTTTTTTTTTGTTGGAAAAATAGGGAACCTCACTTGCTCCATCTTTTATTAAAATGAAACCAAACCACCATCTCTATGATATGTTGACATCTCAGTTTGTGAATTTTGTTTCAACTTTTTTTTCAGGGTATCTGATACAGGGCTGTCTGCAATTGCTGAAGGTTGCTCTCTTCAAAAGTTAAACTTGTGTGGCTGCCAATTAATCACTGATGATGGACTGACTGCCATTGCAAGAGGATGTCCTGATCTTATCTTTCTGGATATAGGTGTTCTCCAGGTATATATATCTTCCGAAATTTGAGAATGCAACCAAAATGGCCTCTACTTCTATTGTTGTTGTCCAAACTAGGTTGGGGAAACCTAAAGTTGACTGTGAAGACCTTTTATCACGTGTTGGAAACTTGTTAATTAACATCCCTAGCACAAGCTGTTAGGAGTTTGGCTTACATATTAGCTCCATTCTACCAAAAGAACTTTAATGATACGAGATACTAGTCCGGATCCAACATATATTCTGTTCATAGCCTTTGATGCCGGAATCTGCATTGTAGATGTACAGAAATCTGGCGCTAGTCTTGATAGATTGTGTTTATCTTCAAGATGTGGAGATGCCCATTTTGTAATTATCCTGATTTCACAGTTCTTTCATATTTTCATGTGCAATTCTCTCGTATCCATCCATATTTAGGAAGAACTTGCATTAATTTTCTCTGCAAGTAGCATTCCAAGTTATGTTATCGTTCCTGATGATTCGCTCCCCAAACTGCAGATCATAGGAGACATGGCGCTGGCAGAGATTGGCGAAGGTTGCCCCCAGCTTAAGGAGATTGCACTCTCGCACTGCCCAGAAGTCACCGATGTCGGCCTGGGACACCTCGTCAGGGGGTGCTTGCAGCTGCAGGTGTGCCACATGGTATACTGTAAGAGAATAACCAGCACCGGAGTAGCGACTGTGGTCTCGAGCTGCCCGAGGCTGAAGAAGCTCTTCGTCGAGGAGGCTAAGGTCAGCGAGAGGACACGGCGTAGGGCAGGACCCGTCTTAGCCTTCCTCTGCACCGGGATTTAGTTCCAGAAGATAAAAATGTTGTGTTACATTAAACTCCGTCGTTTACAAATTAGCAGCTGCGCTGAAGTTGCTGGACTTCAATACCTTTATAGCTCAAAAATGTTGTATGGAACTGTTGTACCAAATCAAGATGTTGTATCCACTATGTAAACAAAATAAAACACAAGTTATGAATAAATTGGGCATTGTCCCCCTCTTATGTTACACAGGAGCAATCCGTTTGCTGCAACTATGCCTTTTTTGTTGGCCGATTTGGAGCAGAAAAAACAAAAACATCTATTTATTTTTAGCCTCTTCTCTCCTCATGTGACATGGAATTGAGATACATTCACAAGAGAGTATATATCTCTCTTGCCACACTTGGAGTTGCTTGAACCAGCAATCGATTGCTTCATGTAGCCATCTGGAATCACAAAAGAAAAC

>BdCOI7 BRADI5G08680.1

AAAATTCCCCATCTCGCTGCCGAGCGCCCCAGCAGGCCAGCCCCCCTCGCTCCGTCGCTCGTCTTCCTCCTCGCCCCATCCCGCTGCCGAGCGCCCCAGCCCCCTCGCCACCCCTCCTCCCCATCCCGCAGACGAGCGCCCCGCCCCAGCCCCCTAGCTCGTCTTCCTCAGCGCTCGCGCTCGCGCGTCCACCGCAGGTTGATCTCCCCTTCGATTCTCTCACTTTTGGGCAGAGAGATCTGGCCGGAGGGGTCGCAGTCCTCCCGCCTCATTTTGGCCAGTGTCCATTCTGGGTTGGTTGGGGCTCGCATGCCTAAATCGGGTACGGATGACAGATGCAGTAACTGCTAATATCTTTTGAAGAATCTTTAGCTGCAGATCTGGAAGCCATTTTGTGCCCTTGCTTCAGTTGGAGCCCTGCTACATGAAGTGAATGATGCTGCTTTCTGGTATCCCCTTGCCATTGGTTGTTACTTGTGTTTGTTCACCAAATCTGGCAGGAGGTGATCTGCCCAAGCCTTAGTTCCGTGCTCTGGGTAAATAAGGTGAAAGCTCCCAGCTCTTGTGTGTTGATTTCTCTGGATTTTTGAGTAACCTTGATAACCTGGAGGCCGCAAGGAAACAGATCCCACCCTGCAGCAGCTCTAGTAATAAGGTGGTACTAGGGGAAGGCACCATGACCTACTTTCCTGAGGAGGTGGTGGAGCACATATTCAGCTTCTTGCCTGGTCAACATGACCGGAACACAGTTTCGCTTGTATGCAAGGTGTGGTATGAGATTGAAAGGCTGAGCCGTCGTACTGTCTTTGTGGGGAACTGCTACGCTGTTCGTCCTGAGCGAGTAGTTCTGCGTTTCCCTAATATGCGGGCACTGACGGTCAAAGGAAAGCCACACTTTGCCGATTTCAACCTTGTGCCACCTGATTGGGGTGGGTATGCTGCTCCGTGGATTGAGGCAGCAGCCAGGGGCTGTGTGGGTCTTGAGGAGCTGCGGATGAAGCGGATGGTGGTGACGGATGAGAGCCTTGAGCTGCTAGCTAAAACATTTCCACGCTTCAGGGCTCTCATCCTAATCAGCTGCGAGGGGTTCAGCACCGATGGGCTAGCAGCTATTGCAAGTCACTGCAAGTGAGTGTACTTAAAATCTTTAAGGCATCATACTTTTCCAATTTGGTGGTGTACTTCTAGATCCTATGGTCTACAATTTTAAGAATTGAAATATGAAATTCCTATGATAATACTCTTTTCTAATAGCCGTGAGGCTTCTTGCCTTCTGTGTTGTTAGTTATGCTTCTTTTGAAATCTATCCCTCTGCATGAATGGATCGATTTTTATTTGTTGAACAGTTCATTTATCTACAGGCTCCCAAATCACAGAATGCAGGTATCTTTGCAGGGCATTCACATCTTGTTTTGTTTAAATTTGTGGTATCTGCGGTTTTATGGACTGTTGTAGAGCTTTTACATGCTAGTTTCAATTCATGTAGCATGTTTTTTATGTACTTAGTTGGCTCCTTAGGTGTTTAACACCGATCAGTTGTTCTCTTCTATACATTTTACTTGTCAGGCTGAGTTAGTGTTGTTATGCCCTGGTTACAACAATCATCCAGGATTATTGTTTTACTGTCGTGCTTATTTCGCACACAACTATTTGCCCGGGCAGAATGCAATTTTTGTTGAATTAAATTATTTAACGTGTTATTGCCCAATTTTGTTATGGCATAAACTTACTGTTACTACATAGATTTTCTGGTTAATACGCTGACATTGTTTTCTATCTTTTATCTATTGTCTAGGCTCCTGAGGGAGTTGGATTTACAGGAAAATGAAGTGGAAGACCGAGGGCCAAGGTGGCTCTCCTGTTTCCCCGATTCCTGTACATCACTTGTCTCCTTGAATTTTGCCTGCATCAAGGGGGAGGTTAATGCTGGTTCATTGGAGAGGCTTGTTGCTAGGTCCCCAAATCTCCGGAGTCTGAGATTGAATCGATCTGTACCCGTAGATACACTCTCGAAGATATTAATGCGCACCCCTAATTTGGAGGACCTTGGGACTGGGAATTTGGCAGATGACTTCCAAACTGAGTCCTACATCAGGCTGGCCCTTGCATTTGATAAATGCAAAATGCTAAGGAGTTTGTCAGGATTTTGGGATGCTTCCCCTTTCTGCCTCCCTTTCATTTATCCTGTTTGTGCGCAACTAACAGGTTTAAACTTAAGCTATGCTCCCACACTTGATTCTTCCGATCTCACTAAAATGATCAGCCACTGTGTGAAACTCCAACGCCTTTGGGTGAGTTACCTTGTTTCTGCACTCGACTCCCCATTTTTATGCTTGATTAGAACAAGGCAGTACTGATTTTGAGTCTTCATCCTTTATATTGTAGGTACTGGATTGCATTGCGGATAAGGGCTTGCAAGTGGTGGCCTCCAGTTGCAAGGATCTACAAGAACTCAGGGTATTCCCATCAGACTTCTATATTGCTGGGTATTCCCCGGTGACTGAAGAGGGACTTGTTGCAATATCCTTGGGCTGTCAAAAATTGAGCTCATTGCTCTATTTCTGTCACCAAATGACCAACGCTGCACTAATTACTATAGCTAAGAACTGCCCAAATTTCACGCGATTCAGACTCTGTATTCTTGAGCCGGGGAAGCCTGATGCCATGACAAACCAACCATTAGATGAAGGCTTTGGTGCTATTGTTCGTGAATGCAAAGGGCTAAGGCGACTGTCAATATCAGGCCTTCTCACGGATAAGGTTTTCATGTATATCGGAACATATGCAAAAGAACTTGAGATGCTTTCAATAGCATTTGCCGGAGATAGTGATGCAGGAATGATGCATGTTATGAAAGGATGCAAAAATCTGAGGAAGTTGGAGATTAGAGATAGCCCATTTGGTGATGCTGCACTCTTGGAGAATGTTGCCAAGTATGAGACAATGCGATCCCTTTGGATGTCATCGTGCAATGTCACAGAAAAGGGGTGCCAAGTCCTTGCATCAAAGATGCCAATGCTTAATGTGGAGGTCATAAATGAGCTAGATGAAAACAATGAAATGGATGAGAACCATGGAGGGCTCCCCAAAGTGGACAAACTATATGTTTACCGCACAACTGCCGGGGGAAGGGATGATGCACCAAATTTTGTTAAAATTCTATAGAGCTAGCTGAGAGTTGCCCGATATCTTATTCTGATAGGTAACCCTTTTCAAATTTTTCCTTCACTTAGAAATATCTTGCTTATTTCAAGGCTTCACTGGTACTGTTCTAGCTTCTACAACCTACTGTTTCAACTTGCATGGTTTTGTTCTTCTGCAGCTTGTTTGATCTTGTAGACATGTACTGTGTGCACAACAACATGCGGATTATTCATTAATAGGCCCTTGTGGATCATTATGTCATCTTAGATGTTTAGGTCAGAAGTAACCTAGGATAGCAGCCTCTTTTGTAGTTATCATAAGCATGCATCACCATATGGTCTTCTAACCAAAATAAGAGATGCAGTGTAAAGTGGCTTATGCTAAGGTTGTTTAAATGAAACCCCACTTATTAATGAAAGATTGAAATGATTCTTGAACCAACTTTGCTCTATGAAATCTTTGTATGCTCGTTTTGTTCTCAGTAGATTGTAGCTACCCATAGTGCTAATGTTGACCATTCATTCTGATTCCAGAGCCTGATGATATATTGCATGATATGATCCCCATCTCATGAGACTCTCCACTCGATTACTTTTACAGATGGTCGGCGGAATCGGGCAGGCCCGGGCTGACAGTGGCAAGTCCTCCGGGTGTCTCCCTCCCCTTTGCGGTGGCCAGGAAAAGGCGCATCTGTGTGCCCCTTATCTTGGAGCATCTCTGTCCGTGGATCCATCATTCCCTTTTAGCGTTTGGTCATATGGCCACCCAATGGCCCTGCCTTTACCCTGTGCGTTCTCAAGAGGACCACCAATCGGACGCCCCCAACTTCCCTTTTTTTCTTCTTCCCTGTATTCCGTAGCAGTACCTATCTCATTTGCTTTTGACATTGCCACAGTGCATCGTGCTGCTGCTGCTGTTGGCTGTTGCGCTTGTTGTTGTTGTTGTTGTTGTTGTTGTTGTTGTTGTTGTTGTTGTTGTAATGTTGTTTGTTTGAACCTTGTCCAGTTGTCCCGGAAGAACATTTCGAGGCGATGGCTAGTTTGTTGCTCCCTTGAATGTATTGAGCGCCTGGAATAATTTGGTAGTACCGTGATTTCATCATTTC

>HvCOI1 MLOC_18524.1

CCCCCCCCTCCTTTCCCTTTTCCCTTCCCACTCTACACCCGGACGCACGCGCACGGACGCCCTCCCCTCAACCTCATCCCAACCCCCGTTGAAATCCGCGGCGGGCGCAGCGCAGGCAGATCGGGGTCCTGCTCCCGGCGATGAGCGGCGACGAGCGGCACCTGGGGAGGACCATGAGCTTCGGGATCCCGGACGTGGCGCTGGGGCTCGTCATGGGGTGCGTGGAGGACCCCTGGGACCGCGACGCCATCTCGCTCGTCTGCCGCCACTGGTGCAAGGTCGACGCGCTCAGCCGCAAGCACGTCACCATCGCCATGGCCTACTCCACCACCCCCGACCGCCTCTTCCGCCGCTTCCCCTGCCTCGAGTCGCTCAAGCTCAAGGCCAAGCCCCGCGCCGCCATGTTCAACCTCATCCCCGAGGACTGGGGCGGCTACGCCTCGCCCTGGATCCGCGAGCTCTCCGCCTCCTTCCAGTTCCTCAAGGTGCTGCATCTCCGCCGGATGATTGTCTCCAACGACGACCTCGCAGTGCTCGTGCGCGCCAAGGCCCACATGCTCGTATCCCTCAAGCTTGACCGCTGCTCCGGCTTCTCTACGCCTTCCCTTGCTCTCGTCGCCCGCCGCTGCAAGTAATTTCTCCCCTCCTTGTTCCGGTTCACAAATGATATGCATGGATGCATTAGCTCGTTTATTATTAATTATTGCAGCACCTCTTTCTTGTCCTACGAATTAATTTCTAGTTTCAGTTATCCTAGTTAGTTTTTCTCTTCTGAAATTGTGTTTTGTTCTATTTGGAAATGGTTGGTGGATGGGATGTTTGTAAAGCGAGTATATATAAGGTGTTCTTTTGTTCATTTGGGAAGGGGAAAAGTAAGTGTAGCGTCTCACCTTTTCATGATGTAAAACATCGTGGCACTCTTTGTAATTTGGGAATAATTCTAGCTTTGGAGCAGGAATTAGGAAATCTGGACTTTAACTGCCTTGCTGAATTTGGGACTTTGCTTAATTGCTTATACTTTCTTAGAGAACTATGATACTTGGCAGAGATAGTTTTGGAAAGCAGATTATTGAAATGCTGTTGTAGAAGTTTAATTAGACTGCAAGAGCATACATTCATAGGTTACTGGTTTAATTAATCATATTTCTTGTCATGTGCAGGAAACTGGAAACGTTGTTTCTTGAAGAAAGCTCCGTTGCTGAGAAAGAAAATGATGAATGGCTCCGCGAGCTTGCTACCAGCAATACTGTCCTTGAGACGCTGAATTTCTTTCTGACAGATCTCAGGGCATCCCCTGCACATCTTCTCCTCCTTGTGCGAAATTGCCGAAGGCTGAAAACACTCAAGATTAGCGACTGTTTCATGTCTGACCTGGTCGACTTGTTCCGCACAGCAGAAACACTACAAGACTTTGCTGGTGGTTCCTTCGATGATCAAGATCAAGGTGGCAATTATGCTAACTACTATTTCCCTCCTTCGGTACAGCGCTTGAGTTTGCTCTACATGGGAACAAATGAGATGCAGATATTATTTCCATATGGCGCCACACTCAAGAAGTTAGACCTTCAGTTTACATTCCTTACTACAGAGGATCACTGTCAATTAGTCCAGCGCTGCCCAAATCTAGAAGTTCTGGAGGTATGTCCTGGCAAATTATTTTAAATGTTTTCGCCATATTTCCTTCCTGGAGGTCGGGTAGTGGATATGCTATCTCAAACGCGTTTTGTTGATAATTTTTTGTCTTCCAACAGGTGAGGGATGTGATAGGAGATCGAGGGTTAGAAGTTGTTGCACAGACCTGCAAGAAATTACATCGACTCAGAGTCGAGAGAGGAGACGATGATCAAGGAGGTCTCGAGGACGAACAAGGTAGAGTGACACAAGTAGGATTGATGGCTGTAGCTCAAGGCTGTCCTGATTTGGAGTACTGGGCAGTACATGTGTCTGACATTACAAATGTAGCTCTTGAGGCCATTGGTACGTTCAGCAAAAACCTGAACGATTTCCGACTTGTCCTGCTTGATAGAGAGGTGCATATAGCTGACCTGCCACTTGACAACGGGGTTCGGGCTTTGCTGAGAGGTTGCACCAAACTCCGGAGGTTTGCATTTTATGTGAGACCTGGAGCTCTATCAGATATTGGCCTTAACTATGTTGGTGAATTTAGCAAGACCGTCCGCTACATGTTGCTTGGTAATGTCGGGGGATCTGATGATGGGCTGCTGGCATTCGCACGAGGATGCCCAAGCTTGCAGAAATTGGAGCTAAGGAGTTGCTGCTTTAGTGAACGTACATTGGCAGTTGCGGCCTTACAGCTGAAGTCACTCAGATATCTATGGGTGCAGGGATACAAGGCATCTTCTACTGGCACCGATCTCATGGCAATGGTACGCCCCTTCTGGAACATTGAGTTTATTGCGCCAAATCAAAGTGAGCCTTGCCCAGAGGGTCAGGCACAGGTTCTGGCGTACTACTCTCTAGCTGGGGCAAGGACAGATTGTCCCATGTCAGTAATTCCCCTCTATCCGTCAGTCGGAAGCTAAAAAGACCATCAGTTTGACTGTACATACATGTTTGATGCCAGCAAAACCCACAATACGGCACATAGGGACATTCCATCCCACAGTGCCATTATGGAACTGAAAGCTCGACTAAAAGCGACCCCCTCTGAATTGTCTTCGTATTTGAGGGGCAACATTCCTGGGTAAGCTGCTCATCTGGCCAACATGGATATCTCTGTGTACTACACCCATTTTGACAAGGCTCGGACACACATTTTCTAATAATGTGCCCAGTTGTAATGGCATTTTTCTGTTCTTGAACCTTTGCCCACTGTATTGTTGTTCTACGAACAGTATTCCTTTAGTTGTTGTACCATCTGTAAACCTCTCTGCGCAATGTTATGACTATCTTGATATTTTGCTGTGGCTTGGCCTCAGTTTTTCTGGTCGCAATTATCCGGC

>HvCOI2 MLOC_52024.5

CTGTGCTCTAGTTTCCGGCTGCTGCTGCTGCTGCTGATCTGAAACTGAACAGCCCATATTTTGATCCAGGCCATTTGGCTACTTTTTTTTGTACAGATAGTCTGCATCCCTGCATATTCATTGTTCTAGCCGAGCCTAGTTATCGTAGCAACATGTGTGGCATGGAAATAAGCACACGTGAGACAGCCGCTTAAATTAGCACCTTCCTCTTGCTGTCCATCTACTACAGAAAACTGGAACAAAAGAAAAAAGTAACTTGAAGATGACATGCACAAAACTGAACTATGTTGTCCTCCGAGCTGCAGCTAACCAATCAGAAAGAGGCATGCCATACAAGGTACTATAAACGCGAGCCATCGCATACACCCAAATCGCACACGAACAGCGCCTCGGATCAGCATGCACACAATCAGCCAATCCACTGCCCAAGCAGATCGAACAGCTTAGGGTTGTACCTGGGGAGGAATCGGCAGCGAAGAAGACAGGGGTGCTGCTGGTCGCCGGCCTGCCTCTTGGTCCTGGCCTCCTTCGCGCTGCAGGTCAGAGAAGAAAAACAGATCAGGGGGGAGAGGGGGGGACCAGGGCAGGGAGAGAGGGNNTCCGCCGTCGCCGCCGTCAGTCATCCATGGGGACGTCCGAGGGAGAGAGGGCAGGGGGAGGCCCAAGCGGAGGAGACGGAGAGAGAGGGGAAGGAGAGGAGAGGGGCCGCACCTCGACGCGCGCCGCCGGACGCCGCCGCCGCTCCTCTGTTNNNNNNNNNNNNNNNAGAGAGAGAGAGAGAGAGGGAGGCGCTGGAGAGGGTTCTGTCAGTGGGGGCCCGCCTGTTAGTGAGAGGGGAGGAGGGGCGAGATAATAAAGGGAGGAAAAAAAAAACGTTTCGATGATAACAATAGCTCCATCGCGTTCGTTCCCCCTCTGACGGTCGTCGCACTCCGCCCGCCGAGCACTCCCACCGAGCCAGAGGAACGCCGCCGGCCACCGCCAAGGGATTTGCCTCTCACTCCGCTGTCGTCGTCGTCGTCCTCCTCCACGGCTTCTCCGCTCCGCTGTACTGGCGATCCGTCCGTCCGGACGGACGGGGACTGGTTCCTTGCTGGTGACGAACCCTAACTTGCTTGCACTGTCCGGATGATTTTGATCTCGTCTTTTCTTACCTTGCTGTGAGGTGCGAGCAGTTTAATTCATCCACTTTCCGGCGGATTCGTTGGCTGCTTGGCGAGCTGATGATAGCCGTGAGTTCCTTTTCCGGAAGAAAAAAAAAGGGGGGAAATTTGGGGTTCCAGTCCAGCCGAACCCCATGTAGCTCTTTGGGTGTCCGGTCGGAGGGCTTCTTTGCGCGGTTTAGCCCGTCGATTGTGCTCTTGGAACTCAATTTCTGCATGTTTCCGGGTTTTAGGGGGATGGTGTTGTTCGGTGATTTTCTTCCGAACACTTTCTACTTAAGGGAAGATGCAAACGTTCGTGTGCCTGTTGCTTAGTTTACTGTGTTGATTCTTGGAAATTTATGAACAAGTGCTTCTTTGAGTATGAGTATATAGCACATGTCAGGCAGTCCAGTGCCAATTTCGAGCAAATGGTCATAAACTGGTACCCGATGCTAATACAGTGTTTTGTTTCTTGTGCTTGTGTGACTGAACAGGCCACAACCGGAAGCTGTAAAAAAATAATTGTGGCTTGTTAGGATCCCGGGAGCCGGCAAGATGCCCTACTTTCCAGACGAAGTGGTGGAGCACATCCTTGGCTTCGTGTCGTCGCATCGTGACCGCAACGCTGCATCTCTCGTGTGCCACGCGTGGTACCGCGTCGAGGGCCTCACTCGCCGCTCCGTCTTCATTTCCAACTGCTATGCGGTGCGCCCTGAGCGAGTGCACGCACGTTTCCCCTGCCTGCGCTCGCTGACCGTGAAGGGCAGGCCATGTTTTGCTGACTTCAACCTTGTCCCCGCGGGGTGGGGTGCCACAGCGGAGCCATGGGTGGATGCATGTGCCCGTACATGCCCTGGCCTTGAAGAGCTCCGCCTGAAGCGGATGGTTATCACTGATGATTGCCTCAATCACCTTGCCCGTTCATTCCCCAATTTGAGGTCACTAGTCCTTGTTAGCTGTGAGGGGTTCAGCACGGATGGTCTTGCTACTATTGCCACCAATTGCAGGTCAGAATGCTACAAGCTTGGAAGTCATTTACTTTTCATGTTATGATAATATTACTGAATGTGGATGTCTCCCATTAGATGTTGAATGTTAAATGGGTTATCACCAGCATGGCCTCATAAACTGCAATCCGTATTCAACCTTCTTGCCATATCCAATTTATCGTGATGCTCTATAATTTTGTAACAGATCATGGAAGCTTGTTTATCCTTGTACAGTTTGTTGAATCTTGGATACCAGAATTTATGTTATGTCTGTTTTTGAATGGAAACCCATAAACTATCAATGTAATGTAAAAGATCCTACAGAAAGCCTTGATAAGTCCTTGGCAGTTGGCATTGCTCTATCTCCATTCCATGAGTTATGATAAACAATCATGACTGAGATTGTGGATGCACTTCAGTTTATTAAAGAAAAATCTTATTTAAGGGATGTTTCTTCAAACCAGTTTGTGCAAACTCATTTAAGAGATTGTAAATGCATTGCATACTTGGGCTTTCCATGTTTCCAGTGTTATATAATCTGCATGACTGCATGTATACATTTCGAGATCTGGTCAATATAAAAAAGTTAGGTATGCTGCATTTATATAAGTGGAATCTTGATGGCTCTTTCACCAATAACTTTAAATGATATATGTGGACTGTTGGTGATTTACATATAGCAATGTAGCATCACCAGAACATCGGAAAGAGAGGAAGGCGCTAATGTAGTAATGTTAGTTATGTCATATTATTCCAATTTAAAGGAGGATTAGAATACTCGAGGGTTCTTTCACCAGTAATTTATTGCCAGTTTCAAATATGCCTTATCTAGAGTGTCTCGACCCTCCCATCAATATAATCGAAGGGCTCACGTTTTTTCTGAAAGCAAACTGCCATGGAAACCCCTACAGTATAGATAGAAATCTAAATTCATGTCTATGAGGACTTGAACCTAGGTGGTGGGGTTGTACGTCCACTCCATAAACCTCTTGAAGAGCTCACATTTGAAGTGCTACCAACTCTTAGTAGGTAAATAATGGTAATTATTCACTATGCTTACCCATAATCATCGTACGTATTTTTTACTTTTAGTTTTGAAACTCACCACAAACTTGTTACTGTAGTTGCATAATTTGGTCTCTGCGTGTGGATTCCATGTGCCCACTGATCGGCATTTACATAAGGCTAGGGAATTTCAGCATTATAAACTGCAATTCCTTAGTACCAAGGACCGCAACGGGGTAAACACTACCTAAGGGTAGCTGCTTGTACTGGCAGTCTCAGCTTGGTCGGGGTAAAGTCAGATCCTAGGTGCAAGCGATGAACTTTGTAGGAGATAACAAATTGGTTCTTCATTCTTTAGGTGCTGTGTCATACGGCACGCCTTATATTGGTGAGCCTGCCCTCTTAAACAATGATACTCCTTAAGTCTCACACAATGAAACCTGGAGATCACAACTTGAAAGATAAAAAAAAAGTGGTTAAAGGGGGTAGTTCCCTCTCTAAACTGTGTGTTGTTGAGGTAAAACTGAGGCACCTGCGGAGGCTGCGCCTCAAACGCGGGTGGGGGAGCTCACAGGCGTGTGTTCTAGCCAACAGAACACCGCTCGGTTCTCGCAACTTCAAAGATAAGATAGTGGAAGATATCTTCTTATTAATCTTAGGTGTACATGGTTGTTACTAGCATTGTGCAGCCCACTTAATGTAGGCCCGAGGCCTCACGTTCTGATCTCCACCCATGACATTTGTAAAACCTGTAAGTACATAAGGCACTAAAAAATTGTTTCATATTTATTATTAACAATTACAGCTATTATTACACTTCCAGTAATATTTACATATTTTTCTCATGTCTAGTCATAAAGCATCATTTCCATGTGACTCCAAATTGAATTATTATAATTTATGTGACATATATTTGGTGATACCATCTTTAAGGCAATTCAATAGTTTGCGCACTGTCTGTTTGTTTTCTGTAGAATTGGTAGGTCGTTTGCATCAGGAGTCACCAAAAATGATGTCATTCGTAAACCAGATTATGTTGGTAACAATTAGAATGTTATTACCTGGCAAGATTTGTGTATCTTGGAAGTTATTGTCTCATACACATGTCCTGTTCGATGAAGATTAATTCATCTAGACCTCTCTTCAGTTTGTTAAGTGTTGCTTCAGAAAGTTCTTTCAGTAATTGCAACTCATATTACTTAATTTGTAACTCTTCATTATAGGTTTCTCAAGGAACTTGACTTACATGGGAGTCAGGTGGAGTTTCGAGGCCCTCATTGGTTTAGTTGTTTCCCCAAGCCTTCGACATCATTAGAATCCTTGAATTTTGCTTGCTTGGATGGAACAGTGAGTGCTAATGCATTGGAAAGTCTTGTTGCAAGGAGTCCAAATCTTAAAAGCTTAAGGTTAAATCGTGCAGTTCCAGCAGCTGTTTTAGCCAACATTCTTACTTCCGCTCCTAAGCTGGTGGATTTAGGTACTGGATTGGTTGCTCAAAACAATAATGCTGATGCACTCAGTCTCTACAATGCTATTCAACAATGCAGTTCTCTGAATAGTTTGTCTGGCTTTTGGGATTCTCCACGTTGGATTACTCCAGTAATTCATTATATTTGCAAGAACCTAACATGCTTGAACCTTAGCTATGCTCCAACGTTTCAGACAGCTGATCTTATTGGAGCTATTCGCCATTGTCAGAATCTCCGACACTTGTGGGTAAGGTTTACTAGCACTGGTGTTGGGGAAATTTAGCCTGTTTCTTATTATGTTGAATTACTTCATGCAGTTACTTTACTTTGTTGGTTGTGTTGTTTCACTTCAAGCCTATGAAATGAGATGTTTATATATTTGGAAGTTCTGCTTTTGTCAATCCTGTTGTACTGCAATTAGCAAGGAATGTTGCCCAGTTTGCTGTTTTAAAGTTGTACTTTCTAGGCAAGAATTTTGTTTATTTAACACACAAAATGCATTTAGTTCAACTTCTTATGTCTTCTTACTCTTTAAGAGAGGCTGCATAAGAAGTTAAGAACACACGTTTACCTGTTGACTGATTTAACGATCTAGTCTGTACTAGTATAGGCATTTGAAAATATCAGACCCTGCGAATGTGGCAAGATATAATAATCTTAAAGATACCAGCAAACTTTGGAATTACACCCTTTTTATCCCTCTTGGCGTCTTCGGTAATGCTACAATGGTTTGTAGTATGCATTTCTAGCCATGCTGTGCACAAGGAAAAGTTGTAGGTGCGAAAAATCTGTCAACTGTATTTTGAGAGTAAGAAAAAATGATGATTTAGTAATAACTTATACGGGTTGATTGATGTCAGGAAACATTGTTTAATAGCTATTGTACTTGATTGTCAGTGTTACTTAACGTGCTCGCCCACACAAACCACACACAAAGAGCTGACATAATTTTGGGTGTTTTAACATCTCTTTGTGTGCATGTTTCTGATTCCGAAGGTCCTTGTGTGGGTGTTTTAACAGCTCTGTAACTAGTATTGAAAGGTTACTTGAAGAGTTATAGTAGTGGAACACTTCTGATAAAAGGATTCTTAAGAGTAAACTTTTGTTGTTCATATGTTTCTTTCATTTGCGTTTTGTCCTTGCTGGTTGTGTCTTCTTATAAAATGTTTTAATTCACCTTGTCTTAATGGTTTTAGGTACTAGATCACATTGGCGATGCAGGATTGAAGGTTGTAGCCTCTTGTTGCCTGGAGCTGCAAGAGTTGAGGGTATTTCCTGCAAATGCAGATGTGTTAGCAAGCACTGATGTGACAGAGGAAGGGCTGGTTGCAGTATCTTCAGGCTGTCGGAAGCTAAGCTCTGTTCTCTATTCCTGCAGCCGAATGACTAATTCTGCTCTGATTACGGTGGCAAAGAATTGCTCGCGAATCACATCCTTCAGACTGCGTATCTGCTTGCATGGGTCAGTGGATGCCGTGACAGGCCAGCCACTGGATGAGGGTTTTGGGGCAATCGTCCGGTCATGCAAGGGCCTCAGGCGTCTATCTATGTCTGGCCTTCTCACGGACAGCGTGTTCCTGTACATCGGCATGTACGCTGAGAGGCTGGAGACGCTCTCTGTTGCGTTTGCAGGAGATAGTGATGATGGCATGATCTACGTGCTCAATGGCTGCAAGAATCTGAGGAAGCTGGAGATGAGAAACTGCCCGTTTGGCGACACGGCGCTTCTTGCAGGCATGCACAGGTACGAGGCGATGCGCTCTCTTTGGATGTCGTCGTGCGACATCACCCTGGGTGGCTGCAGGTCCCTCGCGGCAACCATGCCAAACCTAAACGTCGAGGTCGTCAGCCAGGTGGATGGAGTCTCGTGCGATGCAAAGAAGGTGGAGAAGCTGTATGTCTACAGGACACTCGCCGGGCCGAGGGGTGATGCCCCTGGATTCGTCTCGGCACTGTAAATTACCTGTACTATCGTGGTTAGTCGGTTTCTGTGTGGCTTGTAATTTTTTTACGGTGACTTAACCGCGGAAGAACCTGCGCGTGCTCAAGGCTAGAGTTTCGATCATCCTGCTATTTCACTTACCAAGTCGATGGAAGCAGTGTATGCTTGGTTGGTGA

>HvCOI3 MLOC_56088.1

CGGCACGCATCCAAACCAAACCTCACCGGGCCGGTCGGACCGGTCGAACCGGACCTGGACACGCCATTATTTGCCGGTTCAGCTCGCATCTCCAGCCTCCTCCAAGTCCTTACTCCTACCACTGACGAGAGAGAGAGAGAGAGAGAGAGCATTGAGCTCTTTGGATTTGTCTACCCCTCCTCTCCAACCTCCCCCCGAGCTGCCTCCCCTTCCAAAGCTCCCCACTTCTCTCGTTCCGGCGTCGCATTCGCCCCGCCCGCCCCGCCGATTTCCTCGTCGCTCACCCTAGCTCGCAGCCTCTCGTCAGCGGGGCTCCACTCCGCTCGGAGTCCCGCCCCCCGCGCCACCCGTCGAATTTCTCGACTCATTCTCTCCGGCGGCTCTGGGTGCGGGCTTTCGGTCCGGGGCGCCGCGGCCCGCGATCATTGCCGGAGGAGAGCTCTTTTCTCGGAGGGGCCTGGTGCGGAGTCTGGAGTATCCGCGGCGTGCATAGGTTAGATCTGGAGCTTTCGGCGCTCTGGATCTGAGCTGTTGATAGGCTGCATTTTTTTTATTTCCTTCTCTCCGTCGCTATCGGTTTGCTTTTTCTTGCAAGTTCCCGGAGACGGCAGGAACTCAGCGCTCGCTGATCTTTCTTTGCAGGTTTTGGTGGAGGAATCGGGACTGGCGGTCGCAATCCTCCCAACCATTTTGGTGGTGTCCGTTCTGGGTTACTAGGGGGGTTTGCGTGTCTAAATAGGGTATGGACGGCAGATGCAGTAACTACTAGCATCTCTTGAGGAAACTTGAGCCGCAGATCTGAAACCCATTTGTGCCCCTTGCTGTAGTTGTAGCCCTGCCAGATGACGTGAACGATGCTGCTCTCTGGTGCTCCCTTGCCATTGCTTGCTGCTAGTGTTTGCTCAACAAATCTGGCTGGAGGCTGATCTGCCCGAAGCCAGCATTAGTTGTATGCTCTCTGCGTCTGGTTGAGCGGGGTGAAAGGTTGCAGCTTCTTTCTGTTGATTTCGTCGGCTTTCTTTAGTAGCTTTCATAACCTGGAGATTGCAAGCAAACAGATCCCCTCCTGCAGCTCTAGTAATAAGGTGGTGCTGGGGGAAGGCACCATGACCTATTTTCCCGAGGAGGTGGTGGAGCACATATTCAGCTTCTTGCCCGCACAATGTGACCGGAACACGGTTTCACTTGTTTGCAAGGTATGGTATGAGATTGAAAGGCTGAGCCGGCGAACTGTCTTTGTGGGTAACTGCTACGCCGTGCGCCCCGAGCGTGTGGTGCTTCGGTTCCCTAATGTGCGGGCACTGACAGTGAAGGGGAAACCACATTTCGCTGATTTCAACCTTGTGCCGCCTGATTGGGGTGGGTATGCCGGACCATGGATCGAGGCAGCAGCCAGGGGCTGCGTGGGTCTTGAGGAGCTGCGGATGAAGCGGATGGTGGTGTCAGATGAGAGCCTGGAGCTGCTTGCCAAGTCATTCCCACGATTCAGGGCCCTAGTTCTTATCAGCTGCGAGGGGTTCAGCACTGATGGACTAGCAGCTATTGCAAGTCACTGCAAGTGAGTCTACTTAAAATTGCAGCTTGTTAAGGCAACATAGTTTTCTGATTTGCTGCTTTAGTTTCTGCATGCATCTAGATCTTATGGTTTAACATGGTGAGGTATTGAAATATGCAATGCCTTTCTTGCTCTTTTCTAATTAGCTACAAGCCTTCTTGCTGAAATATTTATGCTTGCATGTGCCAATCCTGGGAGGGGGCACAGCCCCCCACCCCCACCCCCACCCAGCCATGCAGTAAGCTCCGGCATTGTGCACAATTCTGTAGCTACAAACACTCCGAATTGGCGGTCCGTTCTAGTGTTAGATTCGTGTATAGTATGTCTGTTTTGTTTTTTACTTAGCTCGTTACACTTTACATTTTATCAGTTAATTTTGTCTATACTTTCTTTTTGGTACGATGAATTGGTATTGTTATGCTCATGGTGCGCACAATCTGCCAGGGCAGGATGCAAGTTCTGATTAATTTAATTAATGCATTATTATCATCTCAATTTTATAAAGGCACTAATGTAGTCTTGTTACATGCATTTAATGGTCAATACACTGACATTGTTTTCTATATTGTTATCTGGTCTAGGCTCCTGAGGGAGTTAGATTTGCAGGAAAATGAGGTGGATGATCGAGGGCCAAGGTGGCTCTCCTGCTTCCCTGATTCCTGCACGTCCCTTGTCTCCTTGAATTTCGCCTGCATCAAAGGGGAGGTCAATGCTGGTTCATTAGAGAGACTTGTTGCTAGATCCCCAAGTCTTCGGAGTTTGAGGTTGAATCGCTCTGTGTCAGTAGATACACTCTCGAAGATATTAATGCGCGCCCCTAATTTAGAGGATCTAGGGACTGGGAACTTGACAGATGACTTCCAAGCTGAGTCGTATCTCAGGCTGACCCTTGCATTGGAGAAATGCAAACTGCTGAGGAGTTTGTCGGGCTTTTGGGATGCTTCCCCTTTCTGCCTTCCATTCATCTATCATGTATGTGGGCAACTAACAGGTTTAAACTTGAGCTATGCTCCGACACTCGATTCTTCTGATCTCACCAAAATGATCAGCCACTGTGCGAAACTCCAACGTCTTTGGGTGAGATCCCTTGCTCCCGTGCTCACTTTACTGTTTTTATGCTTGATTAAGACAATTCAGCACAGGTTCTGAGCCTTTATCCTGTATGCTGTAGGTACTGGATTGCATCGGGGATAAGGGCTTGCAAGTGGTGGCCTCCAGTTGCAAGGATCTACAAGAACTCAGGGTATTCCCGTCAGACTTCTATATCGCCGGGTATTCCCCAGTAACAGAGGAGGGACTTGTTGCAATATCCTTGGGCTGTCCAAAACTGAGCTCATTGCTATATTTTTGTCACCAAATGACCAACGCCGCACTGCATACTATAGCTAAGAACTGCCCTAATTTCACGCGATTCAGACTCTGTATTCTTGAGCCTGGGAAGCCTGATGCCATGACAAACCAACCATTAGATGAAGGTTTTGGTGCCATTGTTCGTGAATGCAAAGGGCTAAGACGATTGTCAATATCAGGTCTTCTCACCGACAAGGTTTTCATGTATATTGGTAAATTTGCAAAACAACTTGAGATGCTTTCAATTGCATTTGCTGGAGATAGTGATGCGGGAATGATGCATGTTATGGAAGGATGCAATAATCTGAGGAAGCTGGAGATTAGAGATAGCCCATTTGGTGATGCTGCACTCTTGGAGAATGTTACCAAGTATGAGACAATGCGATCCCTTTGGATGTCATCGTGCAACGTCACAGAAAAGGGGTGCCAAATTCTTGCATCAAAGATGCCAATGCTTAATGTGGAGGTTATAAATGAGGTAGATGAGAGCAATGAAATGGATGAGAACCATGGAATCCCCAAAGTTGACAAGTTATATGTTTACCGCACCACTGCTGGGGCAAGGGATGATGCACCAAATTTTGTTAAAATCCTATAGAGCTAGCTGAGAATTGCCTGGTATCTTGTTAATGTAAAAGGTAATCCTTCTCGAATTATCTACTTCTTTAATTTAGAAAACATCTTGTTTGTTTGAAGAGTTTTCACTTTGAAGTTGCATTCTTTTGCTTCTCCGCAGCTCTCATATGATCCTGTAAATATGTTGTGCATGCAACACAAGATGCAAGTGATTTTTTCAAAAGATGGAAATACAGTACATGCTTGAAAATGATGTCGGAACCAACTTTATTCATCCAAATCTCAATCTACTTTTCTCACTAGATTGTAGTTAAGTCAATCATAGTGCCAACACATAGAGTCTGGCGATATATTGCATGATATGATCCCCATCTGACCTCTCCCTATACTGGATTGCTTTTACAGATGGTCGGCGGAATGGAATCCGGCCGGGGCCTGTGGACAGTGGCAAGTCTCCGGCTGCACCCCCTCCGCTTTGCAGCGCCAGGGAAAGCCACGTCAGTGTGCTCCTTATCCTGGAGCATCTCTGTCCGGGGATCCATCATTCCCTTTTTAGCGTCTGGTCATATGGCCACTGCCTTTACCCTGTGCATTGTTCTTTTTCTTAAGAGGACCACCAACCAACCAACCAATCCTTTCTTTTCCTTTTTCCTCTACTATGTTGTGTACTCCGTAGCCATTTGCTTTTGACATTGCCACAGTGGGATGTGCTTCTGCTGTCGTTCTTGTAAACGTTTGTTTGAACCTGACCAAAAGGACGGTGGCTAATGATCTTGCTCCCGTGAAAATGAAATCATGCAGCAGAGGCTGGAATAATTTGTTGCTCATCAGCCCATTACTTCATTTTCG

>HvCOI4 MLOC_67830.1

GTGGGAGTGCATGACAAATGTCCAGGGAGAGGAAGTCTGCATTGCAAATGGACTGAAAACTAATCACCCCTTCACCGAAAAAACTCCTAACTCCAACTCCGGCCATGTCTGCATCACCGTCGGAAGGCGACGAGGCCGTCATCAACGGGGTGCTGACCGACGATGAGCTCCGTGCGGTCCTCACCCGCCTGGGGCCCGAGTCGGAGCGCGACGCGTTCGGGCTCGTGTGCAGGCGCTGGCTCCGGATCCAGAGCTCCGAGCGCCGCCGCTTGCGCGCGCGCGCCGGTCCATCCATGCTGCGCCGCCTGGCAGCACGCTTCCCGGGCATTCTCGAGCTCGATCTTTCCCAATCGCCGTCCCGCTCGTTCTACCCCGGTGTTATCGACGACGATCTGAACGTCATTGCAGGGGGGTTCTGCAATCTGCGAGTCCTCGCCCTGCAGAACTGCAAAGGTGAGACCTCCTCTTTTGCCCTGATCCATTCGCGCTGTCTGTGTGCGTGAATTCACAAATTGAGAAAGCTCTCCATCTTATCAAATAAGATTTCTGATTTATTGGGTTTATTACCCTACAAAGATGGGAGAGAATAAGCAAACTGAGGCGTCTGAAGAACTTTGTTGTTGCACTTTTTTTTTTTTTGAATGAGAGGGGTTTCCCCCCCTGCCCCATTTTATTAAAATGAAGCAACATTGTCTTACATATTCCTCCATAACCACTACAGAAAGCACTAAGCAAAATAAAGACTAGGCTAAAGGCCAACAGCTTGAAAGTTTTTTCCCAGCATCCACAAGCTGGGAAGAGAATAGCAGCACTGACATAGAAACTCCTAGAGTGCTTTTTTATTACAGACCAGAGAAGGAAAGCGAGAATAAGATCAGCTACTCGTCTCCCCCACGGCCTCCCAGCGTCATCTCCATGCGATCCGAAGCAGCTCCCCATGCTTCCTGTTCAGCAACCTGATACATTCCCGCAGAAGAACTGCCTCAGCATCAGCGAACAGTATTTTCCATTGATTTAGATAAATGTCCAGCTTGGCAAGAATGCATTTTACACTTATCCAAGATCGGCCCTGAAAGCAAAATTCATTCCTTAGCTTCCAAATGCTCCACAAGGAGGCAGCAATCATCATGTTTAGAACAACATTTGTCTTAGGACTACCCCACAACAATGTAACATTATCAAAGCCTGAAATTTCTGGGATGTTAAACACCTTAGAGATAAATTCCCAAAGCTGTTTAGCAACCACACAGTCAAAGAACAGATGTTGCACTGATTCCTCACAGCTACAGAATAGACAAGTAGGATCTTCCACCGGCGTTTTTCAAGGTTATCTCTAGTTAGCACTTTATTATAAACACGTAACCGTAAAAAAATATGTATTTTTTGTGGACAAATCACCTTCCAAAGACTATCACCAATGGTAGATACCACTCCCCAAAAATTAATTTGCTCATAGAAAGATTTAACCGAGTAAGTGCCATTTGACTCCAGTTTCCAGATAGGAGAATCAGGTTCTCTTGAGAGAGGAGTTTGTTTCACCGCCTCAATCAGTTGATCCCAAAGTGCTAAACCTGCCTGATCAACACATCTCCGGAATGTCAATCTTAGGACCACCCCGTCCCAAACTTGAGCCACTGTGCATTCTGTCTGGTTACAAATTTCGAATAAGTTCCAGAATTGAACTTTAAGGGAACAGTCCCCAGCCCAAAACACCATGCCGGAAGCTAATCGATTCCCCATTTCCCAGTTTCCATTTATAAAAGATTTTCGCTCCAGAGAGAGCCCAGGTGATGCTCTTCCAAAAGGTTGAGCCACTCCCACCACCGCTCCAAAAAATATTTGGAGAGTTCACATTATACCTGTGACTGATAATTTGTTTCCAATCTCTAACCGAATCGTCCATAAAGTGCTTCCCCCATGAAGCCAACAAAGCCATGTTAAACTCCTTTATGTTAGGGATCCCTAACCCTCCAAATTCTTTCTTCCTAGAAATCTGCCCCCACCTGGCCAAGTGGTATTTATGTTTGTCACCCAAGCGCCCCCAAAAAAAGTGAGTCATTTGGGAATCGATAGCATTAATAGCCCACTTAGGAAACCTAATCATAGAGGAGATGTATTTGTCACCCAACTTTGTTGTTGCATCATTTTAACTTTGTCAAATTTCAATCGTTCAATCACAAACACATGAACAAGGAAATTTAATGAAATACAAGCTGGTAAGCCACACAATATACATGTTCGAATCCTACCAAATAACTCGGGCAGCTACAGCCACTGCTCTAATTCGAAGTGAGAAGTGAAATTTCCCAAATTTTTGGAAGGTCGTTTCCGGCGCAGGTTACATTACGTTGTCTACTGTCTTAGACTGGAGAACTCGACTCTAGTGGAGATGTTAGTCTACTGTGGGATCAGGGAGATATATGTAGCCAATAGCCATGGTTAAATGTTTGGTTCTATATGCTGGTCAGATGCAGATGTTCAGTTGTCCTCCATTGTGTTTATAGAACATGCTGATTTCTCTGATGTGGAATTCCATTTGTACTTCTTTTACAGCTCCGGGACAAAGTTTTGTTTGGTACTTTCTTGTGGTAATAACCCACCAGTTCAATTTGGGTAGTCTGGTAGAGAATTTTGAGCTGGGCCATCCGACAAAATATGTATTAATAGAACTTGTGGTCTTTGTAACATATCTCTGACACGGTGATAAGTATTGCTAATTTTTTGGAGGAAAATTAATATTGGTAATCTTCAGCACTGTGAAGTTAATAGTATTGCTAGAAGTTATGAGTTTTCGGTATTGATGCATTGACGTAGTTCACAACTGGAGCTGACATCTTTCATTGCCATTTTGGCTTCAGGCTCACAGTTTTTACTTTTTAGCATGCACTGAAACTTGTGTTTTTATTTATTTCCTTGAACCTAAATGGCACTTAGTGTTTATGGAGACTTCATGTGATAGGAATCACCAATTATCTCATTTAGGTGTGAATTGTTGGTATTTCCTTAATCTAATACATTTACATATTCTACTGTTCACGTGTGTCTTAGGTATCACTGATGTTGGAATGGTCAAATTAGGAGAAGGGCTGCCATGTCTGCAAACTCTAGATGTCTCTCACTGCAAAAAACTTAGTGATAAAGGTTTAAAGGTGGTTGCGTCAGGGTGCCGGAAGTTGAGACAGTTGCACATCGCAGGTTGTAGATTAATAACTGATAATTTGTTGCGTGCTATGTCAAAAAGCTGTTTAAACTTGGAAGAGCTTGGGGCTGCAGGATTGAACAGCATAACAGATGCTGGAATCTCAGCTCTAGCCGATGGTTGTCATAAAATGAAGTCGCTAGACATAAGTAAATGCAATAAAGTTGGTGATCCTGGAATTTGCAAAATTGCCGAGGCCTCGTCGTCATCTCTGGTGTCATTGAAACTGTTGGATTGCAGCAAAGTGGGCAATAAGTCCATCCATTCACTAGCCAAGTTCTGCTGCAACCTAGAGACTCTCATCATCGGTGGATGTCAGCATATTAGCGATGAGTCCATAGAAGCACTAGCTCTTGCCTGTTGTAGCAGACTCAGGATCTTAAGAATGGACTGGTGCTTGAAAATAACGGATGCCTCATTGAGAAGTCTGCTGTGTAACTGTAAACTTCTTGCTGCCATCGACGTCGGATGCTGCGACCAAATAACTGATGCGGCATTTCAGGGCATGGAAGCGAACTTGTTTCGGTCGGAACTGAGAGTTCTGAAGATCAACAATTGTGTTGGCCTCACAGTTCTGGGGGTGAGCAGGGTGATAGAATCTTGCAAGGCACTCGAGTACCTCGACGTCCGGTCATGTCCTCAGGTTACACGGCAGAGCTGTGAGGAAGCTGGGTTGCAGCTGCCTGGTAGCTGCAAGGTGAACTTTGAAGGTAGCTTGTCGGAGTCTGATTCATCTGTTGATAGGTTCTTCTAGGGTCAGCGATGAACTCCGATTACAGCTGTTGTGGCACTGCCCAACTTGCATGGCATGTACTGTAGAAAGTAGCAGCACATGCAATGAATAAGAAAAGGTATGAGTTGTATCAATTGGAA

>HvCOI5 MLOC_72489.1
[truncated: 227,222 more chars]
